# Integration of Transcriptomics and Metabolomics to Reveal the Molecular Mechanisms Underlying Rhodium Nanoparticles-Based Photodynamic Cancer Therapy

**DOI:** 10.3390/pharmaceutics13101629

**Published:** 2021-10-06

**Authors:** Andres Machuca, Estefania Garcia-Calvo, Daniela S. Anunciação, Jose L. Luque-Garcia

**Affiliations:** 1 Department Analytical Chemistry, Faculty of Chemical Sciences, Complutense University of Madrid, 28040 Madrid, Spain; amachu01@ucm.es (A.M.); egcalvo@ucm.es (E.G.-C.); 2 Institute of Chemistry and Biotechnology, Federal University of Alagoas, Campus A. C. Simões, 57072-900 Maceió, Brazil; daniela.anunciacao@iqb.ufal.br

**Keywords:** rhodium nanoparticles, photodynamic therapy, cancer, transcriptomics, metabolomics

## Abstract

Rhodium nanoparticles have recently been described as promising photosensitizers due to their low toxicity in the absence of near-infrared irradiation, but their high cytotoxicity when irradiated. Irradiation is usually carried out with a laser source, which allows the treatment to be localized in a specific area, thus avoiding undesirable side effects on healthy tissues. In this study, a multi-omics approach based on the combination of microarray-based transcriptomics and mass spectrometry-based untargeted and targeted metabolomics has provided a global picture of the molecular mechanisms underlying the anti-tumoral effect of rhodium nanoparticle-based photodynamic therapy. The results have shown the ability of these nanoparticles to promote apoptosis by suppressing or promoting anti- and pro-apoptotic factors, respectively, and by affecting the energy machinery of tumor cells, mainly blocking the β-oxidation, which is reflected in the accumulation of free fatty acids and in the decrease in ATP, ADP and NAD+ levels.

## 1. Introduction

The use of nanomaterials in cancer treatment has attracted much interest over recent years because of the wide range of possibilities that these materials can offer due to their unique properties such as their reduced size, improved physico-chemical properties and ease of surface modifications, which have shown great potential for biomedical applications [1,2,3,4]. Among the broad range of nanoparticle types, the use of metallic nanoparticles has shown great potential in the development of both new therapeutic and diagnostic tools in cancer research [5,6,7,8]. An interesting characteristic of some metal nanoparticles is their highly tunable interaction properties with external electromagnetic radiation, which is potentially useful in photodynamic therapy (PDT). PDT exploits the interaction between the nanomaterial and the electromagnetic radiation to induce cell damage and prevent tumor growth [9]. In addition, because the irradiation can be performed in a perfectly confined area, where the tumor is located, the use of metallic nanoparticles as photosensitizing agents in PDT constitutes a highly selective approach to tumor treatment, thus minimizing side effects on healthy cells [10,11]. Among the different types of nanoparticles that have been proposed as photosensitizing agents for PDT, the use of rhodium nanoparticles (RhNPs) is particularly promising due to their low toxicity over a wide range of concentrations when near-infrared (NIR) irradiation is not applied [12]. However, despite the good results obtained, it is necessary to further investigate the molecular mechanisms responsible for the observed anti-tumoral effect, as a prior step to their evaluation in pre-clinical models.

Transcriptomics and metabolomics are powerful discovery analytical strategies that allow for the characterization of biological systems at the molecular level [8,13]. Transcriptomics provide a set of altered mRNAs between two or more studied conditions, whereas metabolomics generate a differential snapshot of the metabolome of those same conditions. Thus, the integration of the results derived from both omics provides complementary and valuable information on the mechanisms and processes involved in the system under study, providing a global picture that cannot be obtained by applying a single omics platform [14]. 

The most common analytical tool for performing a transcriptomic assay is the use of mRNA microarrays [15], which simultaneously provide relative quantification of thousands of transcripts. By using different fluorescent probes, this technique allows the identification of transcripts that exhibit differential expression between control and different conditions. On the other hand, metabolomics can be carried out in two ways: targeted metabolomics, to validate or to obtain information about a particular metabolic pathway; and untargeted metabolomics, to discover altered metabolites in the system under study without any pre-existing insights [14]. Both metabolomics approaches are mainly supported with the use of mass spectrometry due to the high sensitivity and selectivity that this technique provides. 

Therefore, we designed a multi-omics approach based on the use of transcriptomics and both untargeted and targeted metabolomics, in order to further investigate the biomolecular mechanisms underlying the photodynamic effects induced by RhNPs in combination with NIR irradiation. The results obtained by both approaches were mutually supportive and have allowed us to better understand such mechanisms, thus supporting the good performance of RhNPs as novel photosensitizing agents in PDT against cancer.

## 2. Materials and Methods

### 2.1. Materials

Dulbecco’s modified Eagle medium (DMEM), fetal bovine serum (FBS), phosphate-buffered solution (PBS), 100X penicillin–streptomycin and 0.05% trypsin–EDTA were purchased from Gibco^®^ (Paisley, Scotland, UK). Diphenylisobenzofuran (DPBF), dimethylsulfoxide (DMSO), N,O-Bis(trimethylsilyl)trifluoroacetamide (BSTFA), trimethylchlorosilane (TMSC), methoxyamine hydrochloride, pyridine, 4-phenylbutyric acid, sodium chloride (NaCl), ammonium bicarbonate (NH_4_HCO_3_), trifluoroacetic acid and formic acid were purchased from Sigma-Aldrich (St. Luis, MO, USA).

### 2.2. Synthesis of RhNPs

RhNPs were synthesized as previously described [12]. Briefly, PVP (MW = 10,000; 53 mg) was solubilized in MilliQ water (18.2 MΩ cm; 1 mL) and then added to ethylene glycol (10 mL) in a round-bottomed flask. The mixture was heated at 196 °C before RhCl_3_ (10.5 mg) was added and then kept under reflux for 1 h. The solid was obtained by centrifugation, washed several times with EtOH:H_2_O, dried, and finally resuspended in MilliQ water. A TEM micrograph of the RhNPs used is shown in Supplemental Figure S1.

### 2.3. Cell Culture and Exposure Conditions

Human cervix cancer cells (HeLa) were obtained from the American Type Culture Collection (ATCC) and maintained in DMEM supplemented with FBS (10%, *v*/*v*), penicillin (50 U mL^−1^), and streptomycin (50 U mL^−1^) at 37 °C and 5% CO_2_. The cells were treated with RhNPs at 5 mg L^−1^ and incubated for 24 h. The culture medium was then removed and replaced with fresh DMEM, and cells were irradiated for 10 min with 2.5 W cm^−2^ 800 nm radiation from a Tsunami 5000 mW femtosecond laser oscillator (SpectraPhysics, Andover, MA, USA). The cells were then incubated for 48 h at 37 °C under 5% CO_2_. These exposure conditions were selected based on a previous study in which it was demonstrated that RhNPs were successfully internalized after 24 h incubation [12]. In addition, the post-irradiation incubation time was set as 48 h, representing the optimal conditions to produce an observable and reproducible response in cell viability after the treatment.

### 2.4. Singlet Oxygen Generation Assay

The ability of RhNPs to generate singlet oxygen after NIR irradiation was evaluated using diphenylisobenzofuran (DPBF) as a probe. Oxidation of DPBF by singlet oxygen was monitored spectrophotometrically by measuring the decrease in absorbance after NIR irradiation [16,17,18]. DPBF was dissolved in DMSO and mixed with an aqueous suspension of RhNPs to a final concentration of 60 μM and 5 mg L^−1^, respectively. Then, 200 µL of the mixture was added into P96 wells and exposed to NIR, as mentioned above. Spectra were immediately obtained, and absorbance was measured at 430 nm using a NanoDropTM One spectrophotometer.

### 2.5. Transcriptome Profiling

Cells were seeded in culture plates for 24 h, and then treated with RhNPs and NIR irradiation as previously described. Control (untreated) cells were seeded in parallel and incubated at 37 °C and 5% CO_2_ for the same period of time. After exposure time, total RNA was extracted using the Purelink™ RNA MiniKit (Invitrogen™) and mRNA expression was performed using Clariom™ S Human Assay according to manufacturer’s instructions. Briefly, cells were trypsinized, centrifuged and the supernatant was removed. Cells were lysed with lysis buffer containing 2-mercaptoethanol. Lysates were then centrifuged, and one volume (1:1) of 70% (*v*/*v*) ethanol was added to the samples. Up to 700 μL of this volume was transferred to a conical tube and centrifuged. Afterwards, samples were rinsed with washing buffer and RNase-free water was added to the spin cartridge prior to centrifugation. The purified RNA was stored at −80 °C for further analysis. RNA quality and quantity were measured in a NanoDropTM One spectrophotometer prior to the analysis. Samples were processed with GeneChip^®^ WT PLUS Reagent Kit (Applied Biosystems, Waltham, MA, USA), hybridized with Clariom™ S Array human (Applied Biosystems) and scanned with a GeneChip^®^ Scanner 3000 7G (Applied Biosystems). Raw data were processed with a robust multi-array algorithm (RMA) included in the Transcriptome Analysis Console (Applied Biosystems) for normalization and gene level analysis. For each experimental condition, three microarray experiments corresponding to three independent biological replicates were processed and analyzed. Fold changes between experimental conditions were calculated as a quotient between the mean of the gene expression signals. Statistical analyses were performed with e-bayes limma included in the Transcriptome Analysis Console (Applied Biosystems). Hierarchical clustering analysis (HCA) and heat maps were performed and illustrated using the ClustVis software (https://biit.cs.ut.ee/clustvis/) (accessed on 1 December 2020) [19]. Logarithmic transformation and unit variance scaling of raw data were performed prior to the analysis, and HCA Ward’s method was then carried out.

### 2.6. Untargeted Metabolomics Analysis

#### 2.6.1. Extraction of Metabolites

Seven replicates of each studied condition, samples treated with RhNPs and NIR irradiation and untreated controls, were prepared. Intracellular metabolite extraction was performed following a previously optimized method [13]. Briefly, cells were washed with 0.9% (*w*/*v*) NaCl and placed on ice. Chilled methanol (−20 °C) and ultrapure H_2_O were added (1:1) and cells were collected and transferred to an Eppendorf vial. Then, the same volume of CHCl_3_ was added and samples were sonicated with 20 pulses (2 s on, 5 s off, 30% amplitude) and centrifuged for 5 min at 4 °C and 16,000× *g*. After centrifugation, two well-differentiated liquid phases were obtained: 100 µL of each was transferred to separate GC glass vials and 2 µL of 50 µg mL^−1^ 4-phenylbutyric acid was added as an internal standard. Samples were evaporated to dryness under a dry nitrogen stream. Bradford assays were performed to determine the total protein content of each sample for further sample normalization. 

#### 2.6.2. Metabolite Derivatization

Before GC-MS analysis, samples were derivatized in a two-step procedure. First, 30 µL of methoxyamine hydrochloride 40 mg mL^−1^ in pyridine was added to each sample and incubated for 90 min at 37 °C while shaking at 500 rpm. Secondly, 60 µL of BSTFA 1% (*v*/*v*) TMCS was added, and samples were then incubated for 60 min at 60 °C and 500 rpm. Finally, samples were filtered through 0.22 µm PTFE membrane filters, transferred to glass inserts and analyzed by GC-MS. Quality control (QC) samples, prepared to monitor and assess the stability and reliability of the metabolomics approach, were analyzed every 5 analytical samples. QC samples were treated identically to the analytical samples during vacuum drying, resuspension, instrumental analysis and data processing. 

#### 2.6.3. GC-MS Analysis Conditions

Samples were analyzed in a gas chromatograph (7890A, Agilent, Santa Clara, CA, USA) coupled to a time-of-flight (TOF) high-resolution mass spectrometer (GCT premier Micromass, Waters, Milford, MA, USA). All samples were analyzed using a 30 m × 0.25 mm × 0.25 µm ZB-5MSplus capillary column (Phenomenex, Torrance, CA, USA). Helium was used as a carrier gas at 1 mL min^−1^. Two microliters were injected in split mode at a 1:10 split ratio. The inlet temperature was set at 270 °C and the oven temperature gradient was set from 60 °C to 325 °C. The total gradient run time was 50 min. The ion source was an electron ionization (EI) model, and the scan mass range was set from 50 to 800 m/z. Chromatograms were obtained in total ion current (TIC) mode.

The mass spectrometer was tuned and calibrated for mass resolution and mass accuracy on a daily basis using authenticated reference standards. Process coefficients of variation involving instrument performance, chromatography and mass calibration were checked to ensure quality. Ultrapure MS-quality water blanks and solvent blanks were also included in the analyses and used to assess the process contribution to signals and for the identification of potential sources of contamination. 

#### 2.6.4. Data Treatment and Statistical Analysis

Mass Lynx software was used to analyze the chromatographic data. The peak area of each metabolite was normalized using the peak area of the internal standard. Metabolite concentrations between samples were normalized based on the total protein content. Identification of the metabolites was carried out using the NIST MS search 2.0 library and considering the mass spectra and the accurate mass. Representative TIC chromatograms from control and treated samples can be found in Supplemental Figure S2.

Principal component analysis (PCA), a non-supervised patter recognition technique, was performed on the set of auto-scaled data using the Unscrambler software (9.7 version). For this, we considered the mean and standard deviation values for each [20]. Excel software was used to carry out the Pearson´s correlation analysis. A total of 48 metabolites as variables and controls and HeLa cells treated with RhNPs and exposed to NIR were used to build the data matrix. The univariate approach for comparison of metabolite peak areas between the two experimental conditions was based on a two-tailed Student´s t test. A *p*-value < 0.05 was established as statistically significant. 

### 2.7. Targeted Metabolomics Analysis

#### 2.7.1. Extraction of Energy-Related Metabolites

For the extraction of the energy-related metabolites, cells were first washed with 0.9% (*w*/*v*) NaCl and placed on ice. Methanol and 2% (*v*/*v*) formic acid were then added prior to cell harvesting. Cells were transferred to an Eppendorf^®^ vial, vortexed, and incubated for 3 min on ice. Finally, 15% (*w*/*v*) NH_4_HCO_3_ was added, and samples were vortexed, incubated for 20 min on ice, and centrifuged for 10 min at 4 °C. Supernatant was collected and filtered using 0.22 µm PTFE membrane filters into LC-MS glass vials. Bradford assay was performed to determine the total protein content of each sample for further sample normalization. Five replicates of each studied condition, samples treated with RhNPs and NIR irradiation and untreated controls, were used. 

#### 2.7.2. LC-QqQ-MS Analysis Conditions

The analysis was performed in a LC-QqQ-MS instrument (LC/MS-8030 Schimazdu, Kioto, Japan) equipped with an electrospray ionization source (ESI) working in negative ionization mode. The quantification of the four metabolites: ATP, ADP, NADH and NAD^+^ was carried out in Multiple Reaction Monitoring (MRM) mode, as previously described [13]. The MRM transitions used for quantification and confirmation of the identity of each analyte are described in Supplemental Table S1. MRM chromatograms of each analyte from both control and treated samples can be found in Supplemental Figure S3.

#### 2.7.3. Statistical Analysis 

Differences in ATP, ADP, NADH and NAD^+^ contents between control and cells treated with RhNPs and exposed to NIR were assessed by ANOVA statistical assays at 95% confidence level (*p*-value < 0.05), followed by Bonferroni’s test.

## 3. Results

### 3.1. Singlet Oxygen Generation Assay

**Figure 1:**
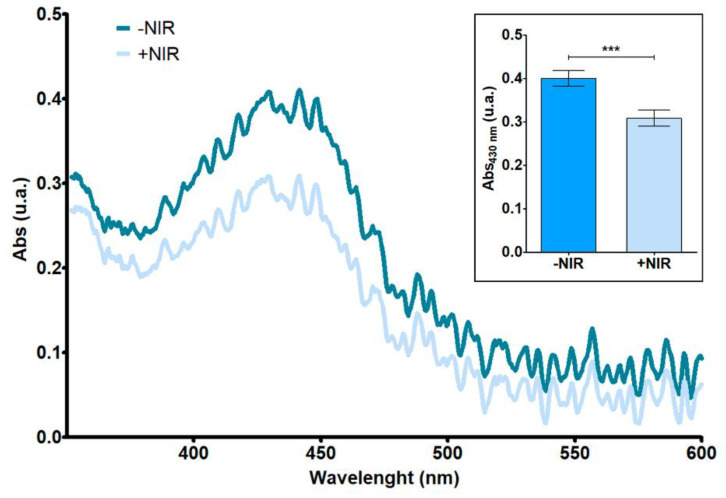
Molecular absorption spectra of an RhNP suspension using DPBF as a probe with and without NIR irradiation. Absorbance measured at 430 nm for each condition (inset). Results are plotted as the mean ± relative standard deviation (*n* = 3). Data were analyzed by t-test (*p* < 0.05). Statistical significance: *** *p* < 0.001.

Photodynamic performance of RhNPs was confirmed by the decrease in absorbance of DPBF after NIR exposure of an aqueous RhNPs suspension (Figure 1). A decrease of approximately 30% of the absorbance of the DPBF containing solution exposed to NIR irradiation was confirmed, thus indicating the oxidation of the probe due to the generation of singlet oxygen species in the nanoparticle suspension when NIR irradiation was applied.

### 3.2. mRNA Expression Assay

**Figure 2:**
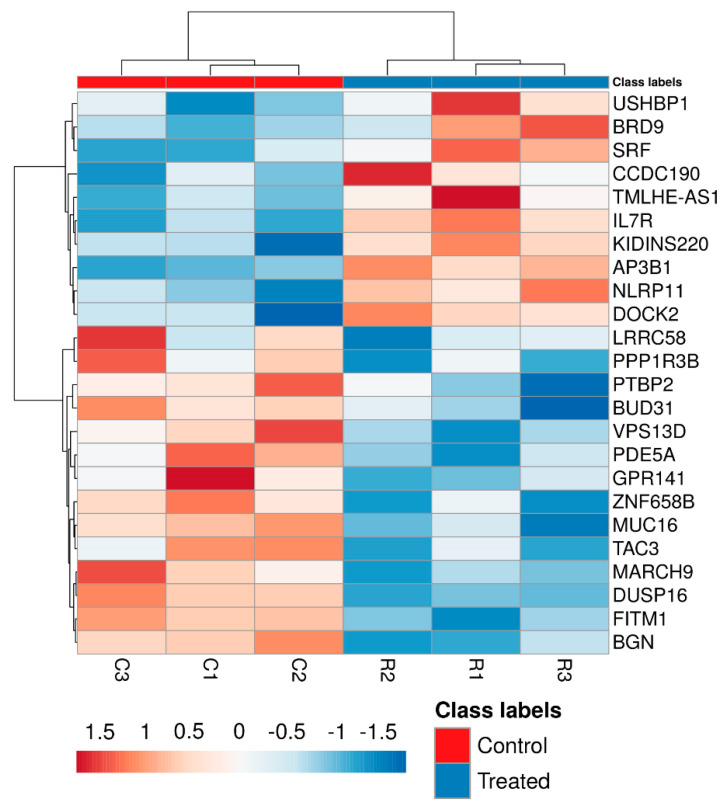
Hierarchically clustered analysis and heat map of differentially expressed genes on control and exposed cells (*p* < 0.05). Gene expression levels are represented by colors. Rows represent genes, whereas columns represent samples.

To evaluate potential alterations in the mRNA expression levels of HeLa cells treated with RhNPs and NIR irradiation, a transcriptome microarray analysis was performed. Among the more than 20,000 well-annotated human genes analyzed (Supplemental Table S2), a total of 24 genes were found to be de-regulated (Table 1) in HeLa cells treated with RhNPs and exposed to NIR irradiation. Fold change thresholds of 0.67 and 1.5 were established for determining significantly altered genes. From those differentially expressed genes, 10 presented overexpression (FC > 1.5) and 14 were found inhibited (FC < 0.67) in treated cells as compared to control untreated ones. Two groups of both overexpressed and inhibited genes were well defined by the hierarchical clustering and heat map graphical representation (Figure 2) in accordance with the microarray results.

### 3.3. Untargeted Metabolomics 

An untargeted metabolomics approach based on the use of gas chromatography time-of-flight mass spectrometry (GC-TOF-MS) was used in order to evaluate changes in HeLa cells induced by the treatment with RhNPs and NIR irradiation, at the metabolome level. After GC-TOF-MS analysis, a total of 48 common metabolites were identified between controls and samples treated with RhNPs and NIR irradiation (Supplemental Table S3) using the NIST library. A minimum NIST Rmatch value of 700 was considered for unequivocal identification of the identified metabolites: fatty acids, sugars, amino acids, organic acids and small molecules. The relationship between metabolites was evaluated on a basis of Pearson’s correlation indexes. The Supplemental Table S4 shows the significant correlations for all identified metabolites in bold. The significance of Pearson correlations among the metabolites was considered for values above 0.5328, which was the lowest value of the Pearson’s correlation matrix with a *p*-value < 0.05 (95% confidence). These values, highlighted in Supplemental Table S4, were positively correlated and presented a directly proportional relationship except for the pair of metabolites which presented a negative correlation: L-isoleucine and 11-trans-octadecenoate (r = −0.7757), so that as one concentration increases, the concentration of the other should decrease [21]. According to the significant value (0.5328), many metabolites would be significantly correlated; however, in order to set a more stringent criteria, only the ones whose Pearson’s correlations were higher than 0.9 stood out because of the high positive correlation. They were propionate and oleate (r = 0.9268); propionate and decanedionate (r = 0.9681); cytein and glucose (r = 0.9329); D-xylofuranose and dehydroabietate (r = 0.9108); pyrimidine and N-pentadecanoate (r = 0.9257); oleate and decanedionate (r = 0.9644); mystirate and monopalmitoglycerol (r = 0.9350); mystirate and cholesterol (r = 0.9083); and monopalmitoglycerol and cholesterol (r = 0.9196).

Considering the average normalized areas for the identified metabolites in at least half of the replicates, a Student’s t statistical test (*p*-value < 0.05) was performed. This statistical analysis enabled the detection of significant differences in metabolite concentration levels between control and treated samples. Among all the common metabolites, a total of 16 appeared significantly altered (Table 2), from which 11 presented higher concentrations in treated cells whereas the other 5 showed higher levels in control cells. Retention times and Rmatch values of each altered metabolite are also shown in Table 2, which assure their accurate identification. RM has been defined as the ratio of the normalized mean areas of each metabolite between treated and control cells.

**Figure 3:**
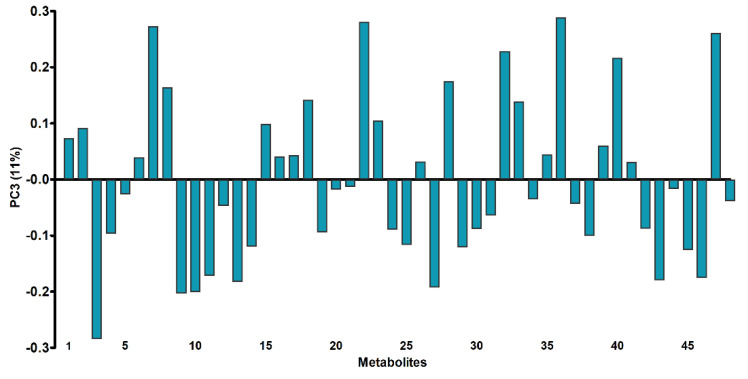
PCA results from the GC-MS data. Loadings plot of the third principal component (PC3) for the 48 metabolites (the number of each metabolite correlates with the identified metabolites as shown in Supplemental Table S3).

The PCA technique enabled the exploratory analysis of cells treated with RhNPs and NIR irradiation as compared to control cells. This approach allows, on the one hand, to group samples into clusters based on the distribution of loading and scores, and on the other hand, to identify outliers in the datasets [22]. Although the first and second principal components accumulated 49% of the total variance, no separation was identified between the control and treated cell groups due the natural variability of this biological samples. The criterion for taking into account the third principal component, which contained only 11% of the information, is the evident separation of the groups from score values, which were all negative for controls and all positive for treated cells (Supplemental Table S5). The analysis of loadings vectors showed that the distinctly separated pattern on the PC3 was in well agreement with the altered metabolites identified in the metabolomics experiment by GC-TOF-MS. Among the 16 altered metabolites, the separation in the PC3 had the highest contribution related to 13 of them (Figure 3). The inhibited serine (3), nonanoate (9) and fructose (27) that presented negative loadings and the over-expressed metabolites: propionate (7), malate (15), creatinine (18), pyrimidine (22), galactose (28), N-pentadecanoate (32), gluconate (33), myo-inositol (36), oleate (40), decanedionate (47), which presented positive loadings.

### 3.4. Targeted Metabolomics

**Figure 4:**
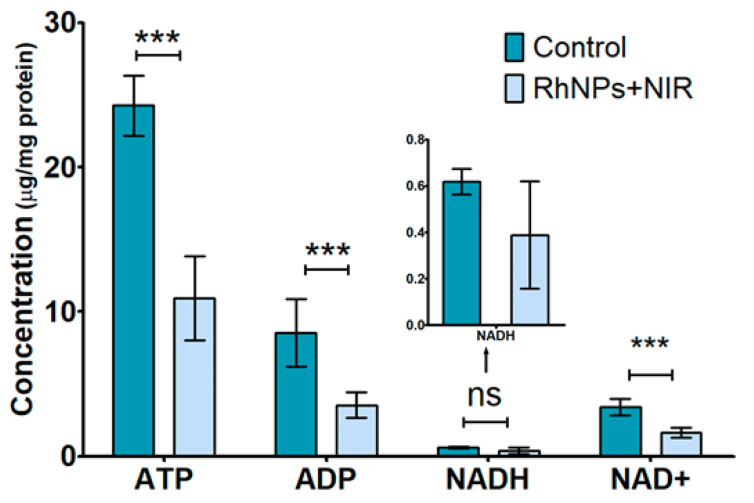
Concentration levels of ATP, ADP, NADH and NAD+ found in control and HeLa cells treated with RhNPs and NIR irradiation. Results are plotted as the mean ± relative standard deviation (*n* = 5). Data were analyzed by ANOVA followed by Bonferroni’s multiple comparison test. Statistical significance: ns = not significant and *** *p* < 0.001.

In this approach, intracellular levels of the main energy-related metabolites ATP, ADP, NADH and NAD^+^ were evaluated. For this purpose, LC-QqQ-MS working in MRM mode was selected as analytical platform due to the high sensitivity and selectivity provided. Analytical features of the LC-QqQ-MS method employed were optimized in a previous study [13]. The four metabolites were quantified in control cells and cells treated with RhNPs and NIR irradiation (*n* = 5). Each concentration was normalized according to the total protein concentration of each sample, determined by a Bradford assay. Statistical ANOVA assays were conducted with a 95% confidence level (*p*-value < 0.05) and Bonferroni’s post-test was performed. Significant differences between ATP, ADP and NAD^+^ levels in control and treated cells were observed (Figure 4). The concentrations of these three metabolites were significantly lower (less than 50%) in cells treated with RhNPs and NIR irradiation as compared to untreated (control) cells. However, differences in the concentration of NADH levels were not significantly different, due to the high variability observed in the NADH levels of treated cells.

## 4. Discussion

In the present study, the performance of RhNPs in photodynamic therapy was confirmed by the detection of singlet oxygen generated when the nanoparticles were subjected to NIR irradiation. HeLa cells were used to evaluate the potential of RhNPs in photodynamic therapy against cancer at both transcriptomic and metabolomic levels. 

**Figure 5:**
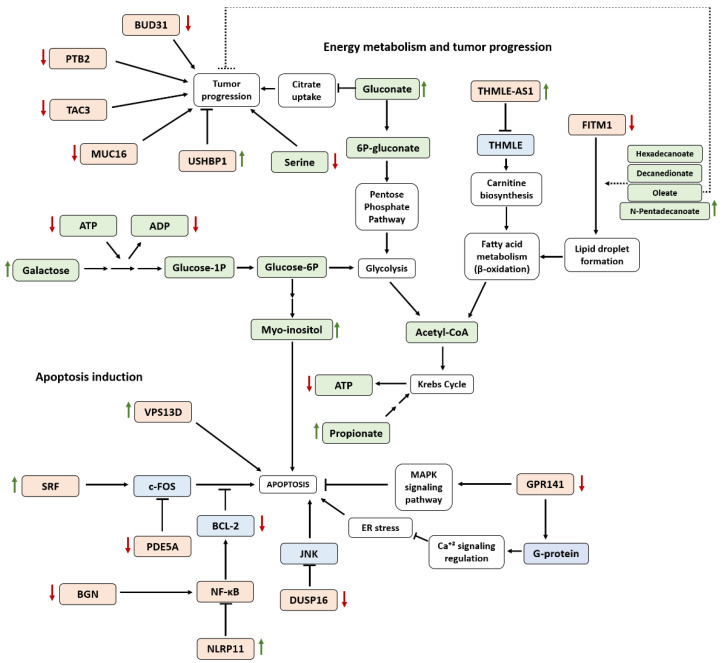
Proposed scheme of the biomolecular mechanisms associated with the anti-tumoral effect induced by the treatment with RhNPs and NIR irradiation provided by both genomic and metabolomics experiments. Colors indicate proteins (blue), genes (pink), metabolites (green) or cellular processes (white).

The transcriptomics assay showed 24 deregulated genes in cells treated with RhNPs and NIR irradiation (Table 1). Several of these genes are involved in apoptosis-regulation pathways (Figure 5). Apoptosis is a key regulator mechanism of physiological growth control and regulation of tissue homeostasis that has attracted much attention over the years. The understanding of apoptosis has provided the basis for novel targeted therapies that can induce death in cancer cells or sensitize them against established cytotoxic agents and therapies. In fact, some types of cancers are characterized by several defects in apoptosis leading to immortal clones of cells [23] and, together with deregulated cell proliferation, constitute the minimal common platform upon which all neoplastic evolution occurs [24]. In a previous study, several anti-apoptotic proteins such as Bcl-2 or XIAPs were found to be inhibited upon RhNP treatment, suggesting that apoptosis was induced [12]. The expression of those proteins has been reported to be regulated by means of nuclear factor-kappa B (NF-κB) downstream signaling. NF-κB signaling is activated by BGN (FC = 0.61) [25], whose expression was found significantly diminished. Furthermore, the NLRP11 (FC = 1.56) gene, which was upregulated in the transcriptomics experiment, has been proposed as a novel inhibitor of NF-κB [26], thus confirming the inhibition of this cell survival pathway. Moreover, apoptosis inducer c-FOS may be activated due to both the upregulation of SRF (FC = 1.51) [27,28] and the inhibition of PDE5A (FC = 0.65) [29,30]. This last gene regulates the degradation of cGMP, a second messenger that controls cell growth and apoptosis. Elevated PDE5 levels have been associated with tumorigenesis in multiple cancer types, such as colon, pancreatic, prostate, lung or breast carcinoma. Additionally, G-protein-coupled receptors (GPCRs) comprise a large family of cell-surface receptors that regulate many cell functions, including cell proliferation, survival and motility, and have recently emerged as key players in tumor growth, angiogenesis and metastasis [31]. We have found a significant depletion of GPR141 (FC = 0.57) after cell exposure to RhNPs and NIR irradiation. G-proteins activated by GPCRs also participate in calcium signaling regulation [32], and it is known that alterations in Ca^2+^ homeostasis leads to endoplasmic reticulum (ER) stress, resulting in cell death by apoptosis. Additionally, mitogen-activated protein kinases (MAPKs) are a family of proteins that form an intracellular signaling network that responds to various stimuli. These signaling pathways include extracellular signal-regulated kinase (ERK), p38, and c-Jun NH2-terminal kinase (JNK) and regulate a variety of cellular activities including proliferation, differentiation, survival and death [33]. Interestingly, we found a significant inhibition of DUSP16 (FC = 0.65), which specifically regulates c-Jun NH2-terminal kinase (JNK) by direct dephosphorylation (deactivation) [34]. JNK downstream generally results in the activation of apoptosis [35] and some studies have proven that non-phosphorylatable mutants of c-Jun confer resistance to apoptosis [36].

Some of the altered transcripts identified after treatment with RhNPs and NIR irradiation were related to cancer progression and survival (Figure 5). This is the case of the downregulated genes BUD31 (FC = 0.60), MUC16 (FC = 0.65), PTB2 (FC = 0.65) and TAC3 (FC = 0.62). The first has been proven to be necessary for the survival of MYC-driven cancers [37], whereas several studies have confirmed the role of MUC16 in cancer progression. Although its exact role is still unknown, its depletion or knockdown prevents cancerous cell proliferation [38,39]. Moreover, PTB2 is considered an oncogenic splicing factor that originates the upregulation of the SRSF3 proto-oncogene, and its inhibition affects cancer cell development [40]. Finally, TAC3, which was also observed to be inhibited in our experiment, has been found overexpressed in tumors such as oral squamous cell carcinoma and seems to play a key role in tumorigenesis [41]. Another fact that suggests the anti-tumoral potential of the proposed treatment was the upregulation of USHBP1 (FC = 1.50), also known as MCC2; this gene is not expressed in different kind of tumors and, together with its homologue MCC1, is considered a tumor suppressor [42]. 

In addition to the altered genes, some of the metabolites found over-expressed (Table 2) have been proven to show tumor growth inhibition by themselves and have been studied as potential therapeutic agents (Figure 5). Such is the case of gluconate (R_M_ = 2.66), oleate (R_M_ = 2.65) and myo-inositol (R_M_ = 2.93). Gluconate blocks the citrate uptake by means of the plasma membrane citrate transporter (pmCiC), which is advantageously used by cancer cells for their metabolism. Different forms of gluconate showed a synergy when administered in combination with other chemotherapeutics [43]. Myo-inositol plays a role in the regulation of diverse cellular processes, including calcium mobilization, vesicular trafficking, chromatin remodeling, DNA repair, and the nuclear export of mRNA [44]. Some studies have proven that the oral administration of myo-inositol has several benefits in cancer treatment [45,46]. In the same manner, oleate has been shown to increase the antitumoral effects of some used chemotherapeutic agents [47]. On the other hand, serine (R_M_ = 0.14) was found depleted in cells after treatment with RhNPs and NIR irradiation. Interestingly, serine-starving conditions have been proposed as an inhibitor of tumoral growth [48,49]. In fact, elevated serine levels act as an adaptative response to oxidative stress [50], thus promoting cell survival in stress conditions.

Several altered genes and metabolites were also associated with energy-acquiring cellular mechanisms (Figure 5). Growth signaling, driver gene activation, translation machineries including DNA/RNA synthesis enzymes and biomolecule production require energy consumption. Therefore, due to the elevated ratio of cell growth, cancer cells require a major regular source of energy [51]. Currently, the consensus in the cancer metabolism field is that tumor cells robustly engage in both glycolysis and mitochondrial metabolism to provide ATP and NADPH, which are essential for cell proliferation [52]. Interestingly, the levels of ATP were lower in cells treated with RhNPs and NIR irradiation as compared to control cells (Figure 4). De-regulation of the energy metabolism was confirmed by alterations of additional energy-related pathways such as β-oxidation of fatty acids. Such is the case of the upregulation of THMLE-AS1 (FC = 1.52); this antisense mRNA blocks the transcription of the trimethyl lysine dioxygenase (THMLE), which plays a key role in carnitine biosynthesis. Carnitine is a key metabolite during fatty acid oxidation (β-oxidation), a more cost-effective energy-acquiring pathway than glycolysis [53]. Furthermore, lipid droplet formation seemed to be inhibited due to the downregulation of FITM1 (FC = 0.64), which plays a central role in fatty acid storage and downstream metabolism [54]. Those findings, together with the increased levels of intracellular long-chain carboxylic acids such as hexadecanoate (R_M_ = 3.86), decanedionate (R_M_ = 3.66), oleate (R_M_ =2.65) and n-pentadecanoate (R_M_ = 1.92), could suggest an impairment of fatty acid metabolism by blocking the β-oxidation pathway, because reduced lipid oxidation has been proven to lead to free fatty acid (FFA) accumulation [55,56]. Furthermore, the imbalance of the energetic pathway has also been reflected by the lower concentration levels of three key energy-related metabolites such as ATP, mentioned above, ADP and NAD^+^ in cells treated with RhNPs and NIR irradiation. Low levels of NAD^+^ might be directly related with the toxicity exerted by the treatment, considering that NAD^+^ plays a role in both calcium homeostasis and inhibition of ROS; thus, its depletion might induce cell death [57]. Moreover, several metabolites found at higher concentrations in cells treated with RhNPs and NIR irradiation are closely involved in alternative mechanisms generally activated in energy-deficient cells. Such is the case of propionate (R_M_ = 3.26). This metabolite acts as a substrate for the glycolysis because it can be transformed into propionyl-CoA. This pathway produces oxaloacetate, which can be further transformed into glucose [58]. Galactose (R_M_ = 14.13), which was observed to be highly over-expressed, could act in the same way as an alternative source of energy, because it can be metabolized by means of the Leloir pathway, which allows it to undergo glycolysis [59]. However, galactose metabolism requires ATP consumption at its first stages [60,61] and might not be the best option in an energy-deficient situation. Nevertheless, excess galactose levels have shown to result in H_2_O_2_ or other free radical accumulations, causing oxidative damage to cells [62]. Another alternative substrate is gluconate (R_M_ = 2.66), which can be degraded through the pentose phosphate pathway [63]. In summary, these altered genes and metabolites appear to reflect a mitochondrial impairment that affects all energy metabolisms. This hypothesis was also supported by the inhibition of VPS13D (FC = 0.65). This gene is required for autophagy, mitochondrial size and clearance [64], and it has previously been demonstrated that knock-down or removal of VPS13D in Drosophila neurons leads to changes in mitochondrial morphology and impairment in mitochondrial distribution along axons [65]. On the other hand, several studies have related the aforementioned deregulation of fatty acids to a situation of oxidative stress [66,67], which is in agreement with the experimental results obtained that demonstrate the generation of oxygen radicals. This strongly correlates with the dysregulation of some oxidative damage-related metabolites mentioned above, such as serine [50] or galactose [68]. Moreover, creatine displays direct antioxidant properties; it is capable of removing or reducing radical and reactive species ions [69]. In this way, increased levels of creatinine (R_M_ = 1.58), a breakdown product of creatine degradation, suggest a depletion of creatine antioxidant ability.

Taking all these results together (Figure 5), it seems clear that a de-regulation of the energy production caused by the disruption of fatty acid metabolism (blockage of the β-oxidation) as a result of PDT-induced oxidative stress, occurs in cells treated with RhNPs and NIR irradiation. This, together with the favorable apoptotic situation and inhibition of tumoral progression and survival observed through both the transcripts and the metabolites altered, confirms the potential of the treatment with RhNPs and NIR irradiation as a promising anti-tumoral therapy.

## 5. Conclusions

A multi-omics approach based on the integration of transcriptomics and metabolomics has enabled a deep characterization of the molecular mechanisms involved in the potential of RhNPs used in combination with NIR irradiation for photodynamic therapy against cancer. On the one hand, the transcriptomic approach allowed the identification of differentially altered transcripts between control and treated cells, demonstrating that most altered genes were mainly related with the induction of apoptosis and the inhibition of tumor growth. On the other hand, the untargeted metabolomics approach based on GC-TOF-MS resulted in the identification and relative quantification of several metabolites mostly involved in energy-acquiring pathways. This indicates an energetic deficiency in tumoral cells treated with RhNPs and NIR irradiation, mainly due to the blockage of β-oxidation, which might ultimately preclude their survival. This energetic metabolic impairment was confirmed by a targeted metabolomics approach using LC-QqQ-MS working in MRM, in which significant alterations in the concentration levels of three energy related metabolites including ATP, ADP and NAD^+^ was observed in tumoral cells after the treatment. 

The results obtained by both omics approaches were consistent and confirmed, at both the transcriptome and the metabolome levels, the potential applicability of RhNPs as a promising novel photosensitizing agent in photodynamic therapy against cancer.

## Figures and Tables

**Table 1:** Significantly altered transcripts (*p*-values < 0.05) in HeLa cells treated with RhNPs and NIR irradiation.

Gene Symbol	Description	Fold Change
BRD9	Bromodomain containing 9	1.61
AP3B1	Adaptor related protein complex 3 subunit beta 1	1.58
NLRP11	NOD-like receptor family, pyrin domain containing 11	1.56
IL7R	Interleukin 7 receptor	1.56
CCDC190	Coiled-coil domain containing 190	1.56
KIDINS220	Kinase D interacting substrate 220	1.52
TMLHE-AS1	Homo sapiens TMLHE antisense RNA 1	1.52
DOCK2	Dedicator of cytokinesis 2	1.51
SRF	Serum response factor	1.51
USHBP1	Usher syndrome 1C binding protein 1	1.50
MARCH9	Membrane-associated ring finger 9	0.66
PDE5A	Phosphodiesterase 5A	0.65
DUSP16	Dual specificity phosphatase 16	0.65
PTBP2	Polypyrimidine tract binding protein 2	0.65
MUC16	Mucin 16, cell surface associated	0.65
VPS13D	Vacuolar protein sorting 13 homolog D	0.65
LRRC58	Leucine rich repeat containing 58	0.64
FITM1	Fat storage-inducing transmembrane protein 1	0.64
TAC3	Tachykinin 3	0.62
BGN	Biglycan	0.61
ZNF658B	Zinc finger protein 658B	0.61
BUD31	BUD31	0.60
PPP1R3B	Protein phosphatase 1, regulatory subunit 3B	0.59
GPR141	G-protein-coupled receptor 141	0.57

**Table 2:** Significantly altered metabolites (*p*-values < 0.05) in HeLa cells treated with RhNPs and NIR irradiation.

Compound	Retention Time (min)	NIST Rmatch	R_M_^a^
Galactose	25.430	857	14.13
Hexadecanoate	27.885	826	3.86
Decanedionate	38.081	844	3.66
Pyrimidine	21.766	733	3.57
Propionate	14.872	835	3.26
Myo-inositol	28.438	814	2.93
Gluconate	26.562	778	2.66
Oleate	30.671	791	2.65
N-Pentadecanoate	26.324	823	1.92
Creatinine	19.198	735	1.58
Malate	18.056	865	1.54
11-trans octadecenoate	30.546	864	0.69
Tetradecanoate	24.696	865	0.58
Fructose	25.022	837	0.40
Serine	13.446	769	0.14
Nonanoate	15.425	778	0.04

^a^ Area RhNPs/Area control.

## Data Availability

Data is contained within the article and supplementary material.

## Supplementary Materials

The following are available online at https://www.mdpi.com/article/10.3390/pharmaceutics13101629/s1, Figure S1: TEM micrograph of RhNPs on aqueous suspension; Figure S2: Representative chromatograms obtained in TIC mode for metabolite extracts from control (**A**) and treated (**B**) samples; Table S1: Transitions and collision energies used for the quantification of ATP, ADP, NADH and NAD^+^ by LC-QqQ-MS working on MRM mode; Table S2: Whole transcriptome analysis of HeLa cells exposed to RhNPs and NIR; Table S3: Common metabolites identified in control and treated samples by GC-TOF-MS with a minimum NIST Rmatch value of 700; Table S4: Pearson’s correlation matrix data for the 48 identified metabolites; Table S5: PCA score values for controls and treated samples on the PC3.
