# Supplementary material for: Integration of Transcriptomics and Metabolomics to Reveal the Molecular Mechanisms Underlying Rhodium Nanoparticles-Based Photodynamic Cancer Therapy
Source: Pharmaceutics. 2021 Oct 6;13(10):1629. doi: 10.3390/pharmaceutics13101629 (PMC8539937; doi:10.3390/pharmaceutics13101629)
Supplement: Supplementary file 1 [file pharmaceutics-13-01629-s001.zip › Supplemental table S2.pdf]

Supplemental table S2. Whole transcriptomanalysis of HeLa cells exposed to RhNPs+NIR.

RhNPs vs. Control	Gene code	Gene name
1,61	BRD9	Transcript Identified by AceView, Entrez Gene ID(s) 65980
1,58	AP3B1	Memczak2013 ALT_ACCEPTOR, ALT_DONOR, coding, INTERNAL, intronic best transcript NM_003664
1,56	NLRP11	NLR family, pyrin domain containing 11
1,56	IL7R	interleukin 7 receptor
1,56	CCDC190	coiled-coil domain containing 190
1,52	KIDINS220	Transcript Identified by AceView, Entrez Gene ID(s) 57498; 100216337
1,52	TMLHE-AS1; LOC101927830; CLIC2; RP13-228J13.1	Homo sapiens TMLHE antisense RNA 1 (TMLHE-AS1), long non-coding RNA.; uncharacterized LOC101927830; Transcript Identified by AceView, Entrez Gene ID(s) 1193; novel transcript
1,51	DOCK2	Memczak2013 ANTISENSE, CDS, coding, INTERNAL best transcript NM_004946
1,51	SRF	Jeck2013 ANTISENSE, CDS, coding, INTERNAL, intronic, OVCODE, OVEXON best transcript NM_003131
1,50	USHBP1	Usher syndrome 1C binding protein 1
1,49	CPA4	carboxypeptidase A4
1,49	SLA	Src-like-adaptor
1,49	MFAP4	microfibrillar associated protein 4
1,48	FSIP1	fibrous sheath interacting protein 1
1,48	RPS6KA1	ribosomal protein S6 kinase, 90kDa, polypeptide 1
1,48	MTFR1	mitochondrial fission regulator 1
1,48	ARR3	arrestin 3, retinal (X-arrestin)
1,46	LVRN	laeverin
1,45	ZNF780A	zinc finger protein 780A
1,45	USP17L12	ubiquitin specific peptidase 17-like family member 12
1,45	UNC13C	unc-13 homolog C (C. elegans)
1,45	IGFL3	IGF like family member 3
1,44	TMC5	transmembrane channel like 5
1,44	SOCS1	suppressor of cytokine signaling 1
1,44	TTC31	Memczak2013 ALT_ACCEPTOR, ALT_DONOR, coding, INTERNAL, intronic best transcript NM_022492
1,44	PSMG4	proteasome (prosome, macropain) assembly chaperone 4
1,44	SPSB2	splA/ryanodine receptor domain and SOCS box containing 2
1,43	TOX2	Memczak2013 ALT_ACCEPTOR, ALT_DONOR, coding, INTERNAL, intronic best transcript NM_001098797

1,43	FRMD3	FERM domain containing 3
1,43	USP18	ubiquitin specific peptidase 18
1,43	SAG	S-antigen; retina and pineal gland (arrestin)
1,43	TAL1	T-cell acute lymphocytic leukemia 1
1,43	FAM234B	family with sequence similarity 234, member B
1,43	OR5K2	olfactory receptor, family 5, subfamily K, member 2
1,42	MAL2	mal, T-cell differentiation protein 2 (gene/pseudogene)
1,42	SNTG1	syntrophin, gamma 1
1,42	RUFY3	RUN and FYVE domain containing 3
1,42	EMCN	endomucin
1,42	HAND2	heart and neural crest derivatives expressed 2
1,42	AKAP6	A kinase (PRKA) anchor protein 6
1,41	ANKAR	ankyrin and armadillo repeat containing
1,41	EFCAB5	EF-hand calcium binding domain 5
1,41	ZNF829	zinc finger protein 829
1,41	FMO1	Transcript Identified by AceView, Entrez Gene ID(s) 2326
1,41	PITPNA	Memczak2013 ALT_ACCEPTOR, ALT_DONOR, coding, INTERNAL, intronic best transcript NM_006224
1,41	FARP1	Jeck2013 ALT_ACCEPTOR, ALT_DONOR, coding, INTERNAL, intronic best transcript NM_005766
1,41	OCLM	oculomedin
1,41	INTU	inturned planar cell polarity protein
1,41	PILRB	Jeck2013 ANTISENSE, INTERNAL, intronic, ncRNA, OVEXON best transcript NR_036569
1,41	LRIG1	
1,41	MCF2L2	MCF.2 cell line derived transforming sequence-like 2
1,40	NEK8	NIMA-related kinase 8
1,40	VCY; VCY1B	variable charge, Y-linked; variable charge, Y-linked 1B
1,40	TMEM105	transmembrane protein 105
1,40	MYO9A	Memczak2013 ANTISENSE, CDS, coding, INTERNAL best transcript NM_006901
1,40	OR51E2	olfactory receptor, family 51, subfamily E, member 2
1,40	ST8SIA1	ST8 alpha-N-acetyl-neuraminide alpha-2,8-sialyltransferase 1
1,40	SLITRK6	SLIT and NTRK-like family, member 6
1,40	SFTA3	surfactant associated 3
1,40	DEFB109P1B	defensin, beta 109, pseudogene 1B
1,39	SELL	Memczak2013 ALT_ACCEPTOR, ALT_DONOR, coding, INTERNAL, intronic best transcript NM_000655
1,39	SYCE2	synaptonemal complex central element protein 2
1,39	ZNF497; A1BG	zinc finger protein 497; alpha-1-B glycoprotein
1,39	LCNL1	lipocalin-like 1
1,39	AFF3	AF4/FMR2 family, member 3
1,39	TMEM167A	Transcript Identified by AceView, Entrez Gene ID(s) 153339

1,39	NADSYN1	NAD synthetase 1
1,39	SLAMF6	Memczak2013 ANTISENSE, coding, INTERNAL, intronic best transcript NM_052931
1,39	LONRF1	Transcript Identified by AceView, Entrez Gene ID(s) 91694
1,39	MAS1	MAS1 proto-oncogene, G protein-coupled receptor
1,39	OR1M1	olfactory receptor, family 1, subfamily M, member 1
1,38	MRLN	myoregulin
1,38	CUZD1	CUB and zona pellucida-like domains 1
1,38	DHRS2	dehydrogenase/reductase (SDR family) member 2
1,38	GZMH	granzyme H
1,38	NXPE2	neurexophilin and PC-esterase domain family, member 2
1,38	TSC1	Transcript Identified by AceView, Entrez Gene ID(s) 7248
1,38	DGKA	diacylglycerol kinase alpha
1,38	PAIP2; CTB-43P18.1	poly(A) binding protein interacting protein 2 [Source:EntrezGene;Acc:51247]; novel transcript, antisense to PAIP2
1,38	RSPH1	radial spoke head 1 homolog (Chlamydomonas)
1,38	JAK2	Memczak2013 ALT_ACCEPTOR, ALT_DONOR, coding, INTERNAL, intronic best transcript NM_004972
1,38	FAM53C	family with sequence similarity 53, member C
1,38	OR2B2	olfactory receptor, family 2, subfamily B, member 2
1,38	KRTAP5-9	keratin associated protein 5-9
1,38	CHRNE	cholinergic receptor, nicotinic epsilon
1,38	OR3A2	olfactory receptor, family 3, subfamily A, member 2
1,37	ACSS2	Memczak2013 ANTISENSE, CDS, coding, INTERNAL best transcript NM_001076552
1,37	CCR2	chemokine (C-C motif) receptor 2
1,37	LINC01496	long intergenic non-protein coding RNA 1496
1,37	IGDCC3	immunoglobulin superfamily, DCC subclass, member 3
1,37	GHDC	GH3 domain containing
1,37	OR2S2	olfactory receptor, family 2, subfamily S, member 2 (gene/pseudogene)
1,37	BIN3-IT1	BIN3 intronic transcript 1
1,37	HRASLS	HRAS-like suppressor
1,36	GRIN1	glutamate receptor, ionotropic, N-methyl D-aspartate 1
1,36	SEC14L4	SEC14-like lipid binding 4
1,36	GBP4	guanylate binding protein 4
1,36	GBP5	guanylate binding protein 5
1,36	PTPRO	protein tyrosine phosphatase, receptor type, O
1,36	ERBB2	Salzman2013 ANNOTATED, coding, OVEXON, UTR5 best transcript NM_001005862
1,36	ANK3	ankyrin 3, node of Ranvier (ankyrin G)
1,36	DYNC1I1	dynein, cytoplasmic 1, intermediate chain 1

1,35	GTF3C5	Zhang2013 ALT_ACCEPTOR, ALT_DONOR, coding, INTERNAL, intronic best transcript NM_001122823
1,35	CYP2B6	cytochrome P450, family 2, subfamily B, polypeptide 6
1,35	EPS15L1	epidermal growth factor receptor pathway substrate 15-like 1
1,35	AKR1C4	aldo-keto reductase family 1, member C4
1,35	ASIC2	acid sensing ion channel 2
1,35	LAMB4	laminin, beta 4
1,35	BIRC6	Transcript Identified by AceView, Entrez Gene ID(s) 57448
1,35	TXNDC2	thioredoxin domain containing 2 (spermatozoa)
1,35	TLN2	talin 2
1,35	PLGLB1; PLGLB2	plasminogen-like B1; plasminogen-like B2
1,35	CTNNA2	catenin (cadherin-associated protein), alpha 2
1,35	PDGFRA	platelet-derived growth factor receptor, alpha polypeptide
1,35	MRVI1	murine retrovirus integration site 1 homolog
1,35	NTSR2	neurotensin receptor 2
1,35	HLTF	Transcript Identified by AceView, Entrez Gene ID(s) 6596
1,35	TAS2R4	taste receptor, type 2, member 4
1,34	MCOLN2	mucolipin 2
1,34	BACH2	Transcript Identified by AceView, Entrez Gene ID(s) 60468
1,34	XAF1	XIAP associated factor 1
1,34	SPRN	shadow of prion protein homolog (zebrafish)
1,34	FCGR2C	Fc fragment of IgG, low affinity IIc, receptor for (CD32) (gene/pseudogene)
1,34	OIT3	oncoprotein induced transcript 3
1,34	RPS15	Memczak2013 ANTISENSE, coding, INTERNAL, intronic best transcript NM_001018
1,34	CDCA5	Memczak2013 ANTISENSE, CDS, coding, INTERNAL, UTR3 best transcript NM_080668
1,34	BTG4	B-cell translocation gene 4
1,34	CA3	carbonic anhydrase III
1,34	VIL1	villin 1
1,34	GIPC2	GIPC PDZ domain containing family, member 2
1,34	SLC26A7	solute carrier family 26 (anion exchanger), member 7
1,34	FA2H	fatty acid 2-hydroxylase
1,34	SULT1C2	sulfotransferase family 1C member 2
1,34	PIP5K1B	phosphatidylinositol-4-phosphate 5-kinase, type I, beta
1,34	SPATS2L	Transcript Identified by AceView, Entrez Gene ID(s) 26010
1,34	DNAH10	dynein, axonemal, heavy chain 10
1,33	FAM153A	Transcript Identified by AceView, Entrez Gene ID(s) 285596
1,33	SP140L	SP140 nuclear body protein-like

1,33	CHAC1	ChaC glutathione-specific gamma-glutamylcyclotransferase 1
1,33	GARNL3	GTPase activating Rap/RanGAP domain-like 3
1,33	ITPR3	Zhang2013 ALT_ACCEPTOR, ALT_DONOR, coding, INTERNAL, intronic best transcript NM_002224
1,33	MBD3L5	methyl-CpG binding domain protein 3-like 5
1,33	ARMC4	armadillo repeat containing 4
1,33	HMBS	hydroxymethylbilane synthase
1,33	CCDC64	coiled-coil domain containing 64
1,33	HDGFL1	hepatoma derived growth factor-like 1
1,33	TSPAN16	tetraspanin 16
1,33	EPO	erythropoietin
1,33	SORL1	Memczak2013 ANTISENSE, CDS, coding, INTERNAL best transcript NM_003105
1,33	DCDC2C	doublecortin domain containing 2C
1,33	GPCPD1	Transcript Identified by AceView, Entrez Gene ID(s) 56261
1,33	ADGRA1	adhesion G protein-coupled receptor A1
1,33	GKN1	gastrokine 1
1,33	OTOL1	otolin 1
1,33	IL1RL1	interleukin 1 receptor-like 1
1,33	XYLT1	xylosyltransferase I
1,33	APOBR	apolipoprotein B receptor
1,33	TBC1D26	TBC1 domain family, member 26
1,33	AZGP1	alpha-2-glycoprotein 1, zinc-binding
1,33	ZNF750	zinc finger protein 750
1,33	CLDN34	claudin 34
1,33	CCZ1B	CCZ1 homolog B, vacuolar protein trafficking and biogenesis associated
1,33	PADI6	peptidyl arginine deiminase, type VI
1,33	RGS4	regulator of G-protein signaling 4
1,33	ROS1	ROS proto-oncogene 1 , receptor tyrosine kinase
1,33	C7orf77	chromosome 7 open reading frame 77
1,33	SLCO2B1	solute carrier organic anion transporter family, member 2B1
1,32	DENND6B	DENN/MADD domain containing 6B
1,32	POLR3G	polymerase (RNA) III (DNA directed) polypeptide G (32kD)
1,32	FAM186B	family with sequence similarity 186, member B
1,32	SCNN1D	sodium channel, non voltage gated 1 delta subunit
1,32	PRNT	prion protein (testis specific)
1,32	ANKRD55	ankyrin repeat domain 55
1,32	FGF16	fibroblast growth factor 16
1,32	FAM169A	family with sequence similarity 169, member A
1,32	ASXL3	additional sex combs like transcriptional regulator 3
1,32	PHGDH	Memczak2013 ANTISENSE, CDS, coding, INTERNAL best transcript NM_006623
1,32	RALYL	RALY RNA binding protein-like

1,32	SLC10A1	Memczak2013 ANTISENSE, CDS, coding, INTERNAL, intronic best transcript NM_003049
1,32	MED12	Memczak2013 ANTISENSE, CDS, coding, INTERNAL best transcript NM_005120
1,32	FILIP1L	filamin A interacting protein 1-like
1,32	LOXHD1	lipoxygenase homology domains 1
1,32	COLCA2	colorectal cancer associated 2
1,32	LINGO4	leucine rich repeat and Ig domain containing 4
1,32	PRY2; PRY	PTPN13-like, Y-linked 2; PTPN13-like, Y-linked
1,32	CYB561D1	cytochrome b561 family, member D1
1,32	CAPN8	calpain 8
1,32	ARPP21	Transcript Identified by AceView, Entrez Gene ID(s) 10777
1,32	PRAMEF13	PRAME family member 13
1,32	CDH18	cadherin 18, type 2
1,32	CNGA4	cyclic nucleotide gated channel alpha 4
1,32	SYN1	synapsin I
1,32	CDH12	cadherin 12, type 2 (N-cadherin 2)
1,32	CHN2	chimerin 2
1,32	SGOL2	shugoshin-like 2 (S. pombe)
1,32	CYP3A5	cytochrome P450, family 3, subfamily A, polypeptide 5
1,31	DNASE1L3	deoxyribonuclease I-like 3
1,31	STKLD1	serine/threonine kinase-like domain containing 1 [Source:HGNC Symbol;Acc:HGNC:28669]
1,31	FAM110D	family with sequence similarity 110, member D
1,31	LCE1D	late cornified envelope 1D
1,31	ART5	ADP-ribosyltransferase 5
1,31	SOD3	superoxide dismutase 3, extracellular
1,31	GALNT14	Memczak2013 ALT_ACCEPTOR, ALT_DONOR, INTERNAL, intronic, ncRNA best transcript NR_045602
1,31	CD300LF	CD300 molecule-like family member f
1,31	TPSB2; TPSAB1	tryptase beta 2 (gene/pseudogene); tryptase alpha/beta 1
1,31	SVEP1	sushi, von Willebrand factor type A, EGF and pentraxin domain containing 1
1,31	RUFY1	Transcript Identified by AceView, Entrez Gene ID(s) 80230
1,31	COL5A3	collagen, type V, alpha 3
1,31	KRTAP20-1	keratin associated protein 20-1
1,31	MYRF	myelin regulatory factor
1,31	NPY1R	neuropeptide Y receptor Y1
1,31	CDC14B	cell division cycle 14B
1,31	OR4C15	olfactory receptor, family 4, subfamily C, member 15
1,31	RBFOX1	Memczak2013 ALT_ACCEPTOR, ALT_DONOR, coding, INTERNAL, intronic best transcript NM_001142333
1,31	ERVMER34-1	endogenous retrovirus group MER34, member 1
1,31	CA1	carbonic anhydrase I

1,31	CCL26	chemokine (C-C motif) ligand 26
1,31	LINC00452	long intergenic non-protein coding RNA 452
1,31	TF	transferrin
1,31	BCL6B	B-cell CLL/lymphoma 6, member B
1,31	OTOR	otoraplin
1,31	ZNF385A	Memczak2013 ANTISENSE, CDS, coding, INTERNAL best transcript NM_001130967
1,31	PTPN6	protein tyrosine phosphatase, non-receptor type 6
1,31	MMP1	matrix metallopeptidase 1
1,31	BBOX1	butyrobetaine (gamma), 2-oxoglutarate dioxygenase (gamma-butyrobetaine hydroxylase) 1
1,31	NOVA1	neuro-oncological ventral antigen 1
1,31	RPS25	ribosomal protein S25
1,31	FAM124A	family with sequence similarity 124 member A
1,31	FAM131C	family with sequence similarity 131, member C
1,31	CHST11	Transcript Identified by AceView, Entrez Gene ID(s) 50515
1,30	RFPL1	ret finger protein-like 1
1,30	GCGR	glucagon receptor
1,30	SPDYA	speedy/RINGO cell cycle regulator family member A
1,30	PKHD1L1	polycystic kidney and hepatic disease 1 (autosomal recessive)-like 1
1,30	GPR45	G protein-coupled receptor 45
1,30	GRM3	glutamate receptor, metabotropic 3
1,30	ABCD3	Transcript Identified by AceView, Entrez Gene ID(s) 5825
1,30	CD3E	CD3e molecule, epsilon (CD3-TCR complex)
1,30	MAGEB3	MAGE family member B3
1,30	GMFG	glia maturation factor, gamma
1,30	KDM4C	Transcript Identified by AceView, Entrez Gene ID(s) 23081
1,30	NALCN	sodium leak channel, non selective
1,30	NXF1	nuclear RNA export factor 1
1,30	EGF	epidermal growth factor
1,30	TRMT2A	Memczak2013 ANTISENSE, CDS, coding, INTERNAL, UTR3 best transcript NM_022727
1,30	ENTHD1	ENTH domain containing 1
1,30	RIMBP3C; RIMBP3B	RIMS binding protein 3C; RIMS binding protein 3B
1,30	FRMD4A	FERM domain containing 4A
1,30	MUC12	mucin 12, cell surface associated
1,30	ZFPM1	zinc finger protein, FOG family member 1
1,30	BMP4	bone morphogenetic protein 4
1,30	NUTM2F	NUT family member 2F
1,30	GRM7	glutamate receptor, metabotropic 7
1,30	OR5D14	olfactory receptor, family 5, subfamily D, member 14
1,30	LAP3	Transcript Identified by AceView, Entrez Gene ID(s) 51056
1,29	IL17A	interleukin 17A

1,29	MYO9B	Memczak2013 ANTISENSE, CDS, coding, INTERNAL best transcript NM_004145
1,29	DNAJC28	DnaJ (Hsp40) homolog, subfamily C, member 28
1,29	PADI1	peptidyl arginine deiminase, type I
1,29	C1QTNF3	C1q and tumor necrosis factor related protein 3
1,29	LRRN4CL	LRRN4 C-terminal like
1,29	TAF1C	TATA box binding protein (TBP)-associated factor, RNA polymerase I, C, 110kDa
1,29	SERHL2	serine hydrolase-like 2
1,29	TMEM140	transmembrane protein 140
1,29	CYP26C1	cytochrome P450, family 26, subfamily C, polypeptide 1
1,29	FNDC7	fibronectin type III domain containing 7
1,29	INHBA	inhibin beta A
1,29	PTGES	prostaglandin E synthase
1,29	TMEM236	transmembrane protein 236
1,29	IQSEC3	IQ motif and Sec7 domain 3
1,29	CCDC85A	coiled-coil domain containing 85A
1,29	PCYT1B	phosphate cytidylyltransferase 1, choline, beta
1,29	CEP85L	centrosomal protein 85kDa-like
1,29	LRCH4	leucine-rich repeats and calponin homology (CH) domain containing 4
1,29	FMN1	formin 1
1,29	OLFML3	olfactomedin like 3
1,29	C3orf33	chromosome 3 open reading frame 33
1,29	ZDHHC3	zinc finger, DHHC-type containing 3
1,29	MSH6	mutS homolog 6
1,29	DEFB136	defensin, beta 136
1,29	RAB25	RAB25, member RAS oncogene family
1,29	USP45	ubiquitin specific peptidase 45
1,29	CABLES2	Cdk5 and Abl enzyme substrate 2
1,29	HOXA1	homeobox A1
1,29	TRPS1	Transcript Identified by AceView, Entrez Gene ID(s) 7227
1,29	KCNMA1; swamu	Jeck2013 ALT_ACCEPTOR, ALT_DONOR, coding, INTERNAL, intronic best transcript NM_001014797; Transcript Identified by AceView
1,28	SPINT4	serine peptidase inhibitor, Kunitz type 4
1,28	HLA-DQB1	major histocompatibility complex, class II, DQ beta 1
1,28	NID2	nidogen 2 (osteonidogen)
1,28	AVPR2	arginine vasopressin receptor 2
1,28	GALC	galactosylceramidase
1,28	UGT3A2	UDP glycosyltransferase 3 family, polypeptide A2
1,28	RIBC1	RIB43A domain with coiled-coils 1
1,28	FAM196B	family with sequence similarity 196, member B
1,28	VSNL1	visinin like 1
1,28	PLGLB1	Transcript Identified by AceView, Entrez Gene ID(s) 285074; 5343
1,28	HPGDS	hematopoietic prostaglandin D synthase
1,28	SGK2	serum/glucocorticoid regulated kinase 2

1,28	ACSM5	acyl-CoA synthetase medium-chain family member 5
1,28	CDH9	cadherin 9, type 2 (T1-cadherin)
1,28	MORN4	MORN repeat containing 4
1,28	LRRC37A5P	leucine rich repeat containing 37, member A5, pseudogene
1,28	RNF39	ring finger protein 39
1,28	CBL	Memczak2013 ANTISENSE, coding, INTERNAL, UTR3 best transcript NM_005188
1,28	SPATA31A5; SPATA31A7	SPATA31 subfamily A, member 5; SPATA31 subfamily A, member 7
1,28	NRXN3	neurexin 3
1,28	S100A7A	S100 calcium binding protein A7A
1,28	LOC100506142; RP11-417F21.1; RHOQ	uncharacterized LOC100506142; Memczak2013 ANTISENSE, coding, INTERNAL, intronic best transcript NM_012249; novel transcript
1,28	VPS16	vacuolar protein sorting 16 homolog (S. cerevisiae)
1,28	DKK1	dickkopf WNT signaling pathway inhibitor 1
1,28	C7orf69	chromosome 7 open reading frame 69
1,28	PKMYT1	Transcript Identified by AceView, Entrez Gene ID(s) 9088
1,28	CLASP2	Transcript Identified by AceView, Entrez Gene ID(s) 23122
1,28	SEMA5A	sema domain, seven thrombospondin repeats (type 1 and type 1-like), transmembrane domain (TM) and short cytoplasmic domain, (semaphorin) 5A
1,28	SLC25A26	solute carrier family 25 (S-adenosylmethionine carrier), member 26
1,28	SPICE1	spindle and centriole associated protein 1
1,28	IGSF9	immunoglobulin superfamily, member 9
1,28	CDH22	Transcript Identified by AceView, Entrez Gene ID(s) 64405
1,28	LINC00311	long intergenic non-protein coding RNA 311
1,27	VCY1B	variable charge, Y-linked 1B
1,27	FAM162B	family with sequence similarity 162, member B
1,27	ATP13A5-AS1	ATP13A5 antisense RNA 1
1,27	SPDEF	SAM pointed domain containing ETS transcription factor
1,27	ALAD	aminolevulinate dehydratase
1,27	LYRM5	Memczak2013 ANTISENSE, coding, INTERNAL, intronic best transcript NM_001001660
1,27	DUX5	double homeobox 5
1,27	POU4F2	POU class 4 homeobox 2
1,27	NFASC	neurofascin
1,27	XPA	xeroderma pigmentosum, complementation group A
1,27	ALKBH6	alkB homolog 6
1,27	MSL3	male-specific lethal 3 homolog (Drosophila)
1,27	CUL2; RP11-297A16.2	Transcript Identified by AceView, Entrez Gene ID(s) 8453; novel transcript
1,27	ARMC3	armadillo repeat containing 3
1,27	PRSS45	protease, serine 45

1,27	CYP46A1	cytochrome P450, family 46, subfamily A, polypeptide 1
1,27	B3GAT1	beta-1,3-glucuronyltransferase 1
1,27	FETUB	fetuin B
1,27	GSX1	GS homeobox 1
1,27	INPP5D	inositol polyphosphate-5-phosphatase D
1,27	CDO1	cysteine dioxygenase type 1
1,27	THSD4	Transcript Identified by AceView, Entrez Gene ID(s) 79875
1,27	GPSM1	G-protein signaling modulator 1
1,27	GATM	glycine amidinotransferase (L-arginine:glycine amidinotransferase)
1,27	OR2A4	olfactory receptor, family 2, subfamily A, member 4
1,27	C1orf189	chromosome 1 open reading frame 189
1,27	ASPDH	aspartate dehydrogenase domain containing
1,27	OVCH2	ovochymase 2 (gene/pseudogene)
1,27	UBA7	Memczak2013 ANTISENSE, coding, INTERNAL, intronic best transcript NM_003335
1,27	SNX32	sorting nexin 32
1,27	LINC00550	long intergenic non-protein coding RNA 550
1,27	CARHSP1	Jeck2013 ANTISENSE, CDS, coding, INTERNAL, OVCODE, OVEXON best transcript NM_001042476
1,27	CEACAM1	carcinoembryonic antigen-related cell adhesion molecule 1 (biliary glycoprotein)
1,27	PCDHB5	protocadherin beta 5
1,27	BCL7A	B-cell CLL/lymphoma 7A
1,27	IL6R	interleukin 6 receptor
1,27	TJP2	tight junction protein 2
1,27	KAT8	K(lysine) acetyltransferase 8
1,27	NXF2B; TCP11X2	nuclear RNA export factor 2B; t-complex 11 family, X-linked 2
1,27	SIX3	SIX homeobox 3
1,27	CXCL1	chemokine (C-X-C motif) ligand 1 (melanoma growth stimulating activity, alpha)
1,27	BRSK1	BR serine/threonine kinase 1
1,27	OR2G3	olfactory receptor, family 2, subfamily G, member 3
1,27	IQCD	IQ motif containing D
1,27	EVA1A	eva-1 homolog A (C. elegans)
1,27	ARHGAP10	Transcript Identified by AceView, Entrez Gene ID(s) 79658
1,26	PRB4	proline-rich protein BstNI subfamily 4
1,26	TGFA	transforming growth factor alpha
1,26	BCL2L15	BCL2-like 15
1,26	AGBL3	ATP/GTP binding protein-like 3
1,26	ACY3	aminoacylase 3
1,26	CCBE1	collagen and calcium binding EGF domains 1
1,26	NRIP2	nuclear receptor interacting protein 2
1,26	PDZD9	PDZ domain containing 9
1,26	PAK6; BUB1B	p21 protein (Cdc42/Rac)-activated kinase 6; BUB1 mitotic checkpoint serine/threonine kinase B

1,26	INMT	indolethylamine N-methyltransferase
1,26	NCF1	neutrophil cytosolic factor 1
1,26	ARHGEF35	Rho guanine nucleotide exchange factor 35
1,26	LANCL3	LanC lantibiotic synthetase component C-like 3 (bacterial)
1,26	KCNJ15	potassium channel, inwardly rectifying subfamily J, member 15
1,26	HIST1H4C	Jeck2013 ANTISENSE, CDS, coding, INTERNAL, OVCODE, OVEXON, UTR3 best transcript NM_003542
1,26	UROC1	urocanate hydratase 1
1,26	NXPH1	neurexophilin 1
1,26	CLIP3	CAP-GLY domain containing linker protein 3
1,26	PYDC1	PYD (pyrin domain) containing 1
1,26	ARHGAP20	Rho GTPase activating protein 20
1,26	TAS2R39	taste receptor, type 2, member 39
1,26	MAF	v-maf avian musculoaponeurotic fibrosarcoma oncogene homolog
1,26	FAM72D	Transcript Identified by AceView, Entrez Gene ID(s) 728833
1,26	PRYP3	PTPN13-like, Y-linked pseudogene 3
1,26	C15orf56	chromosome 15 open reading frame 56
1,26	ESRRG	estrogen-related receptor gamma
1,26	DDX60L	DEAD (Asp-Glu-Ala-Asp) box polypeptide 60-like
1,26	TUBA1C	tubulin, alpha 1c
1,26	CXorf66	chromosome X open reading frame 66
1,26	MRGPRE	MAS-related GPR, member E
1,26	XKR5	X-linked Kx blood group related 5
1,26	C7orf62	chromosome 7 open reading frame 62
1,26	ANKRD61	ankyrin repeat domain 61
1,26	VPS8	Transcript Identified by AceView, Entrez Gene ID(s) 23355
1,26	SCG3	secretogranin III
1,26	SHANK2-AS3	SHANK2 antisense RNA 3
1,26	CCDC160	coiled-coil domain containing 160
1,26	TAS1R3	taste receptor, type 1, member 3
1,26	PRSS54	protease, serine, 54
1,26	FBLL1	fibrillarin-like 1
1,26	BLNK	B-cell linker
1,26	F2	coagulation factor II (thrombin)
1,26	L3MBTL4	l(3)mbt-like 4 (Drosophila)
1,26	INMT; FAM188B; INMT-FAM188B	indolethylamine N-methyltransferase; family with sequence similarity 188, member B; INMT-FAM188B readthrough (NMD candidate)
1,26	BSPRY	B-box and SPRY domain containing
1,26	GAGE12I; GAGE12F	G antigen 12I; G antigen 12F
1,26	FOXO1	Memczak2013 ALT_ACCEPTOR, ALT_DONOR, coding, INTERNAL, intronic best transcript NM_002015

1,26	NT5M	5,3-nucleotidase, mitochondrial
1,26	OR6N1	olfactory receptor, family 6, subfamily N, member 1
1,26	PSMC6	Memczak2013 ALT_ACCEPTOR, ALT_DONOR, coding, INTERNAL, intronic best transcript NM_002806
1,26	PASK	PAS domain containing serine/threonine kinase
1,26	BCAS3	breast carcinoma amplified sequence 3
1,26	ACTR5	ARP5 actin-related protein 5 homolog (yeast)
1,26	SECTM1	secreted and transmembrane 1
1,26	SPEF1	sperm flagellar 1
1,26	ALG13	Transcript Identified by AceView, Entrez Gene ID(s) 79868
1,26	CCDC85C	coiled-coil domain containing 85C
1,26	ABCA4	ATP binding cassette subfamily A member 4
1,25	OR2D3	olfactory receptor, family 2, subfamily D, member 3
1,25	FLJ22763; RP11-59E19.1	uncharacterized LOC401081; uncharacterized LOC401081 [Source:EntrezGene;Acc:401081]; novel transcript
1,25	TLR5	toll-like receptor 5
1,25	RGS18	regulator of G-protein signaling 18
1,25	SPRY1	sprouty RTK signaling antagonist 1
1,25	ATAT1	alpha tubulin acetyltransferase 1
1,25	OR2A25	olfactory receptor, family 2, subfamily A, member 25
1,25	GPR15	G protein-coupled receptor 15
1,25	VPS11	VPS11, CORVET/HOPS core subunit [Source:HGNC Symbol;Acc:HGNC:14583]
1,25	VWCE	von Willebrand factor C and EGF domains
1,25	LYPD6B	LY6/PLAUR domain containing 6B
1,25	GPBAR1	G protein-coupled bile acid receptor 1
1,25	LINC00649	long intergenic non-protein coding RNA 649
1,25	AIM2	absent in melanoma 2
1,25	OR2AG2	olfactory receptor, family 2, subfamily AG, member 2
1,25	EBI3	Epstein-Barr virus induced 3
1,25	STIM2	stromal interaction molecule 2
1,25	ACTN2	actinin, alpha 2
1,25	ARL4A	ADP-ribosylation factor like GTPase 4A
1,25	LAD1	ladinin 1
1,25	LTC4S	leukotriene C4 synthase
1,25	MPZ	myelin protein zero
1,25	ARL4D	ADP-ribosylation factor like GTPase 4D
1,25	NPM2	nucleophosmin/nucleoplasmin 2
1,25	A1CF	APOBEC1 complementation factor
1,25	CCER1	coiled-coil glutamate rich protein 1
1,25	ZNF335	zinc finger protein 335
1,25	TMEM177	transmembrane protein 177
1,25	PRRG4	proline rich Gla (G-carboxyglutamic acid) 4 (transmembrane)
1,25	COLEC11	collectin subfamily member 11
1,25	ANKRD42	ankyrin repeat domain 42

1,25	ELF5	E74-like factor 5 (ets domain transcription factor)
1,25	PDE11A	phosphodiesterase 11A
1,25	WIPF2	WAS/WASL interacting protein family, member 2
1,25	ELMO1	engulfment and cell motility 1
1,25	CP	ceruloplasmin (ferroxidase)
1,25	SCN8A	sodium channel, voltage gated, type VIII alpha subunit
1,25	ADGRB1	adhesion G protein-coupled receptor B1
1,25	ILDR1	immunoglobulin-like domain containing receptor 1
1,25	MRGPRX4	MAS-related GPR, member X4
1,25	IRS1	insulin receptor substrate 1
1,25	GALNTL5	polypeptide N-acetylgalactosaminyltransferase-like 5
1,25	SLC9B2	solute carrier family 9, subfamily B (NHA2, cation proton antiporter 2), member 2
1,25	HCRP1	Transcript Identified by AceView, Entrez Gene ID(s) 387535
1,25	SPTSSB	serine palmitoyltransferase, small subunit B
1,25	RBMS1	Memczak2013 ALT_ACCEPTOR, ALT_DONOR, coding, INTERNAL, intronic best transcript NM_016836
1,25	C2orf61	chromosome 2 open reading frame 61
1,25	TLR10	toll-like receptor 10
1,25	TNS1	tensin 1
1,24	RASSF9	Ras association (RalGDS/AF-6) domain family (N-terminal) member 9
1,24	ATG16L1	autophagy related 16-like 1
1,24	SYTL4	synaptotagmin-like 4
1,24	ATXN1	Memczak2013 ALT_ACCEPTOR, ALT_DONOR, coding, INTERNAL, intronic best transcript NM_000332
1,24	CFAP99	cilia and flagella associated protein 99
1,24	CA8	carbonic anhydrase VIII
1,24	ATP11AUN	ATP11A upstream neighbor
1,24	NHSL2	NHS-like 2
1,24	BEX1	brain expressed X-linked 1
1,24	GALNT18	polypeptide N-acetylgalactosaminyltransferase 18
1,24	SLC1A2	solute carrier family 1 (glial high affinity glutamate transporter), member 2
1,24	POGZ	Transcript Identified by AceView, Entrez Gene ID(s) 23126
1,24	DACH1	dachshund family transcription factor 1
1,24	MYLK4	myosin light chain kinase family member 4
1,24	LILRA4	leukocyte immunoglobulin-like receptor, subfamily A (with TM domain), member 4
1,24	KCNIP4	Kv channel interacting protein 4
1,24	PIGL	phosphatidylinositol glycan anchor biosynthesis class L
1,24	KCNE1	potassium channel, voltage gated subfamily E regulatory beta subunit 1
1,24	NELL1	neural EGFL like 1
1,24	DSG3	desmoglein 3

1,24	TMEM143	transmembrane protein 143
1,24	CDNF	cerebral dopamine neurotrophic factor
1,24	CDY2B; CDY2A	chromodomain protein, Y-linked, 2B; chromodomain protein, Y-linked, 2A
1,24	FAM65A	Memczak2013 ALT_ACCEPTOR, ALT_DONOR, coding, INTERNAL, intronic best transcript NM_001193522
1,24	PPP1R12C	Memczak2013 ANTISENSE, CDS, coding, INTERNAL best transcript NM_017607
1,24	CATSPER2	cation channel, sperm associated 2
1,24	CACNA1A	calcium channel, voltage-dependent, P/Q type, alpha 1A subunit
1,24	IRS2	Transcript Identified by AceView, Entrez Gene ID(s) 8660
1,24	TRPM6	transient receptor potential cation channel, subfamily M, member 6
1,24	LMNB2	Zhang2013 ALT_ACCEPTOR, ALT_DONOR, coding, INTERNAL, intronic best transcript NM_032737
1,24	GPSM3	G-protein signaling modulator 3
1,24	PPP3CC	protein phosphatase 3, catalytic subunit, gamma isozyme
1,24	PIK3C2B	Transcript Identified by AceView, Entrez Gene ID(s) 5287
1,24	FAT3	FAT atypical cadherin 3
1,24	SOX1	SRY box 1
1,24	PLK3	polo-like kinase 3
1,24	MMP13	matrix metallopeptidase 13
1,24	PLAC8	placenta specific 8
1,24	FCGR2B	Fc fragment of IgG, low affinity IIb, receptor (CD32)
1,24	GIMAP1-GIMAP5; GIMAP5; GIMAP1	GIMAP1-GIMAP5 readthrough; GTPase, IMAP family member 5; GTPase, IMAP family member 1
1,24	JAK2	Janus kinase 2
1,24	BHLHA9	basic helix-loop-helix family, member a9
1,24	TTLL9	tubulin tyrosine ligase-like family member 9
1,24	LINC00173	long intergenic non-protein coding RNA 173
1,24	ARMC2	Memczak2013 ANTISENSE, coding, INTERNAL, intronic best transcript NM_032131
1,24	ERP27	endoplasmic reticulum protein 27
1,24	TXNDC8	thioredoxin domain containing 8 (spermatozoa)
1,24	RTL1	retrotransposon-like 1
1,24	ARID1B	Memczak2013 ANTISENSE, CDS, coding, INTERNAL best transcript NM_017519
1,24	LOC105377348; RP11-10L12.4; UBE2D3; pugo	uncharacterized LOC105377348; Salzman2013 ANTISENSE, coding, INTERNAL, intronic, OVERLAPTX best transcript NM_181890; novel transcript, antisense UBE2D3; Transcript Identified by AceView
1,24	RBBP8NL	RBBP8 N-terminal like

1,24	RALY	Jeck2013 ALT_DONOR, coding, INTERNAL, intronic best transcript NM_016732
1,24	ZBTB45	Memczak2013 ALT_ACCEPTOR, ALT_DONOR, coding, INTERNAL, intronic best transcript NM_032792
1,24	PSG1	pregnancy specific beta-1-glycoprotein 1
1,24	RPS6KA1	ribosomal protein S6 kinase, 90kDa, polypeptide 1
1,24	RHBDD1	rhomboid domain containing 1
1,24	TTC9B	tetratricopeptide repeat domain 9B
1,24	SRP72	Memczak2013 ANTISENSE, coding, INTERNAL, intronic best transcript NM_006947
1,24	HKR1	HKR1, GLI-Kruppel zinc finger family member
1,24	TRIM59	tripartite motif containing 59
1,24	GPRASP2	G protein-coupled receptor associated sorting protein 2
1,24	SOX14	SRY box 14
1,24	SESN3	Transcript Identified by AceView, Entrez Gene ID(s) 143686
1,24	ACE	angiotensin I converting enzyme
1,24	HKR1	HKR1, GLI-Kruppel zinc finger family member
1,23	USP17L7	ubiquitin specific peptidase 17-like family member 7
1,23	XAGE5	X antigen family, member 5
1,23	SRM	spermidine synthase
1,23	WDR31	WD repeat domain 31
1,23	FMN1	Transcript Identified by AceView, Entrez Gene ID(s) 342184
1,23	C1QL2	complement component 1, q subcomponent-like 2
1,23	P4HA2	prolyl 4-hydroxylase, alpha polypeptide II
1,23	HERC6	Memczak2013 ANTISENSE, coding, INTERNAL, intronic best transcript NM_001165136
1,23	OPRK1	opioid receptor, kappa 1
1,23	HCG22	HLA complex group 22
1,23	CLEC1A	C-type lectin domain family 1, member A
1,23	RFPL3S	RFPL3 antisense
1,23	SIRPG	signal-regulatory protein gamma
1,23	MT4	metallothionein 4
1,23	ZNF780A	zinc finger protein 780A
1,23	GOLGA8N	golgin A8 family, member N
1,23	SMCO3	single-pass membrane protein with coiled-coil domains 3
1,23	MYOC	myocilin, trabecular meshwork inducible glucocorticoid response
1,23	KRTAP27-1	keratin associated protein 27-1
1,23	KBTBD8	kelch repeat and BTB (POZ) domain containing 8
1,23	LMLN	leishmanolysin-like (metallopeptidase M8 family)
1,23	POU5F1B	POU class 5 homeobox 1B
1,23	LEKR1	leucine, glutamate and lysine rich 1
1,23	BPGM	Transcript Identified by AceView, Entrez Gene ID(s) 669
1,23	CFHR2	complement factor H-related 2

1,23	SELP	selectin P (granule membrane protein 140kDa, antigen CD62)
1,23	TMPRSS11E	transmembrane protease, serine 11E
1,23	IAPP	islet amyloid polypeptide
1,23	SHOX	short stature homeobox
1,23	OR10S1	olfactory receptor, family 10, subfamily S, member 1
1,23	ABCA6	ATP binding cassette subfamily A member 6
1,23	ZNF506	zinc finger protein 506
1,23	TNFSF11	tumor necrosis factor (ligand) superfamily, member 11
1,23	C6orf163	chromosome 6 open reading frame 163
1,23	OPN4	opsin 4
1,23	FIGF	c-fos induced growth factor (vascular endothelial growth factor D)
1,23	KANK3	KN motif and ankyrin repeat domains 3
1,23	KRAS	Memczak2013 ANTISENSE, coding, INTERNAL, intronic best transcript NM_033360
1,23	BAHCC1	BAH domain and coiled-coil containing 1
1,23	PAPL	iron/zinc purple acid phosphatase-like protein; Iron/zinc purple acid phosphatase-like protein [Source:UniProtKB/Swiss-Prot;Acc:Q6ZNF0]; Transcript Identified by AceView, Entrez Gene ID(s) 390928
1,23	TTC5	tetratricopeptide repeat domain 5
1,23	SPATA12	spermatogenesis associated 12
1,23	ESRP2; MIR6773	epithelial splicing regulatory protein 2; microRNA 6773
1,23	CRISP3	cysteine-rich secretory protein 3
1,23	NUTM1	NUT midline carcinoma, family member 1
1,23	SLC9A2	solute carrier family 9, subfamily A (NHE2, cation proton antiporter 2), member 2
1,23	OR12D1	olfactory receptor, family 12, subfamily D, member 1 (gene/pseudogene) [Source:HGNC Symbol;Acc:HGNC:8177]
1,23	TMBIM1; MIR6513	transmembrane BAX inhibitor motif containing 1; microRNA 6513
1,23	MYBPC1	myosin binding protein C, slow type
1,23	GPER1	G protein-coupled estrogen receptor 1
1,23	ZNF225	zinc finger protein 225
1,23	FOXG1	forkhead box G1
1,23	RAB4B; MIA-RAB4B; RAB4B-EGLN2	RAB4B, member RAS oncogene family; MIA-RAB4B readthrough (NMD candidate); RAB4B-EGLN2 readthrough (NMD candidate)
1,23	TEX36	testis expressed 36
1,23	CST8	cystatin 8 (cystatin-related epididymal specific)
1,23	KIAA1033	Memczak2013 ALT_ACCEPTOR, ALT_DONOR, coding, INTERNAL, intronic best transcript NM_015275
1,23	ZNF780A	zinc finger protein 780A
1,23	ALS2CR11	amyotrophic lateral sclerosis 2 chromosome region candidate 11

1,23	ZNF215	zinc finger protein 215
1,23	GK	glycerol kinase
1,23	ZNF71	zinc finger protein 71
1,23	ZPLD1	zona pellucida-like domain containing 1
1,23	GPR160	G protein-coupled receptor 160
1,23	USP24	Transcript Identified by AceView, Entrez Gene ID(s) 23358
1,23	LOC79999; LOC388436; RP11-744A16.4	uncharacterized LOC79999; Homo sapiens uncharacterized protein ENSP00000382042 (LOC388436), mRNA.; novel transcript
1,23	DGKK	diacylglycerol kinase, kappa
1,23	C7orf31	chromosome 7 open reading frame 31
1,23	PLEKHA8P1	pleckstrin homology domain containing, family A member 8 pseudogene 1
1,23	CLEC4M	C-type lectin domain family 4, member M
1,23	NFE4	nuclear factor, erythroid 4
1,23	C1QTNF9B	C1q and tumor necrosis factor related protein 9B
1,23	FAM45A	Transcript Identified by AceView, Entrez Gene ID(s) 404636
1,23	NLRP12	NLR family, pyrin domain containing 12
1,23	VN1R3	vomeronasal 1 receptor 3 (gene/pseudogene)
1,23	PLN	phospholamban
1,23	FCRL4	Fc receptor-like 4
1,23	AGR2	anterior gradient 2, protein disulphide isomerase family member
1,23	CCDC105	coiled-coil domain containing 105
1,23	IZUMO1R	IZUMO1 receptor, JUNO
1,23	PPP2R4	protein phosphatase 2A activator, regulatory subunit 4
1,23	C17orf77	chromosome 17 open reading frame 77
1,23	ATOH1	atonal bHLH transcription factor 1
1,23	ZNF28	zinc finger protein 28
1,23	FPGT	fucose-1-phosphate guanylyltransferase
1,23	FGF18	fibroblast growth factor 18
1,23	RIIAD1	regulatory subunit of type II PKA R-subunit (RIIa) domain containing 1
1,23	RRP12	Zhang2013 ALT_ACCEPTOR, ALT_DONOR, coding, INTERNAL, intronic best transcript NM_015179
1,23	ZNF546	zinc finger protein 546
1,23	B4GALNT2	beta-1,4-N-acetyl-galactosaminyl transferase 2
1,23	PRKG1	Memczak2013 ALT_ACCEPTOR, ALT_DONOR, coding, INTERNAL, intronic best transcript NM_001098512
1,23	ZNF676	zinc finger protein 676
1,23	IL1RL2	interleukin 1 receptor-like 2
1,23	MEIG1	meiosis/spermiogenesis associated 1
1,22	BDH1	3-hydroxybutyrate dehydrogenase, type 1
1,22	C11orf94	chromosome 11 open reading frame 94

1,22	GOLGA6L22; GOLGA6L1	golgin A6 family-like 22; golgin A6 family-like 1
1,22	ASPHD2	aspartate beta-hydroxylase domain containing 2
1,22	UNC93A	unc-93 homolog A (C. elegans)
1,22	WNT5A	wingless-type MMTV integration site family, member 5A
1,22	MIR1-1HG; MIR133A2	MIR1-1 host gene; microRNA 133a-2
1,22	GRM8	glutamate receptor, metabotropic 8
1,22	IDO1	indoleamine 2,3-dioxygenase 1
1,22	PROCA1	protein interacting with cyclin A1
1,22	NAF1	nuclear assembly factor 1 ribonucleoprotein
1,22	C3orf52	chromosome 3 open reading frame 52
1,22	KBTBD13	kelch repeat and BTB (POZ) domain containing 13
1,22	EGR4	early growth response 4
1,22	LOC79160; RP5-907D15.4	uncharacterized LOC79160; novel transcript
1,22	XIRP1	xin actin binding repeat containing 1
1,22	B4GALT3	UDP-Gal:betaGlcNAc beta 1,4- galactosyltransferase, polypeptide 3
1,22	ANGEL1	angel homolog 1 (Drosophila)
1,22	SOHLH2	spermatogenesis and oogenesis specific basic helix-loop-helix 2
1,22	EFEMP1	EGF containing fibulin-like extracellular matrix protein 1
1,22	COL22A1	collagen, type XXII, alpha 1
1,22	ANXA9	annexin A9
1,22	GPX3	glutathione peroxidase 3
1,22	TNN	tenascin N
1,22	ARMC7	armadillo repeat containing 7
1,22	C22orf34	chromosome 22 open reading frame 34
1,22	C11orf91	chromosome 11 open reading frame 91
1,22	ALG3	ALG3, alpha-1,3- mannosyltransferase
1,22	TMSB15B	thymosin beta 15B
1,22	OR52J3	olfactory receptor, family 52, subfamily J, member 3
1,22	IFNGR2	Memczak2013 ALT_ACCEPTOR, ALT_DONOR, coding, INTERNAL, intronic best transcript NM_005534
1,22	TRIM72	tripartite motif containing 72, E3 ubiquitin protein ligase
1,22	CASS4	Cas scaffolding protein family member 4
1,22	APOC4-APOC2; APOC2	APOC4-APOC2 readthrough (NMD candidate); apolipoprotein C-II
1,22	GRIN3B	glutamate receptor, ionotropic, N-methyl-D-aspartate 3B
1,22	KRTAP9-3	keratin associated protein 9-3
1,22	SMAD6	SMAD family member 6
1,22	NDUFA8	Transcript Identified by AceView, Entrez Gene ID(s) 4702
1,22	BLK	BLK proto-oncogene, Src family tyrosine kinase
1,22	LECT1	leukocyte cell derived chemotaxin 1

1,22	CDH17	cadherin 17, LI cadherin (liver-intestine)
1,22	CCDC148	coiled-coil domain containing 148
1,22	MCF2	MCF.2 cell line derived transforming sequence
1,22	ITGBL1	integrin beta like 1
1,22	ANK1	ankyrin 1, erythrocytic
1,22	MAGEA10	MAGE family member A10
1,22	FAM90A1	family with sequence similarity 90, member A1
1,22	FHL3	four and a half LIM domains 3
1,22	COQ3	coenzyme Q3 methyltransferase
1,22	VCAM1	vascular cell adhesion molecule 1
1,22	ADD3	adducin 3 (gamma)
1,22	PRYP4	PTPN13-like, Y-linked pseudogene 4
1,22	WSCD1	WSC domain containing 1
1,22	TAF1	Transcript Identified by AceView, Entrez Gene ID(s) 6872
1,22	NCOA7	Transcript Identified by AceView, Entrez Gene ID(s) 135112
1,22	ADRB1	adrenoceptor beta 1
1,22	PLBD1	Memczak2013 ANTISENSE, coding, INTERNAL, intronic best transcript NM_024829
1,22	ALDH1L1	aldehyde dehydrogenase 1 family, member L1
1,22	RAB9B	RAB9B, member RAS oncogene family
1,22	C4BPA	complement component 4 binding protein, alpha
1,22	ZNF75A	zinc finger protein 75a
1,22	ST5	Transcript Identified by AceView, Entrez Gene ID(s) 6764
1,22	KIR2DS5; KIR3DL2; KIR2DL1; KIR3DL3; KIR3DL1; KIR2DS4; KIR2DL3; KIR3DS1; KIR2DS2; KIR2DP1; KIR3DP1	killer cell immunoglobulin-like receptor, two domains, short cytoplasmic tail, 5; killer cell immunoglobulin-like receptor, three domains, long cytoplasmic tail, 2; killer cell immunoglobulin-like receptor, two domains, long cytoplasmic tail, 1; killer cell immunoglobulin-like receptor, three domains, long cytoplasmic tail, 3; killer cell immunoglobulin-like receptor, three domains, long cytoplasmic tail, 1; killer cell immunoglobulin-like receptor, two domains, short cytoplasmic tail, 4; killer cell immunoglobulin-like receptor, two domains, long cytoplasmic tail, 3; killer cell immunoglobulin-like receptor, three domains, short cytoplasmic tail, 1; killer cell immunoglobulin-like receptor, two domains, short cytoplasmic tail, 2; killer cell immunoglobulin-like receptor, two domains, pseudogene 1; killer cell immunoglobulin-like receptor, three domains, pseudogene 1
1,22	ZNF280D	zinc finger protein 280D
1,22	SLC30A4	solute carrier family 30 (zinc transporter), member 4
1,22	NRK	Nik related kinase
1,22	PRSS2	protease, serine, 2 (trypsin 2)
1,22	FAM71C	family with sequence similarity 71, member C
1,22	SH3GL2	SH3-domain GRB2-like 2

1,22	SH3BGRL2	Transcript Identified by AceView, Entrez Gene ID(s) 83699
1,22	OR8J1	olfactory receptor, family 8, subfamily J, member 1
1,22	C15orf39	chromosome 15 open reading frame 39
1,22	ITIH2	inter-alpha-trypsin inhibitor heavy chain 2
1,22	NECAB1	N-terminal EF-hand calcium binding protein 1
1,22	GRIA1	glutamate receptor, ionotropic, AMPA 1
1,22	PEX14	peroxisomal biogenesis factor 14
1,22	DIS3L2	DIS3 like 3-5 exoribonuclease 2
1,22	TRMT10B	tRNA methyltransferase 10B
1,22	CFAP73	cilia and flagella associated protein 73
1,22	ND3	NADH dehydrogenase, subunit 3 (complex I)
1,22	ANO4	anoctamin 4
1,22	ADAMTS14	ADAM metallopeptidase with thrombospondin type 1 motif 14
1,22	PDCD1	programmed cell death 1
1,22	RIN1	Ras and Rab interactor 1
1,22	ago-04	argonaute RISC catalytic component 4
1,22	MMEL1	membrane metallo-endopeptidase-like 1
1,22	NDUFA10	NADH dehydrogenase (ubiquinone) 1 alpha subcomplex, 10, 42kDa
1,22	SNCAIP	synuclein alpha interacting protein
1,22	HAVCR2	hepatitis A virus cellular receptor 2
1,22	CIART	circadian associated repressor of transcription
1,22	ACP5	acid phosphatase 5, tartrate resistant
1,22	OR9A2	olfactory receptor, family 9, subfamily A, member 2
1,22	SLC25A53	solute carrier family 25, member 53
1,22	C19orf35	chromosome 19 open reading frame 35
1,22	CMTM7	CKLF-like MARVEL transmembrane domain containing 7
1,22	CAMKV	CaM kinase-like vesicle-associated
1,22	OR11H1	olfactory receptor, family 11, subfamily H, member 1
1,22	EGLN3	egl-9 family hypoxia-inducible factor 3
1,22	STK40	serine/threonine kinase 40
1,22	RASEF	RAS and EF-hand domain containing
1,22	GPR6	G protein-coupled receptor 6
1,22	KLHL12	kelch-like family member 12
1,22	WBSCR28	Williams-Beuren syndrome chromosome region 28
1,22	TNK2	tyrosine kinase, non-receptor, 2
1,22	ADHFE1; C8orf46	alcohol dehydrogenase, iron containing 1; chromosome 8 open reading frame 46
1,22	ZNF671	zinc finger protein 671
1,22	FOXB1	forkhead box B1
1,21	CNKSR3	CNKSR family member 3
1,21	ATP6AP1L	ATPase, H+ transporting, lysosomal accessory protein 1-like
1,21	CECR5	cat eye syndrome chromosome region, candidate 5
1,21	FUT10	fucosyltransferase 10 (alpha (1,3) fucosyltransferase)
1,21	DEFB128	defensin, beta 128

1,21	CNTNAP3B	Salzman2013 ANNOTATED, CDS, coding, INTERNAL, OVCODE, OVEXON best transcript NM_001201380
1,21	CCDC30	coiled-coil domain containing 30
1,21	THAP6	THAP domain containing 6
1,21	OVOL3	ovo-like zinc finger 3
1,21	ZNF384	zinc finger protein 384
1,21	ZKSCAN7	zinc finger with KRAB and SCAN domains 7
1,21	LIPG	lipase, endothelial
1,21	C5AR1	complement component 5a receptor 1
1,21	ZNF626	zinc finger protein 626
1,21	OR2T2	olfactory receptor, family 2, subfamily T, member 2
1,21	RHOU	ras homolog family member U
1,21	CHRM5	cholinergic receptor, muscarinic 5
1,21	HRCT1	histidine rich carboxyl terminus 1
1,21	ZNF780A	zinc finger protein 780A
1,21	GDPD4	glycerophosphodiester phosphodiesterase domain containing 4
1,21	SKAP2	src kinase associated phosphoprotein 2
1,21	DUSP18	dual specificity phosphatase 18
1,21	FBXL5	F-box and leucine-rich repeat protein 5
1,21	DLEU7	deleted in lymphocytic leukemia, 7
1,21	NR2E3	nuclear receptor subfamily 2, group E, member 3
1,21	NT5E	5-nucleotidase, ecto (CD73)
1,21	NARS2	asparaginyl-tRNA synthetase 2, mitochondrial (putative)
1,21	IL6	interleukin 6
1,21	FAM231A; FAM231C; RP5-1182A14.6	Homo sapiens family with sequence similarity 231, member A (FAM231A), mRNA.; Homo sapiens family with sequence similarity 231, member C (FAM231C), mRNA.; Protein FAM231A/C [Source:UniProtKB/Swiss-Prot;Acc:A6NEA5]
1,21	UFSP2	UFM1-specific peptidase 2
1,21	THRAP3	Jeck2013 ALT_ACCEPTOR, ALT_DONOR, coding, INTERNAL, intronic best transcript NM_005119
1,21	AGBL1	ATP/GTP binding protein-like 1
1,21	GAS8	Memczak2013 ALT_ACCEPTOR, ALT_DONOR, INTERNAL, intronic, ncRNA best transcript NR_023348
1,21	LOC285692; CTD-2143L24.1; TAS2R1	uncharacterized LOC285692; novel transcript; Transcript Identified by AceView, Entrez Gene ID(s) 285692; 50834
1,21	LOC79999; LOC388436; AC007952.5	Homo sapiens uncharacterized LOC79999 (LOC79999), mRNA.; uncharacterized protein ENSP00000382042; novel transcript
1,21	NEUROD6	neuronal differentiation 6
1,21	C2orf42	chromosome 2 open reading frame 42
1,21	TNP1	transition protein 1 (during histone to protamine replacement)
1,21	HSD11B2	hydroxysteroid (11-beta) dehydrogenase 2
1,21	STOX1	storkhead box 1

1,21	CUX2	cut-like homeobox 2
1,21	VOPP1	Jeck2013 ALT_ACCEPTOR, ALT_DONOR, coding, INTERNAL, intronic best transcript NM_030796
1,21	KLHL14	kelch-like family member 14
1,21	HIGD1B	HIG1 hypoxia inducible domain family, member 1B
1,21	GRID2	glutamate receptor, ionotropic, delta 2
1,21	SHC3	SHC (Src homology 2 domain containing) transforming protein 3
1,21	ARHGEF38	Rho guanine nucleotide exchange factor 38
1,21	IL16	interleukin 16
1,21	TMEM217	transmembrane protein 217
1,21	EIF3F	Eukaryotic translation initiation factor 3 subunit F [Source:UniProtKB/Swiss-Prot;Acc:O00303]
1,21	TTC6	tetratricopeptide repeat domain 6
1,21	PLIN2	perilipin 2
1,21	C4B	complement component 4B (Chido blood group)
1,21	C4A	complement component 4A (Rodgers blood group)
1,21	TMEM206	transmembrane protein 206
1,21	SLC2A8	solute carrier family 2 (facilitated glucose transporter), member 8
1,21	PPP1R3E	protein phosphatase 1, regulatory subunit 3E
1,21	RAB19	RAB19, member RAS oncogene family
1,21	KIF5A	kinesin family member 5A
1,21	LRRC25	leucine rich repeat containing 25
1,21	FOXE3	forkhead box E3
1,21	NOX3	NADPH oxidase 3
1,21	HIGD2B	HIG1 hypoxia inducible domain family, member 2B
1,21	ZNF660	zinc finger protein 660
1,21	INPP5D	inositol polyphosphate-5-phosphatase D
1,21	SLC2A6	solute carrier family 2 (facilitated glucose transporter), member 6
1,21	LDLRAD1	low density lipoprotein receptor class A domain containing 1
1,21	AGPAT4	1-acylglycerol-3-phosphate O-acyltransferase 4
1,21	SERPINE2	serpin peptidase inhibitor, clade E (nexin, plasminogen activator inhibitor type 1), member 2
1,21	CSF1R	colony stimulating factor 1 receptor
1,21	PCDH11X	protocadherin 11 X-linked
1,21	APOL6	apolipoprotein L, 6
1,21	UBE2L6	ubiquitin-conjugating enzyme E2L 6
1,21	C1orf127	chromosome 1 open reading frame 127
1,21	IRX2	Transcript Identified by AceView, Entrez Gene ID(s) 153572
1,21	NDUFV3	Transcript Identified by AceView, Entrez Gene ID(s) 4731
1,21	JAKMIP1	janus kinase and microtubule interacting protein 1
1,21	SCNN1B	sodium channel, non voltage gated 1 beta subunit
1,21	ENKUR	enkurin, TRPC channel interacting protein
1,21	RAPGEF5	Rap guanine nucleotide exchange factor 5
1,21	MYEOV	myeloma overexpressed

1,21	CPA5	carboxypeptidase A5
1,21	BST1	bone marrow stromal cell antigen 1
1,21	CARD17	caspase recruitment domain family, member 17
1,21	LINC01590; SMIM8	long intergenic non-protein coding RNA 1590; small integral membrane protein 8
1,21	GSTA3	glutathione S-transferase alpha 3
1,21	PXYLP1	2-phosphoxylose phosphatase 1
1,21	TAGLN3	transgelin 3
1,21	ISLR2	immunoglobulin superfamily containing leucine-rich repeat 2
1,21	ZNF720	zinc finger protein 720
1,21	ZNF253	zinc finger protein 253
1,21	RNF144B	ring finger protein 144B
1,21	GPRIN2	G protein regulated inducer of neurite outgrowth 2
1,21	KRTDAP	keratinocyte differentiation-associated protein
1,21	UTY	ubiquitously transcribed tetratricopeptide repeat containing, Y-linked
1,21	PCM1	Transcript Identified by AceView, Entrez Gene ID(s) 5108
1,21	LINC01205	long intergenic non-protein coding RNA 1205
1,21	IL25	interleukin 25
1,21	SERPINB12	serpin peptidase inhibitor, clade B (ovalbumin), member 12
1,21	ERCC2	excision repair cross-complementation group 2
1,21	KRTAP19-7	keratin associated protein 19-7
1,21	F2R	coagulation factor II (thrombin) receptor
1,21	CBX8	chromobox homolog 8
1,21	ABHD12B	abhydrolase domain containing 12B
1,21	CASP14	caspase 14
1,21	LOC100128554; RP5-944M2.3	uncharacterized LOC100128554; Transcript Identified by AceView, Entrez Gene ID(s) 144678; 100128554; novel transcript
1,21	CLDN12	claudin 12
1,21	MYOM2	myomesin 2
1,21	RTBDN	retbindin
1,21	C6orf10	chromosome 6 open reading frame 10
1,21	OR56A1	olfactory receptor, family 56, subfamily A, member 1
1,21	COG7	component of oligomeric golgi complex 7
1,21	PAPPA2	pappalysin 2
1,21	C16orf95	chromosome 16 open reading frame 95
1,21	DACT1	Transcript Identified by AceView, Entrez Gene ID(s) 51339
1,20	BCL2L14	BCL2-like 14 (apoptosis facilitator)
1,20	AMPH	amphiphysin
1,20	SLC12A3	solute carrier family 12 (sodium/chloride transporter), member 3
1,20	IL27	interleukin 27
1,20	SERTAD4	SERTA domain containing 4
1,20	LGR5	leucine-rich repeat containing G protein-coupled receptor 5

1,20	OR4D5	olfactory receptor, family 4, subfamily D, member 5
1,20	ZNF732	zinc finger protein 732
1,20	ZNF573	zinc finger protein 573
1,20	DYSF; kydor	Memczak2013 ANTISENSE, coding, INTERNAL, UTR5 best transcript NM_003494; Transcript Identified by AceView
1,20	BEND5	BEN domain containing 5
1,20	TNFRSF8	tumor necrosis factor receptor superfamily, member 8
1,20	CEACAM5	carcinoembryonic antigen-related cell adhesion molecule 5
1,20	NPHP1	nephronophthisis 1 (juvenile)
1,20	FAM155A	Jeck2013 ALT_ACCEPTOR, ALT_DONOR, coding, INTERNAL, intronic best transcript NM_001080396
1,20	SPINK1	serine peptidase inhibitor, Kazal type 1
1,20	USP17L10	ubiquitin specific peptidase 17-like family member 10
1,20	FLRT3	fibronectin leucine rich transmembrane protein 3
1,20	DGKD	diacylglycerol kinase, delta 130kDa
1,20	LIN37	lin-37 DREAM MuvB core complex component
1,20	NCKAP1L	NCK-associated protein 1-like
1,20	MARS2	methionyl-tRNA synthetase 2, mitochondrial
1,20	ABCA13	Memczak2013 ALT_ACCEPTOR, ALT_DONOR, coding, INTERNAL, intronic best transcript NM_152701
1,20	TGM4	transglutaminase 4
1,20	ZNF140	zinc finger protein 140
1,20	SAG	S-antigen; retina and pineal gland (arrestin)
1,20	KCNAB1	potassium channel, voltage gated subfamily A regulatory beta subunit 1
1,20	FUBP3	Zhang2013 ALT_ACCEPTOR, ALT_DONOR, coding, INTERNAL, intronic best transcript NM_003934
1,20	GPR26	G protein-coupled receptor 26
1,20	BPIFA1	BPI fold containing family A, member 1
1,20	DPP6	dipeptidyl-peptidase 6
1,20	VSTM5	V-set and transmembrane domain containing 5
1,20	TMEM147-AS1	TMEM147 antisense RNA 1
1,20	USP41	ubiquitin specific peptidase 41
1,20	ADGRF4	adhesion G protein-coupled receptor F4
1,20	STRA13	stimulated by retinoic acid 13
1,20	RFX3	Memczak2013 ALT_ACCEPTOR, ALT_DONOR, coding, INTERNAL, intronic best transcript NM_002919
1,20	IRX2	iroquois homeobox 2

1,20	GAGE12F; GAGE12J; GAGE12D; GAGE5; GAGE6; GAGE12B; GAGE4; GAGE2E	G antigen 12F; G antigen 12J; G antigen 12D; G antigen 5; G antigen 6; G antigen 12B; G antigen 4; G antigen 2E
1,20	DZANK1	double zinc ribbon and ankyrin repeat domains 1
1,20	ESM1	endothelial cell-specific molecule 1
1,20	C12orf80	chromosome 12 open reading frame 80
1,20	ZNF408	zinc finger protein 408
1,20	AGXT2	alanine--glyoxylate aminotransferase 2
1,20	BEST1	bestrophin 1
1,20	OR2A1	olfactory receptor, family 2, subfamily A, member 1
1,20	ROGDI	rogdi homolog
1,20	STOML3	stomatin (EPB72)-like 3
1,20	TIMM50	translocase of inner mitochondrial membrane 50 homolog (S. cerevisiae)
1,20	SLC6A1	solute carrier family 6 (neurotransmitter transporter), member 1
1,20	RASSF1-AS1	RASSF1 antisense RNA 1
1,20	NCAM1	neural cell adhesion molecule 1
1,20	OTX1	orthodenticle homeobox 1
1,20	MBD3L2; CTB-25J19.1	Synthetic construct Homo sapiens clone IMAGE:100069019, MGC:199030 methyl-CpG binding domain protein 3-like 2 (MBD3L2) mRNA, encodes complete protein.; novel transcript
1,20	TRPC5	transient receptor potential cation channel, subfamily C, member 5
1,20	MT1HL1	metallothionein 1H-like 1
1,20	POU3F2	POU class 3 homeobox 2
1,20	FMN1	formin 1
1,20	GRN	Memczak2013 ANTISENSE, CDS, coding, INTERNAL best transcript NM_002087
1,20	VAX2	ventral anterior homeobox 2
1,20	MVB12A; BISPR	multivesicular body subunit 12A; BST2 interferon stimulated positive regulator (non-protein coding)
1,20	RRP12	Memczak2013 ALT_ACCEPTOR, ALT_DONOR, coding, INTERNAL, intronic best transcript NM_001145114
1,20	MS4A10	membrane-spanning 4-domains, subfamily A, member 10
1,20	ADAM9	ADAM metallopeptidase domain 9
1,20	ELSPBP1	epididymal sperm binding protein 1
1,20	VSIG10	V-set and immunoglobulin domain containing 10
1,20	OR9Q1	olfactory receptor, family 9, subfamily Q, member 1
1,20	PMM1	phosphomannomutase 1
1,20	PDZD4	PDZ domain containing 4
1,20	MBD3L2	methyl-CpG binding domain protein 3-like 2

1,20	WNT10B	wingless-type MMTV integration site family, member 10B
1,20	OR4A8	olfactory receptor, family 4, subfamily A, member 8 (gene/pseudogene) [Source:HGNC Symbol;Acc:HGNC:15165]
1,20	CRISP1	cysteine-rich secretory protein 1
1,20	LCORL	Transcript Identified by AceView, Entrez Gene ID(s) 254251
1,20	SMTNL2	smoothelin-like 2
1,20	C19orf81	chromosome 19 open reading frame 81
1,20	CSMD1	Transcript Identified by AceView, Entrez Gene ID(s) 64478
1,20	TRIM9	tripartite motif containing 9
1,20	TMEM45B	transmembrane protein 45B
1,20	ZDHHC1	zinc finger, DHHC-type containing 1
1,20	OR1A2	olfactory receptor, family 1, subfamily A, member 2
1,20	TNNC2	troponin C type 2 (fast)
1,20	ECHDC3	enoyl-CoA hydratase domain containing 3
1,20	CD1C	CD1c molecule
1,20	CSRP1; markobu	Memczak2013 ALT_ACCEPTOR, ALT_DONOR, coding, INTERNAL, intronic best transcript NM_001193571; Transcript Identified by AceView
1,20	SERTAD4	SERTA domain containing 4
1,20	CYP19A1	cytochrome P450, family 19, subfamily A, polypeptide 1
1,20	FAM181A	family with sequence similarity 181, member A
1,20	ARID3C	AT rich interactive domain 3C (BRIGHT-like)
1,20	ASMT; AKAP17A	acetylserotonin O-methyltransferase; A kinase (PRKA) anchor protein 17A
1,20	ASMT; SFRS17AandASMT	Homo sapiens acetylserotonin O-methyltransferase (ASMT), transcript variant 1, mRNA.; Homo sapiens acetylserotonin O-methyltransferase (ASMT), transcript variant 3, mRNA.; acetylserotonin O-methyltransferase [Source:HGNC Symbol;Acc:HGNC:750]; Homo sapiens acetylserotonin O-methyltransferase, mRNA (cDNA clone MGC:2145 IMAGE:3356470), complete cds.; Transcript Identified by AceView, Entrez Gene ID(s) 438; 8227, RefSeq ID(s) NM_001171038
1,20	SLC17A8	solute carrier family 17 (vesicular glutamate transporter), member 8
1,20	TAS2R60	taste receptor, type 2, member 60
1,20	HAMP	hepcidin antimicrobial peptide
1,20	SLC4A10	solute carrier family 4, sodium bicarbonate transporter, member 10
1,20	PCDHB12	protocadherin beta 12
1,20	NAA11	N(alpha)-acetyltransferase 11, NatA catalytic subunit
1,20	GPRASP1	G protein-coupled receptor associated sorting protein 1
1,20	RCE1; C11orf80	Ras converting CAAX endopeptidase 1; chromosome 11 open reading frame 80

1,20	N4BP3	NEDD4 binding protein 3
1,20	ENPP7	ectonucleotide pyrophosphatase/phosphodiesterase 7
1,20	PRAMEF15; PRAMEF9	PRAME family member 15; PRAME family member 9
1,20	CCL27	chemokine (C-C motif) ligand 27
1,20	TRIM47	tripartite motif containing 47
1,20	C1QTNF5; MFRP	C1q and tumor necrosis factor related protein 5; membrane frizzled-related protein
1,20	CDCA7	cell division cycle associated 7
1,20	GTPBP3	GTP binding protein 3 (mitochondrial)
1,20	TARM1	T cell-interacting, activating receptor on myeloid cells 1
1,20	OR5L1	olfactory receptor, family 5, subfamily L, member 1 (gene/pseudogene)
1,20	RXRG	retinoid X receptor gamma
1,20	ELMOD3	Transcript Identified by AceView, Entrez Gene ID(s) 84173
1,20	BRINP3	bone morphogenetic protein/retinoic acid inducible neural-specific 3
1,20	MECR	mitochondrial trans-2-enoyl-CoA reductase
1,20	PLAGL2	pleiomorphic adenoma gene-like 2
1,20	TMEM198	transmembrane protein 198
1,20	CDYL2	chromodomain protein, Y-like 2
1,20	PARS2	prolyl-tRNA synthetase 2, mitochondrial (putative)
1,20	SPATA31A3	SPATA31 subfamily A, member 3
1,20	SSX2B	synovial sarcoma, X breakpoint 2B
1,20	LYSMD1	LysM, putative peptidoglycan-binding, domain containing 1
1,20	MAP4K2	Memczak2013 ANTISENSE, CDS, coding, INTERNAL, intronic best transcript NM_004579
1,20	USP29	ubiquitin specific peptidase 29
1,20	ACTBL2	actin, beta-like 2
1,20	RAB31	Transcript Identified by AceView, Entrez Gene ID(s) 11031
1,20	ZSWIM1	zinc finger, SWIM-type containing 1
1,20	LIAS	lipoic acid synthetase
1,20	ICA1	islet cell autoantigen 1
1,20	TMEM241	transmembrane protein 241
1,20	ADGRL4	adhesion G protein-coupled receptor L4
1,20	RASGRF2	Jeck2013 ALT_ACCEPTOR, ALT_DONOR, coding, INTERNAL, intronic best transcript NM_006909
1,20	KCNAB3	potassium channel, voltage gated subfamily A regulatory beta subunit 3
1,20	REC8	REC8 meiotic recombination protein
1,20	CC2D2A	Transcript Identified by AceView, Entrez Gene ID(s) 57545
1,20	FAM26E	family with sequence similarity 26, member E
1,20	OR56A3	olfactory receptor, family 56, subfamily A, member 3
1,19	PORCN	porcupine homolog (Drosophila)
1,19	USP17L21	ubiquitin specific peptidase 17-like family member 21

1,19	NEDD1	neural precursor cell expressed, developmentally down-regulated 1
1,19	TNNT1	troponin T type 1 (skeletal, slow)
1,19	ZNF98	zinc finger protein 98
1,19	DQX1	DEAQ box RNA-dependent ATPase 1
1,19	BRDT	bromodomain, testis-specific
1,19	ODF4	outer dense fiber of sperm tails 4
1,19	PFN3	profilin 3
1,19	SFTA2	surfactant associated 2
1,19	CLASP2	Transcript Identified by AceView, Entrez Gene ID(s) 23122
1,19	PXDN	Transcript Identified by AceView, Entrez Gene ID(s) 7837
1,19	PUS10	pseudouridylate synthase 10
1,19	KLHL33	kelch-like family member 33
1,19	GPRC5A; MIR614	G protein-coupled receptor, class C, group 5, member A; microRNA 614
1,19	PYCARD-AS1	PYCARD antisense RNA 1
1,19	RBM46	RNA binding motif protein 46
1,19	LY75-CD302; CD302; LY75	LY75-CD302 readthrough; CD302 molecule; lymphocyte antigen 75
1,19	DMKN	dermokine
1,19	AJUBA	ajuba LIM protein
1,19	KCNJ3	potassium channel, inwardly rectifying subfamily J, member 3
1,19	ABCB11	ATP binding cassette subfamily B member 11
1,19	USP17L28; USP17L5; USP17L24	ubiquitin specific peptidase 17-like family member 28; ubiquitin specific peptidase 17-like family member 5; ubiquitin specific peptidase 17-like family member 24
1,19	LIPK	lipase, family member K
1,19	KCNJ18	potassium channel, inwardly rectifying subfamily J, member 18
1,19	C6orf201	chromosome 6 open reading frame 201
1,19	PGPEP1L	pyroglutamyl-peptidase I-like
1,19	EZH1	enhancer of zeste 1 polycomb repressive complex 2 subunit
1,19	ANKRD20A4	ankyrin repeat domain 20 family, member A4
1,19	JAKMIP2-AS1	JAKMIP2 antisense RNA 1
1,19	OR10G4	olfactory receptor, family 10, subfamily G, member 4
1,19	KCNJ4	potassium channel, inwardly rectifying subfamily J, member 4
1,19	FBXO8	Transcript Identified by AceView, Entrez Gene ID(s) 26269
1,19	ANKRD20A3; ANKRD20A2	ankyrin repeat domain 20 family, member A3; ankyrin repeat domain 20 family, member A2
1,19	DGAT2	diacylglycerol O-acyltransferase 2
1,19	DAPK1	death-associated protein kinase 1
1,19	ZNF808; RPL39P34	zinc finger protein 808; ribosomal protein L39 pseudogene 34
1,19	FAM47B	family with sequence similarity 47, member B

1,19	LYPD3	LY6/PLAUR domain containing 3
1,19	RP11-295G24.4; GFI1B	Transcript Identified by AceView, Entrez Gene ID(s) 8328; novel transcript
1,19	PLIN5	perilipin 5
1,19	C8B	complement component 8, beta polypeptide
1,19	PRPS1L1	phosphoribosyl pyrophosphate synthetase 1-like 1
1,19	CLVS2	clavesin 2
1,19	LOC100130357; RP1-257A7.4	uncharacterized LOC100130357; novel transcript, antisense to PHACTR1
1,19	DEFB134	defensin, beta 134
1,19	FCN3	ficolin (collagen/fibrinogen domain containing) 3
1,19	ANO2	anoctamin 2, calcium activated chloride channel
1,19	SNX15	sorting nexin 15
1,19	OR51B4	olfactory receptor, family 51, subfamily B, member 4
1,19	SLFN11	schlafen family member 11
1,19	KRTCAP3	keratinocyte associated protein 3
1,19	GAPT	GRB2-binding adaptor protein, transmembrane
1,19	IL18RAP	interleukin 18 receptor accessory protein
1,19	HNRNPCL2	heterogeneous nuclear ribonucleoprotein C-like 2 [Source:HGNC Symbol;Acc:HGNC:48813]
1,19	GOLGA7B	golgin A7 family, member B
1,19	CLDN1	claudin 1
1,19	RUNX1	runt-related transcription factor 1
1,19	ZNF736	zinc finger protein 736
1,19	HDAC6	Memczak2013 ANTISENSE, CDS, coding, INTERNAL best transcript NM_006044
1,19	ZNF546	zinc finger protein 546
1,19	CDH3	cadherin 3, type 1, P-cadherin (placental)
1,19	MS4A4E	membrane-spanning 4-domains, subfamily A, member 4E
1,19	SPDYE16	speedy/RINGO cell cycle regulator family member E16 [Source:HGNC Symbol;Acc:HGNC:51512]
1,19	DPCR1	diffuse panbronchiolitis critical region 1
1,19	OR2AG1	olfactory receptor, family 2, subfamily AG, member 1 (gene/pseudogene)
1,19	HSFY2	heat shock transcription factor, Y-linked 2
1,19	MALL	mal, T-cell differentiation protein-like
1,19	FAM180B	family with sequence similarity 180, member B
1,19	SCT	secretin
1,19	MPP5	membrane protein, palmitoylated 5
1,19	DAW1	dynein assembly factor with WDR repeat domains 1
1,19	CDH11	cadherin 11, type 2, OB-cadherin (osteoblast)
1,19	JAZF1	Memczak2013 ANTISENSE, coding, INTERNAL, intronic best transcript NM_175061
1,19	CRYBA2	crystallin beta A2
1,19	LIMK1	Memczak2013 ANTISENSE, CDS, coding, INTERNAL best transcript NM_001204426
1,19	ILDR2	immunoglobulin-like domain containing receptor 2
1,19	GSTA1	glutathione S-transferase alpha 1

1,19	FAM231C; FAM231A	family with sequence similarity 231, member C; family with sequence similarity 231, member A
1,19	RASD2	Memczak2013 ANTISENSE, coding, INTERNAL, UTR3 best transcript NM_014310
1,19	GHRHR	growth hormone releasing hormone receptor
1,19	WRB	tryptophan rich basic protein
1,19	DGKB	diacylglycerol kinase, beta 90kDa
1,19	SYT15	synaptotagmin XV
1,19	TM9SF3	transmembrane 9 superfamily member 3
1,19	DPY19L2	dpy-19-like 2 (C. elegans)
1,19	C3orf49	chromosome 3 open reading frame 49
1,19	CSRP3	cysteine and glycine-rich protein 3 (cardiac LIM protein)
1,19	PRAMEF6	PRAME family member 6 [Source:HGNC Symbol;Acc:HGNC:30583]
1,19	PRAMEF6	PRAME family member 6 [Source:HGNC Symbol;Acc:HGNC:30583]
1,19	PDE1A	Transcript Identified by AceView, Entrez Gene ID(s) 5136
1,19	SLC35A4	solute carrier family 35, member A4
1,19	OR1L4	olfactory receptor, family 1, subfamily L, member 4
1,19	APOC3	apolipoprotein C-III
1,19	TCP10L2	t-complex 10-like 2
1,19	ZPBP	zona pellucida binding protein
1,19	ADM	adrenomedullin
1,19	FAM20A	family with sequence similarity 20, member A
1,19	DYX1C1	dyslexia susceptibility 1 candidate 1
1,19	ADGRG5	adhesion G protein-coupled receptor G5
1,19	TOX3	TOX high mobility group box family member 3
1,19	IL17B	interleukin 17B
1,19	WFS1	Wolfram syndrome 1 (wolframin)
1,19	UBE2D3	Memczak2013 ANTISENSE, coding, INTERNAL, intronic best transcript NM_181893
1,19	LOC388780; RP11-128M1.1	uncharacterized LOC388780; putative novel transcript
1,19	MUC15	mucin 15, cell surface associated
1,19	FSD1L	fibronectin type III and SPRY domain containing 1-like
1,19	NTN4	netrin 4
1,19	CACNG7	calcium channel, voltage-dependent, gamma subunit 7
1,19	DNAH6	Transcript Identified by AceView, Entrez Gene ID(s) 1768
1,19	MROH7; TTC4; MROH7-TTC4	maestro heat-like repeat family member 7; tetratricopeptide repeat domain 4; MROH7-TTC4 readthrough (NMD candidate)
1,19	EARS2	glutamyl-tRNA synthetase 2, mitochondrial
1,19	SPTA1	spectrin, alpha, erythrocytic 1
1,19	OR5M9	olfactory receptor, family 5, subfamily M, member 9
1,19	FGFBP3	fibroblast growth factor binding protein 3
1,19	KCNE3	potassium channel, voltage gated subfamily E regulatory beta subunit 3

1,19	TSSK3	testis-specific serine kinase 3
1,19	AP1S1	Transcript Identified by AceView, Entrez Gene ID(s) 1174
1,19	HAO2	hydroxyacid oxidase 2 (long chain)
1,19	SHISA2	shisa family member 2
1,19	TTN	titin
1,19	KHK	ketohexokinase
1,19	OR6T1	olfactory receptor, family 6, subfamily T, member 1
1,19	SLC8A2	solute carrier family 8 (sodium/calcium exchanger), member 2
1,19	HOXD11	homeobox D11
1,19	FBL	fibrillarin
1,19	PTPN2	Transcript Identified by AceView, Entrez Gene ID(s) 5771
1,19	GABRA4	gamma-aminobutyric acid (GABA) A receptor, alpha 4
1,19	MAP3K15	mitogen-activated protein kinase kinase kinase 15
1,19	POSTN	periostin, osteoblast specific factor
1,19	GUCY2D	guanylate cyclase 2D, membrane (retina-specific)
1,19	TP53TG3C	TP53 target 3C
1,19	MARCH1	Memczak2013 ANTISENSE, coding, INTERNAL, intronic best transcript NM_001166373
1,19	CDK12	Transcript Identified by AceView, Entrez Gene ID(s) 51755
1,19	ORMDL3	ORMDL sphingolipid biosynthesis regulator 3
1,19	HOXD12	homeobox D12
1,19	IQCA1	Transcript Identified by AceView, Entrez Gene ID(s) 79781
1,19	SPATA31A7; SPATA31A5	SPATA31 subfamily A, member 7; SPATA31 subfamily A, member 5
1,19	CD34	CD34 molecule
1,19	AP4S1	adaptor-related protein complex 4, sigma 1 subunit
1,19	KAT6B	K(lysine) acetyltransferase 6B
1,19	HSPB6	heat shock protein, alpha-crystallin-related, B6
1,19	TARSL2	threonyl-tRNA synthetase-like 2
1,19	F8A2; F8A3; F8A1	coagulation factor VIII-associated 2; coagulation factor VIII-associated 3; coagulation factor VIII-associated 1
1,19	SMIM8	small integral membrane protein 8
1,19	LRFN2	leucine rich repeat and fibronectin type III domain containing 2
1,19	CCDC162P	coiled-coil domain containing 162, pseudogene
1,19	PRR35	proline rich 35
1,18	OAF	out at first homolog
1,18	MSMO1	methylsterol monooxygenase 1
1,18	KIAA1468	KIAA1468
1,18	CSN1S1	casein alpha s1
1,18	CCND1	cyclin D1
1,18	C1orf141	chromosome 1 open reading frame 141
1,18	KRBA1	KRAB-A domain containing 1

1,18	MIXL1	Mix paired-like homeobox
1,18	REM1	RAS (RAD and GEM)-like GTP-binding 1
1,18	PI15	peptidase inhibitor 15
1,18	GABRG1	gamma-aminobutyric acid (GABA) A receptor, gamma 1
1,18	VAMP1	vesicle associated membrane protein 1
1,18	TAF1L	TAF1 RNA polymerase II, TATA box binding protein (TBP)-associated factor, 210kDa-like
1,18	C3orf35	chromosome 3 open reading frame 35
1,18	GALM	galactose mutarotase (aldose 1-epimerase)
1,18	GLB1L3	galactosidase beta 1 like 3
1,18	GCLC	glutamate-cysteine ligase, catalytic subunit
1,18	MAGEA12; CSAG4	MAGE family member A12; CSAG family, member 4 (pseudogene)
1,18	LEUTX	leucine twenty homeobox
1,18	NUAK2	NUAK family, SNF1-like kinase, 2
1,18	UBE2J2	Transcript Identified by AceView, Entrez Gene ID(s) 118424
1,18	RBMY1D; RBMY1B; RBMY1A1	RNA binding motif protein, Y-linked, family 1, member D; RNA binding motif protein, Y-linked, family 1, member B; RNA binding motif protein, Y-linked, family 1, member A1
1,18	FAM19A1	family with sequence similarity 19 (chemokine (C-C motif)-like), member A1
1,18	SERTAD4	SERTA domain containing 4
1,18	FIBCD1	fibrinogen C domain containing 1
1,18	DKFZp779M0652; CTD-2210P24.4	uncharacterized DKFZp779M0652; Transcript Identified by AceView, Entrez Gene ID(s) 374387, RefSeq ID(s) NR_027134; novel transcript; uncharacterized DKFZp779M0652 [Source:EntrezGene;Acc:374387]
1,18	TMEM212	transmembrane protein 212
1,18	SPRED3	sprouty-related, EVH1 domain containing 3
1,18	ARRDC5	arrestin domain containing 5
1,18	COL24A1	collagen, type XXIV, alpha 1
1,18	KCNE1	potassium channel, voltage gated subfamily E regulatory beta subunit 1
1,18	ATP12A	ATPase, H+/K+ transporting, nongastric, alpha polypeptide
1,18	DEFA1B; DEFA1; DEFA3	defensin, alpha 1B; defensin, alpha 1; defensin, alpha 3, neutrophil-specific
1,18	DEFA1; DEFA1B	defensin, alpha 1; defensin, alpha 1B
1,18	WNT2B	wingless-type MMTV integration site family, member 2B
1,18	USP9X	Memczak2013 ANTISENSE, CDS, coding, INTERNAL best transcript NM_001039590
1,18	SEMA3E	sema domain, immunoglobulin domain (Ig), short basic domain, secreted, (semaphorin) 3E

1,18	SYT15; ABC7-42404400C24.1	Homo sapiens synaptotagmin XV (SYT15), transcript variant a, mRNA.; Homo sapiens synaptotagmin XV (SYT15), transcript variant b, mRNA.; Homo sapiens synaptotagmin XV, mRNA (cDNA clone MGC:164845 IMAGE:40147906), complete cds.; Synaptotagmin-15 [Source:UniProtKB/Swiss-Prot;Acc:Q9BQS2]
1,18	PUS7	pseudouridylate synthase 7 (putative)
1,18	CLRN1	clarin 1
1,18	ACKR4	atypical chemokine receptor 4
1,18	RBP7	retinol binding protein 7, cellular
1,18	MFSD5	major facilitator superfamily domain containing 5
1,18	KCNH3	potassium channel, voltage gated eag related subfamily H, member 3
1,18	CEP76	centrosomal protein 76kDa
1,18	FAM188A	family with sequence similarity 188, member A
1,18	SRRM5	serine/arginine repetitive matrix 5
1,18	MADD	MAP-kinase activating death domain
1,18	IQCF5	IQ motif containing F5
1,18	ARHGAP25	Rho GTPase activating protein 25
1,18	UPK1A-AS1	UPK1A antisense RNA 1
1,18	GPC6	Transcript Identified by AceView, Entrez Gene ID(s) 10082
1,18	MUC17	mucin 17, cell surface associated
1,18	LPIN1	Transcript Identified by AceView, Entrez Gene ID(s) 23175
1,18	C1orf61; RP11-98G7.1	Transcript Identified by AceView, Entrez Gene ID(s) 10485; novel transcript
1,18	GPLD1	glycosylphosphatidylinositol specific phospholipase D1
1,18	GUCY1A2	guanylate cyclase 1, soluble, alpha 2
1,18	PLA2G4E	phospholipase A2, group IVE
1,18	NRSN2-AS1	NRSN2 antisense RNA 1
1,18	POGZ	Transcript Identified by AceView, Entrez Gene ID(s) 23126
1,18	ELL3	elongation factor RNA polymerase II-like 3
1,18	PATL1	protein associated with topoisomerase II homolog 1 (yeast)
1,18	CHN2	chimerin 2
1,18	DRAXIN	dorsal inhibitory axon guidance protein
1,18	CCT8L2	chaperonin containing TCP1, subunit 8 (theta)-like 2
1,18	ODAM	odontogenic, ameloblast asssociated
1,18	ZBTB7C	zinc finger and BTB domain containing 7C
1,18	SALL1	spalt-like transcription factor 1
1,18	ALS2	ALS2, alsin Rho guanine nucleotide exchange factor
1,18	TMCO6	transmembrane and coiled-coil domains 6
1,18	AEBP1; MIR4649	AE binding protein 1; microRNA 4649
1,18	ANP32D	acidic nuclear phosphoprotein 32 family member D
1,18	SDSL	serine dehydratase-like
1,18	CTSK	cathepsin K

1,18	OR6C68	olfactory receptor, family 6, subfamily C, member 68
1,18	GAB3	GRB2-associated binding protein 3
1,18	DUOX2	dual oxidase 2
1,18	A2ML1	alpha-2-macroglobulin-like 1
1,18	LTB	lymphotoxin beta (TNF superfamily, member 3)
1,18	ACADL	acyl-CoA dehydrogenase, long chain
1,18	NOS1	nitric oxide synthase 1 (neuronal)
1,18	THSD7A	thrombospondin type 1 domain containing 7A
1,18	SPINK7	serine peptidase inhibitor, Kazal type 7 (putative)
1,18	CT55	cancer/testis antigen 55
1,18	RTN4RL1	reticulon 4 receptor-like 1
1,18	SAG	S-antigen; retina and pineal gland (arrestin)
1,18	TGIF2LX	TGFB-induced factor homeobox 2-like, X-linked
1,18	SPARCL1	SPARC like 1
1,18	TMEM30C	transmembrane protein 30C
1,18	MEIOC	meiosis specific with coiled-coil domain
1,18	TNNI3K	TNNI3 interacting kinase
1,18	CD36	CD36 molecule (thrombospondin receptor)
1,18	HRASLS5	HRAS-like suppressor family, member 5
1,18	ACBD7	acyl-CoA binding domain containing 7
1,18	GABRR1	gamma-aminobutyric acid (GABA) A receptor, rho 1
1,18	DOHH	deoxyhypusine hydroxylase/monooxygenase
1,18	FXYD2	FXYD domain containing ion transport regulator 2
1,18	CD52	CD52 molecule
1,18	LINC00851	long intergenic non-protein coding RNA 851
1,18	RGMA	repulsive guidance molecule family member a
1,18	WLS	Jeck2013 ALT_ACCEPTOR, ALT_DONOR, coding, INTERNAL, intronic, OVERLAPTX best transcript NM_024911
1,18	CST2	cystatin SA
1,18	MRS2	MRS2 magnesium transporter
1,18	REXO4	REX4 homolog, 3'-5' exonuclease [Source:HGNC Symbol;Acc:HGNC:12820]
1,18	C2orf88	chromosome 2 open reading frame 88
1,18	GAGE4; GAGE7; GAGE12G	G antigen 4; G antigen 7; G antigen 12G
1,18	ARHGEF19	Rho guanine nucleotide exchange factor 19
1,18	OLFM2	olfactomedin 2
1,18	MEOX1	mesenchyme homeobox 1
1,18	ZNF85	zinc finger protein 85
1,18	ABCC10	ATP binding cassette subfamily C member 10
1,18	SAG	S-antigen; retina and pineal gland (arrestin)
1,18	OR2V2	olfactory receptor, family 2, subfamily V, member 2
1,18	PAGE1	P antigen family, member 1 (prostate associated)
1,18	TRADD	TNFRSF1A-associated via death domain
1,18	API5L1	Transcript Identified by AceView, Entrez Gene ID(s) 642812
1,18	TTC29	tetratricopeptide repeat domain 29
1,18	SSTR4	somatostatin receptor 4

1,18	RHOBTB3	Rho-related BTB domain containing 3
1,18	EVC2	Ellis van Creveld syndrome 2
1,18	TUBA8	tubulin, alpha 8
1,18	GML	glycosylphosphatidylinositol anchored molecule like
1,18	FCGR1B	Fc fragment of IgG, high affinity Ib, receptor (CD64)
1,18	ALK	Transcript Identified by AceView, Entrez Gene ID(s) 238
1,18	SLC27A6	solute carrier family 27 (fatty acid transporter), member 6
1,18	MIF-AS1	MIF antisense RNA 1
1,18	SAXO1	stabilizer of axonemal microtubules 1
1,18	ZFP57	ZFP57 zinc finger protein
1,18	OR11H4	olfactory receptor, family 11, subfamily H, member 4
1,18	TRIM22	Memczak2013 ANTISENSE, CDS, coding, INTERNAL, UTR5 best transcript NM_001199573
1,18	OR4E1	olfactory receptor family 4 subfamily E member 1 (gene/pseudogene) [Source:HGNC Symbol;Acc:HGNC:8296]
1,18	SLC31A2	solute carrier family 31 (copper transporter), member 2
1,18	FCER1G	Fc fragment of IgE, high affinity I, receptor for; gamma polypeptide
1,18	UNG	uracil DNA glycosylase
1,18	TMEM196	transmembrane protein 196
1,18	CNRIP1	cannabinoid receptor interacting protein 1
1,18	CTGF	connective tissue growth factor
1,18	CNR1	cannabinoid receptor 1 (brain)
1,18	C10orf90	chromosome 10 open reading frame 90
1,18	LPCAT2	lysophosphatidylcholine acyltransferase 2
1,18	PCDHGC3; PCDHGA12; PCDHGB4; PCDHGA8; PCDHGA1; PCDHGA10; PCDHGA11; PCDHGA2; PCDHGA3; PCDHGA4; PCDHGA5; PCDHGA6; PCDHGA7; PCDHGA9; PCDHGB1; PCDHGB2; PCDHGB3; PCDHGB5; PCDHGB6; PCDHGB7; PCDHGC4; PCDHGC5	protocadherin gamma subfamily C, 3; protocadherin gamma subfamily A, 12; protocadherin gamma subfamily B, 4; protocadherin gamma subfamily A, 8; protocadherin gamma subfamily A, 1; protocadherin gamma subfamily A, 10; protocadherin gamma subfamily A, 11; protocadherin gamma subfamily A, 2; protocadherin gamma subfamily A, 3; protocadherin gamma subfamily A, 4; protocadherin gamma subfamily A, 5; protocadherin gamma subfamily A, 6; protocadherin gamma subfamily A, 7; protocadherin gamma subfamily A, 9; protocadherin gamma subfamily B, 1; protocadherin gamma subfamily B, 2; protocadherin gamma subfamily B, 3; protocadherin gamma subfamily B, 5; protocadherin gamma subfamily B, 6; protocadherin gamma subfamily B, 7; protocadherin gamma subfamily C, 4; protocadherin gamma subfamily C, 5

1,18	ATG16L1	autophagy related 16-like 1
1,18	SHISA4	shisa family member 4
1,18	COL20A1	collagen, type XX, alpha 1
1,18	COL6A1	collagen, type VI, alpha 1
1,18	ADGRL3	adhesion G protein-coupled receptor L3
1,18	FAM216B	family with sequence similarity 216, member B
1,18	CXCL8	chemokine (C-X-C motif) ligand 8
1,17	MOK	MOK protein kinase
1,17	CGB2	chorionic gonadotropin, beta polypeptide 2
1,17	CNGB3	cyclic nucleotide gated channel beta 3
1,17	SIGLEC7	sialic acid binding Ig-like lectin 7
1,17	AIF1L	allograft inflammatory factor 1-like
1,17	CCDC66	coiled-coil domain containing 66
1,17	CDH8	cadherin 8, type 2
1,17	LIMS3L; LIMS3	LIM and senescent cell antigen-like domains 3-like; LIM and senescent cell antigen-like domains 3
1,17	GPR151	G protein-coupled receptor 151
1,17	TSNARE1	t-SNARE domain containing 1
1,17	LRRN4	leucine rich repeat neuronal 4
1,17	PRR4	proline rich 4 (lacrimal)
1,17	IGFLR1	IGF like family receptor 1
1,17	LRTM2	leucine-rich repeats and transmembrane domains 2
1,17	MSH2	mutS homolog 2
1,17	MMRN1	multimerin 1
1,17	GNG4	guanine nucleotide binding protein (G protein), gamma 4
1,17	OSBPL1A	Transcript Identified by AceView, Entrez Gene ID(s) 114876
1,17	TTLL2	tubulin tyrosine ligase-like family member 2
1,17	SMIM10L2B	small integral membrane protein 10 like 2B
1,17	NCR1	natural cytotoxicity triggering receptor 1
1,17	EFNA2	ephrin-A2
1,17	SAMSN1	SAM domain, SH3 domain and nuclear localization signals 1
1,17	RETNLB	resistin like beta
1,17	TNFSF14	tumor necrosis factor (ligand) superfamily, member 14
1,17	PSMF1	proteasome inhibitor subunit 1
1,17	HSPG2	Jeck2013 ANTISENSE, CDS, coding, INTERNAL, intronic, OVCODE, OVEXON best transcript NM_005529
1,17	MAST1	microtubule associated serine/threonine kinase 1
1,17	RRAD	Ras-related associated with diabetes
1,17	LY6G5C	lymphocyte antigen 6 complex, locus G5C
1,17	GAGE1	G antigen 1
1,17	FAM189B	family with sequence similarity 189, member B
1,17	CEMIP	cell migration inducing protein, hyaluronan binding
1,17	DUSP15	dual specificity phosphatase 15
1,17	SLC24A2	solute carrier family 24 (sodium/potassium/calcium exchanger), member 2

1,17	DPM2	dolichyl-phosphate mannosyltransferase polypeptide 2, regulatory subunit
1,17	PPM1E	protein phosphatase, Mg2+/Mn2+ dependent, 1E
1,17	ZNF684	zinc finger protein 684
1,17	ARSH	arylsulfatase family, member H
1,17	SERPINA9	serpin peptidase inhibitor, clade A (alpha-1 antiproteinase, antitrypsin), member 9
1,17	C17orf75	chromosome 17 open reading frame 75
1,17	SYCP2L	synaptonemal complex protein 2-like
1,17	CALHM1	calcium homeostasis modulator 1
1,17	GRIA1	Transcript Identified by AceView, Entrez Gene ID(s) 2890
1,17	TGM4	transglutaminase 4
1,17	DEFB119	defensin, beta 119
1,17	MYF6	myogenic factor 6 (herculin)
1,17	GRIN3A	glutamate receptor, ionotropic, N-methyl-D-aspartate 3A
1,17	GRK1	G protein-coupled receptor kinase 1
1,17	EVPL	envoplakin
1,17	ATF7IP2	activating transcription factor 7 interacting protein 2
1,17	PRRT3	proline-rich transmembrane protein 3
1,17	CEACAM8	carcinoembryonic antigen-related cell adhesion molecule 8
1,17	ARPP21	Transcript Identified by AceView, Entrez Gene ID(s) 10777
1,17	SEC31B	SEC31 homolog B, COPII coat complex component
1,17	GABRR2	gamma-aminobutyric acid (GABA) A receptor, rho 2
1,17	LSP1; MIR7847	lymphocyte-specific protein 1; microRNA 7847
1,17	WDR26	Transcript Identified by AceView, Entrez Gene ID(s) 80232
1,17	IGF2; INS-IGF2	insulin-like growth factor 2; INS-IGF2 readthrough
1,17	MYCN	v-myc avian myelocytomatosis viral oncogene neuroblastoma derived homolog
1,17	SMCP	sperm mitochondria-associated cysteine-rich protein
1,17	PSG8	pregnancy specific beta-1-glycoprotein 8
1,17	LOC100506127; RP11-111M22.2; LOC387790	putative uncharacterized protein FLJ37770-like; Transcript Identified by AceView, Entrez Gene ID(s) 387790; novel transcript
1,17	SLC4A11	solute carrier family 4, sodium borate transporter, member 11
1,17	LILRA2	leukocyte immunoglobulin-like receptor, subfamily A (with TM domain), member 2
1,17	KRTAP13-3	keratin associated protein 13-3
1,17	SPATA6	spermatogenesis associated 6
1,17	OR2H2	olfactory receptor, family 2, subfamily H, member 2
1,17	ATG16L1	autophagy related 16-like 1
1,17	TNFRSF17	tumor necrosis factor receptor superfamily, member 17
1,17	PRAMEF6	PRAME family member 6 [Source:HGNC Symbol;Acc:HGNC:30583]
1,17	RAD9A	RAD9 checkpoint clamp component A

1,17	PGBD5	piggyBac transposable element derived 5
1,17	SOX8	SRY box 8
1,17	FBXO16	F-box protein 16
1,17	PDIK1L	PDLIM1 interacting kinase 1 like
1,17	SMAGP	small cell adhesion glycoprotein
1,17	PP2D1	protein phosphatase 2C-like domain containing 1
1,17	AFAP1L2	actin filament associated protein 1-like 2
1,17	CTXN2	cortexin 2
1,17	CBLN2	cerebellin 2 precursor
1,17	GAS6	growth arrest-specific 6
1,17	AKR1B15	aldo-keto reductase family 1, member B15
1,17	ZNF775	zinc finger protein 775
1,17	PID1	phosphotyrosine interaction domain containing 1
1,17	MGAM	maltase-glucoamylase
1,17	TXLNB	taxilin beta
1,17	GALR1	galanin receptor 1
1,17	MAGED4; MAGED4B; SNORA11D; SNORA11E	melanoma antigen family D4; melanoma antigen family D4B; small nucleolar RNA, H/ACA box 11D; small nucleolar RNA, H/ACA box 11E
1,17	SCN11A	sodium channel, voltage gated, type XI alpha subunit
1,17	BMPR1B	bone morphogenetic protein receptor type IB
1,17	PSG3	pregnancy specific beta-1-glycoprotein 3
1,17	BFSP2	beaded filament structural protein 2, phakinin
1,17	ZNF208	zinc finger protein 208
1,17	OCA2	oculocutaneous albinism II
1,17	PLEKHH3	pleckstrin homology domain containing, family H (with MyTH4 domain) member 3
1,17	KLRC2	killer cell lectin-like receptor subfamily C, member 2
1,17	SEPN1	selenoprotein N, 1
1,17	POGZ	Transcript Identified by AceView, Entrez Gene ID(s) 23126
1,17	RBM11	RNA binding motif protein 11
1,17	PLEK	pleckstrin
1,17	SLC44A4	solute carrier family 44, member 4
1,17	PAQR5	progestin and adipoQ receptor family member V
1,17	WNT11	wingless-type MMTV integration site family, member 11
1,17	TEX28	testis expressed 28
1,17	ZNF491	zinc finger protein 491
1,17	CST6	cystatin E/M
1,17	AMY2A	amylase, alpha 2A (pancreatic)
1,17	CHST13	carbohydrate (chondroitin 4) sulfotransferase 13
1,17	TBX20	T-box 20
1,17	OR10H5	olfactory receptor, family 10, subfamily H, member 5
1,17	CDH16	cadherin 16, KSP-cadherin
1,17	LRP2BP	LRP2 binding protein
1,17	KBTBD3	kelch repeat and BTB (POZ) domain containing 3
1,17	SRMS	src-related kinase lacking C-terminal regulatory tyrosine and N-terminal myristylation sites

1,17	PLEKHD1	pleckstrin homology domain containing, family D (with coiled-coil domains) member 1
1,17	TP53INP1	tumor protein p53 inducible nuclear protein 1
1,17	IQCF3	IQ motif containing F3
1,17	SOX15	SRY box 15
1,17	KCNK10	potassium channel, two pore domain subfamily K, member 10
1,17	TCP10L	t-complex 10-like
1,17	CSN2	casein beta
1,17	SRY	sex determining region Y
1,17	CRABP1	cellular retinoic acid binding protein 1
1,17	EXOC3	exocyst complex component 3
1,17	WFDC12	WAP four-disulfide core domain 12
1,17	TCEAL5	transcription elongation factor A (SII)-like 5
1,17	OR5H1	olfactory receptor, family 5, subfamily H, member 1
1,17	TGM5	transglutaminase 5
1,17	FCHO1	Memczak2013 ANTISENSE, coding, intronic, upstream_start, UTR5 best transcript NM_001161357
1,17	PTGDR2	prostaglandin D2 receptor 2
1,17	ZPBP	zona pellucida binding protein
1,17	ROBO3	roundabout guidance receptor 3
1,17	VAMP5	vesicle associated membrane protein 5
1,17	PIPOX	pipecolic acid oxidase
1,17	WTIP	Wilms tumor 1 interacting protein
1,17	CSPP1	centrosome and spindle pole associated protein 1
1,17	ORAI1	ORAI calcium release-activated calcium modulator 1
1,17	PRICKLE1	prickle homolog 1
1,17	CKMT2	creatine kinase, mitochondrial 2 (sarcomeric)
1,17	EDA	ectodysplasin A
1,17	BBS1	Bardet-Biedl syndrome 1
1,17	METTL18	methyltransferase like 18
1,17	SAMD9L	sterile alpha motif domain containing 9-like
1,17	ZNF25	zinc finger protein 25
1,17	AC007566.10; GATAD1	Transcript Identified by AceView, Entrez Gene ID(s) 57798; novel transcript, antisense to ERVW-1 and PEX1
1,17	TBK1	TANK-binding kinase 1
1,17	ISYNA1	inositol-3-phosphate synthase 1
1,17	SNTG2	syntrophin, gamma 2
1,17	LINC00612	long intergenic non-protein coding RNA 612
1,17	PPDPF	pancreatic progenitor cell differentiation and proliferation factor
1,17	ARAF	A-Raf proto-oncogene, serine/threonine kinase
1,17	OR10R2	olfactory receptor, family 10, subfamily R, member 2
1,17	TRPV6	transient receptor potential cation channel, subfamily V, member 6
1,17	KRTAP19-1	keratin associated protein 19-1
1,17	FUK	fucokinase
1,17	PPARG	peroxisome proliferator-activated receptor gamma

1,17	USP28	ubiquitin specific peptidase 28
1,17	ERAP2	endoplasmic reticulum aminopeptidase 2
1,17	MARCH3	membrane associated ring finger 3
1,17	BTRC	beta-transducin repeat containing E3 ubiquitin protein ligase
1,17	ANKRD33B	ankyrin repeat domain 33B
1,17	CRYGB	crystallin gamma B
1,17	CCDC70	coiled-coil domain containing 70
1,17	LYN	LYN proto-oncogene, Src family tyrosine kinase
1,17	ZNF546	zinc finger protein 546
1,17	ACTL6B	actin-like 6B
1,17	FBXO17	F-box protein 17
1,17	OR10H1	olfactory receptor, family 10, subfamily H, member 1
1,17	VPREB3	pre-B lymphocyte 3
1,17	MXD4	Memczak2013 ALT_ACCEPTOR, ALT_DONOR, coding, INTERNAL, intronic best transcript NM_006454
1,17	KRTAP10-4	keratin associated protein 10-4
1,17	ZBTB39	zinc finger and BTB domain containing 39
1,17	SLC29A2	solute carrier family 29 (equilibrative nucleoside transporter), member 2
1,17	TMEM144	transmembrane protein 144
1,17	TDRD6	tudor domain containing 6
1,17	C3orf18	chromosome 3 open reading frame 18
1,17	ATXN2L	Transcript Identified by AceView, Entrez Gene ID(s) 11273
1,17	MAP1S	microtubule-associated protein 1S
1,17	RENBP	renin binding protein
1,17	SLMAP	sarcolemma associated protein
1,16	TCEANC2; MIR4781	transcription elongation factor A (SII) N-terminal and central domain containing 2; microRNA 4781
1,16	OR12D2	olfactory receptor, family 12, subfamily D, member 2 (gene/pseudogene)
1,16	NKAP	NFKB activating protein
1,16	DARS2	aspartyl-tRNA synthetase 2, mitochondrial
1,16	CNBD2	cyclic nucleotide binding domain containing 2
1,16	SORBS2	Transcript Identified by AceView, Entrez Gene ID(s) 8470
1,16	ANO3	anoctamin 3
1,16	FAM177A1	family with sequence similarity 177, member A1
1,16	STMN2	stathmin 2
1,16	BCL2L14	BCL2-like 14 (apoptosis facilitator)
1,16	ZNF774	zinc finger protein 774
1,16	ATG4B	autophagy related 4B, cysteine peptidase
1,16	GABRG2	gamma-aminobutyric acid (GABA) A receptor, gamma 2
1,16	KCNK2	potassium channel, two pore domain subfamily K, member 2
1,16	CPNE4	Transcript Identified by AceView, Entrez Gene ID(s) 131034

1,16	LIPM	lipase, family member M
1,16	FBXL21	F-box and leucine-rich repeat protein 21 (gene/pseudogene)
1,16	CELF2	CUGBP, Elav-like family member 2
1,16	RPS6KA1	ribosomal protein S6 kinase, 90kDa, polypeptide 1
1,16	TYW5	tRNA-yW synthesizing protein 5
1,16	PHOSPHO2	phosphatase, orphan 2
1,16	SLC35B3	solute carrier family 35 (adenosine 3-phospho 5-phosphosulfate transporter), member B3
1,16	F11	coagulation factor XI
1,16	GATA6	GATA binding protein 6
1,16	ELOVL1; MIR6734	ELOVL fatty acid elongase 1; microRNA 6734
1,16	DCDC5; DCDC1	doublecortin domain containing 5; doublecortin domain containing 1
1,16	FZR1	Jeck2013 ANTISENSE, coding, INTERNAL, intronic best transcript NM_016263
1,16	AC208162.1	
1,16	CTSS	cathepsin S
1,16	SPATA31A1	SPATA31 subfamily A, member 1
1,16	C21orf91; C21orf91-OT1	chromosome 21 open reading frame 91; C21orf91 overlapping transcript 1
1,16	F8A2	coagulation factor VIII-associated 2
1,16	GNG7	Memczak2013 ALT_ACCEPTOR, ALT_DONOR, coding, INTERNAL, intronic best transcript NM_052847
1,16	HEPN1	hepatocellular carcinoma, down-regulated 1
1,16	MYL1	myosin light chain 1
1,16	CSMD2	CUB and Sushi multiple domains 2
1,16	GSDMB	gasdermin B
1,16	GSTM4	glutathione S-transferase mu 4
1,16	MAN1B1-AS1	MAN1B1 antisense RNA 1 (head to head)
1,16	MMP8	matrix metallopeptidase 8
1,16	C11orf88	chromosome 11 open reading frame 88
1,16	LRRC10	leucine rich repeat containing 10
1,16	SLC16A12	solute carrier family 16, member 12
1,16	EPB41L3	erythrocyte membrane protein band 4.1-like 3
1,16	OR1E1	olfactory receptor, family 1, subfamily E, member 1
1,16	NOL6	nucleolar protein 6 (RNA-associated)
1,16	OR2T27	olfactory receptor, family 2, subfamily T, member 27
1,16	AC008753.6; MYADM	Memczak2013 ANTISENSE, coding, INTERNAL, intronic best transcript NM_001020819; novel transcript, antisense to MYADM
1,16	ZIC1	Zic family member 1
1,16	PRSS23	protease, serine, 23
1,16	GPR62	G protein-coupled receptor 62
1,16	IKBKB	Transcript Identified by AceView, Entrez Gene ID(s) 3551
1,16	DUSP16	dual specificity phosphatase 16
1,16	RBM43	RNA binding motif protein 43

1,16	USP26	ubiquitin specific peptidase 26
1,16	FMO4	flavin containing monooxygenase 4
1,16	sep-04	septin 4
1,16	NLRP3	NLR family, pyrin domain containing 3
1,16	CLPSL1	colipase-like 1
1,16	PRAMEF4	PRAME family member 4
1,16	ISG20L2	interferon stimulated exonuclease gene 20kDa like 2
1,16	TRAPPC4	trafficking protein particle complex 4
1,16	DIO2	deiodinase, iodothyronine, type II
1,16	POTEE	POTE ankyrin domain family, member E
1,16	TMEM169	Transcript Identified by AceView, Entrez Gene ID(s) 92691
1,16	SPPL3	signal peptide peptidase like 3
1,16	TRMT44	tRNA methyltransferase 44 homolog (S. cerevisiae)
1,16	ERVK13-1	endogenous retrovirus group K13, member 1
1,16	DYTN	dystrotelin
1,16	STXBP6	syntaxin binding protein 6 (amisyn)
1,16	LTBP4	Transcript Identified by AceView, Entrez Gene ID(s) 8425
1,16	POTEB	POTE ankyrin domain family, member B
1,16	B3GNT2	UDP-GlcNAc:betaGal beta-1,3-N-acetylglucosaminyltransferase 2
1,16	ASMTL	acetylserotonin O-methyltransferase-like
1,16	ASMTL	acetylserotonin O-methyltransferase-like
1,16	CASZ1	castor zinc finger 1
1,16	EDNRA	endothelin receptor type A
1,16	SELL	selectin L
1,16	TIMP1	TIMP metallopeptidase inhibitor 1
1,16	MAGEB6	MAGE family member B6
1,16	CLCNKA	chloride channel, voltage-sensitive Ka
1,16	ZNF680	zinc finger protein 680
1,16	CCDC155	coiled-coil domain containing 155
1,16	OR8S1	olfactory receptor, family 8, subfamily S, member 1
1,16	TFAP2C	transcription factor AP-2 gamma (activating enhancer binding protein 2 gamma)
1,16	DPT	dermatopontin
1,16	BTBD10	BTB (POZ) domain containing 10
1,16	PPIE	Transcript Identified by AceView, Entrez Gene ID(s) 10450
1,16	DCP1A	decapping mRNA 1A
1,16	STX17	syntaxin 17
1,16	OR4L1	olfactory receptor, family 4, subfamily L, member 1
1,16	CHRM2	cholinergic receptor, muscarinic 2
1,16	ZNF705G	zinc finger protein 705G
1,16	HOMEZ	homeobox and leucine zipper encoding
1,16	FAM153A	family with sequence similarity 153, member A
1,16	USP43	ubiquitin specific peptidase 43
1,16	DTNA	dystrobrevin, alpha
1,16	KRT74	keratin 74, type II
1,16	CH25H	cholesterol 25-hydroxylase

1,16	ST6GALNAC3	Memczak2013 ANTISENSE, coding, INTERNAL, intronic best transcript NM_152996
1,16	ZBTB3	zinc finger and BTB domain containing 3
1,16	SH2D3A	SH2 domain containing 3A
1,16	RTP1	receptor (chemosensory) transporter protein 1
1,16	BMF	Bcl2 modifying factor
1,16	KANK4	KN motif and ankyrin repeat domains 4
1,16	ANK2	Transcript Identified by AceView, Entrez Gene ID(s) 287
1,16	MEF2C	myocyte enhancer factor 2C
1,16	BCHE	butyrylcholinesterase
1,16	C19orf25	chromosome 19 open reading frame 25
1,16	KHDRBS2	KH domain containing, RNA binding, signal transduction associated 2
1,16	GCKR	Transcript Identified by AceView, Entrez Gene ID(s) 2646
1,16	SYNE1	spectrin repeat containing, nuclear envelope 1
1,16	MDM1	Mdm1 nuclear protein
1,16	SLC12A1	solute carrier family 12 (sodium/potassium/chloride transporter), member 1
1,16	C6	complement component 6
1,16	EIF4E3	eukaryotic translation initiation factor 4E family member 3
1,16	FAM231D; LINC00869	family with sequence similarity 231, member D; long intergenic non-protein coding RNA 869
1,16	ANKRD19P	ankyrin repeat domain 19, pseudogene
1,16	HS6ST2	heparan sulfate 6-O-sulfotransferase 2
1,16	ZNF830	zinc finger protein 830
1,16	ZNF479	zinc finger protein 479
1,16	MT1A	metallothionein 1A
1,16	VGF	VGF nerve growth factor inducible
1,16	RP11-9L18.3; FAM78B	Transcript Identified by AceView, Entrez Gene ID(s) 149297; novel transcript
1,16	C1orf185	chromosome 1 open reading frame 185
1,16	NDUFA10	NADH dehydrogenase (ubiquinone) 1 alpha subcomplex, 10, 42kDa
1,16	LRRIQ4	leucine-rich repeats and IQ motif containing 4
1,16	CDA	cytidine deaminase
1,16	C21orf62	chromosome 21 open reading frame 62
1,16	SULF2	Memczak2013 ANTISENSE, coding, INTERNAL, intronic best transcript NM_001161841
1,16	RP11-281A20.2; TLR4	novel transcript; Transcript Identified by AceView, Entrez Gene ID(s) 7099
1,16	GPR39	G protein-coupled receptor 39
1,16	CLIC3	chloride intracellular channel 3
1,16	C4orf36	chromosome 4 open reading frame 36
1,16	FAM78B	family with sequence similarity 78, member B
1,16	FMN1	formin 1
1,16	TRDMT1	tRNA aspartic acid methyltransferase 1
1,16	VEGFC	vascular endothelial growth factor C

1,16	MEX3A	mex-3 RNA binding family member A
1,16	PIP4K2A	phosphatidylinositol-5-phosphate 4-kinase, type II, alpha
1,16	C15orf65	chromosome 15 open reading frame 65
1,16	AAAS	achalasia, adrenocortical insufficiency, alacrimia
1,16	OMA1	OMA1 zinc metallopeptidase
1,16	IQCF2	IQ motif containing F2
1,16	LCN8	lipocalin 8
1,16	MBLAC1	metallo-beta-lactamase domain containing 1
1,16	CCHCR1	coiled-coil alpha-helical rod protein 1
1,16	GUCA1A	guanylate cyclase activator 1A (retina)
1,16	LMAN2L	lectin, mannose-binding 2-like
1,16	SPATC1	spermatogenesis and centriole associated 1
1,16	TNFSF12	tumor necrosis factor (ligand) superfamily, member 12
1,16	NANOG	Nanog homeobox
1,16	ATN1	atrophin 1
1,16	TGFBR3	transforming growth factor beta receptor III
1,16	LYAR	Ly1 antibody reactive
1,16	ORAOV1	oral cancer overexpressed 1
1,16	PHYHD1	phytanoyl-CoA dioxygenase domain containing 1
1,16	PLAC8L1	PLAC8-like 1
1,16	KCNH8	potassium channel, voltage gated eag related subfamily H, member 8
1,16	NEK5	NIMA-related kinase 5
1,16	C5orf34	chromosome 5 open reading frame 34
1,16	RFNG	RFNG O-fucosylpeptide 3-beta-N-acetylglucosaminyltransferase
1,16	TSPAN10	tetraspanin 10
1,16	PRELID2	PRELI domain containing 2
1,16	ANGPTL5	angiopoietin like 5
1,16	CRACR2A	calcium release activated channel regulator 2A
1,16	FAM133B	Transcript Identified by AceView, Entrez Gene ID(s) 257415
1,16	RBMXL2	RNA binding motif protein, X-linked-like 2
1,16	PTPRZ1	protein tyrosine phosphatase, receptor-type, Z polypeptide 1
1,16	MSANTD3-TMEFF1	MSANTD3-TMEFF1 readthrough
1,16	ARHGEF10	Jeck2013 ALT_ACCEPTOR, ALT_DONOR, coding, INTERNAL, intronic best transcript NM_014629
1,16	SAG	S-antigen; retina and pineal gland (arrestin)
1,16	DNTT	DNA nucleotidylexotransferase
1,16	CALN1	calneuron 1
1,16	WRN	Transcript Identified by AceView, Entrez Gene ID(s) 7486
1,16	URGCP	Salzman2013 ANNOTATED, coding, OVEXON, UTR5 best transcript NM_001077664
1,16	SLC22A16	solute carrier family 22 (organic cation/carnitine transporter), member 16

1,16	SPRY3	sprouty RTK signaling antagonist 3
1,16	SPRY3	Homo sapiens sprouty RTK signaling antagonist 3 (SPRY3), transcript variant 1, mRNA.; sprouty homolog 3 (Drosophila) [Source:HGNC Symbol;Acc:HGNC:11271]; Homo sapiens sprouty homolog 3 (Drosophila), mRNA (cDNA clone MGC:97072 IMAGE:7262284), complete cds.; Homo sapiens sprouty homolog 3 (Drosophila), mRNA (cDNA clone MGC:103904 IMAGE:30915293), complete cds.; Homo sapiens sprouty homolog 3 (Drosophila), mRNA (cDNA clone MGC:104063 IMAGE:30915511), complete cds.
1,16	FAM69A	family with sequence similarity 69, member A
1,16	NCAM1	Transcript Identified by AceView, Entrez Gene ID(s) 4684
1,16	MYO1D	myosin ID
1,16	FABP2	fatty acid binding protein 2, intestinal
1,16	PPT2; EGFL8; PPT2-EGFL8	palmitoyl-protein thioesterase 2; EGF-like-domain, multiple 8; PPT2-EGFL8 readthrough (NMD candidate)
1,16	EXOC4	exocyst complex component 4
1,16	MSANTD3	Myb/SANT-like DNA-binding domain containing 3
1,16	SLC15A5	solute carrier family 15, member 5
1,16	buskee; FAM25D; FAM25E; zawskaw	Transcript Identified by AceView; family with sequence similarity 25, member D [Source:HGNC Symbol;Acc:23588]; family with sequence similarity 25, member E [Source:HGNC Symbol;Acc:HGNC:23587]
1,16	AMHR2	anti-Mullerian hormone receptor, type II
1,16	CLDN14	claudin 14
1,16	NCF2	neutrophil cytosolic factor 2
1,16	OR5F1	olfactory receptor, family 5, subfamily F, member 1
1,16	ASB16-AS1	ASB16 antisense RNA 1
1,16	UVRAG	Memczak2013 ALT_ACCEPTOR, ALT_DONOR, coding, INTERNAL, intronic best transcript NM_003369
1,16	ALKBH8	alkB homolog 8, tRNA methyltransferase
1,16	TAS2R30; TAS2R43; PRR4	taste receptor, type 2, member 30; taste receptor, type 2, member 43; proline rich 4 (lacrimal)
1,15	LPAR3	lysophosphatidic acid receptor 3
1,15	ZNF415	zinc finger protein 415
1,15	PARD6B	par-6 family cell polarity regulator beta
1,15	SELV	selenoprotein V; selenoprotein V [Source:EntrezGene;Acc:348303]; Transcript Identified by AceView, Entrez Gene ID(s) 348303, RefSeq ID(s) NM_182704
1,15	BORA	bora, aurora kinase A activator
1,15	MYOM3	myomesin 3
1,15	LRP6	LDL receptor related protein 6
1,15	CYB5D1	cytochrome b5 domain containing 1

1,15	FBXO36	Transcript Identified by AceView, Entrez Gene ID(s) 130888
1,15	VSTM1	V-set and transmembrane domain containing 1
1,15	NLN	neurolysin (metallopeptidase M3 family)
1,15	NEU4	sialidase 4
1,15	CYP2D6	cytochrome P450, family 2, subfamily D, polypeptide 6
1,15	CD164L2	CD164 sialomucin-like 2
1,15	CLIC2	chloride intracellular channel 2
1,15	CNPY1	canopy FGF signaling regulator 1
1,15	CAMK2N2	calcium/calmodulin-dependent protein kinase II inhibitor 2
1,15	ANPEP	alanyl (membrane) aminopeptidase
1,15	TRIM51	tripartite motif-containing 51
1,15	NDUFA10	Memczak2013 ANTISENSE, coding, INTERNAL, intronic best transcript NM_004544
1,15	CSTL1	cystatin-like 1
1,15	KRT83	keratin 83, type II
1,15	ELOVL7	ELOVL fatty acid elongase 7
1,15	FEV	FEV (ETS oncogene family)
1,15	FAM47C	family with sequence similarity 47, member C
1,15	RASSF1	Ras association (RalGDS/AF-6) domain family member 1
1,15	SPOCD1	SPOC domain containing 1
1,15	L3MBTL2	l(3)mbt-like 2 (Drosophila)
1,15	VPS52	vacuolar protein sorting 52 homolog (S. cerevisiae)
1,15	ZNF514	zinc finger protein 514
1,15	USP51	ubiquitin specific peptidase 51
1,15	OR1N2	olfactory receptor, family 1, subfamily N, member 2
1,15	ABCG8	ATP binding cassette subfamily G member 8
1,15	SEMG1	semenogelin I
1,15	SKI; kerlabu	Memczak2013 ALT_ACCEPTOR, ALT_DONOR, coding, INTERNAL, intronic best transcript NM_003036; Transcript Identified by AceView
1,15	WFDC2	WAP four-disulfide core domain 2
1,15	CTTNBP2	cortactin binding protein 2
1,15	ANKDD1A	ankyrin repeat and death domain containing 1A
1,15	KRT2	keratin 2, type II
1,15	FBXO44	F-box protein 44
1,15	TNXA	tenascin XA (pseudogene)
1,15	PSKH2	protein serine kinase H2
1,15	HMBS	hydroxymethylbilane synthase
1,15	POTEG; POTEM	POTE ankyrin domain family, member G; POTE ankyrin domain family, member M
1,15	KLK7	kallikrein related peptidase 7
1,15	PIAS3	protein inhibitor of activated STAT 3
1,15	HEPACAM2	HEPACAM family member 2
1,15	RPH3A	rabphilin 3A
1,15	KITLG	KIT ligand
1,15	CST9	cystatin 9 (testatin)

1,15	SKA3	spindle and kinetochore associated complex subunit 3
1,15	TMEM42	transmembrane protein 42
1,15	INTS9	integrator complex subunit 9
1,15	FNDC1	fibronectin type III domain containing 1
1,15	XKRY2; XKRY	X-linked Kx blood group related, Y-linked 2; X-linked Kx blood group related, Y-linked
1,15	CELA3B	chymotrypsin-like elastase family, member 3B
1,15	ZNF852	zinc finger protein 852
1,15	JRKL	JRK-like
1,15	RPSAP58; RPSA	ribosomal protein SA pseudogene 58; ribosomal protein SA
1,15	ZNF805	zinc finger protein 805
1,15	ATP1B2	ATPase, Na+/K+ transporting, beta 2 polypeptide
1,15	PRAMEF5	PRAME family member 5
1,15	MAOB	monoamine oxidase B
1,15	PZP	pregnancy-zone protein
1,15	DPP4	dipeptidyl-peptidase 4
1,15	FOXD4L3	forkhead box D4-like 3
1,15	CSRNP3	cysteine-serine-rich nuclear protein 3
1,15	FABP5	fatty acid binding protein 5 (psoriasis-associated)
1,15	TEX38	testis expressed 38
1,15	NT5C1A	5-nucleotidase, cytosolic IA
1,15	LINC01089	long intergenic non-protein coding RNA 1089
1,15	MICALCL	MICAL C-terminal like
1,15	MANBA	mannosidase, beta A, lysosomal
1,15	FCER2	Fc fragment of IgE, low affinity II, receptor for (CD23)
1,15	ADRA2B	adrenoceptor alpha 2B
1,15	ABCC2	ATP binding cassette subfamily C member 2
1,15	BDKRB1	bradykinin receptor B1
1,15	PLCH2	phospholipase C, eta 2
1,15	OLAH	oleoyl-ACP hydrolase
1,15	C4orf51	chromosome 4 open reading frame 51
1,15	ND2	MTND2
1,15	CASQ2	calsequestrin 2 (cardiac muscle)
1,15	MUC5AC	mucin 5AC, oligomeric mucus/gel-forming
1,15	JAK3	Janus kinase 3
1,15	PROSER1	proline and serine rich 1
1,15	ACAN	aggrecan
1,15	NCALD	neurocalcin delta
1,15	AIM1	absent in melanoma 1
1,15	SASS6	SAS-6 centriolar assembly protein
1,15	GUF1	GUF1 homolog, GTPase
1,15	BCL2L14	BCL2-like 14 (apoptosis facilitator)
1,15	PDC	phosducin
1,15	MYH9	Jeck2013 ANTISENSE, CDS, coding, INTERNAL, intronic, OVCODE, OVEXON best transcript NM_002473
1,15	C10orf107	chromosome 10 open reading frame 107
1,15	KLHL23	kelch-like family member 23

1,15	SGF29	SAGA complex associated factor 29
1,15	ZNF780B	zinc finger protein 780B
1,15	YARS2	tyrosyl-tRNA synthetase 2, mitochondrial
1,15	FAM153B	family with sequence similarity 153, member B
1,15	TEX33	testis expressed 33
1,15	SIPA1	signal-induced proliferation-associated 1
1,15	GDPGP1	GDP-D-glucose phosphorylase 1
1,15	SLC11A2	solute carrier family 11 (proton-coupled divalent metal ion transporter), member 2
1,15	TMEM185A	transmembrane protein 185A
1,15	KCNB2	potassium channel, voltage gated Shab related subfamily B, member 2
1,15	ZNF764	zinc finger protein 764
1,15	B3GNT4	UDP-GlcNAc:betaGal beta-1,3-N-acetylglucosaminyltransferase 4
1,15	HIVEP1	human immunodeficiency virus type I enhancer binding protein 1
1,15	SHOX	short stature homeobox
1,15	FSD2	fibronectin type III and SPRY domain containing 2
1,15	USF1	upstream transcription factor 1
1,15	CCER2	coiled-coil glutamate rich protein 2
1,15	CRYGC	crystallin gamma C
1,15	NRP1	Transcript Identified by AceView, Entrez Gene ID(s) 8829
1,15	TEAD3	TEA domain family member 3
1,15	SPATA33	spermatogenesis associated 33
1,15	PTGS1	prostaglandin-endoperoxide synthase 1 (prostaglandin G/H synthase and cyclooxygenase)
1,15	SLC6A3	solute carrier family 6 (neurotransmitter transporter), member 3
1,15	S100A7L2	S100 calcium binding protein A7 like 2
1,15	CROCC2	ciliary rootlet coiled-coil, rootletin family member 2 [Source:HGNC Symbol;Acc:HGNC:51677]
1,15	SPRYD4	SPRY domain containing 4
1,15	C2orf78	chromosome 2 open reading frame 78
1,15	HIP1R	Memczak2013 ALT_ACCEPTOR, ALT_DONOR, coding, INTERNAL, intronic best transcript NM_003959
1,15	SFTPC	surfactant protein C
1,15	CEP164	centrosomal protein 164kDa
1,15	KIAA0125	KIAA0125
1,15	RP11-466A19.5; MYO1D	Transcript Identified by AceView, Entrez Gene ID(s) 4642; novel transcript, sense intronic MYO1D
1,15	VPS8	vacuolar protein sorting 8 homolog (S. cerevisiae)
1,15	NEUROG2	neurogenin 2
1,15	PTPRE	protein tyrosine phosphatase, receptor type, E
1,15	MAP4K1	mitogen-activated protein kinase kinase kinase kinase 1
1,15	DPP9-AS1	DPP9 antisense RNA 1

1,15	GS1-259H13.2; TCONS_l2_00026125; TCONS_l2_00026126; ZNF655	transmembrane protein 225-like; Jeck2013 ALT_ACCEPTOR, ALT_DONOR, ncRNA, OVCODE, OVEXON, upstream_start best transcript TCONS_l2_00026125; Salzman2013 ANNOTATED, INTERNAL, ncRNA, OVCODE, OVEXON best transcript TCONS_l2_00026126; novel transcript; Transcript Identified by AceView, Entrez Gene ID(s) 79027
1,15	SLITRK5	SLIT and NTRK-like family, member 5
1,15	TP73-AS1	TP73 antisense RNA 1
1,15	MTRNR2L4	MT-RNR2-like 4
1,15	DIO3	deiodinase, iodothyronine, type III
1,15	PMS2	PMS1 homolog 2, mismatch repair system component
1,15	ADGRA3	adhesion G protein-coupled receptor A3
1,15	CD244	CD244 molecule, natural killer cell receptor 2B4
1,15	TMEM184C	transmembrane protein 184C
1,15	TOE1	target of EGR1, member 1 (nuclear)
1,15	TBX6	T-box 6
1,15	GNA15	guanine nucleotide binding protein (G protein), alpha 15 (Gq class)
1,15	SLC45A3	solute carrier family 45, member 3
1,15	RGS16	regulator of G-protein signaling 16
1,15	MLXIPL	MLX interacting protein-like
1,15	SLC5A4	solute carrier family 5 (glucose activated ion channel), member 4
1,15	PPP2R2B	protein phosphatase 2, regulatory subunit B, beta
1,15	CFHR1	complement factor H-related 1
1,15	SLC13A2	solute carrier family 13 (sodium-dependent dicarboxylate transporter), member 2
1,15	RFPL4A	ret finger protein-like 4A
1,15	PPP6R1	Memczak2013 ANTISENSE, CDS, coding, INTERNAL best transcript NM_014931
1,15	C1orf94	chromosome 1 open reading frame 94
1,15	CHKA	choline kinase alpha
1,15	SRRD	SRR1 domain containing
1,15	GPR17	G protein-coupled receptor 17
1,15	SDR16C5	short chain dehydrogenase/reductase family 16C, member 5
1,15	FOXD4L5	forkhead box D4-like 5
1,15	CASP4	caspase 4
1,15	GBA2	glucosidase, beta (bile acid) 2
1,15	CCDC3	coiled-coil domain containing 3
1,15	FBXL12	F-box and leucine-rich repeat protein 12
1,15	CYGB; RP11-666A8.8	Transcript Identified by AceView, Entrez Gene ID(s) 114757; novel transcript, antisense to PRCD
1,15	FAM127C	family with sequence similarity 127, member C
1,15	BBS12	Bardet-Biedl syndrome 12
1,15	DNAH11	dynein, axonemal, heavy chain 11
1,15	ZNF100	zinc finger protein 100
1,15	HSD17B7	hydroxysteroid (17-beta) dehydrogenase 7

1,15	KCNMA1	potassium channel, calcium activated large conductance subfamily M alpha, member 1
1,15	C19orf52	chromosome 19 open reading frame 52
1,15	ATP6V0A1; MIR5010	ATPase, H+ transporting, lysosomal V0 subunit a1; microRNA 5010
1,15	CTAGE8; CTAGE4	CTAGE family, member 8; CTAGE family, member 4
1,15	CCDC129	coiled-coil domain containing 129
1,15	ABCG5	ATP binding cassette subfamily G member 5
1,15	FAM105A	family with sequence similarity 105, member A
1,15	NTN5	netrin 5
1,15	CT62	cancer/testis antigen 62
1,15	PATE4	prostate and testis expressed 4
1,15	DNAH10OS	dynein, axonemal, heavy chain 10 opposite strand
1,15	CD226	CD226 molecule
1,15	PCDH8	protocadherin 8
1,15	MTFR1	mitochondrial fission regulator 1
1,15	ATG16L1	autophagy related 16-like 1
1,15	PIGM	phosphatidylinositol glycan anchor biosynthesis class M
1,15	EQTN	equatorin, sperm acrosome associated
1,15	IFNA7	interferon, alpha 7
1,15	POU2F2	POU class 2 homeobox 2
1,15	PKNOX1	PBX/knotted 1 homeobox 1
1,15	TIGD3	tigger transposable element derived 3
1,15	PRICKLE3	prickle homolog 3
1,15	HELZ2	helicase with zinc finger 2, transcriptional coactivator
1,15	C9orf3; MIR24-1; MIR23B; MIR27B	chromosome 9 open reading frame 3; microRNA 24-1; microRNA 23b; microRNA 27b
1,15	ASB7	ankyrin repeat and SOCS box containing 7
1,15	SLIT2	slit guidance ligand 2
1,15	RRS1	ribosome biogenesis regulator homolog
1,15	RAB11FIP4; MIR4724	RAB11 family interacting protein 4 (class II); microRNA 4724
1,15	RPS6KA1	ribosomal protein S6 kinase, 90kDa, polypeptide 1
1,15	PCDHB6	protocadherin beta 6
1,15	LBX1	ladybird homeobox 1
1,15	KAZALD1	Kazal-type serine peptidase inhibitor domain 1
1,15	BEAN1	brain expressed, associated with NEDD4, 1
1,15	SMIM5	small integral membrane protein 5
1,15	PTGFR	prostaglandin F receptor (FP)
1,15	LHX3	LIM homeobox 3
1,15	ATP8B3	ATPase, aminophospholipid transporter, class I, type 8B, member 3
1,15	TMEM202	transmembrane protein 202
1,15	TEX13A	testis expressed 13A
1,15	TMEM244	transmembrane protein 244

1,15	EXOC7	Zhang2013 ALT_ACCEPTOR, ALT_DONOR, coding, INTERNAL, intronic best transcript NM_001145297
1,15	PKMYT1	protein kinase, membrane associated tyrosine/threonine 1
1,15	C10orf54	chromosome 10 open reading frame 54
1,15	KIAA1107	KIAA1107
1,15	NDUFAF6	NADH dehydrogenase (ubiquinone) complex I, assembly factor 6
1,15	PHLDA2	pleckstrin homology-like domain, family A, member 2
1,15	EDARADD	EDAR-associated death domain
1,15	ARHGEF1	Rho guanine nucleotide exchange factor 1
1,15	S100B	S100 calcium binding protein B
1,15	DOCK8	dedicator of cytokinesis 8
1,14	ATG16L1	autophagy related 16-like 1
1,14	SLC6A5	solute carrier family 6 (neurotransmitter transporter), member 5
1,14	CFHR5	complement factor H-related 5
1,14	DNM2	Transcript Identified by AceView, Entrez Gene ID(s) 1785
1,14	PSMC4	proteasome 26S subunit, ATPase 4 [Source:HGNC Symbol;Acc:HGNC:9551]
1,14	HNRNPCL4	heterogeneous nuclear ribonucleoprotein C-like 4
1,14	LRIG1	
1,14	TEKT1	tektin 1
1,14	CRHR2	corticotropin releasing hormone receptor 2
1,14	IL17RE	interleukin 17 receptor E
1,14	CLDN16	claudin 16
1,14	SPANXC; SPANXD	SPANX family, member C; SPANX family, member D
1,14	CXorf65	chromosome X open reading frame 65
1,14	OCM	oncomodulin
1,14	PSMA3-AS1	PSMA3 antisense RNA 1
1,14	PDE6A	phosphodiesterase 6A, cGMP-specific, rod, alpha
1,14	SERPINB11	serpin peptidase inhibitor, clade B (ovalbumin), member 11 (gene/pseudogene)
1,14	EFCAB10	EF-hand calcium binding domain 10
1,14	BTLA	B and T lymphocyte associated
1,14	OMD	osteomodulin
1,14	GAST	gastrin
1,14	KANK1	KN motif and ankyrin repeat domains 1
1,14	RPS6KA5	ribosomal protein S6 kinase, 90kDa, polypeptide 5
1,14	NRGN	neurogranin (protein kinase C substrate, RC3)
1,14	GTPBP10	GTP-binding protein 10 (putative)
1,14	ACACA	Transcript Identified by AceView, Entrez Gene ID(s) 31
1,14	OR52K2	olfactory receptor, family 52, subfamily K, member 2
1,14	UPK1A	uroplakin 1A
1,14	SYNE2	spectrin repeat containing, nuclear envelope 2

1,14	ABAT	4-aminobutyrate aminotransferase
1,14	ANGPTL4	angiopoietin like 4
1,14	GDF6	growth differentiation factor 6
1,14	HMBS	hydroxymethylbilane synthase
1,14	PXMP4	peroxisomal membrane protein 4
1,14	SERPINE1	serpin peptidase inhibitor, clade E (nexin, plasminogen activator inhibitor type 1), member 1
1,14	QRICH2	glutamine rich 2
1,14	MED14OS	MED14 opposite strand
1,14	UGT2B28	UDP glucuronosyltransferase 2 family, polypeptide B28
1,14	LINC01342	long intergenic non-protein coding RNA 1342
1,14	CNIH3	cornichon family AMPA receptor auxiliary protein 3
1,14	PDE7A	phosphodiesterase 7A
1,14	DYDC1	DPY30 domain containing 1
1,14	KRTAP9-8	keratin associated protein 9-8
1,14	NRBF2	nuclear receptor binding factor 2
1,14	ZC3H10	zinc finger CCCH-type containing 10
1,14	IFNA17	interferon, alpha 17
1,14	PTPRJ	protein tyrosine phosphatase, receptor type, J
1,14	ENTPD1	ectonucleoside triphosphate diphosphohydrolase 1
1,14	TMEM170A	transmembrane protein 170A
1,14	DAGLB	diacylglycerol lipase, beta
1,14	E2F5	E2F transcription factor 5, p130-binding
1,14	IL12RB1	interleukin 12 receptor, beta 1
1,14	PRAMEF9	
1,14	NEUROD2	neuronal differentiation 2
1,14	VAX1	ventral anterior homeobox 1
1,14	SGPP1	sphingosine-1-phosphate phosphatase 1
1,14	HDHD3	haloacid dehalogenase-like hydrolase domain containing 3
1,14	SGMS2	sphingomyelin synthase 2
1,14	SLCO4C1	solute carrier organic anion transporter family, member 4C1
1,14	ALG5	ALG5, dolichyl-phosphate beta-glucosyltransferase
1,14	NOP2	NOP2 nucleolar protein
1,14	DEFB118	defensin, beta 118
1,14	EHF	ets homologous factor
1,14	SCN5A	sodium channel, voltage gated, type V alpha subunit
1,14	SIX5	SIX homeobox 5
1,14	GPR50	G protein-coupled receptor 50
1,14	MYOZ1	myozenin 1
1,14	CCDC102B	coiled-coil domain containing 102B
1,14	AMIGO2	adhesion molecule with Ig-like domain 2
1,14	REEP2	receptor accessory protein 2
1,14	NOVA2	neuro-oncological ventral antigen 2
1,14	PNPLA7	Transcript Identified by AceView, Entrez Gene ID(s) 375775
1,14	PLTP	phospholipid transfer protein

1,14	SLC39A10	Transcript Identified by AceView, Entrez Gene ID(s) 57181
1,14	VPS11	VPS11, CORVET/HOPS core subunit [Source:HGNC Symbol;Acc:HGNC:14583]
1,14	OLFML1	olfactomedin like 1
1,14	KCNAB1	Transcript Identified by AceView, Entrez Gene ID(s) 7881
1,14	CLK3	CDC like kinase 3
1,14	CLDN15	claudin 15
1,14	MOSPD1	motile sperm domain containing 1
1,14	NOCT	nocturnin
1,14	RDH10	retinol dehydrogenase 10 (all-trans)
1,14	FBXO33	F-box protein 33
1,14	GADL1	glutamate decarboxylase like 1
1,14	SYCP2	synaptonemal complex protein 2
1,14	METTL17	methyltransferase like 17
1,14	OR11L1	olfactory receptor, family 11, subfamily L, member 1
1,14	CA11	carbonic anhydrase XI
1,14	RAD9B	RAD9 checkpoint clamp component B
1,14	ZNF83	zinc finger protein 83
1,14	IFNA6	interferon, alpha 6
1,14	NFE2L3	nuclear factor, erythroid 2-like 3
1,14	CFAP46	cilia and flagella associated protein 46
1,14	ATP11A	Transcript Identified by AceView, Entrez Gene ID(s) 23250
1,14	HSD11B1	hydroxysteroid (11-beta) dehydrogenase 1
1,14	ZHX2	zinc fingers and homeoboxes 2
1,14	OR2L2	olfactory receptor, family 2, subfamily L, member 2
1,14	CYP27A1	cytochrome P450, family 27, subfamily A, polypeptide 1
1,14	MSI1	musashi RNA binding protein 1
1,14	ERBB2	erb-b2 receptor tyrosine kinase 2
1,14	NTNG1	netrin G1
1,14	MLLT10	myeloid/lymphoid or mixed-lineage leukemia; translocated to, 10
1,14	SLC7A13	solute carrier family 7 (anionic amino acid transporter), member 13
1,14	PNLIP	pancreatic lipase
1,14	TIGD7	tigger transposable element derived 7
1,14	AGXT	alanine-glyoxylate aminotransferase
1,14	GJB4	gap junction protein beta 4
1,14	KLC3	kinesin light chain 3
1,14	PMP2	peripheral myelin protein 2
1,14	PIGW	phosphatidylinositol glycan anchor biosynthesis class W
1,14	CTAGE15	CTAGE family, member 15
1,14	GLIS1	GLIS family zinc finger 1
1,14	DUSP2	dual specificity phosphatase 2
1,14	UBD	ubiquitin D
1,14	MTRNR2L2	MT-RNR2-like 2

1,14	BCL2L14	BCL2-like 14 (apoptosis facilitator)
1,14	TNFRSF9	tumor necrosis factor receptor superfamily, member 9
1,14	SSX1	synovial sarcoma, X breakpoint 1
1,14	YIF1A	Yip1 interacting factor homolog A (S. cerevisiae)
1,14	SEMG2	semenogelin II
1,14	TBC1D9	Memczak2013 ALT_ACCEPTOR, ALT_DONOR, coding, INTERNAL, intronic best transcript NM_015130
1,14	JRK	Jrk helix-turn-helix protein
1,14	SNX18	sorting nexin 18
1,14	ALPP	alkaline phosphatase, placental
1,14	DOPEY2	dopey family member 2
1,14	POTEH	POTE ankyrin domain family, member H
1,14	PABPC3	poly(A) binding protein, cytoplasmic 3
1,14	PALM3	paralemmin 3
1,14	ANKRD7	ankyrin repeat domain 7
1,14	SLC27A2	solute carrier family 27 (fatty acid transporter), member 2
1,14	ZNF35	zinc finger protein 35
1,14	GLIPR1L2	GLI pathogenesis-related 1 like 2
1,14	FHAD1	forkhead-associated (FHA) phosphopeptide binding domain 1
1,14	RNF38	ring finger protein 38
1,14	PCYT2	phosphate cytidylyltransferase 2, ethanolamine
1,14	PIAS1	protein inhibitor of activated STAT 1
1,14	USP17L1	ubiquitin specific peptidase 17-like family member 1
1,14	KRT84	keratin 84, type II
1,14	GIMAP8	GTPase, IMAP family member 8
1,14	ABCA10	ATP binding cassette subfamily A member 10
1,14	CDC42SE2	CDC42 small effector 2
1,14	BOD1L2	biorientation of chromosomes in cell division 1-like 2
1,14	C12orf74	chromosome 12 open reading frame 74
1,14	CCDC69	coiled-coil domain containing 69
1,14	KRTAP5-3	keratin associated protein 5-3
1,14	NFS1	NFS1 cysteine desulfurase
1,14	C2orf88	chromosome 2 open reading frame 88
1,14	PIK3CG	phosphatidylinositol-4,5-bisphosphate 3-kinase, catalytic subunit gamma
1,14	ISX	intestine-specific homeobox
1,14	TMEM136	transmembrane protein 136
1,14	PPP1R10	protein phosphatase 1, regulatory subunit 10
1,14	CAMK1D	Memczak2013 ALT_ACCEPTOR, ALT_DONOR, coding, INTERNAL, intronic best transcript NM_020397
1,14	SNAI1	snail family zinc finger 1
1,14	VWA2	von Willebrand factor A domain containing 2
1,14	DNAJC30	DnaJ (Hsp40) homolog, subfamily C, member 30
1,14	AMPD3	adenosine monophosphate deaminase 3
1,14	SULT1A4; SULT1A3	sulfotransferase family 1A member 4; sulfotransferase family 1A member 3

1,14	MSH4	mutS homolog 4
1,14	TSPAN19	tetraspanin 19
1,14	TRIM77	tripartite motif containing 77
1,14	NRM	nurim (nuclear envelope membrane protein)
1,14	HMBS	hydroxymethylbilane synthase
1,14	C12orf10	chromosome 12 open reading frame 10
1,14	OSTN	osteocrin
1,14	FAM49A	family with sequence similarity 49, member A
1,14	CDHR1	cadherin-related family member 1
1,14	PKP4	Transcript Identified by AceView, Entrez Gene ID(s) 8502
1,14	ZNF646	zinc finger protein 646
1,14	LOC101928694; RP11-355N15.1	uncharacterized LOC101928694; novel transcript, antisense to LIPC
1,14	ACTN3	actinin, alpha 3 (gene/pseudogene)
1,14	RP11-93O14.2; VPS35	novel transcript; Transcript Identified by AceView, Entrez Gene ID(s) 55737
1,14	ZNF844	zinc finger protein 844
1,14	OR6V1	olfactory receptor, family 6, subfamily V, member 1
1,14	AREG	amphiregulin
1,14	IER3	immediate early response 3
1,14	USB1	U6 snRNA biogenesis 1
1,14	REXO1; MIR1909	REX1, RNA exonuclease 1 homolog; microRNA 1909
1,14	MTHFSD	methenyltetrahydrofolate synthetase domain containing
1,14	SPDYE3	speedy/RINGO cell cycle regulator family member E3
1,14	MLXIP	MLX interacting protein
1,14	HDLBP	Transcript Identified by AceView, Entrez Gene ID(s) 3069
1,14	LRRC8D	leucine rich repeat containing 8 family, member D
1,14	LY6G6C	lymphocyte antigen 6 complex, locus G6C
1,14	MYBPC3	myosin binding protein C, cardiac
1,14	PGLYRP3	peptidoglycan recognition protein 3
1,14	SHD	Src homology 2 domain containing transforming protein D
1,14	PKD1L2	polycystic kidney disease 1-like 2 (gene/pseudogene)
1,14	ZSCAN18	zinc finger and SCAN domain containing 18
1,14	FAXC	failed axon connections homolog
1,14	HHAT	hedgehog acyltransferase
1,14	PHACTR3	phosphatase and actin regulator 3
1,14	EFCAB7; DLEU2L	EF-hand calcium binding domain 7; deleted in lymphocytic leukemia 2-like
1,14	CDK14	cyclin-dependent kinase 14
1,14	H2AFX	H2A histone family, member X
1,14	ZNF563	zinc finger protein 563
1,14	OR51B5	olfactory receptor, family 51, subfamily B, member 5
1,14	PRR34	proline rich 34
1,14	KLF7	Kruppel-like factor 7 (ubiquitous)

1,14	GDF7	growth differentiation factor 7
1,14	ANKMY1	ankyrin repeat and MYND domain containing 1
1,14	C12orf50	chromosome 12 open reading frame 50
1,14	TGM4	transglutaminase 4
1,14	PRKCG	protein kinase C, gamma
1,14	PPP4R4	protein phosphatase 4, regulatory subunit 4
1,14	RBM41	RNA binding motif protein 41
1,14	ZCWPW2	zinc finger, CW type with PWWP domain 2
1,14	IGLON5	IgLON family member 5
1,14	IL36B	interleukin 36, beta
1,14	SQLE	squalene epoxidase
1,14	KRTAP11-1	keratin associated protein 11-1
1,14	OPN3	opsin 3
1,14	GRK4	G protein-coupled receptor kinase 4
1,14	OR51F2	olfactory receptor, family 51, subfamily F, member 2
1,14	AMOTL1	Memczak2013 ANTISENSE, coding, INTERNAL, UTR3 best transcript NM_130847
1,14	NMT2	N-myristoyltransferase 2
1,14	IL1RN	interleukin 1 receptor antagonist
1,14	CCDC88C	coiled-coil domain containing 88C
1,14	GIP	gastric inhibitory polypeptide
1,14	CDH19	cadherin 19, type 2
1,14	REG1A	regenerating islet-derived 1 alpha
1,14	ADAMTS17	ADAM metallopeptidase with thrombospondin type 1 motif 17
1,14	SPZ1	spermatogenic leucine zipper 1
1,14	TRPC7	transient receptor potential cation channel, subfamily C, member 7
1,14	PHLDA1	pleckstrin homology-like domain, family A, member 1
1,14	LINC01225	long intergenic non-protein coding RNA 1225
1,14	CES1	carboxylesterase 1
1,14	ZNF169	zinc finger protein 169
1,14	BCL7A	Memczak2013 ANTISENSE, coding, INTERNAL, intronic best transcript NM_001024808
1,14	PLCL1	phospholipase C-like 1
1,14	ZNF14	zinc finger protein 14
1,14	RHOXF2; RHOXF2B	Rhox homeobox family, member 2; Rhox homeobox family, member 2B
1,14	WBP2	WW domain binding protein 2
1,14	PTCD1	pentatricopeptide repeat domain 1
1,14	ZNF804B	zinc finger protein 804B
1,14	COL7A1; MIR711	collagen, type VII, alpha 1; microRNA 711
1,14	FMN1	formin 1
1,14	RNF135	ring finger protein 135
1,14	CLEC17A	C-type lectin domain family 17, member A
1,14	MAPK11	mitogen-activated protein kinase 11
1,14	CRLF3	cytokine receptor-like factor 3

1,14	KLHL25	Memczak2013 ALT_ACCEPTOR, ALT_DONOR, coding, INTERNAL, intronic best transcript NM_022480
1,14	MTMR11	myotubularin related protein 11
1,14	GAL3ST1	galactose-3-O-sulfotransferase 1
1,14	SLC22A1	solute carrier family 22 (organic cation transporter), member 1
1,14	TRIM29	tripartite motif containing 29
1,14	RAMP1	receptor (G protein-coupled) activity modifying protein 1
1,14	URM1	ubiquitin related modifier 1
1,14	ZNF232	zinc finger protein 232
1,14	ELK3	ELK3, ETS-domain protein (SRF accessory protein 2)
1,14	UBP1	upstream binding protein 1 (LBP-1a)
1,14	HADHA	Transcript Identified by AceView, Entrez Gene ID(s) 3030
1,14	PLEKHS1	pleckstrin homology domain containing, family S member 1
1,14	KRTAP4-3	keratin associated protein 4-3
1,14	OR2AT4	olfactory receptor, family 2, subfamily AT, member 4
1,14	MYRFL	myelin regulatory factor-like
1,14	NRG1	neuregulin 1
1,14	IL2RA	interleukin 2 receptor, alpha
1,14	FABP7	fatty acid binding protein 7, brain
1,14	RUFY2	RUN and FYVE domain containing 2
1,14	CT45A1	cancer/testis antigen family 45, member A1
1,14	CHUK	conserved helix-loop-helix ubiquitous kinase
1,14	ATL1	atlastin GTPase 1
1,14	DNAH1	dynein, axonemal, heavy chain 1
1,14	OR7G3	olfactory receptor, family 7, subfamily G, member 3
1,14	MYT1	myelin transcription factor 1
1,14	PRPF39	pre-mRNA processing factor 39
1,14	OR8B12	olfactory receptor, family 8, subfamily B, member 12
1,14	ZNF30	zinc finger protein 30
1,13	ZCCHC13	zinc finger, CCHC domain containing 13
1,13	IDO2	indoleamine 2,3-dioxygenase 2
1,13	ELF4	Memczak2013 ALT_DONOR, coding, INTERNAL, intronic best transcript NM_001421
1,13	CCDC89	coiled-coil domain containing 89
1,13	ATOX1	antioxidant 1 copper chaperone
1,13	BFSP1	beaded filament structural protein 1, filensin
1,13	ACHE	acetylcholinesterase (Yt blood group)
1,13	NODAL	nodal growth differentiation factor
1,13	LIPF	lipase, gastric
1,13	TRPA1	transient receptor potential cation channel, subfamily A, member 1
1,13	ZNF862	zinc finger protein 862
1,13	TMEM242	transmembrane protein 242
1,13	BBC3; MIR3191	BCL2 binding component 3; microRNA 3191

1,13	SEMA6B	sema domain, transmembrane domain (TM), and cytoplasmic domain, (semaphorin) 6B
1,13	CABYR	calcium binding tyrosine-(Y)-phosphorylation regulated
1,13	MTPAP	mitochondrial poly(A) polymerase
1,13	RWDD3; TMEM56; TMEM56-RWDD3	RWD domain containing 3; transmembrane protein 56; TMEM56-RWDD3 readthrough
1,13	SNIP1	Smad nuclear interacting protein 1
1,13	SLC34A1	solute carrier family 34 (type II sodium/phosphate cotransporter), member 1
1,13	CORO6	coronin 6
1,13	F10	coagulation factor X
1,13	OR4N4; OR4N3P; OR4M2	olfactory receptor, family 4, subfamily N, member 4; olfactory receptor, family 4, subfamily N, member 3 pseudogene; olfactory receptor, family 4, subfamily M, member 2
1,13	CLEC1B	C-type lectin domain family 1, member B
1,13	ZNF280B	zinc finger protein 280B
1,13	TRIM16	tripartite motif containing 16
1,13	HMHA1	histocompatibility (minor) HA-1
1,13	NTRK1	neurotrophic tyrosine kinase, receptor, type 1
1,13	RPA4	replication protein A4
1,13	ZNF35	zinc finger protein 35
1,13	TCP11X2	t-complex 11 family, X-linked 2
1,13	GSN	gelsolin
1,13	TJAP1	tight junction associated protein 1 (peripheral)
1,13	SLC19A1	solute carrier family 19 (folate transporter), member 1
1,13	DLX5	distal-less homeobox 5
1,13	HERC5	HECT and RLD domain containing E3 ubiquitin protein ligase 5
1,13	OR6N2	olfactory receptor, family 6, subfamily N, member 2
1,13	SLC43A3	solute carrier family 43, member 3
1,13	HSD17B13	hydroxysteroid (17-beta) dehydrogenase 13
1,13	NUP188	Memczak2013 ANTISENSE, coding, INTERNAL, UTR3 best transcript NM_015354
1,13	CD2BP2	CD2 (cytoplasmic tail) binding protein 2
1,13	WHAMM	WAS protein homolog associated with actin, golgi membranes and microtubules
1,13	DUOX1	dual oxidase 1
1,13	IL2	interleukin 2
1,13	RAD9A	RAD9 checkpoint clamp component A
1,13	FAM19A2	family with sequence similarity 19 (chemokine (C-C motif)-like), member A2
1,13	TMPRSS6	transmembrane protease, serine 6
1,13	OBP2A	odorant binding protein 2A
1,13	TBC1D3	TBC1 domain family, member 3
1,13	EXOC6	exocyst complex component 6
1,13	EIF1AY	eukaryotic translation initiation factor 1A, Y-linked

1,13	BMP7	bone morphogenetic protein 7
1,13	MED9	mediator complex subunit 9
1,13	RP11-380G5.2; PTEN	Jeck2013 ALT_ACCEPTOR, ALT_DONOR, coding, INTERNAL, intronic best transcript NM_000314; novel transcript, sense intronic to PTEN
1,13	NKX3-1	NK3 homeobox 1
1,13	MS4A8	membrane-spanning 4-domains, subfamily A, member 8
1,13	L3MBTL4	Memczak2013 ANTISENSE, coding, INTERNAL, intronic best transcript NM_173464
1,13	RNASE1	ribonuclease, RNase A family, 1 (pancreatic)
1,13	OR4B1	olfactory receptor, family 4, subfamily B, member 1
1,13	HMBS	hydroxymethylbilane synthase
1,13	SHISA9	shisa family member 9
1,13	GABBR1	gamma-aminobutyric acid (GABA) B receptor, 1
1,13	KIAA0753	KIAA0753
1,13	BIRC3	baculoviral IAP repeat containing 3
1,13	GPM6A	glycoprotein M6A
1,13	GDF10	growth differentiation factor 10
1,13	TMEM31	transmembrane protein 31
1,13	ARHGEF6	Rac/Cdc42 guanine nucleotide exchange factor 6
1,13	LRRC4B	leucine rich repeat containing 4B
1,13	SLC24A1	solute carrier family 24 (sodium/potassium/calcium exchanger), member 1
1,13	NLRP10	NLR family, pyrin domain containing 10
1,13	MCRS1	microspherule protein 1
1,13	UTP4	UTP4 small subunit (SSU) processome component
1,13	VAT1L	vesicle amine transport 1-like
1,13	NOXO1	NADPH oxidase organizer 1
1,13	ABRA	actin binding Rho activating protein
1,13	SLC22A24	solute carrier family 22, member 24
1,13	YIPF2	Yip1 domain family member 2
1,13	METTL7B	methyltransferase like 7B
1,13	CCDC78	coiled-coil domain containing 78
1,13	SLC38A2	solute carrier family 38, member 2
1,13	TANGO2	transport and golgi organization 2 homolog
1,13	DSC1	desmocollin 1
1,13	OR4D11	olfactory receptor, family 4, subfamily D, member 11
1,13	OR5H15; OR5H5P	olfactory receptor, family 5, subfamily H, member 15; olfactory receptor, family 5, subfamily H, member 5 pseudogene
1,13	BRINP2	bone morphogenetic protein/retinoic acid inducible neural-specific 2
1,13	AMTN	amelotin
1,13	NRIP3	nuclear receptor interacting protein 3
1,13	NOV	nephroblastoma overexpressed
1,13	C19orf54	chromosome 19 open reading frame 54
1,13	TPRA1	transmembrane protein, adipocyte asscociated 1
1,13	P2RY10	purinergic receptor P2Y, G-protein coupled, 10
1,13	MTBP	MDM2 binding protein

1,13	TRIM35	tripartite motif containing 35
1,13	SLC1A1	solute carrier family 1 (neuronal/epithelial high affinity glutamate transporter, system Xag), member 1
1,13	KLHL30	kelch-like family member 30
1,13	LOC388282; CTD-2600O9.1	uncharacterized LOC388282; novel transcript
1,13	ZNRF1	zinc and ring finger 1, E3 ubiquitin protein ligase
1,13	DDC	dopa decarboxylase
1,13	RLN2	relaxin 2
1,13	CLEC6A	C-type lectin domain family 6, member A
1,13	NLRP10	NLR family, pyrin domain containing 10
1,13	DKK2	dickkopf WNT signaling pathway inhibitor 2
1,13	UCHL1	ubiquitin C-terminal hydrolase L1
1,13	KRTAP19-3	keratin associated protein 19-3
1,13	SLC41A3	solute carrier family 41, member 3
1,13	SLC6A7	solute carrier family 6 (neurotransmitter transporter), member 7
1,13	NAALAD2	N-acetylated alpha-linked acidic dipeptidase 2
1,13	UGCG	UDP-glucose ceramide glucosyltransferase
1,13	FANCE	Fanconi anemia complementation group E
1,13	DDN	dendrin
1,13	RCHY1	ring finger and CHY zinc finger domain containing 1, E3 ubiquitin protein ligase
1,13	APOL1	apolipoprotein L1
1,13	NMS	neuromedin S
1,13	PSEN2	presenilin 2
1,13	FEZ1	fasciculation and elongation protein zeta 1
1,13	MT1B; MT1CP	metallothionein 1B; metallothionein 1C, pseudogene
1,13	MS4A13	membrane-spanning 4-domains, subfamily A, member 13
1,13	GHRH	growth hormone releasing hormone
1,13	ARHGEF26	Rho guanine nucleotide exchange factor 26
1,13	KLHL35	kelch-like family member 35
1,13	LRRTM4	leucine rich repeat transmembrane neuronal 4
1,13	MED11	mediator complex subunit 11
1,13	QRFPR	pyroglutamylated RFamide peptide receptor
1,13	USP17L3; USP17L8	ubiquitin specific peptidase 17-like family member 3; ubiquitin specific peptidase 17-like family member 8
1,13	PROK2	prokineticin 2
1,13	TCF7L1	transcription factor 7-like 1 (T-cell specific, HMG-box)
1,13	MKRN3	makorin ring finger protein 3
1,13	LINC00174	long intergenic non-protein coding RNA 174
1,13	DNAJA4	DnaJ (Hsp40) homolog, subfamily A, member 4
1,13	CLIC5	chloride intracellular channel 5
1,13	CAV2	caveolin 2
1,13	FCRL6	Fc receptor-like 6
1,13	DDX25	DEAD (Asp-Glu-Ala-Asp) box helicase 25
1,13	APLF	aprataxin and PNKP like factor
1,13	CHRNA4	cholinergic receptor, nicotinic alpha 4

1,13	CDKL4	cyclin-dependent kinase-like 4
1,13	ELF1	Transcript Identified by AceView, Entrez Gene ID(s) 1997; 100128628
1,13	KIRREL3	kin of IRRE like 3 (Drosophila)
1,13	SLC45A4	solute carrier family 45, member 4
1,13	MAP1LC3C	microtubule associated protein 1 light chain 3 gamma
1,13	PSG2	pregnancy specific beta-1-glycoprotein 2
1,13	HIST2H3D	histone cluster 2, H3d
1,13	KPNA3	karyopherin alpha 3 (importin alpha 4)
1,13	KRT39	keratin 39, type I
1,13	FBXL6	F-box and leucine-rich repeat protein 6
1,13	USP40	ubiquitin specific peptidase 40
1,13	SPATA1	spermatogenesis associated 1
1,13	SHROOM4	shroom family member 4
1,13	MESDC1	mesoderm development candidate 1
1,13	LOC401052; AC022007.5; TMEM111	uncharacterized LOC401052; Salzman2013 ANNOTATED, coding, INTERNAL, OVERLAPTX, OVEXON, UTR5 best transcript NM_001008737; novel transcript; Transcript Identified by AceView, Entrez Gene ID(s) 401052; 55831
1,13	ATP6V0E2	ATPase, H+ transporting V0 subunit e2
1,13	N6AMT1	N-6 adenine-specific DNA methyltransferase 1 (putative)
1,13	CTNNBIP1	catenin, beta interacting protein 1
1,13	FKBP3	FK506 binding protein 3
1,13	RASL11A	RAS-like, family 11, member A
1,13	MSTO1; MSTO2P	misato 1, mitochondrial distribution and morphology regulator; misato family member 2, pseudogene
1,13	SNX2	sorting nexin 2
1,13	COPRS	coordinator of PRMT5, differentiation stimulator
1,13	C1orf106	chromosome 1 open reading frame 106
1,13	SLC36A1	solute carrier family 36 (proton/amino acid symporter), member 1
1,13	KCNH4	potassium channel, voltage gated eag related subfamily H, member 4
1,13	ATXN3L	ataxin 3-like
1,13	LDHD	lactate dehydrogenase D
1,13	TAS2R10	taste receptor, type 2, member 10
1,13	EPHA4	EPH receptor A4
1,13	EMC9	ER membrane protein complex subunit 9
1,13	UMODL1	uromodulin-like 1
1,13	LOC388882; AP000345.1	uncharacterized LOC388882; novel transcript
1,13	ANXA13	annexin A13
1,13	PCBP3	poly(rC) binding protein 3
1,13	KIAA1456	KIAA1456
1,13	SCAF11	Memczak2013 ANTISENSE, CDS, coding, INTERNAL best transcript NM_004719

1,13	BAGE5	Synthetic construct Homo sapiens clone IMAGE:100062553, MGC:190557 B melanoma antigen family, member 5 (BAGE5) mRNA, encodes complete protein.; B melanoma antigen family, member 5 [Source:EntrezGene;Acc:85316]
1,13	SAMD3	sterile alpha motif domain containing 3
1,13	DMAP1	DNA methyltransferase 1 associated protein 1
1,13	CFAP43	cilia and flagella associated protein 43
1,13	CS	citrate synthase
1,13	TSSK1B	testis-specific serine kinase 1B
1,13	STEAP1	six transmembrane epithelial antigen of the prostate 1
1,13	MED21	mediator complex subunit 21
1,13	ABHD18	abhydrolase domain containing 18
1,13	AQP9	aquaporin 9
1,13	RUNDC3A	RUN domain containing 3A
1,13	GNG13	guanine nucleotide binding protein (G protein), gamma 13
1,13	PPIL2	peptidylprolyl isomerase (cyclophilin)-like 2
1,13	ZNF785	zinc finger protein 785
1,13	IFT57	intraflagellar transport 57
1,13	DAAM2	dishevelled associated activator of morphogenesis 2
1,13	IL12B	interleukin 12B
1,13	COL16A1	collagen, type XVI, alpha 1
1,13	DEFB121	defensin, beta 121
1,13	DNAAF1	dynein, axonemal, assembly factor 1
1,13	SLC35F3	solute carrier family 35, member F3
1,13	NSUN6	NOP2/Sun domain family, member 6
1,13	C6orf25	chromosome 6 open reading frame 25
1,13	OR9G1; OR9G9	olfactory receptor, family 9, subfamily G, member 1; olfactory receptor, family 9, subfamily G, member 9
1,13	TBC1D10C	TBC1 domain family, member 10C
1,13	RNF138	ring finger protein 138, E3 ubiquitin protein ligase
1,13	IL17REL	interleukin 17 receptor E-like
1,13	HSPB8	heat shock 22kDa protein 8
1,13	HYOU1	hypoxia up-regulated 1
1,13	BEST2	bestrophin 2
1,13	LOC100288966; POTED; BX072566.1	POTE ankyrin domain family member D-like; Synthetic construct Homo sapiens clone IMAGE:100066427, MGC:195564 POTE ankyrin domain family, member D (POTED) mRNA, encodes complete protein.; uncharacterized protein LOC100288966 [Source:RefSeq peptide;Acc:NP_001244291]
1,13	FAM114A2	family with sequence similarity 114, member A2
1,13	ALX1	ALX homeobox 1
1,13	SPANXN3	SPANX family, member N3
1,13	PPP1CA	protein phosphatase 1, catalytic subunit, alpha isozyme
1,13	ALPPL2	alkaline phosphatase, placental like 2
1,13	PLK2	polo-like kinase 2

1,13	SPI1	Spi-1 proto-oncogene
1,13	HHIPL2	HHIP-like 2
1,13	CARF	calcium responsive transcription factor
1,13	NUGGC	nuclear GTPase, germinal center associated
1,13	HLA-DQB2	major histocompatibility complex, class II, DQ beta 2
1,13	TEX10	testis expressed 10
1,13	TVP23A	trans-golgi network vesicle protein 23 homolog A (S. cerevisiae)
1,13	MXD1	MAX dimerization protein 1
1,13	ZNF763	zinc finger protein 763
1,13	NANOS3	nanos homolog 3 (Drosophila)
1,13	ADCY2	Transcript Identified by AceView, Entrez Gene ID(s) 108
1,13	HARS2	histidyl-tRNA synthetase 2, mitochondrial
1,13	LRRC14B	leucine rich repeat containing 14B
1,13	CRKL	Memczak2013 ANTISENSE, CDS, coding, INTERNAL best transcript NM_005207
1,13	SPATS2L	spermatogenesis associated, serine-rich 2-like
1,13	COL1A1	Jeck2013 ANTISENSE, coding, INTERNAL, intronic best transcript NM_000088
1,13	WDR72	WD repeat domain 72
1,13	CD1B	CD1b molecule
1,13	LGALS14	lectin, galactoside-binding, soluble, 14
1,13	SH3GLB2	SH3-domain GRB2-like endophilin B2
1,13	TMEM74B	transmembrane protein 74B
1,13	ARHGDIB	Rho GDP dissociation inhibitor (GDI) beta
1,13	AGAP9; BMS1P6	ArfGAP with GTPase domain, ankyrin repeat and PH domain 9; BMS1 ribosome biogenesis factor pseudogene 6
1,13	RASGRF2	Ras protein-specific guanine nucleotide-releasing factor 2
1,13	TIMP3	TIMP metallopeptidase inhibitor 3
1,13	FAM171A2	family with sequence similarity 171, member A2
1,13	USP17L24; USP17L26; USP17L5; USP17L27; USP17L29; USP17L30	ubiquitin specific peptidase 17-like family member 24; ubiquitin specific peptidase 17-like family member 26; ubiquitin specific peptidase 17-like family member 5; ubiquitin specific peptidase 17-like family member 27; ubiquitin specific peptidase 17-like family member 29; ubiquitin specific peptidase 17-like family member 30
1,13	USP17L25; USP17L27	ubiquitin specific peptidase 17-like family member 25; ubiquitin specific peptidase 17-like family member 27
1,13	MYH11	myosin, heavy chain 11, smooth muscle
1,13	PIP5K1A	phosphatidylinositol-4-phosphate 5-kinase, type I, alpha
1,13	CSGALNACT1	chondroitin sulfate N-acetylgalactosaminyltransferase 1
1,13	ETV1	ets variant 1
1,13	C1D	C1D nuclear receptor corepressor
1,13	FGFR1OP	FGFR1 oncogene partner

1,13	PLCD4	phospholipase C, delta 4
1,13	TBCD	Transcript Identified by AceView, Entrez Gene ID(s) 6904
1,13	ATG12	autophagy related 12
1,13	STAC	SH3 and cysteine rich domain
1,13	KLHL25	kelch-like family member 25
1,13	ZCCHC7	zinc finger, CCHC domain containing 7
1,13	ATG16L1	autophagy related 16-like 1
1,13	ARMC12	armadillo repeat containing 12
1,13	RP11-569G13.3; LOC158434; setora	uncharacterized protein LOC158434 [Source:RefSeq peptide;Acc:NP_001243337]; uncharacterized LOC158434; Protein LOC158434 [Source:UniProtKB/TrEMBL;Acc:A0A087X204]; novel transcript; Transcript Identified by AceView
1,13	SULT4A1	sulfotransferase family 4A member 1
1,13	FLJ33360; CTD-2324F15.2	FLJ33360 protein; FLJ33360 protein [Source:EntrezGene;Acc:401172]; putative novel transcript
1,13	C14orf28	chromosome 14 open reading frame 28
1,13	ZNFX1	zinc finger, NFX1-type containing 1
1,13	sep-03	septin 3
1,13	TGFB1I1	transforming growth factor beta 1 induced transcript 1
1,13	HPS3	Hermansky-Pudlak syndrome 3
1,13	C6orf1	chromosome 6 open reading frame 1
1,13	ZNF43	zinc finger protein 43
1,13	CYP4B1	cytochrome P450, family 4, subfamily B, polypeptide 1
1,13	IFNA10	interferon, alpha 10
1,13	SSX4B; SSX4	synovial sarcoma, X breakpoint 4B; synovial sarcoma, X breakpoint 4
1,13	DTNA	Transcript Identified by AceView, Entrez Gene ID(s) 1837
1,13	ITIH6	inter-alpha-trypsin inhibitor heavy chain family, member 6
1,13	ART3	ADP-ribosyltransferase 3
1,13	CCL4L2; CCL4; CCL4L1	chemokine (C-C motif) ligand 4-like 2; chemokine (C-C motif) ligand 4; chemokine (C-C motif) ligand 4-like 1
1,13	C15orf52	chromosome 15 open reading frame 52
1,13	TGFB1	transforming growth factor beta 1
1,13	BTNL3	butyrophilin-like 3
1,13	CARD16	caspase recruitment domain family, member 16
1,13	TCERG1	Transcript Identified by AceView, Entrez Gene ID(s) 10915
1,13	CLEC4C	C-type lectin domain family 4, member C
1,13	OR51A4	olfactory receptor, family 51, subfamily A, member 4
1,13	CDHR4	cadherin-related family member 4
1,13	OR7D2	olfactory receptor, family 7, subfamily D, member 2
1,13	CCDC175	coiled-coil domain containing 175
1,13	TINAGL1	tubulointerstitial nephritis antigen-like 1
1,13	ZNF35	zinc finger protein 35

1,13	BOC	BOC cell adhesion associated, oncogene regulated
1,13	PRRX1	paired related homeobox 1
1,13	KRT13	keratin 13, type I
1,13	TMEM266	transmembrane protein 266
1,13	KCTD21-AS1	KCTD21 antisense RNA 1
1,13	NEU2	sialidase 2 (cytosolic sialidase)
1,13	RORB	RAR-related orphan receptor B
1,13	RBM14	RNA binding motif protein 14
1,13	CAPNS2	calpain, small subunit 2
1,13	C8orf82; LRRC24	chromosome 8 open reading frame 82; leucine rich repeat containing 24
1,13	SMIM3	small integral membrane protein 3
1,13	BMX	BMX non-receptor tyrosine kinase
1,13	OVGP1	oviductal glycoprotein 1
1,13	OR7D4	olfactory receptor, family 7, subfamily D, member 4
1,13	LY6E	lymphocyte antigen 6 complex, locus E
1,13	FMNL3	formin like 3
1,13	GK2	glycerol kinase 2
1,13	LINC00222	long intergenic non-protein coding RNA 222
1,13	LEF1	lymphoid enhancer-binding factor 1
1,13	UGT2B11	UDP glucuronosyltransferase 2 family, polypeptide B11
1,13	FAM181B	family with sequence similarity 181, member B
1,13	NRSN1	neurensin 1
1,13	TMEM27	transmembrane protein 27
1,13	STARD13	StAR-related lipid transfer domain containing 13
1,13	HCG9	HLA complex group 9 (non-protein coding)
1,13	ADGB	androglobin
1,12	RHBG	Rh family, B glycoprotein (gene/pseudogene)
1,12	MED17	mediator complex subunit 17
1,12	OR4F17	olfactory receptor, family 4, subfamily F, member 17
1,12	TUBB2B	tubulin, beta 2B class IIb
1,12	LINC00116	long intergenic non-protein coding RNA 116
1,12	GRPEL1	GrpE-like 1, mitochondrial (E. coli)
1,12	B4GALNT3	beta-1,4-N-acetyl-galactosaminyl transferase 3
1,12	ZKSCAN7	zinc finger with KRAB and SCAN domains 7
1,12	ZNF681	zinc finger protein 681
1,12	CLK4	CDC like kinase 4
1,12	DZIP1	DAZ interacting zinc finger protein 1
1,12	CLEC4A	C-type lectin domain family 4, member A
1,12	ADCK1	aarF domain containing kinase 1
1,12	MDK	midkine (neurite growth-promoting factor 2)
1,12	PLAT	plasminogen activator, tissue
1,12	MTCH2	mitochondrial carrier 2
1,12	FMO2	flavin containing monooxygenase 2
1,12	MYADM	myeloid-associated differentiation marker
1,12	HAUS5	HAUS augmin like complex subunit 5
1,12	NKX2-1	NK2 homeobox 1
1,12	KLHL7	kelch-like family member 7
1,12	HOXA3	homeobox A3

1,12	SVOP	SV2 related protein
1,12	LMAN1L	lectin, mannose-binding, 1 like
1,12	ZNF708	zinc finger protein 708
1,12	APOBEC4	apolipoprotein B mRNA editing enzyme, catalytic polypeptide-like 4 (putative)
1,12	HTN3	histatin 3
1,12	TMEM145	transmembrane protein 145
1,12	CCDC181	coiled-coil domain containing 181
1,12	RAPSN	receptor-associated protein of the synapse
1,12	NME8	NME/NM23 family member 8
1,12	MT1X	metallothionein 1X
1,12	LOC643802	u3 small nucleolar ribonucleoprotein protein MPP10-like
1,12	PAX9	paired box 9
1,12	FAM205A	family with sequence similarity 205, member A
1,12	NCEH1	neutral cholesterol ester hydrolase 1
1,12	ERV3-1; ZNF117	endogenous retrovirus group 3, member 1; zinc finger protein 117
1,12	LHB	luteinizing hormone beta polypeptide
1,12	OR2G6	olfactory receptor, family 2, subfamily G, member 6
1,12	CCNE1	cyclin E1
1,12	DUX4L6	double homeobox 4 like 6
1,12	DUX4L5	double homeobox 4 like 5
1,12	DUX4L1	double homeobox 4 like 1
1,12	DUX4L3	double homeobox 4 like 3
1,12	DUX4L2	double homeobox 4 like 2
1,12	DHODH	dihydroorotate dehydrogenase (quinone)
1,12	HSPA6	heat shock 70kDa protein 6 (HSP70B)
1,12	CCDC36	coiled-coil domain containing 36
1,12	RBM24	RNA binding motif protein 24
1,12	C12orf77	chromosome 12 open reading frame 77
1,12	OTOA	otoancorin
1,12	HYOU1	hypoxia up-regulated 1
1,12	PRR3	proline rich 3
1,12	TCP11	t-complex 11, testis-specific
1,12	UBLCP1	ubiquitin-like domain containing CTD phosphatase 1
1,12	UCK1	uridine-cytidine kinase 1
1,12	SLC5A11	solute carrier family 5 (sodium/inositol cotransporter), member 11
1,12	P2RX7	purinergic receptor P2X, ligand gated ion channel, 7
1,12	DOK3	docking protein 3
1,12	LINC01235	long intergenic non-protein coding RNA 1235
1,12	LEPR; LEPROT	leptin receptor; leptin receptor overlapping transcript
1,12	ZNF442	zinc finger protein 442
1,12	MYL4	myosin light chain 4
1,12	TXNRD2	Transcript Identified by AceView, Entrez Gene ID(s) 10587
1,12	WDR47	WD repeat domain 47
1,12	ZIC3	Zic family member 3
1,12	LINC01537	long intergenic non-protein coding RNA 1537

1,12	LIX1	Memczak2013 ANTISENSE, coding, INTERNAL, intronic best transcript NM_153234
1,12	PRR4	proline rich 4 (lacrimal)
1,12	TRGJ1; TRGC2; TRGJ2; TRGV9; TRGC1; TRGJP; TRGJP2	T cell receptor gamma joining 1; T cell receptor gamma constant 2; T cell receptor gamma joining 2; T cell receptor gamma variable 9; T cell receptor gamma constant 1; T cell receptor gamma joining P; T cell receptor gamma joining P2
1,12	DGUOK	deoxyguanosine kinase
1,12	GKAP1	G kinase anchoring protein 1
1,12	PTCH1	patched 1
1,12	DAOA	D-amino acid oxidase activator
1,12	NARFL	nuclear prelamin A recognition factor-like
1,12	VPS37D	vacuolar protein sorting 37 homolog D (S. cerevisiae)
1,12	SERTAD4	SERTA domain containing 4
1,12	ALKBH1	alkB homolog 1, histone H2A dioxygenase
1,12	SH3BGRL3	SH3 domain binding glutamate-rich protein like 3
1,12	MXRA5	matrix-remodelling associated 5
1,12	MTRNR2L9	MT-RNR2-like 9
1,12	NOP10	NOP10 ribonucleoprotein
1,12	PIH1D3	PIH1 domain containing 3
1,12	C1orf87	chromosome 1 open reading frame 87
1,12	KRTAP6-1	keratin associated protein 6-1
1,12	RFX6	regulatory factor X, 6
1,12	SOGA1	suppressor of glucose, autophagy associated 1
1,12	PRMT7	protein arginine methyltransferase 7
1,12	CCDC74B	coiled-coil domain containing 74B
1,12	SEPP1	selenoprotein P, plasma, 1
1,12	FRMPD4	FERM and PDZ domain containing 4
1,12	FGF6	fibroblast growth factor 6
1,12	DMC1	DNA meiotic recombinase 1
1,12	AKAP14	A kinase (PRKA) anchor protein 14
1,12	HHIPL1	HHIP-like 1
1,12	NCDN	neurochondrin
1,12	CALML3	calmodulin-like 3
1,12	PTX3	pentraxin 3, long
1,12	GJB5	gap junction protein beta 5
1,12	KLHL38	kelch-like family member 38
1,12	FOXF2	forkhead box F2
1,12	SMIM9	small integral membrane protein 9
1,12	ZNF496	zinc finger protein 496
1,12	DTX1	deltex 1, E3 ubiquitin ligase
1,12	WNT6	wingless-type MMTV integration site family, member 6
1,12	PDE2A	phosphodiesterase 2A, cGMP-stimulated
1,12	PGM5	phosphoglucomutase 5
1,12	FOXA1	forkhead box A1
1,12	GSDMC	gasdermin C
1,12	MT1L	metallothionein 1L (gene/pseudogene)
1,12	RAB37	RAB37, member RAS oncogene family

1,12	HELQ	helicase, POLQ-like
1,12	PSD4	pleckstrin and Sec7 domain containing 4
1,12	FABP4	fatty acid binding protein 4, adipocyte
1,12	SLC25A29	solute carrier family 25 (mitochondrial carnitine/acylcarnitine carrier), member 29
1,12	FAM124B	family with sequence similarity 124 member B
1,12	GDA	guanine deaminase
1,12	C1orf234	chromosome 1 open reading frame 234
1,12	GAR1	GAR1 homolog, ribonucleoprotein
1,12	YBX2	Y box binding protein 2
1,12	STIL	SCL/TAL1 interrupting locus
1,12	ZNF812P	zinc finger protein 812, pseudogene
1,12	CLDND2	claudin domain containing 2
1,12	TRPC6	transient receptor potential cation channel, subfamily C, member 6
1,12	SH3BGR	SH3 domain binding glutamate-rich protein
1,12	TSEN2	TSEN2 tRNA splicing endonuclease subunit
1,12	ARL4C	ADP-ribosylation factor like GTPase 4C
1,12	PSME1	proteasome activator subunit 1
1,12	BNIPL	BCL2/adenovirus E1B 19kD interacting protein like
1,12	CDY2A	chromodomain protein, Y-linked, 2A
1,12	CPA2	carboxypeptidase A2 (pancreatic)
1,12	ATP1A2	ATPase, Na+/K+ transporting, alpha 2 polypeptide
1,12	LRRC69; MIR4661	leucine rich repeat containing 69; microRNA 4661
1,12	MPEG1	macrophage expressed 1
1,12	C9orf139	chromosome 9 open reading frame 139
1,12	SIL1	SIL1 nucleotide exchange factor
1,12	IL2RB	interleukin 2 receptor, beta
1,12	GLS	glutaminase
1,12	CNPPD1	cyclin Pas1/PHO80 domain containing 1
1,12	TBX15	T-box 15
1,12	RBM26	Memczak2013 ANTISENSE, CDS, coding, INTERNAL best transcript NM_022118
1,12	ACSBG2	acyl-CoA synthetase bubblegum family member 2
1,12	GLIPR1L1	GLI pathogenesis-related 1 like 1
1,12	TUBGCP3	Transcript Identified by AceView, Entrez Gene ID(s) 10426
1,12	FAIM2	Fas apoptotic inhibitory molecule 2
1,12	MUC4	mucin 4, cell surface associated
1,12	POU2AF1	POU class 2 associating factor 1
1,12	WI2-2373I1.2; pajoyby	forkhead box L1-like; novel transcript; Transcript Identified by AceView
1,12	THAP1	THAP domain containing, apoptosis associated protein 1
1,12	SLC2A4	solute carrier family 2 (facilitated glucose transporter), member 4
1,12	NME6	NME/NM23 nucleoside diphosphate kinase 6
1,12	IVL	involucrin
1,12	NFE2	nuclear factor, erythroid 2

1,12	KRT23	keratin 23, type I
1,12	CYP1A1	cytochrome P450, family 1, subfamily A, polypeptide 1
1,12	ND6	NADH dehydrogenase, subunit 6 (complex I)
1,12	ACTC1	actin, alpha, cardiac muscle 1
1,12	LHFPL5	lipoma HMGIC fusion partner-like 5
1,12	KRTAP17-1	keratin associated protein 17-1
1,12	PSMC4	proteasome 26S subunit, ATPase 4
1,12	OR10G3	olfactory receptor, family 10, subfamily G, member 3
1,12	RPRML	reprimo-like
1,12	SYCE3	synaptonemal complex central element protein 3
1,12	CHAF1B	chromatin assembly factor 1, subunit B (p60)
1,12	ERICH2	glutamate rich 2
1,12	OTUD7A	OTU deubiquitinase 7A
1,12	TMEM8C	transmembrane protein 8C
1,12	MPND	MPN domain containing
1,12	B4GALT2	Memczak2013 ANTISENSE, CDS, coding, INTERNAL best transcript NM_001005417
1,12	MTRNR2L3	MT-RNR2-like 3
1,12	CYP4A11	cytochrome P450, family 4, subfamily A, polypeptide 11
1,12	CASP10	caspase 10
1,12	PRR29	proline rich 29
1,12	EIF3F	Eukaryotic translation initiation factor 3 subunit F [Source:UniProtKB/Swiss-Prot;Acc:O00303]
1,12	SETD3	Transcript Identified by AceView, Entrez Gene ID(s) 84193
1,12	KIF2C	Jeck2013 ALT_DONOR, coding, INTERNAL, intronic best transcript NM_006845
1,12	IER2	immediate early response 2
1,12	INO80E	INO80 complex subunit E
1,12	HMGN5	high mobility group nucleosome binding domain 5
1,12	TRIM74	tripartite motif containing 74
1,12	LST1	leukocyte specific transcript 1
1,12	PATE3	prostate and testis expressed 3
1,12	RNF212	ring finger protein 212
1,12	TRIM52	tripartite motif containing 52
1,12	OR2B11	olfactory receptor, family 2, subfamily B, member 11
1,12	C10orf25	chromosome 10 open reading frame 25
1,12	NUTM2B	NUT family member 2B
1,12	NRN1	neuritin 1
1,12	RBM14-RBM4	RBM14-RBM4 readthrough
1,12	NDUFA3	NADH dehydrogenase (ubiquinone) 1 alpha subcomplex, 3, 9kDa
1,12	TDRD5	tudor domain containing 5
1,12	B3GNT5	UDP-GlcNAc:betaGal beta-1,3-N-acetylglucosaminyltransferase 5
1,12	SHISA5	shisa family member 5
1,12	ACKR1	atypical chemokine receptor 1 (Duffy blood group)

1,12	FBXO31	Memczak2013 ANTISENSE, INTERNAL, intronic, ncRNA best transcript NR_024568
1,12	GAGE2D; GAGE13; GAGE2E; GAGE8; GAGE2B; GAGE2A; GAGE2C	G antigen 2D; G antigen 13; G antigen 2E; G antigen 8; G antigen 2B; G antigen 2A; G antigen 2C
1,12	BRD2	bromodomain containing 2
1,12	LOC100129307	putative UPF0607 protein ENSP00000383144
1,12	CYB561	cytochrome b561
1,12	TNKS	tankyrase, TRF1-interacting ankyrin-related ADP-ribose polymerase
1,12	LGALS8	lectin, galactoside-binding, soluble, 8
1,12	IFNG	interferon, gamma
1,12	LDLRAP1	low density lipoprotein receptor adaptor protein 1
1,12	LFNG; MIR4648	LFNG O-fucosylpeptide 3-beta-N-acetylglucosaminyltransferase; microRNA 4648
1,12	STMND1	stathmin domain containing 1
1,12	URGCP	upregulator of cell proliferation
1,12	LOC102724323; RP11-67C2.2; MARCH8	uncharacterized LOC102724323; Transcript Identified by AceView, Entrez Gene ID(s) 220972; novel transcript
1,12	FHDC1	FH2 domain containing 1
1,12	USP3	Memczak2013 ALT_ACCEPTOR, ALT_DONOR, coding, INTERNAL, intronic best transcript NM_006537
1,12	ESCO2	establishment of sister chromatid cohesion N-acetyltransferase 2
1,12	CACNA1A	calcium channel, voltage-dependent, P/Q type, alpha 1A subunit
1,12	FAM76B	family with sequence similarity 76, member B
1,12	CLSTN2	calsyntenin 2
1,12	ZNF721; ABCA11P	zinc finger protein 721; ATP binding cassette subfamily A member 11, pseudogene
1,12	GPHB5	glycoprotein hormone beta 5
1,12	RTN4R	reticulon 4 receptor
1,12	SRPK2	SRSF protein kinase 2
1,12	DEFB116	defensin, beta 116
1,12	CIDEC	cell death-inducing DFFA-like effector c
1,12	DNAJC27	DnaJ (Hsp40) homolog, subfamily C, member 27
1,12	PRSS58	protease, serine, 58
1,12	SLCO6A1	solute carrier organic anion transporter family, member 6A1
1,12	TEFM	transcription elongation factor, mitochondrial
1,12	MSRA	methionine sulfoxide reductase A
1,12	MADCAM1	mucosal vascular addressin cell adhesion molecule 1
1,12	TTLL4	tubulin tyrosine ligase-like family member 4
1,12	GOT1L1	glutamic-oxaloacetic transaminase 1-like 1
1,12	PRR13	proline rich 13

1,12	CCDC87	coiled-coil domain containing 87
1,12	FCMR	Fc fragment of IgM receptor
1,12	INSRR	insulin receptor-related receptor
1,12	BSN	bassoon presynaptic cytomatrix protein
1,12	SORBS1	Transcript Identified by AceView, Entrez Gene ID(s) 10580
1,12	ODF1	outer dense fiber of sperm tails 1
1,12	MYEF2	myelin expression factor 2
1,12	ZNF549	zinc finger protein 549
1,12	BCL11A	B-cell CLL/lymphoma 11A (zinc finger protein)
1,12	A2M	alpha-2-macroglobulin
1,12	TAS2R41	taste receptor, type 2, member 41
1,12	BMP15	bone morphogenetic protein 15
1,12	CCDC173	coiled-coil domain containing 173
1,12	PTHLH	parathyroid hormone-like hormone
1,12	ZMIZ1	zinc finger, MIZ-type containing 1
1,12	OR1J4	olfactory receptor, family 1, subfamily J, member 4
1,12	PSME3	proteasome activator subunit 3
1,12	PLA2G5	phospholipase A2, group V
1,12	DNAJB14	DnaJ (Hsp40) homolog, subfamily B, member 14
1,12	PRKX	protein kinase, X-linked
1,12	CDHR5	cadherin-related family member 5
1,12	NLGN4Y	neuroligin 4, Y-linked
1,12	ZNF235	zinc finger protein 235
1,12	CUTC	cutC copper transporter
1,12	PPP1R17	protein phosphatase 1, regulatory subunit 17
1,12	TAT	tyrosine aminotransferase
1,12	MEST	mesoderm specific transcript
1,12	PPIAL4C	peptidylprolyl isomerase A (cyclophilin A)-like 4C
1,12	SH2D4B	SH2 domain containing 4B
1,12	CDX1	caudal type homeobox 1
1,12	C18orf25	chromosome 18 open reading frame 25
1,12	U2SURP	U2 snRNP-associated SURP domain containing
1,12	ATP11A	ATPase, class VI, type 11A
1,12	EPS8L1	EPS8-like 1
1,12	CELA2B	chymotrypsin-like elastase family, member 2B
1,12	PLA2G16	phospholipase A2, group XVI
1,12	XG; XGY2	Xg blood group; Xg pseudogene, Y-linked 2
1,12	F3	coagulation factor III (thromboplastin, tissue factor)
1,12	LY86	lymphocyte antigen 86
1,12	ACTL9	actin-like 9
1,12	GDNF-AS1	GDNF antisense RNA 1 (head to head)
1,12	IQCF4; IQCF3	IQ motif containing F5 pseudogene; Transcript Identified by AceView, Entrez Gene ID(s) 401067
1,12	TGM4	transglutaminase 4
1,12	SULT1C4	sulfotransferase family 1C member 4
1,12	PADI1	peptidyl arginine deiminase, type I
1,12	DYRK4	dual specificity tyrosine-(Y)-phosphorylation regulated kinase 4

1,12	OPN1MW; OPN1MW2; OPN1MW3	opsin 1 (cone pigments), medium-wave-sensitive; opsin 1 (cone pigments), medium-wave-sensitive 2; opsin 1 (cone pigments), medium-wave-sensitive 3
1,12	NT5DC4	5-nucleotidase domain containing 4
1,12	SPNS3	spinster homolog 3 (Drosophila)
1,12	OR5AP2	olfactory receptor, family 5, subfamily AP, member 2
1,12	POTEJ	POTE ankyrin domain family, member J
1,12	GNG2	guanine nucleotide binding protein (G protein), gamma 2
1,12	CD163L1	CD163 molecule-like 1
1,12	AKAP5	A kinase (PRKA) anchor protein 5
1,12	RELA	v-rel avian reticuloendotheliosis viral oncogene homolog A
1,12	GOLGA8S	golgin A8 family, member S
1,12	SMG1	Salzman2013 ALT_ACCEPTOR, ALT_DONOR, coding, INTERNAL, intronic best transcript NM_015092
1,12	TEP1	telomerase-associated protein 1
1,12	PRAMEF9	
1,12	CBX7	chromobox homolog 7
1,12	OR51G1	olfactory receptor, family 51, subfamily G, member 1 (gene/pseudogene)
1,12	CRYGN	crystallin gamma N
1,12	CYP2D6	cytochrome P450, family 2, subfamily D, polypeptide 6
1,12	PLGRKT	plasminogen receptor, C-terminal lysine transmembrane protein
1,12	ZFP41	ZFP41 zinc finger protein
1,12	COG1	component of oligomeric golgi complex 1
1,12	SPACA5B	sperm acrosome associated 5B
1,12	SCAP	Memczak2013 ANTISENSE, CDS, coding, INTERNAL best transcript NM_012235
1,12	PCDHB1	protocadherin beta 1
1,12	LUM	lumican
1,12	C20orf202	chromosome 20 open reading frame 202
1,12	PRELP	proline/arginine-rich end leucine-rich repeat protein
1,12	SOD3	superoxide dismutase 3, extracellular
1,12	PKD1L3	polycystic kidney disease 1-like 3
1,12	FAM184B	family with sequence similarity 184, member B
1,12	OR5B17	olfactory receptor, family 5, subfamily B, member 17
1,12	ZSCAN23	zinc finger and SCAN domain containing 23
1,12	ZNF358	zinc finger protein 358
1,12	ZNF91	zinc finger protein 91
1,12	TMPRSS2	transmembrane protease, serine 2
1,12	CCDC39	coiled-coil domain containing 39
1,12	EXTL1	exostosin-like glycosyltransferase 1
1,12	USP25	ubiquitin specific peptidase 25
1,12	TYW1	tRNA-yW synthesizing protein 1 homolog (S. cerevisiae)
1,12	ZNF703	zinc finger protein 703

1,12	SFRP2	secreted frizzled-related protein 2
1,12	FLJ45513; RP11-304F15.3	uncharacterized LOC729220; novel transcript antisense to TAC4
1,12	CLN8	ceroid-lipofuscinosis, neuronal 8
1,12	CFTR	cystic fibrosis transmembrane conductance regulator
1,12	IL22	interleukin 22
1,12	TSPYL4	TSPY-like 4
1,12	SOSTDC1	sclerostin domain containing 1
1,12	C11orf95	chromosome 11 open reading frame 95
1,12	SNRPD3	small nuclear ribonucleoprotein D3 polypeptide
1,12	KCTD13	potassium channel tetramerization domain containing 13
1,12	LDOC1	leucine zipper, down-regulated in cancer 1
1,12	SLCO4A1	solute carrier organic anion transporter family, member 4A1
1,12	NEU3	sialidase 3 (membrane sialidase)
1,12	KRTAP5-AS1	KRTAP5-1/KRTAP5-2 antisense RNA 1
1,12	KRT6B	keratin 6B, type II
1,12	GPN2	GPN-loop GTPase 2
1,12	FAM159B	family with sequence similarity 159, member B
1,12	MEIOC	meiosis specific with coiled-coil domain
1,12	C5orf22	chromosome 5 open reading frame 22
1,12	BANF1	barrier to autointegration factor 1
1,12	PDGFRL	platelet-derived growth factor receptor-like
1,12	SAYSD1	SAYSVFN motif domain containing 1
1,12	PRAMEF2	PRAME family member 2
1,12	TRAPPC11	trafficking protein particle complex 11
1,12	USP10	ubiquitin specific peptidase 10
1,12	MLXIP	MLX interacting protein
1,12	ZNF35	zinc finger protein 35
1,12	BDH2	3-hydroxybutyrate dehydrogenase, type 2
1,12	ADAM33	ADAM metallopeptidase domain 33
1,12	BLNK	Memczak2013 ALT_ACCEPTOR, ALT_DONOR, coding, INTERNAL, intronic best transcript NM_013314
1,12	WDR86	WD repeat domain 86
1,12	RRAGD	Ras-related GTP binding D
1,12	GEMIN7	gem nuclear organelle associated protein 7
1,12	SOX2	SRY box 2
1,12	PLA2G4C	phospholipase A2, group IVC (cytosolic, calcium-independent)
1,12	LRIG1	
1,12	TMEM138	transmembrane protein 138
1,12	FAM231A	family with sequence similarity 231, member A
1,12	KCNA3	potassium channel, voltage gated shaker related subfamily A, member 3
1,11	NOP58	NOP58 ribonucleoprotein
1,11	PLEKHG5	pleckstrin homology domain containing, family G (with RhoGef domain) member 5
1,11	FAM86C1	family with sequence similarity 86, member C1

1,11	CEP68	centrosomal protein 68kDa
1,11	SLC50A1	solute carrier family 50 (sugar efflux transporter), member 1
1,11	MT1F	metallothionein 1F
1,11	LOC101928137; RP11-314D7.2	uncharacterized LOC101928137; novel transcript
1,11	TSC22D2	TSC22 domain family, member 2
1,11	MAP3K2	mitogen-activated protein kinase kinase kinase 2
1,11	DEDD2	death effector domain containing 2
1,11	TMEM39B	transmembrane protein 39B
1,11	C1orf54	chromosome 1 open reading frame 54
1,11	COX6B2	cytochrome c oxidase subunit VIb polypeptide 2 (testis)
1,11	RIMBP3	RIMS binding protein 3
1,11	ZDHHC11B	zinc finger, DHHC-type containing 11B
1,11	KDM4A	lysine (K)-specific demethylase 4A
1,11	ZNF696	zinc finger protein 696
1,11	C6orf15	chromosome 6 open reading frame 15
1,11	TIAM1	T-cell lymphoma invasion and metastasis 1
1,11	PANK4	pantothenate kinase 4
1,11	DEFB123	defensin, beta 123
1,11	CXCL13	chemokine (C-X-C motif) ligand 13
1,11	KIAA2026	KIAA2026
1,11	SHANK3	SH3 and multiple ankyrin repeat domains 3
1,11	SIRT6	sirtuin 6
1,11	ATP5G1	ATP synthase, H+ transporting, mitochondrial Fo complex subunit C1 (subunit 9)
1,11	KCTD1	potassium channel tetramerization domain containing 1
1,11	C17orf58	chromosome 17 open reading frame 58
1,11	STARD10	StAR-related lipid transfer domain containing 10
1,11	PNMAL1	paraneoplastic Ma antigen family-like 1
1,11	LOC442028; ANKRD20B	uncharacterized LOC442028; Transcript Identified by AceView, Entrez Gene ID(s) 729171
1,11	C1orf195	chromosome 1 open reading frame 195
1,11	CAMK1	calcium/calmodulin-dependent protein kinase I
1,11	FSTL5	follistatin-like 5
1,11	MANBAL	mannosidase, beta A, lysosomal-like
1,11	SORBS2	Transcript Identified by AceView, Entrez Gene ID(s) 8470
1,11	C9orf84	chromosome 9 open reading frame 84
1,11	ELP3	elongator acetyltransferase complex subunit 3
1,11	LCN1	lipocalin 1
1,11	TMPRSS11B	transmembrane protease, serine 11B
1,11	CAB39L	calcium binding protein 39-like
1,11	SRPX	sushi-repeat containing protein, X-linked
1,11	GNRH1	gonadotropin releasing hormone 1
1,11	DISP2	dispatched homolog 2 (Drosophila)
1,11	UTS2R	urotensin 2 receptor
1,11	RASL10A	RAS-like, family 10, member A

1,11	PPM1N	protein phosphatase, Mg2+/Mn2+ dependent, 1N (putative)
1,11	METAP2	methionyl aminopeptidase 2
1,11	PON1	paraoxonase 1
1,11	MS4A12	membrane-spanning 4-domains, subfamily A, member 12
1,11	HCLS1	hematopoietic cell-specific Lyn substrate 1
1,11	EPN1	epsin 1
1,11	STK11	serine/threonine kinase 11
1,11	EGR1	early growth response 1
1,11	OR2C1	olfactory receptor, family 2, subfamily C, member 1
1,11	RP11-732A19.2; TAF10	novel transcript, sense overlapping TAF10; Transcript Identified by AceView, Entrez Gene ID(s) 6881
1,11	DPPA3	developmental pluripotency associated 3
1,11	TMEM170B	transmembrane protein 170B
1,11	GDPD3	glycerophosphodiester phosphodiesterase domain containing 3
1,11	PNPO	pyridoxamine 5-phosphate oxidase
1,11	ATG16L1	autophagy related 16-like 1
1,11	NEDD4L	neural precursor cell expressed, developmentally down-regulated 4-like, E3 ubiquitin protein ligase
1,11	LTBP4	latent transforming growth factor beta binding protein 4
1,11	ANKUB1	ankyrin repeat and ubiquitin domain containing 1
1,11	RAB17	RAB17, member RAS oncogene family
1,11	FCGRT	Fc fragment of IgG, receptor, transporter, alpha
1,11	STX7	syntaxin 7
1,11	TMC1	transmembrane channel like 1
1,11	TOMM6; PRICKLE4	translocase of outer mitochondrial membrane 6 homolog (yeast); prickle homolog 4
1,11	CLC	Charcot-Leyden crystal galectin
1,11	ME3	malic enzyme 3, NADP(+)-dependent, mitochondrial
1,11	ARL11	ADP-ribosylation factor like GTPase 11
1,11	IRF2	interferon regulatory factor 2
1,11	OR1D4	olfactory receptor, family 1, subfamily D, member 4 (gene/pseudogene)
1,11	RNF43	ring finger protein 43
1,11	DLGAP1	discs, large (Drosophila) homolog-associated protein 1
1,11	OGT	O-linked N-acetylglucosamine (GlcNAc) transferase
1,11	IKZF1	IKAROS family zinc finger 1
1,11	UTP11L	UTP11-like, U3 small nucleolar ribonucleoprotein (yeast)
1,11	ACTR3B	ARP3 actin-related protein 3 homolog B (yeast)
1,11	GPX3	glutathione peroxidase 3
1,11	KIAA1211L	KIAA1211-like
1,11	ZC4H2	zinc finger, C4H2 domain containing
1,11	SULT6B1	sulfotransferase family 6B member 1
1,11	CHDH	choline dehydrogenase

1,11	PTCD2	pentatricopeptide repeat domain 2
1,11	ATG16L1	autophagy related 16-like 1
1,11	TMC7	transmembrane channel like 7
1,11	PPP1R32	protein phosphatase 1, regulatory subunit 32
1,11	DBF4	Transcript Identified by AceView, Entrez Gene ID(s) 10926
1,11	TTC5	tetratricopeptide repeat domain 5
1,11	DDB2	damage-specific DNA binding protein 2
1,11	MAPKAP1	mitogen-activated protein kinase associated protein 1
1,11	KLRD1	killer cell lectin-like receptor subfamily D, member 1
1,11	TNFRSF18	tumor necrosis factor receptor superfamily, member 18
1,11	FGFR4	fibroblast growth factor receptor 4
1,11	CNDP1	carnosine dipeptidase 1 (metallopeptidase M20 family)
1,11	RAD52	RAD52 homolog, DNA repair protein
1,11	BHMT2	betaine--homocysteine S-methyltransferase 2
1,11	C3orf84	chromosome 3 open reading frame 84
1,11	DAND5	DAN domain family member 5, BMP antagonist
1,11	EDN1	endothelin 1
1,11	SCARA5	scavenger receptor class A, member 5
1,11	KBTBD12	kelch repeat and BTB (POZ) domain containing 12
1,11	KDM4B	lysine (K)-specific demethylase 4B
1,11	TTC27; MIR4765	tetratricopeptide repeat domain 27; microRNA 4765
1,11	CYP4V2	cytochrome P450, family 4, subfamily V, polypeptide 2
1,11	GPR89A	G protein-coupled receptor 89A
1,11	PPP1R16A	protein phosphatase 1, regulatory subunit 16A
1,11	ITFG2	integrin alpha FG-GAP repeat containing 2
1,11	CCDC63	coiled-coil domain containing 63
1,11	SSX2; SSX2B	synovial sarcoma, X breakpoint 2; synovial sarcoma, X breakpoint 2B
1,11	RIC3	RIC3 acetylcholine receptor chaperone
1,11	CLEC12A	C-type lectin domain family 12, member A
1,11	ADAM21	ADAM metallopeptidase domain 21
1,11	ANKRD31	ankyrin repeat domain 31
1,11	IL17RB	interleukin 17 receptor B
1,11	ARSI	arylsulfatase family, member I
1,11	TRIM14	tripartite motif containing 14
1,11	NUTM2A	NUT family member 2A
1,11	OR6Q1	olfactory receptor, family 6, subfamily Q, member 1 (gene/pseudogene)
1,11	BRE	brain and reproductive organ-expressed (TNFRSF1A modulator)
1,11	LOC102723701; RP11-863K10.7	uncharacterized LOC102723701; novel transcript, antisense to ERLIN2
1,11	LRRD1	leucine-rich repeats and death domain containing 1
1,11	BTBD18	BTB (POZ) domain containing 18
1,11	MYL5	myosin light chain 5
1,11	ARHGEF37	Rho guanine nucleotide exchange factor 37

1,11	TNFRSF10A	tumor necrosis factor receptor superfamily, member 10a
1,11	C9orf50	chromosome 9 open reading frame 50
1,11	IFNL3	interferon, lambda 3
1,11	BHMT	betaine--homocysteine S-methyltransferase
1,11	OR9Q2	olfactory receptor, family 9, subfamily Q, member 2
1,11	FOXP3	forkhead box P3
1,11	TFF2	trefoil factor 2
1,11	HBB	hemoglobin, beta
1,11	TMEM238	transmembrane protein 238
1,11	AGAP5	ArfGAP with GTPase domain, ankyrin repeat and PH domain 5
1,11	PATL2	protein associated with topoisomerase II homolog 2 (yeast)
1,11	SHISA3	shisa family member 3
1,11	ERRFI1	ERBB receptor feedback inhibitor 1
1,11	CEACAM18	carcinoembryonic antigen-related cell adhesion molecule 18
1,11	ECHDC1	ethylmalonyl-CoA decarboxylase 1
1,11	HINT3	histidine triad nucleotide binding protein 3
1,11	C9orf153	chromosome 9 open reading frame 153
1,11	LINC01140	long intergenic non-protein coding RNA 1140
1,11	HSPB7	heat shock 27kDa protein family, member 7 (cardiovascular)
1,11	NXN	nucleoredoxin
1,11	OPLAH	5-oxoprolinase (ATP-hydrolysing)
1,11	DEC1	deleted in esophageal cancer 1
1,11	CYP2S1	cytochrome P450, family 2, subfamily S, polypeptide 1
1,11	LTB4R; LTB4R2	leukotriene B4 receptor; leukotriene B4 receptor 2
1,11	TBC1D31	TBC1 domain family, member 31
1,11	CHRNB2	cholinergic receptor, nicotinic beta 2
1,11	MRPL32	mitochondrial ribosomal protein L32
1,11	EYA2	EYA transcriptional coactivator and phosphatase 2
1,11	UBALD1	UBA-like domain containing 1
1,11	C5orf66-AS1	C5orf66 antisense RNA 1
1,11	FAM195A	family with sequence similarity 195, member A
1,11	ENTPD7	ectonucleoside triphosphate diphosphohydrolase 7
1,11	NR1D1	nuclear receptor subfamily 1, group D, member 1
1,11	CPNE6	copine VI (neuronal)
1,11	SLC35A3	solute carrier family 35 (UDP-N-acetylglucosamine (UDP-GlcNAc) transporter), member A3
1,11	PRODH; LOC102724788; AC007325.2	Homo sapiens proline dehydrogenase (oxidase) 1 (PRODH), transcript variant 2, mRNA.; Homo sapiens proline dehydrogenase (oxidase) 1 (PRODH), transcript variant 1, mRNA.; proline dehydrogenase 1, mitochondrial; Homo sapiens proline dehydrogenase (oxidase) 1, mRNA (cDNA clone MGC:148079 IMAGE:40108133), complete cds.
1,11	LARP7	La ribonucleoprotein domain family, member 7

1,11	OR2H1	olfactory receptor, family 2, subfamily H, member 1
1,11	POFUT2	protein O-fucosyltransferase 2
1,11	SMAP2	small ArfGAP2
1,11	ANKRD13A	ankyrin repeat domain 13A
1,11	C14orf80	chromosome 14 open reading frame 80
1,11	ZAN	zonadhesin (gene/pseudogene)
1,11	ZKSCAN7	zinc finger with KRAB and SCAN domains 7
1,11	OR2B3	olfactory receptor, family 2, subfamily B, member 3
1,11	TMEM247	transmembrane protein 247
1,11	MDS2	myelodysplastic syndrome 2 translocation associated
1,11	ARHGEF28	Rho guanine nucleotide exchange factor 28
1,11	LY6G6D	lymphocyte antigen 6 complex, locus G6D
1,11	CAMKMT	calmodulin-lysine N-methyltransferase
1,11	PDCL3	phosducin like 3
1,11	FAM227A	family with sequence similarity 227, member A
1,11	FRG2	FSHD region gene 2
1,11	PRKCD	protein kinase C, delta
1,11	CCDC86	coiled-coil domain containing 86
1,11	SLC4A1AP	solute carrier family 4 (anion exchanger), member 1, adaptor protein
1,11	ENPP4	ectonucleotide pyrophosphatase/phosphodiesterase 4 (putative)
1,11	NEK7	NIMA-related kinase 7
1,11	DHRSX	Jeck2013 ALT_ACCEPTOR, ALT_DONOR, coding, INTERNAL, intronic best transcript NM_145177
1,11	STX1A	syntaxin 1A (brain)
1,11	OR52N1	olfactory receptor, family 52, subfamily N, member 1
1,11	FOXD3	forkhead box D3
1,11	ARNTL2	aryl hydrocarbon receptor nuclear translocator-like 2
1,11	ZRANB2	zinc finger, RAN-binding domain containing 2
1,11	GLP1R	glucagon-like peptide 1 receptor
1,11	DSG1	desmoglein 1
1,11	SMURF2	SMAD specific E3 ubiquitin protein ligase 2
1,11	ANKRD30B	ankyrin repeat domain 30B
1,11	TIGD6	tigger transposable element derived 6
1,11	KLRC4-KLRK1; KLRK1; KLRC4	KLRC4-KLRK1 readthrough; killer cell lectin-like receptor subfamily K, member 1; killer cell lectin-like receptor subfamily C, member 4
1,11	SMTN	smoothelin
1,11	RGPD8; RGPD6	RANBP2-like and GRIP domain containing 8; RANBP2-like and GRIP domain containing 6
1,11	PIK3CD-AS1	PIK3CD antisense RNA 1
1,11	CEP152	centrosomal protein 152kDa
1,11	PEMT	phosphatidylethanolamine N-methyltransferase
1,11	PAF1	PAF1 homolog, Paf1/RNA polymerase II complex component
1,11	SFXN2	sideroflexin 2
1,11	FAM177A1	family with sequence similarity 177, member A1
1,11	FMN2; ADH5P3	formin 2; alcohol dehydrogenase 5 (class III), chi polypeptide, pseudogene 3

1,11	CYB5D2	Transcript Identified by AceView, Entrez Gene ID(s) 124936
1,11	CEP135	centrosomal protein 135kDa
1,11	ADGRF2	adhesion G protein-coupled receptor F2
1,11	LNX2	ligand of numb-protein X 2
1,11	BEX2	brain expressed X-linked 2
1,11	PISD; MIR7109	phosphatidylserine decarboxylase; microRNA 7109
1,11	C17orf78	chromosome 17 open reading frame 78
1,11	OR13C9	olfactory receptor, family 13, subfamily C, member 9
1,11	RPS25	ribosomal protein S25
1,11	BCKDK	branched chain ketoacid dehydrogenase kinase
1,11	SLC4A8	solute carrier family 4, sodium bicarbonate cotransporter, member 8
1,11	GGTLC1	gamma-glutamyltransferase light chain 1
1,11	TMEM232	transmembrane protein 232
1,11	PLSCR3; TMEM256; TMEM256-PLSCR3	phospholipid scramblase 3; transmembrane protein 256; TMEM256-PLSCR3 readthrough (NMD candidate)
1,11	MLNR	motilin receptor
1,11	OXA1L	oxidase (cytochrome c) assembly 1-like
1,11	ADH1B	alcohol dehydrogenase 1B (class I), beta polypeptide
1,11	AADAT	aminoadipate aminotransferase
1,11	COMP	cartilage oligomeric matrix protein
1,11	DEF6	DEF6 guanine nucleotide exchange factor
1,11	GCSAML	germinal center-associated, signaling and motility-like
1,11	TROAP	trophinin associated protein
1,11	C6orf52	chromosome 6 open reading frame 52
1,11	DUS2	dihydrouridine synthase 2
1,11	DLX2	distal-less homeobox 2
1,11	C10orf113	chromosome 10 open reading frame 113
1,11	APOL4	apolipoprotein L, 4
1,11	COX20	COX20 cytochrome c oxidase assembly factor
1,11	VCX3B; VCX	variable charge, X-linked 3B; variable charge, X-linked
1,11	LRRC4C	leucine rich repeat containing 4C
1,11	ACPP	acid phosphatase, prostate
1,11	BMP5	bone morphogenetic protein 5
1,11	SGCD	sarcoglycan delta
1,11	TAF9B	TAF9B RNA polymerase II, TATA box binding protein (TBP)-associated factor, 31kDa
1,11	AMZ1	archaelysin family metallopeptidase 1
1,11	WDR41	WD repeat domain 41
1,11	PAX1	paired box 1
1,11	CABP4	calcium binding protein 4
1,11	TKTL1	transketolase-like 1
1,11	PNPLA3	patatin-like phospholipase domain containing 3
1,11	BRMS1L	Transcript Identified by AceView, Entrez Gene ID(s) 84312
1,11	C14orf132	chromosome 14 open reading frame 132

1,11	OR51G2	olfactory receptor, family 51, subfamily G, member 2
1,11	SLC35F2	solute carrier family 35, member F2
1,11	C2orf27A	chromosome 2 open reading frame 27A
1,11	VCX3A; VCX	variable charge, X-linked 3A; variable charge, X-linked
1,11	AGMO	alkylglycerol monooxygenase
1,11	SMYD5	SMYD family member 5
1,11	SPERT	spermatid associated
1,11	EVI5	Transcript Identified by AceView, Entrez Gene ID(s) 7813
1,11	C9orf142	chromosome 9 open reading frame 142
1,11	DUOXA2	dual oxidase maturation factor 2
1,11	SRRM4	serine/arginine repetitive matrix 4
1,11	CDC25A	cell division cycle 25A
1,11	CCNO	cyclin O
1,11	RABGGTA	Rab geranylgeranyltransferase, alpha subunit
1,11	OR1J2	olfactory receptor, family 1, subfamily J, member 2
1,11	ZNF510	zinc finger protein 510
1,11	EIF1AX	eukaryotic translation initiation factor 1A, X-linked
1,11	CYTH4	cytohesin 4
1,11	OR12D3	olfactory receptor, family 12, subfamily D, member 3
1,11	DHX37	DEAH (Asp-Glu-Ala-His) box polypeptide 37
1,11	DEFB113	defensin, beta 113
1,11	BEND2	BEN domain containing 2
1,11	ESRRB	estrogen-related receptor beta
1,11	JMJD8	jumonji domain containing 8
1,11	TFF3	trefoil factor 3
1,11	TSSK6	testis-specific serine kinase 6
1,11	PAQR8	progestin and adipoQ receptor family member VIII
1,11	ZNF486	zinc finger protein 486
1,11	CHMP7	Transcript Identified by AceView, Entrez Gene ID(s) 91782
1,11	NRXN2	neurexin 2
1,11	MEGF6	multiple EGF-like-domains 6
1,11	MZF1	myeloid zinc finger 1
1,11	GOLGA6A	golgin A6 family, member A
1,11	FASTKD3	FAST kinase domains 3
1,11	FGF9	fibroblast growth factor 9
1,11	GAS7	growth arrest-specific 7
1,11	TERT	telomerase reverse transcriptase
1,11	ABCA12	ATP binding cassette subfamily A member 12
1,11	SOX10	SRY box 10
1,11	NT5C	5, 3-nucleotidase, cytosolic
1,11	THUMPD3-AS1	THUMPD3 antisense RNA 1
1,11	TBC1D8	TBC1 domain family, member 8 (with GRAM domain)
1,11	SLC6A11	solute carrier family 6 (neurotransmitter transporter), member 11
1,11	HIBCH	3-hydroxyisobutyryl-CoA hydrolase

1,11	POGZ	Transcript Identified by AceView, Entrez Gene ID(s) 23126
1,11	NOL7	nucleolar protein 7
1,11	ASIP	agouti signaling protein
1,11	VIM	Jeck2013 ANTISENSE, coding, INTERNAL, intronic best transcript NM_003380
1,11	CSNK2A3	casein kinase 2, alpha 3 polypeptide
1,11	XYLB	xylulokinase homolog (H. influenzae)
1,11	BCAN	brevican
1,11	AVP	arginine vasopressin
1,11	ZNF490	zinc finger protein 490
1,11	INPP5J	inositol polyphosphate-5-phosphatase J
1,11	ANTXRL	anthrax toxin receptor-like
1,11	SCML2	sex comb on midleg-like 2 (Drosophila)
1,11	GATA2	GATA binding protein 2
1,11	INTS4	integrator complex subunit 4
1,11	UBL3	ubiquitin-like 3
1,11	USP17L27; USP17L28	ubiquitin specific peptidase 17-like family member 27; ubiquitin specific peptidase 17-like family member 28
1,11	USP17L28; USP17L30	ubiquitin specific peptidase 17-like family member 28; ubiquitin specific peptidase 17-like family member 30
1,11	USP17L29; USP17L5	ubiquitin specific peptidase 17-like family member 29; ubiquitin specific peptidase 17-like family member 5
1,11	USP17L25; USP17L30	ubiquitin specific peptidase 17-like family member 25; ubiquitin specific peptidase 17-like family member 30
1,11	RNF19B	Memczak2013 ANTISENSE, CDS, coding, INTERNAL best transcript NM_153341
1,11	HIST1H4B	histone cluster 1, H4b
1,11	RHBDF1	rhomboid 5 homolog 1 (Drosophila)
1,11	TUBA3E	tubulin, alpha 3e
1,11	TMEM99	transmembrane protein 99
1,11	GTF2A2	general transcription factor IIA 2
1,11	HORMAD2	HORMA domain containing 2
1,11	ZDHHC23	zinc finger, DHHC-type containing 23
1,11	GLYATL1	glycine-N-acyltransferase-like 1
1,11	OR6B2	olfactory receptor, family 6, subfamily B, member 2
1,11	IL10RA	interleukin 10 receptor, alpha
1,11	TNFRSF11B	tumor necrosis factor receptor superfamily, member 11b
1,11	LRRC20	leucine rich repeat containing 20
1,11	NKD2	naked cuticle homolog 2 (Drosophila)
1,11	OR2T34	olfactory receptor, family 2, subfamily T, member 34
1,11	MBOAT7	membrane bound O-acyltransferase domain containing 7

1,11	SEC22B	Salzman2013 ALT_ACCEPTOR, CDS, coding, INTERNAL, OVCODE, OVEXON best transcript NM_004892; Salzman2013 ANNOTATED, CDS, coding, OVCODE, OVEXON, UTR3 best transcript NM_004892
1,11	ELMOD3	ELMO/CED-12 domain containing 3
1,11	ZSCAN5A	zinc finger and SCAN domain containing 5A
1,11	BBS4	Bardet-Biedl syndrome 4
1,11	OXNAD1	oxidoreductase NAD-binding domain containing 1
1,11	IQCA1	IQ motif containing with AAA domain 1
1,11	USP7	ubiquitin specific peptidase 7 (herpes virus-associated)
1,11	PPP2R2D	protein phosphatase 2, regulatory subunit B, delta
1,11	PRKG2	protein kinase, cGMP-dependent, type II
1,11	AKT2	v-akt murine thymoma viral oncogene homolog 2
1,11	RBM44	RNA binding motif protein 44
1,11	KIAA1462	KIAA1462
1,11	ASB18	ankyrin repeat and SOCS box containing 18
1,11	ANLN	Transcript Identified by AceView, Entrez Gene ID(s) 54443
1,11	ATP2B2	ATPase, Ca++ transporting, plasma membrane 2
1,11	FLAD1	flavin adenine dinucleotide synthetase 1
1,11	GOLT1A	golgi transport 1A
1,11	GRIN2B	glutamate receptor, ionotropic, N-methyl D-aspartate 2B
1,11	TBX5	T-box 5
1,11	ZNF98	zinc finger protein 98
1,11	MGEA5	Salzman2013 ALT_ACCEPTOR, ALT_DONOR, coding, INTERNAL, intronic best transcript NM_012215
1,11	CBX6	chromobox homolog 6
1,11	NPAS2	neuronal PAS domain protein 2
1,11	NAGS	N-acetylglutamate synthase
1,11	PLA2G2D	phospholipase A2, group IID
1,11	TMEM110	transmembrane protein 110
1,11	RRP1	ribosomal RNA processing 1
1,11	DNTTIP2	deoxynucleotidyltransferase, terminal, interacting protein 2
1,11	H2AFB1; H2AFB2; H2AFB3	H2A histone family, member B1; H2A histone family, member B2; H2A histone family, member B3
1,11	PSCA	prostate stem cell antigen
1,11	PRPF19	Memczak2013 ALT_ACCEPTOR, ALT_DONOR, coding, INTERNAL, intronic best transcript NM_014502
1,11	HOXC13	homeobox C13
1,11	HCFC2	host cell factor C2
1,11	TIPIN	TIMELESS interacting protein
1,11	SLC16A1	solute carrier family 16 (monocarboxylate transporter), member 1

1,11	OR52Z1	olfactory receptor, family 52, subfamily Z, member 1 (gene/pseudogene)
1,11	PHKG1	phosphorylase kinase, gamma 1 (muscle)
1,11	PLEKHA3	Transcript Identified by AceView, Entrez Gene ID(s) 65977
1,11	CAPZA3	capping protein (actin filament) muscle Z-line, alpha 3
1,11	MAPK12	mitogen-activated protein kinase 12
1,11	MLXIP	MLX interacting protein
1,11	AQP7	aquaporin 7
1,11	PRAMEF2	PRAME family member 2
1,11	CCDC88B	coiled-coil domain containing 88B
1,11	PDE6G	phosphodiesterase 6G, cGMP-specific, rod, gamma
1,11	INPP5B	inositol polyphosphate-5-phosphatase B
1,11	SPRYD7	SPRY domain containing 7
1,11	MDM4	MDM4, p53 regulator
1,11	NR2F2	nuclear receptor subfamily 2, group F, member 2
1,11	NHLRC1	NHL repeat containing E3 ubiquitin protein ligase 1
1,11	KLK4	kallikrein related peptidase 4
1,11	MAFTRR	MAF transcriptional regulator RNA
1,11	WNT16	wingless-type MMTV integration site family, member 16
1,11	NIPAL3	NIPA-like domain containing 3
1,11	TMC3	transmembrane channel like 3
1,11	BMP2K	BMP2 inducible kinase
1,11	NMUR1	neuromedin U receptor 1
1,11	METTL11B	methyltransferase like 11B
1,11	CFL2	cofilin 2 (muscle)
1,11	LINC00885	long intergenic non-protein coding RNA 885
1,11	TTLL3	tubulin tyrosine ligase-like family member 3
1,11	RSAD2	radical S-adenosyl methionine domain containing 2
1,11	CYP4B1	cytochrome P450, family 4, subfamily B, polypeptide 1
1,11	COL26A1	collagen, type XXVI, alpha 1
1,11	ALDH3A1	aldehyde dehydrogenase 3 family, member A1
1,11	VSIG8	V-set and immunoglobulin domain containing 8
1,11	FBP1	fructose-1,6-bisphosphatase 1
1,11	EXOC3	Memczak2013 ANTISENSE, CDS, coding, INTERNAL best transcript NM_007277
1,11	ITM2A	integral membrane protein 2A
1,11	ANHX	anomalous homeobox
1,11	CXorf23	chromosome X open reading frame 23
1,11	TMEM235	transmembrane protein 235
1,11	LGALS2	lectin, galactoside-binding, soluble, 2
1,10	IL11RA	interleukin 11 receptor, alpha
1,10	TGFB3	transforming growth factor beta 3
1,10	ABO	ABO blood group (transferase A, alpha 1-3-N-acetylgalactosaminyltransferase; transferase B, alpha 1-3-galactosyltransferase)
1,10	ERAS	ES cell expressed Ras
1,10	CHORDC1	cysteine and histidine rich domain containing 1

1,10	ZSCAN21	zinc finger and SCAN domain containing 21
1,10	ZNF852	zinc finger protein 852
1,10	SLC5A7	solute carrier family 5 (sodium/choline cotransporter), member 7
1,10	LINGO2	leucine rich repeat and Ig domain containing 2
1,10	BIRC2	baculoviral IAP repeat containing 2
1,10	ZNHIT3	zinc finger, HIT-type containing 3
1,10	FAM231B	family with sequence similarity 231, member B
1,10	MS4A2	membrane-spanning 4-domains, subfamily A, member 2
1,10	MBD3L1	methyl-CpG binding domain protein 3-like 1
1,10	TINAG	tubulointerstitial nephritis antigen
1,10	TMCC1	transmembrane and coiled-coil domain family 1
1,10	TIMD4	T-cell immunoglobulin and mucin domain containing 4
1,10	PLK5	polo-like kinase 5
1,10	DEFA3	defensin, alpha 3, neutrophil-specific
1,10	C4orf46	chromosome 4 open reading frame 46
1,10	NETO1	neuropilin (NRP) and tolloid (TLL)-like 1
1,10	OSBPL5	Memczak2013 ALT_ACCEPTOR, ALT_DONOR, coding, INTERNAL, intronic best transcript NM_020896
1,10	PI3	peptidase inhibitor 3, skin-derived
1,10	ZNF729	zinc finger protein 729
1,10	RNASET2	ribonuclease T2
1,10	MFSD14A	major facilitator superfamily domain containing 14A
1,10	UQCRHL	ubiquinol-cytochrome c reductase hinge protein like
1,10	ZNF449	zinc finger protein 449
1,10	HMGXB4	HMG box domain containing 4
1,10	TMEM251	transmembrane protein 251
1,10	ZNF45	zinc finger protein 45
1,10	NDUFA3	NADH dehydrogenase (ubiquinone) 1 alpha subcomplex, 3, 9kDa
1,10	S100A11	S100 calcium binding protein A11
1,10	OR7C2	olfactory receptor, family 7, subfamily C, member 2
1,10	FAM24B	family with sequence similarity 24, member B
1,10	C8orf89	chromosome 8 open reading frame 89
1,10	COQ4	coenzyme Q4
1,10	PNN	pinin, desmosome associated protein
1,10	PDCD1LG2	programmed cell death 1 ligand 2
1,10	MARVELD3	MARVEL domain containing 3
1,10	ARG1	arginase 1
1,10	PRDM12	PR domain containing 12
1,10	KRTAP23-1	keratin associated protein 23-1
1,10	CD46	Transcript Identified by AceView, Entrez Gene ID(s) 4179
1,10	LGALS7; LGALS7B	lectin, galactoside-binding, soluble, 7; lectin, galactoside-binding, soluble, 7B
1,10	YLPM1	YLP motif containing 1
1,10	FMN1	formin 1

1,10	SFTPA2; SFTPA1	surfactant protein A2; surfactant protein A1
1,10	GOLGA6L6	golgin A6 family-like 6
1,10	TNNI2	troponin I type 2 (skeletal, fast)
1,10	RPS6KB2	ribosomal protein S6 kinase, 70kDa, polypeptide 2
1,10	ANP32C	acidic nuclear phosphoprotein 32 family member C
1,10	EVPLL	envoplakin-like
1,10	OR5R1	olfactory receptor, family 5, subfamily R, member 1 (gene/pseudogene)
1,10	HABP2	hyaluronan binding protein 2
1,10	STIM2	stromal interaction molecule 2
1,10	GPR34	G protein-coupled receptor 34
1,10	RAB2A	Memczak2013 ALT_ACCEPTOR, ALT_DONOR, coding, INTERNAL, intronic best transcript NM_002865
1,10	CCBL2; RBMXL1	cysteine conjugate-beta lyase 2; RNA binding motif protein, X-linked-like 1
1,10	HOOK1	hook microtubule-tethering protein 1
1,10	GGT1	gamma-glutamyltransferase 1
1,10	NPS	neuropeptide S
1,10	FRMD6	FERM domain containing 6
1,10	ARHGAP28	Rho GTPase activating protein 28
1,10	TBC1D28	TBC1 domain family, member 28
1,10	HTR5A-AS1	HTR5A antisense RNA 1
1,10	FOXD4L6	forkhead box D4-like 6
1,10	ANTXR2	anthrax toxin receptor 2
1,10	MOGAT2	monoacylglycerol O-acyltransferase 2
1,10	PGM5	phosphoglucomutase 5
1,10	DAPL1; OR7E89P	death associated protein like 1; olfactory receptor, family 7, subfamily E, member 89 pseudogene
1,10	SARDH	sarcosine dehydrogenase
1,10	RNF148	ring finger protein 148
1,10	PLEKHH2	pleckstrin homology domain containing, family H (with MyTH4 domain) member 2
1,10	PRODH	proline dehydrogenase (oxidase) 1
1,10	PRDX3	peroxiredoxin 3
1,10	ARMC5	armadillo repeat containing 5
1,10	NOD2	nucleotide-binding oligomerization domain containing 2
1,10	FCHSD1	FCH and double SH3 domains 1
1,10	EPSTI1	epithelial stromal interaction 1 (breast)
1,10	DLGAP1	Memczak2013 ANTISENSE, coding, INTERNAL, intronic best transcript NM_001242765
1,10	CTAG1B; CTAG1A	cancer/testis antigen 1B; cancer/testis antigen 1A
1,10	NLRP8	NLR family, pyrin domain containing 8
1,10	CTBS	chitobiase, di-N-acetyl-
1,10	CCDC174	coiled-coil domain containing 174
1,10	ECE2	endothelin converting enzyme 2
1,10	RBP3	retinol binding protein 3, interstitial

1,10	NPPA	natriuretic peptide A
1,10	TM4SF18	transmembrane 4 L six family member 18
1,10	MKL1	megakaryoblastic leukemia (translocation) 1
1,10	SART3	squamous cell carcinoma antigen recognized by T-cells 3
1,10	LGALS9C; LGALS9B	lectin, galactoside-binding, soluble, 9C; lectin, galactoside-binding, soluble, 9B
1,10	GBGT1	globoside alpha-1,3-N-acetylgalactosaminyltransferase 1
1,10	NLRP2	NLR family, pyrin domain containing 2
1,10	WDR91	WD repeat domain 91
1,10	YEATS2	Memczak2013 ANTISENSE, coding, INTERNAL, intronic best transcript NM_018023
1,10	EPS15	epidermal growth factor receptor pathway substrate 15
1,10	SULT2B1	sulfotransferase family 2B member 1
1,10	HELT	helt bHLH transcription factor
1,10	RP11-998D10.7; FLJ10357	Transcript Identified by AceView, Entrez Gene ID(s) 55701
1,10	FAM9B	family with sequence similarity 9, member B
1,10	RRAGB	Ras-related GTP binding B
1,10	PARM1	prostate androgen-regulated mucin-like protein 1
1,10	IQCG	IQ motif containing G
1,10	ITGA9	integrin alpha 9
1,10	POLG2	polymerase (DNA directed), gamma 2, accessory subunit
1,10	PHEX	phosphate regulating endopeptidase homolog, X-linked
1,10	SALL3	spalt-like transcription factor 3
1,10	FAM204A	family with sequence similarity 204, member A
1,10	C17orf51	chromosome 17 open reading frame 51
1,10	C1orf204	chromosome 1 open reading frame 204
1,10	SUMO4	small ubiquitin-like modifier 4
1,10	FABP3	fatty acid binding protein 3, muscle and heart
1,10	SCGB1A1	secretoglobin, family 1A, member 1 (uteroglobin)
1,10	LRRN1	leucine rich repeat neuronal 1
1,10	PYROXD2; MIR1287	pyridine nucleotide-disulphide oxidoreductase domain 2; microRNA 1287
1,10	C1QL3	complement component 1, q subcomponent-like 3
1,10	TTC9C	tetratricopeptide repeat domain 9C
1,10	ADAM28	Memczak2013 ANTISENSE, coding, INTERNAL, intronic best transcript NM_014265
1,10	MEOX2	mesenchyme homeobox 2
1,10	RAI2	retinoic acid induced 2
1,10	RASD2	RASD family, member 2
1,10	ALG8	ALG8, alpha-1,3-glucosyltransferase
1,10	ZFP2	ZFP2 zinc finger protein
1,10	PRM3	protamine 3

1,10	KU-MEL-3; RP4-529N6.1	uncharacterized LOC497048; uncharacterized LOC497048 [Source:EntrezGene;Acc:497048]; putative novel transcript
1,10	RBM22	RNA binding motif protein 22
1,10	HIP1	huntingtin interacting protein 1
1,10	ST6GAL2	Transcript Identified by AceView, Entrez Gene ID(s) 84620
1,10	TEX22	testis expressed 22
1,10	ZNF480	zinc finger protein 480
1,10	TAPBPL	TAP binding protein-like
1,10	ZSCAN10	zinc finger and SCAN domain containing 10
1,10	RHBDD2	rhomboid domain containing 2
1,10	ZNF677	zinc finger protein 677
1,10	SEMA3A	sema domain, immunoglobulin domain (Ig), short basic domain, secreted, (semaphorin) 3A
1,10	ENTHD2	ENTH domain containing 2
1,10	TCP10	t-complex 10
1,10	DPAGT1	dolichyl-phosphate (UDP-N-acetylglucosamine) N-acetylglucosaminephosphotransferase 1 (GlcNAc-1-P transferase)
1,10	SLC38A9	solute carrier family 38, member 9
1,10	MCHR2	melanin-concentrating hormone receptor 2
1,10	HAAO	3-hydroxyanthranilate 3,4-dioxygenase
1,10	HEPHL1	hephaestin-like 1
1,10	CEP128	centrosomal protein 128kDa
1,10	RAB11FIP1	RAB11 family interacting protein 1 (class I)
1,10	A2BP1	Transcript Identified by AceView, Entrez Gene ID(s) 54715
1,10	ZNF625	zinc finger protein 625
1,10	SF3B1	Memczak2013 ANTISENSE, CDS, coding, INTERNAL best transcript NM_012433
1,10	RORA	Jeck2013 ALT_ACCEPTOR, ALT_DONOR, coding, INTERNAL, intronic best transcript NM_134261
1,10	RBM4B	RNA binding motif protein 4B
1,10	LGALS7B	lectin, galactoside-binding, soluble, 7B
1,10	LOC388692; FAM231D; RP11-403I13.7; RP11-403I13.8	Homo sapiens uncharacterized LOC388692 (LOC388692), long non-coding RNA.; Homo sapiens family with sequence similarity 231, member D (FAM231D), long non-coding RNA.; novel transcript
1,10	ARL14	ADP-ribosylation factor like GTPase 14
1,10	NDUFA6	NADH dehydrogenase (ubiquinone) 1 alpha subcomplex, 6, 14kDa
1,10	SYT13	synaptotagmin XIII
1,10	ARL13A	ADP-ribosylation factor like GTPase 13A
1,10	ARHGEF16	Rho guanine nucleotide exchange factor 16
1,10	SYT14	synaptotagmin XIV
1,10	CACNB2	calcium channel, voltage-dependent, beta 2 subunit
1,10	TBC1D22A-AS1	TBC1D22A antisense RNA 1
1,10	ECSCR	endothelial cell surface expressed chemotaxis and apoptosis regulator

1,10	PGRMC1	Memczak2013 ALT_ACCEPTOR, ALT_DONOR, coding, INTERNAL, intronic best transcript NM_006667
1,10	ANXA10	annexin A10
1,10	WFDC10B	WAP four-disulfide core domain 10B
1,10	SLC4A4	solute carrier family 4 (sodium bicarbonate cotransporter), member 4
1,10	CCDC185	coiled-coil domain containing 185
1,10	CLCN7	chloride channel, voltage-sensitive 7
1,10	C5orf30	chromosome 5 open reading frame 30
1,10	CEBPZOS	CEBPZ opposite strand
1,10	BPIFB2	BPI fold containing family B, member 2
1,10	C9orf131	chromosome 9 open reading frame 131
1,10	TRAF6	TNF receptor-associated factor 6, E3 ubiquitin protein ligase
1,10	CCDC138	coiled-coil domain containing 138
1,10	TMEM132E	transmembrane protein 132E
1,10	FAM161B	family with sequence similarity 161, member B
1,10	KCNJ9	potassium channel, inwardly rectifying subfamily J, member 9
1,10	UNC5CL	unc-5 family C-terminal like
1,10	GABPB1	GA binding protein transcription factor, beta subunit 1
1,10	TBC1D3P5	TBC1 domain family, member 3 pseudogene 5
1,10	LARP1B	La ribonucleoprotein domain family, member 1B
1,10	CMTM6	CKLF-like MARVEL transmembrane domain containing 6
1,10	GRB14	growth factor receptor bound protein 14
1,10	LINC00602	long intergenic non-protein coding RNA 602
1,10	CROCC	ciliary rootlet coiled-coil, rootletin
1,10	IL1RAP	interleukin 1 receptor accessory protein
1,10	LIPT2	lipoyl(octanoyl) transferase 2 (putative)
1,10	BRSK2	BR serine/threonine kinase 2
1,10	CCDC178	coiled-coil domain containing 178
1,10	RAPGEF4	Rap guanine nucleotide exchange factor 4
1,10	ELOVL2	ELOVL fatty acid elongase 2
1,10	TMEM130	transmembrane protein 130
1,10	NOL9	nucleolar protein 9
1,10	TMEM2	Transcript Identified by AceView, Entrez Gene ID(s) 23670
1,10	PLAGL1; HYMAI	pleiomorphic adenoma gene-like 1; hydatidiform mole associated and imprinted (non-protein coding)
1,10	MEGF11	multiple EGF-like-domains 11
1,10	TYW3	tRNA-yW synthesizing protein 3 homolog (S. cerevisiae)
1,10	CACNA1H	calcium channel, voltage-dependent, T type, alpha 1H subunit
1,10	PIP4K2B	phosphatidylinositol-5-phosphate 4-kinase, type II, beta
1,10	AGPAT2	1-acylglycerol-3-phosphate O-acyltransferase 2

1,10	DNAJC11	Zhang2013 ALT_ACCEPTOR, ALT_DONOR, coding, INTERNAL, intronic best transcript NM_018198
1,10	GMNC	geminin coiled-coil domain containing
1,10	MAGEA4	MAGE family member A4
1,10	OR2C3; GCSAML-AS1	olfactory receptor, family 2, subfamily C, member 3; GCSAML antisense RNA 1
1,10	OR4A15	olfactory receptor, family 4, subfamily A, member 15
1,10	SERPINB9	serpin peptidase inhibitor, clade B (ovalbumin), member 9
1,10	LCP1	lymphocyte cytosolic protein 1 (L-plastin)
1,10	IPPK	inositol 1,3,4,5,6-pentakisphosphate 2-kinase
1,10	XPR1	xenotropic and polytropic retrovirus receptor 1
1,10	PLAC1	placenta specific 1
1,10	SLC51A	solute carrier family 51, alpha subunit
1,10	FANCD2OS	FANCD2 opposite strand
1,10	FREM1	FRAS1 related extracellular matrix 1
1,10	SLC35B1	solute carrier family 35, member B1
1,10	ITGA6	integrin alpha 6
1,10	PLD6	phospholipase D family, member 6
1,10	ACAD10	acyl-CoA dehydrogenase family, member 10
1,10	COL13A1	collagen, type XIII, alpha 1
1,10	KIAA1210	KIAA1210
1,10	CYTIP	cytohesin 1 interacting protein
1,10	NUP93	nucleoporin 93kDa
1,10	NFRKB	nuclear factor related to kappaB binding protein
1,10	LSM7	LSM7 homolog, U6 small nuclear RNA and mRNA degradation associated
1,10	CFAP65	cilia and flagella associated protein 65
1,10	LMF2	lipase maturation factor 2
1,10	CYP2A13	cytochrome P450, family 2, subfamily A, polypeptide 13
1,10	ZNF683	zinc finger protein 683
1,10	HYAL4	hyaluronoglucosaminidase 4
1,10	DLC1	DLC1 Rho GTPase activating protein
1,10	CNTN5	contactin 5
1,10	ECH1	enoyl-CoA hydratase 1, peroxisomal
1,10	NRARP	NOTCH-regulated ankyrin repeat protein
1,10	HIC2	hypermethylated in cancer 2
1,10	SLC27A1	solute carrier family 27 (fatty acid transporter), member 1
1,10	TMEM260	transmembrane protein 260
1,10	RIN2	Ras and Rab interactor 2
1,10	C2orf49	chromosome 2 open reading frame 49
1,10	ENDOU	endonuclease, polyU-specific
1,10	D2HGDH	D-2-hydroxyglutarate dehydrogenase
1,10	TSSC1	tumor suppressing subtransferable candidate 1
1,10	TMEM104	transmembrane protein 104
1,10	CLECL1	C-type lectin-like 1
1,10	GPAT2	glycerol-3-phosphate acyltransferase 2, mitochondrial

1,10	PLB1	phospholipase B1
1,10	ECM1	extracellular matrix protein 1
1,10	TRPV1	transient receptor potential cation channel, subfamily V, member 1
1,10	LIM2	lens intrinsic membrane protein 2
1,10	GSR	glutathione reductase
1,10	OR2T12	olfactory receptor, family 2, subfamily T, member 12
1,10	RABEP2	rabaptin, RAB GTPase binding effector protein 2
1,10	ST6GAL1	ST6 beta-galactosamide alpha-2,6-sialyltranferase 1
1,10	OR13A1	olfactory receptor, family 13, subfamily A, member 1
1,10	C9orf116	chromosome 9 open reading frame 116
1,10	GNG3	guanine nucleotide binding protein (G protein), gamma 3
1,10	SETSIP	SET-like protein
1,10	ATG16L1	autophagy related 16-like 1
1,10	NTF3	neurotrophin 3
1,10	C17orf98	chromosome 17 open reading frame 98
1,10	SSX6	synovial sarcoma, X breakpoint 6 (pseudogene)
1,10	STKLD1	serine/threonine kinase-like domain containing 1 [Source:HGNC Symbol;Acc:HGNC:28669]
1,10	TTC36	tetratricopeptide repeat domain 36
1,10	FOLH1B	folate hydrolase 1B
1,10	ZNF366	zinc finger protein 366
1,10	ICA1L	islet cell autoantigen 1 like
1,10	SFRP4	secreted frizzled-related protein 4
1,10	CECR1	cat eye syndrome chromosome region, candidate 1
1,10	DGCR14	DiGeorge syndrome critical region gene 14
1,10	XRRA1	X-ray radiation resistance associated 1
1,10	FAM72C	family with sequence similarity 72, member C
1,10	FERMT3	fermitin family member 3
1,10	RGS5	regulator of G-protein signaling 5
1,10	MTRNR2L1	MT-RNR2-like 1
1,10	FAM208B	family with sequence similarity 208, member B
1,10	HES2	hes family bHLH transcription factor 2
1,10	SHE	Src homology 2 domain containing E
1,10	TBX18	T-box 18
1,10	POU6F2	POU class 6 homeobox 2
1,10	H2AFY2	H2A histone family, member Y2
1,10	RXRA; MIR4669	retinoid X receptor alpha; microRNA 4669
1,10	ZNF718	zinc finger protein 718
1,10	RHBDF2	rhomboid 5 homolog 2 (Drosophila)
1,10	KDM5C	Transcript Identified by AceView, Entrez Gene ID(s) 8242
1,10	ZNF561	zinc finger protein 561
1,10	APIP	APAF1 interacting protein
1,10	ROPN1L	rhophilin associated tail protein 1-like
1,10	SLC35G6	solute carrier family 35, member G6
1,10	NKAP	NFKB activating protein
1,10	S1PR5	sphingosine-1-phosphate receptor 5
1,10	ARHGAP24	Rho GTPase activating protein 24

1,10	CYP3A7; CYP3A7-CYP3A51P	cytochrome P450, family 3, subfamily A, polypeptide 7; CYP3A7-CYP3A51P readthrough
1,10	CLDN24	claudin 24
1,10	ELAC1	elaC ribonuclease Z 1
1,10	LOC81691; AC004381.6; U4atac	exonuclease NEF-sp; Putative RNA exonuclease NEF-sp [Source:UniProtKB/Swiss-Prot;Acc:Q96IC2]; Salzman2013 ANNOTATED, CDS, coding, OVCODE, OVERLAPTX, OVEXON, UTR3, UTR5 best transcript NM_001199053; Salzman2013 ANNOTATED, CDS, coding, INTERNAL, OVCODE, OVERLAPTX, OVEXON best transcript NM_001199053; Salzman2013 ANNOTATED, CDS, coding, OVCODE, OVERLAPTX, OVEXON, UTR3 best transcript NM_001199053; Transcript Identified by AceView, Entrez Gene ID(s) 81691; U4atac minor spliceosomal RNA [Source:RFAM;Acc:RF00618]
1,10	FAM133B	family with sequence similarity 133, member B
1,10	FOXRED1	FAD-dependent oxidoreductase domain containing 1
1,10	ZNF433	zinc finger protein 433
1,10	L3HYPDH	L-3-hydroxyproline dehydratase (trans-)
1,10	RGS9	regulator of G-protein signaling 9
1,10	TMEM164	transmembrane protein 164
1,10	ZFP36L1	ZFP36 ring finger protein-like 1
1,10	CNGB1	cyclic nucleotide gated channel beta 1
1,10	FZD5	frizzled class receptor 5
1,10	DNASE1	deoxyribonuclease I
1,10	TREML4	triggering receptor expressed on myeloid cells-like 4
1,10	MAGEL2	MAGE family member L2
1,10	NLRP9	NLR family, pyrin domain containing 9
1,10	TTLL12	tubulin tyrosine ligase-like family member 12
1,10	RIT2	Ras-like without CAAX 2
1,10	PKN3	protein kinase N3
1,10	RBM20	RNA binding motif protein 20
1,10	INPP5A	inositol polyphosphate-5-phosphatase A
1,10	EPS8	epidermal growth factor receptor pathway substrate 8
1,10	ACOT12	acyl-CoA thioesterase 12
1,10	PRSS1	protease, serine, 1 (trypsin 1)
1,10	AQP6	aquaporin 6, kidney specific
1,10	RNPEP	arginyl aminopeptidase (aminopeptidase B)
1,10	PTCD3	pentatricopeptide repeat domain 3
1,10	RP11-12M5.1; FAM163A	Transcript Identified by AceView, Entrez Gene ID(s) 148753; novel transcript
1,10	ANXA7	annexin A7
1,10	LRRC32	leucine rich repeat containing 32
1,10	ZNF189	zinc finger protein 189
1,10	OR8J2	olfactory receptor, family 8, subfamily J, member 2 (gene/pseudogene)
1,10	HYOU1	hypoxia up-regulated 1
1,10	PRDM4	PR domain containing 4

1,10	ZFP36	ZFP36 ring finger protein
1,10	RCN3	reticulocalbin 3, EF-hand calcium binding domain
1,10	IL18	interleukin 18
1,10	FAM27E3	family with sequence similarity 27, member E3
1,10	HABP4	hyaluronan binding protein 4
1,10	AARS2	alanyl-tRNA synthetase 2, mitochondrial
1,10	SCN3A	sodium channel, voltage gated, type III alpha subunit
1,10	FGF7	fibroblast growth factor 7
1,10	NUCB1	Memczak2013 ANTISENSE, CDS, coding, INTERNAL best transcript NM_006184
1,10	CHRNB3	cholinergic receptor, nicotinic beta 3
1,10	DMRT1	doublesex and mab-3 related transcription factor 1
1,10	DLGAP1	Transcript Identified by AceView, Entrez Gene ID(s) 9229
1,10	MYC	v-myc avian myelocytomatosis viral oncogene homolog
1,10	CTAGE1	cutaneous T-cell lymphoma-associated antigen 1
1,10	PILRA	paired immunoglobin-like type 2 receptor alpha
1,10	XKR9	X-linked Kx blood group related 9
1,10	SLC26A10	solute carrier family 26, member 10
1,10	PLGLB2	plasminogen-like B2
1,10	NOBOX	NOBOX oogenesis homeobox
1,10	LYPD6	LY6/PLAUR domain containing 6
1,10	KRT7	keratin 7, type II
1,10	CDHR3	cadherin-related family member 3
1,10	PNISR	PNN-interacting serine/arginine-rich protein
1,10	TMEFF1	transmembrane protein with EGF-like and two follistatin-like domains 1
1,10	PRAMEF7; PRAMEF8	PRAME family member 7; PRAME family member 8
1,10	EML6	echinoderm microtubule associated protein like 6
1,10	ANK3	Transcript Identified by AceView, Entrez Gene ID(s) 288
1,10	C2	complement component 2
1,10	LOC100506422; RP11-80I3.1; tayamu	putative deoxyuridine 5-triphosphate nucleotidohydrolase-like protein FLJ16323; novel transcript; Transcript Identified by AceView
1,10	NANP	N-acetylneuraminic acid phosphatase
1,10	OR9K2	olfactory receptor, family 9, subfamily K, member 2
1,10	BBS5	Bardet-Biedl syndrome 5
1,10	C8orf46	chromosome 8 open reading frame 46
1,10	IL20RA	interleukin 20 receptor, alpha
1,10	TUFT1	tuftelin 1
1,10	KLHL10	kelch-like family member 10
1,10	LMO2	LIM domain only 2 (rhombotin-like 1)
1,10	AMER1	APC membrane recruitment protein 1
1,10	ABCC9	ATP binding cassette subfamily C member 9
1,10	ADGRF3	adhesion G protein-coupled receptor F3
1,10	C10orf76	chromosome 10 open reading frame 76

1,10	ATP8A2	ATPase, aminophospholipid transporter, class I, type 8A, member 2
1,10	PTRH2	peptidyl-tRNA hydrolase 2
1,10	TNRC6C	trinucleotide repeat containing 6C
1,10	OR3A1	olfactory receptor, family 3, subfamily A, member 1
1,10	RNASEK; C17orf49; RNASEK-C17orf49	ribonuclease, RNase K; chromosome 17 open reading frame 49; RNASEK-C17orf49 readthrough
1,10	FAM168A	family with sequence similarity 168, member A
1,10	VPS11	VPS11, CORVET/HOPS core subunit [Source:HGNC Symbol;Acc:HGNC:14583]
1,10	APOBEC3A	apolipoprotein B mRNA editing enzyme, catalytic polypeptide-like 3A
1,10	ARL6	ADP-ribosylation factor like GTPase 6
1,10	NDRG1	N-myc downstream regulated 1
1,10	MRPL10	mitochondrial ribosomal protein L10
1,10	TRABD	Transcript Identified by AceView, Entrez Gene ID(s) 80305
1,10	ZNF707	zinc finger protein 707
1,10	SPATA5	Transcript Identified by AceView, Entrez Gene ID(s) 166378
1,10	ADAMTSL1	ADAMTS like 1
1,10	KRTAP10-5	keratin associated protein 10-5
1,10	TAZ	tafazzin
1,10	OR3A3	olfactory receptor, family 3, subfamily A, member 3
1,10	AKR1C1	aldo-keto reductase family 1, member C1
1,10	BDKRB2	bradykinin receptor B2
1,10	PPP1R18	protein phosphatase 1, regulatory subunit 18
1,10	SDCCAG8	serologically defined colon cancer antigen 8
1,10	NTRK2	neurotrophic tyrosine kinase, receptor, type 2
1,10	NLRC3	NLR family, CARD domain containing 3
1,10	RP11-875O11.1; RHOBTB2	Salzman2013 ANTISENSE, CDS, coding, INTERNAL, intronic, OVCODE, OVERLAPTX, OVEXON, UTR3 best transcript NM_015178; novel transcript, antisense to RHOBTB2
1,10	EPN3	Transcript Identified by AceView, Entrez Gene ID(s) 55040
1,10	PAWR	PRKC, apoptosis, WT1, regulator
1,10	NCOA1	nuclear receptor coactivator 1
1,10	OR1S2	olfactory receptor, family 1, subfamily S, member 2
1,10	C7orf33	chromosome 7 open reading frame 33
1,10	XKR6	X-linked Kx blood group related 6
1,10	HES6	hes family bHLH transcription factor 6
1,10	ADAMTS13	ADAM metallopeptidase with thrombospondin type 1 motif 13
1,10	ACMSD	aminocarboxymuconate semialdehyde decarboxylase
1,10	PROSC	proline synthetase co-transcribed homolog (bacterial)
1,10	NDRG2; MIR6717	NDRG family member 2; microRNA 6717
1,10	CERS2	ceramide synthase 2

1,10	SLC20A2	solute carrier family 20 (phosphate transporter), member 2
1,10	CDC42EP3	CDC42 effector protein (Rho GTPase binding) 3
1,10	PITPNB	Memczak2013 ANTISENSE, coding, INTERNAL, intronic best transcript NM_012399
1,10	PIK3CB	Salzman2013 ANNOTATED, CDS, coding, INTERNAL, OVCODE, OVEXON best transcript NM_006219
1,10	NPW	neuropeptide W
1,10	SMARCC2	Memczak2013 ANTISENSE, CDS, coding, INTERNAL best transcript NM_003075
1,10	CMTM3	CKLF-like MARVEL transmembrane domain containing 3
1,10	LRRC74A	leucine rich repeat containing 74A
1,10	ZNF185	zinc finger protein 185 (LIM domain)
1,10	ZNF836	zinc finger protein 836
1,10	ARHGAP11B	Rho GTPase activating protein 11B
1,10	GLRA3	glycine receptor alpha 3
1,10	ANKRD27	ankyrin repeat domain 27 (VPS9 domain)
1,10	CAPN6	calpain 6
1,10	TMEM189-UBE2V1	TMEM189-UBE2V1 readthrough
1,10	ZNF428	zinc finger protein 428
1,10	MRPS2	mitochondrial ribosomal protein S2
1,10	TUBA1C	tubulin, alpha 1c
1,10	LINC00273	long intergenic non-protein coding RNA 273
1,10	NXNL2	nucleoredoxin-like 2
1,10	RAX	retina and anterior neural fold homeobox
1,10	PLEKHA6	Transcript Identified by AceView, Entrez Gene ID(s) 22874
1,10	C15orf43	chromosome 15 open reading frame 43
1,10	MXRA7	matrix-remodelling associated 7
1,10	ERVFRD-1	endogenous retrovirus group FRD, member 1
1,10	TAF13	TAF13 RNA polymerase II, TATA box binding protein (TBP)-associated factor, 18kDa
1,10	SLC5A9	solute carrier family 5 (sodium/sugar cotransporter), member 9
1,10	FAM189A2	family with sequence similarity 189, member A2
1,10	DNAH8	dynein, axonemal, heavy chain 8
1,10	PSMD12	proteasome 26S subunit, non-ATPase 12
1,10	HSP90AA1	heat shock protein 90kDa alpha (cytosolic), class A member 1
1,10	RGL3	ral guanine nucleotide dissociation stimulator-like 3
1,10	DDI1	DNA-damage inducible 1 homolog 1
1,10	IL16	interleukin 16
1,10	FAM46B	family with sequence similarity 46, member B
1,10	RNASE12; RNASE11	ribonuclease, RNase A family, 12 (non-active); ribonuclease, RNase A family, 11 (non-active)
1,10	FAM83G	family with sequence similarity 83, member G
1,10	L1TD1	LINE-1 type transposase domain containing 1

1,10	GIN1	gypsy retrotransposon integrase 1
1,10	WDR20	WD repeat domain 20
1,10	DPH6	diphthamine biosynthesis 6
1,10	MX2	MX dynamin-like GTPase 2
1,10	ASH1L-AS1	ASH1L antisense RNA 1
1,09	LRRC6	leucine rich repeat containing 6
1,09	ADORA2B	adenosine A2b receptor
1,09	FAM160A2	family with sequence similarity 160, member A2
1,09	EFCAB1	EF-hand calcium binding domain 1
1,09	DHRS4	dehydrogenase/reductase (SDR family) member 4
1,09	ZNF816	zinc finger protein 816
1,09	ERICH6	glutamate rich 6
1,09	MATN4	matrilin 4
1,09	WT1	Wilms tumor 1
1,09	PRY; PRY2	PTPN13-like, Y-linked; PTPN13-like, Y-linked 2
1,09	SMIM2	small integral membrane protein 2
1,09	CNTN4	contactin 4
1,09	MBD1	methyl-CpG binding domain protein 1
1,09	COLQ	collagen-like tail subunit (single strand of homotrimer) of asymmetric acetylcholinesterase
1,09	SPIN2B	spindlin family, member 2B
1,09	RAC3	ras-related C3 botulinum toxin substrate 3 (rho family, small GTP binding protein Rac3)
1,09	PRELID3A	PRELI domain containing 3A
1,09	NIPAL4	NIPA-like domain containing 4
1,09	INHBB	inhibin beta B
1,09	C5orf28	chromosome 5 open reading frame 28
1,09	MACC1	metastasis associated in colon cancer 1
1,09	GATA3	GATA binding protein 3
1,09	SLC7A14	solute carrier family 7, member 14
1,09	C8orf74	chromosome 8 open reading frame 74
1,09	RP9	retinitis pigmentosa 9 (autosomal dominant)
1,09	MRPL2	mitochondrial ribosomal protein L2
1,09	DMGDH	dimethylglycine dehydrogenase
1,09	ZNF852	zinc finger protein 852
1,09	LIPA	lipase A, lysosomal acid, cholesterol esterase
1,09	CHRDL2	chordin-like 2
1,09	WRAP53	WD repeat containing, antisense to TP53
1,09	LY9	lymphocyte antigen 9
1,09	CPA6	carboxypeptidase A6
1,09	MBD3L3	methyl-CpG binding domain protein 3-like 3
1,09	UCMA	upper zone of growth plate and cartilage matrix associated
1,09	WFDC3	WAP four-disulfide core domain 3
1,09	OR2T6	olfactory receptor, family 2, subfamily T, member 6
1,09	DIRAS1	DIRAS family, GTP-binding RAS-like 1
1,09	VENTX	VENT homeobox
1,09	NOX5; SPESP1	NADPH oxidase, EF-hand calcium binding domain 5; sperm equatorial segment protein 1

1,09	NBPF6; NBPF5P	neuroblastoma breakpoint family, member 6; neuroblastoma breakpoint family, member 5, pseudogene
1,09	CFAP70	cilia and flagella associated protein 70
1,09	CCDC8	coiled-coil domain containing 8
1,09	NCAM2	neural cell adhesion molecule 2
1,09	SETMAR	SET domain and mariner transposase fusion gene
1,09	MFSD13A	major facilitator superfamily domain containing 13A
1,09	PCNXL4	pecanex-like 4 (Drosophila)
1,09	SLC13A5	solute carrier family 13 (sodium-dependent citrate transporter), member 5
1,09	LCE4A	late cornified envelope 4A
1,09	ELOF1	ELF1 homolog, elongation factor 1
1,09	SPIB	Spi-B transcription factor (Spi-1/PU.1 related)
1,09	SLC2A6	solute carrier family 2 (facilitated glucose transporter), member 6 [Source:HGNC Symbol;Acc:HGNC:11011]
1,09	ZBED3	zinc finger, BED-type containing 3
1,09	ZNF404	zinc finger protein 404
1,09	MYOZ2	myozenin 2
1,09	CTCFL	CCCTC-binding factor (zinc finger protein)-like
1,09	SCGB1C2	secretoglobin, family 1C, member 2
1,09	RPH3AL	rabphilin 3A-like (without C2 domains)
1,09	TRIB1	tribbles pseudokinase 1
1,09	RTCA	RNA 3-terminal phosphate cyclase
1,09	MT1G	metallothionein 1G
1,09	VPS37C	vacuolar protein sorting 37 homolog C (S. cerevisiae)
1,09	SPIRE1	spire-type actin nucleation factor 1
1,09	CCNI2	cyclin I family, member 2
1,09	RSRP1	arginine/serine-rich protein 1
1,09	SSBP4	single stranded DNA binding protein 4
1,09	PPP1R11	protein phosphatase 1, regulatory (inhibitor) subunit 11
1,09	IRF1	interferon regulatory factor 1
1,09	DRAM2	DNA-damage regulated autophagy modulator 2
1,09	CPA1	carboxypeptidase A1 (pancreatic)
1,09	NUP62; IL4I1	nucleoporin 62kDa; interleukin 4 induced 1
1,09	NUP37	nucleoporin 37kDa
1,09	GRIA2	glutamate receptor, ionotropic, AMPA 2
1,09	AGTR2	angiotensin II receptor, type 2
1,09	IMPG1	interphotoreceptor matrix proteoglycan 1
1,09	RBMS3	RNA binding motif, single stranded interacting protein 3
1,09	TBC1D17; MIR4750	TBC1 domain family, member 17; microRNA 4750
1,09	CCL21	chemokine (C-C motif) ligand 21
1,09	ZNF101	zinc finger protein 101
1,09	SPAG6	sperm associated antigen 6
1,09	SH3RF3	SH3 domain containing ring finger 3
1,09	SSTR5	somatostatin receptor 5

1,09	SH3BGRL2	SH3 domain binding glutamate-rich protein like 2
1,09	XKRX	X-linked Kx blood group related, X-linked
1,09	ATXN1L	ataxin 1-like
1,09	HIST1H1T	histone cluster 1, H1t
1,09	MRPL1	mitochondrial ribosomal protein L1
1,09	FAM3C	family with sequence similarity 3, member C
1,09	MT1H	metallothionein 1H
1,09	SLCO1B3	solute carrier organic anion transporter family, member 1B3
1,09	AC006128.2; AKAP8L	Memczak2013 ANTISENSE, coding, INTERNAL, intronic best transcript NM_014371; TEC
1,09	CYCS	cytochrome c, somatic
1,09	CACNA1F	calcium channel, voltage-dependent, L type, alpha 1F subunit
1,09	AGAP9; CTGLF11P	Salzman2013 ANNOTATED, CDS, coding, INTERNAL, OVCODE, OVEXON best transcript NM_001190810; centaurin, gamma-like family, member 11 pseudogene [Source:HGNC Symbol;Acc:HGNC:23660]
1,09	ZC2HC1C	zinc finger, C2HC-type containing 1C
1,09	ACIN1	apoptotic chromatin condensation inducer 1
1,09	MDP1; NEDD8-MDP1; NEDD8; CHMP4A	magnesium-dependent phosphatase 1; NEDD8-MDP1 readthrough; neural precursor cell expressed, developmentally down-regulated 8; charged multivesicular body protein 4A
1,09	BTBD1	BTB (POZ) domain containing 1
1,09	LOC100505478; RP11-402G3.3	uncharacterized LOC100505478; putative novel transcript
1,09	SYNGR4	synaptogyrin 4
1,09	HARBI1	harbinger transposase derived 1
1,09	KLRG2	killer cell lectin-like receptor subfamily G, member 2
1,09	RRP15	ribosomal RNA processing 15 homolog
1,09	FAM222A	family with sequence similarity 222, member A
1,09	FAM127B	family with sequence similarity 127, member B
1,09	KATNBL1	katanin p80 subunit B-like 1
1,09	IGFBP1	insulin like growth factor binding protein 1
1,09	IBA57	IBA57 homolog, iron-sulfur cluster assembly
1,09	KLF3	Kruppel-like factor 3 (basic)
1,09	CHP1	calcineurin-like EF-hand protein 1
1,09	ERMARD	ER membrane-associated RNA degradation
1,09	HLX	H2.0-like homeobox
1,09	FSD1	fibronectin type III and SPRY domain containing 1
1,09	FNBP4	formin binding protein 4
1,09	SLX1A; SLX1B; SLX1A-SULT1A3; SLX1B-SULT1A4; SULT1A3	SLX1 homolog A, structure-specific endonuclease subunit; SLX1 homolog B, structure-specific endonuclease subunit; SLX1A-SULT1A3 readthrough (NMD candidate); SLX1B-SULT1A4 readthrough (NMD candidate); sulfotransferase family 1A member 3
1,09	SPANXN2	SPANX family, member N2
1,09	STK4	serine/threonine kinase 4

1,09	UBE2S	Zhang2013 ALT_ACCEPTOR, ALT_DONOR, coding, INTERNAL, intronic, OVERLAPTX best transcript NM_014501
1,09	A2BP1	Transcript Identified by AceView, Entrez Gene ID(s) 54715
1,09	GRAP2	GRB2-related adaptor protein 2
1,09	AMY1A; AMY1C	amylase, alpha 1A (salivary); amylase, alpha 1C (salivary)
1,09	CCDC97	coiled-coil domain containing 97
1,09	TFF1	trefoil factor 1
1,09	KIF1BP	KIF1 binding protein
1,09	RNF152	ring finger protein 152
1,09	TAAR6	trace amine associated receptor 6
1,09	PLEK2	pleckstrin 2
1,09	SKA1	spindle and kinetochore associated complex subunit 1
1,09	GDF15	growth differentiation factor 15
1,09	KCTD21	potassium channel tetramerization domain containing 21
1,09	CDC7	cell division cycle 7
1,09	SIRPA	signal-regulatory protein alpha
1,09	SYT5	synaptotagmin V
1,09	PDCL2	phosducin like 2
1,09	GTF2F2	general transcription factor IIF subunit 2
1,09	COPG2; TSGA13	coatomer protein complex subunit gamma 2; testis specific 13
1,09	PNPLA5	patatin-like phospholipase domain containing 5
1,09	SLC25A12	solute carrier family 25 (aspartate/glutamate carrier), member 12
1,09	TMEM139	transmembrane protein 139
1,09	BUB1	BUB1 mitotic checkpoint serine/threonine kinase
1,09	GNAT2	guanine nucleotide binding protein (G protein), alpha transducing activity polypeptide 2
1,09	SORBS1	sorbin and SH3 domain containing 1
1,09	CILP	cartilage intermediate layer protein, nucleotide pyrophosphohydrolase
1,09	C5orf60	chromosome 5 open reading frame 60
1,09	TULP2	tubby like protein 2
1,09	B3GALNT1	beta-1,3-N-acetylgalactosaminyltransferase 1 (globoside blood group)
1,09	FOXC2	forkhead box C2
1,09	USP9Y; TTTY15	ubiquitin specific peptidase 9, Y-linked; testis-specific transcript, Y-linked 15 (non-protein coding)
1,09	KRTAP21-1	keratin associated protein 21-1
1,09	ZFP28	ZFP28 zinc finger protein
1,09	ADAMTS13	ADAM metallopeptidase with thrombospondin type 1 motif 13
1,09	HNRNPA1L2	heterogeneous nuclear ribonucleoprotein A1-like 2
1,09	ATP5J2-PTCD1; PTCD1	ATP5J2-PTCD1 readthrough; pentatricopeptide repeat domain 1
1,09	IGF2BP3	insulin-like growth factor 2 mRNA binding protein 3
1,09	IQCK	IQ motif containing K

1,09	ETHE1	ethylmalonic encephalopathy 1
1,09	DPH3	diphthamide biosynthesis 3
1,09	GLDN	gliomedin
1,09	TMCO4	transmembrane and coiled-coil domains 4
1,09	DNAJB4	DnaJ (Hsp40) homolog, subfamily B, member 4
1,09	PAGE4	P antigen family, member 4 (prostate associated)
1,09	MCCD1	mitochondrial coiled-coil domain 1
1,09	CLCN1	chloride channel, voltage-sensitive 1
1,09	CTNS	cystinosin, lysosomal cystine transporter
1,09	DNAJC25	DnaJ (Hsp40) homolog, subfamily C , member 25
1,09	TRIM37	Transcript Identified by AceView, Entrez Gene ID(s) 4591
1,09	IFITM5	interferon induced transmembrane protein 5
1,09	VNN2	vanin 2
1,09	KCNN3	potassium channel, calcium activated intermediate/small conductance subfamily N alpha, member 3
1,09	ALX3	ALX homeobox 3
1,09	TCEANC	transcription elongation factor A (SII) N-terminal and central domain containing
1,09	ZFAND2A	zinc finger, AN1-type domain 2A
1,09	TRAPPC4	trafficking protein particle complex 4
1,09	HTR1D	5-hydroxytryptamine (serotonin) receptor 1D, G protein-coupled
1,09	KCNJ8	potassium channel, inwardly rectifying subfamily J, member 8
1,09	OR13F1	olfactory receptor, family 13, subfamily F, member 1
1,09	TPPP	tubulin polymerization promoting protein
1,09	RPEL1	ribulose-5-phosphate-3-epimerase-like 1
1,09	SPANXD	SPANX family, member D
1,09	ALG13	ALG13, UDP-N-acetylglucosaminyltransferase subunit
1,09	USP50	ubiquitin specific peptidase 50
1,09	PCSK2	proprotein convertase subtilisin/kexin type 2
1,09	UBE2V1	ubiquitin conjugating enzyme E2 variant 1
1,09	PRAMEF14	PRAME family member 14
1,09	DCST1	DC-STAMP domain containing 1
1,09	FAM86B2	family with sequence similarity 86, member B2
1,09	HOXB3; HOXB4; MIR10A	homeobox B3; homeobox B4; microRNA 10a
1,09	VAV3	vav 3 guanine nucleotide exchange factor
1,09	LOC101927550; AC004893.11; SMURF1	uncharacterized LOC101927550; Salzman2013 ANTISENSE, CDS, coding, INTERNAL, intronic, OVCODE, OVERLAPTX, OVEXON best transcript NM_020429; novel transcript
1,09	MLN	motilin
1,09	TOX2	TOX high mobility group box family member 2

1,09	SELM	selenoprotein M; selenoprotein M [Source:EntrezGene;Acc:140606]; Salzman2013 ANNOTATED, CDS, coding, OVCODE, OVERLAPTX, OVEXON, UTR3 best transcript NM_080430; Salzman2013 ANNOTATED, CDS, coding, OVCODE, OVERLAPTX, OVEXON, UTR3, UTR5 best transcript NM_080430; Transcript Identified by AceView, Entrez Gene ID(s) 140606
1,09	SLC2A6	solute carrier family 2 (facilitated glucose transporter), member 6 [Source:HGNC Symbol;Acc:HGNC:11011]
1,09	TMEM169	transmembrane protein 169
1,09	LINC00521	long intergenic non-protein coding RNA 521
1,09	UCP1	uncoupling protein 1 (mitochondrial, proton carrier)
1,09	PDE10A	phosphodiesterase 10A
1,09	SLC9C2	solute carrier family 9, member C2 (putative)
1,09	TRIM64	tripartite motif containing 64
1,09	ENAM	enamelin
1,09	LDAH	lipid droplet associated hydrolase
1,09	STT3A	STT3A, subunit of the oligosaccharyltransferase complex (catalytic)
1,09	WDR37	WD repeat domain 37
1,09	STRC	stereocilin
1,09	KLRC1	killer cell lectin-like receptor subfamily C, member 1
1,09	JAKMIP3	Janus kinase and microtubule interacting protein 3
1,09	COX4I2	cytochrome c oxidase subunit IV isoform 2 (lung)
1,09	ADM5; CPT1C	adrenomedullin 5 (putative); carnitine palmitoyltransferase 1C
1,09	MYO5B	myosin VB
1,09	ZCCHC5	zinc finger, CCHC domain containing 5
1,09	C9orf172	chromosome 9 open reading frame 172
1,09	MTMR8	myotubularin related protein 8
1,09	TOM1L1	target of myb1 like 1 membrane trafficking protein
1,09	TP53I11	Memczak2013 ALT_ACCEPTOR, ALT_DONOR, coding, INTERNAL, intronic best transcript NM_001076787
1,09	C2orf72	chromosome 2 open reading frame 72
1,09	ELP6	elongator acetyltransferase complex subunit 6
1,09	C19orf60	chromosome 19 open reading frame 60
1,09	MIEF2	mitochondrial elongation factor 2
1,09	GLT1D1	glycosyltransferase 1 domain containing 1
1,09	CSMD1	CUB and Sushi multiple domains 1
1,09	ZNF692	zinc finger protein 692
1,09	FMN1	formin 1
1,09	MAP7D2	MAP7 domain containing 2
1,09	PBX1	pre-B-cell leukemia homeobox 1
1,09	FHIT	fragile histidine triad
1,09	RAB40C	RAB40C, member RAS oncogene family
1,09	GFRA3	GDNF family receptor alpha 3
1,09	LMX1A	LIM homeobox transcription factor 1, alpha

1,09	CHCHD1	coiled-coil-helix-coiled-coil-helix domain containing 1
1,09	ARHGAP1	Rho GTPase activating protein 1
1,09	RND2	Rho family GTPase 2
1,09	TRMT6	tRNA methyltransferase 6
1,09	ZBBX	zinc finger, B-box domain containing
1,09	GYS2	glycogen synthase 2 (liver)
1,09	ZNF385D	zinc finger protein 385D
1,09	CLDN4	claudin 4
1,09	COG6	component of oligomeric golgi complex 6
1,09	ESF1	ESF1 nucleolar pre-rRNA processing protein homolog
1,09	FGF8	fibroblast growth factor 8 (androgen-induced)
1,09	NELFA; MIR943	negative elongation factor complex member A; microRNA 943
1,09	CCL16	chemokine (C-C motif) ligand 16
1,09	ZNRF4	zinc and ring finger 4
1,09	NAP1L2	nucleosome assembly protein 1-like 2
1,09	RASGRP3	RAS guanyl releasing protein 3 (calcium and DAG-regulated)
1,09	CCDC107	coiled-coil domain containing 107
1,09	NXPE1	neurexophilin and PC-esterase domain family, member 1
1,09	DRD5	dopamine receptor D5
1,09	ELOVL6	ELOVL fatty acid elongase 6
1,09	TGIF2	TGFB-induced factor homeobox 2
1,09	NUDT15	nudix hydrolase 15
1,09	TIMM22	translocase of inner mitochondrial membrane 22 homolog (yeast)
1,09	TUSC5	tumor suppressor candidate 5
1,09	OR51I1	olfactory receptor, family 51, subfamily I, member 1
1,09	BZRAP1	benzodiazepine receptor (peripheral) associated protein 1
1,09	SC5D	sterol-C5-desaturase
1,09	GPC4	glypican 4
1,09	EPM2AIP1	EPM2A (laforin) interacting protein 1
1,09	PDGFD	platelet derived growth factor D
1,09	PIK3R5	phosphoinositide-3-kinase, regulatory subunit 5
1,09	FAM25C	family with sequence similarity 25, member C
1,09	GATSL2	GATS protein-like 2
1,09	PRAMEF8	PRAME family member 8
1,09	SIX2	SIX homeobox 2
1,09	ZNF195	zinc finger protein 195
1,09	EBNA1BP2	EBNA1 binding protein 2
1,09	HOXA-AS3	HOXA cluster antisense RNA 3
1,09	SURF4	surfeit 4
1,09	PRNP	prion protein
1,09	GJD3	gap junction protein delta 3
1,09	CYB5R2	cytochrome b5 reductase 2
1,09	HPR	haptoglobin-related protein

1,09	PUSL1	pseudouridylate synthase-like 1
1,09	CUL5	cullin 5
1,09	OR4M2	olfactory receptor, family 4, subfamily M, member 2
1,09	MAP4	Transcript Identified by AceView, Entrez Gene ID(s) 4134
1,09	PRDM7	PR domain containing 7
1,09	LCORL	ligand dependent nuclear receptor corepressor like
1,09	KLF4	Kruppel-like factor 4 (gut)
1,09	SLC44A2	solute carrier family 44 (choline transporter), member 2
1,09	ZNF789	zinc finger protein 789
1,09	ZNF678	zinc finger protein 678
1,09	OR1J1	olfactory receptor, family 1, subfamily J, member 1
1,09	SETBP1	Memczak2013 ALT_ACCEPTOR, ALT_DONOR, coding, INTERNAL, intronic best transcript NM_001130110
1,09	NTRK3	neurotrophic tyrosine kinase, receptor, type 3
1,09	MIR5195; IGHV5-78	microRNA 5195; immunoglobulin heavy variable 5-78 (pseudogene)
1,09	RSPO2	R-spondin 2
1,09	PXN	paxillin
1,09	BARX1	BARX homeobox 1
1,09	SMYD3	Jeck2013 ALT_ACCEPTOR, ALT_DONOR, coding, INTERNAL, intronic best transcript NM_001167740
1,09	FEZ2	fasciculation and elongation protein zeta 2 (zygin II)
1,09	ARRB2	arrestin, beta 2
1,09	ADRA1A	adrenoceptor alpha 1A
1,09	CREBL2	cAMP responsive element binding protein-like 2
1,09	PNLIPRP3	pancreatic lipase-related protein 3
1,09	ETV4	ets variant 4
1,09	UACA	uveal autoantigen with coiled-coil domains and ankyrin repeats
1,09	CDC42SE2	CDC42 small effector 2
1,09	FAM102A	Memczak2013 ANTISENSE, CDS, coding, INTERNAL, intronic, UTR3 best transcript NM_203305
1,09	C12orf45	chromosome 12 open reading frame 45
1,09	ABI3	ABI family, member 3
1,09	FBXO40	F-box protein 40
1,09	PLA2G2A	phospholipase A2, group IIA (platelets, synovial fluid)
1,09	FAM184A	family with sequence similarity 184, member A
1,09	FAM13B	family with sequence similarity 13, member B
1,09	KRT73	keratin 73, type II
1,09	GABRE; MIR224; MIR452	gamma-aminobutyric acid (GABA) A receptor, epsilon; microRNA 224; microRNA 452
1,09	H2AFB2	H2A histone family, member B2
1,09	RANBP10	RAN binding protein 10
1,09	CDKAL1	CDK5 regulatory subunit associated protein 1-like 1
1,09	GCNT3	glucosaminyl (N-acetyl) transferase 3, mucin type

1,09	HLA-DRB1	major histocompatibility complex, class II, DR beta 1
1,09	TMEM120B	transmembrane protein 120B
1,09	SLC9A3R2	solute carrier family 9, subfamily A (NHE3, cation proton antiporter 3), member 3 regulator 2
1,09	BSDC1	BSD domain containing 1
1,09	NUP214	nucleoporin 214kDa
1,09	ZDHHC9	zinc finger, DHHC-type containing 9
1,09	USP6	ubiquitin specific peptidase 6
1,09	PUDP	pseudouridine 5-phosphatase
1,09	PVRL2	poliovirus receptor-related 2 (herpesvirus entry mediator B)
1,09	ANKRD1	ankyrin repeat domain 1 (cardiac muscle)
1,09	DAXX	death-domain associated protein
1,09	TVP23B	trans-golgi network vesicle protein 23 homolog B (S. cerevisiae)
1,09	KRTAP20-3	keratin associated protein 20-3
1,09	TFG	TRK-fused gene
1,09	HECW1	HECT, C2 and WW domain containing E3 ubiquitin protein ligase 1
1,09	DGCR2; DGCR11	DiGeorge syndrome critical region gene 2; DiGeorge syndrome critical region gene 11 (non-protein coding)
1,09	CCDC116	coiled-coil domain containing 116
1,09	TMEM17	transmembrane protein 17
1,09	TMEM119	transmembrane protein 119
1,09	PLCL1	phospholipase C-like 1
1,09	OR8D1	olfactory receptor, family 8, subfamily D, member 1
1,09	TMEM126A	transmembrane protein 126A
1,09	C9orf64	Transcript Identified by AceView, Entrez Gene ID(s) 84267
1,09	CARD14	caspase recruitment domain family, member 14
1,09	YPEL2	yippee like 2
1,09	FAM98B	family with sequence similarity 98, member B
1,09	SLC25A31	solute carrier family 25 (mitochondrial carrier; adenine nucleotide translocator), member 31
1,09	AMER3	APC membrane recruitment protein 3
1,09	KCTD6	potassium channel tetramerization domain containing 6
1,09	TPTE	transmembrane phosphatase with tensin homology
1,09	HTR2B	5-hydroxytryptamine (serotonin) receptor 2B, G protein-coupled
1,09	NDC1	NDC1 transmembrane nucleoporin
1,09	HIBADH	Salzman2013 ALT_ACCEPTOR, ALT_DONOR, coding, INTERNAL, intronic best transcript NM_152740
1,09	PSMB3	proteasome subunit beta 3
1,09	NSUN7	NOP2/Sun domain family, member 7
1,09	KLKB1	kallikrein B1
1,09	WFDC6	WAP four-disulfide core domain 6
1,09	SETD7	SET domain containing (lysine methyltransferase) 7

1,09	SLC16A1	solute carrier family 16 (monocarboxylate transporter), member 1
1,09	ANKRD37	ankyrin repeat domain 37
1,09	FAM171A1	family with sequence similarity 171, member A1
1,09	GINM1	glycoprotein integral membrane 1
1,09	HS3ST4	heparan sulfate (glucosamine) 3-O-sulfotransferase 4
1,09	DMRTA1	DMRT-like family A1
1,09	PCDH17	protocadherin 17
1,09	ALDH1A3	aldehyde dehydrogenase 1 family, member A3
1,09	SLC6A13	solute carrier family 6 (neurotransmitter transporter), member 13
1,09	EML2; MIR330	echinoderm microtubule associated protein like 2; microRNA 330
1,09	ASB6	ankyrin repeat and SOCS box containing 6
1,09	SIGLEC12	sialic acid binding Ig-like lectin 12 (gene/pseudogene)
1,09	USP8	ubiquitin specific peptidase 8
1,09	LAIR2	leukocyte-associated immunoglobulin-like receptor 2
1,09	SMO	smoothened, frizzled class receptor
1,09	TRIML1	tripartite motif family like 1
1,09	CALB2	calbindin 2
1,09	ZNF219	zinc finger protein 219
1,09	NLRP14	NLR family, pyrin domain containing 14
1,09	CYP11A1	cytochrome P450, family 11, subfamily A, polypeptide 1
1,09	DCAF5	DDB1 and CUL4 associated factor 5
1,09	PSRC1	proline/serine-rich coiled-coil 1
1,09	PGAP2	post-GPI attachment to proteins 2
1,09	HPS4	Hermansky-Pudlak syndrome 4
1,09	RNLS	renalase, FAD-dependent amine oxidase
1,09	DUSP5	dual specificity phosphatase 5
1,09	NXPE3	neurexophilin and PC-esterase domain family, member 3
1,09	APOF	apolipoprotein F
1,09	GNGT1	guanine nucleotide binding protein (G protein), gamma transducing activity polypeptide 1
1,09	TRAPPC1	trafficking protein particle complex 1
1,09	LOC149950; RP11-410N8.4	uncharacterized LOC149950; novel transcript
1,09	TMED8	transmembrane p24 trafficking protein family member 8
1,09	ITPK1	inositol-tetrakisphosphate 1-kinase
1,09	C10orf53	chromosome 10 open reading frame 53
1,09	ACP2	acid phosphatase 2, lysosomal
1,09	GPR137B	G protein-coupled receptor 137B
1,09	ARHGAP18	Rho GTPase activating protein 18
1,09	OTUD6A	OTU deubiquitinase 6A
1,09	HSDL1	hydroxysteroid dehydrogenase like 1
1,09	CUL4A	cullin 4A
1,09	SCRG1	stimulator of chondrogenesis 1
1,09	MYOZ3	myozenin 3

1,09	SLC30A8	solute carrier family 30 (zinc transporter), member 8
1,09	GAD2	glutamate decarboxylase 2
1,09	USP21	ubiquitin specific peptidase 21
1,09	FOXA3	forkhead box A3
1,09	WASF2	WAS protein family, member 2
1,09	SAMD8	sterile alpha motif domain containing 8
1,09	UGT2B15	UDP glucuronosyltransferase 2 family, polypeptide B15
1,09	C10orf35	chromosome 10 open reading frame 35
1,09	RABGGTB; SNORD45B; SNORD45A; SNORD45C	Rab geranylgeranyltransferase, beta subunit; small nucleolar RNA, C/D box 45B; small nucleolar RNA, C/D box 45A; small nucleolar RNA, C/D box 45C
1,09	PLEKHG4B	pleckstrin homology domain containing, family G (with RhoGef domain) member 4B
1,09	ZNF502	zinc finger protein 502
1,09	ADIPOR1	adiponectin receptor 1
1,09	COTL1	coactosin-like F-actin binding protein 1
1,09	ANGPT1	angiopoietin 1
1,09	PIGG	phosphatidylinositol glycan anchor biosynthesis class G
1,09	DAZL	deleted in azoospermia-like
1,09	OR5K3	olfactory receptor, family 5, subfamily K, member 3
1,09	ZNF675	zinc finger protein 675
1,09	ZFP1	ZFP1 zinc finger protein
1,09	CAP1	CAP, adenylate cyclase-associated protein 1 (yeast)
1,09	ABCB4	ATP binding cassette subfamily B member 4
1,09	ZNF197	zinc finger protein 197
1,09	PHF3	PHD finger protein 3
1,09	PBOV1	prostate and breast cancer overexpressed 1
1,09	TMEM92	transmembrane protein 92
1,09	KCNN1	potassium channel, calcium activated intermediate/small conductance subfamily N alpha, member 1
1,09	PIWIL3	piwi-like RNA-mediated gene silencing 3
1,09	ZKSCAN3	zinc finger with KRAB and SCAN domains 3
1,09	PPP6C	protein phosphatase 6, catalytic subunit
1,09	LYVE1	lymphatic vessel endothelial hyaluronan receptor 1
1,09	GPBP1L1	GC-rich promoter binding protein 1-like 1
1,09	COX10	COX10 heme A:farnesyltransferase cytochrome c oxidase assembly factor
1,09	MYO1B	myosin IB
1,09	ACTR8	ARP8 actin-related protein 8 homolog (yeast)
1,09	ZNF630	zinc finger protein 630
1,09	MPV17L	MPV17 mitochondrial membrane protein-like
1,09	GJB1	gap junction protein beta 1
1,09	NUDT22	nudix hydrolase 22
1,09	CFAP58	cilia and flagella associated protein 58
1,09	NOG	noggin
1,09	NT5C2	5-nucleotidase, cytosolic II

1,09	IFIT3	interferon-induced protein with tetratricopeptide repeats 3
1,09	ORC4	origin recognition complex subunit 4
1,09	C6orf47	chromosome 6 open reading frame 47
1,09	ARSK	arylsulfatase family, member K
1,09	AP3B2	adaptor-related protein complex 3, beta 2 subunit
1,09	LPAR6	lysophosphatidic acid receptor 6
1,09	FAM43B	family with sequence similarity 43, member B
1,09	SFT2D3	SFT2 domain containing 3
1,09	TMEM87B	transmembrane protein 87B
1,09	ADAM29	ADAM metallopeptidase domain 29
1,09	CALY	calcyon neuron-specific vesicular protein
1,09	H3F3C	H3 histone, family 3C
1,09	DCBLD1	discoidin, CUB and LCCL domain containing 1
1,09	CLNK	cytokine-dependent hematopoietic cell linker
1,09	HEATR9	HEAT repeat containing 9
1,09	CYS1	cystin 1
1,09	JADE2	jade family PHD finger 2
1,09	RNF31	ring finger protein 31
1,09	CCNB3	cyclin B3
1,09	NAAA	N-acylethanolamine acid amidase
1,09	CEND1	cell cycle exit and neuronal differentiation 1
1,09	ATP5G2	ATP synthase, H+ transporting, mitochondrial Fo complex subunit C2 (subunit 9)
1,09	TNIK	TRAF2 and NCK interacting kinase
1,09	MGST1	microsomal glutathione S-transferase 1
1,09	NFKB2	nuclear factor of kappa light polypeptide gene enhancer in B-cells 2 (p49/p100)
1,09	ZNF132	zinc finger protein 132
1,09	ENOX2	ecto-NOX disulfide-thiol exchanger 2
1,09	EYS	eyes shut homolog (Drosophila)
1,09	TCHH	trichohyalin
1,09	RALGPS2	Ral GEF with PH domain and SH3 binding motif 2
1,09	KEL	Kell blood group, metallo-endopeptidase
1,09	CEACAM21	carcinoembryonic antigen-related cell adhesion molecule 21
1,09	CYP11B2	cytochrome P450, family 11, subfamily B, polypeptide 2
1,09	LRRN3	leucine rich repeat neuronal 3
1,09	FOLR3	folate receptor 3 (gamma)
1,09	DCX	doublecortin
1,09	TRPC5OS	TRPC5 opposite strand
1,09	PYGM	phosphorylase, glycogen, muscle
1,09	SESTD1	SEC14 and spectrin domains 1
1,09	TADA2A	transcriptional adaptor 2A
1,09	SLC35F1	solute carrier family 35, member F1
1,09	TOM1L2	target of myb1 like 2 membrane trafficking protein
1,09	MURC	muscle-related coiled-coil protein
1,09	MARCO	macrophage receptor with collagenous structure
1,09	FOLR2	folate receptor 2 (fetal)

1,09	IRS4	insulin receptor substrate 4
1,09	TMEM211	transmembrane protein 211
1,09	NUDT10	nudix hydrolase 10
1,09	INPP5D	inositol polyphosphate-5-phosphatase D
1,09	TBCK	Memczak2013 ALT_ACCEPTOR, ALT_DONOR, coding, INTERNAL, intronic best transcript NM_001163435
1,09	DTWD1	DTW domain containing 1
1,09	SPATA31A6	SPATA31 subfamily A, member 6
1,09	TCEAL7	transcription elongation factor A (SII)-like 7
1,09	PKN2	protein kinase N2
1,09	EFHD2	EF-hand domain family member D2
1,09	BMS1	BMS1 ribosome biogenesis factor
1,09	CLDN19	claudin 19
1,09	AMPD3	adenosine monophosphate deaminase 3
1,09	PLEKHG4	pleckstrin homology domain containing, family G (with RhoGef domain) member 4
1,09	TUBB	tubulin, beta class I
1,09	LOC400661; AC034110.1	uncharacterized LOC400661; novel transcript
1,09	TXNRD3NB; TXNRD3	thioredoxin reductase 3 neighbor; thioredoxin reductase 3
1,09	ALOX15B	arachidonate 15-lipoxygenase, type B
1,09	CHRNG	cholinergic receptor, nicotinic gamma
1,09	HDAC9	Jeck2013 ALT_ACCEPTOR, ALT_DONOR, coding, INTERNAL, intronic best transcript NM_178423
1,09	TFB1M	transcription factor B1, mitochondrial
1,09	PDCD6IP	programmed cell death 6 interacting protein
1,09	CDC42EP1	CDC42 effector protein (Rho GTPase binding) 1
1,09	EXOSC10	exosome component 10
1,09	TMEM98	transmembrane protein 98
1,09	SSPO	SCO-spondin
1,09	TRPM2	transient receptor potential cation channel, subfamily M, member 2
1,09	ENO1	Memczak2013 ANTISENSE, coding, INTERNAL, UTR3 best transcript NM_001428
1,09	SLC38A6	solute carrier family 38, member 6
1,09	DESI1	desumoylating isopeptidase 1
1,09	OR6C74	olfactory receptor, family 6, subfamily C, member 74
1,09	PRR32	proline rich 32
1,09	ABHD3	Transcript Identified by AceView, Entrez Gene ID(s) 171586
1,09	USP17L26; USP17L24	ubiquitin specific peptidase 17-like family member 26; ubiquitin specific peptidase 17-like family member 24
1,09	SULT1C3	sulfotransferase family 1C member 3
1,09	MT1IP	metallothionein 1I, pseudogene
1,09	ZNF502	zinc finger protein 502
1,09	ZNF26	zinc finger protein 26
1,09	SUV39H1	suppressor of variegation 3-9 homolog 1 (Drosophila)

1,09	PFKFB3	6-phosphofructo-2-kinase/fructose-2,6-biphosphatase 3
1,08	POU6F2-AS2; POU6F2; AC011292.2	POU6F2 antisense RNA 2 [Source:HGNC Symbol;Acc:HGNC:21887]; Salzman2013 ANTISENSE, coding, INTERNAL, intronic, OVERLAPTX best transcript NM_007252; novel transcript, antisense to POU6F2
1,08	NUDT18	nudix hydrolase 18
1,08	SLC38A4	solute carrier family 38, member 4
1,08	TMEM161A	transmembrane protein 161A
1,08	RAI1	retinoic acid induced 1
1,08	RNASE10	ribonuclease, RNase A family, 10 (non-active)
1,08	POGZ	Transcript Identified by AceView, Entrez Gene ID(s) 23126
1,08	ATF6B	activating transcription factor 6 beta
1,08	XRCC2	X-ray repair complementing defective repair in Chinese hamster cells 2
1,08	REPS1	RALBP1 associated Eps domain containing 1
1,08	DACH2	dachshund family transcription factor 2
1,08	CDK12	cyclin-dependent kinase 12
1,08	CXorf57	chromosome X open reading frame 57
1,08	HHAT	hedgehog acyltransferase
1,08	PRR15	proline rich 15
1,08	LALBA	lactalbumin, alpha-
1,08	LILRB5	leukocyte immunoglobulin-like receptor, subfamily B (with TM and ITIM domains), member 5
1,08	GP5	glycoprotein V (platelet)
1,08	VAMP7	Transcript Identified by AceView, Entrez Gene ID(s) 6845
1,08	PNPLA6	patatin-like phospholipase domain containing 6
1,08	C19orf53	chromosome 19 open reading frame 53
1,08	RPS26	Homo sapiens ribosomal protein S26, mRNA (cDNA clone MGC:27148 IMAGE:4807004), complete cds.; Homo sapiens ribosomal protein S26, mRNA (cDNA clone MGC:88201 IMAGE:6388128), complete cds.; Homo sapiens ribosomal protein S26, mRNA (cDNA clone MGC:104291 IMAGE:4287636), complete cds.; Homo sapiens ribosomal protein S26, mRNA (cDNA clone MGC:104292 IMAGE:6726218), complete cds.
1,08	OR11A1	olfactory receptor, family 11, subfamily A, member 1
1,08	ATG13	autophagy related 13
1,08	SLC35F6	solute carrier family 35, member F6
1,08	MYH7	myosin, heavy chain 7, cardiac muscle, beta
1,08	HACD3	3-hydroxyacyl-CoA dehydratase 3
1,08	PLPP3	phospholipid phosphatase 3
1,08	CXCL16	chemokine (C-X-C motif) ligand 16
1,08	NCR2	natural cytotoxicity triggering receptor 2
1,08	ADRM1	adhesion regulating molecule 1
1,08	TIGD1; MIR5001	tigger transposable element derived 1; microRNA 5001

1,08	CELF6	CUGBP, Elav-like family member 6
1,08	TWIST1	twist family bHLH transcription factor 1
1,08	CDK11B	cyclin-dependent kinase 11B
1,08	NPHS1	nephrosis 1, congenital, Finnish type (nephrin)
1,08	KRT1	keratin 1, type II
1,08	RPL3L	ribosomal protein L3-like
1,08	NUDT11	nudix hydrolase 11
1,08	KCNMB3	potassium channel subfamily M regulatory beta subunit 3
1,08	PNPT1	polyribonucleotide nucleotidyltransferase 1
1,08	OPN5	opsin 5
1,08	SPDYE2; SPDYE2B	speedy/RINGO cell cycle regulator family member E2; speedy/RINGO cell cycle regulator family member E2B
1,08	SPDYE2B	speedy/RINGO cell cycle regulator family member E2B
1,08	FNDC9	fibronectin type III domain containing 9
1,08	TRAT1	T cell receptor associated transmembrane adaptor 1
1,08	DNAJC5G	DnaJ (Hsp40) homolog, subfamily C, member 5 gamma
1,08	TRIM17	tripartite motif containing 17
1,08	FUOM	fucose mutarotase
1,08	GARS	glycyl-tRNA synthetase
1,08	DDX60	DEAD (Asp-Glu-Ala-Asp) box polypeptide 60
1,08	RP11-307P5.1; SAMD5	novel transcript; Transcript Identified by AceView, Entrez Gene ID(s) 389432
1,08	KCNH6	potassium channel, voltage gated eag related subfamily H, member 6
1,08	RRP12	ribosomal RNA processing 12 homolog
1,08	FBXO28	F-box protein 28
1,08	IL32	interleukin 32
1,08	ZNF776	zinc finger protein 776
1,08	ZG16	zymogen granule protein 16
1,08	SENP1	SUMO1/sentrin specific peptidase 1
1,08	WNT2	wingless-type MMTV integration site family member 2
1,08	GPR182	G protein-coupled receptor 182
1,08	KIF7	kinesin family member 7
1,08	ZNF705E	zinc finger protein 705E
1,08	PLA2G6	phospholipase A2, group VI (cytosolic, calcium-independent)
1,08	FAM195A	family with sequence similarity 195, member A
1,08	ZNF121	zinc finger protein 121
1,08	C9orf16	chromosome 9 open reading frame 16
1,08	CPXM2	carboxypeptidase X (M14 family), member 2
1,08	GOLGA6B	golgin A6 family, member B
1,08	SGMS1	sphingomyelin synthase 1
1,08	CCDC184	coiled-coil domain containing 184
1,08	H6PD	hexose-6-phosphate dehydrogenase (glucose 1-dehydrogenase)

1,08	GPR22	G protein-coupled receptor 22
1,08	RGPD2; RGPD1	RANBP2-like and GRIP domain containing 2; RANBP2-like and GRIP domain containing 1
1,08	REG4	regenerating islet-derived family, member 4
1,08	S100Z	S100 calcium binding protein Z
1,08	SNX5	sorting nexin 5
1,08	CHST11	Transcript Identified by AceView, Entrez Gene ID(s) 50515
1,08	RGS13	regulator of G-protein signaling 13
1,08	ANKRA2	ankyrin repeat, family A (RFXANK-like), 2
1,08	CCDC122	coiled-coil domain containing 122
1,08	UGT2B10	UDP glucuronosyltransferase 2 family, polypeptide B10
1,08	STK26	serine/threonine protein kinase 26
1,08	PRG1	p53-responsive gene 1
1,08	CST3	cystatin C
1,08	MYO6	Transcript Identified by AceView, Entrez Gene ID(s) 4646
1,08	CHST12	carbohydrate (chondroitin 4) sulfotransferase 12
1,08	DCAF12L1	DDB1 and CUL4 associated factor 12-like 1
1,08	BBS4	Memczak2013 ALT_ACCEPTOR, ALT_DONOR, coding, INTERNAL, intronic best transcript NM_033028
1,08	LRRC28	Transcript Identified by AceView, Entrez Gene ID(s) 123355
1,08	TUBE1	tubulin, epsilon 1
1,08	NCOA7	nuclear receptor coactivator 7
1,08	PNLIPRP2	pancreatic lipase-related protein 2
1,08	FARSB	phenylalanyl-tRNA synthetase beta subunit
1,08	TM4SF5	transmembrane 4 L six family member 5
1,08	SLC37A1	solute carrier family 37 (glucose-6-phosphate transporter), member 1
1,08	PIK3CD	phosphatidylinositol-4,5-bisphosphate 3-kinase, catalytic subunit delta
1,08	CXCL14	chemokine (C-X-C motif) ligand 14
1,08	MAP3K19	mitogen-activated protein kinase kinase kinase 19
1,08	MBD6	methyl-CpG binding domain protein 6
1,08	PCDH1	protocadherin 1
1,08	SRRM1	serine/arginine repetitive matrix 1
1,08	GRHL2	grainyhead-like transcription factor 2
1,08	PTGIR	prostaglandin I2 (prostacyclin) receptor (IP)
1,08	ZNF382	zinc finger protein 382
1,08	USP46	ubiquitin specific peptidase 46
1,08	ASB5	ankyrin repeat and SOCS box containing 5
1,08	NR1H3	nuclear receptor subfamily 1, group H, member 3
1,08	C11orf74	chromosome 11 open reading frame 74
1,08	CXorf38	chromosome X open reading frame 38
1,08	C8orf48	chromosome 8 open reading frame 48
1,08	RCVRN	recoverin
1,08	ZBTB17	zinc finger and BTB domain containing 17

1,08	ZNF589	zinc finger protein 589
1,08	OR4C13	olfactory receptor, family 4, subfamily C, member 13
1,08	DUSP27	dual specificity phosphatase 27 (putative)
1,08	HMBS	hydroxymethylbilane synthase
1,08	DPF3	D4, zinc and double PHD fingers, family 3
1,08	KIAA1919	KIAA1919
1,08	SEH1L	SEH1-like nucleoporin
1,08	OR6C65	olfactory receptor, family 6, subfamily C, member 65
1,08	C9orf170	chromosome 9 open reading frame 170
1,08	CCL5	chemokine (C-C motif) ligand 5
1,08	AMD1	adenosylmethionine decarboxylase 1
1,08	TMEM70; RPS20P21	transmembrane protein 70; ribosomal protein S20 pseudogene 21
1,08	TLX1NB	TLX1 neighbor
1,08	PRKAG3	protein kinase, AMP-activated, gamma 3 non-catalytic subunit
1,08	NTN1	netrin 1
1,08	GPR83	G protein-coupled receptor 83
1,08	AKR1C3	aldo-keto reductase family 1, member C3
1,08	ZNF160	Transcript Identified by AceView, Entrez Gene ID(s) 90338
1,08	KRTAP21-3	keratin associated protein 21-3
1,08	MKRN2OS	MKRN2 opposite strand
1,08	GLTPD2	glycolipid transfer protein domain containing 2
1,08	CLASP2	Transcript Identified by AceView, Entrez Gene ID(s) 23122
1,08	RAD51AP2	RAD51 associated protein 2
1,08	KREMEN1	kringle containing transmembrane protein 1
1,08	ATG16L1	autophagy related 16-like 1
1,08	BOLA1	bolA family member 1
1,08	ADM2	adrenomedullin 2
1,08	SIGLEC10	sialic acid binding Ig-like lectin 10
1,08	LYRM4	LYR motif containing 4
1,08	KIZ	kizuna centrosomal protein
1,08	PLCB2	phospholipase C, beta 2
1,08	SPIN2A	spindlin family, member 2A
1,08	GRXCR1	glutaredoxin, cysteine rich 1
1,08	NECAB3	N-terminal EF-hand calcium binding protein 3
1,08	LENG9	leukocyte receptor cluster (LRC) member 9
1,08	FUT7	fucosyltransferase 7 (alpha (1,3) fucosyltransferase)
1,08	SLC6A19	solute carrier family 6 (neutral amino acid transporter), member 19
1,08	DXO	decapping exoribonuclease
1,08	KYNU	kynureninase
1,08	NUDCD2	NudC domain containing 2
1,08	TYMSOS	TYMS opposite strand
1,08	CT47B1	cancer/testis antigen family 47, member B1
1,08	GAGE10	G antigen 10
1,08	TMEM163	transmembrane protein 163
1,08	ZNF577	zinc finger protein 577

1,08	GPR173	G protein-coupled receptor 173
1,08	LIMK1	LIM domain kinase 1
1,08	CRY2	cryptochrome circadian clock 2
1,08	ZNF501	zinc finger protein 501 [Source:HGNC Symbol;Acc:HGNC:23717]
1,08	CTDSPL	CTD small phosphatase like
1,08	PNO1	partner of NOB1 homolog
1,08	MMAA	methylmalonic aciduria (cobalamin deficiency) cblA type
1,08	NMNAT2	nicotinamide nucleotide adenylyltransferase 2
1,08	VASH2	vasohibin 2
1,08	ZNF91	zinc finger protein 91
1,08	TTC21B	tetratricopeptide repeat domain 21B
1,08	POMC	proopiomelanocortin
1,08	IGFBP2	insulin like growth factor binding protein 2
1,08	AIF1	allograft inflammatory factor 1
1,08	SMG9	SMG9 nonsense mediated mRNA decay factor
1,08	SWSAP1	SWIM-type zinc finger 7 associated protein 1
1,08	ZNF518B	zinc finger protein 518B
1,08	IREB2	iron responsive element binding protein 2
1,08	SPATA17	spermatogenesis associated 17
1,08	FZD10	frizzled class receptor 10
1,08	GRP	gastrin-releasing peptide
1,08	ASCC3	Memczak2013 ALT_ACCEPTOR, ALT_DONOR, coding, INTERNAL, intronic best transcript NM_006828
1,08	MAGEA1	MAGE family member A1
1,08	ZNF131	zinc finger protein 131
1,08	NAT8	N-acetyltransferase 8 (GCN5-related, putative)
1,08	TNFRSF1A	tumor necrosis factor receptor superfamily, member 1A
1,08	PARVG	parvin, gamma
1,08	RFESD	Rieske (Fe-S) domain containing
1,08	NKAIN3	Na+/K+ transporting ATPase interacting 3
1,08	KLHL5	kelch-like family member 5
1,08	ARL2BP	ADP-ribosylation factor like GTPase 2 binding protein
1,08	CEP44	centrosomal protein 44kDa
1,08	RCAN2	regulator of calcineurin 2
1,08	ARMCX5-GPRASP2	ARMCX5-GPRASP2 readthrough
1,08	ZNF668	zinc finger protein 668
1,08	KCNA5	potassium channel, voltage gated shaker related subfamily A, member 5
1,08	FABP6	Memczak2013 ANTISENSE, coding, INTERNAL, intronic best transcript NM_001040442
1,08	ARMCX4	armadillo repeat containing, X-linked 4
1,08	CDKN2A	cyclin-dependent kinase inhibitor 2A
1,08	FLT1	fms-related tyrosine kinase 1
1,08	VAT1	vesicle amine transport 1

1,08	TMPRSS5	transmembrane protease, serine 5
1,08	TBXA2R	thromboxane A2 receptor
1,08	OLA1	Obg-like ATPase 1
1,08	HNRNPCL1	heterogeneous nuclear ribonucleoprotein C-like 1
1,08	FAM86B1	family with sequence similarity 86, member B1
1,08	SCTR	secretin receptor
1,08	PLCL2	Memczak2013 ANTISENSE, coding, INTERNAL, intronic best transcript NM_001144382
1,08	FOLR1	folate receptor 1 (adult)
1,08	SEMA4G	sema domain, immunoglobulin domain (Ig), transmembrane domain (TM) and short cytoplasmic domain, (semaphorin) 4G
1,08	HAPLN1	hyaluronan and proteoglycan link protein 1
1,08	ITGA2	integrin, alpha 2 (CD49B, alpha 2 subunit of VLA-2 receptor)
1,08	ZGRF1	zinc finger, GRF-type containing 1
1,08	LOC101929372; AC004019.13	uncharacterized LOC101929372; novel transcript
1,08	NUFIP1	nuclear fragile X mental retardation protein interacting protein 1
1,08	LHX4	LIM homeobox 4
1,08	GAREM2	GRB2 associated regulator of MAPK1 2
1,08	CDK5R2	cyclin-dependent kinase 5, regulatory subunit 2 (p39)
1,08	GALP	galanin-like peptide
1,08	C2CD5	C2 calcium-dependent domain containing 5
1,08	RIMS2	regulating synaptic membrane exocytosis 2
1,08	CD93	CD93 molecule
1,08	RPL7A	ribosomal protein L7a
1,08	GAS8-AS1	GAS8 antisense RNA 1
1,08	SNX21	sorting nexin family member 21
1,08	SCNN1A	sodium channel, non voltage gated 1 alpha subunit
1,08	GLI2	GLI family zinc finger 2
1,08	SF3B4	splicing factor 3b, subunit 4, 49kDa
1,08	SPG11	spastic paraplegia 11 (autosomal recessive)
1,08	MT1M	metallothionein 1M
1,08	GDAP1	ganglioside induced differentiation associated protein 1
1,08	NAT10	N-acetyltransferase 10 (GCN5-related)
1,08	LAPTM5	lysosomal protein transmembrane 5
1,08	TNFSF4	tumor necrosis factor (ligand) superfamily, member 4
1,08	ASPG	asparaginase
1,08	C11orf24	chromosome 11 open reading frame 24
1,08	KAAG1	kidney associated antigen 1
1,08	TMEM176A	transmembrane protein 176A
1,08	RTN4RL2	reticulon 4 receptor-like 2
1,08	PKDREJ	polycystin (PKD) family receptor for egg jelly
1,08	HCN1	hyperpolarization activated cyclic nucleotide gated potassium channel 1
1,08	ST20-AS1	ST20 antisense RNA 1
1,08	ZNF75D	zinc finger protein 75D

1,08	DLX1	distal-less homeobox 1
1,08	RHBDL2	rhomboid, veinlet-like 2 (Drosophila)
1,08	KIF25-AS1	KIF25 antisense RNA 1
1,08	PNPLA2	patatin-like phospholipase domain containing 2
1,08	PRSS3	protease, serine, 3
1,08	HDLBP	Jeck2013 ANTISENSE, coding, INTERNAL, intronic best transcript NM_005336
1,08	MOS	v-mos Moloney murine sarcoma viral oncogene homolog
1,08	LINC01599	long intergenic non-protein coding RNA 1599
1,08	SNRPF	small nuclear ribonucleoprotein polypeptide F
1,08	AXDND1	axonemal dynein light chain domain containing 1
1,08	ACTA1	actin, alpha 1, skeletal muscle
1,08	PIGU	phosphatidylinositol glycan anchor biosynthesis class U
1,08	AKR1C2	aldo-keto reductase family 1, member C2
1,08	ZNF134	zinc finger protein 134
1,08	CCNJL	cyclin J-like
1,08	YIPF4	Yip1 domain family member 4
1,08	TBC1D12	TBC1 domain family, member 12
1,08	DLAT	dihydrolipoamide S-acetyltransferase
1,08	NIT2	nitrilase family, member 2
1,08	FAM160B1	family with sequence similarity 160, member B1
1,08	LRRCC1	Transcript Identified by AceView, Entrez Gene ID(s) 85444
1,08	KIAA1217	KIAA1217
1,08	ZNF823	zinc finger protein 823
1,08	DLK1	delta-like 1 homolog (Drosophila)
1,08	C16orf82	chromosome 16 open reading frame 82
1,08	LIG4	ligase IV, DNA, ATP-dependent
1,08	SYTL3	synaptotagmin-like 3
1,08	TBC1D8B	TBC1 domain family, member 8B (with GRAM domain)
1,08	MANSC1	MANSC domain containing 1
1,08	RASA3	RAS p21 protein activator 3
1,08	PIM2	Pim-2 proto-oncogene, serine/threonine kinase
1,08	TCEAL4	transcription elongation factor A (SII)-like 4
1,08	TNFSF9	tumor necrosis factor (ligand) superfamily, member 9
1,08	OR51I2	olfactory receptor, family 51, subfamily I, member 2
1,08	HBG2; HBG1	hemoglobin, gamma G; hemoglobin, gamma A
1,08	LCE3C	late cornified envelope 3C
1,08	OR5AC1	olfactory receptor, family 5, subfamily AC, member 1 (gene/pseudogene)
1,08	PRPH2	peripherin 2 (retinal degeneration, slow)
1,08	RSBN1L	round spermatid basic protein 1-like
1,08	C2CD4D	C2 calcium-dependent domain containing 4D
1,08	IL36G	interleukin 36, gamma
1,08	SLC45A1	solute carrier family 45, member 1
1,08	USP27X	ubiquitin specific peptidase 27, X-linked
1,08	IFNA4	interferon, alpha 4

1,08	PERP	PERP, TP53 apoptosis effector
1,08	PPIAP30	peptidylprolyl isomerase A (cyclophilin A) pseudogene 30
1,08	RTKN2	rhotekin 2
1,08	SPTBN4	spectrin, beta, non-erythrocytic 4
1,08	CCDC84	coiled-coil domain containing 84
1,08	CABP2	calcium binding protein 2
1,08	TNR	tenascin R
1,08	FAM153C	family with sequence similarity 153, member C, pseudogene
1,08	RPE65	retinal pigment epithelium-specific protein 65kDa
1,08	GPR180	G protein-coupled receptor 180
1,08	CDC42BPG	CDC42 binding protein kinase gamma (DMPK-like)
1,08	TPTE2	transmembrane phosphoinositide 3-phosphatase and tensin homolog 2
1,08	PCBP4	poly(rC) binding protein 4
1,08	CHRM1	cholinergic receptor, muscarinic 1
1,08	CPZ; GPR78	carboxypeptidase Z; G protein-coupled receptor 78
1,08	GABRD	gamma-aminobutyric acid (GABA) A receptor, delta
1,08	KIAA0226L	KIAA0226-like
1,08	VWA5A	von Willebrand factor A domain containing 5A
1,08	EFHD1	EF-hand domain family member D1
1,08	SMIM10	small integral membrane protein 10
1,08	KRTAP22-2	keratin associated protein 22-2
1,08	TBC1D10A	TBC1 domain family, member 10A
1,08	CLEC19A	C-type lectin domain family 19, member A
1,08	ANKRD12	ankyrin repeat domain 12
1,08	NCK2	NCK adaptor protein 2
1,08	PENK	proenkephalin
1,08	IGFL2	IGF like family member 2
1,08	TAS2R16	taste receptor, type 2, member 16
1,08	TMSB4Y	thymosin beta 4, Y-linked
1,08	RFC3	Memczak2013 ALT_ACCEPTOR, ALT_DONOR, coding, INTERNAL, intronic best transcript NM_181558
1,08	ZIM3	zinc finger, imprinted 3
1,08	ACTR6	ARP6 actin-related protein 6 homolog (yeast)
1,08	SFTPA1	surfactant protein A1
1,08	ATG16L1	autophagy related 16-like 1
1,08	MED18	mediator complex subunit 18
1,08	CYP2C18	cytochrome P450, family 2, subfamily C, polypeptide 18
1,08	TMX2; C11orf31	thioredoxin-related transmembrane protein 2; chromosome 11 open reading frame 31
1,08	BAG6	BCL2 associated athanogene 6
1,08	CABP1	calcium binding protein 1
1,08	ADAM19	ADAM metallopeptidase domain 19
1,08	MPZL1	myelin protein zero-like 1
1,08	OR8B8	olfactory receptor, family 8, subfamily B, member 8
1,08	CTRB1	chymotrypsinogen B1

1,08	PRAC1	prostate cancer susceptibility candidate 1
1,08	POMT2	protein-O-mannosyltransferase 2
1,08	ABI2	abl-interactor 2
1,08	FERD3L	Fer3-like bHLH transcription factor
1,08	IL1RAPL1	interleukin 1 receptor accessory protein-like 1
1,08	TLX3	T-cell leukemia homeobox 3
1,08	KCNA4	potassium channel, voltage gated shaker related subfamily A, member 4
1,08	GAS6-AS1	GAS6 antisense RNA 1
1,08	PEX7	peroxisomal biogenesis factor 7
1,08	REEP1	receptor accessory protein 1
1,08	OR5T2	olfactory receptor, family 5, subfamily T, member 2
1,08	DPPA4	developmental pluripotency associated 4
1,08	CYP2D6	cytochrome P450, family 2, subfamily D, polypeptide 6
1,08	ZFP64	ZFP64 zinc finger protein
1,08	BATF	basic leucine zipper transcription factor, ATF-like
1,08	TEF	thyrotrophic embryonic factor
1,08	GSTT2; GSTT2B	glutathione S-transferase theta 2 (gene/pseudogene); glutathione S-transferase theta 2B (gene/pseudogene)
1,08	RASA4	Salzman2013 ALT_ACCEPTOR, ALT_DONOR, coding, INTERNAL, intronic best transcript NM_006989
1,08	RPLP2; SNORA52	ribosomal protein, large, P2; small nucleolar RNA, H/ACA box 52
1,08	MPC1L	mitochondrial pyruvate carrier 1-like
1,08	APOBEC3C	apolipoprotein B mRNA editing enzyme, catalytic polypeptide-like 3C
1,08	OSBPL9	oxysterol binding protein-like 9
1,08	DPYS	dihydropyrimidinase
1,08	NOLC1	nucleolar and coiled-body phosphoprotein 1
1,08	CNNM3	Zhang2013 ALT_ACCEPTOR, ALT_DONOR, coding, INTERNAL, intronic, OVERLAPTX best transcript NM_017623
1,08	DDX11	Zhang2013 ALT_ACCEPTOR, ALT_DONOR, coding, INTERNAL, intronic best transcript NM_030653
1,08	ZNF614	zinc finger protein 614
1,08	TELO2	telomere maintenance 2
1,08	KCNQ5	potassium channel, voltage gated KQT-like subfamily Q, member 5
1,08	CCDC141	coiled-coil domain containing 141
1,08	IFI44	interferon-induced protein 44
1,08	DSCR10	Down syndrome critical region 10 (non-protein coding)
1,08	RETN	resistin
1,08	PRDM13	PR domain containing 13
1,08	OR4A16	olfactory receptor, family 4, subfamily A, member 16
1,08	CFP	complement factor properdin
1,08	LBX2	ladybird homeobox 2

1,08	SLITRK3	SLIT and NTRK-like family, member 3
1,08	CRMP1	Salzman2013 ANTISENSE, CDS, coding, INTERNAL, intronic, OVCODE, OVERLAPTX, OVEXON best transcript NM_001014809
1,08	ALDOB	aldolase B, fructose-bisphosphate
1,08	C9orf3; RP11-49O14.3	Transcript Identified by AceView, Entrez Gene ID(s) 84909; novel transcript
1,08	FAM71D	family with sequence similarity 71, member D
1,08	LINC01521	long intergenic non-protein coding RNA 1521
1,08	FXYD5	FXYD domain containing ion transport regulator 5
1,08	PLD4	phospholipase D family, member 4
1,08	ALPK2	alpha kinase 2
1,08	SLC29A4	solute carrier family 29 (equilibrative nucleoside transporter), member 4
1,08	KLF8	Kruppel-like factor 8
1,08	CXCL2	chemokine (C-X-C motif) ligand 2
1,08	MAPK8IP3	mitogen-activated protein kinase 8 interacting protein 3
1,08	MAGI3	Jeck2013 ALT_ACCEPTOR, ALT_DONOR, coding, INTERNAL, intronic best transcript NM_152900
1,08	ZDHHC3	zinc finger, DHHC-type containing 3
1,08	DVL1; MIR6808	dishevelled segment polarity protein 1; microRNA 6808
1,08	LDHC	lactate dehydrogenase C
1,08	TMC8	transmembrane channel like 8
1,08	MTMR4	myotubularin related protein 4
1,08	TRIM58	tripartite motif containing 58
1,08	ACHE	acetylcholinesterase (Yt blood group)
1,08	PRKAA1	protein kinase, AMP-activated, alpha 1 catalytic subunit
1,08	MUCL1	mucin-like 1
1,08	HS3ST3B1	heparan sulfate (glucosamine) 3-O-sulfotransferase 3B1
1,08	HAP1	huntingtin-associated protein 1
1,08	RMND5B	required for meiotic nuclear division 5 homolog B
1,08	IGFALS	insulin-like growth factor binding protein, acid labile subunit
1,08	HMX1	H6 family homeobox 1
1,08	HNRNPCL1	heterogeneous nuclear ribonucleoprotein C-like 1
1,08	TRMT2B	tRNA methyltransferase 2 homolog B
1,08	ALS2CR12	amyotrophic lateral sclerosis 2 chromosome region candidate 12
1,08	TERF2	telomeric repeat binding factor 2
1,08	TP53TG3D	TP53 target 3D
1,08	CIB2	calcium and integrin binding family member 2
1,08	FNDC3B	fibronectin type III domain containing 3B
1,08	NRN1L	neuritin 1-like
1,08	AUNIP	aurora kinase A and ninein interacting protein
1,08	SLC25A16	solute carrier family 25 (mitochondrial carrier), member 16

1,08	RAB6C	RAB6C, member RAS oncogene family
1,08	FARSA	phenylalanyl-tRNA synthetase alpha subunit
1,08	FAM151A	family with sequence similarity 151, member A
1,08	SLC35C2	solute carrier family 35 (GDP-fucose transporter), member C2
1,08	TRMT11	tRNA methyltransferase 11 homolog
1,08	P2RX6	purinergic receptor P2X, ligand gated ion channel, 6
1,08	NMRAL1	NmrA-like family domain containing 1
1,08	TMEM59L	transmembrane protein 59-like
1,08	S100A10	S100 calcium binding protein A10
1,08	SAG	S-antigen; retina and pineal gland (arrestin)
1,08	VCX2	variable charge, X-linked 2
1,08	PRSS56	protease, serine, 56
1,08	GRHL1	grainyhead-like transcription factor 1
1,08	CCDC136	coiled-coil domain containing 136
1,08	METAP1	methionyl aminopeptidase 1
1,08	RBM12; CPNE1	RNA binding motif protein 12; copine I
1,08	MPP4	membrane protein, palmitoylated 4
1,08	KIF5B	Memczak2013 ANTISENSE, CDS, coding, INTERNAL, UTR3 best transcript NM_004521
1,08	C14orf39	chromosome 14 open reading frame 39
1,08	RTN4	reticulon 4
1,08	EHD3	EH domain containing 3
1,08	MT2A	metallothionein 2A
1,08	LTK	leukocyte receptor tyrosine kinase
1,08	TNFSF10	tumor necrosis factor (ligand) superfamily, member 10
1,08	ATP5L2	ATP synthase, H+ transporting, mitochondrial Fo complex subunit G2
1,08	HKR1	HKR1, GLI-Kruppel zinc finger family member
1,08	ZNF35	zinc finger protein 35
1,08	VPS11	VPS11, CORVET/HOPS core subunit [Source:HGNC Symbol;Acc:HGNC:14583]
1,08	MRPS35	mitochondrial ribosomal protein S35
1,08	MYL10	myosin light chain 10
1,08	RFK	riboflavin kinase
1,08	OR14J1	olfactory receptor, family 14, subfamily J, member 1
1,08	POP4	POP4 homolog, ribonuclease P/MRP subunit
1,08	MTRR	5-methyltetrahydrofolate-homocysteine methyltransferase reductase
1,08	MYO1A	myosin IA
1,08	CCSER1	coiled-coil serine rich protein 1
1,08	ESRRA	estrogen-related receptor alpha
1,08	CSNK2A1	casein kinase 2, alpha 1 polypeptide
1,08	LRP5	LDL receptor related protein 5
1,08	CD38	CD38 molecule
1,08	CHMP6	charged multivesicular body protein 6
1,08	INSR	insulin receptor
1,08	SLC6A12	solute carrier family 6 (neurotransmitter transporter), member 12

1,08	HCRT	hypocretin (orexin) neuropeptide precursor
1,08	HOXD1; MIR7704	homeobox D1; microRNA 7704
1,08	ORC6	origin recognition complex subunit 6
1,08	DTL	denticleless E3 ubiquitin protein ligase homolog (Drosophila)
1,08	GFI1B	growth factor independent 1B transcription repressor
1,08	TMEM156	transmembrane protein 156
1,08	ICAM3	intercellular adhesion molecule 3
1,08	TMEM179B; MIR6748	transmembrane protein 179B; microRNA 6748
1,08	DNAH5	Transcript Identified by AceView, Entrez Gene ID(s) 1767
1,08	PLSCR5	phospholipid scramblase family, member 5
1,08	PRKRIP1	PRKR interacting protein 1 (IL11 inducible)
1,08	CHCHD7	coiled-coil-helix-coiled-coil-helix domain containing 7
1,08	SCARB1	scavenger receptor class B, member 1
1,08	C12orf66	chromosome 12 open reading frame 66
1,08	SLC25A22	solute carrier family 25 (mitochondrial carrier: glutamate), member 22
1,08	LRRC2	leucine rich repeat containing 2
1,08	SCN1B	sodium channel, voltage gated, type I beta subunit
1,08	TAGAP	T-cell activation RhoGTPase activating protein
1,08	MSGN1	mesogenin 1
1,08	ZNF547; TRAPPC2B; TRAPPC2	zinc finger protein 547; trafficking protein particle complex 2B; trafficking protein particle complex 2
1,08	OBP2B	odorant binding protein 2B
1,08	CRYBB3	crystallin beta B3
1,08	CSPG4P1Y; DNM1P24	chondroitin sulfate proteoglycan 4 pseudogene 1, Y-linked; dynamin 1 pseudogene 24
1,08	TFR2	transferrin receptor 2
1,08	PLEKHB1	pleckstrin homology domain containing, family B (evectins) member 1
1,08	WFIKKN1	WAP, follistatin/kazal, immunoglobulin, kunitz and netrin domain containing 1
1,08	MPZL2	myelin protein zero-like 2
1,08	ZBTB49	zinc finger and BTB domain containing 49
1,08	MBOAT2	Memczak2013 ALT_ACCEPTOR, ALT_DONOR, coding, INTERNAL, intronic best transcript NM_138799
1,08	ADAMDEC1	ADAM-like, decysin 1
1,08	SPATA8	spermatogenesis associated 8
1,08	COX2	cytochrome c oxidase subunit II
1,08	TBC1D3B; TBC1D3H; TBC1D3I; TBC1D3F; TBC1D3G	TBC1 domain family, member 3B; TBC1 domain family, member 3H; TBC1 domain family, member 3I; TBC1 domain family, member 3F; TBC1 domain family, member 3G
1,08	METTL24	methyltransferase like 24

1,08	RPRD1B	regulation of nuclear pre-mRNA domain containing 1B
1,08	CNTD1	cyclin N-terminal domain containing 1
1,08	GALNT9	polypeptide N-acetylgalactosaminyltransferase 9
1,08	PITX2	paired-like homeodomain 2
1,08	DPYSL5	dihydropyrimidinase-like 5
1,08	SUPV3L1	SUV3-like helicase
1,08	NSUN5	NOP2/Sun domain family, member 5
1,08	SH2D1A	SH2 domain containing 1A
1,08	SYNJ2BP-COX16; COX16; SYNJ2BP	SYNJ2BP-COX16 readthrough; COX16 cytochrome c oxidase assembly homolog; synaptojanin 2 binding protein
1,08	PAPD4	PAP associated domain containing 4
1,08	CTB-75G16.3; MRM1	Transcript Identified by AceView, Entrez Gene ID(s) 79922; novel transcript
1,08	WDR19	WD repeat domain 19
1,08	CYP2A7	cytochrome P450, family 2, subfamily A, polypeptide 7
1,08	ACSL4	acyl-CoA synthetase long-chain family member 4
1,08	SLC46A2	solute carrier family 46, member 2
1,08	KIRREL3	Jeck2013 ALT_ACCEPTOR, ALT_DONOR, coding, INTERNAL, intronic best transcript NM_032531
1,08	GIMD1	GIMAP family P-loop NTPase domain containing 1
1,08	CCDC138	coiled-coil domain containing 138
1,08	SYCP3	synaptonemal complex protein 3
1,08	ASTE1	asteroid homolog 1 (Drosophila)
1,08	CYTB	cytochrome b
1,08	GAL	galanin/GMAP prepropeptide
1,08	OR7E24	olfactory receptor, family 7, subfamily E, member 24
1,08	ZDHHC8	zinc finger, DHHC-type containing 8
1,08	FKBP4	FK506 binding protein 4
1,08	FCGR3A	Fc fragment of IgG, low affinity IIIa, receptor (CD16a)
1,08	ME2	malic enzyme 2, NAD(+)-dependent, mitochondrial
1,08	RNPS1	RNA binding protein S1, serine-rich domain
1,08	FAM186A	family with sequence similarity 186, member A
1,08	GDE1	glycerophosphodiester phosphodiesterase 1
1,08	MOB3A	MOB kinase activator 3A
1,08	OR13D1	olfactory receptor, family 13, subfamily D, member 1
1,08	NME5	NME/NM23 family member 5
1,08	OBP2B	odorant binding protein 2B
1,08	C12orf76	chromosome 12 open reading frame 76
1,08	CFAP157	cilia and flagella associated protein 157
1,08	ATP8A1	ATPase, aminophospholipid transporter (APLT), class I, type 8A, member 1
1,08	TMEM259	transmembrane protein 259
1,08	FLRT2	fibronectin leucine rich transmembrane protein 2
1,08	IARS	isoleucyl-tRNA synthetase
1,08	MIR4738; H3F3B	microRNA 4738; H3 histone, family 3B (H3.3B)

1,08	PRH1; TAS2R14; PRR4	proline-rich protein HaeIII subfamily 1; taste receptor, type 2, member 14; proline rich 4 (lacrimal)
1,08	ARPP21	Transcript Identified by AceView, Entrez Gene ID(s) 10777
1,08	BRD7	bromodomain containing 7
1,08	CDRT15L2	CMT1A duplicated region transcript 15-like 2
1,08	CC2D2A	coiled-coil and C2 domain containing 2A
1,08	PANO1	proapoptotic nucleolar protein 1
1,08	DNAJC7	Zhang2013 ALT_ACCEPTOR, ALT_DONOR, coding, INTERNAL, intronic, OVERLAPTX, OVEXON best transcript NM_003315
1,08	GIGYF2	GRB10 interacting GYF protein 2
1,08	UTS2	urotensin 2
1,08	LCE2D	late cornified envelope 2D
1,08	POGZ	Transcript Identified by AceView, Entrez Gene ID(s) 23126
1,08	SCGB3A1	secretoglobin, family 3A, member 1
1,08	HMBS	hydroxymethylbilane synthase
1,08	CNOT2	CCR4-NOT transcription complex subunit 2
1,08	RPRD1A	regulation of nuclear pre-mRNA domain containing 1A
1,08	TMEM43	transmembrane protein 43
1,08	CYP1B1	cytochrome P450, family 1, subfamily B, polypeptide 1
1,08	COPS7A	COP9 signalosome subunit 7A
1,08	ZNF467	zinc finger protein 467
1,08	GRAMD1A	GRAM domain containing 1A
1,08	PARP8	poly(ADP-ribose) polymerase family member 8
1,08	IL10	interleukin 10
1,08	ADORA3; TMIGD3	adenosine A3 receptor; transmembrane and immunoglobulin domain containing 3
1,08	CRK	v-crk avian sarcoma virus CT10 oncogene homolog
1,08	SH2D6	SH2 domain containing 6
1,08	DEFB108B	defensin, beta 108B
1,08	DFNA5	deafness, autosomal dominant 5
1,08	IL1A	interleukin 1 alpha
1,08	DDX39B; SNORD84	DEAD (Asp-Glu-Ala-Asp) box polypeptide 39B; small nucleolar RNA, C/D box 84
1,07	KRT9	keratin 9, type I
1,07	FRS3	fibroblast growth factor receptor substrate 3
1,07	LOC100130880; AC022173.2	uncharacterized LOC100130880; novel transcript
1,07	STH	saitohin
1,07	PAPSS2	3-phosphoadenosine 5-phosphosulfate synthase 2
1,07	CNEP1R1	CTD nuclear envelope phosphatase 1 regulatory subunit 1
1,07	CEP126	centrosomal protein 126kDa
1,07	GZMA	granzyme A
1,07	SLC25A39	solute carrier family 25, member 39
1,07	IZUMO1	izumo sperm-egg fusion 1

1,07	RAI14	Transcript Identified by AceView, Entrez Gene ID(s) 26064
1,07	WASL	Wiskott-Aldrich syndrome-like
1,07	MTA1	metastasis associated 1
1,07	TRIB2	tribbles pseudokinase 2
1,07	SIAH1	siah E3 ubiquitin protein ligase 1
1,07	LMF1	lipase maturation factor 1
1,07	PAK7	p21 protein (Cdc42/Rac)-activated kinase 7
1,07	TEPP	testis, prostate and placenta expressed
1,07	SLC26A5	solute carrier family 26 (anion exchanger), member 5
1,07	CNDP2	CNDP dipeptidase 2 (metallopeptidase M20 family)
1,07	OSGIN2	oxidative stress induced growth inhibitor family member 2
1,07	SIRT3	sirtuin 3
1,07	RNH1	ribonuclease/angiogenin inhibitor 1
1,07	VIP	vasoactive intestinal peptide
1,07	DCAF15	DDB1 and CUL4 associated factor 15
1,07	SLC1A3	solute carrier family 1 (glial high affinity glutamate transporter), member 3
1,07	HIST1H2BK	histone cluster 1, H2bk
1,07	RNF133	ring finger protein 133
1,07	CDH5	cadherin 5, type 2 (vascular endothelium)
1,07	VHLL	von Hippel-Lindau tumor suppressor-like
1,07	CNTROB	Transcript Identified by AceView, Entrez Gene ID(s) 116840
1,07	SLC2A6	solute carrier family 2 (facilitated glucose transporter), member 6 [Source:HGNC Symbol;Acc:HGNC:11011]
1,07	SLC9A5	solute carrier family 9, subfamily A (NHE5, cation proton antiporter 5), member 5
1,07	OR4E2	olfactory receptor, family 4, subfamily E, member 2
1,07	KRBOX1	KRAB box domain containing 1
1,07	NKG7	natural killer cell granule protein 7
1,07	SEC31A	SEC31 homolog A, COPII coat complex component
1,07	ABCD2	ATP binding cassette subfamily D member 2
1,07	CACNA1D	calcium channel, voltage-dependent, L type, alpha 1D subunit
1,07	RPUSD1	RNA pseudouridylate synthase domain containing 1
1,07	TMEM38B	transmembrane protein 38B
1,07	URAD	ureidoimidazoline (2-oxo-4-hydroxy-4-carboxy-5-) decarboxylase
1,07	B3GALT5	UDP-Gal:betaGlcNAc beta 1,3-galactosyltransferase 5
1,07	PRPF4	pre-mRNA processing factor 4
1,07	IL33	interleukin 33
1,07	ASL	argininosuccinate lyase
1,07	TRIM21	tripartite motif containing 21
1,07	CTDP1	CTD phosphatase subunit 1
1,07	DOK5	docking protein 5
1,07	ARFIP1	ADP-ribosylation factor interacting protein 1

1,07	ZDHHC12	zinc finger, DHHC-type containing 12
1,07	CTSC	cathepsin C
1,07	MPO	Memczak2013 ANTISENSE, CDS, coding, INTERNAL best transcript NM_000250
1,07	NINJ1	ninjurin 1
1,07	SERPINI2	serpin peptidase inhibitor, clade I (pancpin), member 2
1,07	DLL4	delta-like 4 (Drosophila)
1,07	HSCB	HscB mitochondrial iron-sulfur cluster co-chaperone
1,07	OR2B6	olfactory receptor, family 2, subfamily B, member 6
1,07	ZNF835	Transcript Identified by AceView, Entrez Gene ID(s) 90485
1,07	ENPP6	ectonucleotide pyrophosphatase/phosphodiesterase 6
1,07	HNRNPH1	heterogeneous nuclear ribonucleoprotein H1 (H)
1,07	MYL2	myosin light chain 2
1,07	ZNF813	zinc finger protein 813
1,07	PLXNA2	plexin A2
1,07	GFOD1	glucose-fructose oxidoreductase domain containing 1
1,07	SYP	synaptophysin
1,07	SLC6A6	solute carrier family 6 (neurotransmitter transporter), member 6
1,07	KLK1	kallikrein 1
1,07	FTCDNL1	formiminotransferase cyclodeaminase N-terminal like
1,07	TM4SF19-AS1	TM4SF19 antisense RNA 1
1,07	VPS11	VPS11, CORVET/HOPS core subunit [Source:HGNC Symbol;Acc:HGNC:14583]
1,07	CALHM2	calcium homeostasis modulator 2
1,07	PLLP	plasmolipin
1,07	TOPAZ1	testis and ovary specific PAZ domain containing 1
1,07	CTC1	CTS telomere maintenance complex component 1
1,07	LOC100505841	zinc finger protein 474-like
1,07	ZNF610	zinc finger protein 610
1,07	TMEM102	transmembrane protein 102
1,07	PIGU	Salzman2013 ALT_ACCEPTOR, ALT_DONOR, coding, INTERNAL, intronic best transcript NM_080476
1,07	TMEM37	transmembrane protein 37
1,07	CAPZA2	capping protein (actin filament) muscle Z-line, alpha 2
1,07	TBC1D19	TBC1 domain family, member 19
1,07	C11orf71	chromosome 11 open reading frame 71
1,07	OST4	oligosaccharyltransferase complex subunit 4 (non-catalytic)
1,07	DCAF8L2	DDB1 and CUL4 associated factor 8-like 2
1,07	APEX2	Transcript Identified by AceView, Entrez Gene ID(s) 27301
1,07	LOC728395; TSPY3	Homo sapiens similar to testis specific protein, Y-linked 1, mRNA (cDNA clone MGC:150413 IMAGE:40120433), complete cds.; Homo sapiens testis specific protein, Y-linked 3, mRNA (cDNA clone MGC:150414 IMAGE:40120435), complete cds.

1,07	BANP	BTG3 associated nuclear protein
1,07	OXSM	3-oxoacyl-ACP synthase, mitochondrial
1,07	ADGRE3	adhesion G protein-coupled receptor E3
1,07	TP53TG3B	TP53 target 3B
1,07	SMC6	structural maintenance of chromosomes 6
1,07	LAMC3	laminin, gamma 3
1,07	FRG1	FSHD region gene 1
1,07	MBD3L4	methyl-CpG binding domain protein 3-like 4
1,07	CLP1; AP000662.4	Transcript Identified by AceView, Entrez Gene ID(s) 10978; novel transcript, sense overlapping CLP1
1,07	LGALS9B	lectin, galactoside-binding, soluble, 9B
1,07	SULT1A2	sulfotransferase family 1A member 2
1,07	MPO	myeloperoxidase
1,07	ANKRD45	ankyrin repeat domain 45
1,07	ADH7	alcohol dehydrogenase 7 (class IV), mu or sigma polypeptide
1,07	WDR75	WD repeat domain 75
1,07	CCDC144NL	coiled-coil domain containing 144 family, N-terminal like
1,07	TSNAXIP1	translin-associated factor X interacting protein 1
1,07	FBXO15	F-box protein 15
1,07	VDAC2	voltage-dependent anion channel 2
1,07	RBMXL3	RNA binding motif protein, X-linked-like 3
1,07	MDM1	Transcript Identified by AceView, Entrez Gene ID(s) 56890
1,07	TBC1D3C	TBC1 domain family, member 3C
1,07	MARVELD1	MARVEL domain containing 1
1,07	SORD	sorbitol dehydrogenase
1,07	LPCAT1	Memczak2013 ANTISENSE, coding, INTERNAL, intronic best transcript NM_024830
1,07	PDE1B	phosphodiesterase 1B, calmodulin-dependent
1,07	CLYBL	citrate lyase beta like
1,07	RABIF	RAB interacting factor
1,07	SPATA31E1	SPATA31 subfamily E, member 1
1,07	OAT	ornithine aminotransferase
1,07	GPR149	G protein-coupled receptor 149
1,07	GCSH	glycine cleavage system protein H (aminomethyl carrier)
1,07	ARHGAP40	Rho GTPase activating protein 40
1,07	FOXO6	forkhead box O6
1,07	WASH1	WAS protein family homolog 1
1,07	NBN	nibrin
1,07	IQCF6	IQ motif containing F6
1,07	GLMP	glycosylated lysosomal membrane protein
1,07	REXO4	REX4 homolog, 3'-5' exonuclease [Source:HGNC Symbol;Acc:HGNC:12820]
1,07	PABPC1; MIR7705	poly(A) binding protein, cytoplasmic 1; microRNA 7705
1,07	ST3GAL6	ST3 beta-galactoside alpha-2,3-sialyltransferase 6
1,07	ARHGEF7	Rho guanine nucleotide exchange factor 7

1,07	NLE1	notchless homolog 1 (Drosophila)
1,07	MFSD2A	major facilitator superfamily domain containing 2A
1,07	MTHFD2L	methylenetetrahydrofolate dehydrogenase (NADP+ dependent) 2-like
1,07	DNAJC11	DnaJ (Hsp40) homolog, subfamily C, member 11
1,07	RFPL2	ret finger protein-like 2
1,07	LOC401357; RP13-580B18.4	uncharacterized LOC401357; novel piRNA host transcript
1,07	SLC35A1	solute carrier family 35 (CMP-sialic acid transporter), member A1
1,07	OR5B2	olfactory receptor, family 5, subfamily B, member 2
1,07	RIPPLY2	ripply transcriptional repressor 2
1,07	SHQ1	SHQ1, H/ACA ribonucleoprotein assembly factor
1,07	C2orf61	chromosome 2 open reading frame 61
1,07	AMN	amnion associated transmembrane protein
1,07	CPSF7	cleavage and polyadenylation specific factor 7
1,07	RPS26	Homo sapiens ribosomal protein S26, mRNA (cDNA clone MGC:104291 IMAGE:4287636), complete cds.
1,07	HTR1F	5-hydroxytryptamine (serotonin) receptor 1F, G protein-coupled
1,07	COX8A	cytochrome c oxidase subunit VIIIA (ubiquitous)
1,07	SEPHS2	selenophosphate synthetase 2
1,07	BPGM	2,3-bisphosphoglycerate mutase
1,07	PARP6	poly(ADP-ribose) polymerase family member 6
1,07	ANKRD2	ankyrin repeat domain 2 (stretch responsive muscle)
1,07	FGFBP2	fibroblast growth factor binding protein 2
1,07	FBXW7	F-box and WD repeat domain containing 7, E3 ubiquitin protein ligase
1,07	PPP2R1B	protein phosphatase 2, regulatory subunit A, beta
1,07	APOL2	apolipoprotein L, 2
1,07	CAPRIN2	caprin family member 2
1,07	MXRA8	matrix-remodelling associated 8
1,07	MRPS18A	mitochondrial ribosomal protein S18A
1,07	TBC1D3P1-DHX40P1	TBC1D3P1-DHX40P1 readthrough transcribed pseudogene
1,07	LINC00654	long intergenic non-protein coding RNA 654
1,07	PPFIA3	protein tyrosine phosphatase, receptor type, f polypeptide (PTPRF), interacting protein (liprin), alpha 3
1,07	UBE2Q2L	ubiquitin conjugating enzyme E2Q family member 2-like
1,07	RPS6KA1	ribosomal protein S6 kinase, 90kDa, polypeptide 1
1,07	SLC38A5	solute carrier family 38, member 5
1,07	CD81	CD81 molecule
1,07	RWDD2B	RWD domain containing 2B
1,07	STAG3L3; STAG3L2	stromal antigen 3-like 3 (pseudogene); stromal antigen 3-like 2 (pseudogene)
1,07	MUC22	mucin 22
1,07	MYH2	myosin, heavy chain 2, skeletal muscle, adult
1,07	MT1E	metallothionein 1E

1,07	CCP110	centriolar coiled coil protein 110kDa
1,07	ETF1	eukaryotic translation termination factor 1
1,07	ERBB2IP	erbb2 interacting protein
1,07	GABRP	gamma-aminobutyric acid (GABA) A receptor, pi
1,07	IGSF22	immunoglobulin superfamily, member 22
1,07	CMTR2	cap methyltransferase 2
1,07	MLH3	mutL homolog 3
1,07	IMPAD1	inositol monophosphatase domain containing 1
1,07	CRY1	cryptochrome circadian clock 1
1,07	NDUFS8; MIR4691; MIR7113	NADH dehydrogenase (ubiquinone) Fe-S protein 8, 23kDa (NADH-coenzyme Q reductase); microRNA 4691; microRNA 7113
1,07	ZNF280C	zinc finger protein 280C
1,07	DUX4L8	double homeobox 4 like 8
1,07	GPR176	G protein-coupled receptor 176
1,07	LMBR1	Transcript Identified by AceView, Entrez Gene ID(s) 64327
1,07	PPTC7	PTC7 protein phosphatase homolog
1,07	SNAP91	synaptosome associated protein 91kDa
1,07	SOWAHD	sosondowah ankyrin repeat domain family member D
1,07	PSMC3IP	PSMC3 interacting protein
1,07	TCF4	transcription factor 4
1,07	CASP6	caspase 6
1,07	TUBA4A	tubulin, alpha 4a
1,07	OR13C8	olfactory receptor, family 13, subfamily C, member 8
1,07	BAGE; BAGE4; BAGE3; BAGE2; BAGE5	B melanoma antigen; B melanoma antigen family, member 4; B melanoma antigen family, member 3; B melanoma antigen family, member 2; B melanoma antigen family, member 5
1,07	ADAMTS13	ADAM metallopeptidase with thrombospondin type 1 motif 13
1,07	CNTN2	contactin 2 (axonal)
1,07	SMARCD3	SWI/SNF related, matrix associated, actin dependent regulator of chromatin, subfamily d, member 3
1,07	CCDC9	coiled-coil domain containing 9
1,07	IZUMO2	IZUMO family member 2
1,07	VDR	vitamin D (1,25- dihydroxyvitamin D3) receptor
1,07	METTL7A	methyltransferase like 7A
1,07	DUX4L24; DUX4L23	double homeobox 4 like 24; double homeobox 4 like 23
1,07	DUX4L22; DUX4L21	double homeobox 4 like 22; double homeobox 4 like 21
1,07	DUX4L10; DUX4L11	double homeobox 4 like 10; double homeobox 4 like 11
1,07	NUDT3	nudix hydrolase 3
1,07	KCNE5; ACSL4	potassium channel, voltage gated subfamily E regulatory beta subunit 5; acyl-CoA synthetase long-chain family member 4
1,07	PKP3	plakophilin 3
1,07	CLN3; NPIPB7	ceroid-lipofuscinosis, neuronal 3; nuclear pore complex interacting protein family, member B7

1,07	FRS2	fibroblast growth factor receptor substrate 2
1,07	PYHIN1	pyrin and HIN domain family, member 1
1,07	ARL6IP6	ADP-ribosylation factor like GTPase 6 interacting protein 6
1,07	HDC	histidine decarboxylase
1,07	NKX2-2	NK2 homeobox 2
1,07	KCTD16	potassium channel tetramerization domain containing 16
1,07	CHST11	Transcript Identified by AceView, Entrez Gene ID(s) 50515
1,07	RICTOR	Memczak2013 ANTISENSE, coding, INTERNAL, intronic best transcript NM_152756
1,07	IFI16	interferon, gamma-inducible protein 16
1,07	ATG16L1	autophagy related 16-like 1
1,07	POMT1	protein-O-mannosyltransferase 1
1,07	IRS2	insulin receptor substrate 2
1,07	PNRC1	proline-rich nuclear receptor coactivator 1
1,07	C16orf70	chromosome 16 open reading frame 70
1,07	PRAMEF25; PRAMEF26	PRAME family member 25; PRAME family member 26
1,07	PES1	pescadillo ribosomal biogenesis factor 1
1,07	FAM45A	family with sequence similarity 45, member A
1,07	CNP	Memczak2013 ANTISENSE, CDS, coding, INTERNAL best transcript NM_033133
1,07	RSPH10B2	radial spoke head 10 homolog B2 (Chlamydomonas)
1,07	CES1P1; CES1	carboxylesterase 1 pseudogene 1; carboxylesterase 1
1,07	CTSB	Jeck2013 ALT_DONOR, coding, INTERNAL, intronic best transcript NM_147780
1,07	ODC1; SNORA80B	ornithine decarboxylase 1; small nucleolar RNA, H/ACA box 80B
1,07	CAPN14	calpain 14
1,07	SERINC3	serine incorporator 3
1,07	GRIK5	glutamate receptor, ionotropic, kainate 5
1,07	DUX4	double homeobox 4
1,07	F8A3	coagulation factor VIII-associated 3
1,07	USP17L22	ubiquitin specific peptidase 17-like family member 22
1,07	CSNK1D	casein kinase 1, delta
1,07	COL28A1	collagen, type XXVIII, alpha 1
1,07	PSMD1	proteasome 26S subunit, non-ATPase 1
1,07	PAQR3	progestin and adipoQ receptor family member III
1,07	LOC101928093; RP11-779O18.1	uncharacterized LOC101928093; novel transcript
1,07	LURAP1	leucine rich adaptor protein 1
1,07	PPP1R14A	protein phosphatase 1, regulatory (inhibitor) subunit 14A
1,07	ZDHHC7	zinc finger, DHHC-type containing 7
1,07	LZTS2	leucine zipper, putative tumor suppressor 2
1,07	DENND3	Memczak2013 ALT_ACCEPTOR, ALT_DONOR, coding, INTERNAL, intronic best transcript NM_014957

1,07	CD70	CD70 molecule
1,07	RRP12	ribosomal RNA processing 12 homolog
1,07	SLC25A34	solute carrier family 25, member 34
1,07	ADAMTS17	Memczak2013 ANTISENSE, coding, INTERNAL, intronic best transcript NM_139057
1,07	C4orf19	chromosome 4 open reading frame 19
1,07	FNDC5	fibronectin type III domain containing 5
1,07	ZBED5	zinc finger, BED-type containing 5
1,07	HIST1H2BF	histone cluster 1, H2bf
1,07	C20orf144	chromosome 20 open reading frame 144
1,07	ABCE1	ATP binding cassette subfamily E member 1
1,07	KIAA1586	KIAA1586
1,07	OR6B1	olfactory receptor, family 6, subfamily B, member 1
1,07	DIRAS3	DIRAS family, GTP-binding RAS-like 3
1,07	ZNF252P-AS1	ZNF252P antisense RNA 1
1,07	CDH10	cadherin 10, type 2 (T2-cadherin)
1,07	UCP3	uncoupling protein 3 (mitochondrial, proton carrier)
1,07	HDAC4	histone deacetylase 4
1,07	SLC39A11	solute carrier family 39, member 11
1,07	LINC01189	long intergenic non-protein coding RNA 1189
1,07	KCNT2	potassium channel, sodium activated subfamily T, member 2
1,07	INIP	INTS3 and NABP interacting protein
1,07	BOP1; MIR7112	block of proliferation 1; microRNA 7112
1,07	ZNF468	zinc finger protein 468
1,07	RAC1	ras-related C3 botulinum toxin substrate 1 (rho family, small GTP binding protein Rac1)
1,07	CCDC92	coiled-coil domain containing 92
1,07	KDR	kinase insert domain receptor
1,07	CLMP	CXADR-like membrane protein
1,07	CDKN2B	cyclin-dependent kinase inhibitor 2B (p15, inhibits CDK4)
1,07	POTEC	POTE ankyrin domain family, member C
1,07	FAM76A	family with sequence similarity 76, member A
1,07	METTL21C	methyltransferase like 21C
1,07	PACS2	phosphofurin acidic cluster sorting protein 2
1,07	SPHKAP	SPHK1 interactor, AKAP domain containing
1,07	MEF2D	myocyte enhancer factor 2D
1,07	MBL1P; BMS1P21	mannose-binding lectin (protein A) 1, pseudogene; BMS1 ribosome biogenesis factor pseudogene 21
1,07	ASPSCR1	alveolar soft part sarcoma chromosome region, candidate 1
1,07	TLR1	toll-like receptor 1
1,07	JAKMIP2	janus kinase and microtubule interacting protein 2
1,07	TRIM13; KCNRG	tripartite motif containing 13; potassium channel regulator
1,07	TRIM3	tripartite motif containing 3
1,07	SCAMP1	secretory carrier membrane protein 1

1,07	LOC79999; LOC388436; AC007952.6	Homo sapiens uncharacterized LOC79999 (LOC79999), mRNA.; Homo sapiens uncharacterized protein ENSP00000382042 (LOC388436), mRNA.; novel transcript antisense to GRAPL
1,07	PTPN12	protein tyrosine phosphatase, non-receptor type 12
1,07	CSTF2T	cleavage stimulation factor, 3 pre-RNA, subunit 2, tau variant
1,07	STAG3	stromal antigen 3
1,07	ZNF205	zinc finger protein 205
1,07	FABP9	fatty acid binding protein 9, testis
1,07	NTM	neurotrimin
1,07	C1orf229	chromosome 1 open reading frame 229
1,07	ZNF331	zinc finger protein 331
1,07	LONP1	Transcript Identified by AceView, Entrez Gene ID(s) 9361
1,07	PTPN23	protein tyrosine phosphatase, non-receptor type 23
1,07	KERA	keratocan
1,07	IMP4	IMP4 homolog, U3 small nucleolar ribonucleoprotein
1,07	WDR62	WD repeat domain 62
1,07	ANGPTL6	angiopoietin like 6
1,07	ZADH2	zinc binding alcohol dehydrogenase domain containing 2
1,07	CCL11	chemokine (C-C motif) ligand 11
1,07	PROX1	prospero homeobox 1
1,07	ZNF717	zinc finger protein 717
1,07	ZNF280A	zinc finger protein 280A
1,07	KCNMA1	Jeck2013 ALT_ACCEPTOR, ALT_DONOR, coding, INTERNAL, intronic best transcript NM_001014797
1,07	KLHDC8A	kelch domain containing 8A
1,07	EFTUD2	elongation factor Tu GTP binding domain containing 2
1,07	ZNF782	zinc finger protein 782
1,07	SSBP1	single-stranded DNA binding protein 1, mitochondrial
1,07	ZNF157	zinc finger protein 157
1,07	DBP	D site of albumin promoter (albumin D-box) binding protein
1,07	SCN2B	sodium channel, voltage gated, type II beta subunit
1,07	DEFB104A; DEFB104B	defensin, beta 104A; defensin, beta 104B
1,07	GPA33	glycoprotein A33 (transmembrane)
1,07	PIBF1	progesterone immunomodulatory binding factor 1
1,07	FIG4	FIG4 phosphoinositide 5-phosphatase
1,07	PAK1IP1	PAK1 interacting protein 1
1,07	EPGN	epithelial mitogen
1,07	ZIC5	Zic family member 5
1,07	OR10G2	olfactory receptor, family 10, subfamily G, member 2
1,07	C1QTNF2	C1q and tumor necrosis factor related protein 2
1,07	EIF3L	eukaryotic translation initiation factor 3, subunit L
1,07	CYB5R3	cytochrome b5 reductase 3
1,07	RFPL4B	ret finger protein-like 4B

1,07	ACTR3	ARP3 actin-related protein 3 homolog (yeast)
1,07	LOC100631378; AC016582.2; LOC728853	uncharacterized 100631378; Transcript Identified by AceView, Entrez Gene ID(s) 728853; novel transcript
1,07	TMEM234	transmembrane protein 234
1,07	CEP131	centrosomal protein 131kDa
1,07	XK	X-linked Kx blood group
1,07	GJD2	gap junction protein delta 2
1,07	CHAMP1	chromosome alignment maintaining phosphoprotein 1
1,07	KIRREL3	Jeck2013 ALT_ACCEPTOR, ALT_DONOR, coding, INTERNAL, intronic best transcript NM_032531
1,07	SOX18	SRY box 18
1,07	NDUFA12	NADH dehydrogenase (ubiquinone) 1 alpha subcomplex, 12
1,07	RHD	Rh blood group, D antigen
1,07	HERC3	HECT and RLD domain containing E3 ubiquitin protein ligase 3
1,07	FAM126A	family with sequence similarity 126, member A
1,07	BATF2	basic leucine zipper transcription factor, ATF-like 2
1,07	AREL1	apoptosis resistant E3 ubiquitin protein ligase 1
1,07	KIF3C	kinesin family member 3C
1,07	ZNF365	zinc finger protein 365
1,07	ZNF629	zinc finger protein 629
1,07	KRT15; MIR6510	keratin 15, type I; microRNA 6510
1,07	PPP2R3C	protein phosphatase 2, regulatory subunit B, gamma
1,07	PSORS1C1	psoriasis susceptibility 1 candidate 1
1,07	CNTNAP4	contactin associated protein-like 4
1,07	WIZ	widely interspaced zinc finger motifs
1,07	PTPN4	protein tyrosine phosphatase, non-receptor type 4 (megakaryocyte)
1,07	RAB11A	RAB11A, member RAS oncogene family
1,07	KHDRBS3	KH domain containing, RNA binding, signal transduction associated 3
1,07	LINC00483	long intergenic non-protein coding RNA 483
1,07	BTN1A1	butyrophilin, subfamily 1, member A1
1,07	VSIG1	V-set and immunoglobulin domain containing 1
1,07	KLHL18	kelch-like family member 18
1,07	ZNF473	zinc finger protein 473
1,07	ATF7; NPFF	activating transcription factor 7; neuropeptide FF-amide peptide precursor
1,07	CFAP45	cilia and flagella associated protein 45
1,07	ARL17B	ADP-ribosylation factor like GTPase 17B
1,07	RTN4IP1	reticulon 4 interacting protein 1
1,07	FAM101A	family with sequence similarity 101, member A
1,07	KIAA0355	KIAA0355
1,07	GNPNAT1	glucosamine-phosphate N-acetyltransferase 1
1,07	TRPM1	transient receptor potential cation channel, subfamily M, member 1
1,07	METTL5	methyltransferase like 5
1,07	SPTLC1	serine palmitoyltransferase, long chain base subunit 1

1,07	TRNP1	TMF1-regulated nuclear protein 1
1,07	ZDHHC6	zinc finger, DHHC-type containing 6
1,07	NYAP2	neuronal tyrosine-phosphorylated phosphoinositide-3-kinase adaptor 2
1,07	TM2D1	Transcript Identified by AceView, Entrez Gene ID(s) 83941
1,07	CCDC64B	coiled-coil domain containing 64B
1,07	PRSS50	protease, serine 50
1,07	GPR101	G protein-coupled receptor 101
1,07	INSL3	insulin-like 3 (Leydig cell)
1,07	API5	apoptosis inhibitor 5
1,07	C9orf106	chromosome 9 open reading frame 106
1,07	POC5	POC5 centriolar protein
1,07	BABAM1	Zhang2013 ALT_ACCEPTOR, ALT_DONOR, coding, INTERNAL, intronic best transcript NM_001033549
1,07	NOTCH4	notch 4
1,07	EDEM1	ER degradation enhancer, mannosidase alpha-like 1
1,07	SLC19A1	solute carrier family 19 (folate transporter), member 1
1,07	YTHDF3	YTH N(6)-methyladenosine RNA binding protein 3
1,07	NRP2	neuropilin 2
1,07	NXPH2	neurexophilin 2
1,07	NDUFA6	NADH dehydrogenase (ubiquinone) 1 alpha subcomplex, 6, 14kDa
1,07	GNG7	guanine nucleotide binding protein (G protein), gamma 7
1,07	RBM39	RNA binding motif protein 39
1,07	MAML3	mastermind-like transcriptional coactivator 3
1,07	MAP3K7CL	MAP3K7 C-terminal like
1,07	U2AF1L4	U2 small nuclear RNA auxiliary factor 1-like 4
1,07	TBC1D9	TBC1 domain family, member 9 (with GRAM domain)
1,07	ACYP2	acylphosphatase 2, muscle type
1,07	CDCA3	cell division cycle associated 3
1,07	GTF2H2C_2; GTF2H2C; GTF2H2B	GTF2H2 family member C, copy 2; GTF2H2 family member C; general transcription factor IIH subunit 2B (pseudogene)
1,07	SEZ6	seizure related 6 homolog (mouse)
1,07	OR4F21	olfactory receptor, family 4, subfamily F, member 21
1,07	KRTAP1-4	keratin associated protein 1-4
1,07	COL27A1	collagen, type XXVII, alpha 1
1,07	PPIAL4F	peptidylprolyl isomerase A (cyclophilin A)-like 4F
1,07	WISP3	WNT1 inducible signaling pathway protein 3
1,07	MUC3A	mucin 3A, cell surface associated
1,07	HADHB	hydroxyacyl-CoA dehydrogenase/3-ketoacyl-CoA thiolase/enoyl-CoA hydratase (trifunctional protein), beta subunit
1,07	BTNL8	butyrophilin-like 8
1,07	IFI35	interferon-induced protein 35
1,07	TUBA1A	tubulin, alpha 1a

1,07	MAPK10	mitogen-activated protein kinase 10
1,07	PSMC4	proteasome 26S subunit, ATPase 4 [Source:HGNC Symbol;Acc:HGNC:9551]
1,07	SNRNP35	small nuclear ribonucleoprotein, U11/U12 35kDa subunit
1,07	APOO	apolipoprotein O
1,07	SIN3B	SIN3 transcription regulator family member B
1,07	ETNPPL	ethanolamine-phosphate phospho-lyase
1,07	KRTAP4-5	keratin associated protein 4-5
1,07	DMD	dystrophin
1,07	PI4K2A	phosphatidylinositol 4-kinase type 2 alpha
1,07	TMPRSS11F	transmembrane protease, serine 11F
1,07	TBC1D3H; TBC1D3G; TBC1D3I; LOC101060389; TBC1D3C; TBC1D3	Homo sapiens TBC1 domain family, member 3H (TBC1D3H), mRNA.; Homo sapiens TBC1 domain family, member 3G (TBC1D3G), mRNA.; Homo sapiens TBC1 domain family, member 3I (TBC1D3I), mRNA.; Homo sapiens TBC1 domain family member-like (LOC101060389), mRNA.; Homo sapiens TBC1 domain family, member 3C, mRNA (cDNA clone MGC:87891 IMAGE:5165385), complete cds.; Homo sapiens TBC1 domain family, member 3, mRNA (cDNA clone MGC:87893 IMAGE:5742850), complete cds.
1,07	ANAPC4	anaphase promoting complex subunit 4
1,07	MMP26	matrix metallopeptidase 26
1,07	TRIM62	tripartite motif containing 62
1,07	DUX4L14; DUX4L15	double homeobox 4 like 14; double homeobox 4 like 15
1,07	RLBP1	retinaldehyde binding protein 1
1,07	VILL	villin-like
1,07	CACFD1	calcium channel flower domain containing 1
1,07	ZNF197	zinc finger protein 197
1,07	CHRFAM7A	CHRNA7 (cholinergic receptor, nicotinic, alpha 7, exons 5-10) and FAM7A (family with sequence similarity 7A, exons A-E) fusion
1,07	OR5D18	olfactory receptor, family 5, subfamily D, member 18
1,07	CHKB	choline kinase beta
1,07	THBS3	Salzman2013 ALT_ACCEPTOR, ALT_DONOR, coding, INTERNAL, intronic best transcript NM_007112
1,07	PHF21A	PHD finger protein 21A
1,07	FAM206A	family with sequence similarity 206, member A
1,07	LSM12	Homo sapiens LSM12 homolog (S. cerevisiae), mRNA (cDNA clone MGC:57206 IMAGE:4794614), complete cds.
1,07	FIGNL2	fidgetin-like 2
1,07	AGFG2	ArfGAP with FG repeats 2
1,07	MRC1	mannose receptor, C type 1
1,07	TM7SF2	transmembrane 7 superfamily member 2
1,07	S100PBP	S100P binding protein
1,07	BANF2	barrier to autointegration factor 2

1,07	MEIKIN	meiotic kinetochore factor
1,07	LILRB4	leukocyte immunoglobulin-like receptor, subfamily B (with TM and ITIM domains), member 4
1,07	SLC25A25	Zhang2013 ALT_ACCEPTOR, ALT_DONOR, coding, INTERNAL, intronic best transcript NM_001006641
1,07	CXCR1	chemokine (C-X-C motif) receptor 1
1,07	CEACAM19	carcinoembryonic antigen-related cell adhesion molecule 19
1,07	SPAG17	sperm associated antigen 17
1,07	GNPAT	glyceronephosphate O-acyltransferase
1,07	CYP2R1	cytochrome P450, family 2, subfamily R, polypeptide 1
1,07	TLR6	toll-like receptor 6
1,07	LY6E	lymphocyte antigen 6 complex, locus E
1,07	HAO1	hydroxyacid oxidase (glycolate oxidase) 1
1,07	CLDN9	claudin 9
1,07	KRTAP4-6	keratin associated protein 4-6
1,07	PRG4	proteoglycan 4
1,07	RHOF	ras homolog family member F (in filopodia)
1,07	SHB	Src homology 2 domain containing adaptor protein B
1,07	KIF15	kinesin family member 15
1,07	TMEM216	transmembrane protein 216
1,07	TMEM173	transmembrane protein 173
1,07	GSTM1	glutathione S-transferase mu 1
1,07	SEMA3C	sema domain, immunoglobulin domain (Ig), short basic domain, secreted, (semaphorin) 3C
1,07	SNX29P2; SNX29P1	sorting nexin 29 pseudogene 2; sorting nexin 29 pseudogene 1
1,07	LOC200726; AC010731.4	hCG1657980; Transcript Identified by AceView, Entrez Gene ID(s) 200726, RefSeq ID(s) NM_001102659; novel transcript
1,07	SP110	SP110 nuclear body protein
1,07	SNRPA	small nuclear ribonucleoprotein polypeptide A
1,07	HIGD2A	HIG1 hypoxia inducible domain family, member 2A
1,07	KLHL23	kelch-like family member 23
1,07	HS6ST2	heparan sulfate 6-O-sulfotransferase 2
1,07	FBXO30	F-box protein 30
1,07	SCML4	sex comb on midleg-like 4 (Drosophila)
1,07	EYA1	EYA transcriptional coactivator and phosphatase 1
1,07	DCP1B	decapping mRNA 1B
1,07	IL36RN	interleukin 36 receptor antagonist
1,07	RPF2	ribosome production factor 2 homolog
1,07	CDR1	cerebellar degeneration related protein 1
1,07	MIR670HG; HSD17B12	MIR670 host gene; hydroxysteroid (17-beta) dehydrogenase 12
1,07	CCDC25	coiled-coil domain containing 25
1,07	NUPL2	nucleoporin like 2
1,07	NISCH	Zhang2013 ALT_DONOR, coding, INTERNAL, intronic best transcript NM_007184

1,07	TAF8	TATA box binding protein associated factor 8
1,07	C20orf166-AS1	C20orf166 antisense RNA 1
1,07	RAB18	RAB18, member RAS oncogene family
1,07	ARHGAP1	Memczak2013 ANTISENSE, CDS, coding, INTERNAL, UTR3 best transcript NM_004308
1,07	MAGEB16	MAGE family member B16
1,07	GRAMD1C	GRAM domain containing 1C
1,07	AFMID	arylformamidase
1,07	FAM212B	family with sequence similarity 212, member B
1,07	PPIP5K2	diphosphoinositol pentakisphosphate kinase 2
1,07	LRWD1; MIR4467; MIR5090	leucine-rich repeats and WD repeat domain containing 1; microRNA 4467; microRNA 5090
1,07	MYOF	myoferlin
1,07	EPG5	ectopic P-granules autophagy protein 5 homolog (C. elegans)
1,07	CACNA2D2	calcium channel, voltage-dependent, alpha 2/delta subunit 2
1,07	ANKRD30BL	ankyrin repeat domain 30B-like
1,07	TPRG1L	tumor protein p63 regulated 1-like
1,07	HTR3A	5-hydroxytryptamine (serotonin) receptor 3A, ionotropic
1,07	ANKRD30A	ankyrin repeat domain 30A
1,07	TNPO2	transportin 2
1,07	MOCS2	molybdenum cofactor synthesis 2
1,07	ANKRD13B	ankyrin repeat domain 13B
1,07	PCSK9	proprotein convertase subtilisin/kexin type 9
1,07	KRCC1	lysine-rich coiled-coil 1
1,07	CYP27B1	cytochrome P450, family 27, subfamily B, polypeptide 1
1,07	GABRA5	gamma-aminobutyric acid (GABA) A receptor, alpha 5
1,07	MFAP3	microfibrillar associated protein 3
1,07	C2orf74	chromosome 2 open reading frame 74
1,07	FCRL1	Fc receptor-like 1
1,07	PCSK5	proprotein convertase subtilisin/kexin type 5
1,07	TG	thyroglobulin
1,07	ENO2	enolase 2 (gamma, neuronal)
1,07	RAB39A	RAB39A, member RAS oncogene family
1,07	PDLIM1	PDZ and LIM domain 1
1,07	GIF	gastric intrinsic factor (vitamin B synthesis)
1,07	ZNF548	zinc finger protein 548
1,07	TMEM63A	transmembrane protein 63A
1,07	XRCC5	Memczak2013 ALT_ACCEPTOR, ALT_DONOR, coding, INTERNAL, intronic best transcript NM_021141
1,07	JAM3	junctional adhesion molecule 3
1,07	HNF1A	HNF1 homeobox A
1,07	IST1	increased sodium tolerance 1 homolog (yeast)
1,07	SPINK5	serine peptidase inhibitor, Kazal type 5

1,07	UGDH	UDP-glucose 6-dehydrogenase
1,07	KCNK1	potassium channel, two pore domain subfamily K, member 1
1,07	PAGE2B	P antigen family, member 2B
1,07	NPC2; MIR4709	Niemann-Pick disease, type C2; microRNA 4709
1,07	LRIG1	
1,07	METTL1	methyltransferase like 1
1,07	C15orf41	chromosome 15 open reading frame 41
1,07	PIM3	Pim-3 proto-oncogene, serine/threonine kinase
1,07	HMGA1	high mobility group AT-hook 1
1,07	SMAD5	SMAD family member 5
1,07	CD28	CD28 molecule
1,07	SPINK2	serine peptidase inhibitor, Kazal type 2 (acrosin-trypsin inhibitor)
1,07	DDX31	DEAD (Asp-Glu-Ala-Asp) box polypeptide 31
1,07	TMEM109	transmembrane protein 109
1,07	GTPBP4	GTP binding protein 4
1,07	OR5D16	olfactory receptor, family 5, subfamily D, member 16
1,07	DEDD	death effector domain containing
1,07	CABP5	calcium binding protein 5
1,07	TAPBP	TAP binding protein (tapasin)
1,07	CRLF2	cytokine receptor-like factor 2
1,07	CRYL1	crystallin lambda 1
1,07	AC009133.12; PAGR1	novel transcript, antisense to C16orf53 and MVP; PAXIP1 associated glutamate-rich protein 1 [Source:HGNC Symbol;Acc:HGNC:28707]
1,07	DNER	delta/notch like EGF repeat containing
1,07	PLS1	plastin 1
1,07	MTG1	mitochondrial ribosome-associated GTPase 1
1,07	PRKAR2A	protein kinase, cAMP-dependent, regulatory, type II, alpha
1,07	TUBB3; MC1R	tubulin, beta 3 class III; melanocortin 1 receptor (alpha melanocyte stimulating hormone receptor)
1,07	TRIM34	tripartite motif containing 34
1,07	PLEKHG4B	Zhang2013 ALT_ACCEPTOR, ALT_DONOR, coding, INTERNAL, intronic best transcript NM_052909
1,07	H1FNT	H1 histone family, member N, testis-specific
1,07	BPIFA3	BPI fold containing family A, member 3
1,07	THEMIS	thymocyte selection associated
1,07	TMEM253	transmembrane protein 253
1,07	ZNF251	zinc finger protein 251
1,07	AFF3	Memczak2013 ALT_ACCEPTOR, ALT_DONOR, coding, INTERNAL, intronic best transcript NM_001025108
1,07	TRAF3IP3	TRAF3 interacting protein 3
1,07	SEZ6L	seizure related 6 homolog (mouse)-like
1,07	MAK16	MAK16 homolog
1,07	WASF1	WAS protein family, member 1
1,07	ZNF738	zinc finger protein 738

1,07	ZKSCAN7	zinc finger with KRAB and SCAN domains 7
1,07	CXorf40B	chromosome X open reading frame 40B
1,07	PRAMEF6; PRAMEF5	PRAME family member 6; PRAME family member 5
1,07	MORC3	MORC family CW-type zinc finger 3
1,07	C11orf72	chromosome 11 open reading frame 72
1,07	DNTT	Transcript Identified by AceView, Entrez Gene ID(s) 1791
1,07	RNF223	ring finger protein 223
1,07	CTAG2	cancer/testis antigen 2
1,07	KIAA1024	KIAA1024
1,07	KIAA1551	KIAA1551
1,07	MED1	mediator complex subunit 1
1,07	RAD54L	Transcript Identified by AceView, Entrez Gene ID(s) 8438
1,07	KBTBD4	kelch repeat and BTB (POZ) domain containing 4
1,07	TBP	TATA box binding protein
1,07	TENM1	teneurin transmembrane protein 1
1,07	MYCL	v-myc avian myelocytomatosis viral oncogene lung carcinoma derived homolog
1,07	HES5	hes family bHLH transcription factor 5
1,07	KRTAP4-2	keratin associated protein 4-2
1,07	DLL1	delta-like 1 (Drosophila)
1,07	C12orf4	chromosome 12 open reading frame 4
1,07	SMIM18	small integral membrane protein 18
1,07	ATP1A1	ATPase, Na+/K+ transporting, alpha 1 polypeptide
1,07	ACTR1A	ARP1 actin-related protein 1 homolog A, centractin alpha (yeast)
1,07	NEK10	NIMA-related kinase 10
1,07	DCDC1	doublecortin domain containing 1
1,07	MSRA	methionine sulfoxide reductase A
1,07	MAGOH	mago homolog, exon junction complex core component
1,07	GPR158-AS1	GPR158 antisense RNA 1
1,07	CENPT	centromere protein T
1,07	CTDNEP1	CTD nuclear envelope phosphatase 1
1,07	FBXO38	F-box protein 38
1,07	CEBPE	CCAAT/enhancer binding protein (C/EBP), epsilon
1,06	RPL18A; SNORA68	ribosomal protein L18a; small nucleolar RNA, H/ACA box 68
1,06	ARSG	arylsulfatase G
1,06	LYPD5	LY6/PLAUR domain containing 5
1,06	DRGX	dorsal root ganglia homeobox
1,06	CTDSP2	CTD small phosphatase 2
1,06	KANSL3	KAT8 regulatory NSL complex subunit 3
1,06	ANKS1B	ankyrin repeat and sterile alpha motif domain containing 1B
1,06	FAM183A	family with sequence similarity 183, member A
1,06	USP53	ubiquitin specific peptidase 53
1,06	KIF15	kinesin family member 15

1,06	RAB3GAP2; AURKAPS1	RAB3 GTPase activating protein subunit 2 (non-catalytic); aurora kinase A pseudogene 1
1,06	SLC4A5	solute carrier family 4 (sodium bicarbonate cotransporter), member 5
1,06	APTX	aprataxin
1,06	ZNF530	zinc finger protein 530
1,06	ZNF227	zinc finger protein 227
1,06	C2CD2L	C2CD2-like
1,06	PNMA3	paraneoplastic Ma antigen 3
1,06	ZNF385B	zinc finger protein 385B
1,06	ZNF445	zinc finger protein 445
1,06	PI16	peptidase inhibitor 16
1,06	PEPD	peptidase D
1,06	TXNRD1	thioredoxin reductase 1
1,06	VTA1	vesicle (multivesicular body) trafficking 1
1,06	OXSR1	oxidative stress responsive 1
1,06	CHMP2B	charged multivesicular body protein 2B
1,06	TSPAN33	tetraspanin 33
1,06	ALB	albumin
1,06	FAM189A1	family with sequence similarity 189, member A1
1,06	NDUFAF3	NADH dehydrogenase (ubiquinone) complex I, assembly factor 3
1,06	TARBP2	TAR (HIV-1) RNA binding protein 2
1,06	RNF220	Memczak2013 ANTISENSE, coding, INTERNAL, intronic best transcript NM_018150
1,06	GALNT3	polypeptide N-acetylgalactosaminyltransferase 3
1,06	TESK1; MIR4667	testis-specific kinase 1; microRNA 4667
1,06	CEACAM6	carcinoembryonic antigen-related cell adhesion molecule 6 (non-specific cross reacting antigen)
1,06	LECT2	leukocyte cell-derived chemotaxin 2
1,06	FOXB1	forkhead box B1
1,06	EXOC3L1	exocyst complex component 3-like 1
1,06	LRRC49	leucine rich repeat containing 49
1,06	CBX2	chromobox homolog 2
1,06	LARP7	Zhang2013 ALT_DONOR, coding, INTERNAL, intronic, OVERLAPTX, OVEXON best transcript NM_016648
1,06	EME2	essential meiotic structure-specific endonuclease subunit 2
1,06	UBE2F-SCLY	UBE2F-SCLY readthrough (NMD candidate)
1,06	UEVLD	UEV and lactate/malate dehyrogenase domains
1,06	MRPL37	mitochondrial ribosomal protein L37
1,06	LTBR	lymphotoxin beta receptor (TNFR superfamily, member 3)
1,06	WDR45; PRAF2	WD repeat domain 45; PRA1 domain family, member 2
1,06	S100A8	S100 calcium binding protein A8
1,06	TTC26	tetratricopeptide repeat domain 26
1,06	HUS1	HUS1 checkpoint clamp component
1,06	MLPH	melanophilin

1,06	SMCO2	single-pass membrane protein with coiled-coil domains 2
1,06	TERF1	telomeric repeat binding factor (NIMA-interacting) 1
1,06	HIST1H2AM; HIST1H3J	histone cluster 1, H2am; histone cluster 1, H3j
1,06	KCNC2	potassium channel, voltage gated Shaw related subfamily C, member 2
1,06	HGS	hepatocyte growth factor-regulated tyrosine kinase substrate
1,06	RFWD3	ring finger and WD repeat domain 3
1,06	ARNT	aryl hydrocarbon receptor nuclear translocator
1,06	SLC2A6	solute carrier family 2 (facilitated glucose transporter), member 6 [Source:HGNC Symbol;Acc:HGNC:11011]
1,06	LOC100505549; RP11-35G9.3	uncharacterized LOC100505549; Salzman2013 ANNOTATED, CDS, coding, OVCODE, OVERLAPTX, OVEXON, UTR3, UTR5 best transcript NM_001242804; novel transcript, antisense to ATP8B1
1,06	WDR61	WD repeat domain 61
1,06	DCAF17	DDB1 and CUL4 associated factor 17
1,06	UBIAD1	UbiA prenyltransferase domain containing 1
1,06	WASF3	WAS protein family, member 3
1,06	CDC16	cell division cycle 16
1,06	TBC1D21	TBC1 domain family, member 21
1,06	SH3KBP1	SH3-domain kinase binding protein 1
1,06	SHMT2	serine hydroxymethyltransferase 2 (mitochondrial)
1,06	ZNF34	zinc finger protein 34
1,06	SLC7A11	solute carrier family 7 (anionic amino acid transporter light chain, xc- system), member 11
1,06	GNL2	guanine nucleotide binding protein-like 2 (nucleolar)
1,06	ARNT2; MIR5572	aryl-hydrocarbon receptor nuclear translocator 2; microRNA 5572
1,06	ANO1	anoctamin 1, calcium activated chloride channel
1,06	CD6	CD6 molecule
1,06	TPD52L2	tumor protein D52-like 2
1,06	PLAA	phospholipase A2-activating protein
1,06	POTEB2; POTEB; POTEB3	POTE ankyrin domain family, member B2; POTE ankyrin domain family, member B; POTE ankyrin domain family, member B3
1,06	ZNF200	zinc finger protein 200
1,06	C4BPB	complement component 4 binding protein, beta
1,06	ZMIZ2	zinc finger, MIZ-type containing 2
1,06	MPLKIP	M-phase specific PLK1 interacting protein
1,06	RNASE8	ribonuclease, RNase A family, 8
1,06	DTD2	D-tyrosyl-tRNA deacylase 2 (putative)
1,06	TBC1D32	TBC1 domain family, member 32
1,06	OR8G1	olfactory receptor, family 8, subfamily G, member 1 (gene/pseudogene)
1,06	PTTG2	pituitary tumor-transforming 2
1,06	IL1F10	interleukin 1 family, member 10 (theta)

1,06	DYNLRB1	dynein, light chain, roadblock-type 1
1,06	CLINT1	clathrin interactor 1
1,06	NPY	neuropeptide Y
1,06	SCN4A	sodium channel, voltage gated, type IV alpha subunit
1,06	CSH1	chorionic somatomammotropin hormone 1 (placental lactogen)
1,06	FUBP3	Transcript Identified by AceView, Entrez Gene ID(s) 8939
1,06	FAM155B	family with sequence similarity 155, member B
1,06	PTK2	Transcript Identified by AceView, Entrez Gene ID(s) 5747
1,06	DDIT3	DNA-damage-inducible transcript 3
1,06	NCOA4	nuclear receptor coactivator 4
1,06	COMMD8	COMM domain containing 8
1,06	DDX49	DEAD (Asp-Glu-Ala-Asp) box polypeptide 49
1,06	CEP104	centrosomal protein 104kDa
1,06	GRIK4	glutamate receptor, ionotropic, kainate 4
1,06	DHDDS	dehydrodolichyl diphosphate synthase subunit
1,06	MYOG	myogenin (myogenic factor 4)
1,06	ASTL	astacin-like metallo-endopeptidase (M12 family)
1,06	IPCEF1	interaction protein for cytohesin exchange factors 1
1,06	DHCR7	7-dehydrocholesterol reductase
1,06	LOC100133091	uncharacterized LOC100133091; Salzman2013 ANNOTATED, INTERNAL, ncRNA, OVEXON best transcript NR_029411; Transcript Identified by AceView, Entrez Gene ID(s) 554248; 100133091, RefSeq ID(s) NR_029411
1,06	ADCY5	adenylate cyclase 5
1,06	GPATCH11	G-patch domain containing 11
1,06	TPM3	tropomyosin 3
1,06	TFEC	transcription factor EC
1,06	MYH9	Zhang2013 ALT_ACCEPTOR, ALT_DONOR, coding, INTERNAL, intronic best transcript NM_002473
1,06	RAB11FIP3	RAB11 family interacting protein 3 (class II)
1,06	APOBEC3H	apolipoprotein B mRNA editing enzyme, catalytic polypeptide-like 3H
1,06	TOP1MT	topoisomerase (DNA) I, mitochondrial
1,06	ZNF512	Memczak2013 ANTISENSE, coding, INTERNAL, UTR3 best transcript NM_032434
1,06	ARRDC1-AS1	ARRDC1 antisense RNA 1
1,06	TSPAN2	tetraspanin 2
1,06	GOLGA6L2	golgin A6 family-like 2
1,06	MAPKAPK3	mitogen-activated protein kinase-activated protein kinase 3
1,06	G3BP2	GTPase activating protein (SH3 domain) binding protein 2
1,06	SLX1A; SLX1B	SLX1 homolog A, structure-specific endonuclease subunit; SLX1 homolog B, structure-specific endonuclease subunit
1,06	VWA5B2	von Willebrand factor A domain containing 5B2

1,06	ARAP2	ArfGAP with RhoGAP domain, ankyrin repeat and PH domain 2
1,06	WBP11	WW domain binding protein 11
1,06	RHAG	Rh-associated glycoprotein
1,06	SLC38A10	solute carrier family 38, member 10
1,06	CYP20A1	cytochrome P450, family 20, subfamily A, polypeptide 1
1,06	ZNF136	zinc finger protein 136
1,06	RAB24	RAB24, member RAS oncogene family
1,06	HMBS	hydroxymethylbilane synthase
1,06	IL36A	interleukin 36, alpha
1,06	SDE2	SDE2 telomere maintenance homolog (S. pombe)
1,06	EAF2	ELL associated factor 2
1,06	IDS	iduronate 2-sulfatase
1,06	TVP23C; CDRT4; TVP23C-CDRT4	trans-golgi network vesicle protein 23 homolog C (S. cerevisiae); CMT1A duplicated region transcript 4; TVP23C-CDRT4 readthrough
1,06	MOGAT3	monoacylglycerol O-acyltransferase 3
1,06	HSPH1	heat shock 105kDa/110kDa protein 1
1,06	FAM71B	family with sequence similarity 71, member B
1,06	DISP1	dispatched homolog 1 (Drosophila)
1,06	CYB5B	cytochrome b5 type B (outer mitochondrial membrane)
1,06	BPI	bactericidal/permeability-increasing protein
1,06	BCL3; MIR8085	B-cell CLL/lymphoma 3; microRNA 8085
1,06	MAGEC3	MAGE family member C3
1,06	CFAP69	cilia and flagella associated protein 69
1,06	FAM19A5	family with sequence similarity 19 (chemokine (C-C motif)-like), member A5
1,06	PRUNE	prune exopolyphosphatase
1,06	WDR88	WD repeat domain 88
1,06	SH2D5	SH2 domain containing 5
1,06	PSMB11	proteasome (prosome, macropain) subunit, beta type, 11
1,06	IRF4	interferon regulatory factor 4
1,06	ZNF333	zinc finger protein 333
1,06	LACTB	lactamase, beta
1,06	TAS2R7	taste receptor, type 2, member 7
1,06	SSX5	synovial sarcoma, X breakpoint 5
1,06	CATIP	ciliogenesis associated TTC17 interacting protein
1,06	NDUFAF1	NADH dehydrogenase (ubiquinone) complex I, assembly factor 1
1,06	CCDC79	coiled-coil domain containing 79
1,06	MARCKS	myristoylated alanine-rich protein kinase C substrate
1,06	GHRL	ghrelin/obestatin prepropeptide
1,06	PROK1	prokineticin 1
1,06	ZNF568	zinc finger protein 568
1,06	HNRNPM	heterogeneous nuclear ribonucleoprotein M
1,06	GGT5	gamma-glutamyltransferase 5
1,06	SERINC1	serine incorporator 1

1,06	DAB2	Dab, mitogen-responsive phosphoprotein, homolog 2 (Drosophila)
1,06	DNAJC8	DnaJ (Hsp40) homolog, subfamily C, member 8
1,06	NDUFS5	NADH dehydrogenase (ubiquinone) Fe-S protein 5, 15kDa (NADH-coenzyme Q reductase)
1,06	ERGIC2	ERGIC and golgi 2
1,06	HSPA4	heat shock 70kDa protein 4
1,06	SCRT1	scratch family zinc finger 1
1,06	TMEM33	transmembrane protein 33
1,06	KAT6B	K(lysine) acetyltransferase 6B
1,06	C14orf180	chromosome 14 open reading frame 180
1,06	C9orf43	chromosome 9 open reading frame 43
1,06	RNF4	ring finger protein 4
1,06	SLC6A8	solute carrier family 6 (neurotransmitter transporter), member 8
1,06	AGAP1; noygloy; torkeybo	Jeck2013 ALT_ACCEPTOR, ALT_DONOR, coding, INTERNAL, intronic best transcript NM_001037131; Transcript Identified by AceView
1,06	CNN2	calponin 2
1,06	OS9	osteosarcoma amplified 9, endoplasmic reticulum lectin
1,06	CLPX	caseinolytic mitochondrial matrix peptidase chaperone subunit
1,06	PRPSAP2	phosphoribosyl pyrophosphate synthetase-associated protein 2
1,06	ZC3H7B	zinc finger CCCH-type containing 7B
1,06	RFX2	regulatory factor X, 2 (influences HLA class II expression)
1,06	ATP5D	ATP synthase, H+ transporting, mitochondrial F1 complex, delta subunit
1,06	C20orf197	chromosome 20 open reading frame 197
1,06	RHNO1	RAD9-HUS1-RAD1 interacting nuclear orphan 1
1,06	OR2M2	olfactory receptor, family 2, subfamily M, member 2
1,06	CDC123	cell division cycle 123
1,06	TTC38	tetratricopeptide repeat domain 38
1,06	GBP6	guanylate binding protein family, member 6
1,06	ZNF519	zinc finger protein 519
1,06	CHIT1	chitinase 1 (chitotriosidase)
1,06	GATS	GATS, stromal antigen 3 opposite strand
1,06	MALT1	MALT1 paracaspase
1,06	AGBL2	ATP/GTP binding protein-like 2
1,06	TMEM55A	transmembrane protein 55A
1,06	SELENBP1	selenium binding protein 1
1,06	DDX21	DEAD (Asp-Glu-Ala-Asp) box helicase 21
1,06	NDFIP2	Nedd4 family interacting protein 2
1,06	DPF1	D4, zinc and double PHD fingers family 1
1,06	WFDC11	WAP four-disulfide core domain 11
1,06	OR4C46	olfactory receptor, family 4, subfamily C, member 46
1,06	PRAM1	PML-RARA regulated adaptor molecule 1
1,06	CCDC33	coiled-coil domain containing 33

1,06	MCF2L	MCF.2 cell line derived transforming sequence like
1,06	TLR1	toll-like receptor 1
1,06	C22orf29; GNB1L	chromosome 22 open reading frame 29; guanine nucleotide binding protein (G protein), beta polypeptide 1-like
1,06	ZNF296	zinc finger protein 296
1,06	ZNF573	zinc finger protein 573
1,06	SPDYE6; SPDYE2	speedy/RINGO cell cycle regulator family member E6; speedy/RINGO cell cycle regulator family member E2
1,06	HOXB7	homeobox B7
1,06	TBL1X	transducin (beta)-like 1X-linked
1,06	NGFR	nerve growth factor receptor
1,06	AKNA	AT-hook transcription factor
1,06	IL26	interleukin 26
1,06	SLC37A3	solute carrier family 37, member 3
1,06	MTA3	Transcript Identified by AceView, Entrez Gene ID(s) 57504
1,06	DCAF16	DDB1 and CUL4 associated factor 16
1,06	DCDC1	doublecortin domain containing 1
1,06	ATCAY	ataxia, cerebellar, Cayman type
1,06	PPP3CB	protein phosphatase 3, catalytic subunit, beta isozyme
1,06	SRP54	signal recognition particle 54kDa
1,06	GRAPL	GRB2-related adaptor protein-like
1,06	PLPPR2	phospholipid phosphatase related 2
1,06	NR1I3	nuclear receptor subfamily 1, group I, member 3
1,06	AADACL3	arylacetamide deacetylase-like 3
1,06	KLHL28	kelch-like family member 28
1,06	JRK	Jrk helix-turn-helix protein
1,06	IL3	interleukin 3
1,06	CNOT4	CCR4-NOT transcription complex subunit 4
1,06	TPRKB	TP53RK binding protein
1,06	GCG	glucagon
1,06	NHP2	NHP2 ribonucleoprotein
1,06	SNW1	SNW domain containing 1
1,06	CSDE1	cold shock domain containing E1, RNA binding
1,06	TOB1	transducer of ERBB2, 1
1,06	G6PC	glucose-6-phosphatase, catalytic subunit
1,06	WIPI1	WD repeat domain, phosphoinositide interacting 1
1,06	P2RY13	purinergic receptor P2Y, G-protein coupled, 13
1,06	PRKACG	protein kinase, cAMP-dependent, catalytic, gamma
1,06	DMRTC1; FAM226B; FAM226A; DMRTC1B	DMRT-like family C1; family with sequence similarity 226, member B (non-protein coding); family with sequence similarity 226, member A (non-protein coding); DMRT-like family C1B
1,06	ELP5	elongator acetyltransferase complex subunit 5
1,06	DSCC1	DNA replication and sister chromatid cohesion 1
1,06	SEZ6L2	seizure related 6 homolog (mouse)-like 2
1,06	BLCAP	bladder cancer associated protein
1,06	TMEM53	transmembrane protein 53

1,06	CADPS	Ca++-dependent secretion activator
1,06	KIT	v-kit Hardy-Zuckerman 4 feline sarcoma viral oncogene homolog
1,06	WDR77	WD repeat domain 77
1,06	GFRA4	GDNF family receptor alpha 4
1,06	PFKFB4; MIR6823	6-phosphofructo-2-kinase/fructose-2,6-biphosphatase 4; microRNA 6823
1,06	LRP3	LDL receptor related protein 3
1,06	KPNA1	karyopherin alpha 1
1,06	PHOX2B	paired-like homeobox 2b
1,06	SACM1L	SAC1 suppressor of actin mutations 1-like (yeast)
1,06	NFKBIA	nuclear factor of kappa light polypeptide gene enhancer in B-cells inhibitor, alpha
1,06	PI4KB	phosphatidylinositol 4-kinase, catalytic, beta
1,06	NUDT6	nudix hydrolase 6
1,06	OSCP1	organic solute carrier partner 1
1,06	NPM1	nucleophosmin (nucleolar phosphoprotein B23, numatrin)
1,06	LAMC2	laminin, gamma 2
1,06	SLC35E3	solute carrier family 35, member E3
1,06	MARCH7	membrane associated ring finger 7
1,06	KCNV1	potassium channel, voltage gated modifier subfamily V, member 1
1,06	FAM25G; FAM25C; FAM25BP	family with sequence similarity 25, member G; family with sequence similarity 25, member C; protein FAM25
1,06	TNFSF8	tumor necrosis factor (ligand) superfamily, member 8
1,06	TTC19	tetratricopeptide repeat domain 19
1,06	NEXN	nexilin (F actin binding protein)
1,06	RAD54L	RAD54-like (S. cerevisiae)
1,06	CLEC4F	C-type lectin domain family 4, member F
1,06	BAG2	BCL2-associated athanogene 2
1,06	ZKSCAN5	zinc finger with KRAB and SCAN domains 5
1,06	HAS2	hyaluronan synthase 2
1,06	HOXB13	homeobox B13
1,06	TREML2	triggering receptor expressed on myeloid cells-like 2
1,06	CTRB2	chymotrypsinogen B2
1,06	NUTM2D	NUT family member 2D
1,06	VPS13C	Memczak2013 ANTISENSE, coding, INTERNAL, intronic best transcript NM_020821
1,06	FTH1	ferritin, heavy polypeptide 1
1,06	BRAT1	BRCA1-associated ATM activator 1
1,06	FAM98A	family with sequence similarity 98, member A
1,06	OSBPL2	oxysterol binding protein-like 2
1,06	BCDIN3D	BCDIN3 domain containing
1,06	TM6SF1	transmembrane 6 superfamily member 1
1,06	ZBTB16	zinc finger and BTB domain containing 16
1,06	IL2RG	interleukin 2 receptor, gamma
1,06	TBCK	TBC1 domain containing kinase
1,06	CDHR3	cadherin-related family member 3

1,06	TSPAN13	tetraspanin 13
1,06	CLN6	ceroid-lipofuscinosis, neuronal 6, late infantile, variant
1,06	CSNK1E	casein kinase 1, epsilon
1,06	SPARC	secreted protein, acidic, cysteine-rich (osteonectin)
1,06	MFSD6L	major facilitator superfamily domain containing 6-like
1,06	RAB7A	RAB7A, member RAS oncogene family
1,06	NEK1	NIMA-related kinase 1
1,06	C16orf13	chromosome 16 open reading frame 13
1,06	MAGEB5	MAGE family member B5
1,06	RSPO4	R-spondin 4
1,06	CALML5	calmodulin-like 5
1,06	FAM131A	family with sequence similarity 131, member A
1,06	ACTL7A	actin-like 7A
1,06	PAX4	paired box 4
1,06	ALKBH2	alkB homolog 2, alpha-ketoglutarate-dependent dioxygenase
1,06	LHX8	LIM homeobox 8
1,06	TRAP1	TNF receptor-associated protein 1
1,06	TBCE	tubulin folding cofactor E
1,06	CIB4	calcium and integrin binding family member 4
1,06	TMEM247	transmembrane protein 247
1,06	SMAD9	SMAD family member 9
1,06	OR52A5	olfactory receptor, family 52, subfamily A, member 5
1,06	TMEM175	transmembrane protein 175
1,06	RNF5	ring finger protein 5, E3 ubiquitin protein ligase
1,06	NFKBIB	nuclear factor of kappa light polypeptide gene enhancer in B-cells inhibitor, beta
1,06	FKBP1B	FK506 binding protein 1B
1,06	PGBD4	piggyBac transposable element derived 4
1,06	LHX9	LIM homeobox 9
1,06	RASIP1	Ras interacting protein 1
1,06	FCN1	ficolin (collagen/fibrinogen domain containing) 1
1,06	SPRR2B	small proline-rich protein 2B
1,06	WWP1	WW domain containing E3 ubiquitin protein ligase 1
1,06	CAV1	caveolin 1
1,06	KCNK7	potassium channel, two pore domain subfamily K, member 7
1,06	TCEB3CL	transcription elongation factor B polypeptide 3C-like
1,06	HMBS	hydroxymethylbilane synthase
1,06	XDH	xanthine dehydrogenase
1,06	RHCG	Rh family, C glycoprotein
1,06	OR2T10	olfactory receptor, family 2, subfamily T, member 10
1,06	FTSJ1	FtsJ RNA methyltransferase homolog 1 (E. coli)
1,06	PTPN13	Transcript Identified by AceView, Entrez Gene ID(s) 5783
1,06	XAGE1B; XAGE1E	X antigen family, member 1B; X antigen family, member 1E
1,06	HSPBP1	HSPA (heat shock 70kDa) binding protein, cytoplasmic cochaperone 1

1,06	RGPD4	RANBP2-like and GRIP domain containing 4
1,06	RPL18A	ribosomal protein L18a
1,06	STARD4	StAR-related lipid transfer domain containing 4
1,06	ATP10B	ATPase, class V, type 10B
1,06	LIPH	lipase, member H
1,06	RHOT1	ras homolog family member T1
1,06	TMEM185B	transmembrane protein 185B
1,06	HSPA8; SNORD14C; SNORD14D	heat shock 70kDa protein 8; small nucleolar RNA, C/D box 14C; small nucleolar RNA, C/D box 14D
1,06	FDFT1	farnesyl-diphosphate farnesyltransferase 1
1,06	PPIL1	peptidylprolyl isomerase (cyclophilin)-like 1
1,06	C3orf56	chromosome 3 open reading frame 56
1,06	SMIM23	small integral membrane protein 23
1,06	LIMD2	LIM domain containing 2
1,06	ERMAP	erythroblast membrane-associated protein (Scianna blood group)
1,06	OAS3	2-5-oligoadenylate synthetase 3
1,06	TMEM132B	transmembrane protein 132B
1,06	PIGZ	phosphatidylinositol glycan anchor biosynthesis class Z
1,06	SPTLC3	serine palmitoyltransferase, long chain base subunit 3
1,06	EEF1E1	eukaryotic translation elongation factor 1 epsilon 1
1,06	ATG16L2	autophagy related 16-like 2
1,06	TMEM69	transmembrane protein 69
1,06	PILRB; STAG3L5P; PVRIG2P; MIR6840; STAG3L5P-PVRIG2P-PILRB	paired immunoglobin-like type 2 receptor beta; stromal antigen 3-like 5 pseudogene; poliovirus receptor related immunoglobulin domain containing 2, pseudogene; microRNA 6840; STAG3L5P-PVRIG2P-PILRB readthrough
1,06	ZNF182	zinc finger protein 182
1,06	DHX58	DEXH (Asp-Glu-X-His) box polypeptide 58
1,06	SPSB4	splA/ryanodine receptor domain and SOCS box containing 4
1,06	ZDHHC11	Transcript Identified by AceView, Entrez Gene ID(s) 79844
1,06	LAP3	leucine aminopeptidase 3
1,06	ZNF567	zinc finger protein 567
1,06	C12orf43	chromosome 12 open reading frame 43
1,06	MAP3K14	mitogen-activated protein kinase kinase kinase 14
1,06	VKORC1L1	vitamin K epoxide reductase complex subunit 1 like 1
1,06	CDC27	cell division cycle 27
1,06	SMIM15	small integral membrane protein 15
1,06	SMPD4	sphingomyelin phosphodiesterase 4, neutral membrane (neutral sphingomyelinase-3)
1,06	CBLB	Cbl proto-oncogene B, E3 ubiquitin protein ligase
1,06	AOAH	acyloxyacyl hydrolase (neutrophil)
1,06	RBPJ	recombination signal binding protein for immunoglobulin kappa J region

1,06	HSPA1L	heat shock 70kDa protein 1-like
1,06	ATG16L1	autophagy related 16-like 1
1,06	VGLL1	vestigial-like family member 1
1,06	SBNO2	strawberry notch homolog 2 (Drosophila)
1,06	MTRNR2L5	MT-RNR2-like 5
1,06	CEP290	centrosomal protein 290kDa
1,06	OR4C5	olfactory receptor, family 4, subfamily C, member 5 (gene/pseudogene)
1,06	CCNL1	cyclin L1
1,06	C1GALT1	core 1 synthase, glycoprotein-N-acetylgalactosamine 3-beta-galactosyltransferase 1
1,06	LUC7L	LUC7-like
1,06	TSTD3	thiosulfate sulfurtransferase (rhodanese)-like domain containing 3
1,06	MBIP	MAP3K12 binding inhibitory protein 1
1,06	ACOT4	acyl-CoA thioesterase 4
1,06	LIN28B	lin-28 homolog B (C. elegans)
1,06	RBM34	RNA binding motif protein 34
1,06	EMP1	epithelial membrane protein 1
1,06	PRSS37	protease, serine, 37
1,06	MIF4GD	MIF4G domain containing
1,06	SERTAD2	SERTA domain containing 2
1,06	MLLT3	myeloid/lymphoid or mixed-lineage leukemia; translocated to, 3
1,06	PDP1	pyruvate dehyrogenase phosphatase catalytic subunit 1
1,06	AWAT2	acyl-CoA wax alcohol acyltransferase 2
1,06	GSC2	goosecoid homeobox 2
1,06	SDC4	syndecan 4
1,06	REXO4	REX4 homolog, 3'-5' exonuclease [Source:HGNC Symbol;Acc:HGNC:12820]
1,06	SYTL1	synaptotagmin like 1
1,06	SPSB3	splA/ryanodine receptor domain and SOCS box containing 3
1,06	ZNF200	Transcript Identified by AceView, Entrez Gene ID(s) 7752
1,06	MDC1	mediator of DNA-damage checkpoint 1
1,06	FXYD7	FXYD domain containing ion transport regulator 7
1,06	SMYD4	SET and MYND domain containing 4
1,06	MZT1	mitotic spindle organizing protein 1
1,06	HSPA12B	heat shock 70kD protein 12B
1,06	ZNF790	zinc finger protein 790
1,06	REM2	RAS (RAD and GEM)-like GTP binding 2
1,06	HNRNPK	Zhang2013 ALT_ACCEPTOR, ALT_DONOR, coding, INTERNAL, intronic, OVERLAPTX best transcript NM_002140
1,06	SWT1	SWT1 RNA endoribonuclease homolog
1,06	NUDCD1	NudC domain containing 1
1,06	ADIPOR2	adiponectin receptor 2

1,06	TNFRSF21	tumor necrosis factor receptor superfamily, member 21
1,06	PPP1R3A	protein phosphatase 1, regulatory subunit 3A
1,06	ZNF337	zinc finger protein 337
1,06	SMARCAL1	SWI/SNF related, matrix associated, actin dependent regulator of chromatin, subfamily a-like 1
1,06	IFNGR1	interferon gamma receptor 1
1,06	UMAD1	UBAP1-MVB12-associated (UMA) domain containing 1
1,06	BEND6	BEN domain containing 6
1,06	SARS2	seryl-tRNA synthetase 2, mitochondrial
1,06	PGR	progesterone receptor
1,06	NANOGNB	NANOG neighbor homeobox
1,06	EPDR1	ependymin related 1
1,06	ZNF214	zinc finger protein 214
1,06	AKR7A2	aldo-keto reductase family 7, member A2
1,06	MAGEA6	MAGE family member A6
1,06	FGF5	fibroblast growth factor 5
1,06	FMO5	flavin containing monooxygenase 5
1,06	CCR9	chemokine (C-C motif) receptor 9
1,06	ITGAM	Transcript Identified by AceView, Entrez Gene ID(s) 3684
1,06	PTPRU	protein tyrosine phosphatase, receptor type, U
1,06	SLC9A6	solute carrier family 9, subfamily A (NHE6, cation proton antiporter 6), member 6
1,06	HSH2D	hematopoietic SH2 domain containing
1,06	CCDC61	coiled-coil domain containing 61
1,06	SLC23A3	solute carrier family 23, member 3
1,06	ZPLD1	zona pellucida-like domain containing 1
1,06	COX1	cytochrome c oxidase subunit I
1,06	GRK7	G protein-coupled receptor kinase 7
1,06	FADS6	fatty acid desaturase 6
1,06	LONRF1	LON peptidase N-terminal domain and ring finger 1
1,06	C1orf131	chromosome 1 open reading frame 131
1,06	SIGLEC8	sialic acid binding Ig-like lectin 8
1,06	CARHSP1	calcium regulated heat stable protein 1
1,06	ZMYND12	zinc finger, MYND-type containing 12
1,06	HMGCL	3-hydroxymethyl-3-methylglutaryl-CoA lyase
1,06	ZNF395	zinc finger protein 395
1,06	ROBO4	roundabout guidance receptor 4
1,06	ABCB9	ATP binding cassette subfamily B member 9
1,06	C1orf228	chromosome 1 open reading frame 228
1,06	MPDU1	mannose-P-dolichol utilization defect 1
1,06	SPOCK1	sparc/osteonectin, cwcv and kazal-like domains proteoglycan (testican) 1
1,06	FAM217B	family with sequence similarity 217, member B
1,06	UBE2N	ubiquitin conjugating enzyme E2N
1,06	TRIOBP; NOL12	TRIO and F-actin binding protein; nucleolar protein 12
1,06	THOC1	THO complex 1

1,06	ELP2	elongator acetyltransferase complex subunit 2
1,06	NLGN4X	neuroligin 4, X-linked
1,06	LEP	leptin
1,06	LARP6	La ribonucleoprotein domain family, member 6
1,06	TSPY3	testis specific protein, Y-linked 3
1,06	KISS1R	KISS1 receptor
1,06	PRM2	protamine 2
1,06	KIAA0556	KIAA0556
1,06	ZSCAN5A	zinc finger and SCAN domain containing 5A
1,06	GLRA4	glycine receptor alpha 4
1,06	USF2	upstream transcription factor 2, c-fos interacting
1,06	C19orf47	chromosome 19 open reading frame 47
1,06	EMILIN2	elastin microfibril interfacer 2
1,06	LMTK3	lemur tyrosine kinase 3
1,06	RNF121	ring finger protein 121
1,06	C14orf37	chromosome 14 open reading frame 37
1,06	OR13C7	olfactory receptor, family 13, subfamily C, member 7 (gene/pseudogene) [Source:HGNC Symbol;Acc:HGNC:15102]
1,06	ADGRG4	adhesion G protein-coupled receptor G4
1,06	ZNF705CP	zinc finger protein 705C, pseudogene
1,06	ARID3A	AT rich interactive domain 3A (BRIGHT-like)
1,06	OR13C5	olfactory receptor, family 13, subfamily C, member 5
1,06	SGCG	sarcoglycan gamma
1,06	KIAA1683	Memczak2013 ANTISENSE, CDS, coding, INTERNAL best transcript NM_001145304
1,06	SDR42E1	short chain dehydrogenase/reductase family 42E, member 1
1,06	NAA30	N(alpha)-acetyltransferase 30, NatC catalytic subunit
1,06	USP22	ubiquitin specific peptidase 22
1,06	PDE6H	phosphodiesterase 6H, cGMP-specific, cone, gamma
1,06	ZNF416	zinc finger protein 416
1,06	TAS2R42	taste receptor, type 2, member 42
1,06	SKP2	S-phase kinase-associated protein 2, E3 ubiquitin protein ligase
1,06	RPL7A	ribosomal protein L7a
1,06	NEK5	NIMA-related kinase 5
1,06	ASCL3	achaete-scute family bHLH transcription factor 3
1,06	C12orf73	chromosome 12 open reading frame 73
1,06	SUGP1	SURP and G-patch domain containing 1
1,06	LSM3	LSM3 homolog, U6 small nuclear RNA and mRNA degradation associated
1,06	SPINK8	serine peptidase inhibitor, Kazal type 8 (putative)
1,06	SLF2	SMC5-SMC6 complex localization factor 2
1,06	ASTN2	astrotactin 2
1,06	CARD6	caspase recruitment domain family, member 6
1,06	SYVN1	synovial apoptosis inhibitor 1, synoviolin
1,06	PLEKHG1	pleckstrin homology domain containing, family G (with RhoGef domain) member 1

1,06	TSPY3; TSPY4; TSPY8; TSPY1	testis specific protein, Y-linked 3; testis specific protein, Y-linked 4; testis specific protein, Y-linked 8; testis specific protein, Y-linked 1
1,06	GPR25	G protein-coupled receptor 25
1,06	OPCML	opioid binding protein/cell adhesion molecule-like
1,06	KCTD3	potassium channel tetramerization domain containing 3
1,06	RPS6KA1	ribosomal protein S6 kinase, 90kDa, polypeptide 1
1,06	SPDL1	spindle apparatus coiled-coil protein 1
1,06	SNAI3	snail family zinc finger 3
1,06	SLC26A2	solute carrier family 26 (anion exchanger), member 2
1,06	BARHL2	BarH-like homeobox 2
1,06	FCN2	ficolin (collagen/fibrinogen domain containing lectin) 2
1,06	DCAF11	DDB1 and CUL4 associated factor 11
1,06	SEC63	SEC63 homolog, protein translocation regulator
1,06	KCTD18	potassium channel tetramerization domain containing 18
1,06	MEN1	Jeck2013 ANTISENSE, CDS, coding, INTERNAL, OVCODE, OVEXON best transcript NM_130803
1,06	AP3S2; MIR5009	adaptor-related protein complex 3, sigma 2 subunit; microRNA 5009
1,06	SLC16A1	solute carrier family 16 (monocarboxylate transporter), member 1
1,06	DYRK1B	dual specificity tyrosine-(Y)-phosphorylation regulated kinase 1B
1,06	DENND2A	DENN/MADD domain containing 2A
1,06	ELL2	elongation factor, RNA polymerase II, 2
1,06	FAM228A	family with sequence similarity 228, member A
1,06	CCDC6	coiled-coil domain containing 6
1,06	ASB10	ankyrin repeat and SOCS box containing 10
1,06	CLDN2	claudin 2
1,06	ZNF770	zinc finger protein 770
1,06	RTN2	reticulon 2
1,06	GNAQ; skeyplorbu	Memczak2013 ANTISENSE, coding, INTERNAL, intronic best transcript NM_002072; Transcript Identified by AceView
1,06	RS1	retinoschisin 1
1,06	FAM135B	family with sequence similarity 135, member B
1,06	DNAJB1	DnaJ (Hsp40) homolog, subfamily B, member 1
1,06	AGER	advanced glycosylation end product-specific receptor
1,06	RSPH10B2; RSPH10B	radial spoke head 10 homolog B2 (Chlamydomonas); radial spoke head 10 homolog B (Chlamydomonas)
1,06	CBWD2	COBW domain containing 2
1,06	TNFSF15	tumor necrosis factor (ligand) superfamily, member 15
1,06	LIN7C	lin-7 homolog C (C. elegans)
1,06	USP26	ubiquitin specific peptidase 26
1,06	KRBOX4	KRAB box domain containing 4
1,06	ZNF578	zinc finger protein 578
1,06	PTS	6-pyruvoyltetrahydropterin synthase

1,06	CIZ1	CDKN1A interacting zinc finger protein 1
1,06	CXorf40A	chromosome X open reading frame 40A
1,06	SARM1	sterile alpha and TIR motif containing 1
1,06	NDUFA10	NADH dehydrogenase (ubiquinone) 1 alpha subcomplex, 10, 42kDa
1,06	SLC43A1	solute carrier family 43 (amino acid system L transporter), member 1
1,06	ABHD2	abhydrolase domain containing 2
1,06	FAM57A	family with sequence similarity 57, member A
1,06	DIS3L	DIS3 like exosome 3-5 exoribonuclease
1,06	DEXI	Dexi homolog (mouse)
1,06	SCARF1	scavenger receptor class F, member 1
1,06	TMEM167A	transmembrane protein 167A
1,06	ITGB2	Memczak2013 ANTISENSE, CDS, coding, INTERNAL best transcript NM_001127491
1,06	ACSM2A	acyl-CoA synthetase medium-chain family member 2A
1,06	HRH1	histamine receptor H1
1,06	BCO1	beta-carotene oxygenase 1
1,06	OR2AE1	olfactory receptor, family 2, subfamily AE, member 1
1,06	MSR1	macrophage scavenger receptor 1
1,06	MYBL2	v-myb avian myeloblastosis viral oncogene homolog-like 2
1,06	SNRPD1	small nuclear ribonucleoprotein D1 polypeptide
1,06	ODF3B	outer dense fiber of sperm tails 3B
1,06	MINOS1	mitochondrial inner membrane organizing system 1
1,06	ZNF628	zinc finger protein 628
1,06	LTF	lactotransferrin
1,06	RPS6KA2	ribosomal protein S6 kinase, 90kDa, polypeptide 2
1,06	GPR4	G protein-coupled receptor 4
1,06	ST8SIA2	ST8 alpha-N-acetyl-neuraminide alpha-2,8-sialyltransferase 2
1,06	AC009133.17; C16orf54	Memczak2013 ANTISENSE, coding, INTERNAL, intronic best transcript NM_175900; novel transcript, antisense to C16orf54
1,06	HIBCH	Transcript Identified by AceView, Entrez Gene ID(s) 26275
1,06	HIST1H2BM	histone cluster 1, H2bm
1,06	ZNF583	zinc finger protein 583
1,06	PLRG1	pleiotropic regulator 1
1,06	SERTAD2	SERTA domain containing 2
1,06	RHOXF2B; RHOXF2	Rhox homeobox family, member 2B; Rhox homeobox family, member 2
1,06	DNAJB7	DnaJ (Hsp40) homolog, subfamily B, member 7
1,06	GLYR1	glyoxylate reductase 1 homolog (Arabidopsis)
1,06	HGH1	HGH1 homolog
1,06	RIMBP3C	RIMS binding protein 3C
1,06	CTXN1	cortexin 1
1,06	OR2T1	olfactory receptor, family 2, subfamily T, member 1
1,06	LMOD1	leiomodin 1 (smooth muscle)

1,06	INTS4P2	integrator complex subunit 4 pseudogene 2
1,06	EXD1	exonuclease 3-5 domain containing 1
1,06	METTL3	methyltransferase like 3
1,06	SURF1	surfeit 1
1,06	DERL1	derlin 1
1,06	LIG1	ligase I, DNA, ATP-dependent
1,06	DRG1	developmentally regulated GTP binding protein 1
1,06	SLC52A3	solute carrier family 52 (riboflavin transporter), member 3
1,06	KSR1	Zhang2013 ALT_ACCEPTOR, ALT_DONOR, coding, INTERNAL, intronic best transcript NM_014238
1,06	ZNF777	zinc finger protein 777
1,06	SLC18A1	solute carrier family 18 (vesicular monoamine transporter), member 1
1,06	TRMT2A	tRNA methyltransferase 2 homolog A
1,06	ANKRD65	ankyrin repeat domain 65
1,06	COL6A3	collagen, type VI, alpha 3
1,06	NSMF; MIR7114	NMDA receptor synaptonuclear signaling and neuronal migration factor; microRNA 7114
1,06	IFNB1	interferon, beta 1, fibroblast
1,06	IL6R	Memczak2013 ANTISENSE, coding, INTERNAL, UTR3 best transcript NM_000565
1,06	NR2C2	Transcript Identified by AceView, Entrez Gene ID(s) 7182
1,06	WDR89	WD repeat domain 89
1,06	NACC1	nucleus accumbens associated 1, BEN and BTB (POZ) domain containing
1,06	OR1P1	olfactory receptor, family 1, subfamily P, member 1 (gene/pseudogene)
1,06	POLA2	polymerase (DNA directed), alpha 2, accessory subunit
1,06	TGM6	transglutaminase 6
1,06	ANKRD46	ankyrin repeat domain 46
1,06	THEMIS2	thymocyte selection associated family member 2
1,06	FCHSD2	FCH and double SH3 domains 2
1,06	COPS8	COP9 signalosome subunit 8
1,06	LDB1	LIM domain binding 1
1,06	AGK	acylglycerol kinase
1,06	PEX13	peroxisomal biogenesis factor 13
1,06	STXBP3	syntaxin binding protein 3
1,06	RNF216	ring finger protein 216
1,06	NCAN	neurocan
1,06	DDX41	DEAD (Asp-Glu-Ala-Asp) box polypeptide 41
1,06	ANKS4B	ankyrin repeat and sterile alpha motif domain containing 4B
1,06	KCNA1	potassium channel, voltage gated shaker related subfamily A, member 1
1,06	ANGPT4	angiopoietin 4
1,06	MSN	moesin

1,06	ZMIZ1	Zhang2013 ALT_ACCEPTOR, ALT_DONOR, coding, INTERNAL, intronic best transcript NM_020338
1,06	POU2F3	POU class 2 homeobox 3
1,06	OR2T4	olfactory receptor, family 2, subfamily T, member 4
1,06	MORN1	MORN repeat containing 1
1,06	KIF19	kinesin family member 19
1,06	CCDC73	coiled-coil domain containing 73
1,06	KIAA0907; SCARNA4	KIAA0907; small Cajal body-specific RNA 4
1,06	SRSF10	serine/arginine-rich splicing factor 10
1,06	AXL	AXL receptor tyrosine kinase
1,06	PRSS53	protease, serine 53
1,06	ACP6	acid phosphatase 6, lysophosphatidic
1,06	PARD3	par-3 family cell polarity regulator
1,06	MIR4728; ERBB2	microRNA 4728; erb-b2 receptor tyrosine kinase 2
1,06	MAP3K11	mitogen-activated protein kinase kinase kinase 11
1,06	C1orf145	chromosome 1 open reading frame 145
1,06	RAB8A	RAB8A, member RAS oncogene family
1,06	ISOC1	isochorismatase domain containing 1
1,06	CAMK1G	calcium/calmodulin-dependent protein kinase IG
1,06	ZCCHC3	zinc finger, CCHC domain containing 3
1,06	LRRC40	leucine rich repeat containing 40
1,06	GPR3	G protein-coupled receptor 3
1,06	HIST1H3I	histone cluster 1, H3i
1,06	GALNT16	polypeptide N-acetylgalactosaminyltransferase 16
1,06	RPS28	ribosomal protein S28
1,06	SCP2D1	SCP2 sterol-binding domain containing 1
1,06	DNAJA1	DnaJ (Hsp40) homolog, subfamily A, member 1
1,06	FGD2	FYVE, RhoGEF and PH domain containing 2
1,06	SFTPC	surfactant protein C
1,06	SYT3	synaptotagmin III
1,06	IGHMBP2	immunoglobulin mu binding protein 2
1,06	ELFN2	extracellular leucine-rich repeat and fibronectin type III domain containing 2
1,06	S100G	S100 calcium binding protein G
1,06	MLANA	melan-A
1,06	SLC12A8	solute carrier family 12, member 8
1,06	TAMM41	TAM41 mitochondrial translocator assembly and maintenance homolog
1,06	TSPY10	testis specific protein, Y-linked 10
1,06	MICALL2	MICAL-like 2
1,06	OR6C70	olfactory receptor, family 6, subfamily C, member 70
1,06	PPP6R1	Memczak2013 ANTISENSE, CDS, coding, INTERNAL best transcript NM_014931
1,06	CBWD3	COBW domain containing 3
1,06	OAZ3	ornithine decarboxylase antizyme 3
1,06	ZFP69B	ZFP69 zinc finger protein B
1,06	OR52B2	olfactory receptor, family 52, subfamily B, member 2

1,06	B4GALT6	UDP-Gal:betaGlcNAc beta 1,4- galactosyltransferase, polypeptide 6
1,06	CES4A	carboxylesterase 4A
1,06	C11orf68	chromosome 11 open reading frame 68
1,06	MMP21	matrix metallopeptidase 21
1,06	ZNF226	zinc finger protein 226
1,06	SETD1A	SET domain containing 1A
1,06	MEIOB; LINC00254	meiosis specific with OB domains; long intergenic non-protein coding RNA 254
1,06	SCGB1D2	secretoglobin, family 1D, member 2
1,06	PNPLA4	patatin-like phospholipase domain containing 4
1,06	FAM35A	family with sequence similarity 35, member A
1,06	TMEM35	transmembrane protein 35
1,06	ZNF664	zinc finger protein 664
1,06	EIF5AL1	eukaryotic translation initiation factor 5A-like 1
1,06	AIPL1	aryl hydrocarbon receptor interacting protein-like 1
1,06	PRR16	proline rich 16
1,06	SEC11C	SEC11 homolog C, signal peptidase complex subunit
1,06	KLK15	kallikrein related peptidase 15
1,06	EIF5A	eukaryotic translation initiation factor 5A
1,06	WDR97	WD repeat domain 97
1,06	MFSD1	major facilitator superfamily domain containing 1
1,06	WDR3	WD repeat domain 3
1,06	DRG2	developmentally regulated GTP binding protein 2
1,06	C12orf56	chromosome 12 open reading frame 56
1,06	SLCO1B7; SLCO1B1	solute carrier organic anion transporter family, member 1B7 (non-functional); solute carrier organic anion transporter family, member 1B1
1,06	MACROD2; RPS3P1	MACRO domain containing 2; ribosomal protein S3 pseudogene 1
1,06	KLF14	Kruppel-like factor 14
1,06	MYO1F	Memczak2013 ANTISENSE, CDS, coding, INTERNAL best transcript NM_012335
1,06	DUSP14	dual specificity phosphatase 14
1,06	ZNF10	zinc finger protein 10
1,05	GSTM3	glutathione S-transferase mu 3 (brain)
1,05	SSPN	sarcospan
1,05	ZSCAN2	zinc finger and SCAN domain containing 2
1,05	NKX2-6	NK2 homeobox 6
1,05	DMRT2	doublesex and mab-3 related transcription factor 2
1,05	RIF1	replication timing regulatory factor 1
1,05	MAGEA9B; MAGEA9	MAGE family member A9B; MAGE family member A9
1,05	SEC14L2	SEC14-like lipid binding 2
1,05	C5orf47	chromosome 5 open reading frame 47
1,05	TEKT3	tektin 3
1,05	TCN1	transcobalamin I (vitamin B12 binding protein, R binder family)
1,05	TMEM106B	transmembrane protein 106B
1,05	ASB9	ankyrin repeat and SOCS box containing 9

1,05	RBM48	RNA binding motif protein 48
1,05	CELF1	Memczak2013 ALT_ACCEPTOR, ALT_DONOR, coding, INTERNAL, intronic best transcript NM_001172639
1,05	CLEC7A	C-type lectin domain family 7, member A
1,05	ZNF560	zinc finger protein 560
1,05	ZNF431; VN1R82P	zinc finger protein 431; vomeronasal 1 receptor 82 pseudogene
1,05	MSMP	microseminoprotein, prostate associated
1,05	LCTL	lactase-like
1,05	MYEOV	myeloma overexpressed
1,05	COX14	COX14 cytochrome c oxidase assembly factor
1,05	OR2W5	olfactory receptor, family 2, subfamily W, member 5 (gene/pseudogene)
1,05	ZSCAN25	zinc finger and SCAN domain containing 25
1,05	CDSN	corneodesmosin
1,05	MARCH11	membrane associated ring finger 11
1,05	IFNL4	interferon, lambda 4 (gene/pseudogene)
1,05	DPP3	dipeptidyl-peptidase 3
1,05	KIF26B	kinesin family member 26B
1,05	CASC3; MIR6866	cancer susceptibility candidate 3; microRNA 6866
1,05	CPEB3	cytoplasmic polyadenylation element binding protein 3
1,05	SLC25A46	solute carrier family 25, member 46
1,05	ATP8; ATP6; COX3	ATP synthase F0 subunit 8; ATP synthase F0 subunit 6; cytochrome c oxidase III
1,05	TMC4	transmembrane channel like 4
1,05	ZBTB22	zinc finger and BTB domain containing 22
1,05	ISCA1	iron-sulfur cluster assembly 1
1,05	SOX9	SRY box 9
1,05	ZNF639	zinc finger protein 639
1,05	TNFSF13B	tumor necrosis factor (ligand) superfamily, member 13b
1,05	MRPL4	mitochondrial ribosomal protein L4
1,05	PROP1	PROP paired-like homeobox 1
1,05	MLKL	mixed lineage kinase domain-like
1,05	ARHGEF3	Rho guanine nucleotide exchange factor 3
1,05	GCAT	glycine C-acetyltransferase
1,05	SLC7A8	solute carrier family 7 (amino acid transporter light chain, L system), member 8
1,05	POMGNT1	protein O-linked mannose N-acetylglucosaminyltransferase 1 (beta 1,2-)
1,05	SEC24A	SEC24 homolog A, COPII coat complex component
1,05	ACRV1	acrosomal vesicle protein 1
1,05	DCAF4L1	DDB1 and CUL4 associated factor 4-like 1
1,05	CENPQ	centromere protein Q
1,05	GTPBP1	GTP binding protein 1

1,05	DDX11	Zhang2013 ALT_ACCEPTOR, ALT_DONOR, coding, INTERNAL, intronic best transcript NM_030653
1,05	HIF1AN	hypoxia inducible factor 1, alpha subunit inhibitor
1,05	LOC100287225; RP11-267C16.1	uncharacterized LOC100287225; novel transcript
1,05	NOB1	NIN1/RPN12 binding protein 1 homolog
1,05	CDC23	cell division cycle 23
1,05	SLCO2A1	solute carrier organic anion transporter family, member 2A1
1,05	SHH	sonic hedgehog
1,05	VPS11	VPS11, CORVET/HOPS core subunit [Source:HGNC Symbol;Acc:HGNC:14583]
1,05	C11orf84	chromosome 11 open reading frame 84
1,05	GIMAP6	GTPase, IMAP family member 6
1,05	TNNI1	troponin I type 1 (skeletal, slow)
1,05	OR52N2	olfactory receptor, family 52, subfamily N, member 2
1,05	AP1M2	adaptor-related protein complex 1, mu 2 subunit
1,05	NUDT19	nudix hydrolase 19
1,05	C2orf48	chromosome 2 open reading frame 48
1,05	RIMBP2	RIMS binding protein 2
1,05	ZNF550	zinc finger protein 550
1,05	HYOU1	hypoxia up-regulated 1
1,05	DEPDC4	DEP domain containing 4
1,05	IMPA1	inositol(myo)-1(or 4)-monophosphatase 1
1,05	AP5S1	adaptor-related protein complex 5, sigma 1 subunit
1,05	CSTF2	cleavage stimulation factor, 3 pre-RNA, subunit 2
1,05	RP11-10L7.1; HERC6	Transcript Identified by AceView, Entrez Gene ID(s) 55008; novel transcript
1,05	C15orf32	chromosome 15 open reading frame 32
1,05	AKAP8L	A kinase (PRKA) anchor protein 8-like
1,05	CYSRT1	cysteine-rich tail protein 1
1,05	PRDM5	PR domain containing 5
1,05	FZD2	frizzled class receptor 2
1,05	TUBB2A	tubulin, beta 2A class IIa
1,05	OPHN1	oligophrenin 1
1,05	CD276	CD276 molecule
1,05	STXBP4	syntaxin binding protein 4
1,05	PKHD1	polycystic kidney and hepatic disease 1 (autosomal recessive)
1,05	RTF1	RTF1 homolog, Paf1/RNA polymerase II complex component
1,05	C19orf24	chromosome 19 open reading frame 24
1,05	VAMP2	vesicle associated membrane protein 2
1,05	SLC5A3; MRPS6	solute carrier family 5 (sodium/myo-inositol cotransporter), member 3; mitochondrial ribosomal protein S6
1,05	NCK1	NCK adaptor protein 1
1,05	GGA3	golgi-associated, gamma adaptin ear containing, ARF binding protein 3

1,05	DUS3L	Memczak2013 ANTISENSE, CDS, coding, INTERNAL best transcript NM_020175
1,05	OR51A7	olfactory receptor, family 51, subfamily A, member 7
1,05	MAFF	v-maf avian musculoaponeurotic fibrosarcoma oncogene homolog F
1,05	EEFSEC	Memczak2013 ALT_ACCEPTOR, ALT_DONOR, coding, INTERNAL, intronic best transcript NM_021937
1,05	POLR1C	polymerase (RNA) I polypeptide C
1,05	ZNF570	zinc finger protein 570
1,05	C16orf62	chromosome 16 open reading frame 62
1,05	SLC40A1	solute carrier family 40 (iron-regulated transporter), member 1
1,05	UBE2D1	ubiquitin conjugating enzyme E2D 1
1,05	GRM4	glutamate receptor, metabotropic 4
1,05	SYT16	synaptotagmin XVI
1,05	ERCC4	excision repair cross-complementation group 4
1,05	RTP2	receptor (chemosensory) transporter protein 2
1,05	TRAF3IP2	TRAF3 interacting protein 2
1,05	GTF3C5	general transcription factor IIIC subunit 5
1,05	UNC119	unc-119 lipid binding chaperone
1,05	VPS35	VPS35 retromer complex component
1,05	ZNF608	zinc finger protein 608
1,05	LEPROTL1	leptin receptor overlapping transcript-like 1
1,05	CEP162	centrosomal protein 162kDa
1,05	PSMC1	Homo sapiens proteasome (prosome, macropain) 26S subunit, ATPase, 1, mRNA (cDNA clone MGC:8541 IMAGE:2822718), complete cds.; Homo sapiens proteasome (prosome, macropain) 26S subunit, ATPase, 1, mRNA (cDNA clone MGC:24583 IMAGE:4133348), complete cds.; Homo sapiens proteasome (prosome, macropain) 26S subunit, ATPase, 1, mRNA (cDNA clone MGC:86994 IMAGE:5264945), complete cds.; Homo sapiens proteasome (prosome, macropain) 26S subunit, ATPase, 1, mRNA (cDNA clone MGC:88853 IMAGE:5456334), complete cds.
1,05	HYI	hydroxypyruvate isomerase (putative)
1,05	KRTAP6-3	keratin associated protein 6-3
1,05	BEX5	brain expressed X-linked 5
1,05	TMEM255A	transmembrane protein 255A
1,05	YIPF7	Yip1 domain family member 7
1,05	ARL17B; ARL17A	ADP-ribosylation factor like GTPase 17B; ADP-ribosylation factor like GTPase 17A
1,05	MYO5A	myosin VA
1,05	F2RL1	coagulation factor II (thrombin) receptor-like 1
1,05	MYB	v-myb avian myeloblastosis viral oncogene homolog
1,05	TRAPPC4	trafficking protein particle complex 4
1,05	ARHGEF1	Rho guanine nucleotide exchange factor 1
1,05	MAP2K3	mitogen-activated protein kinase kinase 3
1,05	MYO16	myosin XVI

1,05	PBDC1	polysaccharide biosynthesis domain containing 1
1,05	POLR2E	polymerase (RNA) II (DNA directed) polypeptide E, 25kDa
1,05	TPSAB1; TPSB2	tryptase alpha/beta 1; tryptase beta 2 (gene/pseudogene)
1,05	PLCH1	phospholipase C, eta 1
1,05	KAT6B	K(lysine) acetyltransferase 6B
1,05	LRRC41	leucine rich repeat containing 41
1,05	LPIN3	lipin 3
1,05	TMEM41A	transmembrane protein 41A
1,05	TFAP2E	transcription factor AP-2 epsilon (activating enhancer binding protein 2 epsilon)
1,05	COL19A1	collagen, type XIX, alpha 1
1,05	TRIM64B	tripartite motif containing 64B
1,05	CAPN3	calpain 3
1,05	ARIH2	ariadne RBR E3 ubiquitin protein ligase 2
1,05	AQP10	aquaporin 10
1,05	PSKH1	protein serine kinase H1
1,05	GATAD2A	Jeck2013 ALT_ACCEPTOR, ALT_DONOR, coding, INTERNAL, intronic best transcript NM_017660
1,05	MIS18A	MIS18 kinetochore protein A
1,05	ABCB7	ATP binding cassette subfamily B member 7
1,05	IFI27	interferon, alpha-inducible protein 27
1,05	MCCC1	methylcrotonoyl-CoA carboxylase 1
1,05	FARS2	phenylalanyl-tRNA synthetase 2, mitochondrial
1,05	ITGAX	integrin alpha X
1,05	FBXO31	F-box protein 31
1,05	TMEM229B	transmembrane protein 229B
1,05	ATP6V1C1	ATPase, H+ transporting, lysosomal 42kDa, V1 subunit C1
1,05	AMH; MIR4321	anti-Mullerian hormone; microRNA 4321
1,05	FGL1	fibrinogen like 1
1,05	LENG1	leukocyte receptor cluster (LRC) member 1
1,05	C6orf183	chromosome 6 open reading frame 183
1,05	PARP3	poly(ADP-ribose) polymerase family member 3
1,05	ENHO	energy homeostasis associated
1,05	EBLN1	endogenous Bornavirus-like nucleoprotein 1
1,05	DSTN	destrin (actin depolymerizing factor)
1,05	VMA21	VMA21 vacuolar H+-ATPase homolog (S. cerevisiae)
1,05	ZBTB48	zinc finger and BTB domain containing 48
1,05	HTR1B	5-hydroxytryptamine (serotonin) receptor 1B, G protein-coupled
1,05	MLLT1	myeloid/lymphoid or mixed-lineage leukemia; translocated to, 1
1,05	OAS2	2-5-oligoadenylate synthetase 2
1,05	XRCC4	X-ray repair complementing defective repair in Chinese hamster cells 4
1,05	THAP7	THAP domain containing 7

1,05	TRIP6; MIR6875	thyroid hormone receptor interactor 6; microRNA 6875
1,05	YAE1D1	Yae1 domain containing 1
1,05	ARHGAP29	Rho GTPase activating protein 29
1,05	GTSF1L	gametocyte specific factor 1-like
1,05	SPRR1A	small proline-rich protein 1A
1,05	GRM5	glutamate receptor, metabotropic 5
1,05	OR2K2	olfactory receptor, family 2, subfamily K, member 2
1,05	B3GALT5	UDP-Gal:betaGlcNAc beta 1,3-galactosyltransferase 5
1,05	OSTM1	osteopetrosis associated transmembrane protein 1
1,05	TEX12	testis expressed 12
1,05	ZNF35	zinc finger protein 35
1,05	G2E3	G2/M-phase specific E3 ubiquitin protein ligase
1,05	TMEM121	transmembrane protein 121
1,05	RAP1GAP2	RAP1 GTPase activating protein 2
1,05	PCNP	PEST proteolytic signal containing nuclear protein
1,05	EIF5A2	eukaryotic translation initiation factor 5A2
1,05	RAB40B	RAB40B, member RAS oncogene family
1,05	DDX58	DEAD (Asp-Glu-Ala-Asp) box polypeptide 58
1,05	MAP4	microtubule associated protein 4
1,05	KLHL3	kelch-like family member 3
1,05	GSTM2	glutathione S-transferase mu 2 (muscle)
1,05	ALG1L	ALG1, chitobiosyldiphosphodolichol beta-mannosyltransferase-like
1,05	TSHB	thyroid stimulating hormone, beta
1,05	PADI4	peptidyl arginine deiminase, type IV
1,05	FKBPL	FK506 binding protein like
1,05	DEFB105B; DEFB105A	defensin, beta 105B; defensin, beta 105A
1,05	HIST1H3B	histone cluster 1, H3b
1,05	PRDX6	peroxiredoxin 6
1,05	STX8	syntaxin 8
1,05	FAM72A	family with sequence similarity 72, member A
1,05	OR8H2	olfactory receptor, family 8, subfamily H, member 2
1,05	JOSD1	Josephin domain containing 1
1,05	SOCS5	suppressor of cytokine signaling 5
1,05	SCYL1	SCY1-like, kinase-like 1
1,05	CDX4	caudal type homeobox 4
1,05	TMEM192	transmembrane protein 192
1,05	FAM213A	family with sequence similarity 213, member A
1,05	WDSUB1	WD repeat, sterile alpha motif and U-box domain containing 1
1,05	NHSL2	NHS-like 2
1,05	ZNF599	zinc finger protein 599
1,05	RPUSD3	RNA pseudouridylate synthase domain containing 3
1,05	OTOG	otogelin
1,05	GINS3	GINS complex subunit 3 (Psf3 homolog)
1,05	ZNF426	zinc finger protein 426
1,05	CAV3	caveolin 3

1,05	ABHD16B	abhydrolase domain containing 16B
1,05	TRY2P	trypsinogen-like pseudogene
1,05	NKX1-1	NK1 homeobox 1
1,05	UFM1	ubiquitin-fold modifier 1
1,05	TOR1A	torsin family 1, member A (torsin A)
1,05	KIAA2026	KIAA2026
1,05	RASGRF1	Ras protein-specific guanine nucleotide-releasing factor 1
1,05	TRAPPC8	trafficking protein particle complex 8
1,05	ZP2	zona pellucida glycoprotein 2 (sperm receptor)
1,05	TSPY2	testis specific protein, Y-linked 2
1,05	DOC2A	double C2-like domains, alpha
1,05	FAAP20	Fanconi anemia core complex associated protein 20
1,05	LYPLA1	lysophospholipase I
1,05	COASY	CoA synthase
1,05	SLC22A18AS	solute carrier family 22 (organic cation transporter), member 18 antisense
1,05	ZMYM3	zinc finger, MYM-type 3
1,05	CCDC84	coiled-coil domain containing 84
1,05	TMEM258; MIR611	transmembrane protein 258; microRNA 611
1,05	TLE3	Memczak2013 ANTISENSE, coding, INTERNAL, intronic best transcript NM_001105192
1,05	TAF5	TAF5 RNA polymerase II, TATA box binding protein (TBP)-associated factor, 100kDa
1,05	SYK	spleen tyrosine kinase
1,05	ISOC2	isochorismatase domain containing 2
1,05	NR0B1	nuclear receptor subfamily 0, group B, member 1
1,05	ANKRD20A1; ANKRD20A3	ankyrin repeat domain 20 family, member A1; ankyrin repeat domain 20 family, member A3
1,05	NRF1	nuclear respiratory factor 1
1,05	BBS9	Bardet-Biedl syndrome 9
1,05	CNIH1	cornichon family AMPA receptor auxiliary protein 1
1,05	DMRTC2	DMRT-like family C2
1,05	CCNJL	cyclin J-like
1,05	ANKRD36B	ankyrin repeat domain 36B
1,05	CCDC62	coiled-coil domain containing 62
1,05	FKBP15	FK506 binding protein 15
1,05	LINGO1-AS1	LINGO1 antisense RNA 1
1,05	C1QB	complement component 1, q subcomponent, B chain
1,05	FREM3	FRAS1 related extracellular matrix 3
1,05	PYCRL	pyrroline-5-carboxylate reductase-like
1,05	TP53AIP1	tumor protein p53 regulated apoptosis inducing protein 1
1,05	NR5A1	nuclear receptor subfamily 5, group A, member 1
1,05	PSMA1	proteasome subunit alpha 1
1,05	FANCF	Fanconi anemia complementation group F
1,05	RSPO1	R-spondin 1
1,05	GPR75-ASB3	GPR75-ASB3 readthrough

1,05	RBPJL	recombination signal binding protein for immunoglobulin kappa J region-like
1,05	TSNAX	translin-associated factor X
1,05	PRAMEF5	PRAME family member 5
1,05	OR10A4	olfactory receptor, family 10, subfamily A, member 4
1,05	TFAP2A	transcription factor AP-2 alpha (activating enhancer binding protein 2 alpha)
1,05	APOB	apolipoprotein B
1,05	MAT2A	methionine adenosyltransferase II, alpha
1,05	DACT2	dishevelled-binding antagonist of beta-catenin 2
1,05	SMIM14	small integral membrane protein 14
1,05	RNF19B	ring finger protein 19B
1,05	ELMOD2	ELMO/CED-12 domain containing 2
1,05	SEC16B	SEC16 homolog B, endoplasmic reticulum export factor
1,05	CRTC2	CREB regulated transcription coactivator 2
1,05	UBE2A	ubiquitin conjugating enzyme E2A
1,05	NOL10	nucleolar protein 10
1,05	DEFB4B	defensin, beta 4B
1,05	ZBTB14	zinc finger and BTB domain containing 14
1,05	C15orf48; MIR147B	chromosome 15 open reading frame 48; microRNA 147b
1,05	C16orf74	chromosome 16 open reading frame 74
1,05	TIGIT	T-cell immunoreceptor with Ig and ITIM domains
1,05	FBF1	Fas (TNFRSF6) binding factor 1
1,05	TBC1D14	TBC1 domain family, member 14
1,05	C5orf38	chromosome 5 open reading frame 38
1,05	AKIRIN1	akirin 1
1,05	NDUFAF4	NADH dehydrogenase (ubiquinone) complex I, assembly factor 4
1,05	EDC3	enhancer of mRNA decapping 3
1,05	CACNA1S	calcium channel, voltage-dependent, L type, alpha 1S subunit
1,05	CMBL	carboxymethylenebutenolidase homolog (Pseudomonas)
1,05	MFAP3L	microfibrillar associated protein 3 like
1,05	OR10G9	olfactory receptor, family 10, subfamily G, member 9
1,05	ZNF329	zinc finger protein 329
1,05	ANXA8	annexin A8
1,05	SLC2A6	solute carrier family 2 (facilitated glucose transporter), member 6 [Source:HGNC Symbol;Acc:HGNC:11011]
1,05	DDX42	DEAD (Asp-Glu-Ala-Asp) box helicase 42
1,05	SLAIN2	SLAIN motif family member 2
1,05	POM121	POM121 transmembrane nucleoporin
1,05	HOXA7	homeobox A7
1,05	LMBR1L	limb development membrane protein 1-like
1,05	CYP2W1	cytochrome P450, family 2, subfamily W, polypeptide 1
1,05	PDIA3	protein disulfide isomerase family A member 3

1,05	TMEM42	transmembrane protein 42
1,05	ATP6V0C	ATPase, H+ transporting, lysosomal 16kDa, V0 subunit c
1,05	BRICD5	BRICHOS domain containing 5
1,05	FCF1	FCF1 rRNA-processing protein
1,05	DGCR14	Transcript Identified by AceView, Entrez Gene ID(s) 8220
1,05	PDS5A	PDS5 cohesin associated factor A
1,05	DHFRL1	dihydrofolate reductase like 1
1,05	THTPA	thiamine triphosphatase
1,05	CALHM3	calcium homeostasis modulator 3
1,05	SETD7	SET domain containing (lysine methyltransferase) 7
1,05	CPSF4	cleavage and polyadenylation specific factor 4
1,05	TRNAU1AP	tRNA selenocysteine 1 associated protein 1
1,05	TEX40	testis expressed 40
1,05	SLC35E1	solute carrier family 35, member E1
1,05	ZNF267	zinc finger protein 267
1,05	MLF2	myeloid leukemia factor 2
1,05	CHTF8	chromosome transmission fidelity factor 8
1,05	RDH13	retinol dehydrogenase 13 (all-trans/9-cis)
1,05	RFC3	replication factor C subunit 3
1,05	KIF9	kinesin family member 9
1,05	BTC	betacellulin
1,05	ZNF438	zinc finger protein 438
1,05	ANKUB1	ankyrin repeat and ubiquitin domain containing 1
1,05	MTSS1L	metastasis suppressor 1-like
1,05	OR2M5	olfactory receptor, family 2, subfamily M, member 5
1,05	TRAPPC4; MIR3656	trafficking protein particle complex 4; microRNA 3656
1,05	ZNF793	zinc finger protein 793
1,05	TPRG1	tumor protein p63 regulated 1
1,05	KLF6	Kruppel-like factor 6
1,05	HOXD3	homeobox D3
1,05	CLK1	CDC like kinase 1
1,05	DEFB130	defensin, beta 130
1,05	DEFB130	defensin, beta 130
1,05	IDH3G	isocitrate dehydrogenase 3 (NAD+) gamma
1,05	FAM136A	family with sequence similarity 136, member A
1,05	RBM33	RNA binding motif protein 33
1,05	SS18	synovial sarcoma translocation, chromosome 18
1,05	RGS6	regulator of G-protein signaling 6
1,05	FIGN	fidgetin
1,05	SSX7	synovial sarcoma, X breakpoint 7
1,05	OR5M3	olfactory receptor, family 5, subfamily M, member 3
1,05	FSIP2	fibrous sheath interacting protein 2
1,05	C3orf17	chromosome 3 open reading frame 17
1,05	SLC25A21	solute carrier family 25 (mitochondrial oxoadipate carrier), member 21
1,05	DNAJC18	DnaJ (Hsp40) homolog, subfamily C, member 18
1,05	EI24	etoposide induced 2.4

1,05	CDC14A	cell division cycle 14A
1,05	LUZP1	leucine zipper protein 1
1,05	ANKRD54; MIR658	ankyrin repeat domain 54; microRNA 658
1,05	DEK	DEK proto-oncogene
1,05	PIK3CB	phosphatidylinositol-4,5-bisphosphate 3-kinase, catalytic subunit beta
1,05	THADA	thyroid adenoma associated
1,05	OPN1MW2	opsin 1 (cone pigments), medium-wave-sensitive 2
1,05	PCP4	Purkinje cell protein 4
1,05	RGPD3	RANBP2-like and GRIP domain containing 3
1,05	LPGAT1	lysophosphatidylglycerol acyltransferase 1
1,05	SLC25A3; SNORA53	solute carrier family 25 (mitochondrial carrier; phosphate carrier), member 3; small nucleolar RNA, H/ACA box 53
1,05	PGM3	phosphoglucomutase 3
1,05	AC018462.2; COMMD1	Salzman2013 ANTISENSE, coding, INTERNAL, intronic, OVERLAPTX best transcript NM_152516; novel transcript
1,05	ATP6V1A	ATPase, H+ transporting, lysosomal 70kDa, V1 subunit A
1,05	SNAPC2	small nuclear RNA activating complex polypeptide 2
1,05	PRDM1	PR domain containing 1, with ZNF domain
1,05	CDH13	cadherin 13
1,05	DHH	desert hedgehog
1,05	SLC35F5	solute carrier family 35, member F5
1,05	SLC25A17	solute carrier family 25 (mitochondrial carrier; peroxisomal membrane protein, 34kDa), member 17
1,05	OSTF1	osteoclast stimulating factor 1
1,05	LINC01600	long intergenic non-protein coding RNA 1600
1,05	GPR156	G protein-coupled receptor 156
1,05	GGT2	gamma-glutamyltransferase 2
1,05	RIPK1	receptor (TNFRSF)-interacting serine-threonine kinase 1
1,05	MYT1L	myelin transcription factor 1-like
1,05	GLB1; TMPPE	galactosidase beta 1; transmembrane protein with metallophosphoesterase domain
1,05	HNRNPH2	heterogeneous nuclear ribonucleoprotein H2 (H)
1,05	C14orf169	chromosome 14 open reading frame 169
1,05	SMS	spermine synthase
1,05	CREG2	cellular repressor of E1A-stimulated genes 2
1,05	ZBTB45	zinc finger and BTB domain containing 45
1,05	PTPRH	protein tyrosine phosphatase, receptor type, H
1,05	C10orf88	chromosome 10 open reading frame 88
1,05	C10orf142	chromosome 10 open reading frame 142
1,05	B3GAT2	beta-1,3-glucuronyltransferase 2
1,05	AMT; NICN1	aminomethyltransferase; nicolin 1
1,05	PLA2G2C	phospholipase A2, group IIC
1,05	FBXW4	F-box and WD repeat domain containing 4
1,05	C9orf91	chromosome 9 open reading frame 91

1,05	GRB7	growth factor receptor bound protein 7
1,05	UTF1	undifferentiated embryonic cell transcription factor 1
1,05	PROM2	prominin 2
1,05	UBFD1	ubiquitin family domain containing 1
1,05	HHEX	hematopoietically expressed homeobox
1,05	WWP2	WW domain containing E3 ubiquitin protein ligase 2
1,05	USP38	ubiquitin specific peptidase 38
1,05	RAB3GAP1	RAB3 GTPase activating protein subunit 1 (catalytic)
1,05	FAM9A	family with sequence similarity 9, member A
1,05	PRKCDBP	protein kinase C, delta binding protein
1,05	FADD	Fas (TNFRSF6)-associated via death domain
1,05	ZNF839	zinc finger protein 839
1,05	ERN1	endoplasmic reticulum to nucleus signaling 1
1,05	CCL3L3; CCL3L1	chemokine (C-C motif) ligand 3-like 3; chemokine (C-C motif) ligand 3-like 1
1,05	ANO9	anoctamin 9
1,05	SMU1	smu-1 suppressor of mec-8 and unc-52 homolog (C. elegans)
1,05	TULP1	tubby like protein 1
1,05	RANBP3	RAN binding protein 3
1,05	REEP5	receptor accessory protein 5
1,05	PPP5C	protein phosphatase 5, catalytic subunit
1,05	SGEF	Transcript Identified by AceView, Entrez Gene ID(s) 26084
1,05	FAM214B	family with sequence similarity 214, member B
1,05	PROSER3	proline and serine rich 3
1,05	FDXACB1	ferredoxin-fold anticodon binding domain containing 1
1,05	MYPOP	Myb-related transcription factor, partner of profilin
1,05	GGCT	gamma-glutamylcyclotransferase
1,05	TBC1D29	TBC1 domain family, member 29
1,05	ZNF772	zinc finger protein 772
1,05	TIMM17A	translocase of inner mitochondrial membrane 17 homolog A (yeast)
1,05	TXLNG	taxilin gamma
1,05	CD4	CD4 molecule
1,05	PTTG1	pituitary tumor-transforming 1
1,05	TM4SF18	Transcript Identified by AceView, Entrez Gene ID(s) 116441
1,05	SPATA6L	spermatogenesis associated 6-like
1,05	MAK	male germ cell-associated kinase
1,05	MTNR1B	melatonin receptor 1B
1,05	RFXANK	regulatory factor X-associated ankyrin-containing protein
1,05	PUM1	pumilio RNA binding family member 1
1,05	OR5B21	olfactory receptor, family 5, subfamily B, member 21
1,05	VGLL2	vestigial-like family member 2
1,05	YDJC	YdjC homolog (bacterial)
1,05	KCTD8	potassium channel tetramerization domain containing 8
1,05	HIF3A	hypoxia inducible factor 3, alpha subunit

1,05	SAA1	serum amyloid A1
1,05	SYCE1; SPRNP1	synaptonemal complex central element protein 1; shadow of prion protein homolog (zebrafish) pseudogene 1
1,05	WDR78	WD repeat domain 78
1,05	ACAA2	acetyl-CoA acyltransferase 2
1,05	TMEM221	transmembrane protein 221
1,05	PRELID1	PRELI domain containing 1
1,05	HOXB1	homeobox B1
1,05	DND1	DND microRNA-mediated repression inhibitor 1
1,05	TMEM127	transmembrane protein 127
1,05	MORN3	MORN repeat containing 3
1,05	LYSMD3	LysM, putative peptidoglycan-binding, domain containing 3
1,05	AIMP1	aminoacyl tRNA synthetase complex-interacting multifunctional protein 1
1,05	NYAP1	neuronal tyrosine-phosphorylated phosphoinositide-3-kinase adaptor 1
1,05	COPE	coatomer protein complex subunit epsilon
1,05	PARVA	parvin, alpha
1,05	EVC	Ellis van Creveld protein
1,05	SPACA4	sperm acrosome associated 4
1,05	CCDC169-SOHLH2	CCDC169-SOHLH2 readthrough
1,05	WDHD1	WD repeat and HMG-box DNA binding protein 1
1,05	LAMP5	lysosomal-associated membrane protein family, member 5
1,05	PKMYT1	protein kinase, membrane associated tyrosine/threonine 1
1,05	LYPD2	LY6/PLAUR domain containing 2
1,05	PMF1; PMF1-BGLAP; BGLAP	polyamine-modulated factor 1; PMF1-BGLAP readthrough; bone gamma-carboxyglutamate (gla) protein
1,05	TMEM225	transmembrane protein 225
1,05	IRF8	interferon regulatory factor 8
1,05	FBP2	fructose-1,6-bisphosphatase 2
1,05	DDX3X	DEAD (Asp-Glu-Ala-Asp) box helicase 3, X-linked
1,05	ZDHHC13	zinc finger, DHHC-type containing 13
1,05	LINC01549	long intergenic non-protein coding RNA 1549
1,05	KLF17	Kruppel-like factor 17
1,05	PANK2	pantothenate kinase 2
1,05	MMGT1	membrane magnesium transporter 1
1,05	LIX1	limb and CNS expressed 1
1,05	RBMS2	RNA binding motif, single stranded interacting protein 2
1,05	KRT5	keratin 5, type II
1,05	AHSA1	AHA1, activator of heat shock 90kDa protein ATPase homolog 1 (yeast)
1,05	DHX35	DEAH (Asp-Glu-Ala-His) box polypeptide 35
1,05	HOXC8	homeobox C8

1,05	SLC48A1	solute carrier family 48 (heme transporter), member 1
1,05	ARHGAP29	Rho GTPase activating protein 29
1,05	HSFY2; HSFY1	heat shock transcription factor, Y-linked 2; heat shock transcription factor, Y-linked 1
1,05	NAT8B	N-acetyltransferase 8B (GCN5-related, putative, gene/pseudogene)
1,05	ZNF502	zinc finger protein 502
1,05	ACTG1	actin gamma 1
1,05	F8	coagulation factor VIII, procoagulant component
1,05	AMY2B; ACTG1P4	amylase, alpha 2B (pancreatic); actin gamma 1 pseudogene 4
1,05	SMIM8	small integral membrane protein 8
1,05	EPN2; EPN2-IT1	epsin 2; EPN2 intronic transcript 1
1,05	C1QTNF8	C1q and tumor necrosis factor related protein 8
1,05	SLC25A19	solute carrier family 25 (mitochondrial thiamine pyrophosphate carrier), member 19
1,05	ZNF582-AS1	ZNF582 antisense RNA 1 (head to head)
1,05	EXOC7; MIR6868	exocyst complex component 7; microRNA 6868
1,05	GPR18	G protein-coupled receptor 18
1,05	UBQLN3	ubiquilin 3
1,05	AMY1B; AMY1A	amylase, alpha 1B (salivary); amylase, alpha 1A (salivary)
1,05	HOXA11	homeobox A11
1,05	BAG5	BCL2-associated athanogene 5
1,05	DNAJC22	DnaJ (Hsp40) homolog, subfamily C, member 22
1,05	ZNF528	zinc finger protein 528
1,05	MOG	myelin oligodendrocyte glycoprotein
1,05	ARX	aristaless related homeobox
1,05	PSMA8	proteasome subunit alpha 8
1,05	SVIP	small VCP/p97-interacting protein
1,05	CCL8	chemokine (C-C motif) ligand 8
1,05	DEFB126	defensin, beta 126
1,05	CINP	cyclin-dependent kinase 2 interacting protein
1,05	ARRDC2	arrestin domain containing 2
1,05	ITGB1BP1	integrin beta 1 binding protein 1
1,05	KAT2B	Transcript Identified by AceView, Entrez Gene ID(s) 8850
1,05	TXNL4B	thioredoxin-like 4B
1,05	ZNF133	zinc finger protein 133
1,05	TCEA1	transcription elongation factor A (SII), 1
1,05	GTF2H2	general transcription factor IIH subunit 2
1,05	NPHS2	nephrosis 2, idiopathic, steroid-resistant (podocin)
1,05	TECPR2	tectonin beta-propeller repeat containing 2
1,05	C6orf48; SNORD52; SNORD48	chromosome 6 open reading frame 48; small nucleolar RNA, C/D box 52; small nucleolar RNA, C/D box 48
1,05	ELMO2	engulfment and cell motility 2
1,05	GPRC5D	G protein-coupled receptor, class C, group 5, member D

1,05	GSG1L2	GSG1-like 2
1,05	GUCA1B	guanylate cyclase activator 1B (retina)
1,05	PANK3	pantothenate kinase 3
1,05	FGFR3	fibroblast growth factor receptor 3
1,05	MMP27	matrix metallopeptidase 27
1,05	LINC00303	long intergenic non-protein coding RNA 303
1,05	FGFR1OP2	FGFR1 oncogene partner 2
1,05	SLC23A1	solute carrier family 23 (ascorbic acid transporter), member 1
1,05	CTBP2	Transcript Identified by AceView, Entrez Gene ID(s) 1488
1,05	GMPS	guanine monophosphate synthase
1,05	MRVI1-AS1	MRVI1 antisense RNA 1
1,05	BTN2A1	butyrophilin, subfamily 2, member A1
1,05	EDDM3B	epididymal protein 3B
1,05	HDAC8	histone deacetylase 8
1,05	KXD1	KxDL motif containing 1
1,05	CD79B	CD79b molecule, immunoglobulin-associated beta
1,05	PPEF2	protein phosphatase, EF-hand calcium binding domain 2
1,05	MMP20	matrix metallopeptidase 20
1,05	KRT75	keratin 75, type II
1,05	FAF2	Fas associated factor family member 2
1,05	ZNF501	zinc finger protein 501 [Source:HGNC Symbol;Acc:HGNC:23717]
1,05	RECQL	RecQ helicase-like
1,05	ORC3	origin recognition complex subunit 3
1,05	TMEM182	transmembrane protein 182
1,05	LOC400553; RP11-863P13.4	uncharacterized LOC400553; novel transcript
1,05	SLAMF6	SLAM family member 6
1,05	C6orf226	chromosome 6 open reading frame 226
1,05	ACCS	1-aminocyclopropane-1-carboxylate synthase homolog (inactive)
1,05	ZBTB21	zinc finger and BTB domain containing 21
1,05	SERPINA7	serpin peptidase inhibitor, clade A (alpha-1 antiproteinase, antitrypsin), member 7
1,05	UBTD1	ubiquitin domain containing 1
1,05	IFRD1	interferon-related developmental regulator 1
1,05	UBQLN4	ubiquilin 4
1,05	CATSPER3	cation channel, sperm associated 3
1,05	MRFAP1	Morf4 family associated protein 1
1,05	ITGAD	integrin alpha D
1,05	PPP1R7	protein phosphatase 1, regulatory subunit 7
1,05	RGS11	regulator of G-protein signaling 11

1,05	AKAP17A; SFRS17A	Homo sapiens A kinase (PRKA) anchor protein 17A (AKAP17A), transcript variant 1, mRNA.; Homo sapiens A kinase (PRKA) anchor protein 17A (AKAP17A), transcript variant 2, non-coding RNA.; A kinase (PRKA) anchor protein 17A [Source:HGNC Symbol;Acc:HGNC:18783]; Homo sapiens splicing factor, arginine/serine-rich 17A, mRNA (cDNA clone MGC:39904 IMAGE:5217247), complete cds.; Homo sapiens splicing factor, arginine/serine-rich 17A, mRNA (cDNA clone MGC:125365 IMAGE:40007662), complete cds.; Homo sapiens splicing factor, arginine/serine-rich 17A, mRNA (cDNA clone MGC:125366 IMAGE:40007663), complete cds.; Jeck2013 ANNOTATED, INTERNAL, ncRNA, OVCODE, OVEXON best transcript NR_027383
1,05	RAD51B	RAD51 paralog B
1,05	LIMS3L	LIM and senescent cell antigen-like domains 3-like
1,05	C2orf40	chromosome 2 open reading frame 40
1,05	HMBS	hydroxymethylbilane synthase
1,05	SGK3	serum/glucocorticoid regulated kinase family, member 3
1,05	WDR48	WD repeat domain 48
1,05	RNMTL1	RNA methyltransferase like 1
1,05	ARHGAP6	Rho GTPase activating protein 6
1,05	WDR86-AS1	WDR86 antisense RNA 1
1,05	GYLTL1B	glycosyltransferase-like 1B
1,05	KIF2B	kinesin family member 2B
1,05	GK5	glycerol kinase 5 (putative)
1,05	CMSS1	cms1 ribosomal small subunit homolog (yeast)
1,05	RNF113B	ring finger protein 113B
1,05	YES1	YES proto-oncogene 1, Src family tyrosine kinase
1,05	ASIC1	acid sensing ion channel 1
1,05	HTR2A	5-hydroxytryptamine (serotonin) receptor 2A, G protein-coupled
1,05	SYDE2	synapse defective 1, Rho GTPase, homolog 2 (C. elegans)
1,05	ND1	NADH dehydrogenase, subunit 1 (complex I)
1,05	ZNF670; ZNF695; ZNF670-ZNF695	zinc finger protein 670; zinc finger protein 695; ZNF670-ZNF695 readthrough (NMD candidate)
1,05	YWHAQ	tyrosine 3-monooxygenase/tryptophan 5-monooxygenase activation protein, theta
1,05	DOCK2	dedicator of cytokinesis 2
1,05	ANAPC1	anaphase promoting complex subunit 1
1,05	NPDC1	neural proliferation, differentiation and control, 1
1,05	WNK2	WNK lysine deficient protein kinase 2
1,05	CCDC90B	coiled-coil domain containing 90B
1,05	OR5B12	olfactory receptor, family 5, subfamily B, member 12
1,05	TPPP2	tubulin polymerization-promoting protein family member 2

1,05	GUCA2B	guanylate cyclase activator 2B (uroguanylin)
1,05	HNRNPF	Memczak2013 ALT_ACCEPTOR, ALT_DONOR, coding, INTERNAL, intronic best transcript NM_001098207
1,05	DNMT3L	DNA (cytosine-5-)-methyltransferase 3-like
1,05	GABBR2	gamma-aminobutyric acid (GABA) B receptor, 2
1,05	PSMB2	proteasome subunit beta 2
1,05	C8orf31	chromosome 8 open reading frame 31
1,05	CFAP20	cilia and flagella associated protein 20
1,05	KRT86	keratin 86, type II
1,05	CNTLN	Transcript Identified by AceView, Entrez Gene ID(s) 54875
1,05	LPCAT3	lysophosphatidylcholine acyltransferase 3
1,05	C14orf119	chromosome 14 open reading frame 119
1,05	DHRS4L2	dehydrogenase/reductase (SDR family) member 4 like 2
1,05	TAF1A	TATA box binding protein (TBP)-associated factor, RNA polymerase I, A, 48kDa
1,05	UBE2D2	ubiquitin conjugating enzyme E2D 2
1,05	VOPP1	vesicular, overexpressed in cancer, prosurvival protein 1
1,05	NCS1	neuronal calcium sensor 1
1,05	TMC6	transmembrane channel like 6
1,05	CPT2	carnitine palmitoyltransferase 2
1,05	DIMT1	DIM1 dimethyladenosine transferase 1 homolog
1,05	LYPD4	LY6/PLAUR domain containing 4
1,05	GSTK1	glutathione S-transferase kappa 1
1,05	OR8G5	olfactory receptor, family 8, subfamily G, member 5
1,05	CLEC4G	C-type lectin domain family 4, member G
1,05	HIST1H4E	histone cluster 1, H4e
1,05	PRIMPOL	primase and DNA directed polymerase
1,05	OR4N2	olfactory receptor, family 4, subfamily N, member 2
1,05	SPHK2	sphingosine kinase 2
1,05	CEBPG	CCAAT/enhancer binding protein (C/EBP), gamma
1,05	LOC101927844; serfawbu	uncharacterized LOC101927844; Transcript Identified by AceView
1,05	MYO1E	myosin IE
1,05	PSMD5	proteasome 26S subunit, non-ATPase 5
1,05	ABHD13	abhydrolase domain containing 13
1,05	SCN7A	sodium channel, voltage gated, type VII alpha subunit
1,05	UBQLN1	ubiquilin 1
1,05	KRTCAP2	keratinocyte associated protein 2
1,05	COMT; MIR4761	catechol-O-methyltransferase; microRNA 4761
1,05	NDOR1	NADPH dependent diflavin oxidoreductase 1
1,05	GATA1	GATA binding protein 1 (globin transcription factor 1)
1,05	KLHL1	kelch-like family member 1
1,05	BAG4	BCL2-associated athanogene 4
1,05	TUBA3D	tubulin, alpha 3d

1,05	TTC1	Transcript Identified by AceView, Entrez Gene ID(s) 7265
1,05	PTOV1; MIR4749	prostate tumor overexpressed 1; microRNA 4749
1,05	SH3GL1	SH3-domain GRB2-like 1
1,05	PRAMEF25	PRAME family member 25
1,05	GPAA1	glycosylphosphatidylinositol anchor attachment 1
1,05	TAF5L	TAF5-like RNA polymerase II, p300/CBP-associated factor (PCAF)-associated factor, 65kDa
1,05	TCL1A	T-cell leukemia/lymphoma 1A
1,05	EIF5	eukaryotic translation initiation factor 5
1,05	FMNL1	formin like 1
1,05	TMED5	transmembrane p24 trafficking protein 5
1,05	STRADA	STE20-related kinase adaptor alpha
1,05	SLC47A1	solute carrier family 47 (multidrug and toxin extrusion), member 1
1,05	KRT18P55	keratin 18 pseudogene 55
1,05	TCEAL1	transcription elongation factor A (SII)-like 1
1,05	RBM23	RNA binding motif protein 23
1,05	RNFT2	ring finger protein, transmembrane 2
1,05	KLHL17	kelch-like family member 17
1,05	UBE2E1	ubiquitin conjugating enzyme E2E 1
1,05	TCHHL1	trichohyalin like 1
1,05	BNC1	basonuclin 1
1,05	TAS2R38	taste receptor, type 2, member 38
1,05	DYNLL1	dynein, light chain, LC8-type 1
1,05	GLA	galactosidase, alpha
1,05	OR1D2	olfactory receptor, family 1, subfamily D, member 2
1,05	NUFIP2; rerdy	Memczak2013 ALT_ACCEPTOR, ALT_DONOR, coding, INTERNAL, intronic best transcript NM_020772; Transcript Identified by AceView
1,05	PPP2R5C	protein phosphatase 2, regulatory subunit B, gamma
1,05	TMEM54	transmembrane protein 54
1,05	SLC35G1	solute carrier family 35, member G1
1,05	KLF5	Kruppel-like factor 5 (intestinal)
1,05	PECR	peroxisomal trans-2-enoyl-CoA reductase
1,05	AP5B1	adaptor-related protein complex 5, beta 1 subunit
1,05	NIPAL1	NIPA-like domain containing 1
1,05	NF2	neurofibromin 2 (merlin)
1,05	LOC100287036; AC137932.6	uncharacterized LOC100287036; novel transcript, antisense to ANKRD11
1,05	RAX2	retina and anterior neural fold homeobox 2
1,05	ZC3HC1	zinc finger, C3HC-type containing 1
1,05	QTRTD1	queuine tRNA-ribosyltransferase domain containing 1
1,05	ZNF845	zinc finger protein 845
1,05	RPAP3	RNA polymerase II associated protein 3
1,05	FXN	frataxin
1,05	SNX31	sorting nexin 31
1,05	TSPAN14	tetraspanin 14
1,05	PABPC5	poly(A) binding protein, cytoplasmic 5

1,05	SLC17A5	solute carrier family 17 (acidic sugar transporter), member 5
1,05	WBP2NL	WBP2 N-terminal like
1,05	TMED3	transmembrane p24 trafficking protein 3
1,05	STK10	serine/threonine kinase 10
1,05	ALOX5	arachidonate 5-lipoxygenase
1,05	NUDT21	nudix hydrolase 21
1,05	OR5H14	olfactory receptor, family 5, subfamily H, member 14
1,05	HPX	hemopexin
1,05	IGBP1	immunoglobulin (CD79A) binding protein 1
1,05	HSPA1A; HSPA1B	heat shock 70kDa protein 1A; heat shock 70kDa protein 1B
1,05	POGLUT1	protein O-glucosyltransferase 1
1,05	ZMYM5	zinc finger, MYM-type 5
1,05	KRTAP1-3	keratin associated protein 1-3
1,05	ERO1A	endoplasmic reticulum oxidoreductase alpha
1,05	ZNF248	zinc finger protein 248
1,05	EIF2A	eukaryotic translation initiation factor 2A, 65kDa
1,05	RGPD1; RGPD2	RANBP2-like and GRIP domain containing 1; RANBP2-like and GRIP domain containing 2
1,05	TMA16	translation machinery associated 16 homolog
1,05	GFOD2	glucose-fructose oxidoreductase domain containing 2
1,05	DENND2C	DENN/MADD domain containing 2C
1,05	RP1-122P22.2; RIN2	novel transcript; Transcript Identified by AceView, Entrez Gene ID(s) 54453
1,05	ADGRG6	adhesion G protein-coupled receptor G6
1,05	CCNB1	cyclin B1
1,05	SLC26A11	solute carrier family 26 (anion exchanger), member 11
1,05	IL18BP	interleukin 18 binding protein
1,05	ZNF571	zinc finger protein 571
1,05	C20orf96	chromosome 20 open reading frame 96
1,05	SLC6A15	solute carrier family 6 (neutral amino acid transporter), member 15
1,05	AP1S3	adaptor-related protein complex 1 sigma 3 subunit
1,05	KDELR1	KDEL (Lys-Asp-Glu-Leu) endoplasmic reticulum protein retention receptor 1
1,05	DMPK	dystrophia myotonica-protein kinase
1,05	ARL6IP5	ADP-ribosylation factor like GTPase 6 interacting protein 5
1,05	NSUN3	Transcript Identified by AceView, Entrez Gene ID(s) 63899
1,05	RASGEF1A	RasGEF domain family member 1A
1,05	CENPL	centromere protein L
1,05	SUPT16H	SPT16 homolog, facilitates chromatin remodeling subunit
1,05	ACACB	acetyl-CoA carboxylase beta
1,05	SLC13A1	solute carrier family 13 (sodium/sulfate symporter), member 1
1,05	TNFAIP8	tumor necrosis factor, alpha-induced protein 8

1,05	FABP5	fatty acid binding protein 5 (psoriasis-associated)
1,05	PADI4	peptidyl arginine deiminase, type IV
1,05	DUSP11	dual specificity phosphatase 11
1,05	TUBA1B	tubulin, alpha 1b
1,05	RPL5; SNORD21; SNORA66	ribosomal protein L5; small nucleolar RNA, C/D box 21; small nucleolar RNA, H/ACA box 66
1,05	MICAL2	microtubule associated monooxygenase, calponin and LIM domain containing 2
1,05	ULK2	unc-51 like autophagy activating kinase 2
1,05	XRCC5	X-ray repair complementing defective repair in Chinese hamster cells 5 (double-strand-break rejoining)
1,05	JUN	jun proto-oncogene
1,05	PLP1	proteolipid protein 1
1,05	RAB2B	RAB2B, member RAS oncogene family
1,05	SYT9	synaptotagmin IX
1,05	PLEKHJ1; MIR6789	pleckstrin homology domain containing, family J member 1; microRNA 6789
1,05	APH1B	APH1B gamma secretase subunit
1,05	SNAI2	snail family zinc finger 2
1,05	TK2	thymidine kinase 2, mitochondrial
1,05	AMIGO3	adhesion molecule with Ig-like domain 3
1,05	CACFD1	calcium channel flower domain containing 1
1,05	OMG	oligodendrocyte myelin glycoprotein
1,05	ULK4	unc-51 like kinase 4
1,05	PTBP1; MIR4745	polypyrimidine tract binding protein 1; microRNA 4745
1,05	HSPA9	heat shock 70kDa protein 9 (mortalin)
1,05	BEST4	bestrophin 4
1,05	CCT2	chaperonin containing TCP1, subunit 2 (beta)
1,05	BPHL	biphenyl hydrolase-like (serine hydrolase)
1,05	CCDC57	coiled-coil domain containing 57
1,05	CLEC18C	C-type lectin domain family 18, member C
1,05	BLACE	B-cell acute lymphoblastic leukemia expressed
1,05	GPX2	glutathione peroxidase 2
1,05	TMTC3	transmembrane and tetratricopeptide repeat containing 3
1,05	ACAD11	acyl-CoA dehydrogenase family, member 11
1,05	TNXB	tenascin XB
1,05	GABRB3	gamma-aminobutyric acid (GABA) A receptor, beta 3
1,05	PIGL	phosphatidylinositol glycan anchor biosynthesis class L
1,05	GDI2	GDP dissociation inhibitor 2
1,05	IL1B	interleukin 1 beta
1,05	APLNR	apelin receptor
1,05	ARHGAP15	Memczak2013 ALT_ACCEPTOR, ALT_DONOR, coding, INTERNAL, intronic best transcript NM_018460
1,05	AVPI1	arginine vasopressin-induced 1

1,05	CPLX3	complexin 3
1,05	NECAP1	NECAP endocytosis associated 1
1,05	VWA7	von Willebrand factor A domain containing 7
1,05	TMED7-TICAM2; TICAM2; TMED7	TMED7-TICAM2 readthrough; toll-like receptor adaptor molecule 2; transmembrane p24 trafficking protein 7
1,05	BRD3	bromodomain containing 3
1,05	RNF165	ring finger protein 165
1,05	MYADM	Memczak2013 ANTISENSE, CDS, coding, INTERNAL best transcript NM_001020819
1,05	CCDC84	coiled-coil domain containing 84
1,05	BAX	BCL2-associated X protein
1,05	SIMC1	SUMO-interacting motifs containing 1
1,05	UBE2Z	ubiquitin-conjugating enzyme E2Z
1,05	IQCF1	IQ motif containing F1
1,05	TSPO2	translocator protein 2
1,05	BRK1	BRICK1, SCAR/WAVE actin-nucleating complex subunit
1,05	NPTX2	neuronal pentraxin II
1,05	OR52H1	olfactory receptor, family 52, subfamily H, member 1
1,05	VPS54	vacuolar protein sorting 54 homolog (S. cerevisiae)
1,05	RAB7B	RAB7B, member RAS oncogene family
1,05	ABCD4	ATP binding cassette subfamily D member 4
1,05	SH2B3	Transcript Identified by AceView, Entrez Gene ID(s) 10019
1,05	CLEC2B	C-type lectin domain family 2, member B
1,05	METTL10	methyltransferase like 10
1,05	UBL7	ubiquitin-like 7
1,05	RMI1	RecQ mediated genome instability 1
1,05	PPA1	pyrophosphatase (inorganic) 1
1,05	P2RX4	purinergic receptor P2X, ligand gated ion channel, 4
1,05	TMEM151B	transmembrane protein 151B
1,05	MARCH10	membrane associated ring finger 10
1,05	HSPE1	heat shock 10kDa protein 1
1,05	EOGT	EGF domain-specific O-linked N-acetylglucosamine (GlcNAc) transferase
1,05	ADAMTS5	ADAM metallopeptidase with thrombospondin type 1 motif 5
1,05	DIABLO	diablo, IAP-binding mitochondrial protein
1,04	FAM8A1	family with sequence similarity 8, member A1
1,04	STYXL1	serine/threonine/tyrosine interacting-like 1
1,04	SLC5A5	solute carrier family 5 (sodium/iodide cotransporter), member 5
1,04	CYP2F1	cytochrome P450, family 2, subfamily F, polypeptide 1
1,04	SPIN1	spindlin 1
1,04	YIF1B	Yip1 interacting factor homolog B (S. cerevisiae)
1,04	SGMS2	sphingomyelin synthase 2

1,04	KDELR2	KDEL (Lys-Asp-Glu-Leu) endoplasmic reticulum protein retention receptor 2
1,04	ENPP2	ectonucleotide pyrophosphatase/phosphodiesterase 2
1,04	JPH2	junctophilin 2
1,04	E2F1	Memczak2013 ANTISENSE, coding, INTERNAL, intronic best transcript NM_005225
1,04	UBE2G2	ubiquitin conjugating enzyme E2G 2
1,04	THUMPD2	THUMP domain containing 2
1,04	OR2L13	olfactory receptor, family 2, subfamily L, member 13
1,04	PSAPL1	prosaposin-like 1 (gene/pseudogene)
1,04	HRSP12	heat-responsive protein 12
1,04	EXOSC8	exosome component 8
1,04	PRKCI	protein kinase C, iota
1,04	WFDC10A	WAP four-disulfide core domain 10A
1,04	SUPT5H	SPT5 homolog, DSIF elongation factor subunit
1,04	CD53	CD53 molecule
1,04	ATG16L1	autophagy related 16-like 1
1,04	MAP3K6	mitogen-activated protein kinase kinase kinase 6
1,04	PPCDC	phosphopantothenoylcysteine decarboxylase
1,04	CCDC188	coiled-coil domain containing 188
1,04	BAK1	BCL2-antagonist/killer 1
1,04	NLRP10	NLR family, pyrin domain containing 10
1,04	PCSK1N	proprotein convertase subtilisin/kexin type 1 inhibitor
1,04	DGCR5; DGCR9	DiGeorge syndrome critical region gene 5 (non-protein coding); DiGeorge syndrome critical region gene 9 (non-protein coding)
1,04	ZNF584	zinc finger protein 584
1,04	TOMM5	translocase of outer mitochondrial membrane 5 homolog (yeast)
1,04	CCNK	cyclin K
1,04	CACNG3	calcium channel, voltage-dependent, gamma subunit 3
1,04	CCNG1	cyclin G1
1,04	HERC6	HECT and RLD domain containing E3 ubiquitin protein ligase family member 6
1,04	ZBTB47	zinc finger and BTB domain containing 47
1,04	AHR	aryl hydrocarbon receptor
1,04	RAD51D	RAD51 paralog D
1,04	MAFG	v-maf avian musculoaponeurotic fibrosarcoma oncogene homolog G
1,04	PCP2	Purkinje cell protein 2
1,04	FNDC8	fibronectin type III domain containing 8
1,04	ABCG4	ATP binding cassette subfamily G member 4
1,04	IRGC	immunity-related GTPase family, cinema
1,04	SLC22A12	solute carrier family 22 (organic anion/urate transporter), member 12
1,04	CLEC3A	C-type lectin domain family 3, member A
1,04	ANAPC13	anaphase promoting complex subunit 13
1,04	RSC1A1; DDI2	regulatory solute carrier protein, family 1, member 1; DNA-damage inducible 1 homolog 2

1,04	RPL27A; SNORA3A; SNORA3B	ribosomal protein L27a; small nucleolar RNA, H/ACA box 3A; small nucleolar RNA, H/ACA box 3B
1,04	MYH6	myosin, heavy chain 6, cardiac muscle, alpha
1,04	EFCAB8	EF-hand calcium binding domain 8
1,04	RDH13	retinol dehydrogenase 13 (all-trans/9-cis)
1,04	CSK	c-src tyrosine kinase
1,04	SLC25A23	solute carrier family 25 (mitochondrial carrier; phosphate carrier), member 23
1,04	RLN3	relaxin 3
1,04	CPNE3	copine III
1,04	TEX261	testis expressed 261
1,04	APOA2	apolipoprotein A-II
1,04	NCLN	nicalin
1,04	ZNF213	zinc finger protein 213
1,04	EEFSEC	eukaryotic elongation factor, selenocysteine-tRNA-specific
1,04	PSENEN	presenilin enhancer gamma secretase subunit
1,04	STEAP1B	STEAP family member 1B
1,04	ABHD12	abhydrolase domain containing 12
1,04	EFNB1	ephrin-B1
1,04	DRP2	dystrophin related protein 2
1,04	COL5A2	collagen, type V, alpha 2
1,04	HPCAL4	hippocalcin like 4
1,04	E2F8	E2F transcription factor 8
1,04	C6orf62	chromosome 6 open reading frame 62
1,04	ZNF274	Memczak2013 ANTISENSE, CDS, coding, INTERNAL best transcript NM_133502
1,04	BVES-AS1	BVES antisense RNA 1
1,04	USP12	Transcript Identified by AceView, Entrez Gene ID(s) 219333
1,04	PPID	peptidylprolyl isomerase D
1,04	C10orf128	chromosome 10 open reading frame 128
1,04	LRRC58	leucine rich repeat containing 58
1,04	MSR1	macrophage scavenger receptor 1
1,04	ASIC5	acid sensing ion channel family member 5
1,04	HNRNPH3	heterogeneous nuclear ribonucleoprotein H3 (2H9)
1,04	TTC39B	Transcript Identified by AceView, Entrez Gene ID(s) 158219
1,04	ANKRD23	ankyrin repeat domain 23
1,04	SLC34A3	solute carrier family 34 (type II sodium/phosphate cotransporter), member 3
1,04	CAMK2B	calcium/calmodulin-dependent protein kinase II beta
1,04	KCNA6; GALNT8	potassium channel, voltage gated shaker related subfamily A, member 6; polypeptide N-acetylgalactosaminyltransferase 8
1,04	DAZ2; DAZ4	deleted in azoospermia 2; deleted in azoospermia 4
1,04	NAT16	N-acetyltransferase 16 (GCN5-related, putative)
1,04	SPEF2	sperm flagellar 2

1,04	TFAP4	transcription factor AP-4 (activating enhancer binding protein 4)
1,04	DEFB115	defensin, beta 115
1,04	CCDC144NL-AS1	CCDC144NL antisense RNA 1
1,04	OR2G2	olfactory receptor, family 2, subfamily G, member 2
1,04	BPIFC	BPI fold containing family C
1,04	HOXD9	homeobox D9
1,04	KIAA0513	KIAA0513
1,04	CCKAR	cholecystokinin A receptor
1,04	DNAAF3	dynein, axonemal, assembly factor 3
1,04	NCKAP1	NCK-associated protein 1
1,04	LRRC74B	leucine rich repeat containing 74B
1,04	SLC9A3	solute carrier family 9, subfamily A (NHE3, cation proton antiporter 3), member 3
1,04	CCL23	chemokine (C-C motif) ligand 23
1,04	HIST1H3D; HIST1H2AD	histone cluster 1, H3d; histone cluster 1, H2ad
1,04	DTNBP1	dystrobrevin binding protein 1
1,04	ZNF837	zinc finger protein 837
1,04	HHAT	hedgehog acyltransferase
1,04	KRT85	keratin 85, type II
1,04	UNC5D	unc-5 netrin receptor D
1,04	BBX	bobby sox homolog (Drosophila)
1,04	AGBL4	ATP/GTP binding protein-like 4
1,04	CRLF3	Memczak2013 ALT_ACCEPTOR, ALT_DONOR, coding, INTERNAL, intronic best transcript NM_015986
1,04	RAD23A	RAD23 homolog A, nucleotide excision repair protein
1,04	ACOT8	acyl-CoA thioesterase 8
1,04	HIVEP2	Jeck2013 ALT_ACCEPTOR, ALT_DONOR, coding, INTERNAL, intronic best transcript NM_006734
1,04	MED23	mediator complex subunit 23
1,04	C6orf191andARHGAP18; RP1-69D17.4; RP1-69D17.3; TCONS_l2_00025470; TCONS_l2_00024911; LAMA2	Transcript Identified by AceView, Entrez Gene ID(s) 93663; 253582; Jeck2013 ALT_ACCEPTOR, ALT_DONOR, downstream_end, ncRNA, OVEXON, upstream_start best transcript TCONS_l2_00025470; Jeck2013 ALT_ACCEPTOR, ncRNA, OVEXON, upstream_start best transcript TCONS_l2_00024911; Salzman2013 ANTISENSE, CDS, coding, downstream_end, intronic, OVCODE, OVEXON, UTR3 best transcript NM_000426; Salzman2013 ANNOTATED, INTERNAL, ncRNA, OVEXON best transcript TCONS_l2_00025470; putative novel transcript; novel transcript
1,04	LINC00266-1	long intergenic non-protein coding RNA 266-1
1,04	NKX6-1	NK6 homeobox 1
1,04	NBPF4	neuroblastoma breakpoint family, member 4
1,04	TRANK1	tetratricopeptide repeat and ankyrin repeat containing 1

1,04	PPP1R16B	protein phosphatase 1, regulatory subunit 16B
1,04	ASAP1	Memczak2013 ALT_ACCEPTOR, ALT_DONOR, coding, INTERNAL, intronic best transcript NM_018482
1,04	APIP	Transcript Identified by AceView, Entrez Gene ID(s) 51074
1,04	DNAJB3	DnaJ (Hsp40) homolog, subfamily B, member 3
1,04	AKAP8	A kinase (PRKA) anchor protein 8
1,04	NASP	Zhang2013 ALT_DONOR, coding, INTERNAL, intronic best transcript NM_002482
1,04	CHST2	carbohydrate (N-acetylglucosamine-6-O) sulfotransferase 2
1,04	ZNF347	zinc finger protein 347
1,04	PPAT	phosphoribosyl pyrophosphate amidotransferase
1,04	MMP2	matrix metallopeptidase 2
1,04	SNORA17A; SNORA17B; SNHG7	small nucleolar RNA, H/ACA box 17A; small nucleolar RNA, H/ACA box 17B; small nucleolar RNA host gene 7
1,04	PGD	phosphogluconate dehydrogenase
1,04	SPANXA2	SPANX family, member A2
1,04	LGMN	legumain
1,04	SPDYE5	speedy/RINGO cell cycle regulator family member E5
1,04	LIX1L	limb and CNS expressed 1 like
1,04	MMP7	matrix metallopeptidase 7
1,04	TUBB4B	tubulin, beta 4B class IVb
1,04	FAM71E1	family with sequence similarity 71, member E1
1,04	BCKDHA	branched chain keto acid dehydrogenase E1, alpha polypeptide
1,04	N4BP2L1	NEDD4 binding protein 2-like 1
1,04	LINC00243	long intergenic non-protein coding RNA 243
1,04	SPATA31C1	SPATA31 subfamily C, member 1
1,04	CCDC84	coiled-coil domain containing 84
1,04	MCM3AP-AS1	MCM3AP antisense RNA 1
1,04	ZNF780A	zinc finger protein 780A
1,04	ANK2	ankyrin 2, neuronal
1,04	ARHGAP12	Rho GTPase activating protein 12
1,04	KPNA5	karyopherin alpha 5 (importin alpha 6)
1,04	BAAT	bile acid-CoA:amino acid N-acyltransferase
1,04	FAM177B	family with sequence similarity 177, member B
1,04	ZNF775	zinc finger protein 775
1,04	APOBEC2	apolipoprotein B mRNA editing enzyme, catalytic polypeptide-like 2
1,04	TMEM86A	transmembrane protein 86A
1,04	TMEM161B	transmembrane protein 161B
1,04	CPNE9	copine family member IX
1,04	SETD9	SET domain containing 9
1,04	MED25; MIR6800	mediator complex subunit 25; microRNA 6800
1,04	CLCA2	chloride channel accessory 2

1,04	ALX4	ALX homeobox 4
1,04	SENP6	SUMO1/sentrin specific peptidase 6
1,04	HSD3B1	hydroxy-delta-5-steroid dehydrogenase, 3 beta- and steroid delta-isomerase 1
1,04	OPRM1	opioid receptor, mu 1
1,04	PPARA	peroxisome proliferator-activated receptor alpha
1,04	DUX4L7	double homeobox 4 like 7
1,04	MEPE	matrix extracellular phosphoglycoprotein
1,04	SHPK; TRPV1	sedoheptulokinase; transient receptor potential cation channel, subfamily V, member 1
1,04	ZFP36L2	ZFP36 ring finger protein-like 2
1,04	FILIP1	filamin A interacting protein 1
1,04	APC2	adenomatosis polyposis coli 2
1,04	AGR3	anterior gradient 3, protein disulphide isomerase family member
1,04	BCAS1	breast carcinoma amplified sequence 1
1,04	REEP4	receptor accessory protein 4
1,04	CTPS1	CTP synthase 1
1,04	AK3	adenylate kinase 3
1,04	LCT	lactase
1,04	DTD1	D-tyrosyl-tRNA deacylase 1
1,04	F11R	F11 receptor
1,04	SRPX2	sushi-repeat containing protein, X-linked 2
1,04	GPATCH4	G-patch domain containing 4
1,04	ACBD3	acyl-CoA binding domain containing 3
1,04	CSF3	colony stimulating factor 3
1,04	STAC3	SH3 and cysteine rich domain 3
1,04	MAPRE2	microtubule-associated protein, RP/EB family, member 2
1,04	MRPL22	mitochondrial ribosomal protein L22
1,04	PROZ	protein Z, vitamin K-dependent plasma glycoprotein
1,04	ASB15	ankyrin repeat and SOCS box containing 15
1,04	CCDC124	coiled-coil domain containing 124
1,04	FPGT-TNNI3K; FPGT	FPGT-TNNI3K readthrough; fucose-1-phosphate guanylyltransferase
1,04	RBM5	RNA binding motif protein 5
1,04	SPPL2C	signal peptide peptidase like 2C
1,04	CLSTN3	calsyntenin 3
1,04	ARSF	arylsulfatase F
1,04	C16orf92	chromosome 16 open reading frame 92
1,04	KIAA1024L	KIAA1024-like
1,04	LINC00471	long intergenic non-protein coding RNA 471
1,04	ATOH7	atonal bHLH transcription factor 7
1,04	DENND1C	DENN/MADD domain containing 1C
1,04	PCDH18	protocadherin 18
1,04	LINC01296; DUXAP10	long intergenic non-protein coding RNA 1296; double homeobox A pseudogene 10
1,04	MRPS27	mitochondrial ribosomal protein S27
1,04	SLC18A3	solute carrier family 18 (vesicular acetylcholine transporter), member 3

1,04	POGZ	Transcript Identified by AceView, Entrez Gene ID(s) 23126
1,04	TMEM117	transmembrane protein 117
1,04	PCGF5	polycomb group ring finger 5
1,04	EVL	Enah/Vasp-like
1,04	CYB5R1	cytochrome b5 reductase 1
1,04	PHAX	phosphorylated adaptor for RNA export
1,04	NPFFR2	neuropeptide FF receptor 2
1,04	RARB	retinoic acid receptor, beta
1,04	FGA	fibrinogen alpha chain
1,04	HPDL	4-hydroxyphenylpyruvate dioxygenase-like
1,04	UMOD	uromodulin
1,04	OR52W1	olfactory receptor, family 52, subfamily W, member 1
1,04	FAM9C	family with sequence similarity 9, member C
1,04	FOSL2	FOS-like antigen 2
1,04	POLRMT	polymerase (RNA) mitochondrial (DNA directed)
1,04	ITIH1	inter-alpha-trypsin inhibitor heavy chain 1
1,04	PRAMEF10	PRAME family member 10
1,04	RPS16	ribosomal protein S16
1,04	ZNF513	zinc finger protein 513
1,04	CGNL1	cingulin-like 1
1,04	EMP3	epithelial membrane protein 3
1,04	DFFA	DNA fragmentation factor, 45kDa, alpha polypeptide
1,04	C1orf50	chromosome 1 open reading frame 50
1,04	RPS4X	ribosomal protein S4, X-linked
1,04	RPE	ribulose-5-phosphate-3-epimerase
1,04	ZDHHC24	zinc finger, DHHC-type containing 24
1,04	PIK3AP1	phosphoinositide-3-kinase adaptor protein 1
1,04	ZAP70	zeta chain of T cell receptor associated protein kinase 70kDa
1,04	PRCC	papillary renal cell carcinoma (translocation-associated)
1,04	ZNF806	zinc finger protein 806
1,04	CCIN	calicin
1,04	ZAR1L	zygote arrest 1-like
1,04	ZFAND5	zinc finger, AN1-type domain 5
1,04	ESPNL	espin-like
1,04	UBAP2L	ubiquitin associated protein 2 like
1,04	PSPN	persephin
1,04	PCSK7	proprotein convertase subtilisin/kexin type 7
1,04	ACVR2A	activin A receptor type IIA
1,04	IQSEC2	IQ motif and Sec7 domain 2
1,04	PIWIL1	piwi-like RNA-mediated gene silencing 1
1,04	AASDH	aminoadipate-semialdehyde dehydrogenase
1,04	CPN1	carboxypeptidase N, polypeptide 1
1,04	SDHAF1	succinate dehydrogenase complex assembly factor 1
1,04	NDNL2	necdin-like 2, SMC5-SMC6 complex component
1,04	VPS37A	vacuolar protein sorting 37 homolog A (S. cerevisiae)
1,04	DRC1	dynein regulatory complex subunit 1
1,04	PFN1	profilin 1

1,04	C21orf140	chromosome 21 open reading frame 140
1,04	C21orf140	chromosome 21 open reading frame 140
1,04	RPL36A-HNRNPH2	RPL36A-HNRNPH2 readthrough
1,04	ABCB8	ATP binding cassette subfamily B member 8
1,04	SYT4	synaptotagmin IV
1,04	ENPP3	ectonucleotide pyrophosphatase/phosphodiesterase 3
1,04	SPANXN1	SPANX family, member N1
1,04	MVP; PAGR1	major vault protein; PAXIP1 associated glutamate-rich protein 1
1,04	GJA1	gap junction protein alpha 1
1,04	HIP1R	huntingtin interacting protein 1 related
1,04	HORMAD1	HORMA domain containing 1
1,04	GEMIN5	gem nuclear organelle associated protein 5
1,04	CCDC115	coiled-coil domain containing 115
1,04	ATRIP; TREX1	ATR interacting protein; three prime repair exonuclease 1
1,04	AP2A2	adaptor-related protein complex 2, alpha 2 subunit
1,04	TRAPPC4	trafficking protein particle complex 4
1,04	CATSPER1	cation channel, sperm associated 1
1,04	ECI1	enoyl-CoA delta isomerase 1
1,04	FAM182B	family with sequence similarity 182, member B
1,04	RRP36	ribosomal RNA processing 36
1,04	FAM157B	family with sequence similarity 157, member B
1,04	SIRT4	sirtuin 4
1,04	KIAA0319L	KIAA0319-like
1,04	RAET1L	retinoic acid early transcript 1L
1,04	M1AP	meiosis 1 associated protein
1,04	ZFPM2	zinc finger protein, FOG family member 2
1,04	SLC38A1	solute carrier family 38, member 1
1,04	BCAR3	breast cancer anti-estrogen resistance 3
1,04	RAB21	RAB21, member RAS oncogene family
1,04	DNAAF2	dynein, axonemal, assembly factor 2
1,04	CR1L	complement component (3b/4b) receptor 1-like
1,04	STX11	syntaxin 11
1,04	SCP2	sterol carrier protein 2
1,04	SUPT4H1	SPT4 homolog, DSIF elongation factor subunit
1,04	DCLRE1A	DNA cross-link repair 1A
1,04	RPL18	ribosomal protein L18
1,04	MTAP	methylthioadenosine phosphorylase
1,04	PPP2CA	protein phosphatase 2, catalytic subunit, alpha isozyme
1,04	EME1	essential meiotic structure-specific endonuclease 1
1,04	FAAH	fatty acid amide hydrolase
1,04	ARFGAP1; MIR4326	ADP-ribosylation factor GTPase activating protein 1; microRNA 4326
1,04	ARPP21	cAMP-regulated phosphoprotein 21kDa
1,04	SCLY	selenocysteine lyase
1,04	CEP83	centrosomal protein 83kDa
1,04	RPL4	ribosomal protein L4

1,04	KAT2A	K(lysine) acetyltransferase 2A
1,04	LRRC34	leucine rich repeat containing 34
1,04	PTGR1	prostaglandin reductase 1
1,04	PNCK	pregnancy up-regulated nonubiquitous CaM kinase
1,04	MAP1LC3B2	microtubule-associated protein 1 light chain 3 beta 2
1,04	PAEP	progestagen-associated endometrial protein
1,04	PEAR1	platelet endothelial aggregation receptor 1
1,04	MYO18B	myosin XVIIIB
1,04	FGF17	fibroblast growth factor 17
1,04	MAGEA5	MAGE family member A5
1,04	POLDIP3	polymerase (DNA-directed), delta interacting protein 3
1,04	NDFIP1	Nedd4 family interacting protein 1
1,04	PTPRK	protein tyrosine phosphatase, receptor type, K
1,04	PROSER2	proline and serine rich 2
1,04	TSPY8	testis specific protein, Y-linked 8
1,04	CDC42SE1	CDC42 small effector 1
1,04	LOC399886; RP11-430H10.2; FLJ41423	uncharacterized LOC399886; Transcript Identified by AceView, Entrez Gene ID(s) 399886; novel transcript
1,04	NPY2R	neuropeptide Y receptor Y2
1,04	MGAT3	mannosyl (beta-1,4-)-glycoprotein beta-1,4-N-acetylglucosaminyltransferase
1,04	NUDT4	nudix hydrolase 4
1,04	PVRL4	poliovirus receptor-related 4
1,04	BIRC6	Transcript Identified by AceView, Entrez Gene ID(s) 57448
1,04	ALDH6A1	aldehyde dehydrogenase 6 family, member A1
1,04	EIF2S2	Zhang2013 ALT_ACCEPTOR, ALT_DONOR, coding, INTERNAL, intronic best transcript NM_003908
1,04	TMEM55B	transmembrane protein 55B
1,04	RPL12	Zhang2013 ALT_ACCEPTOR, ALT_DONOR, coding, INTERNAL, intronic best transcript NM_000976
1,04	PSMD4	proteasome 26S subunit, non-ATPase 4
1,04	PTRH1	peptidyl-tRNA hydrolase 1 homolog
1,04	HECTD3	HECT domain containing E3 ubiquitin protein ligase 3
1,04	MAP7D1	MAP7 domain containing 1
1,04	LINC01343	long intergenic non-protein coding RNA 1343
1,04	PIGH	phosphatidylinositol glycan anchor biosynthesis class H
1,04	LRG1	leucine-rich alpha-2-glycoprotein 1
1,04	DDHD2	DDHD domain containing 2
1,04	COMMD2	COMM domain containing 2
1,04	RAB27B	RAB27B, member RAS oncogene family
1,04	CHCHD4	coiled-coil-helix-coiled-coil-helix domain containing 4
1,04	PIAS4	protein inhibitor of activated STAT 4

1,04	STX4	syntaxin 4
1,04	RILPL1	Rab interacting lysosomal protein-like 1
1,04	DCTN5	dynactin 5 (p25)
1,04	RAB40AL	RAB40A, member RAS oncogene family-like
1,04	TXNRD2	thioredoxin reductase 2
1,04	DPP10	dipeptidyl-peptidase 10 (inactive)
1,04	ZKSCAN2	zinc finger with KRAB and SCAN domains 2
1,04	IBTK	inhibitor of Bruton agammaglobulinemia tyrosine kinase
1,04	PSMG4	Transcript Identified by AceView, Entrez Gene ID(s) 389362
1,04	RGPD6; RGPD5	RANBP2-like and GRIP domain containing 6; RANBP2-like and GRIP domain containing 5
1,04	CIC	capicua transcriptional repressor
1,04	LRRC75B	leucine rich repeat containing 75B
1,04	ZNF197	zinc finger protein 197
1,04	CCNT1	cyclin T1
1,04	R3HDML	R3H domain containing-like
1,04	PRAMEF8	PRAME family member 8
1,04	GNL1	guanine nucleotide binding protein-like 1
1,04	HBA2	hemoglobin, alpha 2
1,04	CCDC47	coiled-coil domain containing 47
1,04	NDUFB7	NADH dehydrogenase (ubiquinone) 1 beta subcomplex, 7, 18kDa
1,04	KCNS1	potassium voltage-gated channel, modifier subfamily S, member 1
1,04	ZNF429	zinc finger protein 429
1,04	ECT2L	epithelial cell transforming 2 like
1,04	CTSA	cathepsin A
1,04	OR8D4	olfactory receptor, family 8, subfamily D, member 4
1,04	BTD	biotinidase
1,04	AUH	AU RNA binding protein/enoyl-CoA hydratase
1,04	CLCNKB	chloride channel, voltage-sensitive Kb
1,04	DAZAP2	DAZ associated protein 2
1,04	ABCC4	ATP binding cassette subfamily C member 4
1,04	WNK3	WNK lysine deficient protein kinase 3
1,04	CRYM	crystallin mu
1,04	FAM120C	family with sequence similarity 120C
1,04	MRPL11	mitochondrial ribosomal protein L11
1,04	DISC1	disrupted in schizophrenia 1
1,04	ZNF559-ZNF177; ZNF177; ZNF559	ZNF559-ZNF177 readthrough; zinc finger protein 177; zinc finger protein 559
1,04	ADAMTSL4	ADAMTS like 4
1,04	CCDC163P	coiled-coil domain containing 163, pseudogene
1,04	RAB39B	RAB39B, member RAS oncogene family
1,04	CBWD5	COBW domain containing 5
1,04	ARL2-SNX15	ARL2-SNX15 readthrough (NMD candidate)
1,04	BTN3A1	butyrophilin, subfamily 3, member A1
1,04	MCCC2	methylcrotonoyl-CoA carboxylase 2
1,04	FOCAD	focadhesin

1,04	OR8H1	olfactory receptor, family 8, subfamily H, member 1
1,04	NEURL3	neuralized E3 ubiquitin protein ligase 3
1,04	UNCX	UNC homeobox
1,04	TMEM233	transmembrane protein 233
1,04	DDTL; DDT	D-dopachrome tautomerase-like; D-dopachrome tautomerase
1,04	PRR23A	proline rich 23A
1,04	HDGF	hepatoma-derived growth factor
1,04	TEX43	testis expressed 43
1,04	RNF219	ring finger protein 219
1,04	PLA2G10	phospholipase A2, group X
1,04	YAF2	YY1 associated factor 2
1,04	MED16	mediator complex subunit 16
1,04	CLDN3	claudin 3
1,04	HOMER1	homer scaffolding protein 1
1,04	GNB3	guanine nucleotide binding protein (G protein), beta polypeptide 3
1,04	TMEM100	transmembrane protein 100
1,04	ALG1L9P	asparagine-linked glycosylation 1-like 9, pseudogene
1,04	MARCH5	membrane associated ring finger 5
1,04	CYP4F22	cytochrome P450, family 4, subfamily F, polypeptide 22
1,04	ANKRD49	ankyrin repeat domain 49
1,04	URGCP; URGCP-MRPS24	upregulator of cell proliferation; URGCP-MRPS24 readthrough
1,04	NABP1	nucleic acid binding protein 1
1,04	AMY1C; AMY1A	amylase, alpha 1C (salivary); amylase, alpha 1A (salivary)
1,04	TNNT2	troponin T type 2 (cardiac)
1,04	ETV6	ets variant 6
1,04	CORO1C	coronin, actin binding protein, 1C
1,04	TRABD	TraB domain containing
1,04	NUP43	nucleoporin 43kDa
1,04	RBMY1F; RBMY2FP; RBMY1J	RNA binding motif protein, Y-linked, family 1, member F; RNA binding motif protein, Y-linked, family 2, member F pseudogene; RNA binding motif protein, Y-linked, family 1, member J
1,04	SAP130	Sin3A associated protein 130kDa
1,04	HCFC1R1	host cell factor C1 regulator 1 (XPO1 dependent)
1,04	PKNOX2	PBX/knotted 1 homeobox 2
1,04	GNAI3	guanine nucleotide binding protein (G protein), alpha inhibiting activity polypeptide 3
1,04	LEMD1	LEM domain containing 1
1,04	LOC100129697; RP11-830F9.6	uncharacterized LOC100129697; novel transcript, antisense to CBFA2T3
1,04	OR10AD1	olfactory receptor, family 10, subfamily AD, member 1
1,04	PROB1	proline-rich basic protein 1
1,04	TDRP	testis development related protein
1,04	ZNF326	zinc finger protein 326

1,04	SFXN1	sideroflexin 1
1,04	AVPR1B	arginine vasopressin receptor 1B
1,04	ZMAT5	zinc finger, matrin-type 5
1,04	STX2	syntaxin 2
1,04	GINS3	GINS complex subunit 3 (Psf3 homolog)
1,04	CYP26A1	cytochrome P450, family 26, subfamily A, polypeptide 1
1,04	TMEM248	transmembrane protein 248
1,04	IFNLR1	interferon, lambda receptor 1
1,04	NEFH	neurofilament, heavy polypeptide
1,04	SPG21	spastic paraplegia 21 (autosomal recessive, Mast syndrome)
1,04	ADAM18	ADAM metallopeptidase domain 18
1,04	GOT2	glutamic-oxaloacetic transaminase 2, mitochondrial
1,04	ZNF788	zinc finger family member 788
1,04	ASPRV1; PCBP1-AS1	aspartic peptidase, retroviral-like 1; PCBP1 antisense RNA 1
1,04	DAG1	dystroglycan 1 (dystrophin-associated glycoprotein 1)
1,04	BFAR	bifunctional apoptosis regulator
1,04	DUSP16	dual specificity phosphatase 16
1,04	FOLH1	folate hydrolase (prostate-specific membrane antigen) 1
1,04	ST20	suppressor of tumorigenicity 20
1,04	INTS6	integrator complex subunit 6
1,04	CLPP	caseinolytic mitochondrial matrix peptidase proteolytic subunit
1,04	GNGT2	guanine nucleotide binding protein (G protein), gamma transducing activity polypeptide 2
1,04	FBXL3	F-box and leucine-rich repeat protein 3
1,04	BTNL2	butyrophilin-like 2
1,04	BCAT2	branched chain amino-acid transaminase 2, mitochondrial
1,04	RASD1	RAS, dexamethasone-induced 1
1,04	SCAPER	Transcript Identified by AceView, Entrez Gene ID(s) 49855
1,04	LRSAM1	leucine rich repeat and sterile alpha motif containing 1
1,04	DYNC2LI1	dynein, cytoplasmic 2, light intermediate chain 1
1,04	HHIP	hedgehog interacting protein
1,04	TAF6	TAF6 RNA polymerase II, TATA box binding protein (TBP)-associated factor, 80kDa
1,04	MICU1	mitochondrial calcium uptake 1
1,04	STKLD1	serine/threonine kinase-like domain containing 1
1,04	RAD54B	Transcript Identified by AceView, Entrez Gene ID(s) 25788
1,04	WDR12	WD repeat domain 12
1,04	CWC27	CWC27 spliceosome-associated protein homolog
1,04	NKX2-3	NK2 homeobox 3
1,04	COX8C	cytochrome c oxidase subunit VIIIC
1,04	SCN4B	sodium channel, voltage gated, type IV beta subunit

1,04	THG1L	tRNA-histidine guanylyltransferase 1-like
1,04	MTDH	metadherin
1,04	FOXK1	Transcript Identified by AceView, Entrez Gene ID(s) 221937
1,04	MAFA	v-maf avian musculoaponeurotic fibrosarcoma oncogene homolog A
1,04	CMTM1	CKLF-like MARVEL transmembrane domain containing 1
1,04	INPP5D	inositol polyphosphate-5-phosphatase D
1,04	CYHR1	cysteine/histidine-rich 1
1,04	sep-06	septin 6
1,04	PPIF	peptidylprolyl isomerase F
1,04	TMEM78	transmembrane protein 78
1,04	HN1L	hematological and neurological expressed 1-like
1,04	FOXJ3	forkhead box J3
1,04	MCM9	minichromosome maintenance 9 homologous recombination repair factor
1,04	ABHD8	abhydrolase domain containing 8
1,04	MSH6	mutS homolog 6
1,04	HNRNPH1	heterogeneous nuclear ribonucleoprotein H1 (H)
1,04	PSG5	pregnancy specific beta-1-glycoprotein 5
1,04	ABCA13	ATP binding cassette subfamily A member 13
1,04	VNN3	vanin 3
1,04	VPS50	VPS50 EARP/GARPII complex subunit
1,04	RPS15	ribosomal protein S15
1,04	C16orf71	chromosome 16 open reading frame 71
1,04	SLC31A1	solute carrier family 31 (copper transporter), member 1
1,04	ILKAP	ILK associated serine/threonine phosphatase
1,04	CCDC84	coiled-coil domain containing 84
1,04	OXNAD1	oxidoreductase NAD-binding domain containing 1
1,04	SOCS4	suppressor of cytokine signaling 4
1,04	ZNF781	zinc finger protein 781
1,04	GPC3	glypican 3
1,04	SART1	squamous cell carcinoma antigen recognized by T-cells 1
1,04	PLD2	phospholipase D2
1,04	GDAP2	ganglioside induced differentiation associated protein 2
1,04	ACRC	acidic repeat containing
1,04	CAB39	calcium binding protein 39
1,04	ZNF430	zinc finger protein 430
1,04	STAU2	staufen double-stranded RNA binding protein 2
1,04	MAT2B	methionine adenosyltransferase II, beta
1,04	SERPINA12	serpin peptidase inhibitor, clade A (alpha-1 antiproteinase, antitrypsin), member 12
1,04	ZNF536	zinc finger protein 536
1,04	RASSF4	Ras association (RalGDS/AF-6) domain family member 4

1,04	GABRA6	gamma-aminobutyric acid (GABA) A receptor, alpha 6
1,04	ACTG2	actin, gamma 2, smooth muscle, enteric
1,04	LNPEP	leucyl/cystinyl aminopeptidase
1,04	SURF1	surfeit 1
1,04	CST7	cystatin F (leukocystatin)
1,04	NBPF12	neuroblastoma breakpoint family, member 12
1,04	IRGQ	immunity-related GTPase family, Q
1,04	RANBP17	RAN binding protein 17
1,04	KRTAP22-1	keratin associated protein 22-1
1,04	MXD1	Memczak2013 ANTISENSE, coding, INTERNAL, intronic best transcript NM_001202514
1,04	C2orf83	chromosome 2 open reading frame 83
1,04	UQCC3	ubiquinol-cytochrome c reductase complex assembly factor 3
1,04	HEATR4	HEAT repeat containing 4
1,04	FRMD5	FERM domain containing 5
1,04	PEX19	peroxisomal biogenesis factor 19
1,04	TNK1	tyrosine kinase, non-receptor, 1
1,04	CYP7A1	cytochrome P450, family 7, subfamily A, polypeptide 1
1,04	KRTAP1-1	keratin associated protein 1-1
1,04	TRIL	TLR4 interactor with leucine-rich repeats
1,04	CSNK1A1L	casein kinase 1, alpha 1-like
1,04	SLC25A13	solute carrier family 25 (aspartate/glutamate carrier), member 13
1,04	DMRTA2	DMRT-like family A2
1,04	POU4F3	POU class 4 homeobox 3
1,04	ATXN7L3B	ataxin 7-like 3B
1,04	TCF7L2	transcription factor 7-like 2 (T-cell specific, HMG-box)
1,04	KAT2A	Memczak2013 ALT_DONOR, coding, INTERNAL, intronic best transcript NM_021078
1,04	TXNIP	thioredoxin interacting protein
1,04	LYZ	lysozyme
1,04	RBM15B	RNA binding motif protein 15B
1,04	TRPV3	transient receptor potential cation channel, subfamily V, member 3
1,04	YPEL5	yippee like 5
1,04	WDR17	WD repeat domain 17
1,04	SPON2	spondin 2, extracellular matrix protein
1,04	CLNS1A	chloride channel, nucleotide-sensitive, 1A
1,04	CLEC2L	C-type lectin domain family 2, member L
1,04	PLAC9	placenta specific 9
1,04	PLPP6	phospholipid phosphatase 6
1,04	VPREB1	pre-B lymphocyte 1
1,04	USP39	ubiquitin specific peptidase 39
1,04	CAND1	cullin-associated and neddylation-dissociated 1
1,04	ZNF761; TPM3P9	zinc finger protein 761; tropomyosin 3 pseudogene 9

1,04	CCDC18	coiled-coil domain containing 18
1,04	CCR5	chemokine (C-C motif) receptor 5 (gene/pseudogene)
1,04	DPH2	DPH2 homolog
1,04	STRIP2	striatin interacting protein 2
1,04	GAGE12H; GAGE12B; GAGE12C	G antigen 12H; G antigen 12B; G antigen 12C
1,04	GAGE12D	G antigen 12D
1,04	GAGE12G; GAGE12E	G antigen 12G; G antigen 12E
1,04	SLC25A44	solute carrier family 25, member 44
1,04	KCNN2	potassium channel, calcium activated intermediate/small conductance subfamily N alpha, member 2
1,04	PTMA	prothymosin, alpha
1,04	NUP58	nucleoporin 58kDa
1,04	ADAT2	adenosine deaminase, tRNA-specific 2
1,04	DPF2	D4, zinc and double PHD fingers family 2
1,04	ERICH4	glutamate rich 4
1,04	KCNK13	potassium channel, two pore domain subfamily K, member 13
1,04	PEG10	paternally expressed 10
1,04	SPATS1	spermatogenesis associated, serine-rich 1
1,04	CCL1	chemokine (C-C motif) ligand 1
1,04	CA2	carbonic anhydrase II
1,04	SEC22C	SEC22 homolog C, vesicle trafficking protein
1,04	DCAF13	DDB1 and CUL4 associated factor 13
1,04	OR51F1	olfactory receptor, family 51, subfamily F, member 1 (gene/pseudogene)
1,04	PDZRN4	PDZ domain containing ring finger 4
1,04	WBP5	WW domain binding protein 5
1,04	ARRDC1	arrestin domain containing 1
1,04	RNF112	ring finger protein 112
1,04	CARM1	coactivator-associated arginine methyltransferase 1
1,04	ARMCX1	armadillo repeat containing, X-linked 1
1,04	ANAPC2	anaphase promoting complex subunit 2
1,04	KCNF1	potassium channel, voltage gated modifier subfamily F, member 1
1,04	ALG1; NAGPA-AS1	ALG1, chitobiosyldiphosphodolichol beta-mannosyltransferase; NAGPA antisense RNA 1
1,04	TTR	transthyretin
1,04	ELK1	ELK1, member of ETS oncogene family
1,04	NAALADL2	N-acetylated alpha-linked acidic dipeptidase-like 2
1,04	FAF1	Fas (TNFRSF6) associated factor 1
1,04	NAE1	NEDD8 activating enzyme E1 subunit 1
1,04	RGS22	regulator of G-protein signaling 22
1,04	UHRF1BP1L	UHRF1 binding protein 1-like
1,04	NHLH2	nescient helix-loop-helix 2
1,04	NT5C3A	5-nucleotidase, cytosolic IIIA
1,04	MOB1A	MOB kinase activator 1A

1,04	ZDHHC15	zinc finger, DHHC-type containing 15
1,04	KIF25	kinesin family member 25
1,04	MEIS2	Meis homeobox 2
1,04	CYBB	cytochrome b-245, beta polypeptide
1,04	TH	tyrosine hydroxylase
1,04	POLR2L	polymerase (RNA) II (DNA directed) polypeptide L, 7.6kDa
1,04	PVRL3	poliovirus receptor-related 3
1,04	RBM25	RNA binding motif protein 25
1,04	NSA2	NSA2 ribosome biogenesis homolog
1,04	KALRN	kalirin, RhoGEF kinase
1,04	FASTKD1	FAST kinase domains 1
1,04	GPT2	glutamic pyruvate transaminase (alanine aminotransferase) 2
1,04	FAM91A1	family with sequence similarity 91, member A1
1,04	ZNF263	zinc finger protein 263
1,04	ATP13A3	Jeck2013 ALT_ACCEPTOR, ALT_DONOR, coding, INTERNAL, intronic best transcript NM_024524
1,04	HAGHL	hydroxyacylglutathione hydrolase-like
1,04	PSMG3-AS1	PSMG3 antisense RNA 1 (head to head)
1,04	SLC25A26	solute carrier family 25 (S-adenosylmethionine carrier), member 26
1,04	CPNE8	copine VIII
1,04	RPS6KB1	ribosomal protein S6 kinase, 70kDa, polypeptide 1
1,04	EOMES	eomesodermin
1,04	FER1L6	fer-1-like family member 6
1,04	SH2D4A	Transcript Identified by AceView, Entrez Gene ID(s) 63898
1,04	TBX1	T-box 1
1,04	PRPF40B	PRP40 homolog, pre-mRNA processing factor B
1,04	ARL10	ADP-ribosylation factor like GTPase 10
1,04	ENGASE	endo-beta-N-acetylglucosaminidase
1,04	RFT1	RFT1 homolog
1,04	AKT1S1	Memczak2013 ANTISENSE, coding, INTERNAL, intronic best transcript NM_001098633
1,04	SYPL1	synaptophysin-like 1
1,04	CLP1	cleavage and polyadenylation factor I subunit 1
1,04	ZNF566	zinc finger protein 566
1,04	SGK1	serum/glucocorticoid regulated kinase 1
1,04	OR2J3	olfactory receptor, family 2, subfamily J, member 3
1,04	TREH	trehalase
1,04	UNC80	unc-80 homolog (C. elegans)
1,04	SNX29	sorting nexin 29
1,04	STAB2	stabilin 2
1,04	RPS6KA1	Zhang2013 ALT_ACCEPTOR, ALT_DONOR, coding, INTERNAL, intronic best transcript NM_002953
1,04	GLDC	glycine dehydrogenase (decarboxylating)
1,04	AOX1	aldehyde oxidase 1
1,04	FAM222B	family with sequence similarity 222, member B

1,04	KRTAP20-2	keratin associated protein 20-2
1,04	C7orf43; MIR4658	chromosome 7 open reading frame 43; microRNA 4658
1,04	STIP1	stress-induced phosphoprotein 1
1,04	MRPS21	mitochondrial ribosomal protein S21
1,04	SPCS1	signal peptidase complex subunit 1
1,04	FAM83C	family with sequence similarity 83, member C
1,04	C3orf14	chromosome 3 open reading frame 14
1,04	ZNF546	zinc finger protein 546
1,04	LOC155060; ZNF783	AI894139 pseudogene; Transcript Identified by AceView, Entrez Gene ID(s) 155060
1,04	IL9R	interleukin 9 receptor
1,04	IL9R	Homo sapiens interleukin 9 receptor (IL9R), transcript variant 1, mRNA.; Homo sapiens interleukin 9 receptor (IL9R), transcript variant 2, mRNA.; interleukin 9 receptor [Source:HGNC Symbol;Acc:HGNC:6030]; Homo sapiens interleukin 9 receptor, mRNA (cDNA clone MGC:59852 IMAGE:6304829), complete cds.; Transcript Identified by AceView, Entrez Gene ID(s) 3581, RefSeq ID(s) NM_002186; Transcript Identified by AceView, Entrez Gene ID(s) 3581
1,04	WDR34	WD repeat domain 34
1,04	SPOCK2	sparc/osteonectin, cwcv and kazal-like domains proteoglycan (testican) 2
1,04	WNT5A	wingless-type MMTV integration site family, member 5A
1,04	CHAT	choline O-acetyltransferase
1,04	SLC25A45	solute carrier family 25, member 45
1,04	TSC1	tuberous sclerosis 1
1,04	OSBPL6	oxysterol binding protein-like 6
1,04	RPL37	ribosomal protein L37
1,04	TBX3	T-box 3
1,04	TPCN1	two pore segment channel 1
1,04	FNBP1L	Transcript Identified by AceView, Entrez Gene ID(s) 54874
1,04	GP6	glycoprotein VI (platelet)
1,04	DUSP8	dual specificity phosphatase 8
1,04	GRM6	glutamate receptor, metabotropic 6
1,04	PAICS	phosphoribosylaminoimidazole carboxylase, phosphoribosylaminoimidazole succinocarboxamide synthetase
1,04	SUPT7L	SPT7-like STAGA complex gamma subunit
1,04	ZFAND4	zinc finger, AN1-type domain 4
1,04	IDI1	isopentenyl-diphosphate delta isomerase 1
1,04	S100A14	S100 calcium binding protein A14
1,04	LONRF3	LON peptidase N-terminal domain and ring finger 3
1,04	ST7L	suppression of tumorigenicity 7 like
1,04	SLC5A10	solute carrier family 5 (sodium/sugar cotransporter), member 10
1,04	FAM185A	family with sequence similarity 185, member A

1,04	ZNF343	zinc finger protein 343
1,04	TM9SF4	transmembrane 9 superfamily protein member 4
1,04	HDLBP	high density lipoprotein binding protein
1,04	RLN1	relaxin 1
1,04	MCAT	malonyl-CoA-acyl carrier protein transacylase
1,04	KIFAP3	kinesin-associated protein 3
1,04	RAB43	RAB43, member RAS oncogene family
1,04	BHLHB9	basic helix-loop-helix domain containing, class B, 9
1,04	PIK3IP1	phosphoinositide-3-kinase interacting protein 1
1,04	CACFD1	calcium channel flower domain containing 1
1,04	CHI3L2	chitinase 3-like 2
1,04	TNPO1	transportin 1
1,04	CCDC134	coiled-coil domain containing 134
1,04	CRHBP	corticotropin releasing hormone binding protein
1,04	RPP14	ribonuclease P/MRP 14kDa subunit
1,04	TEKT4P2	tektin 4 pseudogene 2
1,04	LINC01559	long intergenic non-protein coding RNA 1559
1,04	FLG	filaggrin
1,04	ZNF641	zinc finger protein 641
1,04	MKNK1	MAP kinase interacting serine/threonine kinase 1
1,04	TCEAL8	transcription elongation factor A (SII)-like 8
1,04	TRMT10C	tRNA methyltransferase 10C, mitochondrial RNase P subunit
1,04	STARD7	StAR-related lipid transfer domain containing 7
1,04	ULK4P1; ULK4P2	ULK4 pseudogene 1; ULK4 pseudogene 2
1,04	WTH3DI; LOC150786	RAB6C-like; Synthetic construct Homo sapiens clone IMAGE:100016523, MGC:184337 RAB6C-like (LOC150786) mRNA, encodes complete protein.
1,04	FANCA	Fanconi anemia complementation group A
1,04	TGFBR3L	transforming growth factor beta receptor III like
1,04	SPP2	secreted phosphoprotein 2
1,04	EIF4G1	eukaryotic translation initiation factor 4 gamma, 1
1,04	APOC1	apolipoprotein C-I
1,04	POMGNT2	protein O-linked mannose N-acetylglucosaminyltransferase 2 (beta 1,4-)
1,04	DCUN1D2	DCN1, defective in cullin neddylation 1, domain containing 2
1,04	OR10A2	olfactory receptor, family 10, subfamily A, member 2
1,04	THAP10	THAP domain containing 10
1,04	ACOXL	acyl-CoA oxidase-like
1,04	DNAJC2	DnaJ (Hsp40) homolog, subfamily C, member 2
1,04	CD2	CD2 molecule
1,04	SPO11	SPO11 meiotic protein covalently bound to DSB
1,04	TARS	threonyl-tRNA synthetase
1,04	TMEM189	transmembrane protein 189
1,04	ACOT7	acyl-CoA thioesterase 7
1,04	MFSD3	major facilitator superfamily domain containing 3

1,04	XKRY2; XKRY	Homo sapiens XK, Kell blood group complex subunit-related, Y-linked 2 (XKRY2), mRNA.; Homo sapiens XK, Kell blood group complex subunit-related, Y-linked (XKRY), mRNA.; Homo sapiens XK, Kell blood group complex subunit-related, Y-linked, mRNA (cDNA clone MGC:164514 IMAGE:40146905), complete cds.; Homo sapiens XK, Kell blood group complex subunit-related, Y-linked, mRNA (cDNA clone MGC:164516 IMAGE:40146907), complete cds.; Homo sapiens XK, Kell blood group complex subunit-related, Y-linked 2, mRNA (cDNA clone MGC:164532 IMAGE:40146923), complete cds.; Homo sapiens XK, Kell blood group complex subunit-related, Y-linked 2, mRNA (cDNA clone MGC:164534 IMAGE:40146925), complete cds.; Synthetic construct Homo sapiens clone IMAGE:100015836, MGC:183189 XK, Kell blood group complex subunit-related, Y-linked 2 (XKRY2) mRNA, encodes complete protein.; Synthetic construct Homo sapiens clone IMAGE:100016528, MGC:184271 XK, Kell blood group complex subunit-related, Y-linked (XKRY) mRNA, encodes complete protein.; Transcript Identified by AceView, Entrez Gene ID(s) 353515, RefSeq ID(s) NM_004677, NM_001002906
1,04	POGZ	Transcript Identified by AceView, Entrez Gene ID(s) 23126
1,04	CCDC28A	coiled-coil domain containing 28A
1,04	HIST1H1C	Memczak2013 ANTISENSE, CDS, coding, INTERNAL best transcript NM_005319
1,04	FBXL22	F-box and leucine-rich repeat protein 22
1,04	APOLD1; DDX47	apolipoprotein L domain containing 1; DEAD (Asp-Glu-Ala-Asp) box polypeptide 47
1,04	OR6F1; OR14A2	olfactory receptor, family 6, subfamily F, member 1; olfactory receptor, family 14, subfamily A, member 2
1,04	APLP1	amyloid beta (A4) precursor-like protein 1
1,04	WFDC5	WAP four-disulfide core domain 5
1,04	CCR1	chemokine (C-C motif) receptor 1
1,04	NPY5R	neuropeptide Y receptor Y5
1,04	EPC1	enhancer of polycomb homolog 1 (Drosophila)
1,04	GRIP1	glutamate receptor interacting protein 1
1,04	KALRN	Transcript Identified by AceView, Entrez Gene ID(s) 8997
1,04	GDPD2	glycerophosphodiester phosphodiesterase domain containing 2
1,04	TRUB2	TruB pseudouridine (psi) synthase family member 2
1,04	TMEM184B	transmembrane protein 184B
1,04	CDPF1	cysteine rich, DPF motif domain containing 1
1,04	ABHD14B	abhydrolase domain containing 14B
1,04	ZBTB5	zinc finger and BTB domain containing 5
1,04	NAP1L1	nucleosome assembly protein 1-like 1

1,04	FN3K	fructosamine 3 kinase
1,04	FEZF2	FEZ family zinc finger 2
1,04	MAPK9	mitogen-activated protein kinase 9
1,04	TFCP2L1	transcription factor CP2-like 1
1,04	ARHGAP9	Rho GTPase activating protein 9
1,04	FITM2	fat storage-inducing transmembrane protein 2
1,04	LYPD5	LY6/PLAUR domain containing 5
1,04	DRC7	dynein regulatory complex subunit 7
1,04	ASNSD1	asparagine synthetase domain containing 1
1,04	ICT1	immature colon carcinoma transcript 1
1,04	CUL4B	cullin 4B
1,04	IDE	insulin-degrading enzyme
1,04	CACNA1G	calcium channel, voltage-dependent, T type, alpha 1G subunit
1,04	CAPN10	calpain 10
1,04	HN1	hematological and neurological expressed 1
1,04	NUDT2	nudix hydrolase 2
1,04	NIPA2	non imprinted in Prader-Willi/Angelman syndrome 2
1,04	TMEM120A	transmembrane protein 120A
1,04	CYLC2	cylicin, basic protein of sperm head cytoskeleton 2
1,04	MBTPS2; YY2	membrane bound transcription factor peptidase, site 2; YY2 transcription factor
1,04	WNT7B	wingless-type MMTV integration site family, member 7B
1,04	GTF2H3	general transcription factor IIH subunit 3
1,04	ABT1	activator of basal transcription 1
1,04	HIST1H2BG	histone cluster 1, H2bg
1,04	TMEM87A	transmembrane protein 87A
1,04	MRPL21	mitochondrial ribosomal protein L21
1,04	SPRYD3	SPRY domain containing 3
1,04	NFYC	nuclear transcription factor Y subunit gamma
1,04	ADAMTS7	ADAM metallopeptidase with thrombospondin type 1 motif 7
1,04	DDX19A	DEAD (Asp-Glu-Ala-Asp) box polypeptide 19A
1,04	NINJ2	ninjurin 2
1,04	RBFOX3	RNA binding protein, fox-1 homolog (C. elegans) 3
1,04	GAS2L2	growth arrest-specific 2 like 2
1,04	PCK1	phosphoenolpyruvate carboxykinase 1 (soluble)
1,04	SLC16A9	solute carrier family 16, member 9
1,04	OR2T3	olfactory receptor, family 2, subfamily T, member 3
1,04	LHX6	LIM homeobox 6
1,04	IZUMO3	IZUMO family member 3
1,04	LINGO3	leucine rich repeat and Ig domain containing 3
1,04	RAVER2	ribonucleoprotein, PTB-binding 2
1,04	KIDINS220	kinase D-interacting substrate 220kDa
1,04	RUNX2	runt-related transcription factor 2
1,04	AOC3	amine oxidase, copper containing 3
1,04	ASMT	Homo sapiens acetylserotonin O-methyltransferase (ASMT), transcript variant 2, mRNA.
1,04	ABCF3	ATP binding cassette subfamily F member 3

1,04	GNL3L	guanine nucleotide binding protein-like 3 (nucleolar)-like
1,04	IGSF1	immunoglobulin superfamily, member 1
1,04	USP6NL	USP6 N-terminal like
1,04	UBOX5; FASTKD5	U-box domain containing 5; FAST kinase domains 5
1,04	TLE3	transducin-like enhancer of split 3
1,04	BCL7C	B-cell CLL/lymphoma 7C
1,04	OR5A2	olfactory receptor, family 5, subfamily A, member 2
1,04	MYH9	Zhang2013 ALT_ACCEPTOR, ALT_DONOR, coding, INTERNAL, intronic best transcript NM_002473
1,04	THUMPD1	THUMP domain containing 1
1,04	KRTAP5-8	keratin associated protein 5-8
1,04	DNAJA2	DnaJ (Hsp40) homolog, subfamily A, member 2
1,04	SLC16A1	solute carrier family 16 (monocarboxylate transporter), member 1
1,04	H3F3B	H3 histone, family 3B (H3.3B)
1,04	CD47	CD47 molecule
1,04	GPR31	G protein-coupled receptor 31
1,04	CALR3	calreticulin 3
1,04	LNP1	leukemia NUP98 fusion partner 1
1,04	NIPA1	non imprinted in Prader-Willi/Angelman syndrome 1
1,04	MFSD14C	major facilitator superfamily domain containing 14C
1,04	COL9A2	collagen, type IX, alpha 2
1,04	COL6A6	collagen, type VI, alpha 6
1,04	UNC13A	unc-13 homolog A (C. elegans)
1,04	C6orf132	Zhang2013 ALT_ACCEPTOR, ALT_DONOR, coding, INTERNAL, intronic best transcript NM_001164446
1,04	RBM19	RNA binding motif protein 19
1,04	TMEM57	transmembrane protein 57
1,04	ZFR	Transcript Identified by AceView, Entrez Gene ID(s) 51663
1,04	SLURP1	secreted LY6/PLAUR domain containing 1
1,04	HOXC6; HOXC4; HOXC5	homeobox C6; homeobox C4; homeobox C5
1,04	MCM8	minichromosome maintenance 8 homologous recombination repair factor
1,03	SCFD2	sec1 family domain containing 2
1,03	IDNK	idnK, gluconokinase homolog (E. coli)
1,03	ZNF429	zinc finger protein 429
1,03	KCNJ5	potassium channel, inwardly rectifying subfamily J, member 5
1,03	OR2T11	olfactory receptor, family 2, subfamily T, member 11 (gene/pseudogene)
1,03	AWAT1	acyl-CoA wax alcohol acyltransferase 1
1,03	LRRC46	leucine rich repeat containing 46
1,03	MUC21	mucin 21, cell surface associated
1,03	TTBK1	tau tubulin kinase 1

1,03	MTRNR2L2; MTRNR2L12	MT-RNR2-like 2; MT-RNR2-like 12
1,03	ADAM10	ADAM metallopeptidase domain 10
1,03	GRK5	G protein-coupled receptor kinase 5
1,03	GUCY2C	guanylate cyclase 2C
1,03	ADH1C	alcohol dehydrogenase 1C (class I), gamma polypeptide
1,03	TTLL10	tubulin tyrosine ligase-like family member 10
1,03	PSMC4	proteasome 26S subunit, ATPase 4 [Source:HGNC Symbol;Acc:HGNC:9551]
1,03	CDK5RAP1	CDK5 regulatory subunit associated protein 1
1,03	ENC1	ectodermal-neural cortex 1 (with BTB domain)
1,03	BECN2	beclin 2
1,03	SPIDR	scaffolding protein involved in DNA repair
1,03	ADAM15	ADAM metallopeptidase domain 15
1,03	SIGLECL1	SIGLEC family like 1
1,03	POR; MIR4651	P450 (cytochrome) oxidoreductase; microRNA 4651
1,03	DCLRE1C	DNA cross-link repair 1C
1,03	HMBS	hydroxymethylbilane synthase
1,03	TIPARP	Memczak2013 ANTISENSE, CDS, coding, INTERNAL best transcript NM_001184717
1,03	SLC26A1	solute carrier family 26 (anion exchanger), member 1
1,03	GGNBP2	gametogenetin binding protein 2
1,03	GPR150	G protein-coupled receptor 150
1,03	DCAF6	DDB1 and CUL4 associated factor 6
1,03	DPEP1	dipeptidase 1 (renal)
1,03	CDH15	cadherin 15, type 1, M-cadherin (myotubule)
1,03	ILK	integrin linked kinase
1,03	CCDC84	coiled-coil domain containing 84
1,03	APELA	apelin receptor early endogenous ligand
1,03	ERCC6L	excision repair cross-complementation group 6-like
1,03	FUBP3	far upstream element (FUSE) binding protein 3
1,03	CD300LD	CD300 molecule-like family member d
1,03	NKX6-3	NK6 homeobox 3
1,03	EWSR1	EWS RNA binding protein 1
1,03	NDUFB11	NADH dehydrogenase (ubiquinone) 1 beta subcomplex, 11, 17.3kDa
1,03	FAM209B	family with sequence similarity 209, member B
1,03	C11orf21	chromosome 11 open reading frame 21
1,03	KLRC3	killer cell lectin-like receptor subfamily C, member 3
1,03	ZBTB6	zinc finger and BTB domain containing 6
1,03	C1QTNF3-AMACR	C1QTNF3-AMACR readthrough (NMD candidate)
1,03	CSF3R	Memczak2013 ANTISENSE, CDS, coding, INTERNAL best transcript NM_156039
1,03	ZNF81	zinc finger protein 81
1,03	NPRL2	NPR2-like, GATOR1 complex subunit
1,03	PPWD1	peptidylprolyl isomerase domain and WD repeat containing 1
1,03	TBX22	T-box 22

1,03	ORC2	origin recognition complex subunit 2
1,03	KRTAP4-12	keratin associated protein 4-12
1,03	ZDHHC18	zinc finger, DHHC-type containing 18
1,03	NUP210L; MIR5698	nucleoporin 210kDa like; microRNA 5698
1,03	TCERG1L	transcription elongation regulator 1-like
1,03	TOMM70A	translocase of outer mitochondrial membrane 70 homolog A (S. cerevisiae)
1,03	LMBR1	limb development membrane protein 1
1,03	RHOA	ras homolog family member A
1,03	CAND2	cullin-associated and neddylation-dissociated 2 (putative)
1,03	BTG1; LINC01619	B-cell translocation gene 1, anti-proliferative; long intergenic non-protein coding RNA 1619
1,03	NBL1	neuroblastoma 1, DAN family BMP antagonist
1,03	SAG	S-antigen; retina and pineal gland (arrestin)
1,03	MTFR1L	mitochondrial fission regulator 1-like
1,03	BTK	Bruton agammaglobulinemia tyrosine kinase
1,03	ZNF512	zinc finger protein 512
1,03	TMEM131	transmembrane protein 131
1,03	TRIM31	tripartite motif containing 31
1,03	FAM83E	family with sequence similarity 83, member E
1,03	RHO	rhodopsin
1,03	ZKSCAN8	zinc finger with KRAB and SCAN domains 8
1,03	NGRN; TTLL13P	neugrin, neurite outgrowth associated; tubulin tyrosine ligase-like family member 13, pseudogene
1,03	TOMM22	translocase of outer mitochondrial membrane 22 homolog (yeast)
1,03	C17orf96	chromosome 17 open reading frame 96
1,03	CCL17	chemokine (C-C motif) ligand 17
1,03	KREMEN2	kringle containing transmembrane protein 2
1,03	PCDH7	protocadherin 7
1,03	ZDHHC3	zinc finger, DHHC-type containing 3
1,03	RITA1	RBPJ interacting and tubulin associated 1
1,03	UGT2B7	UDP glucuronosyltransferase 2 family, polypeptide B7
1,03	PHC3	polyhomeotic homolog 3 (Drosophila)
1,03	NPM1	Zhang2013 ALT_ACCEPTOR, ALT_DONOR, coding, INTERNAL, intronic best transcript NM_002520
1,03	BAALC-AS2	BAALC antisense RNA 2
1,03	SLC46A1	solute carrier family 46 (folate transporter), member 1
1,03	PLPP2	phospholipid phosphatase 2
1,03	ECHDC2	enoyl-CoA hydratase domain containing 2
1,03	TFAP2B	transcription factor AP-2 beta (activating enhancer binding protein 2 beta)
1,03	C2orf44	chromosome 2 open reading frame 44
1,03	COLEC12	collectin sub-family member 12
1,03	C5orf66	chromosome 5 open reading frame 66
1,03	ATG4C	autophagy related 4C, cysteine peptidase

1,03	CLK2	CDC like kinase 2
1,03	ADAMTS12	ADAM metallopeptidase with thrombospondin type 1 motif 12
1,03	LRMP	lymphoid-restricted membrane protein
1,03	HOXD10	homeobox D10
1,03	CRYBA1	crystallin beta A1
1,03	TP53TG3B	TP53 target 3B
1,03	TP53TG3B; TP53TG3C; TP53TG3; LOC102723655; RP11-23E10.6	Homo sapiens TP53 target 3B (TP53TG3B), transcript variant 1, mRNA.; Homo sapiens TP53 target 3C (TP53TG3C), transcript variant 1, mRNA.; Homo sapiens TP53 target 3 (TP53TG3), transcript variant 1, mRNA.; Homo sapiens TP53 target 3 (TP53TG3), transcript variant 3, non-coding RNA.; Homo sapiens TP53 target 3B (TP53TG3B), transcript variant 3, non-coding RNA.; Homo sapiens TP53 target 3 (TP53TG3), transcript variant 4, non-coding RNA.; Homo sapiens TP53 target 3C (TP53TG3C), transcript variant 3, non-coding RNA.; TP53-target gene 3 protein; Homo sapiens TP53 target 3, mRNA (cDNA clone MGC:119889 IMAGE:40015196), complete cds.; Homo sapiens TP53 target 3, mRNA (cDNA clone MGC:119888 IMAGE:40015195), complete cds.; TP53-target gene 3 protein [Source:UniProtKB/Swiss-Prot;Acc:Q9ULZ0]
1,03	TP53TG3B; TP53TG3C; TP53TG3; LOC102723655; hotema; RP11-1277H1.5	Homo sapiens TP53 target 3B (TP53TG3B), transcript variant 1, mRNA.; Homo sapiens TP53 target 3C (TP53TG3C), transcript variant 1, mRNA.; Homo sapiens TP53 target 3 (TP53TG3), transcript variant 1, mRNA.; Homo sapiens TP53 target 3 (TP53TG3), transcript variant 3, non-coding RNA.; Homo sapiens TP53 target 3B (TP53TG3B), transcript variant 3, non-coding RNA.; Homo sapiens TP53 target 3 (TP53TG3), transcript variant 4, non-coding RNA.; Homo sapiens TP53 target 3C (TP53TG3C), transcript variant 3, non-coding RNA.; TP53-target gene 3 protein; Homo sapiens TP53 target 3, mRNA (cDNA clone MGC:119889 IMAGE:40015196), complete cds.; Homo sapiens TP53 target 3, mRNA (cDNA clone MGC:119888 IMAGE:40015195), complete cds.; Transcript Identified by AceView; TP53-target gene 3 protein [Source:UniProtKB/Swiss-Prot;Acc:Q9ULZ0]
1,03	MTRNR2L8; MIR4485	MT-RNR2-like 8; microRNA 4485
1,03	TRMU	tRNA 5-methylaminomethyl-2-thiouridylate methyltransferase
1,03	PCIF1	PDX1 C-terminal inhibiting factor 1
1,03	NANS	N-acetylneuraminic acid synthase
1,03	TRIM25; MIR3614	tripartite motif containing 25; microRNA 3614

1,03	KIAA1143	KIAA1143 [Source:HGNC Symbol;Acc:HGNC:29198]
1,03	LRRFIP1	leucine rich repeat (in FLII) interacting protein 1
1,03	ANGPT2	angiopoietin 2
1,03	HIST1H4J	histone cluster 1, H4j
1,03	HIST2H2BF	histone cluster 2, H2bf
1,03	MRPL38	mitochondrial ribosomal protein L38
1,03	ATXN7	ataxin 7
1,03	LATS1	large tumor suppressor kinase 1
1,03	RALB	v-ral simian leukemia viral oncogene homolog B
1,03	TRHR	thyrotropin-releasing hormone receptor
1,03	GLCCI1	glucocorticoid induced 1
1,03	ZDHHC11	zinc finger, DHHC-type containing 11
1,03	HIGD1A	HIG1 hypoxia inducible domain family, member 1A
1,03	SAG	S-antigen; retina and pineal gland (arrestin)
1,03	YJEFN3	YjeF N-terminal domain containing 3
1,03	CYP2D6	cytochrome P450, family 2, subfamily D, polypeptide 6
1,03	EDA2R	ectodysplasin A2 receptor
1,03	KIR3DX1	killer cell immunoglobulin-like receptor, three domains, X1
1,03	C9orf40	chromosome 9 open reading frame 40
1,03	HYDIN	HYDIN, axonemal central pair apparatus protein
1,03	SCGB2A1	secretoglobin, family 2A, member 1
1,03	CCDC150	coiled-coil domain containing 150
1,03	FBXO46	F-box protein 46
1,03	KCNQ1	potassium channel, voltage gated KQT-like subfamily Q, member 1
1,03	SAMD15	sterile alpha motif domain containing 15
1,03	NAALADL1	N-acetylated alpha-linked acidic dipeptidase-like 1
1,03	CYP4A22	cytochrome P450, family 4, subfamily A, polypeptide 22
1,03	ABCA2	ATP binding cassette subfamily A member 2
1,03	SPDYE4	speedy/RINGO cell cycle regulator family member E4
1,03	FAM53C	family with sequence similarity 53, member C
1,03	PINX1; MIR1322	PIN2/TERF1 interacting, telomerase inhibitor 1; microRNA 1322
1,03	LOC105371242; AC243756.1	peptidyl-prolyl cis-trans isomerase A-like 4G; Peptidyl-prolyl cis-trans isomerase [Source:UniProtKB/TrEMBL;Acc:A0A075B767]
1,03	ATOH8	atonal bHLH transcription factor 8
1,03	EXD3	exonuclease 3-5 domain containing 3
1,03	NGLY1	N-glycanase 1
1,03	CTSW	cathepsin W
1,03	LYL1	Memczak2013 ANTISENSE, coding, INTERNAL, UTR5 best transcript NM_005583
1,03	VPS33A	vacuolar protein sorting 33 homolog A (S. cerevisiae)
1,03	RIOK2	RIO kinase 2
1,03	RANGRF	RAN guanine nucleotide release factor

1,03	KCMF1	potassium channel modulatory factor 1
1,03	ELF4	E74-like factor 4 (ets domain transcription factor)
1,03	SCGB3A2	secretoglobin, family 3A, member 2
1,03	ELFN2	Transcript Identified by AceView, Entrez Gene ID(s) 114794
1,03	BCL2L14	BCL2-like 14 (apoptosis facilitator)
1,03	GADD45A	growth arrest and DNA-damage-inducible, alpha
1,03	BIN2	bridging integrator 2
1,03	RLTPR	RGD motif, leucine rich repeats, tropomodulin domain and proline-rich containing
1,03	SPNS1	spinster homolog 1 (Drosophila)
1,03	SLC51A	solute carrier family 51, alpha subunit
1,03	LSR	lipolysis stimulated lipoprotein receptor
1,03	SFRP1	secreted frizzled-related protein 1
1,03	SFT2D2	SFT2 domain containing 2
1,03	A4GALT	alpha 1,4-galactosyltransferase
1,03	PCDHB7	protocadherin beta 7
1,03	RDH5	retinol dehydrogenase 5 (11-cis/9-cis)
1,03	LINC01429	long intergenic non-protein coding RNA 1429
1,03	TNFRSF12A	tumor necrosis factor receptor superfamily, member 12A
1,03	PSMC4	proteasome 26S subunit, ATPase 4 [Source:HGNC Symbol;Acc:HGNC:9551]
1,03	STAG3L1	stromal antigen 3-like 1 (pseudogene)
1,03	PTPN23	Jeck2013 ANTISENSE, CDS, coding, INTERNAL, intronic, OVCODE, OVEXON best transcript NM_015466
1,03	RNF10	ring finger protein 10
1,03	CHRAC1	chromatin accessibility complex 1
1,03	ATXN2L	ataxin 2-like
1,03	ZNF223	zinc finger protein 223
1,03	MMS22L	MMS22-like, DNA repair protein
1,03	PIGA	phosphatidylinositol glycan anchor biosynthesis class A
1,03	GLYATL3	glycine-N-acyltransferase-like 3
1,03	TCP10L2	t-complex 10-like 2
1,03	PARN	poly(A)-specific ribonuclease
1,03	AOC2	amine oxidase, copper containing 2 (retina-specific)
1,03	MTO1	mitochondrial tRNA translation optimization 1
1,03	TUBGCP3	Memczak2013 ANTISENSE, coding, INTERNAL, intronic best transcript NM_006322
1,03	SRPR	signal recognition particle receptor (docking protein)
1,03	NPRL3	NPR3-like, GATOR1 complex subunit
1,03	FAM25A	family with sequence similarity 25, member A
1,03	FAM19A3	family with sequence similarity 19 (chemokine (C-C motif)-like), member A3
1,03	SLC16A1	solute carrier family 16 (monocarboxylate transporter), member 1
1,03	CLEC4E	C-type lectin domain family 4, member E
1,03	THEG	theg spermatid protein

1,03	AMBP	alpha-1-microglobulin/bikunin precursor
1,03	KCNIP1	Kv channel interacting protein 1
1,03	RPAP1	RNA polymerase II associated protein 1
1,03	CDK4	cyclin-dependent kinase 4
1,03	SAMD9	sterile alpha motif domain containing 9
1,03	SLC25A26	solute carrier family 25 (S-adenosylmethionine carrier), member 26
1,03	POPDC2	popeye domain containing 2
1,03	CLHC1	clathrin heavy chain linker domain containing 1
1,03	SRP19; ZRSR1	signal recognition particle 19kDa; zinc finger (CCCH type), RNA binding motif and serine/arginine rich 1
1,03	PRR23B	proline rich 23B
1,03	C1orf95	chromosome 1 open reading frame 95
1,03	CCR7	chemokine (C-C motif) receptor 7
1,03	MBOAT2	membrane bound O-acyltransferase domain containing 2
1,03	MRPS30	mitochondrial ribosomal protein S30
1,03	TMEM50B	transmembrane protein 50B
1,03	FAM197Y1	family with sequence similarity 197, Y-linked, member 1
1,03	CYP2D6	cytochrome P450, family 2, subfamily D, polypeptide 6
1,03	LOR	loricrin
1,03	ZBTB42	zinc finger and BTB domain containing 42
1,03	ACAT1	acetyl-CoA acetyltransferase 1
1,03	KLHL3	Transcript Identified by AceView, Entrez Gene ID(s) 26249
1,03	SLIRP	SRA stem-loop interacting RNA binding protein
1,03	GAGE2B; GAGE2A; GAGE2C	G antigen 2B; G antigen 2A; G antigen 2C
1,03	SLC10A3	solute carrier family 10, member 3
1,03	OR2W3	olfactory receptor, family 2, subfamily W, member 3
1,03	BTF3L4	basic transcription factor 3-like 4
1,03	CAMSAP2	calmodulin regulated spectrin-associated protein family, member 2
1,03	HIST2H2AC	histone cluster 2, H2ac
1,03	KLHDC4	kelch domain containing 4
1,03	SSTR1	somatostatin receptor 1
1,03	CFL1	cofilin 1 (non-muscle)
1,03	C1QTNF4	C1q and tumor necrosis factor related protein 4
1,03	FAS	Fas cell surface death receptor
1,03	IGFL4	IGF like family member 4
1,03	TSPAN8	tetraspanin 8
1,03	SMAD3	SMAD family member 3
1,03	KRT35	keratin 35, type I
1,03	CDV3	CDV3 homolog (mouse)
1,03	MPST	mercaptopyruvate sulfurtransferase
1,03	ACTN4	actinin, alpha 4
1,03	TOX4	TOX high mobility group box family member 4

1,03	PLEKHH1	pleckstrin homology domain containing, family H (with MyTH4 domain) member 1
1,03	RSPRY1	ring finger and SPRY domain containing 1
1,03	MAP3K10	mitogen-activated protein kinase kinase kinase 10
1,03	SLC30A3	solute carrier family 30 (zinc transporter), member 3
1,03	FECH	ferrochelatase
1,03	C19orf73	chromosome 19 open reading frame 73
1,03	PTAR1	protein prenyltransferase alpha subunit repeat containing 1
1,03	KLHL31	kelch-like family member 31
1,03	GGT6	gamma-glutamyltransferase 6
1,03	PADI1	peptidyl arginine deiminase, type I
1,03	RPL28; MIR6805	ribosomal protein L28; microRNA 6805
1,03	NOTCH1	notch 1
1,03	SMN1; SMN2	survival of motor neuron 1, telomeric; survival of motor neuron 2, centromeric
1,03	ZNF648	zinc finger protein 648
1,03	SAMD4B	sterile alpha motif domain containing 4B
1,03	CLLU1OS	chronic lymphocytic leukemia up-regulated 1 opposite strand
1,03	OLIG2	oligodendrocyte lineage transcription factor 2
1,03	OR4Q3	olfactory receptor, family 4, subfamily Q, member 3
1,03	NOSTRIN	nitric oxide synthase trafficking
1,03	DHTKD1	dehydrogenase E1 and transketolase domain containing 1
1,03	METTL21B	methyltransferase like 21B
1,03	NPAS1	neuronal PAS domain protein 1
1,03	AFF4	Memczak2013 ANTISENSE, CDS, coding, INTERNAL best transcript NM_014423
1,03	RASGEF1C	RasGEF domain family member 1C
1,03	CNTLN	centlein, centrosomal protein
1,03	FAAH2	fatty acid amide hydrolase 2
1,03	GYS1	glycogen synthase 1 (muscle)
1,03	SKIV2L2	superkiller viralicidic activity 2-like 2 (S. cerevisiae)
1,03	DYRK1B	dual specificity tyrosine-(Y)-phosphorylation regulated kinase 1B
1,03	ATAD2B	ATPase family, AAA domain containing 2B
1,03	CD55	CD55 molecule, decay accelerating factor for complement (Cromer blood group)
1,03	TRIT1	tRNA isopentenyltransferase 1
1,03	ANO6	anoctamin 6
1,03	DUSP7	dual specificity phosphatase 7
1,03	SF3A1	splicing factor 3a, subunit 1, 120kDa
1,03	RXFP2	relaxin/insulin-like family peptide receptor 2
1,03	DLEU2; MIR15A; MIR16-1; MIR3613	deleted in lymphocytic leukemia 2 (non-protein coding); microRNA 15a; microRNA 16-1; microRNA 3613
1,03	LRIG1	
1,03	EIF3F	Eukaryotic translation initiation factor 3 subunit F [Source:UniProtKB/Swiss-Prot;Acc:O00303]

1,03	GRWD1	glutamate-rich WD repeat containing 1
1,03	ADRA1D	adrenoceptor alpha 1D
1,03	AMBRA1	autophagy/beclin-1 regulator 1
1,03	MRGPRG-AS1	MRGPRG antisense RNA 1
1,03	HCG27	HLA complex group 27 (non-protein coding)
1,03	MCTP1	multiple C2 domains, transmembrane 1
1,03	OTOF	otoferlin
1,03	MMADHC	methylmalonic aciduria (cobalamin deficiency) cblD type, with homocystinuria
1,03	DDX46	DEAD (Asp-Glu-Ala-Asp) box polypeptide 46
1,03	PSPH	phosphoserine phosphatase
1,03	HECTD2	Transcript Identified by AceView, Entrez Gene ID(s) 143279
1,03	LMO7DN	LMO7 downstream neighbor
1,03	LANCL2	LanC lantibiotic synthetase component C-like 2 (bacterial)
1,03	RTP5	receptor (chemosensory) transporter protein 5 (putative)
1,03	GOLGA8O	golgin A8 family, member O
1,03	OR5B3	olfactory receptor, family 5, subfamily B, member 3
1,03	SNAPC1	small nuclear RNA activating complex polypeptide 1
1,03	ABHD3	abhydrolase domain containing 3
1,03	TFDP1	transcription factor Dp-1
1,03	HMBS	hydroxymethylbilane synthase
1,03	RP11-351M8.2; MESDC2; RP11-351M8.1	Transcript Identified by AceView, Entrez Gene ID(s) 23184; novel transcript, antisense to CEMIP
1,03	ATF6	activating transcription factor 6
1,03	PIK3CA	phosphatidylinositol-4,5-bisphosphate 3-kinase, catalytic subunit alpha
1,03	POLR3K	polymerase (RNA) III (DNA directed) polypeptide K, 12.3 kDa
1,03	NET1	neuroepithelial cell transforming 1
1,03	WNT7A	wingless-type MMTV integration site family, member 7A
1,03	NKAIN1	Na+/K+ transporting ATPase interacting 1
1,03	OR51B6	olfactory receptor, family 51, subfamily B, member 6
1,03	KLK11	kallikrein related peptidase 11
1,03	CLDN10	claudin 10
1,03	CCL28	chemokine (C-C motif) ligand 28
1,03	THBD	thrombomodulin
1,03	PPP2R2A	protein phosphatase 2, regulatory subunit B, alpha
1,03	C17orf107	chromosome 17 open reading frame 107
1,03	ZSWIM8	zinc finger, SWIM-type containing 8
1,03	SMIM10L1	small integral membrane protein 10 like 1
1,03	RBBP6	retinoblastoma binding protein 6
1,03	POLR3H	polymerase (RNA) III (DNA directed) polypeptide H (22.9kD)
1,03	SLC9A8	solute carrier family 9, subfamily A (NHE8, cation proton antiporter 8), member 8

1,03	GSPT1	G1 to S phase transition 1
1,03	HIST2H4A; HIST2H4B	histone cluster 2, H4a; histone cluster 2, H4b
1,03	NCMAP	noncompact myelin associated protein
1,03	RPS28	ribosomal protein S28
1,03	PLIN3	perilipin 3
1,03	PPP1R26	protein phosphatase 1, regulatory subunit 26
1,03	CHAC2	ChaC, cation transport regulator homolog 2 (E. coli)
1,03	CASD1	CAS1 domain containing 1
1,03	EPPIN	epididymal peptidase inhibitor
1,03	ATP13A3	Transcript Identified by AceView, Entrez Gene ID(s) 79572
1,03	RALGPS1	Ral GEF with PH domain and SH3 binding motif 1
1,03	BET1	Bet1 golgi vesicular membrane trafficking protein
1,03	LYRM5	LYR motif containing 5
1,03	MREG	melanoregulin
1,03	TMSB15B	thymosin beta 15B
1,03	AKT1S1	AKT1 substrate 1 (proline rich)
1,03	OPA1	optic atrophy 1 (autosomal dominant)
1,03	NETO2	neuropilin (NRP) and tolloid (TLL)-like 2
1,03	NPAS3	neuronal PAS domain protein 3
1,03	CA12	carbonic anhydrase XII
1,03	MMP3	matrix metallopeptidase 3
1,03	ORAI3	ORAI calcium release-activated calcium modulator 3
1,03	CLDN11	claudin 11
1,03	SEC24C	SEC24 homolog C, COPII coat complex component
1,03	ARF6	ADP-ribosylation factor 6
1,03	AGPS	alkylglycerone phosphate synthase
1,03	GPR157	G protein-coupled receptor 157
1,03	ADAMTSL2	ADAMTS like 2
1,03	AP1B1	Zhang2013 ALT_ACCEPTOR, ALT_DONOR, coding, INTERNAL, intronic best transcript NM_001127
1,03	BTNL10	butyrophilin-like 10
1,03	CDK6	cyclin-dependent kinase 6
1,03	GAL3ST3	galactose-3-O-sulfotransferase 3
1,03	FLRT1	fibronectin leucine rich transmembrane protein 1
1,03	MAFIP; TEKT4P2	MAFF interacting protein (pseudogene); tektin 4 pseudogene 2
1,03	TMEM147	transmembrane protein 147
1,03	RCN1	reticulocalbin 1, EF-hand calcium binding domain
1,03	PRAC2	prostate cancer susceptibility candidate 2
1,03	UVSSA	UV stimulated scaffold protein A
1,03	ATP6V0A4	ATPase, H+ transporting, lysosomal V0 subunit a4
1,03	NKD1	naked cuticle homolog 1 (Drosophila)
1,03	FAM83D	family with sequence similarity 83, member D
1,03	ROPN1B	rhophilin associated tail protein 1B
1,03	MTTP	microsomal triglyceride transfer protein
1,03	KRTAP9-9	keratin associated protein 9-9
1,03	MEP1B	meprin A, beta

1,03	DNAJC14	DnaJ (Hsp40) homolog, subfamily C, member 14
1,03	dorsnarby; IGF2BP2	Transcript Identified by AceView; Zhang2013 ALT_ACCEPTOR, ALT_DONOR, coding, INTERNAL, intronic best transcript NM_006548
1,03	RALA	v-ral simian leukemia viral oncogene homolog A (ras related)
1,03	ENAH	enabled homolog (Drosophila)
1,03	FKBP14	FK506 binding protein 14
1,03	C1orf162	chromosome 1 open reading frame 162
1,03	DNAH5	dynein, axonemal, heavy chain 5
1,03	LY6D	lymphocyte antigen 6 complex, locus D
1,03	GLIPR1	GLI pathogenesis-related 1
1,03	ASNS	asparagine synthetase (glutamine-hydrolyzing)
1,03	NDUFA5	NADH dehydrogenase (ubiquinone) 1 alpha subcomplex, 5
1,03	EGFL7	EGF-like-domain, multiple 7
1,03	SQSTM1	sequestosome 1
1,03	TIMM21	translocase of inner mitochondrial membrane 21 homolog (yeast)
1,03	HERC4	HECT and RLD domain containing E3 ubiquitin protein ligase 4
1,03	WDR54	WD repeat domain 54
1,03	BHLHA15	basic helix-loop-helix family, member a15
1,03	RP2	retinitis pigmentosa 2 (X-linked recessive)
1,03	DOK1	docking protein 1
1,03	EML5	echinoderm microtubule associated protein like 5
1,03	LTA4H	leukotriene A4 hydrolase
1,03	KRTAP10-3	keratin associated protein 10-3
1,03	KLC2	kinesin light chain 2
1,03	TOMM34	translocase of outer mitochondrial membrane 34
1,03	DYRK1A	dual specificity tyrosine-(Y)-phosphorylation regulated kinase 1A
1,03	PPP2R5A	protein phosphatase 2, regulatory subunit B, alpha
1,03	SCAMP2	secretory carrier membrane protein 2
1,03	AADACL2	arylacetamide deacetylase-like 2
1,03	C7orf49	chromosome 7 open reading frame 49
1,03	FAM21C	family with sequence similarity 21, member C
1,03	MFSD14B	major facilitator superfamily domain containing 14B
1,03	FOXL2	forkhead box L2
1,03	SLC5A6	solute carrier family 5 (sodium/multivitamin and iodide cotransporter), member 6
1,03	PSMB1	proteasome subunit beta 1
1,03	CLPTM1	cleft lip and palate associated transmembrane protein 1
1,03	KLHDC7A	kelch domain containing 7A
1,03	SPRR1B	small proline-rich protein 1B
1,03	AQP2	aquaporin 2 (collecting duct)
1,03	RP11-295P9.3; PRPF18	novel transcript; Transcript Identified by AceView, Entrez Gene ID(s) 8559; RP11-295P9.3 (from geneSymbol)

1,03	TSN	translin
1,03	MLXIP	MLX interacting protein
1,03	SLC25A24	solute carrier family 25 (mitochondrial carrier; phosphate carrier), member 24
1,03	CPQ	carboxypeptidase Q
1,03	RAB14	RAB14, member RAS oncogene family
1,03	UPF3B	UPF3 regulator of nonsense transcripts homolog B (yeast)
1,03	MAATS1	MYCBP-associated, testis expressed 1
1,03	EXOC3L2	exocyst complex component 3-like 2
1,03	INSL6	insulin-like 6
1,03	ZCCHC8	zinc finger, CCHC domain containing 8
1,03	YWHAEP7	tyrosine 3-monooxygenase/tryptophan 5-monooxygenase activation protein, epsilon pseudogene 7
1,03	TLL2	tolloid like 2
1,03	PWP1	PWP1 homolog, endonuclein
1,03	TSACC	TSSK6 activating co-chaperone
1,03	HHAT	hedgehog acyltransferase
1,03	TMEM229A	transmembrane protein 229A
1,03	ALG11; UTP14C	ALG11, alpha-1,2-mannosyltransferase; UTP14, U3 small nucleolar ribonucleoprotein, homolog C (yeast)
1,03	CD248	CD248 molecule, endosialin
1,03	CCNB1IP1; SNORD126	cyclin B1 interacting protein 1, E3 ubiquitin protein ligase; small nucleolar RNA, C/D box 126
1,03	PRSS42	protease, serine 42
1,03	SLC25A26	solute carrier family 25 (S-adenosylmethionine carrier), member 26
1,03	OR51T1	olfactory receptor, family 51, subfamily T, member 1
1,03	VPS11	VPS11, CORVET/HOPS core subunit [Source:HGNC Symbol;Acc:HGNC:14583]
1,03	CBLL1	Cbl proto-oncogene-like 1, E3 ubiquitin protein ligase
1,03	C17orf100	chromosome 17 open reading frame 100
1,03	PPP1R15A	protein phosphatase 1, regulatory subunit 15A
1,03	CAST	calpastatin
1,03	NTAN1	N-terminal asparagine amidase
1,03	STAG1	stromal antigen 1
1,03	RAD51	RAD51 recombinase
1,03	SDHAF2	succinate dehydrogenase complex assembly factor 2
1,03	PA2G4	proliferation-associated 2G4
1,03	ZMYND10	zinc finger, MYND-type containing 10
1,03	MAN1A1	mannosidase, alpha, class 1A, member 1
1,03	BAIAP3	BAI1-associated protein 3
1,03	CDR2	cerebellar degeneration related protein 2
1,03	PRKAA2	protein kinase, AMP-activated, alpha 2 catalytic subunit
1,03	MPPED2	metallophosphoesterase domain containing 2
1,03	CEP97	centrosomal protein 97kDa
1,03	OTOS	otospiralin
1,03	SCUBE2	signal peptide, CUB domain, EGF-like 2

1,03	SLC25A11	solute carrier family 25 (mitochondrial carrier; oxoglutarate carrier), member 11
1,03	MEN1	multiple endocrine neoplasia I
1,03	MMP28	matrix metallopeptidase 28
1,03	NFATC4	nuclear factor of activated T-cells, cytoplasmic, calcineurin-dependent 4
1,03	MRGPRX2	MAS-related GPR, member X2
1,03	EFHC2	EF-hand domain (C-terminal) containing 2
1,03	HOXD3	homeobox D3
1,03	SUFU	suppressor of fused homolog (Drosophila)
1,03	SLC29A1	solute carrier family 29 (equilibrative nucleoside transporter), member 1
1,03	SLC6A4	solute carrier family 6 (neurotransmitter transporter), member 4
1,03	ANP32B	acidic nuclear phosphoprotein 32 family member B
1,03	FSHR	follicle stimulating hormone receptor
1,03	MLXIP	MLX interacting protein
1,03	LAPTM4B	lysosomal protein transmembrane 4 beta
1,03	PHF12	PHD finger protein 12
1,03	SSB	Sjogren syndrome antigen B (autoantigen La)
1,03	FGF19	fibroblast growth factor 19
1,03	DHRS11	dehydrogenase/reductase (SDR family) member 11
1,03	MICB	MHC class I polypeptide-related sequence B
1,03	ZNF470	zinc finger protein 470
1,03	SLC45A2	solute carrier family 45, member 2
1,03	DGCR6L	DiGeorge syndrome critical region gene 6-like
1,03	FIP1L1	factor interacting with PAPOLA and CPSF1
1,03	CYP2D6	cytochrome P450, family 2, subfamily D, polypeptide 6
1,03	PRKCQ	protein kinase C, theta
1,03	KIF4A	kinesin family member 4A
1,03	CHMP4B	charged multivesicular body protein 4B
1,03	LINC00615	long intergenic non-protein coding RNA 615
1,03	DUSP3	dual specificity phosphatase 3
1,03	CLN5	ceroid-lipofuscinosis, neuronal 5
1,03	COIL	coilin
1,03	ACTRT3	actin-related protein T3
1,03	ZNF32-AS3	ZNF32 antisense RNA 3
1,03	SPRR4	small proline-rich protein 4
1,03	ZPBP2	zona pellucida binding protein 2
1,03	RGL4	ral guanine nucleotide dissociation stimulator-like 4
1,03	GNB2L1; SNORD95; SNORD96A	guanine nucleotide binding protein (G protein), beta polypeptide 2-like 1; small nucleolar RNA, C/D box 95; small nucleolar RNA, C/D box 96A
1,03	NFATC1	nuclear factor of activated T-cells, cytoplasmic, calcineurin-dependent 1
1,03	MYCBP; GJA9	MYC binding protein; gap junction protein alpha 9
1,03	NEMF	nuclear export mediator factor
1,03	CDC42	cell division cycle 42
1,03	UBA2	ubiquitin-like modifier activating enzyme 2

1,03	ZFYVE16	zinc finger, FYVE domain containing 16
1,03	ZNF26	zinc finger protein 26
1,03	CCNI	cyclin I
1,03	IPMK	inositol polyphosphate multikinase
1,03	ANO5	anoctamin 5
1,03	SP8	Sp8 transcription factor
1,03	UBE3A	ubiquitin protein ligase E3A
1,03	ZMIZ1	Zhang2013 ALT_ACCEPTOR, ALT_DONOR, coding, INTERNAL, intronic best transcript NM_020338
1,03	SCNM1; TNFAIP8L2	sodium channel modifier 1; tumor necrosis factor, alpha-induced protein 8-like 2
1,03	MAX	MYC associated factor X
1,03	FUCA2	fucosidase, alpha-L- 2, plasma
1,03	PLK1	polo-like kinase 1
1,03	NUAK1	NUAK family, SNF1-like kinase, 1
1,03	HMGB1	high mobility group box 1
1,03	DCLK2	doublecortin-like kinase 2
1,03	RECQL4	RecQ helicase-like 4
1,03	PTBP2	polypyrimidine tract binding protein 2
1,03	EIF3B	eukaryotic translation initiation factor 3, subunit B
1,03	CXXC5	Memczak2013 ANTISENSE, coding, INTERNAL, intronic best transcript NM_016463
1,03	SMNDC1	survival motor neuron domain containing 1
1,03	LGALSL	lectin, galactoside-binding-like
1,03	MROH1	maestro heat-like repeat family member 1
1,03	SPPL2B	signal peptide peptidase like 2B
1,03	ZAR1	zygote arrest 1
1,03	TPBG	trophoblast glycoprotein
1,03	MMACHC	methylmalonic aciduria (cobalamin deficiency) cblC type, with homocystinuria
1,03	OSBPL5	oxysterol binding protein-like 5
1,03	PRAMEF11	PRAME family member 11
1,03	ATF2	activating transcription factor 2
1,03	RGS19	regulator of G-protein signaling 19
1,03	SIK3	SIK family kinase 3
1,03	POLR2C	polymerase (RNA) II (DNA directed) polypeptide C, 33kDa
1,03	PLCXD1	phosphatidylinositol-specific phospholipase C, X domain containing 1
1,03	THRB	thyroid hormone receptor, beta
1,03	ZNF621	zinc finger protein 621
1,03	AMELX	amelogenin, X-linked
1,03	TAF9; AK6	TAF9 RNA polymerase II, TATA box binding protein (TBP)-associated factor, 32kDa; adenylate kinase 6
1,03	SLC7A6OS	solute carrier family 7, member 6 opposite strand
1,03	IDH3A	isocitrate dehydrogenase 3 (NAD+) alpha
1,03	CSAD	cysteine sulfinic acid decarboxylase

1,03	SRD5A1	steroid-5-alpha-reductase, alpha polypeptide 1 (3-oxo-5 alpha-steroid delta 4-dehydrogenase alpha 1)
1,03	ATF7IP	activating transcription factor 7 interacting protein
1,03	GYG1	glycogenin 1
1,03	AIFM2	Memczak2013 ANTISENSE, coding, INTERNAL, intronic best transcript NM_001198696
1,03	TP53TG5	TP53 target 5
1,03	SEMA4D	sema domain, immunoglobulin domain (Ig), transmembrane domain (TM) and short cytoplasmic domain, (semaphorin) 4D
1,03	DHRS4L1	dehydrogenase/reductase (SDR family) member 4 like 1
1,03	PCBP3	poly(rC) binding protein 3
1,03	C4orf17	chromosome 4 open reading frame 17
1,03	DPP4	Transcript Identified by AceView, Entrez Gene ID(s) 1803
1,03	LAGE3	L antigen family, member 3
1,03	NUP153	nucleoporin 153kDa
1,03	ZNF300	zinc finger protein 300
1,03	TCTN1	tectonic family member 1
1,03	SLC7A6	solute carrier family 7 (amino acid transporter light chain, y+L system), member 6
1,03	TMPRSS9	transmembrane protease, serine 9
1,03	CDK19	cyclin-dependent kinase 19
1,03	ALG2	ALG2, alpha-1,3/1,6-mannosyltransferase
1,03	PGK2	phosphoglycerate kinase 2
1,03	PAX7	paired box 7
1,03	C14orf177	chromosome 14 open reading frame 177
1,03	HIST2H2BE	histone cluster 2, H2be
1,03	C6orf141	chromosome 6 open reading frame 141
1,03	FOXL1	forkhead box L1
1,03	INTS10	integrator complex subunit 10
1,03	PDLIM5	PDZ and LIM domain 5
1,03	VWA9	von Willebrand factor A domain containing 9
1,03	FLNA	Memczak2013 ANTISENSE, CDS, coding, INTERNAL best transcript NM_001456
1,03	TGFBR2	transforming growth factor beta receptor II
1,03	SCP2	Jeck2013 ANTISENSE, coding, INTERNAL, OVEXON, UTR3 best transcript NM_001007098
1,03	MEI1	Salzman2013 ALT_ACCEPTOR, ALT_DONOR, coding, INTERNAL, intronic best transcript NM_152513
1,03	DGAT2L6	diacylglycerol O-acyltransferase 2-like 6
1,03	CTIF; MIR4743	CBP80/20-dependent translation initiation factor; microRNA 4743
1,03	TJP1	Transcript Identified by AceView, Entrez Gene ID(s) 7082
1,03	AK7	adenylate kinase 7
1,03	AP2B1	adaptor-related protein complex 2, beta 1 subunit
1,03	YWHAZ	tyrosine 3-monooxygenase/tryptophan 5-monooxygenase activation protein, zeta

1,03	CCND2	cyclin D2
1,03	CLDN23	claudin 23
1,03	NAPEPLD	N-acyl phosphatidylethanolamine phospholipase D
1,03	SAMD4A	sterile alpha motif domain containing 4A
1,03	CHST10	carbohydrate sulfotransferase 10
1,03	NAPG	N-ethylmaleimide-sensitive factor attachment protein, gamma
1,03	GBA3	Jeck2013 ANTISENSE, coding, INTERNAL, intronic best transcript NM_020973
1,03	WNT10A	wingless-type MMTV integration site family, member 10A
1,03	CCDC84	coiled-coil domain containing 84
1,03	LGI1	leucine-rich, glioma inactivated 1
1,03	C7orf50	chromosome 7 open reading frame 50
1,03	OR7A10	olfactory receptor, family 7, subfamily A, member 10
1,03	UQCRH	ubiquinol-cytochrome c reductase hinge protein
1,03	RRAS2	related RAS viral (r-ras) oncogene homolog 2
1,03	DDX5; MIR3064; MIR5047	DEAD (Asp-Glu-Ala-Asp) box helicase 5; microRNA 3064; microRNA 5047
1,03	POLR1D	polymerase (RNA) I polypeptide D
1,03	AP3M1	adaptor-related protein complex 3, mu 1 subunit
1,03	SOX17	SRY box 17
1,03	ISCU	iron-sulfur cluster assembly enzyme
1,03	ITIH3	inter-alpha-trypsin inhibitor heavy chain 3
1,03	MAP9	microtubule-associated protein 9
1,03	PTPRQ	protein tyrosine phosphatase, receptor type, Q
1,03	ERCC6; ERCC6-PGBD3; PGBD3	excision repair cross-complementation group 6; ERCC6-PGBD3 readthrough; piggyBac transposable element derived 3
1,03	TBC1D3K	TBC1 domain family, member 3K
1,03	EPS8L2	EPS8-like 2
1,03	CDKN2AIP	CDKN2A interacting protein
1,03	SLC2A11	solute carrier family 2 (facilitated glucose transporter), member 11
1,03	ATF5; MIR4751	activating transcription factor 5; microRNA 4751
1,03	BANK1	B-cell scaffold protein with ankyrin repeats 1
1,03	HHLA1	HERV-H LTR-associating 1
1,03	NRAS	neuroblastoma RAS viral (v-ras) oncogene homolog
1,03	RAB3B	RAB3B, member RAS oncogene family
1,03	GALK2	galactokinase 2
1,03	CNIH3	cornichon family AMPA receptor auxiliary protein 3
1,03	ABLIM1	actin binding LIM protein 1
1,03	TROVE2	TROVE domain family, member 2
1,03	SCX	scleraxis bHLH transcription factor
1,03	PDHA2	pyruvate dehydrogenase (lipoamide) alpha 2
1,03	TNRC6B	Transcript Identified by AceView, Entrez Gene ID(s) 23112
1,03	COL1A2	Jeck2013 ANTISENSE, CDS, coding, INTERNAL, intronic, OVCODE, OVEXON best transcript NM_000089

1,03	SLC22A8	solute carrier family 22 (organic anion transporter), member 8
1,03	CDK16	cyclin-dependent kinase 16
1,03	VAMP3	vesicle associated membrane protein 3
1,03	FAM110C	family with sequence similarity 110, member C
1,03	TNP2	transition protein 2 (during histone to protamine replacement)
1,03	OR2M4	olfactory receptor, family 2, subfamily M, member 4
1,03	WNT3	wingless-type MMTV integration site family, member 3
1,03	C11orf65	chromosome 11 open reading frame 65
1,03	HEATR6	HEAT repeat containing 6
1,03	MICU2	mitochondrial calcium uptake 2
1,03	CYP11B1	cytochrome P450, family 11, subfamily B, polypeptide 1
1,03	HSPA14	heat shock 70kDa protein 14
1,03	C3orf36	chromosome 3 open reading frame 36
1,03	PRDX1	peroxiredoxin 1
1,03	CCDC94	coiled-coil domain containing 94
1,03	MDH1	malate dehydrogenase 1
1,03	BHLHE41	basic helix-loop-helix family, member e41
1,03	DGAT1; MIR6848	diacylglycerol O-acyltransferase 1; microRNA 6848
1,03	HLF	hepatic leukemia factor
1,03	XPO5	exportin 5
1,03	MIR1247	microRNA 1247
1,03	PTP4A2	protein tyrosine phosphatase type IVA, member 2
1,03	CACNB3	calcium channel, voltage-dependent, beta 3 subunit
1,03	TFPI2	tissue factor pathway inhibitor 2
1,03	SIT1	signaling threshold regulating transmembrane adaptor 1
1,03	ITSN1	Transcript Identified by AceView, Entrez Gene ID(s) 6453
1,03	LHX4-AS1; ACBD6	LHX4 antisense RNA 1; acyl-CoA binding domain containing 6
1,03	ILDR2	immunoglobulin-like domain containing receptor 2
1,03	MC5R	melanocortin 5 receptor
1,03	GPR27	G protein-coupled receptor 27
1,03	MPV17L2	MPV17 mitochondrial membrane protein-like 2
1,03	MFSD9	major facilitator superfamily domain containing 9
1,03	PXT1	peroxisomal, testis specific 1
1,03	MTBP	MDM2 binding protein
1,03	C8orf88	chromosome 8 open reading frame 88
1,03	MED20	mediator complex subunit 20
1,03	DCAF4	DDB1 and CUL4 associated factor 4
1,03	BOLA3	bolA family member 3
1,03	RCAN3	RCAN family member 3
1,03	IMPDH1	IMP (inosine 5-monophosphate) dehydrogenase 1
1,03	LSM1	LSM1 homolog, mRNA degradation associated
1,03	ARL5A	ADP-ribosylation factor like GTPase 5A

1,03	QRICH1	glutamine-rich 1
1,03	DCAKD	dephospho-CoA kinase domain containing
1,03	LRRC52	leucine rich repeat containing 52
1,03	ARAP2	ArfGAP with RhoGAP domain, ankyrin repeat and PH domain 2
1,03	FOXL2NB	FOXL2 neighbor
1,03	ZNF493	zinc finger protein 493
1,03	PM20D1	peptidase M20 domain containing 1
1,03	DAAM1	dishevelled associated activator of morphogenesis 1
1,03	IL23R	interleukin 23 receptor
1,03	ANKRD20A1	ankyrin repeat domain 20 family, member A1
1,03	TAF4	TAF4 RNA polymerase II, TATA box binding protein (TBP)-associated factor, 135kDa
1,03	ZNF672	zinc finger protein 672
1,03	DUSP4	dual specificity phosphatase 4
1,03	UAP1L1	UDP-N-acetylglucosamine pyrophosphorylase 1 like 1
1,03	TEX37	testis expressed 37
1,03	DENND1A	DENN/MADD domain containing 1A
1,03	WDR45B	WD repeat domain 45B
1,03	GPR89B	G protein-coupled receptor 89B
1,03	APOOL	apolipoprotein O-like
1,03	IL22RA2	interleukin 22 receptor, alpha 2
1,03	HMBS	hydroxymethylbilane synthase
1,03	AGBL5	ATP/GTP binding protein-like 5
1,03	SNX7	sorting nexin 7
1,03	PURA	purine-rich element binding protein A
1,03	TMEM60	transmembrane protein 60
1,03	MCFD2	multiple coagulation factor deficiency 2
1,03	TM2D1	TM2 domain containing 1
1,03	CD44	CD44 molecule (Indian blood group)
1,03	PTRHD1	peptidyl-tRNA hydrolase domain containing 1
1,03	CCNC	cyclin C
1,03	ZNF616	zinc finger protein 616
1,03	RPL7	ribosomal protein L7
1,03	HIRIP3	HIRA interacting protein 3
1,03	MVD	mevalonate (diphospho) decarboxylase
1,03	TMEM11	transmembrane protein 11
1,03	SCFD1	sec1 family domain containing 1
1,03	PTPRA; VPS16	protein tyrosine phosphatase, receptor type, A; vacuolar protein sorting 16 homolog (S. cerevisiae)
1,03	FAM168B	family with sequence similarity 168, member B
1,03	PRDM15	PR domain containing 15
1,03	LRRK2	leucine-rich repeat kinase 2
1,03	RYR2	ryanodine receptor 2 (cardiac)
1,03	KIAA0930	KIAA0930
1,03	PSEN1	presenilin 1
1,03	MAP10	microtubule-associated protein 10
1,03	IKZF5	IKAROS family zinc finger 5
1,03	ATL2	atlastin GTPase 2

1,03	DNAH14	dynein, axonemal, heavy chain 14
1,03	FGGY	FGGY carbohydrate kinase domain containing
1,03	COX6A2	cytochrome c oxidase subunit VIa polypeptide 2
1,03	NCAPD3	non-SMC condensin II complex subunit D3
1,03	LOC286238; RP13-60M5.2	uncharacterized LOC286238; Transcript Identified by AceView, Entrez Gene ID(s) 286238, RefSeq ID(s) NM_001100111; novel transcript
1,03	GRPR	gastrin-releasing peptide receptor
1,03	INTS1	Memczak2013 ANTISENSE, CDS, coding, INTERNAL best transcript NM_001080453
1,03	OR5P2	olfactory receptor, family 5, subfamily P, member 2
1,03	ZNF662	zinc finger protein 662
1,03	STC2	stanniocalcin 2
1,03	HNRNPDL	heterogeneous nuclear ribonucleoprotein D like
1,03	RAB3A	RAB3A, member RAS oncogene family
1,03	OPN1SW	opsin 1 (cone pigments), short-wave-sensitive
1,03	SLC51B	solute carrier family 51, beta subunit
1,03	GORASP2	golgi reassembly stacking protein 2
1,03	RNF212B	ring finger protein 212B
1,03	CERS1; GDF1	ceramide synthase 1; growth differentiation factor 1
1,03	GTF3C2	general transcription factor IIIC subunit 2
1,03	SLC2A2	solute carrier family 2 (facilitated glucose transporter), member 2
1,03	RTN4	reticulon 4
1,03	MAGEE2	MAGE family member E2
1,03	PKIG	protein kinase (cAMP-dependent, catalytic) inhibitor gamma
1,03	NCOR1	nuclear receptor corepressor 1
1,03	MRPL30	mitochondrial ribosomal protein L30
1,03	KDM4D	lysine (K)-specific demethylase 4D
1,03	COLGALT1	collagen beta(1-O)galactosyltransferase 1
1,03	SLCO3A1	solute carrier organic anion transporter family, member 3A1
1,03	SCG5	secretogranin V
1,03	KDM1A	Transcript Identified by AceView, Entrez Gene ID(s) 23028
1,03	FBXL18	F-box and leucine-rich repeat protein 18
1,03	BMPER	BMP binding endothelial regulator
1,03	IGF2BP2	insulin-like growth factor 2 mRNA binding protein 2
1,03	GNPDA2	glucosamine-6-phosphate deaminase 2
1,03	SHISA7	shisa family member 7
1,03	GABRB1	gamma-aminobutyric acid (GABA) A receptor, beta 1
1,03	UBE4B	ubiquitination factor E4B
1,03	PEA15	phosphoprotein enriched in astrocytes 15
1,03	HS6ST1	heparan sulfate 6-O-sulfotransferase 1
1,03	TBC1D27	TBC1 domain family, member 27
1,03	ANKRD28	ankyrin repeat domain 28
1,03	ZFC3H1	zinc finger, C3H1-type containing
1,03	EDEM2	ER degradation enhancer, mannosidase alpha-like 2

1,03	IRAK1BP1	interleukin 1 receptor associated kinase 1 binding protein 1
1,03	KCNQ4	potassium channel, voltage gated KQT-like subfamily Q, member 4
1,03	ARCN1	archain 1
1,03	OR9A4	olfactory receptor, family 9, subfamily A, member 4
1,03	KLK9	kallikrein related peptidase 9
1,03	KIAA1524	KIAA1524
1,03	MAB21L1	mab-21-like 1 (C. elegans)
1,03	TEX15	testis expressed 15
1,03	LOC441178; RP11-351J23.1	uncharacterized LOC441178; novel tanscript
1,03	ZNF24	zinc finger protein 24
1,03	ZNF320	zinc finger protein 320
1,03	DIRAS2	DIRAS family, GTP-binding RAS-like 2
1,03	NPIPA8	nuclear pore complex interacting protein family, member A8
1,03	EXT2	exostosin glycosyltransferase 2
1,03	ID3	inhibitor of DNA binding 3, dominant negative helix-loop-helix protein
1,03	NFE2L1	nuclear factor, erythroid 2-like 1
1,03	OR10G8	olfactory receptor, family 10, subfamily G, member 8
1,03	FBXW2	F-box and WD repeat domain containing 2
1,03	MLLT11	myeloid/lymphoid or mixed-lineage leukemia; translocated to, 11
1,03	NSUN5P2; NSUN5P1	NOP2/Sun domain family, member 5 pseudogene 2; NOP2/Sun domain family, member 5 pseudogene 1
1,03	SCIMP	SLP adaptor and CSK interacting membrane protein
1,03	ELF3	E74-like factor 3 (ets domain transcription factor, epithelial-specific)
1,03	SHC4	SHC (Src homology 2 domain containing) family, member 4
1,03	MAGEB3	Transcript Identified by AceView, Entrez Gene ID(s) 4114
1,03	SFI1	SFI1 centrin binding protein
1,03	FGFBP1	fibroblast growth factor binding protein 1
1,03	VPS4B	vacuolar protein sorting 4 homolog B (S. cerevisiae)
1,03	SAR1B	Transcript Identified by AceView, Entrez Gene ID(s) 51128
1,03	C8orf4	chromosome 8 open reading frame 4
1,03	TCP10L2	t-complex 10-like 2
1,03	TM9SF2	transmembrane 9 superfamily member 2
1,03	IFNGR2	interferon gamma receptor 2 (interferon gamma transducer 1)
1,03	FAM179A	family with sequence similarity 179, member A
1,03	PRRG3	proline rich Gla (G-carboxyglutamic acid) 3 (transmembrane)
1,03	ITPKC	Transcript Identified by AceView, Entrez Gene ID(s) 80271
1,03	FERMT1	fermitin family member 1

1,03	SNRNP27	small nuclear ribonucleoprotein, U4/U6.U5 27kDa subunit
1,03	RPS6KA1	ribosomal protein S6 kinase, 90kDa, polypeptide 1
1,03	SAMD5	sterile alpha motif domain containing 5
1,03	AVEN	apoptosis, caspase activation inhibitor
1,03	SURF6	surfeit 6
1,03	FGF21	fibroblast growth factor 21
1,03	PRAMEF19	PRAME family member 19 [Source:EntrezGene;Acc:645414]
1,03	PGAM4	phosphoglycerate mutase family member 4
1,03	DLL3	delta-like 3 (Drosophila)
1,03	HMMR	hyaluronan-mediated motility receptor (RHAMM)
1,03	ADRA2C	adrenoceptor alpha 2C
1,03	METTL16	methyltransferase like 16
1,03	STAT5A	signal transducer and activator of transcription 5A
1,03	MND1	meiotic nuclear divisions 1
1,03	SRFBP1	serum response factor binding protein 1
1,03	NENF	neudesin neurotrophic factor
1,03	PDP2	pyruvate dehyrogenase phosphatase catalytic subunit 2
1,03	RUVBL2	RuvB-like AAA ATPase 2
1,03	PCDHB9	protocadherin beta 9
1,03	ISM1	isthmin 1, angiogenesis inhibitor
1,03	EIF3K	eukaryotic translation initiation factor 3, subunit K
1,03	CFAP161	cilia and flagella associated protein 161
1,03	SLC30A5	solute carrier family 30 (zinc transporter), member 5
1,03	CTNNBL1	catenin, beta like 1
1,03	KCNJ11	potassium channel, inwardly rectifying subfamily J, member 11
1,03	CMPK1	cytidine monophosphate (UMP-CMP) kinase 1, cytosolic
1,03	INSM2	insulinoma-associated 2
1,03	EXTL2	exostosin-like glycosyltransferase 2
1,03	KRTAP4-7	keratin associated protein 4-7
1,03	TRPT1	tRNA phosphotransferase 1
1,03	CRKL	v-crk avian sarcoma virus CT10 oncogene homolog-like
1,03	UBXN1	UBX domain protein 1
1,03	BRIX1	BRX1, biogenesis of ribosomes
1,03	PSMA3	proteasome subunit alpha 3
1,03	ERLIN1	ER lipid raft associated 1
1,03	SASH3	SAM and SH3 domain containing 3
1,03	DLST	dihydrolipoamide S-succinyltransferase (E2 component of 2-oxo-glutarate complex)
1,03	HAS1	hyaluronan synthase 1
1,03	PHACTR2	Jeck2013 ALT_ACCEPTOR, ALT_DONOR, coding, INTERNAL, intronic best transcript NM_001100164
1,03	U2AF2	U2 small nuclear RNA auxiliary factor 2
1,03	PITX3	paired-like homeodomain 3

1,03	MAPK1IP1L	mitogen-activated protein kinase 1 interacting protein 1-like
1,03	EGR2	early growth response 2
1,03	CRLF2	cytokine receptor-like factor 2
1,03	FKBP1A-SDCBP2	FKBP1A-SDCBP2 readthrough (NMD candidate)
1,03	sep-11	septin 11
1,03	SRSF1	serine/arginine-rich splicing factor 1
1,03	ITGA7	integrin alpha 7
1,03	OR5D13	olfactory receptor, family 5, subfamily D, member 13 (gene/pseudogene)
1,03	TMEM178A	transmembrane protein 178A
1,03	ASGR2	asialoglycoprotein receptor 2
1,03	GNAS	GNAS complex locus
1,03	LGALS17A	Charcot-Leyden crystal protein pseudogene
1,03	MMP15	matrix metallopeptidase 15 (membrane-inserted)
1,03	TDRD12	tudor domain containing 12
1,03	SOS2	SOS Ras/Rho guanine nucleotide exchange factor 2
1,03	FAM156A	family with sequence similarity 156, member A
1,03	RPL19	ribosomal protein L19
1,03	RXRB	retinoid X receptor beta
1,03	ATP5J	ATP synthase, H+ transporting, mitochondrial Fo complex subunit F6
1,03	ERAL1	Era-like 12S mitochondrial rRNA chaperone 1
1,03	C10orf99	chromosome 10 open reading frame 99
1,03	SPIN3	spindlin family, member 3
1,03	NLRC4	NLR family, CARD domain containing 4
1,03	INPP5D	inositol polyphosphate-5-phosphatase D
1,03	OR8B3	olfactory receptor, family 8, subfamily B, member 3
1,03	RPL26	ribosomal protein L26
1,03	UBE2L3	ubiquitin conjugating enzyme E2L 3
1,03	SIM2	single-minded family bHLH transcription factor 2
1,03	ASUN	asunder spermatogenesis regulator
1,03	RP11-362J17.1; PACRGL	novel transcript, antisense to KCNIP4; Transcript Identified by AceView, Entrez Gene ID(s) 133015
1,03	LZTS3; ProSAPiP1	leucine zipper, putative tumor suppressor family member 3; Leucine zipper putative tumor suppressor 3 [Source:UniProtKB/Swiss-Prot;Acc:O60299]; Salzman2013 ANNOTATED, CDS, coding, OVCODE, OVEXON, UTR5 best transcript NM_014731
1,03	GPNMB	glycoprotein (transmembrane) nmb
1,03	ZNF69	zinc finger protein 69
1,03	MRE11A	MRE11 homolog A, double strand break repair nuclease
1,03	HERPUD2	HERPUD family member 2
1,03	CD40	CD40 molecule, TNF receptor superfamily member 5
1,03	SLC16A7	solute carrier family 16 (monocarboxylate transporter), member 7
1,03	TSGA10	testis specific 10

1,03	SAG	S-antigen; retina and pineal gland (arrestin)
1,03	ZC3H15	zinc finger CCCH-type containing 15
1,02	ALG10B	ALG10B, alpha-1,2-glucosyltransferase
1,02	DBF4	DBF4 zinc finger
1,02	ZFP14	ZFP14 zinc finger protein
1,02	PANX1	pannexin 1
1,02	NEURL2	neuralized E3 ubiquitin protein ligase 2
1,02	RPL6	ribosomal protein L6
1,02	TSGA10IP	testis specific 10 interacting protein
1,02	GOLGA8G	golgin A8 family, member G
1,02	L3MBTL1	l(3)mbt-like 1 (Drosophila)
1,02	KRI1	KRI1 homolog
1,02	PARK2	parkin RBR E3 ubiquitin protein ligase
1,02	CNGA2	cyclic nucleotide gated channel alpha 2
1,02	PDHA1	pyruvate dehydrogenase (lipoamide) alpha 1
1,02	NT5C1B; NT5C1B-RDH14; RDH14	5-nucleotidase, cytosolic IB; NT5C1B-RDH14 readthrough; retinol dehydrogenase 14 (all-trans/9-cis/11-cis)
1,02	RPP38	ribonuclease P/MRP 38kDa subunit
1,02	TOMM20L	translocase of outer mitochondrial membrane 20 homolog (yeast)-like
1,02	SDCBP	syndecan binding protein
1,02	PRSS33	protease, serine, 33
1,02	HIST2H2AA4; HIST2H2AA3	histone cluster 2, H2aa4; histone cluster 2, H2aa3
1,02	C20orf78	chromosome 20 open reading frame 78
1,02	CAPG	capping protein (actin filament), gelsolin-like
1,02	PIAS2	protein inhibitor of activated STAT 2
1,02	ZNF502	zinc finger protein 502
1,02	CARTPT	CART prepropeptide
1,02	PRG3	proteoglycan 3
1,02	LOC388242; LOC613038; RP11-345J4.3	SAGA complex associated factor 29 pseudogene; Transcript Identified by AceView, Entrez Gene ID(s) 388242; Uncharacterized protein [Source:UniProtKB/TrEMBL;Acc:H0YHM3]
1,02	ITGB7	integrin beta 7
1,02	RHOU	ras homolog family member U
1,02	TRIM2	tripartite motif containing 2
1,02	XKR4	X-linked Kx blood group related 4
1,02	RNF122	ring finger protein 122
1,02	WNK1	Transcript Identified by AceView, Entrez Gene ID(s) 378465; 65125
1,02	KAT7	K(lysine) acetyltransferase 7
1,02	ZNF814	zinc finger protein 814
1,02	KSR2	kinase suppressor of ras 2
1,02	POMP	proteasome maturation protein
1,02	AIRE	autoimmune regulator
1,02	ZNF418	zinc finger protein 418
1,02	COMMD1	copper metabolism (Murr1) domain containing 1
1,02	FAXDC2	fatty acid hydroxylase domain containing 2

1,02	LOC339862; RP11-320G10.1; skerdorbo	uncharacterized LOC339862; novel transcript; Transcript Identified by AceView
1,02	NDUFC2-KCTD14; NDUFC2; KCTD14	NDUFC2-KCTD14 readthrough; NADH dehydrogenase (ubiquinone) 1, subcomplex unknown, 2, 14.5kDa; potassium channel tetramerization domain containing 14
1,02	RIOK1	RIO kinase 1
1,02	SURF6	surfeit 6
1,02	CEP55	centrosomal protein 55kDa
1,02	ZNF80	zinc finger protein 80
1,02	PAPOLA	poly(A) polymerase alpha
1,02	SLC44A1	solute carrier family 44 (choline transporter), member 1
1,02	COLGALT2	collagen beta(1-O)galactosyltransferase 2
1,02	TXN	thioredoxin
1,02	RBFA; RBFADN	ribosome binding factor A (putative); RBFA downstream neighbor (non-protein coding)
1,02	PACSIN2	protein kinase C and casein kinase substrate in neurons 2
1,02	HOGA1	4-hydroxy-2-oxoglutarate aldolase 1
1,02	ZNF784	zinc finger protein 784
1,02	RPAIN	RPA interacting protein
1,02	SAE1	SUMO1 activating enzyme subunit 1
1,02	PRR14	proline rich 14
1,02	ZDHHC14	zinc finger, DHHC-type containing 14
1,02	PPP1R8	protein phosphatase 1, regulatory subunit 8
1,02	ZFYVE28	zinc finger, FYVE domain containing 28
1,02	ASCC2	activating signal cointegrator 1 complex subunit 2
1,02	NDEL1	nudE neurodevelopment protein 1-like 1
1,02	OR5AK2	olfactory receptor, family 5, subfamily AK, member 2
1,02	MT3	metallothionein 3
1,02	CHST9	carbohydrate (N-acetylgalactosamine 4-0) sulfotransferase 9
1,02	UPF3A	UPF3 regulator of nonsense transcripts homolog A (yeast)
1,02	MANEAL	mannosidase, endo-alpha-like
1,02	PDZD7	PDZ domain containing 7
1,02	FYN	FYN proto-oncogene, Src family tyrosine kinase
1,02	CSAG3	CSAG family, member 3
1,02	ASCL4	achaete-scute family bHLH transcription factor 4
1,02	RPS27	ribosomal protein S27
1,02	MDH1B	malate dehydrogenase 1B
1,02	ARHGAP33	Rho GTPase activating protein 33
1,02	ARV1	ARV1 homolog, fatty acid homeostasis modulator
1,02	SLX1A-SULT1A3	SLX1A-SULT1A3 readthrough (NMD candidate)
1,02	DENR	density-regulated protein
1,02	GMDS	GDP-mannose 4,6-dehydratase
1,02	CEP95	centrosomal protein 95kDa

1,02	MYD88	myeloid differentiation primary response 88
1,02	BTG3	BTG family, member 3
1,02	C16orf47	chromosome 16 open reading frame 47
1,02	TMX3	thioredoxin-related transmembrane protein 3
1,02	CHURC1	churchill domain containing 1
1,02	GTPBP8	GTP-binding protein 8 (putative)
1,02	DAP	death-associated protein
1,02	ABCF2	ATP binding cassette subfamily F member 2
1,02	AGPAT3	1-acylglycerol-3-phosphate O-acyltransferase 3
1,02	SLC17A9	solute carrier family 17 (vesicular nucleotide transporter), member 9
1,02	OR5W2	olfactory receptor, family 5, subfamily W, member 2
1,02	GDPD5	glycerophosphodiester phosphodiesterase domain containing 5
1,02	AES	amino-terminal enhancer of split
1,02	PDHB	pyruvate dehydrogenase (lipoamide) beta
1,02	NCBP3	nuclear cap binding subunit 3
1,02	MME	membrane metallo-endopeptidase
1,02	BPY2C; BPY2	basic charge, Y-linked, 2C; basic charge, Y-linked, 2
1,02	PRRT1	proline-rich transmembrane protein 1
1,02	FAM159A	family with sequence similarity 159, member A
1,02	ETV3L	ets variant 3-like
1,02	HMGCR	3-hydroxy-3-methylglutaryl-CoA reductase
1,02	DCTPP1	dCTP pyrophosphatase 1
1,02	POLR3C	polymerase (RNA) III (DNA directed) polypeptide C (62kD)
1,02	VPS28	vacuolar protein sorting 28 homolog (S. cerevisiae)
1,02	ACD	adrenocortical dysplasia homolog (mouse)
1,02	CSHL1	chorionic somatomammotropin hormone-like 1
1,02	SFSWAP	splicing factor, suppressor of white-apricot family
1,02	PARP12	poly(ADP-ribose) polymerase family member 12
1,02	CD79A	CD79a molecule, immunoglobulin-associated alpha
1,02	RRM2B	ribonucleotide reductase M2 B (TP53 inducible)
1,02	RPL17; SNORD58A; SNORD58B; SNORD58C	ribosomal protein L17; small nucleolar RNA, C/D box 58A; small nucleolar RNA, C/D box 58B; small nucleolar RNA, C/D box 58C
1,02	BCL2L1	BCL2-like 1
1,02	HMHB1	histocompatibility (minor) HB-1
1,02	AMICA1	adhesion molecule, interacts with CXADR antigen 1
1,02	GLMN	glomulin, FKBP associated protein
1,02	AC009133.12; PAGR1	novel transcript, antisense to C16orf53 and MVP; PAXIP1 associated glutamate-rich protein 1 [Source:HGNC Symbol;Acc:HGNC:28707]
1,02	TBX18	T-box 18
1,02	TYROBP	TYRO protein tyrosine kinase binding protein
1,02	OCSTAMP	osteoclast stimulatory transmembrane protein
1,02	PRX	periaxin
1,02	EEF1A1P5	eukaryotic translation elongation factor 1 alpha 1 pseudogene 5

1,02	CD209	CD209 molecule
1,02	LAMTOR3	late endosomal/lysosomal adaptor, MAPK and MTOR activator 3
1,02	SRSF2; MIR636	serine/arginine-rich splicing factor 2; microRNA 636
1,02	C11orf45	chromosome 11 open reading frame 45
1,02	CCDC102A	coiled-coil domain containing 102A
1,02	GLIS2	GLIS family zinc finger 2
1,02	RFPL4AL1	ret finger protein-like 4A-like 1
1,02	CDIP1	cell death-inducing p53 target 1
1,02	OSBPL1A	oxysterol binding protein-like 1A
1,02	BCL2L14	BCL2-like 14 (apoptosis facilitator)
1,02	ISM2	isthmin 2
1,02	KRT78	keratin 78, type II
1,02	HLA-DPA1	major histocompatibility complex, class II, DP alpha 1
1,02	MGAT1	mannosyl (alpha-1,3-)-glycoprotein beta-1,2-N-acetylglucosaminyltransferase
1,02	TMSB4X	thymosin beta 4, X-linked
1,02	PRDM10	PR domain containing 10
1,02	CLLU1	chronic lymphocytic leukemia up-regulated 1
1,02	FAM192A	family with sequence similarity 192, member A
1,02	FTL	ferritin, light polypeptide
1,02	CALML4	calmodulin-like 4
1,02	B4GALT4	UDP-Gal:betaGlcNAc beta 1,4- galactosyltransferase, polypeptide 4
1,02	IKBKE	inhibitor of kappa light polypeptide gene enhancer in B-cells, kinase epsilon
1,02	ERCC1	excision repair cross-complementation group 1
1,02	ABCB1	ATP binding cassette subfamily B member 1
1,02	HMBS	hydroxymethylbilane synthase
1,02	SLC13A3; TP53RK	solute carrier family 13 (sodium-dependent dicarboxylate transporter), member 3; TP53 regulating kinase
1,02	TGM3	transglutaminase 3
1,02	FYB	FYN binding protein
1,02	CGB5; CGB8	chorionic gonadotropin, beta polypeptide 5; chorionic gonadotropin, beta polypeptide 8
1,02	FAM127A	family with sequence similarity 127, member A
1,02	CCDC140	coiled-coil domain containing 140
1,02	MTFMT	mitochondrial methionyl-tRNA formyltransferase
1,02	CLCN2	chloride channel, voltage-sensitive 2
1,02	GALNT6	polypeptide N-acetylgalactosaminyltransferase 6
1,02	WDR36	WD repeat domain 36
1,02	CAPZB	capping protein (actin filament) muscle Z-line, beta
1,02	UBE2R2	ubiquitin-conjugating enzyme E2R 2
1,02	GCLM	glutamate-cysteine ligase, modifier subunit
1,02	ACY1	aminoacylase 1
1,02	TAF2	TAF2 RNA polymerase II, TATA box binding protein (TBP)-associated factor, 150kDa
1,02	OXGR1	oxoglutarate (alpha-ketoglutarate) receptor 1

1,02	ZCWPW1	zinc finger, CW type with PWWP domain 1
1,02	CCDC13	coiled-coil domain containing 13
1,02	VPS11	vacuolar protein sorting 11 homolog (S. cerevisiae)
1,02	MED24; MIR6884	mediator complex subunit 24; microRNA 6884
1,02	HIST2H2AA3; HIST2H2AA4	histone cluster 2, H2aa3; histone cluster 2, H2aa4
1,02	OR8D2	olfactory receptor, family 8, subfamily D, member 2 (gene/pseudogene)
1,02	MGLL	monoglyceride lipase
1,02	ZNF277	zinc finger protein 277
1,02	CEACAM16	carcinoembryonic antigen-related cell adhesion molecule 16
1,02	HIST1H2BE	histone cluster 1, H2be
1,02	HAUS2	HAUS augmin like complex subunit 2
1,02	HYOU1	hypoxia up-regulated 1
1,02	TSEN15	TSEN15 tRNA splicing endonuclease subunit
1,02	NR4A2	nuclear receptor subfamily 4, group A, member 2
1,02	EIF2AK1	eukaryotic translation initiation factor 2-alpha kinase 1
1,02	UBE2G1	ubiquitin conjugating enzyme E2G 1
1,02	OLR1	oxidized low density lipoprotein (lectin-like) receptor 1
1,02	FADS3	fatty acid desaturase 3
1,02	PLEKHB2	pleckstrin homology domain containing, family B (evectins) member 2
1,02	C5orf15	chromosome 5 open reading frame 15
1,02	EBAG9	estrogen receptor binding site associated, antigen, 9
1,02	PJA2	praja ring finger 2, E3 ubiquitin protein ligase
1,02	USP1	ubiquitin specific peptidase 1
1,02	EIF3F	Eukaryotic translation initiation factor 3 subunit F [Source:UniProtKB/Swiss-Prot;Acc:O00303]
1,02	DSCR8	Down syndrome critical region 8
1,02	PPIA	peptidylprolyl isomerase A (cyclophilin A)
1,02	KIF20A	kinesin family member 20A
1,02	HIST1H2BC	histone cluster 1, H2bc
1,02	OASL	2-5-oligoadenylate synthetase-like
1,02	LHFPL4	lipoma HMGIC fusion partner-like 4
1,02	SRP9	signal recognition particle 9kDa
1,02	TDO2	tryptophan 2,3-dioxygenase
1,02	CAPN5	calpain 5
1,02	ZBTB32	zinc finger and BTB domain containing 32
1,02	UPK1B	uroplakin 1B
1,02	OTUD4	OTU deubiquitinase 4
1,02	C7orf57	chromosome 7 open reading frame 57
1,02	ATP1A3	ATPase, Na+/K+ transporting, alpha 3 polypeptide
1,02	SLC35C1	solute carrier family 35 (GDP-fucose transporter), member C1
1,02	DDX11	DEAD/H (Asp-Glu-Ala-Asp/His) box helicase 11
1,02	P4HA1	prolyl 4-hydroxylase, alpha polypeptide I

1,02	CUTA	cutA divalent cation tolerance homolog (E. coli)
1,02	RNASE9	ribonuclease, RNase A family, 9 (non-active)
1,02	NUDT4P1; NUDT4P2; NUDT4	nudix hydrolase 4 pseudogene 1; nudix hydrolase 4 pseudogene 2; nudix hydrolase 4
1,02	NUDT4; NUDT4P1	nudix hydrolase 4; nudix hydrolase 4 pseudogene 1
1,02	EMC10	ER membrane protein complex subunit 10
1,02	DKC1; SNORA36A; SNORA56; MIR664B	dyskeratosis congenita 1, dyskerin; small nucleolar RNA, H/ACA box 36A; small nucleolar RNA, H/ACA box 56; microRNA 664b
1,02	UBAC1	UBA domain containing 1
1,02	ELK4	ELK4, ETS-domain protein (SRF accessory protein 1)
1,02	TRAM2	translocation associated membrane protein 2
1,02	ZNF667	zinc finger protein 667
1,02	TWSG1	twisted gastrulation BMP signaling modulator 1
1,02	PDIA2	protein disulfide isomerase family A, member 2
1,02	KRTAP10-11; KRTAP12-3; KRTAP10-12	keratin associated protein 10-11; keratin associated protein 12-3; keratin associated protein 10-12
1,02	ADH5	alcohol dehydrogenase 5 (class III), chi polypeptide
1,02	HSPA5	heat shock 70kDa protein 5 (glucose-regulated protein, 78kDa)
1,02	CSNK1G3	casein kinase 1, gamma 3
1,02	CASKIN2	CASK interacting protein 2
1,02	LOC101927572; AC002116.7; CLIP3	uncharacterized LOC101927572; Memczak2013 ANTISENSE, CDS, coding, INTERNAL, intronic best transcript NM_015526; novel transcript, antisense to CLIP3 and THAP8
1,02	PSME3	proteasome activator subunit 3
1,02	INTS12	integrator complex subunit 12
1,02	C11orf44	chromosome 11 open reading frame 44
1,02	VPS36	vacuolar protein sorting 36 homolog (S. cerevisiae)
1,02	CERS5	ceramide synthase 5
1,02	HRG	histidine-rich glycoprotein
1,02	SYNRG	synergin, gamma
1,02	PCBD2	pterin-4 alpha-carbinolamine dehydratase/dimerization cofactor of hepatocyte nuclear factor 1 alpha (TCF1) 2
1,02	SFXN3	sideroflexin 3
1,02	HRC	histidine rich calcium binding protein
1,02	OR5P3	olfactory receptor, family 5, subfamily P, member 3
1,02	XPO6	exportin 6
1,02	N6AMT2	N-6 adenine-specific DNA methyltransferase 2 (putative)
1,02	VIM	vimentin
1,02	PLCB3	phospholipase C, beta 3 (phosphatidylinositol-specific)
1,02	PCDH20	protocadherin 20
1,02	RASL12	RAS-like, family 12

1,02	PWWP2B	PWWP domain containing 2B
1,02	GLTSCR1	glioma tumor suppressor candidate region gene 1
1,02	TMEM249	transmembrane protein 249
1,02	SLCO4A1-AS1	SLCO4A1 antisense RNA 1
1,02	RFX4	regulatory factor X, 4 (influences HLA class II expression)
1,02	BTBD11	BTB (POZ) domain containing 11
1,02	MYPN	myopalladin
1,02	PIGS	phosphatidylinositol glycan anchor biosynthesis class S
1,02	PGLS	6-phosphogluconolactonase
1,02	UBASH3A	ubiquitin associated and SH3 domain containing A
1,02	C9	complement component 9
1,02	RGS14	regulator of G-protein signaling 14
1,02	ATP11C	ATPase, class VI, type 11C
1,02	DDX55	DEAD (Asp-Glu-Ala-Asp) box polypeptide 55
1,02	SDK2	sidekick cell adhesion molecule 2
1,02	CHST11	carbohydrate (chondroitin 4) sulfotransferase 11
1,02	POTEB3; POTEB; AC136352.4	Homo sapiens POTE ankyrin domain family, member B3 (POTEB3), transcript variant 1, mRNA.; Homo sapiens POTE ankyrin domain family, member B, mRNA (cDNA clone MGC:119371 IMAGE:40006483), complete cds.; Homo sapiens POTE ankyrin domain family, member B, mRNA (cDNA clone MGC:119372 IMAGE:40006485), complete cds.; Homo sapiens POTE ankyrin domain family, member B, mRNA (cDNA clone MGC:119373 IMAGE:40006489), complete cds.; Homo sapiens POTE ankyrin domain family, member B, mRNA (cDNA clone MGC:156116 IMAGE:40006490), complete cds.
1,02	CDH23	cadherin-related 23
1,02	TNFSF18	tumor necrosis factor (ligand) superfamily, member 18
1,02	SCGB1C1	secretoglobin, family 1C, member 1
1,02	ILF3	interleukin enhancer binding factor 3
1,02	WDR46; MIR6873	WD repeat domain 46; microRNA 6873
1,02	MAVS	Jeck2013 ANTISENSE, coding, INTERNAL, OVEXON, UTR3 best transcript NM_020746
1,02	LRAT	lecithin retinol acyltransferase (phosphatidylcholine--retinol O-acyltransferase)
1,02	ACTL6A	actin-like 6A
1,02	MYL7	myosin light chain 7
1,02	XRCC1	X-ray repair complementing defective repair in Chinese hamster cells 1
1,02	TMEM125	transmembrane protein 125
1,02	CEP250	centrosomal protein 250kDa
1,02	SKAP1	src kinase associated phosphoprotein 1

1,02	TBC1D3H; TBC1D3L; TBC1D3I; LOC101060389; TBC1D3C; TBC1D3; TBC1D3D	Homo sapiens TBC1 domain family, member 3H (TBC1D3H), mRNA.; Homo sapiens TBC1 domain family, member 3L (TBC1D3L), mRNA.; Homo sapiens TBC1 domain family, member 3I (TBC1D3I), mRNA.; Homo sapiens TBC1 domain family member-like (LOC101060389), mRNA.; TBC1 domain family member-like; Homo sapiens TBC1 domain family, member 3C, mRNA (cDNA clone MGC:87891 IMAGE:5165385), complete cds.; Homo sapiens TBC1 domain family, member 3, mRNA (cDNA clone MGC:87893 IMAGE:5742850), complete cds.; TBC1 domain family, member 3D [Source:HGNC Symbol;Acc:HGNC:28944]
1,02	RBM12B	RNA binding motif protein 12B
1,02	DYRK1B	dual specificity tyrosine-(Y)-phosphorylation regulated kinase 1B
1,02	SMARCA2	SWI/SNF related, matrix associated, actin dependent regulator of chromatin, subfamily a, member 2
1,02	CDIPT	CDP-diacylglycerol--inositol 3-phosphatidyltransferase
1,02	USP30	ubiquitin specific peptidase 30
1,02	MLH1	mutL homolog 1
1,02	CELA2A	chymotrypsin-like elastase family, member 2A
1,02	UQCRC2	ubiquinol-cytochrome c reductase core protein II
1,02	FAM111B	family with sequence similarity 111, member B
1,02	RPS6KA6	ribosomal protein S6 kinase, 90kDa, polypeptide 6
1,02	SLC15A1	solute carrier family 15 (oligopeptide transporter), member 1
1,02	ARF1; MIR3620	ADP-ribosylation factor 1; microRNA 3620
1,02	MKS1	Meckel syndrome, type 1
1,02	MSN	moesin
1,02	SLC22A17	solute carrier family 22, member 17
1,02	YWHAG	Memczak2013 ALT_ACCEPTOR, ALT_DONOR, coding, INTERNAL, intronic best transcript NM_012479
1,02	PLCB1	phospholipase C, beta 1 (phosphoinositide-specific)
1,02	SLC35G4	solute carrier family 35, member G4
1,02	HYOU1	hypoxia up-regulated 1
1,02	HNRNPD	heterogeneous nuclear ribonucleoprotein D
1,02	NT5DC1	5-nucleotidase domain containing 1
1,02	AZIN1	antizyme inhibitor 1
1,02	DMGDH	dimethylglycine dehydrogenase
1,02	C1QL4	complement component 1, q subcomponent-like 4
1,02	GPR1	G protein-coupled receptor 1
1,02	TBC1D3E	TBC1 domain family, member 3E
1,02	TMEM246	transmembrane protein 246
1,02	AQP8	aquaporin 8
1,02	ST3GAL6	ST3 beta-galactoside alpha-2,3-sialyltransferase 6
1,02	PDGFC	platelet derived growth factor C
1,02	LOC400558; RP11-46C24.3	uncharacterized LOC400558; Transcript Identified by AceView, Entrez Gene ID(s) 400558; novel transcript

1,02	RAI14	retinoic acid induced 14
1,02	PRH1	proline-rich protein HaeIII subfamily 1
1,02	IPO11; LRRC70	importin 11; leucine rich repeat containing 70
1,02	C7	complement component 7
1,02	ACO2	aconitase 2, mitochondrial
1,02	SDHD	succinate dehydrogenase complex subunit D, integral membrane protein
1,02	TCF21	transcription factor 21
1,02	DDA1	DET1 and DDB1 associated 1
1,02	PSMD9	proteasome 26S subunit, non-ATPase 9
1,02	MPP3	membrane protein, palmitoylated 3
1,02	RNGTT	RNA guanylyltransferase and 5-phosphatase
1,02	DUS3L	dihydrouridine synthase 3-like
1,02	FASTKD2	FAST kinase domains 2
1,02	PNPLA7	patatin-like phospholipase domain containing 7
1,02	ZNF286A	zinc finger protein 286A
1,02	ERAP1	Transcript Identified by AceView, Entrez Gene ID(s) 51752
1,02	PGRMC1	progesterone receptor membrane component 1
1,02	ARMCX5	armadillo repeat containing, X-linked 5
1,02	SSSCA1-AS1	SSSCA1 antisense RNA 1 (head to head)
1,02	MYL12A	myosin light chain 12A
1,02	WNT9B	wingless-type MMTV integration site family, member 9B
1,02	SZT2	seizure threshold 2 homolog (mouse)
1,02	HIST1H2AB	histone cluster 1, H2ab
1,02	FAM101B	family with sequence similarity 101, member B
1,02	ADARB2	adenosine deaminase, RNA-specific, B2 (inactive)
1,02	BLOC1S6	biogenesis of lysosomal organelles complex-1, subunit 6, pallidin
1,02	GCC2	GRIP and coiled-coil domain containing 2
1,02	CYTL1	cytokine like 1
1,02	ZNF44	zinc finger protein 44
1,02	FHL2	four and a half LIM domains 2
1,02	RBMX; SNORD61	RNA binding motif protein, X-linked; small nucleolar RNA, C/D box 61
1,02	ENO4	enolase family member 4
1,02	HNRNPCL4	heterogeneous nuclear ribonucleoprotein C-like 4 [Source:HGNC Symbol;Acc:HGNC:51333]
1,02	NKX1-2	NK1 homeobox 2
1,02	LRTOMT	leucine rich transmembrane and O-methyltransferase domain containing
1,02	LHFP	lipoma HMGIC fusion partner
1,02	VDAC1	voltage-dependent anion channel 1
1,02	HSD17B12	hydroxysteroid (17-beta) dehydrogenase 12
1,02	TONSL	tonsoku-like, DNA repair protein
1,02	SPATA16	spermatogenesis associated 16
1,02	GAS1	growth arrest-specific 1
1,02	NCBP2L	nuclear cap binding protein subunit 2-like
1,02	NSDHL	NAD(P) dependent steroid dehydrogenase-like

1,02	APOL3	apolipoprotein L, 3
1,02	COX7C; MIR3607	cytochrome c oxidase subunit VIIc; microRNA 3607
1,02	IDH1	isocitrate dehydrogenase 1 (NADP+)
1,02	NFAM1	NFAT activating protein with ITAM motif 1
1,02	CEP57L1	centrosomal protein 57kDa-like 1
1,02	IFT80	intraflagellar transport 80
1,02	OR6K2	olfactory receptor, family 6, subfamily K, member 2
1,02	MTUS2	microtubule associated tumor suppressor candidate 2
1,02	FTO	fat mass and obesity associated
1,02	NGEF	neuronal guanine nucleotide exchange factor
1,02	DCTN1	dynactin 1
1,02	DEFB131	defensin, beta 131
1,02	SALL4	spalt-like transcription factor 4
1,02	URI1	URI1, prefoldin-like chaperone
1,02	GTF2H1	general transcription factor IIH subunit 1
1,02	CRTAP	cartilage associated protein
1,02	C14orf105	chromosome 14 open reading frame 105
1,02	KIF5C	kinesin family member 5C
1,02	DRAP1	DR1-associated protein 1 (negative cofactor 2 alpha)
1,02	EMC8	ER membrane protein complex subunit 8
1,02	AAGAB	Transcript Identified by AceView, Entrez Gene ID(s) 79719
1,02	GON4L	gon-4-like (C. elegans)
1,02	ITPA	inosine triphosphatase (nucleoside triphosphate pyrophosphatase)
1,02	GRAMD1B	GRAM domain containing 1B
1,02	ABCC9	Transcript Identified by AceView, Entrez Gene ID(s) 10060
1,02	UBXN8	UBX domain protein 8
1,02	CD101	CD101 molecule
1,02	KIAA0368	KIAA0368
1,02	TAC1	tachykinin, precursor 1
1,02	PLCZ1	phospholipase C, zeta 1
1,02	USP34	Transcript Identified by AceView, Entrez Gene ID(s) 9736
1,02	MFAP3L	microfibrillar associated protein 3 like
1,02	KCNG1	potassium channel, voltage gated modifier subfamily G, member 1
1,02	CBX1	chromobox homolog 1
1,02	PPRC1	peroxisome proliferator-activated receptor gamma, coactivator-related 1
1,02	SYS1	Sys1 golgi trafficking protein
1,02	PDAP1	PDGFA associated protein 1
1,02	PLP2	proteolipid protein 2 (colonic epithelium-enriched)
1,02	KRTAP4-8	keratin associated protein 4-8
1,02	CDH3	cadherin 3, type 1, P-cadherin (placental)
1,02	AP5M1	adaptor-related protein complex 5, mu 1 subunit
1,02	NSUN2	NOP2/Sun RNA methyltransferase family, member 2
1,02	SH3D21	SH3 domain containing 21

1,02	LRRC61	leucine rich repeat containing 61
1,02	YTHDF2	YTH N(6)-methyladenosine RNA binding protein 2
1,02	OR5P2	olfactory receptor, family 5, subfamily P, member 2
1,02	MYO1G	Memczak2013 ANTISENSE, CDS, coding, INTERNAL best transcript NM_033054
1,02	EIF4H	eukaryotic translation initiation factor 4H
1,02	C1QBP	complement component 1, q subcomponent binding protein
1,02	LRIG1	
1,02	NOX1	NADPH oxidase 1
1,02	PEX2	peroxisomal biogenesis factor 2
1,02	IL11	interleukin 11
1,02	SOAT1	sterol O-acyltransferase 1
1,02	GOLGA8N; GOLGA8H	golgin A8 family, member N; golgin A8 family, member H
1,02	ANGEL2	angel homolog 2 (Drosophila)
1,02	MED29	mediator complex subunit 29
1,02	S1PR1	sphingosine-1-phosphate receptor 1
1,02	CALCRL	calcitonin receptor like receptor
1,02	IQSEC1	IQ motif and Sec7 domain 1
1,02	CXCL3	chemokine (C-X-C motif) ligand 3
1,02	PROKR2	prokineticin receptor 2
1,02	CMTM5	CKLF-like MARVEL transmembrane domain containing 5
1,02	CCDC59	coiled-coil domain containing 59
1,02	TACR1	tachykinin receptor 1
1,02	SIGIRR	single immunoglobulin and toll-interleukin 1 receptor (TIR) domain
1,02	MRPL34	mitochondrial ribosomal protein L34
1,02	ATP5SL	ATP5S-like
1,02	IFFO1	intermediate filament family orphan 1
1,02	FAM65C	family with sequence similarity 65, member C
1,02	HYOU1	hypoxia up-regulated 1
1,02	ZNF230	zinc finger protein 230
1,02	PPIB	Jeck2013 ALT_ACCEPTOR, ALT_DONOR, coding, INTERNAL, intronic, OVERLAPTX, OVEXON best transcript NM_000942
1,02	TUBA4B	tubulin, alpha 4b
1,02	FBXL2	F-box and leucine-rich repeat protein 2
1,02	FAM118B	family with sequence similarity 118, member B
1,02	ANKRD66	ankyrin repeat domain 66
1,02	SLC6A9	solute carrier family 6 (neurotransmitter transporter, glycine), member 9
1,02	NADSYN1	NAD synthetase 1
1,02	KDM6A	lysine (K)-specific demethylase 6A
1,02	AC208162.1	
1,02	LANCL1	LanC lantibiotic synthetase component C-like 1 (bacterial)
1,02	EIF3A	eukaryotic translation initiation factor 3, subunit A
1,02	SUSD3	sushi domain containing 3

1,02	NONO	non-POU domain containing, octamer-binding
1,02	MARS; MIR6758	methionyl-tRNA synthetase; microRNA 6758
1,02	KRT6A	keratin 6A, type II
1,02	FSCB	fibrous sheath CABYR binding protein
1,02	LURAP1L	leucine rich adaptor protein 1-like
1,02	FIBP	fibroblast growth factor (acidic) intracellular binding protein
1,02	RAVER1	ribonucleoprotein, PTB-binding 1
1,02	C20orf173	chromosome 20 open reading frame 173
1,02	ASB8	ankyrin repeat and SOCS box containing 8
1,02	SMIM21	small integral membrane protein 21
1,02	NMUR2	neuromedin U receptor 2
1,02	CCAR1; SNORD98	cell division cycle and apoptosis regulator 1; small nucleolar RNA, C/D box 98
1,02	SMCO4	single-pass membrane protein with coiled-coil domains 4
1,02	DPH1; OVCA2	diphthamide biosynthesis 1; ovarian tumor suppressor candidate 2
1,02	SLFN14	schlafen family member 14
1,02	PCK2	phosphoenolpyruvate carboxykinase 2 (mitochondrial)
1,02	OR56B4	olfactory receptor, family 56, subfamily B, member 4
1,02	MRPL46	mitochondrial ribosomal protein L46
1,02	HMBS	hydroxymethylbilane synthase
1,02	EIF4A2; SNORA63; SNORD2; SNORA4; SNORA81; MIR1248	eukaryotic translation initiation factor 4A2; small nucleolar RNA, H/ACA box 63; small nucleolar RNA, C/D box 2; small nucleolar RNA, H/ACA box 4; small nucleolar RNA, H/ACA box 81; microRNA 1248
1,02	WDR43; SNORD53	WD repeat domain 43; small nucleolar RNA, C/D box 53
1,02	PPP1R1C	protein phosphatase 1, regulatory (inhibitor) subunit 1C
1,02	CFAP74	cilia and flagella associated protein 74
1,02	CBFB	core-binding factor, beta subunit
1,02	AQP12A	aquaporin 12A
1,02	OR6Y1	olfactory receptor, family 6, subfamily Y, member 1
1,02	RSL1D1	ribosomal L1 domain containing 1
1,02	PABPN1L	poly(A) binding protein, nuclear 1-like (cytoplasmic)
1,02	RAB1B	RAB1B, member RAS oncogene family
1,02	AP3M2	adaptor-related protein complex 3, mu 2 subunit
1,02	ZNF618	Zhang2013 ALT_ACCEPTOR, ALT_DONOR, coding, INTERNAL, intronic best transcript NM_133374
1,02	AFG3L2	AFG3-like AAA ATPase 2
1,02	FAM107B	Memczak2013 ALT_ACCEPTOR, ALT_DONOR, coding, INTERNAL, intronic best transcript NM_031453
1,02	ATP6V1F	ATPase, H+ transporting, lysosomal 14kDa, V1 subunit F

1,02	SPANXN5	SPANX family, member N5
1,02	TMCO3	transmembrane and coiled-coil domains 3
1,02	NUDT17	nudix hydrolase 17
1,02	ATF3	activating transcription factor 3
1,02	GJA3	gap junction protein alpha 3
1,02	CDC20	cell division cycle 20
1,02	NAA10	N(alpha)-acetyltransferase 10, NatA catalytic subunit
1,02	ADSS	adenylosuccinate synthase
1,02	PSMC6	proteasome 26S subunit, ATPase 6
1,02	SLC25A43	solute carrier family 25, member 43
1,02	DYNC1I2	dynein, cytoplasmic 1, intermediate chain 2
1,02	ZNF451	zinc finger protein 451
1,02	KIR2DL4	killer cell immunoglobulin-like receptor, two domains, long cytoplasmic tail, 4
1,02	MRPL51	mitochondrial ribosomal protein L51
1,02	VSTM2L	V-set and transmembrane domain containing 2 like
1,02	PHLDB3	pleckstrin homology-like domain, family B, member 3
1,02	FKBP11; ARF3	FK506 binding protein 11; ADP-ribosylation factor 3
1,02	KIAA1324L	KIAA1324-like
1,02	POLR2J4	polymerase (RNA) II (DNA directed) polypeptide J4, pseudogene
1,02	THEG5; AC011525.4; pleysey	testis highly expressed protein 5; novel transcript; Transcript Identified by AceView
1,02	ZNF174	zinc finger protein 174
1,02	NOP56; SNORD57; SNORD56; SNORA51; SNORD110; SNORD86; MIR1292	NOP56 ribonucleoprotein; small nucleolar RNA, C/D box 57; small nucleolar RNA, C/D box 56; small nucleolar RNA, H/ACA box 51; small nucleolar RNA, C/D box 110; small nucleolar RNA, C/D box 86; microRNA 1292
1,02	HMGCLL1	3-hydroxymethyl-3-methylglutaryl-CoA lyase-like 1
1,02	EID3	EP300 interacting inhibitor of differentiation 3
1,02	CEL	carboxyl ester lipase
1,02	B3GALT6	UDP-Gal:betaGal beta 1,3-galactosyltransferase 6
1,02	SULF2	sulfatase 2
1,02	LINC00482	long intergenic non-protein coding RNA 482
1,02	THOC3	THO complex 3
1,02	ARL5B	ADP-ribosylation factor like GTPase 5B
1,02	LOC149373; RP5-1097F14.3; kosimo	uncharacterized LOC149373; Transcript Identified by AceView; novel transcript
1,02	ZSCAN22; MIR6806	zinc finger and SCAN domain containing 22; microRNA 6806
1,02	RNF11	ring finger protein 11
1,02	RBBP8; MIR4741	retinoblastoma binding protein 8; microRNA 4741
1,02	DYRK1B	dual specificity tyrosine-(Y)-phosphorylation regulated kinase 1B
1,02	TAS2R46	taste receptor, type 2, member 46
1,02	EHD2	EH domain containing 2

1,02	OR2T33	olfactory receptor, family 2, subfamily T, member 33
1,02	FAM129C	family with sequence similarity 129, member C
1,02	TGOLN2	Memczak2013 ANTISENSE, CDS, coding, INTERNAL best transcript NM_001206844
1,02	TRIM8	tripartite motif containing 8
1,02	SERP2	stress-associated endoplasmic reticulum protein family member 2
1,02	APOE	apolipoprotein E
1,02	CCDC153	coiled-coil domain containing 153
1,02	ERI1	exoribonuclease 1
1,02	CDK9	cyclin-dependent kinase 9
1,02	PRKCA	protein kinase C, alpha
1,02	SF3A3	splicing factor 3a subunit 3
1,02	LDLRAD2	low density lipoprotein receptor class A domain containing 2
1,02	RAC2	ras-related C3 botulinum toxin substrate 2 (rho family, small GTP binding protein Rac2)
1,02	AKR1B1	aldo-keto reductase family 1, member B1 (aldose reductase)
1,02	BCAR1	breast cancer anti-estrogen resistance 1
1,02	PMFBP1	polyamine modulated factor 1 binding protein 1
1,02	TOR1AIP1	torsin A interacting protein 1
1,02	CCDC117	coiled-coil domain containing 117
1,02	SPRR2E	small proline-rich protein 2E
1,02	PDLIM3	PDZ and LIM domain 3
1,02	THOC5	THO complex 5
1,02	C18orf32	chromosome 18 open reading frame 32
1,02	C22orf24	chromosome 22 open reading frame 24
1,02	BEND7	BEN domain containing 7
1,02	ARC	activity-regulated cytoskeleton-associated protein
1,02	MAST3	microtubule associated serine/threonine kinase 3
1,02	RNF215	ring finger protein 215
1,02	RIF1	replication timing regulatory factor 1
1,02	SIGMAR1	sigma non-opioid intracellular receptor 1
1,02	FOXC1	forkhead box C1
1,02	CCDC189	coiled-coil domain containing 189
1,02	PGRMC2	progesterone receptor membrane component 2
1,02	KLF16	Kruppel-like factor 16
1,02	CRIPT	cysteine-rich PDZ-binding protein
1,02	TFE3	transcription factor binding to IGHM enhancer 3
1,02	ZNF585B	zinc finger protein 585B
1,02	MGRN1	mahogunin ring finger 1, E3 ubiquitin protein ligase
1,02	ATXN2	ataxin 2
1,02	LOC101928161; AC010890.1; NCKAP5; spawfa	uncharacterized LOC101928161; Salzman2013 ANTISENSE, coding, INTERNAL, intronic, OVERLAPTX best transcript NM_207363; novel transcript, antisense to NCKAP5; Transcript Identified by AceView
1,02	PNLIPRP1	pancreatic lipase-related protein 1
1,02	ANO7	anoctamin 7

1,02	PPP1R13L	protein phosphatase 1, regulatory subunit 13 like
1,02	CREB5	cAMP responsive element binding protein 5
1,02	TACC2	transforming, acidic coiled-coil containing protein 2
1,02	SNX27	sorting nexin family member 27
1,02	CFAP57	cilia and flagella associated protein 57
1,02	KPNA4	karyopherin alpha 4 (importin alpha 3)
1,02	SLTM	SAFB-like, transcription modulator
1,02	NRCAM	neuronal cell adhesion molecule
1,02	GLTSCR1L	GLTSCR1-like
1,02	OR6K3	olfactory receptor, family 6, subfamily K, member 3
1,02	CNOT7	CCR4-NOT transcription complex subunit 7
1,02	FAM109B	family with sequence similarity 109, member B
1,02	RAB33A	RAB33A, member RAS oncogene family
1,02	RING1	ring finger protein 1
1,02	EMC1	ER membrane protein complex subunit 1
1,02	CA5B	carbonic anhydrase VB, mitochondrial
1,02	USPL1	ubiquitin specific peptidase like 1
1,02	RNASEH1	ribonuclease H1
1,02	GMIP	GEM interacting protein
1,02	TEX14	testis expressed 14
1,02	STAG2	stromal antigen 2
1,02	USP14	ubiquitin specific peptidase 14 (tRNA-guanine transglycosylase)
1,02	MIPOL1	mirror-image polydactyly 1
1,02	EXOSC4; MIR6847	exosome component 4; microRNA 6847
1,02	PDZRN3	PDZ domain containing ring finger 3
1,02	SLC43A2	solute carrier family 43 (amino acid system L transporter), member 2
1,02	ETV3	ets variant 3
1,02	BCL2	B-cell CLL/lymphoma 2
1,02	DLD	dihydrolipoamide dehydrogenase
1,02	ALDH1A1	aldehyde dehydrogenase 1 family, member A1
1,02	SYT7	synaptotagmin VII
1,02	BOLA2B; BOLA2	bolA family member 2B; bolA family member 2
1,02	THAP4	THAP domain containing 4
1,02	P2RY4	pyrimidinergic receptor P2Y, G-protein coupled, 4
1,02	SOX5	Transcript Identified by AceView, Entrez Gene ID(s) 6660
1,02	MRPL9	mitochondrial ribosomal protein L9
1,02	SLC46A3	solute carrier family 46, member 3
1,02	SYNDIG1L	synapse differentiation inducing 1-like
1,02	PRR5	proline rich 5 (renal)
1,02	SLC26A6; MIR6824	solute carrier family 26 (anion exchanger), member 6; microRNA 6824
1,02	SPATC1L	spermatogenesis and centriole associated 1-like
1,02	TMEM86B	transmembrane protein 86B
1,02	EEPD1	endonuclease/exonuclease/phosphatase family domain containing 1

1,02	CDC73	cell division cycle 73
1,02	GSTCD	glutathione S-transferase, C-terminal domain containing
1,02	SLC12A6	solute carrier family 12 (potassium/chloride transporter), member 6
1,02	TCOF1	Treacher Collins-Franceschetti syndrome 1
1,02	DDX17	DEAD (Asp-Glu-Ala-Asp) box helicase 17
1,02	TADA2B	transcriptional adaptor 2B
1,02	TNFRSF10C	tumor necrosis factor receptor superfamily, member 10c, decoy without an intracellular domain
1,02	RTFDC1	replication termination factor 2 domain containing 1
1,02	FZD4	frizzled class receptor 4
1,02	NEK11	NIMA-related kinase 11
1,02	C2orf57	chromosome 2 open reading frame 57
1,02	CREB1	cAMP responsive element binding protein 1
1,02	NR1I2	nuclear receptor subfamily 1, group I, member 2
1,02	ALK	anaplastic lymphoma receptor tyrosine kinase
1,02	IL17RA	interleukin 17 receptor A
1,02	ANKDD1B	ankyrin repeat and death domain containing 1B
1,02	AGAP4	ArfGAP with GTPase domain, ankyrin repeat and PH domain 4
1,02	EP400NL	EP400 N-terminal like
1,02	GJA5	gap junction protein alpha 5
1,02	IMPDH2	IMP (inosine 5-monophosphate) dehydrogenase 2
1,02	RINT1	RAD50 interactor 1
1,02	VMO1	vitelline membrane outer layer 1 homolog (chicken)
1,02	ZNF765	zinc finger protein 765
1,02	NIFK	nucleolar protein interacting with the FHA domain of MKI67
1,02	CCNL2	cyclin L2
1,02	ACAP3	ArfGAP with coiled-coil, ankyrin repeat and PH domains 3
1,02	PLA2G2F	phospholipase A2, group IIF
1,02	HSPA4L	heat shock 70kDa protein 4-like
1,02	MRPS17	mitochondrial ribosomal protein S17
1,02	GALNT1	polypeptide N-acetylgalactosaminyltransferase 1
1,02	ZCCHC4	zinc finger, CCHC domain containing 4
1,02	KLK5	kallikrein related peptidase 5
1,02	TAAR8	trace amine associated receptor 8
1,02	HEPACAM	hepatic and glial cell adhesion molecule
1,02	ZNF768	zinc finger protein 768
1,02	PDE6C	phosphodiesterase 6C, cGMP-specific, cone, alpha prime
1,02	WAC	WW domain containing adaptor with coiled-coil
1,02	FBL	fibrillarin
1,02	RBM18	RNA binding motif protein 18
1,02	CPXM1	carboxypeptidase X (M14 family), member 1
1,02	PRKAG2	protein kinase, AMP-activated, gamma 2 non-catalytic subunit
1,02	HYOU1	hypoxia up-regulated 1

1,02	CYP51A1	cytochrome P450, family 51, subfamily A, polypeptide 1
1,02	RTCB	RNA 2,3-cyclic phosphate and 5-OH ligase
1,02	TCTEX1D1	Tctex1 domain containing 1
1,02	POM121C	POM121 transmembrane nucleoporin C
1,02	MPV17	MpV17 mitochondrial inner membrane protein
1,02	CHADL	chondroadherin-like
1,02	FOXR1	forkhead box R1
1,02	PRAMEF12	PRAME family member 12
1,02	DKK3	dickkopf WNT signaling pathway inhibitor 3
1,02	C1QTNF6	C1q and tumor necrosis factor related protein 6
1,02	AMDHD2	amidohydrolase domain containing 2
1,02	CASC10	cancer susceptibility candidate 10
1,02	STXBP2	syntaxin binding protein 2
1,02	UGGT2	UDP-glucose glycoprotein glucosyltransferase 2
1,02	PTPN18	protein tyrosine phosphatase, non-receptor type 18 (brain-derived)
1,02	RBSN	rabenosyn, RAB effector
1,02	ELOVL5	ELOVL fatty acid elongase 5
1,02	FBL	fibrillarin
1,02	CYP2C19	cytochrome P450, family 2, subfamily C, polypeptide 19
1,02	TSR1	TSR1, 20S rRNA accumulation, homolog (S. cerevisiae)
1,02	PTGIS	prostaglandin I2 (prostacyclin) synthase
1,02	OR11H2	olfactory receptor, family 11, subfamily H, member 2
1,02	PDDC1	Parkinson disease 7 domain containing 1
1,02	MAP4K3	mitogen-activated protein kinase kinase kinase kinase 3
1,02	CCDC179	coiled-coil domain containing 179
1,02	GLRX3	glutaredoxin 3
1,02	GOLGA6L3	golgin A6 family-like 3
1,02	RUFY4	RUN and FYVE domain containing 4
1,02	PHF19	Transcript Identified by AceView, Entrez Gene ID(s) 26147
1,02	ZNF792	zinc finger protein 792
1,02	CHIC1	cysteine rich hydrophobic domain 1
1,02	DGKD	diacylglycerol kinase, delta 130kDa
1,02	EXOSC1	exosome component 1
1,02	ZNF345	zinc finger protein 345
1,02	NMD3	NMD3 ribosome export adaptor
1,02	IDUA	iduronidase, alpha-L-
1,02	SH3BP2	SH3-domain binding protein 2
1,02	TCF7	transcription factor 7 (T-cell specific, HMG-box)
1,02	SCAMP4; ADAT3	secretory carrier membrane protein 4; adenosine deaminase, tRNA-specific 3
1,02	UBE2F	ubiquitin-conjugating enzyme E2F (putative)
1,02	TRAPPC5	trafficking protein particle complex 5
1,02	GLUD2	glutamate dehydrogenase 2

1,02	GNG10	guanine nucleotide binding protein (G protein), gamma 10
1,02	KRTAP3-3	keratin associated protein 3-3
1,02	GCM1	glial cells missing homolog 1 (Drosophila)
1,02	GAP43	growth associated protein 43
1,02	ZNF146	zinc finger protein 146
1,02	PDE6B	phosphodiesterase 6B, cGMP-specific, rod, beta
1,02	PLPP7	phospholipid phosphatase 7 (inactive)
1,02	PAPD5	PAP associated domain containing 5
1,02	FJX1	four jointed box 1
1,02	FGFR1	fibroblast growth factor receptor 1
1,02	ZNF534	zinc finger protein 534
1,02	ADAM29	ADAM metallopeptidase domain 29
1,02	SREK1	splicing regulatory glutamine/lysine-rich protein 1
1,02	RUFY1	RUN and FYVE domain containing 1
1,02	HMGA2	high mobility group AT-hook 2
1,02	CLPTM1L	CLPTM1-like
1,02	ANKLE2	ankyrin repeat and LEM domain containing 2
1,02	SUN2	Sad1 and UNC84 domain containing 2
1,02	SMARCAD1	SWI/SNF-related, matrix-associated actin-dependent regulator of chromatin, subfamily a, containing DEAD/H box 1
1,02	C17orf50	chromosome 17 open reading frame 50
1,02	C20orf194	chromosome 20 open reading frame 194
1,02	HNRNPA0	heterogeneous nuclear ribonucleoprotein A0
1,02	CPM	carboxypeptidase M
1,02	CATSPERD	catsper channel auxiliary subunit delta
1,02	OSM	oncostatin M
1,02	TRAPPC9	trafficking protein particle complex 9
1,02	LETM2	leucine zipper-EF-hand containing transmembrane protein 2
1,02	TAF1B	TATA box binding protein (TBP)-associated factor, RNA polymerase I, B, 63kDa
1,02	SDK1	sidekick cell adhesion molecule 1
1,02	INO80B	INO80 complex subunit B
1,02	HSP90B1; MIR3652	heat shock protein 90kDa beta (Grp94), member 1; microRNA 3652
1,02	LCE3D	late cornified envelope 3D
1,02	SLC35D1	solute carrier family 35 (UDP-GlcA/UDP-GalNAc transporter), member D1
1,02	PEX3	peroxisomal biogenesis factor 3
1,02	TAF1D; SNORA8; SNORA18; SNORA40; SNORA1; SNORA25; SNORA32; SNORD5; MIR1304	TATA box binding protein associated factor 1D; small nucleolar RNA, H/ACA box 8; small nucleolar RNA, H/ACA box 18; small nucleolar RNA, H/ACA box 40; small nucleolar RNA, H/ACA box 1; small nucleolar RNA, H/ACA box 25; small nucleolar RNA, H/ACA box 32; small nucleolar RNA, C/D box 5; microRNA 1304
1,02	ACTL10	actin-like 10

1,02	PSMC2	proteasome 26S subunit, ATPase 2
1,02	UNK	unkempt family zinc finger
1,02	DUSP1	dual specificity phosphatase 1
1,02	MYO9B	myosin IXB
1,02	ND5	NADH dehydrogenase, subunit 5 (complex I)
1,02	DMTF1	cyclin D binding myb-like transcription factor 1
1,02	TBC1D3L; TBC1D3E	TBC1 domain family, member 3L; TBC1 domain family, member 3E
1,02	CRYZ	crystallin zeta
1,02	GABRA2	gamma-aminobutyric acid (GABA) A receptor, alpha 2
1,02	RGPD5; RGPD8	RANBP2-like and GRIP domain containing 5; RANBP2-like and GRIP domain containing 8
1,02	CTAG1B	cancer/testis antigen 1B
1,02	DCK	deoxycytidine kinase
1,02	SMIM11A	small integral membrane protein 11A
1,02	ISL1	ISL LIM homeobox 1
1,02	SCYL2	SCY1-like, kinase-like 2
1,02	SCAF11	SR-related CTD-associated factor 11
1,02	APEX1	APEX nuclease (multifunctional DNA repair enzyme) 1
1,02	RAB22A	RAB22A, member RAS oncogene family
1,02	VPS53; RPS4XP17	vacuolar protein sorting 53 homolog (S. cerevisiae); ribosomal protein S4X pseudogene 17
1,02	LIPA	lipase A, lysosomal acid, cholesterol esterase
1,02	SSR1	signal sequence receptor, alpha
1,02	STKLD1	serine/threonine kinase-like domain containing 1 [Source:HGNC Symbol;Acc:HGNC:28669]
1,02	SAV1	salvador family WW domain containing protein 1
1,02	FGD4	FYVE, RhoGEF and PH domain containing 4
1,02	PTPN2	protein tyrosine phosphatase, non-receptor type 2
1,02	OR7G1	olfactory receptor, family 7, subfamily G, member 1
1,02	CAMTA1	calmodulin binding transcription activator 1
1,02	CCSER2	coiled-coil serine rich protein 2
1,02	LOC388813; bafor; AF165138.7	uncharacterized protein ENSP00000383407-like; Transcript Identified by AceView; HCG1818297; Protein LOC388813 [Source:UniProtKB/TrEMBL;Acc:A0A087WSY0]
1,02	MOB4	MOB family member 4, phocein
1,02	KCNK3	potassium channel, two pore domain subfamily K, member 3
1,02	G6PD	glucose-6-phosphate dehydrogenase
1,02	GLRB	glycine receptor beta
1,02	ATP13A5	ATPase type 13A5
1,02	SP2	Sp2 transcription factor
1,02	EIF1	eukaryotic translation initiation factor 1
1,02	CRCP	CGRP receptor component
1,02	RAPGEF2	Rap guanine nucleotide exchange factor 2
1,02	TIA1	TIA1 cytotoxic granule-associated RNA binding protein

1,02	C14orf166	chromosome 14 open reading frame 166
1,02	FGF2	fibroblast growth factor 2 (basic)
1,02	ZNF99	zinc finger protein 99
1,02	ATP2B3	ATPase, Ca++ transporting, plasma membrane 3
1,02	TIMM44	translocase of inner mitochondrial membrane 44 homolog (yeast)
1,02	QSOX1	quiescin Q6 sulfhydryl oxidase 1
1,02	NLRP13	NLR family, pyrin domain containing 13
1,02	ZNF321P	zinc finger protein 321, pseudogene
1,02	DBR1	debranching RNA lariats 1
1,02	PACSIN3	protein kinase C and casein kinase substrate in neurons 3
1,02	TIMM8B	translocase of inner mitochondrial membrane 8 homolog B (yeast)
1,02	CDH1	cadherin 1, type 1
1,02	LY6G5B; CSNK2B	lymphocyte antigen 6 complex, locus G5B; casein kinase 2, beta polypeptide
1,02	CYSLTR1	cysteinyl leukotriene receptor 1
1,02	GNRH2	gonadotropin releasing hormone 2
1,02	SLC38A3	solute carrier family 38, member 3
1,02	HEYL	Transcript Identified by AceView, Entrez Gene ID(s) 26508
1,02	IK; MIR3655	IK cytokine, down-regulator of HLA II; microRNA 3655
1,02	CARNMT1	carnosine N-methyltransferase 1
1,02	FGD3	FYVE, RhoGEF and PH domain containing 3
1,02	MUL1	mitochondrial E3 ubiquitin protein ligase 1
1,02	ZNF317	zinc finger protein 317
1,02	ELF1	E74-like factor 1 (ets domain transcription factor)
1,02	C12orf71	chromosome 12 open reading frame 71
1,02	PGF	placental growth factor
1,02	MGAT2	mannosyl (alpha-1,6-)-glycoprotein beta-1,2-N-acetylglucosaminyltransferase
1,02	DDX60L	DEAD (Asp-Glu-Ala-Asp) box polypeptide 60-like
1,02	HMBS	hydroxymethylbilane synthase
1,02	TPRXL	tetra-peptide repeat homeobox-like
1,02	RCC2	regulator of chromosome condensation 2
1,02	TSG101	tumor susceptibility 101
1,02	MEPCE	methylphosphate capping enzyme
1,02	MRPS22	mitochondrial ribosomal protein S22
1,02	SRSF8	serine/arginine-rich splicing factor 8
1,02	COA7	cytochrome c oxidase assembly factor 7 (putative)
1,02	NBEAL2	neurobeachin like 2
1,02	DNAJC9	DnaJ (Hsp40) homolog, subfamily C, member 9
1,02	YBX3	Y box binding protein 3
1,02	RPGRIP1L	RPGRIP1-like
1,02	NDNF	neuron-derived neurotrophic factor
1,02	EPN3	epsin 3
1,02	LKAAEAR1	LKAAEAR motif containing 1
1,02	OR56B1	olfactory receptor, family 56, subfamily B, member 1

1,02	PMPCB	peptidase (mitochondrial processing) beta
1,02	ADNP2	ADNP homeobox 2
1,02	RAB11A	RAB11A, member RAS oncogene family
1,02	DOCK6	dedicator of cytokinesis 6
1,02	SPANXA1	sperm protein associated with the nucleus, X-linked, family member A1
1,02	KCNIP2	Kv channel interacting protein 2
1,02	LRFN3	leucine rich repeat and fibronectin type III domain containing 3
1,02	CD48	CD48 molecule
1,02	ANXA1	annexin A1
1,02	KMT5B	lysine (K)-specific methyltransferase 5B
1,02	SRPK3	SRSF protein kinase 3
1,02	HOXA2	homeobox A2
1,02	FUT1	fucosyltransferase 1 (galactoside 2-alpha-L-fucosyltransferase, H blood group)
1,02	LEAP2	liver expressed antimicrobial peptide 2
1,02	BABAM1	BRISC and BRCA1 A complex member 1
1,02	SERPINH1	Jeck2013 ANTISENSE, coding, INTERNAL, intronic best transcript NM_001207014
1,02	CPO	carboxypeptidase O
1,02	ZBTB34	zinc finger and BTB domain containing 34
1,02	PAPD7	PAP associated domain containing 7
1,02	U2AF1	U2 small nuclear RNA auxiliary factor 1
1,02	ULBP2	UL16 binding protein 2
1,02	IFIH1	interferon induced, with helicase C domain 1
1,02	SOX12	SRY box 12
1,02	NUP98	Transcript Identified by AceView, Entrez Gene ID(s) 4928
1,02	HOXA13	homeobox A13
1,02	PSMB6	proteasome subunit beta 6
1,02	ALOXE3	arachidonate lipoxygenase 3
1,02	MFN2	mitofusin 2
1,02	PRSS21	protease, serine, 21 (testisin)
1,02	YPEL1	yippee like 1
1,02	NXF5	nuclear RNA export factor 5
1,02	RPL23; SNORA21	ribosomal protein L23; small nucleolar RNA, H/ACA box 21
1,02	TRIM24	tripartite motif containing 24
1,02	GCDH	glutaryl-CoA dehydrogenase
1,02	MFSD11	major facilitator superfamily domain containing 11
1,02	TTYH3	tweety family member 3
1,02	ND4L; ND4	NADH dehydrogenase, subunit 4L (complex I); NADH dehydrogenase, subunit 4 (complex I)
1,02	LRRC16A	leucine rich repeat containing 16A
1,02	CXorf49B; CXorf49	chromosome X open reading frame 49B; chromosome X open reading frame 49
1,02	PEX5L	peroxisomal biogenesis factor 5-like
1,02	MFF	mitochondrial fission factor

1,02	LHPP	phospholysine phosphohistidine inorganic pyrophosphate phosphatase
1,02	PAQR6	progestin and adipoQ receptor family member VI
1,02	CFD	complement factor D (adipsin)
1,02	POPDC3	popeye domain containing 3
1,02	BEAN1-AS1	BEAN1 antisense RNA 1
1,02	GADD45B	growth arrest and DNA-damage-inducible, beta
1,02	SATB2	SATB homeobox 2
1,02	PAFAH1B3	platelet-activating factor acetylhydrolase 1b, catalytic subunit 3 (29kDa)
1,02	GFM1	G elongation factor, mitochondrial 1
1,02	HOXA4	homeobox A4
1,02	HSD17B6	hydroxysteroid (17-beta) dehydrogenase 6
1,02	MSRB3	methionine sulfoxide reductase B3
1,02	PTH1R	parathyroid hormone 1 receptor
1,02	PHLDB1; MIR6716	pleckstrin homology-like domain, family B, member 1; microRNA 6716
1,02	RBM38	RNA binding motif protein 38
1,02	C5orf63	chromosome 5 open reading frame 63
1,02	RAP2B	RAP2B, member of RAS oncogene family
1,02	PTK2B	protein tyrosine kinase 2 beta
1,02	PPP1R14C	protein phosphatase 1, regulatory (inhibitor) subunit 14C
1,02	R3HCC1	R3H domain and coiled-coil containing 1
1,02	AGFG1; MIR5703	ArfGAP with FG repeats 1; microRNA 5703
1,02	ASAH2	N-acylsphingosine amidohydrolase (non-lysosomal ceramidase) 2
1,02	MINPP1	multiple inositol-polyphosphate phosphatase 1
1,02	ADAMTSL5	ADAMTS like 5
1,02	PPIG	peptidylprolyl isomerase G (cyclophilin G)
1,02	OSBPL8	oxysterol binding protein-like 8
1,02	GLUD1	glutamate dehydrogenase 1
1,02	OR10J4	olfactory receptor, family 10, subfamily J, member 4 (gene/pseudogene) [Source:HGNC Symbol;Acc:HGNC:15408]
1,02	CRCT1	cysteine rich C-terminal 1
1,02	PSMG1	proteasome (prosome, macropain) assembly chaperone 1
1,02	DCST2	DC-STAMP domain containing 2
1,02	SCAF4	SR-related CTD-associated factor 4
1,02	SLC13A4	solute carrier family 13 (sodium/sulfate symporter), member 4
1,02	NOP16	NOP16 nucleolar protein
1,02	LINC01587	long intergenic non-protein coding RNA 1587
1,02	KIAA2013	KIAA2013
1,02	C1orf146	chromosome 1 open reading frame 146
1,02	TAB3	TGF-beta activated kinase 1/MAP3K7 binding protein 3
1,02	IFNA2	interferon, alpha 2

1,02	TTC21B	tetratricopeptide repeat domain 21B
1,02	BMP8A	bone morphogenetic protein 8a
1,02	WDR92	WD repeat domain 92
1,02	GATAD1	GATA zinc finger domain containing 1
1,02	EBF3	early B-cell factor 3
1,02	IL5	interleukin 5
1,02	KRT7	keratin 7, type II
1,02	C1QTNF1	C1q and tumor necrosis factor related protein 1
1,02	TNF	tumor necrosis factor
1,02	ZNF780A	zinc finger protein 780A
1,02	HPCA	hippocalcin
1,02	PTCHD4	patched domain containing 4
1,02	MIPOL1	mirror-image polydactyly 1
1,02	FAM3C2	FAM3C pseudogene
1,02	CCR6	chemokine (C-C motif) receptor 6
1,02	C1orf35	chromosome 1 open reading frame 35
1,02	EPT1	ethanolaminephosphotransferase 1
1,02	CTTNBP2NL	CTTNBP2 N-terminal like
1,02	GCSH	glycine cleavage system protein H (aminomethyl carrier)
1,02	DUOXA1	dual oxidase maturation factor 1
1,02	PTTG1IP	pituitary tumor-transforming 1 interacting protein
1,02	WFIKKN2	WAP, follistatin/kazal, immunoglobulin, kunitz and netrin domain containing 2
1,02	TP53I11	Memczak2013 ALT_ACCEPTOR, ALT_DONOR, coding, INTERNAL, intronic best transcript NM_001076787
1,02	CCDC92	coiled-coil domain containing 92
1,02	RXRA	Memczak2013 ANTISENSE, CDS, coding, INTERNAL best transcript NM_002957
1,02	PLS3	plastin 3
1,02	PLG	plasminogen
1,02	CACNA2D4	calcium channel, voltage-dependent, alpha 2/delta subunit 4
1,02	SHF	Src homology 2 domain containing F
1,02	BNIP2	BCL2/adenovirus E1B 19kDa interacting protein 2
1,02	CLASP1	cytoplasmic linker associated protein 1
1,02	SCAND2P	SCAN domain containing 2 pseudogene
1,02	LUZP4	leucine zipper protein 4
1,02	AHSA2	AHA1, activator of heat shock 90kDa protein ATPase homolog 2 (yeast)
1,02	P2RY14	purinergic receptor P2Y, G-protein coupled, 14
1,02	TMEM252	transmembrane protein 252
1,02	CXorf51A; CXorf51B; CXorf51	Homo sapiens chromosome X open reading frame 51A (CXorf51A), mRNA.; Homo sapiens chromosome X open reading frame 51B (CXorf51B), mRNA.; Transcript Identified by AceView, Entrez Gene ID(s) 100129239, RefSeq ID(s) NM_001144064; chromosome X open reading frame 51A [Source:HGNC Symbol;Acc:HGNC:30533]; chromosome X open reading frame 51

1,02	TNFRSF10D	tumor necrosis factor receptor superfamily, member 10d, decoy with truncated death domain
1,02	POGK	pogo transposable element with KRAB domain
1,02	SPCS3	signal peptidase complex subunit 3
1,02	NDUFC1	NADH dehydrogenase (ubiquinone) 1, subcomplex unknown, 1, 6kDa
1,02	STAG3L4	stromal antigen 3-like 4 (pseudogene)
1,02	UCHL5	ubiquitin C-terminal hydrolase L5
1,02	EFR3A	EFR3 homolog A
1,02	RRP1B	ribosomal RNA processing 1B
1,02	CCDC17	coiled-coil domain containing 17
1,02	RPIA	ribose 5-phosphate isomerase A
1,02	ADO	2-aminoethanethiol (cysteamine) dioxygenase
1,02	GTF2H4	general transcription factor IIH subunit 4
1,02	C19orf70	chromosome 19 open reading frame 70
1,02	TMPRSS13	transmembrane protease, serine 13
1,02	DEGS1	delta(4)-desaturase, sphingolipid 1
1,02	COPB1	coatomer protein complex subunit beta 1
1,02	SHANK1	SH3 and multiple ankyrin repeat domains 1
1,02	ZYX	zyxin
1,02	UPK3A	uroplakin 3A
1,02	TPX2	TPX2, microtubule-associated
1,02	CALU	calumenin
1,02	SCOC	short coiled-coil protein
1,02	KLF11	Kruppel-like factor 11
1,02	TMIGD1	transmembrane and immunoglobulin domain containing 1
1,02	PLA2G2E	phospholipase A2, group IIE
1,02	ADORA2A	adenosine A2a receptor
1,02	SPATA31D1	SPATA31 subfamily D, member 1
1,02	P2RX1	purinergic receptor P2X, ligand gated ion channel, 1
1,02	AGAP6	ArfGAP with GTPase domain, ankyrin repeat and PH domain 6
1,02	PADI1	peptidyl arginine deiminase, type I
1,02	POTEB3	POTE ankyrin domain family, member B3
1,02	MARK2	MAP/microtubule affinity-regulating kinase 2
1,02	CCL22	chemokine (C-C motif) ligand 22
1,02	WSB1	WD repeat and SOCS box containing 1
1,02	TTC24	tetratricopeptide repeat domain 24
1,02	AATF	apoptosis antagonizing transcription factor
1,02	ADA	adenosine deaminase
1,02	EPB41L1	erythrocyte membrane protein band 4.1-like 1
1,02	INCA1	inhibitor of CDK, cyclin A1 interacting protein 1
1,02	KIAA0196	KIAA0196

1,02	LOC388242; LOC613038; RP11-347C12.3	Homo sapiens coiled-coil domain containing 101 pseudogene (LOC388242), non-coding RNA.; Homo sapiens SAGA complex associated factor 29 pseudogene (LOC613038), non-coding RNA.; Transcript Identified by AceView, Entrez Gene ID(s) 613038, RefSeq ID(s) NR_002557; Transcript Identified by AceView, Entrez Gene ID(s) 613038; Protein LOC101929849 [Source:UniProtKB/TrEMBL;Acc:H0YHN6]
1,02	FAM199X	family with sequence similarity 199, X-linked
1,02	FBLN2	fibulin 2
1,02	ZC3H18	zinc finger CCCH-type containing 18
1,02	INPP5D	inositol polyphosphate-5-phosphatase D
1,02	PDLIM2	PDZ and LIM domain 2 (mystique)
1,02	GNB5	guanine nucleotide binding protein (G protein), beta 5
1,02	TRIM11; MIR6742	tripartite motif containing 11; microRNA 6742
1,02	EID2	EP300 interacting inhibitor of differentiation 2
1,02	SPG7	spastic paraplegia 7 (pure and complicated autosomal recessive)
1,02	FAM49B	family with sequence similarity 49, member B
1,02	EPHA10	EPH receptor A10
1,02	CTTN	cortactin
1,02	PFKFB1	6-phosphofructo-2-kinase/fructose-2,6-biphosphatase 1
1,02	MOCS3	molybdenum cofactor synthesis 3
1,02	SLC3A2	solute carrier family 3 (amino acid transporter heavy chain), member 2
1,02	GOLGA8T	golgin A8 family, member T
1,01	NUP107	nucleoporin 107kDa
1,01	UBA3	ubiquitin-like modifier activating enzyme 3
1,01	OR1D5	olfactory receptor, family 1, subfamily D, member 5
1,01	PDE12	phosphodiesterase 12
1,01	LTV1	LTV1 ribosome biogenesis factor
1,01	RPS24	ribosomal protein S24
1,01	LIN9	lin-9 DREAM MuvB core complex component
1,01	MILR1	mast cell immunoglobulin-like receptor 1
1,01	TGIF2LY	TGFB-induced factor homeobox 2-like, Y-linked
1,01	HS3ST3A1	Jeck2013 ALT_ACCEPTOR, ALT_DONOR, coding, INTERNAL, intronic best transcript NM_006042
1,01	KDM1A; MIR3115	lysine (K)-specific demethylase 1A; microRNA 3115
1,01	WHSC1	Wolf-Hirschhorn syndrome candidate 1
1,01	SRRM2	serine/arginine repetitive matrix 2
1,01	STAM2	signal transducing adaptor molecule (SH3 domain and ITAM motif) 2
1,01	STX6	syntaxin 6
1,01	RNF8	ring finger protein 8, E3 ubiquitin protein ligase
1,01	ASPA	aspartoacylase
1,01	PGGT1B	protein geranylgeranyltransferase type I, beta subunit

1,01	APPBP2	amyloid beta precursor protein (cytoplasmic tail) binding protein 2
1,01	CKAP2	cytoskeleton associated protein 2
1,01	ALOX12B	arachidonate 12-lipoxygenase, 12R type
1,01	FAM65A	family with sequence similarity 65, member A
1,01	USP42	ubiquitin specific peptidase 42
1,01	DMP1	dentin matrix acidic phosphoprotein 1
1,01	TMEM68	transmembrane protein 68
1,01	MED10	mediator complex subunit 10
1,01	HDAC1	histone deacetylase 1
1,01	ZMYM1	zinc finger, MYM-type 1
1,01	B9D2	B9 protein domain 2
1,01	RPGRIP1	retinitis pigmentosa GTPase regulator interacting protein 1
1,01	FAM193B	family with sequence similarity 193, member B
1,01	EPC2	enhancer of polycomb homolog 2 (Drosophila)
1,01	MX1	MX dynamin-like GTPase 1
1,01	CEP63	centrosomal protein 63kDa
1,01	HIST3H2BB	histone cluster 3, H2bb
1,01	MCF2L	MCF.2 cell line derived transforming sequence like
1,01	SLC39A6	solute carrier family 39 (zinc transporter), member 6
1,01	TUSC1	tumor suppressor candidate 1
1,01	RAD21; MIR3610	RAD21 cohesin complex component; microRNA 3610
1,01	ANKH	ANKH inorganic pyrophosphate transport regulator
1,01	PLCB1	Memczak2013 ANTISENSE, coding, INTERNAL, intronic best transcript NM_182734
1,01	NPBWR2	neuropeptides B/W receptor 2
1,01	ZNF611	zinc finger protein 611
1,01	CAPN7	calpain 7
1,01	ANKRD11	ankyrin repeat domain 11
1,01	RXFP1	relaxin/insulin-like family peptide receptor 1
1,01	SRSF6	serine/arginine-rich splicing factor 6
1,01	PIDD1	p53-induced death domain protein 1
1,01	DMRTB1	DMRT-like family B with proline-rich C-terminal, 1
1,01	UQCRB	ubiquinol-cytochrome c reductase binding protein
1,01	USP24	Transcript Identified by AceView, Entrez Gene ID(s) 23358
1,01	SRSF5	serine/arginine-rich splicing factor 5
1,01	OR4C12	olfactory receptor, family 4, subfamily C, member 12
1,01	ST7; ST7-OT4; ST7-OT3; MIR6132	suppression of tumorigenicity 7; ST7 overlapping transcript 4; ST7 overlapping transcript 3; microRNA 6132
1,01	MASP2	mannan-binding lectin serine peptidase 2
1,01	PGAP3	post-GPI attachment to proteins 3
1,01	PSMA5	proteasome subunit alpha 5
1,01	G6PC2	glucose-6-phosphatase, catalytic, 2
1,01	HOXA6	homeobox A6
1,01	GCFC2	GC-rich sequence DNA-binding factor 2
1,01	SAR1B	secretion associated, Ras related GTPase 1B

1,01	GOLT1B	golgi transport 1B
1,01	INTS8	integrator complex subunit 8
1,01	PRKACB	protein kinase, cAMP-dependent, catalytic, beta
1,01	MYF5	myogenic factor 5
1,01	KCTD11	potassium channel tetramerization domain containing 11
1,01	VN1R5	vomeronasal 1 receptor 5 (gene/pseudogene)
1,01	CIITA	class II, major histocompatibility complex, transactivator
1,01	CDC26	cell division cycle 26
1,01	RPL7A	ribosomal protein L7a
1,01	S100A9	S100 calcium binding protein A9
1,01	PHB	prohibitin
1,01	BAG3	BCL2-associated athanogene 3
1,01	LDHB	lactate dehydrogenase B
1,01	GSG1	germ cell associated 1
1,01	ANO10	anoctamin 10
1,01	EBNA1BP2; MIR6733	EBNA1 binding protein 2; microRNA 6733
1,01	ATP1B1	ATPase, Na+/K+ transporting, beta 1 polypeptide
1,01	SNX8; MIR6836	sorting nexin 8; microRNA 6836
1,01	RNF149	ring finger protein 149
1,01	PUS3	pseudouridylate synthase 3
1,01	GTF2I	general transcription factor IIi
1,01	TEX101	testis expressed 101
1,01	APRT	adenine phosphoribosyltransferase
1,01	SNAP23	synaptosome associated protein 23kDa
1,01	SOX6; MIR6073	SRY box 6; microRNA 6073
1,01	VAPA	Memczak2013 ALT_ACCEPTOR, ALT_DONOR, coding, INTERNAL, intronic best transcript NM_003574
1,01	RNF183	ring finger protein 183
1,01	RTP4	receptor (chemosensory) transporter protein 4
1,01	RANBP2	RAN binding protein 2
1,01	MAN2C1	mannosidase, alpha, class 2C, member 1
1,01	BROX	BRO1 domain and CAAX motif containing
1,01	EXOSC3	exosome component 3
1,01	HIST1H2BH	histone cluster 1, H2bh
1,01	ZNF354B	zinc finger protein 354B
1,01	ANP32E	acidic nuclear phosphoprotein 32 family member E
1,01	SNRPC	small nuclear ribonucleoprotein polypeptide C
1,01	MPP7	membrane protein, palmitoylated 7
1,01	TRAPPC6A	trafficking protein particle complex 6A
1,01	TTI1	TELO2 interacting protein 1
1,01	C10orf2	chromosome 10 open reading frame 2
1,01	PIGC	phosphatidylinositol glycan anchor biosynthesis class C
1,01	FUT2	fucosyltransferase 2 (secretor status included)
1,01	RBBP5	retinoblastoma binding protein 5
1,01	SYAP1	synapse associated protein 1

1,01	SGTA	small glutamine-rich tetratricopeptide repeat (TPR)-containing, alpha
1,01	MAOA	monoamine oxidase A
1,01	SLC25A33	solute carrier family 25 (pyrimidine nucleotide carrier), member 33
1,01	ACOX3	acyl-CoA oxidase 3, pristanoyl
1,01	TRIM71	tripartite motif containing 71, E3 ubiquitin protein ligase
1,01	GPBP1	GC-rich promoter binding protein 1
1,01	ZSWIM6	zinc finger, SWIM-type containing 6
1,01	FAM71F2	family with sequence similarity 71, member F2
1,01	CTSE	cathepsin E
1,01	FBXO27	F-box protein 27
1,01	RCC2	regulator of chromosome condensation 2
1,01	NSUN4	NOP2/Sun domain family, member 4
1,01	BEX4	brain expressed X-linked 4
1,01	COLEC10	collectin sub-family member 10 (C-type lectin)
1,01	DYSF	dysferlin
1,01	CTPS2	CTP synthase 2
1,01	TNFAIP3	tumor necrosis factor, alpha-induced protein 3
1,01	GINS3	GINS complex subunit 3 (Psf3 homolog)
1,01	DNAJC3	DnaJ (Hsp40) homolog, subfamily C, member 3
1,01	IGLL1	immunoglobulin lambda-like polypeptide 1
1,01	ATP6V1G2-DDX39B	ATP6V1G2-DDX39B readthrough (NMD candidate)
1,01	RERG	RAS-like, estrogen-regulated, growth inhibitor
1,01	ADAP1	ArfGAP with dual PH domains 1
1,01	GBP3	guanylate binding protein 3
1,01	POP1	POP1 homolog, ribonuclease P/MRP subunit
1,01	C3orf79	chromosome 3 open reading frame 79
1,01	ADAMTS13	ADAM metallopeptidase with thrombospondin type 1 motif 13
1,01	RGAG4	retrotransposon gag domain containing 4
1,01	HPN-AS1	HPN antisense RNA 1
1,01	AIDA	axin interactor, dorsalization associated
1,01	GRIFIN	galectin-related inter-fiber protein
1,01	RPS19BP1	ribosomal protein S19 binding protein 1
1,01	DNAAF5	dynein, axonemal, assembly factor 5
1,01	GRK4	G protein-coupled receptor kinase 4
1,01	RFFL	ring finger and FYVE-like domain containing E3 ubiquitin protein ligase
1,01	BIVM-ERCC5	BIVM-ERCC5 readthrough
1,01	LOC729970; RP4-639F20.1	hCG2028352-like; novel transcript
1,01	DYRK1B	dual specificity tyrosine-(Y)-phosphorylation regulated kinase 1B
1,01	N4BP1	NEDD4 binding protein 1
1,01	CPB2	carboxypeptidase B2 (plasma)
1,01	RAB29	RAB29, member RAS oncogene family
1,01	TMEM167B	transmembrane protein 167B

1,01	LOC400927; CSNK1E	TPTE and PTEN homologous inositol lipid phosphatase pseudogene; Transcript Identified by AceView, Entrez Gene ID(s) 1454; 400927
1,01	ZNF699	zinc finger protein 699
1,01	SS18L1	synovial sarcoma translocation gene on chromosome 18-like 1
1,01	SGK3; C8orf44-SGK3	serum/glucocorticoid regulated kinase family, member 3; C8orf44-SGK3 readthrough
1,01	TBX4	T-box 4
1,01	DNAJC7	DnaJ (Hsp40) homolog, subfamily C, member 7
1,01	CABIN1	calcineurin binding protein 1
1,01	BOD1	biorientation of chromosomes in cell division 1
1,01	SLC25A48	solute carrier family 25, member 48
1,01	RPS17	ribosomal protein S17
1,01	SUPT20H	SPT20 homolog, SAGA complex component
1,01	MUT	methylmalonyl-CoA mutase
1,01	RPL32; SNORA7A	ribosomal protein L32; small nucleolar RNA, H/ACA box 7A
1,01	GTF2H2	general transcription factor IIH subunit 2
1,01	CRYBB1	crystallin beta B1
1,01	SH2D1B	SH2 domain containing 1B
1,01	ELAVL1	ELAV like RNA binding protein 1
1,01	ZNF705D	zinc finger protein 705D
1,01	GLI4	GLI family zinc finger 4
1,01	FAM69C	family with sequence similarity 69, member C
1,01	DHRS7B	dehydrogenase/reductase (SDR family) member 7B
1,01	MRAP2	melanocortin 2 receptor accessory protein 2
1,01	FHAD1	forkhead-associated (FHA) phosphopeptide binding domain 1
1,01	SFTPD	surfactant protein D
1,01	LYNX1	Ly6/neurotoxin 1
1,01	TES	testin LIM domain protein
1,01	IL15RA	interleukin 15 receptor, alpha
1,01	MCPH1	microcephalin 1
1,01	SENP5	SUMO1/sentrin specific peptidase 5
1,01	AK2	adenylate kinase 2
1,01	SOAT2	sterol O-acyltransferase 2
1,01	RPS27L	ribosomal protein S27-like
1,01	CRLF3	cytokine receptor-like factor 3
1,01	ARAP3	ArfGAP with RhoGAP domain, ankyrin repeat and PH domain 3
1,01	ABO	ABO blood group (transferase A, alpha 1-3-N-acetylgalactosaminyltransferase; transferase B, alpha 1-3-galactosyltransferase) [Source:HGNC Symbol;Acc:HGNC:79]
1,01	PPM1B	Memczak2013 ANTISENSE, coding, INTERNAL, intronic best transcript NM_001033556
1,01	RPL10; SNORA70	ribosomal protein L10; small nucleolar RNA, H/ACA box 70
1,01	FBL	fibrillarin

1,01	H2AFZ	H2A histone family, member Z
1,01	DYM	dymeclin
1,01	PKP4	Transcript Identified by AceView, Entrez Gene ID(s) 8502
1,01	GCHFR	GTP cyclohydrolase I feedback regulator
1,01	RAP1B	RAP1B, member of RAS oncogene family
1,01	BCAS2	breast carcinoma amplified sequence 2
1,01	ZNF532	zinc finger protein 532
1,01	LPAR2	lysophosphatidic acid receptor 2
1,01	RPL37A	ribosomal protein L37a
1,01	TCP11L2	t-complex 11, testis-specific-like 2
1,01	CCDC39	coiled-coil domain containing 39
1,01	YBX1	Y box binding protein 1
1,01	NMT1	N-myristoyltransferase 1
1,01	TCHP	trichoplein, keratin filament binding
1,01	RPL22	ribosomal protein L22
1,01	GPC5	glypican 5
1,01	RHOBTB1	Rho-related BTB domain containing 1
1,01	CPTP	ceramide-1-phosphate transfer protein
1,01	CDH24	cadherin 24, type 2
1,01	FAM234A; ARHGDIG	family with sequence similarity 234, member A; Rho GDP dissociation inhibitor (GDI) gamma
1,01	RGS7BP	regulator of G-protein signaling 7 binding protein
1,01	CSTB	cystatin B (stefin B)
1,01	CDC42	Transcript Identified by AceView, Entrez Gene ID(s) 998
1,01	FGR	FGR proto-oncogene, Src family tyrosine kinase
1,01	KDM1B	lysine (K)-specific demethylase 1B
1,01	NR2C1	nuclear receptor subfamily 2, group C, member 1
1,01	LAMTOR2	late endosomal/lysosomal adaptor, MAPK and MTOR activator 2
1,01	C11orf98; LBHD1	chromosome 11 open reading frame 98; LBH domain containing 1
1,01	HMGN1	high mobility group nucleosome binding domain 1
1,01	GEM	GTP binding protein overexpressed in skeletal muscle
1,01	QPRT	quinolinate phosphoribosyltransferase
1,01	PTK2	Transcript Identified by AceView, Entrez Gene ID(s) 5747
1,01	DYNC1LI2	dynein, cytoplasmic 1, light intermediate chain 2
1,01	OR4A5	olfactory receptor, family 4, subfamily A, member 5
1,01	BRF1	BRF1, RNA polymerase III transcription initiation factor 90 kDa subunit
1,01	UBASH3B	ubiquitin associated and SH3 domain containing B
1,01	SCGB2B2	secretoglobin, family 2B, member 2
1,01	GNAI1	guanine nucleotide binding protein (G protein), alpha inhibiting activity polypeptide 1
1,01	KDSR	3-ketodihydrosphingosine reductase
1,01	PDIA4	protein disulfide isomerase family A, member 4
1,01	TBRG1	transforming growth factor beta regulator 1

1,01	CNOT8	CCR4-NOT transcription complex subunit 8
1,01	LIN7B	lin-7 homolog B (C. elegans)
1,01	SPOCK3	sparc/osteonectin, cwcv and kazal-like domains proteoglycan (testican) 3
1,01	HIST1H2AC	histone cluster 1, H2ac
1,01	PSMD7	proteasome 26S subunit, non-ATPase 7
1,01	CDCA8	cell division cycle associated 8
1,01	ACSM3	acyl-CoA synthetase medium-chain family member 3
1,01	CNPY3	canopy FGF signaling regulator 3
1,01	ORMDL1	ORMDL sphingolipid biosynthesis regulator 1
1,01	TMEM176B	transmembrane protein 176B
1,01	RHOT2	ras homolog family member T2
1,01	SIGLEC6	sialic acid binding Ig-like lectin 6
1,01	DOK7	docking protein 7
1,01	FAM60A	family with sequence similarity 60, member A
1,01	MED15	mediator complex subunit 15
1,01	VEGFB	vascular endothelial growth factor B
1,01	CXCL6	chemokine (C-X-C motif) ligand 6
1,01	MCTS1	malignant T-cell amplified sequence 1
1,01	CTSO	cathepsin O
1,01	SPC24	SPC24, NDC80 kinetochore complex component
1,01	SF3A2	splicing factor 3a subunit 2
1,01	OAZ1	Memczak2013 ALT_ACCEPTOR, ALT_DONOR, coding, INTERNAL, intronic best transcript NM_004152
1,01	RPL21	Homo sapiens ribosomal protein L21, mRNA (cDNA clone MGC:2150 IMAGE:3543702), complete cds.; Homo sapiens ribosomal protein L21, mRNA (cDNA clone MGC:4136 IMAGE:2964441), complete cds.; Homo sapiens ribosomal protein L21, mRNA (cDNA clone MGC:71252 IMAGE:6084268), complete cds.; Homo sapiens ribosomal protein L21, mRNA (cDNA clone MGC:88165 IMAGE:4281405), complete cds.; Homo sapiens ribosomal protein L21, mRNA (cDNA clone MGC:88326 IMAGE:4248655), complete cds.; Homo sapiens ribosomal protein L21, mRNA (cDNA clone MGC:88334 IMAGE:3931616), complete cds.; Homo sapiens ribosomal protein L21, mRNA (cDNA clone MGC:88581 IMAGE:4901492), complete cds.; Homo sapiens ribosomal protein L21, mRNA (cDNA clone MGC:104275 IMAGE:6724053), complete cds.
1,01	TMPO	thymopoietin
1,01	FOSB	FBJ murine osteosarcoma viral oncogene homolog B
1,01	CNGA3	cyclic nucleotide gated channel alpha 3
1,01	PHF6	PHD finger protein 6
1,01	FUT5	fucosyltransferase 5 (alpha (1,3) fucosyltransferase)
1,01	ABCC12	ATP binding cassette subfamily C member 12
1,01	LIN52	lin-52 DREAM MuvB core complex component
1,01	POLR2J	polymerase (RNA) II (DNA directed) polypeptide J, 13.3kDa
1,01	ZBED9	zinc finger, BED-type containing 9

1,01	HMGB3	high mobility group box 3
1,01	BCL2L14	BCL2-like 14 (apoptosis facilitator)
1,01	PFDN5	prefoldin subunit 5
1,01	TCP1; SNORA20; SNORA29	t-complex 1; small nucleolar RNA, H/ACA box 20; small nucleolar RNA, H/ACA box 29
1,01	ENTPD5	ectonucleoside triphosphate diphosphohydrolase 5
1,01	SLC27A3	solute carrier family 27 (fatty acid transporter), member 3
1,01	SIRT7	sirtuin 7
1,01	NKX6-2	NK6 homeobox 2
1,01	SPTBN5	spectrin, beta, non-erythrocytic 5
1,01	PCF11	PCF11 cleavage and polyadenylation factor subunit
1,01	TFCP2	transcription factor CP2
1,01	DHX40	DEAH (Asp-Glu-Ala-His) box polypeptide 40
1,01	ZNF552	zinc finger protein 552
1,01	RPL7L1	ribosomal protein L7-like 1
1,01	SERINC2	serine incorporator 2
1,01	INPP5D	inositol polyphosphate-5-phosphatase D
1,01	RPL12	ribosomal protein L12
1,01	F13B	coagulation factor XIII, B polypeptide
1,01	TLR4	toll-like receptor 4
1,01	SPON1	spondin 1, extracellular matrix protein
1,01	ZNF90	zinc finger protein 90
1,01	ADCYAP1	adenylate cyclase activating polypeptide 1 (pituitary)
1,01	LRRC43	leucine rich repeat containing 43
1,01	QPCTL	glutaminyl-peptide cyclotransferase-like
1,01	RNF125	ring finger protein 125, E3 ubiquitin protein ligase
1,01	CLIP4	CAP-GLY domain containing linker protein family, member 4
1,01	LOC100131315; RP11-758N13.1	uncharacterized LOC100131315; novel transcript
1,01	SEC14L1; SCARNA16; SNHG20; MIR6516	SEC14-like lipid binding 1; small Cajal body-specific RNA 16; small nucleolar RNA host gene 20; microRNA 6516
1,01	METTL6	methyltransferase like 6
1,01	IGFL1	IGF like family member 1
1,01	DHX30	DEAH (Asp-Glu-Ala-His) box helicase 30
1,01	ADAMTS13	ADAM metallopeptidase with thrombospondin type 1 motif 13
1,01	RAPGEF3	Rap guanine nucleotide exchange factor 3
1,01	KRTAP19-5	keratin associated protein 19-5
1,01	CYP4F3	cytochrome P450, family 4, subfamily F, polypeptide 3
1,01	C12orf75	chromosome 12 open reading frame 75
1,01	CASP5	caspase 5
1,01	SLC39A2	solute carrier family 39 (zinc transporter), member 2
1,01	IRX3	iroquois homeobox 3
1,01	OBP2B	odorant binding protein 2B

1,01	LMBRD2	LMBR1 domain containing 2
1,01	FAM114A1	family with sequence similarity 114, member A1
1,01	RIPK2	receptor-interacting serine-threonine kinase 2
1,01	RBMY1E; RBMY1B	RNA binding motif protein, Y-linked, family 1, member E; RNA binding motif protein, Y-linked, family 1, member B
1,01	RBMY1E; RBMY1D; RBMY1B	RNA binding motif protein, Y-linked, family 1, member E; RNA binding motif protein, Y-linked, family 1, member D; RNA binding motif protein, Y-linked, family 1, member B
1,01	EIF4A1; SNORD10; SNORA67; SNORA48	eukaryotic translation initiation factor 4A1; small nucleolar RNA, C/D box 10; small nucleolar RNA, H/ACA box 67; small nucleolar RNA, H/ACA box 48
1,01	ANKRD63	ankyrin repeat domain 63
1,01	PEF1	penta-EF-hand domain containing 1
1,01	KLK3	kallikrein related peptidase 3
1,01	INPP5D	inositol polyphosphate-5-phosphatase D
1,01	FAM3D	family with sequence similarity 3, member D
1,01	GRHL3	grainyhead-like transcription factor 3
1,01	TMOD2	tropomodulin 2 (neuronal)
1,01	TMPRSS4	transmembrane protease, serine 4
1,01	PCDH11X	Salzman2013 ANTISENSE, coding, INTERNAL, intronic best transcript NM_032968
1,01	ARL13B	ADP-ribosylation factor like GTPase 13B
1,01	GPATCH2L	G-patch domain containing 2 like
1,01	COX15	cytochrome c oxidase assembly homolog 15 (yeast)
1,01	KPRP	keratinocyte proline-rich protein
1,01	BHMG1	basic helix-loop-helix and HMG box domain containing 1
1,01	TIMM50	translocase of inner mitochondrial membrane 50 homolog (S. cerevisiae)
1,01	CD9	CD9 molecule
1,01	SEZ6	Memczak2013 ANTISENSE, CDS, coding, INTERNAL, intronic best transcript NM_178860
1,01	UBE2NL	ubiquitin conjugating enzyme E2N-like (gene/pseudogene)
1,01	BLCAP	bladder cancer associated protein
1,01	PAGE2	P antigen family, member 2 (prostate associated)
1,01	GOLPH3L	golgi phosphoprotein 3-like
1,01	MLF1	myeloid leukemia factor 1
1,01	SSH1	slingshot protein phosphatase 1
1,01	GPAT3	glycerol-3-phosphate acyltransferase 3
1,01	METAP1D	methionyl aminopeptidase type 1D (mitochondrial)
1,01	AC007292.7; MPND	Transcript Identified by AceView, Entrez Gene ID(s) 84954; novel transcript, antisense to STAP2
1,01	ZFX	zinc finger protein, X-linked
1,01	TUT1	terminal uridylyl transferase 1, U6 snRNA-specific
1,01	JAG2	jagged 2

1,01	TMSB15B; RP11-722G7.1	Homo sapiens thymosin beta 15B (TMSB15B), mRNA.; Thymosin beta-15B [Source:UniProtKB/Swiss-Prot;Acc:P0CG35]
1,01	SMURF1	SMAD specific E3 ubiquitin protein ligase 1
1,01	CAMK2N1	calcium/calmodulin-dependent protein kinase II inhibitor 1
1,01	ARL2	ADP-ribosylation factor like GTPase 2
1,01	NNAT	neuronatin
1,01	SAG	S-antigen; retina and pineal gland (arrestin)
1,01	C19orf33	chromosome 19 open reading frame 33
1,01	LRIG1	
1,01	TRNT1	tRNA nucleotidyl transferase, CCA-adding, 1
1,01	RPRD2	regulation of nuclear pre-mRNA domain containing 2
1,01	STKLD1	serine/threonine kinase-like domain containing 1 [Source:HGNC Symbol;Acc:HGNC:28669]
1,01	SAMD11	sterile alpha motif domain containing 11
1,01	PSMD3	proteasome 26S subunit, non-ATPase 3
1,01	TRIM60	tripartite motif containing 60
1,01	EZH2	enhancer of zeste 2 polycomb repressive complex 2 subunit
1,01	AAK1	AP2 associated kinase 1
1,01	DDB1	damage-specific DNA binding protein 1
1,01	CFL1	cofilin 1 (non-muscle)
1,01	PPME1	protein phosphatase methylesterase 1
1,01	RCOR3	REST corepressor 3
1,01	POLDIP2	polymerase (DNA-directed), delta interacting protein 2
1,01	ZNF780A	zinc finger protein 780A
1,01	TM4SF1	transmembrane 4 L six family member 1
1,01	ATP8B1	Salzman2013 ANTISENSE, CDS, coding, INTERNAL, intronic, OVCODE, OVERLAPTX, OVEXON, UTR5 best transcript NM_005603
1,01	FZD8; MIR4683	frizzled class receptor 8; microRNA 4683
1,01	NDUFV3	NADH dehydrogenase (ubiquinone) flavoprotein 3, 10kDa
1,01	DCAF12	DDB1 and CUL4 associated factor 12
1,01	MYO15B	Memczak2013 ANTISENSE, INTERNAL, ncRNA best transcript NR_003587
1,01	SMIM10L2A	small integral membrane protein 10 like 2A
1,01	SLC18B1	solute carrier family 18, subfamily B, member 1
1,01	LRRC16B	leucine rich repeat containing 16B
1,01	SLC17A6	solute carrier family 17 (vesicular glutamate transporter), member 6
1,01	ECD	ecdysoneless homolog (Drosophila)
1,01	NUDT1	nudix hydrolase 1
1,01	GGPS1	geranylgeranyl diphosphate synthase 1
1,01	RPSA; SNORA62; SNORA6	ribosomal protein SA; small nucleolar RNA, H/ACA box 62; small nucleolar RNA, H/ACA box 6
1,01	C10orf95	chromosome 10 open reading frame 95

1,01	STMN4	stathmin-like 4
1,01	C6orf132	chromosome 6 open reading frame 132
1,01	DHPS	deoxyhypusine synthase
1,01	EDAR	ectodysplasin A receptor
1,01	SP7	Sp7 transcription factor
1,01	WDR53	WD repeat domain 53
1,01	XKR8	X-linked Kx blood group related 8
1,01	UQCRC1	ubiquinol-cytochrome c reductase core protein I
1,01	FUBP1	far upstream element (FUSE) binding protein 1
1,01	PMS1	PMS1 homolog 1, mismatch repair system component
1,01	LRRC47	leucine rich repeat containing 47
1,01	HIST1H2AL; HIST1H2BN	histone cluster 1, H2al; histone cluster 1, H2bn
1,01	HPN	hepsin
1,01	ZNF646	Memczak2013 ANTISENSE, coding, INTERNAL, intronic best transcript NM_014699
1,01	TMEM19	transmembrane protein 19
1,01	PLPPR3	phospholipid phosphatase related 3
1,01	STT3B	STT3B, subunit of the oligosaccharyltransferase complex (catalytic)
1,01	LIG3	ligase III, DNA, ATP-dependent
1,01	HIST1H2AK	histone cluster 1, H2ak
1,01	MINOS1-NBL1	MINOS1-NBL1 readthrough
1,01	SLC2A9	solute carrier family 2 (facilitated glucose transporter), member 9
1,01	HSD3B7	hydroxy-delta-5-steroid dehydrogenase, 3 beta- and steroid delta-isomerase 7
1,01	MLXIP	MLX interacting protein
1,01	LYPD8	LY6/PLAUR domain containing 8
1,01	PDRG1	p53 and DNA-damage regulated 1
1,01	BCL11B	B-cell CLL/lymphoma 11B (zinc finger protein)
1,01	RARS	arginyl-tRNA synthetase
1,01	ZNRF3	zinc and ring finger 3
1,01	PACS1	phosphofurin acidic cluster sorting protein 1
1,01	PTF1A	pancreas specific transcription factor, 1a
1,01	EEA1	early endosome antigen 1
1,01	FAM98C	family with sequence similarity 98, member C
1,01	FBN3	fibrillin 3
1,01	RNF146	ring finger protein 146
1,01	THEM5	thioesterase superfamily member 5
1,01	ARPC2	actin related protein 2/3 complex subunit 2
1,01	CD24	CD24 molecule

1,01	PGAM1	Homo sapiens phosphoglycerate mutase 1 (brain), mRNA (cDNA clone MGC:8462 IMAGE:2821567), complete cds.; Homo sapiens phosphoglycerate mutase 1 (brain), mRNA (cDNA clone MGC:19732 IMAGE:3604026), complete cds.; Homo sapiens phosphoglycerate mutase 1 (brain), mRNA (cDNA clone MGC:15086 IMAGE:4299008), complete cds.; Homo sapiens phosphoglycerate mutase 1 (brain), mRNA (cDNA clone MGC:61519 IMAGE:5787678), complete cds.; Homo sapiens phosphoglycerate mutase 1 (brain), mRNA (cDNA clone MGC:71323 IMAGE:6584466), complete cds.; Homo sapiens phosphoglycerate mutase 1 (brain), mRNA (cDNA clone MGC:87445 IMAGE:5262935), complete cds.; Homo sapiens phosphoglycerate mutase 1 (brain), mRNA (cDNA clone MGC:88744 IMAGE:6297173), complete cds.; Homo sapiens phosphoglycerate mutase 1 (brain), mRNA (cDNA clone MGC:189702 IMAGE:8862699), complete cds.
1,01	MTIF2	mitochondrial translational initiation factor 2
1,01	ZNF679	zinc finger protein 679
1,01	NOXA1	NADPH oxidase activator 1
1,01	JAZF1	JAZF zinc finger 1
1,01	SLC25A15; MIR621	solute carrier family 25 (mitochondrial carrier; ornithine transporter) member 15; microRNA 621
1,01	ICE2	interactor of little elongation complex ELL subunit 2
1,01	NCAPH	non-SMC condensin I complex subunit H
1,01	CCT5	chaperonin containing TCP1, subunit 5 (epsilon)
1,01	CELF3	CUGBP, Elav-like family member 3
1,01	KRT27	keratin 27, type I
1,01	THOC7	THO complex 7
1,01	HIST1H4G	histone cluster 1, H4g
1,01	NXF2B; NXF2	nuclear RNA export factor 2B; nuclear RNA export factor 2
1,01	RSPH9	radial spoke head 9 homolog (Chlamydomonas)
1,01	SET	SET nuclear proto-oncogene
1,01	OGG1	8-oxoguanine DNA glycosylase
1,01	SPATA31D3	SPATA31 subfamily D, member 3
1,01	NKRF	NFKB repressing factor
1,01	RPL11	ribosomal protein L11
1,01	PCOLCE2	procollagen C-endopeptidase enhancer 2
1,01	PCLO	piccolo presynaptic cytomatrix protein
1,01	OPALIN	oligodendrocytic myelin paranodal and inner loop protein
1,01	sep-01	septin 1
1,01	PAFAH1B2	platelet-activating factor acetylhydrolase 1b, catalytic subunit 2 (30kDa)
1,01	CHRM3	cholinergic receptor, muscarinic 3
1,01	RB1CC1	RB1-inducible coiled-coil 1
1,01	TMEM243	transmembrane protein 243, mitochondrial
1,01	SAMD12	sterile alpha motif domain containing 12

1,01	ARHGEF25	Rho guanine nucleotide exchange factor 25
1,01	ARSB	arylsulfatase B
1,01	TRIM41	tripartite motif containing 41
1,01	BCLAF1	BCL2-associated transcription factor 1
1,01	POT1	protection of telomeres 1
1,01	ID2	inhibitor of DNA binding 2, dominant negative helix-loop-helix protein
1,01	COL9A1	collagen, type IX, alpha 1
1,01	SEC23A	Sec23 homolog A, COPII coat complex component
1,01	DLGAP4	discs, large (Drosophila) homolog-associated protein 4
1,01	CHID1	chitinase domain containing 1
1,01	KISS1	KiSS-1 metastasis-suppressor
1,01	MBL2	mannose-binding lectin (protein C) 2, soluble
1,01	TMEM39A	transmembrane protein 39A
1,01	IL19	interleukin 19
1,01	MAPK14	mitogen-activated protein kinase 14
1,01	CALM2	calmodulin 2 (phosphorylase kinase, delta)
1,01	HNRNPU	heterogeneous nuclear ribonucleoprotein U (scaffold attachment factor A)
1,01	MIOS	missing oocyte, meiosis regulator, homolog (Drosophila)
1,01	TSR2	TSR2, 20S rRNA accumulation, homolog (S. cerevisiae)
1,01	ATP6V0D1	ATPase, H+ transporting, lysosomal 38kDa, V0 subunit d1
1,01	SLC5A2	solute carrier family 5 (sodium/glucose cotransporter), member 2
1,01	CTR9	CTR9 homolog, Paf1/RNA polymerase II complex component
1,01	PHKA1	phosphorylase kinase, alpha 1 (muscle)
1,01	ZNHIT1	zinc finger, HIT-type containing 1
1,01	EIF4E2	eukaryotic translation initiation factor 4E family member 2
1,01	ZNF540; ZNF571-AS1	zinc finger protein 540; ZNF571 antisense RNA 1
1,01	UNKL	unkempt family zinc finger-like
1,01	ZNF598	zinc finger protein 598
1,01	CCDC182	coiled-coil domain containing 182
1,01	TPP1	tripeptidyl peptidase I
1,01	HSBP1L1	heat shock factor binding protein 1-like 1
1,01	OR7G2	olfactory receptor, family 7, subfamily G, member 2
1,01	MTMR9	myotubularin related protein 9
1,01	JAGN1	jagunal homolog 1
1,01	SOHLH1	spermatogenesis and oogenesis specific basic helix-loop-helix 1
1,01	RGP1	RGP1 homolog, RAB6A GEF complex partner 1
1,01	MAF1	MAF1 homolog, negative regulator of RNA polymerase III
1,01	GOT1	glutamic-oxaloacetic transaminase 1, soluble

1,01	ASAH1	N-acylsphingosine amidohydrolase (acid ceramidase) 1
1,01	FAM229B	family with sequence similarity 229, member B
1,01	KAT6B	K(lysine) acetyltransferase 6B
1,01	HSFX2; HSFX1	heat shock transcription factor family, X-linked 2; heat shock transcription factor family, X-linked 1
1,01	HSPA2	heat shock 70kDa protein 2
1,01	TNFAIP6	tumor necrosis factor, alpha-induced protein 6
1,01	HIST4H4	histone cluster 4, H4
1,01	GPR132	G protein-coupled receptor 132
1,01	TMEM158	transmembrane protein 158 (gene/pseudogene)
1,01	CSH2	chorionic somatomammotropin hormone 2
1,01	HOXC10	homeobox C10
1,01	GID4	GID complex subunit 4 homolog
1,01	POC1B; POC1B-GALNT4; GALNT4	POC1 centriolar protein B; POC1B-GALNT4 readthrough; polypeptide N-acetylgalactosaminyltransferase 4
1,01	HIST1H3F	histone cluster 1, H3f
1,01	DHX9	DEAH (Asp-Glu-Ala-His) box helicase 9
1,01	CHML	choroideremia-like (Rab escort protein 2)
1,01	RTKN	rhotekin
1,01	SMIM11A	small integral membrane protein 11A
1,01	MFSD10	major facilitator superfamily domain containing 10
1,01	DGCR6	DiGeorge syndrome critical region gene 6
1,01	VBP1	von Hippel-Lindau binding protein 1
1,01	ITFG2	integrin alpha FG-GAP repeat containing 2
1,01	NAA50	N(alpha)-acetyltransferase 50, NatE catalytic subunit
1,01	TMEM230	transmembrane protein 230
1,01	TP53TG3B; TP53TG3; TP53TG3C	TP53 target 3B; TP53 target 3; TP53 target 3C
1,01	NRDC; MIR761	nardilysin convertase; microRNA 761
1,01	SLAMF8	SLAM family member 8
1,01	ago-01	argonaute RISC catalytic component 1
1,01	ENO1	enolase 1, (alpha)
1,01	C15orf53	chromosome 15 open reading frame 53
1,01	SIVA1	SIVA1, apoptosis-inducing factor
1,01	TGIF2-C20orf24	TGIF2-C20orf24 readthrough
1,01	SLC39A1	solute carrier family 39 (zinc transporter), member 1
1,01	TMPRSS12	transmembrane (C-terminal) protease, serine 12
1,01	VCL	vinculin
1,01	DCBLD2	discoidin, CUB and LCCL domain containing 2
1,01	MAGEF1	MAGE family member F1
1,01	SLC35A5	solute carrier family 35, member A5
1,01	GJB2	gap junction protein beta 2
1,01	XRN2	5-3 exoribonuclease 2
1,01	C8orf22	chromosome 8 open reading frame 22
1,01	NPC1L1	NPC1-like 1
1,01	TNFSF12-TNFSF13	TNFSF12-TNFSF13 readthrough

1,01	ABCD3	ATP binding cassette subfamily D member 3
1,01	TMEM14B	transmembrane protein 14B
1,01	INPP5D	inositol polyphosphate-5-phosphatase D
1,01	UCK2; MIR3658	uridine-cytidine kinase 2; microRNA 3658
1,01	BORCS7-ASMT	BORCS7-ASMT readthrough (NMD candidate)
1,01	SLC35D3	solute carrier family 35, member D3
1,01	SNRNP40	small nuclear ribonucleoprotein, U5 40kDa subunit
1,01	RPL14	ribosomal protein L14
1,01	RPS6KA1	ribosomal protein S6 kinase, 90kDa, polypeptide 1
1,01	PPOX	protoporphyrinogen oxidase
1,01	GEMIN2	gem nuclear organelle associated protein 2
1,01	ESPL1	extra spindle pole bodies like 1, separase
1,01	GUCD1	guanylyl cyclase domain containing 1
1,01	FBXO48	F-box protein 48
1,01	OR2L3	olfactory receptor, family 2, subfamily L, member 3
1,01	MC2R	melanocortin 2 receptor (adrenocorticotropic hormone)
1,01	IQCH	IQ motif containing H
1,01	GAS2L3	growth arrest-specific 2 like 3
1,01	GYPE	glycophorin E (MNS blood group)
1,01	MED30	mediator complex subunit 30
1,01	TMEM239; C20orf141	transmembrane protein 239; chromosome 20 open reading frame 141
1,01	ADAM30	ADAM metallopeptidase domain 30
1,01	YEATS4	YEATS domain containing 4
1,01	CUL2	cullin 2
1,01	EXOC1	exocyst complex component 1
1,01	GRIA4	glutamate receptor, ionotropic, AMPA 4
1,01	FAM71E2	family with sequence similarity 71, member E2
1,01	SF3B2	splicing factor 3b subunit 2
1,01	FAM151B	family with sequence similarity 151, member B
1,01	PHKG2	phosphorylase kinase, gamma 2 (testis)
1,01	PIP5K1B	Transcript Identified by AceView, Entrez Gene ID(s) 8395
1,01	PLAU	plasminogen activator, urokinase
1,01	TRIM50	tripartite motif containing 50
1,01	SNAPC3	small nuclear RNA activating complex polypeptide 3
1,01	BAP1	BRCA1 associated protein 1
1,01	IRX1	iroquois homeobox 1
1,01	NRAP	nebulin-related anchoring protein
1,01	INPP1	inositol polyphosphate-1-phosphatase
1,01	ERCC5	excision repair cross-complementation group 5
1,01	NMNAT1	nicotinamide nucleotide adenylyltransferase 1
1,01	PRR11	proline rich 11
1,01	DEFA6	defensin, alpha 6, Paneth cell-specific
1,01	ZNF397	zinc finger protein 397
1,01	MIER2	mesoderm induction early response 1, family member 2
1,01	SMR3A	submaxillary gland androgen regulated protein 3A
1,01	PRKCB	protein kinase C, beta

1,01	ZC2HC1B	zinc finger, C2HC-type containing 1B
1,01	RELL2	RELT-like 2
1,01	CPSF6	cleavage and polyadenylation specific factor 6
1,01	TBC1D3G	TBC1 domain family, member 3G
1,01	FAM126A	family with sequence similarity 126, member A
1,01	STAM	signal transducing adaptor molecule (SH3 domain and ITAM motif) 1
1,01	MYBL1	v-myb avian myeloblastosis viral oncogene homolog-like 1
1,01	FAM53A	family with sequence similarity 53, member A
1,01	PSTPIP1	proline-serine-threonine phosphatase interacting protein 1
1,01	FXYD3; MIR6887	FXYD domain containing ion transport regulator 3; microRNA 6887
1,01	ZFP90	ZFP90 zinc finger protein
1,01	RASA3	RAS p21 protein activator 3
1,01	INTS2	integrator complex subunit 2
1,01	LRCH3	leucine-rich repeats and calponin homology (CH) domain containing 3
1,01	MAP4K5	mitogen-activated protein kinase kinase kinase kinase 5
1,01	CALCR	calcitonin receptor
1,01	FBL	fibrillarin
1,01	ZSWIM4	Transcript Identified by AceView, Entrez Gene ID(s) 65249
1,01	SMC6	Transcript Identified by AceView, Entrez Gene ID(s) 79677
1,01	JUP	junction plakoglobin
1,01	GNA13	guanine nucleotide binding protein (G protein), alpha 13
1,01	PHF14	PHD finger protein 14
1,01	MPHOSPH6	M-phase phosphoprotein 6
1,01	MBD4	methyl-CpG binding domain 4 DNA glycosylase
1,01	TCAIM	T cell activation inhibitor, mitochondrial
1,01	PXMP2	peroxisomal membrane protein 2
1,01	SMAD7	SMAD family member 7
1,01	USF3	upstream transcription factor family member 3
1,01	GRAP	GRB2-related adaptor protein
1,01	PTH2R	parathyroid hormone 2 receptor
1,01	VAPB	VAMP (vesicle-associated membrane protein)-associated protein B and C
1,01	TNS2	tensin 2
1,01	SLCO5A1	solute carrier organic anion transporter family, member 5A1
1,01	NAA15	N(alpha)-acetyltransferase 15, NatA auxiliary subunit
1,01	RARRES3	retinoic acid receptor responder (tazarotene induced) 3
1,01	CAP2	CAP, adenylate cyclase-associated protein, 2 (yeast)
1,01	MYO15B	myosin XVB
1,01	SYNCRIP	synaptotagmin binding, cytoplasmic RNA interacting protein

1,01	ADGRL3	adhesion G protein-coupled receptor L3
1,01	POLR2J3	polymerase (RNA) II (DNA directed) polypeptide J3
1,01	BRD8	bromodomain containing 8
1,01	SP140	SP140 nuclear body protein
1,01	BRD1	bromodomain containing 1
1,01	KLHL26	kelch-like family member 26
1,01	NDST1	N-deacetylase/N-sulfotransferase (heparan glucosaminyl) 1
1,01	HMP19	HMP19 protein; Neuron-specific protein family member 2 [Source:UniProtKB/Swiss-Prot;Acc:Q9Y328]; Transcript Identified by AceView, Entrez Gene ID(s) 51617
1,01	HES3	hes family bHLH transcription factor 3
1,01	TMEM134	transmembrane protein 134
1,01	SLC14A2	solute carrier family 14 (urea transporter), member 2
1,01	ACER3	alkaline ceramidase 3
1,01	ALCAM	activated leukocyte cell adhesion molecule
1,01	ATG3	autophagy related 3
1,01	ASAP1; ASAP1-IT2	ArfGAP with SH3 domain, ankyrin repeat and PH domain 1; ASAP1 intronic transcript 2
1,01	LDLR; MIR6886	low density lipoprotein receptor; microRNA 6886
1,01	TRAPPC4	trafficking protein particle complex 4
1,01	IL18R1	interleukin 18 receptor 1
1,01	NDUFA2	NADH dehydrogenase (ubiquinone) 1 alpha subcomplex, 2, 8kDa
1,01	ACAA1	acetyl-CoA acyltransferase 1
1,01	SCN1A	sodium channel, voltage gated, type I alpha subunit
1,01	LDHA	lactate dehydrogenase A
1,01	TOB2	transducer of ERBB2, 2
1,01	CFAP77	cilia and flagella associated protein 77
1,01	IWS1	IWS1 homolog (S. cerevisiae)
1,01	CCDC43	coiled-coil domain containing 43
1,01	RPS26	ribosomal protein S26
1,01	GOLGA8DP	golgin A8 family, member D, pseudogene
1,01	MMP19	matrix metallopeptidase 19
1,01	CYB5R4	cytochrome b5 reductase 4
1,01	UCHL3	ubiquitin C-terminal hydrolase L3
1,01	KCNQ5	Jeck2013 ALT_ACCEPTOR, ALT_DONOR, coding, INTERNAL, intronic best transcript NM_001160133
1,01	CREG1	cellular repressor of E1A-stimulated genes 1
1,01	TNFRSF11A	tumor necrosis factor receptor superfamily, member 11a, NFKB activator
1,01	TNPO3	transportin 3
1,01	MESP2	mesoderm posterior bHLH transcription factor 2
1,01	GSTP1	glutathione S-transferase pi 1
1,01	ADRB3	adrenoceptor beta 3
1,01	ZNHIT6	zinc finger, HIT-type containing 6
1,01	RAB3IL1	RAB3A interacting protein (rabin3)-like 1
1,01	SATL1	spermidine/spermine N1-acetyl transferase-like 1
1,01	VDAC3	voltage-dependent anion channel 3

1,01	SLC22A15	solute carrier family 22, member 15
1,01	HNRNPCL3; HNRNPCL4	heterogeneous nuclear ribonucleoprotein C-like 3; heterogeneous nuclear ribonucleoprotein C-like 4
1,01	HSP90AB1	heat shock protein 90kDa alpha (cytosolic), class B member 1
1,01	ENPP1	ectonucleotide pyrophosphatase/phosphodiesterase 1
1,01	RCOR1	REST corepressor 1
1,01	USP17L22; USP17L20	ubiquitin specific peptidase 17-like family member 22; ubiquitin specific peptidase 17-like family member 20
1,01	RAD50	RAD50 homolog, double strand break repair protein
1,01	EXOSC2	exosome component 2
1,01	SYNGR2	synaptogyrin 2
1,01	ST8SIA1	ST8 alpha-N-acetyl-neuraminide alpha-2,8-sialyltransferase 1
1,01	MRRF	mitochondrial ribosome recycling factor
1,01	XRCC3	X-ray repair complementing defective repair in Chinese hamster cells 3
1,01	LSMEM2	leucine-rich single-pass membrane protein 2
1,01	LOC100421561	family with sequence similarity 133, member A pseudogene
1,01	ATPAF2	ATP synthase mitochondrial F1 complex assembly factor 2
1,01	SNAP29	synaptosome associated protein 29kDa
1,01	LSM12	LSM12 homolog
1,01	RAB44	RAB44, member RAS oncogene family
1,01	FRG1BP	FSHD region gene 1 family member B, pseudogene
1,01	ZNF454	zinc finger protein 454
1,01	FAM102A	family with sequence similarity 102, member A
1,01	AP1M1	Memczak2013 ALT_ACCEPTOR, ALT_DONOR, coding, INTERNAL, intronic best transcript NM_032493
1,01	MYLIP; MIR4639	myosin regulatory light chain interacting protein; microRNA 4639
1,01	ATP5B	ATP synthase, H+ transporting, mitochondrial F1 complex, beta polypeptide
1,01	DMRT3	doublesex and mab-3 related transcription factor 3
1,01	STYX	serine/threonine/tyrosine interacting protein
1,01	ADGRE1	adhesion G protein-coupled receptor E1
1,01	PDK2	pyruvate dehydrogenase kinase, isozyme 2
1,01	ZXDB	zinc finger, X-linked, duplicated B
1,01	AIFM1	apoptosis-inducing factor, mitochondrion-associated, 1
1,01	LMNTD1	lamin tail domain containing 1
1,01	CNOT10	CCR4-NOT transcription complex subunit 10
1,01	DCANP1	dendritic cell-associated nuclear protein
1,01	PDCD4; MIR4680	programmed cell death 4 (neoplastic transformation inhibitor); microRNA 4680
1,01	CCNH	cyclin H
1,01	SAA2; SAA2-SAA4; SAA4	serum amyloid A2; SAA2-SAA4 readthrough; serum amyloid A4, constitutive

1,01	FOXH1	forkhead box H1
1,01	CD1A	CD1a molecule
1,01	BLK	Transcript Identified by AceView, Entrez Gene ID(s) 640
1,01	ASPH	aspartate beta-hydroxylase
1,01	CNIH3; RP11-449J1.1	Transcript Identified by AceView, Entrez Gene ID(s) 149111; novel transcript
1,01	SLC16A1	solute carrier family 16 (monocarboxylate transporter), member 1
1,01	GRM2	glutamate receptor, metabotropic 2
1,01	NDUFA10	NADH dehydrogenase (ubiquinone) 1 alpha subcomplex, 10, 42kDa
1,01	TREM1	triggering receptor expressed on myeloid cells 1
1,01	TCTA	T-cell leukemia translocation altered
1,01	LRP10	LDL receptor related protein 10
1,01	ZNF865	zinc finger protein 865
1,01	PRF1	perforin 1 (pore forming protein)
1,01	LRP6	LDL receptor related protein 6
1,01	RPS12	ribosomal protein S12
1,01	NME4	NME/NM23 nucleoside diphosphate kinase 4
1,01	C10orf55	chromosome 10 open reading frame 55
1,01	MXD4; MIR4800	MAX dimerization protein 4; microRNA 4800
1,01	P2RY6	pyrimidinergic receptor P2Y, G-protein coupled, 6
1,01	MKRN2	makorin ring finger protein 2
1,01	TJP1	tight junction protein 1
1,01	C1orf64	chromosome 1 open reading frame 64
1,01	SLC28A1	solute carrier family 28 (concentrative nucleoside transporter), member 1
1,01	TOMM40	translocase of outer mitochondrial membrane 40 homolog (yeast)
1,01	ABCC5	ATP binding cassette subfamily C member 5
1,01	TADA3	transcriptional adaptor 3
1,01	PGC	progastricsin (pepsinogen C)
1,01	GPRIN3	GPRIN family member 3
1,01	DALRD3	DALR anticodon binding domain containing 3
1,01	C2CD4C	C2 calcium-dependent domain containing 4C
1,01	ATP5L	ATP synthase, H+ transporting, mitochondrial Fo complex subunit G
1,01	PPP1R12B	protein phosphatase 1, regulatory subunit 12B
1,01	REXO4	REX4 homolog, 3-5 exonuclease
1,01	KIN	Kin17 DNA and RNA binding protein
1,01	POLR1A	polymerase (RNA) I polypeptide A
1,01	NXPH3	neurexophilin 3
1,01	WBSCR22	Williams Beuren syndrome chromosome region 22
1,01	IL6ST	interleukin 6 signal transducer
1,01	TACSTD2	tumor-associated calcium signal transducer 2
1,01	PRKD2	protein kinase D2
1,01	APMAP	adipocyte plasma membrane associated protein
1,01	BANK1	B-cell scaffold protein with ankyrin repeats 1
1,01	LARS	leucyl-tRNA synthetase

1,01	PDGFB	platelet-derived growth factor beta polypeptide
1,01	NRG2	neuregulin 2
1,01	RIMKLB	ribosomal modification protein rimK-like family member B
1,01	DHX15	DEAH (Asp-Glu-Ala-His) box helicase 15
1,01	RP11-325B23.2; OR5H8	novel transcript; olfactory receptor, family 5, subfamily H, member 8 (gene/pseudogene) [Source:HGNC Symbol;Acc:HGNC:14773]
1,01	TMCO5A	transmembrane and coiled-coil domains 5A
1,01	RAB5A	RAB5A, member RAS oncogene family
1,01	ZNF665	zinc finger protein 665
1,01	CBLN1	cerebellin 1 precursor
1,01	TGOLN2	trans-golgi network protein 2
1,01	OSGEP	O-sialoglycoprotein endopeptidase
1,01	NPC1	Niemann-Pick disease, type C1
1,01	ABCB5	ATP binding cassette subfamily B member 5
1,01	TRAPPC2	trafficking protein particle complex 2
1,01	PIK3R6	phosphoinositide-3-kinase, regulatory subunit 6
1,01	C7orf71	chromosome 7 open reading frame 71
1,01	TUBG1	tubulin, gamma 1
1,01	OVCH1	ovochymase 1
1,01	ALG10	ALG10, alpha-1,2-glucosyltransferase
1,01	THRA	thyroid hormone receptor, alpha
1,01	ZNF197	zinc finger protein 197
1,01	TNFRSF10B	tumor necrosis factor receptor superfamily, member 10b
1,01	ALDH5A1	aldehyde dehydrogenase 5 family, member A1
1,01	SLC9B1	solute carrier family 9, subfamily B (NHA1, cation proton antiporter 1), member 1
1,01	THEM6	thioesterase superfamily member 6
1,01	PTBP3	polypyrimidine tract binding protein 3
1,01	PTPN9	protein tyrosine phosphatase, non-receptor type 9
1,01	HS3ST6	heparan sulfate (glucosamine) 3-O-sulfotransferase 6
1,01	CLEC10A	C-type lectin domain family 10, member A
1,01	TNFRSF13C; MIR378I	tumor necrosis factor receptor superfamily, member 13C; microRNA 378i
1,01	CD7	CD7 molecule
1,01	CRYBB2	crystallin beta B2
1,01	SLC4A9	solute carrier family 4, sodium bicarbonate cotransporter, member 9
1,01	CYP21A1P; CYP21A2	cytochrome P450, family 21, subfamily A, polypeptide 1 pseudogene; cytochrome P450, family 21, subfamily A, polypeptide 2
1,01	STARD3NL	STARD3 N-terminal like
1,01	RPUSD4	RNA pseudouridylate synthase domain containing 4
1,01	GAK	cyclin G associated kinase
1,01	GLE1	GLE1 RNA export mediator
1,01	ATF1	activating transcription factor 1
1,01	OR5AC2	olfactory receptor, family 5, subfamily AC, member 2

1,01	RP11-484D2.4; TTC17	novel transcript, sense overlapping TTC17; Transcript Identified by AceView, Entrez Gene ID(s) 55761
1,01	NSMCE1	NSE1 homolog, SMC5-SMC6 complex component
1,01	INPP5K	inositol polyphosphate-5-phosphatase K
1,01	TMEM47	transmembrane protein 47
1,01	LRRN2	leucine rich repeat neuronal 2
1,01	GCC1	GRIP and coiled-coil domain containing 1
1,01	KLRG1	killer cell lectin-like receptor subfamily G, member 1
1,01	ARHGAP10	Rho GTPase activating protein 10
1,01	PMPCA	peptidase (mitochondrial processing) alpha
1,01	TRMT1	tRNA methyltransferase 1
1,01	CHCHD2	coiled-coil-helix-coiled-coil-helix domain containing 2
1,01	RPL21	ribosomal protein L21
1,01	EVX1	even-skipped homeobox 1
1,01	RPS19	ribosomal protein S19
1,01	HOOK3	hook microtubule-tethering protein 3
1,01	CACYBP	calcyclin binding protein
1,01	CISD2	CDGSH iron sulfur domain 2
1,01	SPIRE2	spire-type actin nucleation factor 2
1,01	SYNGR1	synaptogyrin 1
1,01	ARL16	ADP-ribosylation factor like GTPase 16
1,01	STK19	Homo sapiens serine/threonine kinase 19 (STK19), transcript variant 1, mRNA.; Homo sapiens serine/threonine kinase 19 (STK19), transcript variant 2, mRNA.; Homo sapiens serine/threonine kinase 19 (STK19), transcript variant 3, non-coding RNA.; Homo sapiens serine/threonine kinase 19, mRNA (cDNA clone MGC:117388 IMAGE:5165123), complete cds.
1,01	CTF1	cardiotrophin 1
1,01	MAP3K5	mitogen-activated protein kinase kinase kinase 5
1,01	HMGN4	high mobility group nucleosomal binding domain 4
1,01	ANKRD34A	ankyrin repeat domain 34A
1,01	SLC30A1	solute carrier family 30 (zinc transporter), member 1
1,01	CBR4	carbonyl reductase 4
1,01	POLR1B	polymerase (RNA) I polypeptide B
1,01	ZDHHC2	zinc finger, DHHC-type containing 2
1,01	RGS7	regulator of G-protein signaling 7
1,01	POLD2	polymerase (DNA directed), delta 2, accessory subunit
1,01	ING2	inhibitor of growth family member 2
1,01	ARMCX2	armadillo repeat containing, X-linked 2
1,01	TMED10	transmembrane p24 trafficking protein 10
1,01	CCDC84	coiled-coil domain containing 84
1,01	LZIC	leucine zipper and CTNNBIP1 domain containing
1,01	SPATA5	spermatogenesis associated 5

1,01	LOC100130691; AC074286.1; NFE2L2	uncharacterized LOC100130691; Transcript Identified by AceView, Entrez Gene ID(s) 100130691; 4780, RefSeq ID(s) NR_026966; novel transcript
1,01	IL3RA	Homo sapiens interleukin 3 receptor, alpha (low affinity) (IL3RA), transcript variant 2, mRNA.; Homo sapiens interleukin 3 receptor, alpha (low affinity) (IL3RA), transcript variant 1, mRNA.; interleukin 3 receptor, alpha (low affinity) [Source:HGNC Symbol;Acc:HGNC:6012]; Homo sapiens interleukin 3 receptor, alpha (low affinity), mRNA (cDNA clone MGC:34174 IMAGE:5167281), complete cds.; Salzman2013 ANNOTATED, CDS, coding, INTERNAL, OVCODE, OVERLAPTX, OVEXON best transcript NM_002183
1,01	ARMC1	armadillo repeat containing 1
1,01	GRIA2	Transcript Identified by AceView, Entrez Gene ID(s) 2891
1,01	HERC3	HECT and RLD domain containing E3 ubiquitin protein ligase 3
1,01	PHGDH	phosphoglycerate dehydrogenase
1,01	ACOT1	acyl-CoA thioesterase 1
1,01	PRAMEF1	PRAME family member 1
1,01	KCTD10	potassium channel tetramerization domain containing 10
1,01	PRPS1	phosphoribosyl pyrophosphate synthetase 1
1,01	DAB2IP	DAB2 interacting protein
1,01	TBC1D23	TBC1 domain family, member 23
1,01	SNTN	sentan, cilia apical structure protein
1,01	UBE2B	ubiquitin conjugating enzyme E2B
1,01	CLIP2	CAP-GLY domain containing linker protein 2
1,01	FGFRL1	fibroblast growth factor receptor-like 1
1,01	FUS	FUS RNA binding protein
1,01	ZNF23	zinc finger protein 23
1,01	VPS26A	VPS26 retromer complex component A
1,01	BCKDHB	branched chain keto acid dehydrogenase E1, beta polypeptide
1,01	LY6G6F	lymphocyte antigen 6 complex, locus G6F
1,01	SLC29A3	solute carrier family 29 (equilibrative nucleoside transporter), member 3
1,01	KAT6B	K(lysine) acetyltransferase 6B
1,01	ENOPH1	enolase-phosphatase 1
1,01	RAB33B	RAB33B, member RAS oncogene family
1,01	RUNX1	runt-related transcription factor 1
1,01	TBC1D25	TBC1 domain family, member 25
1,01	RABL2B	RAB, member of RAS oncogene family-like 2B
1,01	SUB1	SUB1 homolog, transcriptional regulator
1,01	MRTO4	MRT4 homolog, ribosome maturation factor
1,01	WIPF3	WAS/WASL interacting protein family, member 3
1,01	SLC25A10	solute carrier family 25 (mitochondrial carrier; dicarboxylate transporter), member 10

1,01	MAGEH1	MAGE family member H1
1,01	PTPN11	protein tyrosine phosphatase, non-receptor type 11
1,01	ZNF142	zinc finger protein 142
1,01	DRD3	dopamine receptor D3
1,01	CDS2	CDP-diacylglycerol synthase 2
1,01	EDN3	endothelin 3
1,01	BTBD6	BTB (POZ) domain containing 6
1,01	SMN1	survival of motor neuron 1, telomeric
1,01	PYCARD	PYD and CARD domain containing
1,01	NPIPA7	nuclear pore complex interacting protein family, member A7
1,01	USO1	USO1 vesicle transport factor
1,01	LUC7L3	LUC7-like 3 pre-mRNA splicing factor
1,01	EGFL6	EGF-like-domain, multiple 6
1,01	UPK3B	uroplakin 3B
1,01	OR4F6	olfactory receptor, family 4, subfamily F, member 6
1,01	MIR1199	microRNA 1199
1,01	RBP5	retinol binding protein 5, cellular
1,01	FAM92B	family with sequence similarity 92, member B
1,01	ARHGAP44	Rho GTPase activating protein 44
1,01	SLC4A7	solute carrier family 4, sodium bicarbonate cotransporter, member 7
1,01	EPB41L5	erythrocyte membrane protein band 4.1 like 5
1,01	WDR76	WD repeat domain 76
1,01	PEBP1	phosphatidylethanolamine binding protein 1
1,01	PLPPR1	phospholipid phosphatase related 1
1,01	HERC2P2	hect domain and RLD 2 pseudogene 2
1,01	C11orf49	chromosome 11 open reading frame 49
1,01	PCBD1	pterin-4 alpha-carbinolamine dehydratase/dimerization cofactor of hepatocyte nuclear factor 1 alpha
1,01	GFM2	G elongation factor, mitochondrial 2
1,01	RRN3	RRN3 homolog, RNA polymerase I transcription factor
1,01	ZNF816-ZNF321P	ZNF816-ZNF321P readthrough
1,01	SS18L2	synovial sarcoma translocation gene on chromosome 18-like 2
1,01	OR2L8	olfactory receptor, family 2, subfamily L, member 8 (gene/pseudogene)
1,01	FXYD6; FXYD6-FXYD2; FXYD2	FXYD domain containing ion transport regulator 6; FXYD6-FXYD2 readthrough; FXYD domain containing ion transport regulator 2
1,01	RASSF6	Ras association (RalGDS/AF-6) domain family member 6
1,01	KRTAP7-1	keratin associated protein 7-1 (gene/pseudogene)
1,01	RASGRP4	RAS guanyl releasing protein 4
1,01	EPHB3	EPH receptor B3
1,01	MRPL35	mitochondrial ribosomal protein L35
1,01	RSU1	Ras suppressor protein 1

1,01	FAM107A	family with sequence similarity 107, member A
1,01	IPO13	importin 13
1,01	SETD3	SET domain containing 3
1,01	DLG3	discs, large homolog 3 (Drosophila)
1,01	REEP6	receptor accessory protein 6
1,01	SNED1	Memczak2013 ALT_DONOR, coding, INTERNAL, intronic best transcript NM_001080437
1,01	ECT2	epithelial cell transforming 2
1,01	ZNF618	zinc finger protein 618
1,01	TCTN3	tectonic family member 3
1,01	PNP	purine nucleoside phosphorylase
1,01	PNMA1	paraneoplastic Ma antigen 1
1,01	SMIM6	small integral membrane protein 6
1,01	SENP7	SUMO1/sentrin specific peptidase 7
1,01	INSL5	insulin-like 5
1,01	ZNF850	zinc finger protein 850
1,01	RAP2C	RAP2C, member of RAS oncogene family
1,01	SERINC4	serine incorporator 4
1,01	GPX5	glutathione peroxidase 5
1,01	FRG2C	FSHD region gene 2 family, member C
1,01	EIF3M	eukaryotic translation initiation factor 3, subunit M
1,01	FAM170B	family with sequence similarity 170, member B
1,01	CEP78	centrosomal protein 78kDa
1,01	MARCH4	membrane associated ring finger 4
1,01	NRG4	neuregulin 4
1,01	CCL7	chemokine (C-C motif) ligand 7
1,01	METTL8	methyltransferase like 8
1,01	OR52L1	olfactory receptor, family 52, subfamily L, member 1
1,01	AGGF1	angiogenic factor with G-patch and FHA domains 1
1,01	MERTK	MER proto-oncogene, tyrosine kinase
1,01	HRK	harakiri, BCL2 interacting protein
1,01	ZNF787	zinc finger protein 787
1,01	LRRC73	leucine rich repeat containing 73
1,01	HIST1H2BA	histone cluster 1, H2ba
1,01	TRPC4	transient receptor potential cation channel, subfamily C, member 4
1,01	PRSS12	protease, serine, 12 (neurotrypsin, motopsin)
1,01	C22orf39; HIRA	chromosome 22 open reading frame 39; histone cell cycle regulator
1,01	UBE2O	ubiquitin-conjugating enzyme E2O
1,01	CD160	CD160 molecule
1,01	TIAM2	T-cell lymphoma invasion and metastasis 2
1,01	IGSF5	immunoglobulin superfamily, member 5
1,01	PSMA6	proteasome subunit alpha 6
1,01	CUEDC1	CUE domain containing 1
1,01	P2RX5	purinergic receptor P2X, ligand gated ion channel, 5
1,01	GPR137	G protein-coupled receptor 137
1,01	SNX24	sorting nexin 24
1,01	CFC1B; CFC1	cripto, FRL-1, cryptic family 1B; cripto, FRL-1, cryptic family 1

1,01	PIP5KL1	phosphatidylinositol-4-phosphate 5-kinase-like 1
1,01	SLC39A3	solute carrier family 39 (zinc transporter), member 3
1,01	C16orf54	chromosome 16 open reading frame 54
1,01	PRPS2	phosphoribosyl pyrophosphate synthetase 2
1,01	CFAP53	cilia and flagella associated protein 53
1,01	KDM7A	lysine (K)-specific demethylase 7A
1,01	TM4SF1-AS1	TM4SF1 antisense RNA 1
1,01	EIF4G2; SNORD97	eukaryotic translation initiation factor 4 gamma, 2; small nucleolar RNA, C/D box 97
1,01	SLC22A11	solute carrier family 22 (organic anion/urate transporter), member 11
1,01	KRTAP5-5	keratin associated protein 5-5
1,01	ITGB5	integrin beta 5
1,01	PEBP1	Memczak2013 ANTISENSE, coding, INTERNAL, UTR3 best transcript NM_002567
1,01	CHMP7	charged multivesicular body protein 7
1,01	DYNLL2	dynein, light chain, LC8-type 2
1,01	HS6ST3	heparan sulfate 6-O-sulfotransferase 3
1,01	ZFP3	ZFP3 zinc finger protein
1,01	CLCN4	chloride channel, voltage-sensitive 4
1,01	HIST1H2AI	histone cluster 1, H2ai
1,01	TYR	tyrosinase
1,01	VAMP8	vesicle associated membrane protein 8
1,01	LINC01465	long intergenic non-protein coding RNA 1465
1,01	ZNF525	zinc finger protein 525
1,01	PPIAL4G	peptidylprolyl isomerase A (cyclophilin A)-like 4G
1,01	ADAM8	ADAM metallopeptidase domain 8
1,01	TMEM208	transmembrane protein 208
1,01	COX6A1	cytochrome c oxidase subunit VIa polypeptide 1
1,01	CREB3	cAMP responsive element binding protein 3
1,01	PPP2R5E	protein phosphatase 2, regulatory subunit B, epsilon isoform
1,01	LNPEP	Memczak2013 ANTISENSE, coding, INTERNAL, UTR3 best transcript NM_175920
1,01	ENTPD3	ectonucleoside triphosphate diphosphohydrolase 3
1,01	AXIN1	axin 1
1,01	C1orf43	chromosome 1 open reading frame 43
1,01	SUSD1	sushi domain containing 1
1,01	RABGEF1	RAB guanine nucleotide exchange factor (GEF) 1
1,01	PRAMEF33P	PRAME family member 33, pseudogene
1,01	B3GALT2	UDP-Gal:betaGlcNAc beta 1,3-galactosyltransferase 2
1,01	USP16	ubiquitin specific peptidase 16
1,01	PCYT1A	phosphate cytidylyltransferase 1, choline, alpha
1,01	BACH2	Memczak2013 ALT_ACCEPTOR, ALT_DONOR, coding, INTERNAL, intronic best transcript NM_001170794
1,01	CAPN2	calpain 2, (m/II) large subunit
1,01	SLC47A2	solute carrier family 47 (multidrug and toxin extrusion), member 2

1,01	ADGRE5	adhesion G protein-coupled receptor E5
1,01	NUFIP2	nuclear fragile X mental retardation protein interacting protein 2
1,01	MRPS5	mitochondrial ribosomal protein S5
1,01	C9orf9	chromosome 9 open reading frame 9
1,01	HIST2H4B; HIST2H4A	histone cluster 2, H4b; histone cluster 2, H4a
1,01	MRPL19	mitochondrial ribosomal protein L19
1,01	SLC9A9	solute carrier family 9, subfamily A (NHE9, cation proton antiporter 9), member 9
1,01	GALNTL6	polypeptide N-acetylgalactosaminyltransferase-like 6
1,01	HLA-DQA1	major histocompatibility complex, class II, DQ alpha 1
1,01	TNNI3	troponin I type 3 (cardiac)
1,01	NACA	nascent polypeptide-associated complex alpha subunit
1,01	AICDA	activation-induced cytidine deaminase
1,01	TMCC3; MIR7844	transmembrane and coiled-coil domain family 3; microRNA 7844
1,01	CCDC169	coiled-coil domain containing 169
1,01	RPL23AP5	ribosomal protein L23a pseudogene 5
1,01	EIF1B	eukaryotic translation initiation factor 1B
1,01	MTFR2	mitochondrial fission regulator 2
1,01	SAMD10	sterile alpha motif domain containing 10
1,01	USP49	ubiquitin specific peptidase 49
1,01	RGS8	regulator of G-protein signaling 8
1,01	STX16	syntaxin 16
1,01	RNF175	ring finger protein 175
1,01	CD3EAP	CD3e molecule, epsilon associated protein
1,01	TCERG1	transcription elongation regulator 1
1,01	C20orf196	Transcript Identified by AceView, Entrez Gene ID(s) 149840
1,01	INAFM2	InaF-motif containing 2
1,01	GOSR2	golgi SNAP receptor complex member 2
1,01	MCM3	minichromosome maintenance complex component 3
1,01	MYH13	myosin, heavy chain 13, skeletal muscle
1,01	LINC01567	long intergenic non-protein coding RNA 1567
1,01	TXNDC5	thioredoxin domain containing 5 (endoplasmic reticulum)
1,01	KIF3B	kinesin family member 3B
1,01	ZNF724P	zinc finger protein 724, pseudogene
1,01	CWC15	CWC15 spliceosome-associated protein
1,00	SAG	S-antigen; retina and pineal gland (arrestin)
1,00	TMEM61	transmembrane protein 61
1,00	PPP1R2	protein phosphatase 1, regulatory (inhibitor) subunit 2
1,00	DYNC2H1	dynein, cytoplasmic 2, heavy chain 1
1,00	SPATA9	spermatogenesis associated 9
1,00	HIST1H2BI	histone cluster 1, H2bi
1,00	ATG16L1	autophagy related 16-like 1

1,00	TCEB1	transcription elongation factor B (SIII), polypeptide 1 (15kDa, elongin C)
1,00	CYP2C8	cytochrome P450, family 2, subfamily C, polypeptide 8
1,00	PDLIM4	PDZ and LIM domain 4
1,00	RNF115	ring finger protein 115
1,00	RRNAD1	ribosomal RNA adenine dimethylase domain containing 1
1,00	SMTNL1	smoothelin-like 1
1,00	STAR	steroidogenic acute regulatory protein
1,00	CXorf56	chromosome X open reading frame 56
1,00	THAP8	THAP domain containing 8
1,00	KANSL1	KAT8 regulatory NSL complex subunit 1
1,00	ATG16L1	autophagy related 16-like 1
1,00	APBA2	amyloid beta (A4) precursor protein-binding, family A, member 2
1,00	SMIM1	small integral membrane protein 1 (Vel blood group)
1,00	FBXL15	F-box and leucine-rich repeat protein 15
1,00	XPO1	exportin 1
1,00	ANLN	anillin actin binding protein
1,00	FAM26D	family with sequence similarity 26, member D
1,00	KIF18B	kinesin family member 18B
1,00	PTPMT1	protein tyrosine phosphatase, mitochondrial 1
1,00	CPAMD8	C3 and PZP-like, alpha-2-macroglobulin domain containing 8
1,00	COL11A1	collagen, type XI, alpha 1
1,00	C17orf53	chromosome 17 open reading frame 53
1,00	LCE3E	late cornified envelope 3E
1,00	REXO1L10P	REX1, RNA exonuclease 1 homolog-like 10, pseudogene
1,00	GRSF1	G-rich RNA sequence binding factor 1
1,00	KCNJ2	potassium channel, inwardly rectifying subfamily J, member 2
1,00	MLXIP	MLX interacting protein
1,00	SRP72	signal recognition particle 72kDa
1,00	CEACAM20	carcinoembryonic antigen-related cell adhesion molecule 20
1,00	MID1IP1	MID1 interacting protein 1
1,00	GBP7	guanylate binding protein 7
1,00	TOM1	target of myb1 membrane trafficking protein
1,00	PSMC5	proteasome 26S subunit, ATPase 5
1,00	EEF1E1-BLOC1S5	EEF1E1-BLOC1S5 readthrough (NMD candidate)
1,00	DGKD	diacylglycerol kinase, delta 130kDa
1,00	MUTYH	mutY DNA glycosylase
1,00	IFT74	intraflagellar transport 74
1,00	SARS	seryl-tRNA synthetase
1,00	LINC00493	long intergenic non-protein coding RNA 493
1,00	PARP8	Memczak2013 ANTISENSE, CDS, coding, INTERNAL, intronic best transcript NM_001178056

1,00	AGTR1	angiotensin II receptor, type 1
1,00	WARS2	tryptophanyl tRNA synthetase 2, mitochondrial
1,00	CBX3	chromobox homolog 3
1,00	GDAP1L1	ganglioside induced differentiation associated protein 1-like 1
1,00	ZNF3	zinc finger protein 3
1,00	EPRS	glutamyl-prolyl-tRNA synthetase
1,00	IRAK1; MIR718	interleukin 1 receptor associated kinase 1; microRNA 718
1,00	STX12	syntaxin 12
1,00	KIF13B	kinesin family member 13B
1,00	IPO9	importin 9
1,00	UNC5A	unc-5 netrin receptor A
1,00	SHFM1	split hand/foot malformation (ectrodactyly) type 1
1,00	C1QTNF7	C1q and tumor necrosis factor related protein 7
1,00	UBA1	ubiquitin-like modifier activating enzyme 1
1,00	CLCN3	chloride channel, voltage-sensitive 3
1,00	NDUFB4	NADH dehydrogenase (ubiquinone) 1 beta subcomplex, 4, 15kDa
1,00	GAPDH	glyceraldehyde-3-phosphate dehydrogenase
1,00	ANGPTL8	angiopoietin like 8
1,00	UNC119B; MIR4700	unc-119 lipid binding chaperone B; microRNA 4700
1,00	ARF4	ADP-ribosylation factor 4
1,00	FOXP2	forkhead box P2
1,00	ZNF726; ZNF92P3	zinc finger protein 726; zinc finger protein 92 pseudogene 3
1,00	IPO4	importin 4
1,00	LOX	lysyl oxidase
1,00	KIAA2012	KIAA2012
1,00	COX6B1	cytochrome c oxidase subunit VIb polypeptide 1 (ubiquitous)
1,00	C6orf222	chromosome 6 open reading frame 222
1,00	KLF1	Kruppel-like factor 1 (erythroid)
1,00	ANKS3	ankyrin repeat and sterile alpha motif domain containing 3
1,00	ZNF319	zinc finger protein 319
1,00	IFNAR1	interferon (alpha, beta and omega) receptor 1
1,00	ADAMTS13	ADAM metallopeptidase with thrombospondin type 1 motif 13
1,00	OTOP1	otopetrin 1
1,00	FEM1C	fem-1 homolog c (C. elegans)
1,00	ARHGAP35	Rho GTPase activating protein 35
1,00	BTN3A3	butyrophilin, subfamily 3, member A3
1,00	FTSJ3	FtsJ homolog 3 (E. coli)
1,00	EIF4E	eukaryotic translation initiation factor 4E
1,00	PLIN4	perilipin 4
1,00	PRPF19	pre-mRNA processing factor 19
1,00	CIPC	CLOCK-interacting pacemaker
1,00	TLR3	toll-like receptor 3

1,00	TSEN54	TSEN54 tRNA splicing endonuclease subunit
1,00	CACNA2D1	calcium channel, voltage-dependent, alpha 2/delta subunit 1
1,00	ZBTB8B	zinc finger and BTB domain containing 8B
1,00	C10orf82	chromosome 10 open reading frame 82
1,00	ADAM8	Memczak2013 ANTISENSE, CDS, coding, INTERNAL best transcript NM_001109
1,00	THEGL	theg spermatid protein-like
1,00	STRN	striatin, calmodulin binding protein
1,00	MANSC1	MANSC domain containing 1
1,00	DARS	aspartyl-tRNA synthetase
1,00	SLC19A3	solute carrier family 19 (thiamine transporter), member 3
1,00	CHEK1	checkpoint kinase 1
1,00	SDHAF4	succinate dehydrogenase complex assembly factor 4
1,00	TISP43; LOC646743; LOC150527	Homo sapiens uncharacterized LOC150527 (TISP43), transcript variant 1, non-coding RNA.; Homo sapiens uncharacterized LOC150527 (TISP43), transcript variant 2, non-coding RNA.; Homo sapiens uncharacterized LOC646743 (LOC646743), non-coding RNA.; Transcript Identified by AceView, Entrez Gene ID(s) 150527; Transcript Identified by AceView, Entrez Gene ID(s) 150527, RefSeq ID(s) NR_027313; uncharacterized LOC150527 [Source:EntrezGene;Acc:150527]
1,00	PRKAB2	protein kinase, AMP-activated, beta 2 non-catalytic subunit
1,00	LCE1E	late cornified envelope 1E
1,00	EMID1	EMI domain containing 1
1,00	XPNPEP3	X-prolyl aminopeptidase 3, mitochondrial
1,00	FBXO18	F-box protein, helicase, 18
1,00	NARS	asparaginyl-tRNA synthetase
1,00	SLC36A3	solute carrier family 36, member 3
1,00	VEZT	vezatin, adherens junctions transmembrane protein
1,00	STAP1	signal transducing adaptor family member 1
1,00	KRT25	keratin 25, type I
1,00	SEPW1	selenoprotein W, 1
1,00	HS3ST3A1	heparan sulfate (glucosamine) 3-O-sulfotransferase 3A1
1,00	ZDHHC21	zinc finger, DHHC-type containing 21
1,00	LAMTOR1	late endosomal/lysosomal adaptor, MAPK and MTOR activator 1
1,00	MITF	microphthalmia-associated transcription factor
1,00	SLC19A2	solute carrier family 19 (thiamine transporter), member 2
1,00	FOXD4L4	forkhead box D4-like 4
1,00	SUCLG1	succinate-CoA ligase, alpha subunit
1,00	FRA10AC1	fragile site, folic acid type, rare, fra(10)(q23.3) or fra(10)(q24.2) candidate 1
1,00	MDH2	malate dehydrogenase 2
1,00	ZC3H3	zinc finger CCCH-type containing 3

1,00	JSRP1	junctional sarcoplasmic reticulum protein 1
1,00	GRAMD3	GRAM domain containing 3
1,00	F9	coagulation factor IX
1,00	FAM174A	family with sequence similarity 174, member A
1,00	CTAGE8	CTAGE family, member 8
1,00	SUMO1	small ubiquitin-like modifier 1
1,00	MSANTD2	Myb/SANT-like DNA-binding domain containing 2
1,00	VSIG10L	V-set and immunoglobulin domain containing 10 like
1,00	MTHFD2	methylenetetrahydrofolate dehydrogenase (NADP+ dependent) 2, methenyltetrahydrofolate cyclohydrolase
1,00	ORC5	origin recognition complex subunit 5
1,00	GLUL	glutamate-ammonia ligase
1,00	GFRAL	GDNF family receptor alpha like
1,00	RAB11FIP5	RAB11 family interacting protein 5 (class I)
1,00	NXT1	nuclear transport factor 2-like export factor 1
1,00	SH2D7	SH2 domain containing 7
1,00	SLC35F4	solute carrier family 35, member F4
1,00	TCHP	trichoplein, keratin filament binding
1,00	THBS4	thrombospondin 4
1,00	TM4SF19	transmembrane 4 L six family member 19
1,00	PDE4C	phosphodiesterase 4C, cAMP-specific
1,00	KDM4E	lysine (K)-specific demethylase 4E
1,00	LYPD1	LY6/PLAUR domain containing 1
1,00	C7orf26	chromosome 7 open reading frame 26
1,00	UBR2	ubiquitin protein ligase E3 component n-recognin 2
1,00	ISY1-RAB43	ISY1-RAB43 readthrough
1,00	CSPG5	chondroitin sulfate proteoglycan 5 (neuroglycan C)
1,00	OXTR	oxytocin receptor
1,00	MRPL16	mitochondrial ribosomal protein L16
1,00	SFMBT1	Scm-like with four mbt domains 1
1,00	C22orf46	chromosome 22 open reading frame 46
1,00	MANEA	mannosidase, endo-alpha
1,00	ACAT2	acetyl-CoA acetyltransferase 2
1,00	SLC7A5	solute carrier family 7 (amino acid transporter light chain, L system), member 5
1,00	EML2-AS1	EML2 antisense RNA 1
1,00	SIAH2	siah E3 ubiquitin protein ligase 2
1,00	ZDHHC22	zinc finger, DHHC-type containing 22
1,00	MBTD1	mbt domain containing 1
1,00	FLT4	fms-related tyrosine kinase 4
1,00	TJP3	tight junction protein 3
1,00	S100A16	S100 calcium binding protein A16
1,00	SEC23IP	SEC23 interacting protein
1,00	USP24	ubiquitin specific peptidase 24
1,00	HNRNPR	heterogeneous nuclear ribonucleoprotein R
1,00	CCR10	chemokine (C-C motif) receptor 10
1,00	DEFB129	defensin, beta 129
1,00	NDUFV2	NADH dehydrogenase (ubiquinone) flavoprotein 2, 24kDa

1,00	PCID2	PCI domain containing 2
1,00	ZNF891	zinc finger protein 891
1,00	CFAP47	cilia and flagella associated protein 47
1,00	ERI2	ERI1 exoribonuclease family member 2
1,00	CANT1	calcium activated nucleotidase 1
1,00	SMAD4	SMAD family member 4
1,00	CCR4	chemokine (C-C motif) receptor 4
1,00	HPCAL1	hippocalcin-like 1
1,00	LAMP2	lysosomal-associated membrane protein 2
1,00	RFX5	regulatory factor X, 5 (influences HLA class II expression)
1,00	MB	myoglobin
1,00	PROCR	protein C receptor, endothelial
1,00	VPS41	vacuolar protein sorting 41 homolog (S. cerevisiae)
1,00	DNPEP	aspartyl aminopeptidase
1,00	MTIF3	mitochondrial translational initiation factor 3
1,00	SMARCB1	SWI/SNF related, matrix associated, actin dependent regulator of chromatin, subfamily b, member 1
1,00	ZNF791	zinc finger protein 791
1,00	PSMC4	proteasome 26S subunit, ATPase 4 [Source:HGNC Symbol;Acc:HGNC:9551]
1,00	ATG16L1	autophagy related 16-like 1
1,00	ENTPD4; LOXL2	ectonucleoside triphosphate diphosphohydrolase 4; lysyl oxidase-like 2
1,00	HNRNPUL1	heterogeneous nuclear ribonucleoprotein U-like 1
1,00	CAMK2D	calcium/calmodulin-dependent protein kinase II delta
1,00	RBM28	RNA binding motif protein 28
1,00	CCDC68	coiled-coil domain containing 68
1,00	XCL1	chemokine (C motif) ligand 1
1,00	FAM196A	family with sequence similarity 196, member A
1,00	HSPA1B; HSPA1A	heat shock 70kDa protein 1B; heat shock 70kDa protein 1A
1,00	CNBD1	cyclic nucleotide binding domain containing 1
1,00	CELSR3; MIR4793	cadherin, EGF LAG seven-pass G-type receptor 3; microRNA 4793
1,00	RASA3	RAS p21 protein activator 3
1,00	SDS	serine dehydratase
1,00	C9orf78	chromosome 9 open reading frame 78
1,00	FBL	fibrillarin
1,00	ASAH2B	N-acylsphingosine amidohydrolase (non-lysosomal ceramidase) 2B
1,00	MOGS	mannosyl-oligosaccharide glucosidase
1,00	RNF13	ring finger protein 13
1,00	DBNDD1	dysbindin (dystrobrevin binding protein 1) domain containing 1
1,00	PPP6R3	protein phosphatase 6, regulatory subunit 3
1,00	MBNL2	muscleblind-like splicing regulator 2
1,00	CRAMP1	cramped chromatin regulator homolog 1
1,00	SPRY2	sprouty RTK signaling antagonist 2
1,00	ZNF324B	zinc finger protein 324B

1,00	OR13C4	olfactory receptor, family 13, subfamily C, member 4
1,00	GFI1	growth factor independent 1 transcription repressor
1,00	C7orf34	chromosome 7 open reading frame 34
1,00	MRPL15	mitochondrial ribosomal protein L15
1,00	RPL5	Memczak2013 ALT_ACCEPTOR, ALT_DONOR, coding, INTERNAL, intronic best transcript NM_000969
1,00	CDCP1	CUB domain containing protein 1
1,00	ZNF727	zinc finger protein 727
1,00	HIST3H3	histone cluster 3, H3
1,00	CCNT2	cyclin T2
1,00	MAPK4	mitogen-activated protein kinase 4
1,00	GALNS	galactosamine (N-acetyl)-6-sulfatase
1,00	ZNF586	zinc finger protein 586
1,00	C1orf115	chromosome 1 open reading frame 115
1,00	SATB1	SATB homeobox 1
1,00	GBA	glucosidase, beta, acid
1,00	ERCC3	excision repair cross-complementation group 3
1,00	PTEN	phosphatase and tensin homolog
1,00	HAUS3; POLN	HAUS augmin like complex subunit 3; polymerase (DNA directed) nu
1,00	MYO19	myosin XIX
1,00	PPP3R1	protein phosphatase 3, regulatory subunit B, alpha
1,00	ITIH4	inter-alpha-trypsin inhibitor heavy chain family, member 4
1,00	GOSR1	golgi SNAP receptor complex member 1
1,00	CD247	CD247 molecule
1,00	TCTEX1D4	Tctex1 domain containing 4
1,00	FIGNL1	fidgetin-like 1
1,00	GALNT7	polypeptide N-acetylgalactosaminyltransferase 7
1,00	TRAPPC4	trafficking protein particle complex 4
1,00	CYP1A2	cytochrome P450, family 1, subfamily A, polypeptide 2
1,00	ETFDH	electron-transferring-flavoprotein dehydrogenase
1,00	EIF3E	eukaryotic translation initiation factor 3, subunit E
1,00	GPD1	glycerol-3-phosphate dehydrogenase 1
1,00	RFX8	RFX family member 8, lacking RFX DNA binding domain
1,00	CREBRF	CREB3 regulatory factor
1,00	BCAP31	B-cell receptor-associated protein 31
1,00	AP1S2	adaptor-related protein complex 1 sigma 2 subunit
1,00	HIST1H1C	histone cluster 1, H1c
1,00	HNRNPC	heterogeneous nuclear ribonucleoprotein C (C1/C2)
1,00	MTMR10	myotubularin related protein 10
1,00	EMX2	empty spiracles homeobox 2
1,00	CDK20	cyclin-dependent kinase 20
1,00	UTP14A	UTP14A small subunit (SSU) processome component
1,00	PSMD14	proteasome 26S subunit, non-ATPase 14
1,00	SIAH1	siah E3 ubiquitin protein ligase 1
1,00	PHF19	PHD finger protein 19

1,00	MRPL40	mitochondrial ribosomal protein L40
1,00	CCDC168	coiled-coil domain containing 168
1,00	YARS	tyrosyl-tRNA synthetase
1,00	ZNF425	zinc finger protein 425
1,00	SRCIN1	SRC kinase signaling inhibitor 1
1,00	C11orf97	chromosome 11 open reading frame 97
1,00	PTDSS1	phosphatidylserine synthase 1
1,00	TEX26	testis expressed 26
1,00	TM2D3	TM2 domain containing 3
1,00	PXK	PX domain containing serine/threonine kinase
1,00	RASAL3	RAS protein activator like 3
1,00	C21orf33	chromosome 21 open reading frame 33
1,00	IL27RA	interleukin 27 receptor, alpha
1,00	IRF2BP1	interferon regulatory factor 2 binding protein 1
1,00	MIEF1	mitochondrial elongation factor 1
1,00	TNNT3	troponin T type 3 (skeletal, fast)
1,00	TYSND1	trypsin domain containing 1
1,00	ATAD3C	ATPase family, AAA domain containing 3C
1,00	SNED1	sushi, nidogen and EGF-like domains 1
1,00	PSMD6	proteasome 26S subunit, non-ATPase 6
1,00	ZZZ3	zinc finger, ZZ-type containing 3
1,00	ITLN1	intelectin 1 (galactofuranose binding)
1,00	QARS; MIR6890	glutaminyl-tRNA synthetase; microRNA 6890
1,00	EGFLAM	EGF-like, fibronectin type III and laminin G domains
1,00	NAP1L3	nucleosome assembly protein 1-like 3
1,00	AGPAT1; MIR6721	1-acylglycerol-3-phosphate O-acyltransferase 1; microRNA 6721
1,00	CNTN6	contactin 6
1,00	PROC	protein C (inactivator of coagulation factors Va and VIIIa)
1,00	POTEM; POTEG	POTE ankyrin domain family, member M; POTE ankyrin domain family, member G
1,00	CAPRIN1	cell cycle associated protein 1
1,00	THSD4	Transcript Identified by AceView, Entrez Gene ID(s) 79875
1,00	ZACN	zinc activated ligand-gated ion channel
1,00	OR4P4	olfactory receptor, family 4, subfamily P, member 4
1,00	COMMD5	COMM domain containing 5
1,00	PSMD11	proteasome 26S subunit, non-ATPase 11
1,00	DPEP2	dipeptidase 2
1,00	CCDC180	coiled-coil domain containing 180
1,00	PTPN3	protein tyrosine phosphatase, non-receptor type 3
1,00	SLC30A6	solute carrier family 30 (zinc transporter), member 6
1,00	NBPF3	neuroblastoma breakpoint family, member 3
1,00	SLMAP	sarcolemma associated protein
1,00	PROS1	protein S (alpha)
1,00	MIA2	melanoma inhibitory activity 2
1,00	RPTOR	regulatory associated protein of MTOR, complex 1
1,00	STRA8	stimulated by retinoic acid 8
1,00	ZBTB2	zinc finger and BTB domain containing 2

1,00	CD3G	CD3g molecule, gamma (CD3-TCR complex)
1,00	ADAMTS19	ADAM metallopeptidase with thrombospondin type 1 motif 19
1,00	NPAS4	neuronal PAS domain protein 4
1,00	ZNHIT2	zinc finger, HIT-type containing 2
1,00	PACSIN1	protein kinase C and casein kinase substrate in neurons 1
1,00	HSPE1-MOB4	HSPE1-MOB4 readthrough
1,00	BTF3	basic transcription factor 3
1,00	KLK14	kallikrein related peptidase 14
1,00	TTC14	tetratricopeptide repeat domain 14
1,00	NPIPA3	nuclear pore complex interacting protein family, member A3
1,00	RND3	Rho family GTPase 3
1,00	LGALS3	lectin, galactoside-binding, soluble, 3
1,00	UBQLN2	ubiquilin 2
1,00	TMEM123	transmembrane protein 123
1,00	NPIPA2	nuclear pore complex interacting protein family, member A2
1,00	TRIP10	thyroid hormone receptor interactor 10
1,00	VHL	von Hippel-Lindau tumor suppressor, E3 ubiquitin protein ligase
1,00	ANKZF1	ankyrin repeat and zinc finger domain containing 1
1,00	DCTN6	dynactin 6
1,00	TAF10	TAF10 RNA polymerase II, TATA box binding protein (TBP)-associated factor, 30kDa
1,00	KIAA1211	KIAA1211
1,00	NOC2L	NOC2-like nucleolar associated transcriptional repressor
1,00	COL4A3BP	collagen, type IV, alpha 3 (Goodpasture antigen) binding protein
1,00	HSDL2	hydroxysteroid dehydrogenase like 2
1,00	TAS2R19	taste receptor, type 2, member 19
1,00	RALGDS	ral guanine nucleotide dissociation stimulator
1,00	ANXA5	annexin A5
1,00	REXO2	RNA exonuclease 2
1,00	CCT4	chaperonin containing TCP1, subunit 4 (delta)
1,00	CFAP97	cilia and flagella associated protein 97
1,00	SGK494; FLJ25006; KIAA0100andSGK494andSPAG5; RP11-192H23.4	uncharacterized serine/threonine-protein kinase SgK494; Synthetic construct Homo sapiens clone IMAGE:100064171, MGC:193254 uncharacterized serine/threonine-protein kinase SgK494 (FLJ25006) mRNA, encodes complete protein.; Salzman2013 ANNOTATED, CDS, coding, INTERNAL, OVCODE, OVERLAPTX, OVEXON best transcript NM_001174103; Transcript Identified by AceView, Entrez Gene ID(s) 124923; 9703; 645851; 10615; uncharacterized serine/threonine-protein kinase SgK494 [Source:EntrezGene;Acc:124923]; Uncharacterized protein [Source:UniProtKB/TrEMBL;Acc:J3KTE0]

1,00	TMIGD2	transmembrane and immunoglobulin domain containing 2
1,00	ZCRB1; MIR7851	zinc finger CCHC-type and RNA binding motif 1; microRNA 7851
1,00	COL18A1-AS1	COL18A1 antisense RNA 1
1,00	CAMK2B	Transcript Identified by AceView, Entrez Gene ID(s) 816
1,00	OR10A6	olfactory receptor family 10 subfamily A member 6 (gene/pseudogene) [Source:HGNC Symbol;Acc:HGNC:15132]
1,00	PPP1R15B	protein phosphatase 1, regulatory subunit 15B
1,00	TEX11	testis expressed 11
1,00	FBL	fibrillarin
1,00	LAT2	linker for activation of T-cells family member 2
1,00	B3GNT7	UDP-GlcNAc:betaGal beta-1,3-N-acetylglucosaminyltransferase 7
1,00	SNX3	sorting nexin 3
1,00	EPB42	erythrocyte membrane protein band 4.2
1,00	TRIP13	thyroid hormone receptor interactor 13
1,00	ZXDC	ZXD family zinc finger C
1,00	EIF2B1	eukaryotic translation initiation factor 2B, subunit 1 alpha, 26kDa
1,00	LOC100131496; RP4-569M23.5	uncharacterized LOC100131496; novel transcript, antisense to ZMYND8
1,00	RAN	RAN, member RAS oncogene family
1,00	GOLGA8EP; GOLGA8DP	golgin A8 family, member E, pseudogene; golgin A8 family, member D, pseudogene
1,00	KLHL32	kelch-like family member 32
1,00	ENTPD6	ectonucleoside triphosphate diphosphohydrolase 6 (putative)
1,00	SRC	SRC proto-oncogene, non-receptor tyrosine kinase
1,00	SRSF4	serine/arginine-rich splicing factor 4
1,00	NR2E1	nuclear receptor subfamily 2, group E, member 1
1,00	KCNJ13	potassium channel, inwardly rectifying subfamily J, member 13
1,00	LY6G6E	lymphocyte antigen 6 complex, locus G6E (pseudogene)
1,00	TOLLIP	toll interacting protein
1,00	ZNF124	zinc finger protein 124
1,00	IFITM3	interferon induced transmembrane protein 3
1,00	CIR1	corepressor interacting with RBPJ, 1
1,00	C1orf210	chromosome 1 open reading frame 210
1,00	EXOSC7	exosome component 7
1,00	ZFAND3	zinc finger, AN1-type domain 3
1,00	DDX27	DEAD (Asp-Glu-Ala-Asp) box polypeptide 27
1,00	TFRC	transferrin receptor
1,00	CD164	CD164 molecule, sialomucin
1,00	SLC22A7	solute carrier family 22 (organic anion transporter), member 7
1,00	NME9	NME/NM23 family member 9

1,00	PIK3C2G	phosphatidylinositol-4-phosphate 3-kinase, catalytic subunit type 2 gamma
1,00	HK3	hexokinase 3 (white cell)
1,00	LCE1B	late cornified envelope 1B
1,00	SLC30A7	solute carrier family 30 (zinc transporter), member 7
1,00	NDE1; MIR484	nudE neurodevelopment protein 1; microRNA 484
1,00	MBD2	methyl-CpG binding domain protein 2
1,00	PLOD1	procollagen-lysine, 2-oxoglutarate 5-dioxygenase 1
1,00	INS; INS-IGF2	insulin; INS-IGF2 readthrough
1,00	TRAK2	trafficking protein, kinesin binding 2
1,00	NUDT7	nudix hydrolase 7
1,00	NLRX1	NLR family member X1
1,00	IFRD2	interferon-related developmental regulator 2
1,00	BRIP1	BRCA1 interacting protein C-terminal helicase 1
1,00	PEX11B	peroxisomal biogenesis factor 11 beta
1,00	OR51Q1	olfactory receptor, family 51, subfamily Q, member 1 (gene/pseudogene)
1,00	NKX2-4	NK2 homeobox 4
1,00	RABEPK	Rab9 effector protein with kelch motifs
1,00	ADGRG1	adhesion G protein-coupled receptor G1
1,00	MOSPD3	motile sperm domain containing 3
1,00	DNAJA3	DnaJ (Hsp40) homolog, subfamily A, member 3
1,00	STRADB	STE20-related kinase adaptor beta
1,00	FHL5	four and a half LIM domains 5
1,00	MAPK6	mitogen-activated protein kinase 6
1,00	TGFBRAP1	transforming growth factor beta receptor associated protein 1
1,00	NKIRAS1	NFKB inhibitor interacting Ras-like 1
1,00	PAPSS1	3-phosphoadenosine 5-phosphosulfate synthase 1
1,00	SLC27A5	solute carrier family 27 (fatty acid transporter), member 5
1,00	CHERP	calcium homeostasis endoplasmic reticulum protein
1,00	CSF1	colony stimulating factor 1 (macrophage)
1,00	PIRT	phosphoinositide-interacting regulator of transient receptor potential channels
1,00	MITD1	microtubule interacting and trafficking domain containing 1
1,00	ANKMY2	ankyrin repeat and MYND domain containing 2
1,00	MIDN	midnolin
1,00	CDK10	cyclin-dependent kinase 10
1,00	GSS	glutathione synthetase
1,00	DTX3	deltex 3, E3 ubiquitin ligase
1,00	TYRP1	tyrosinase-related protein 1
1,00	MEGF9	multiple EGF-like-domains 9
1,00	MYH3	myosin, heavy chain 3, skeletal muscle, embryonic
1,00	GAGE12C; GAGE12E; GAGE12H	G antigen 12C; G antigen 12E; G antigen 12H
1,00	FBXO5	F-box protein 5
1,00	BZW1	basic leucine zipper and W2 domains 1

1,00	NWD1	NACHT and WD repeat domain containing 1
1,00	NDUFA9	NADH dehydrogenase (ubiquinone) 1 alpha subcomplex, 9, 39kDa
1,00	PODNL1	podocan-like 1
1,00	ZNF233	zinc finger protein 233
1,00	LENG8	leukocyte receptor cluster (LRC) member 8
1,00	OBP2B	odorant binding protein 2B
1,00	OR7A17	olfactory receptor, family 7, subfamily A, member 17
1,00	HEPH	hephaestin
1,00	COX6C	cytochrome c oxidase subunit VIc
1,00	LEMD3	LEM domain containing 3
1,00	HPSE	heparanase
1,00	ZKSCAN7	zinc finger with KRAB and SCAN domains 7
1,00	SLC39A8	solute carrier family 39 (zinc transporter), member 8
1,00	TIPRL	TOR signaling pathway regulator
1,00	SUPT20HL1	SPT20 homolog, SAGA complex component-like 1
1,00	OGDH	oxoglutarate (alpha-ketoglutarate) dehydrogenase (lipoamide)
1,00	CDC42EP2	CDC42 effector protein (Rho GTPase binding) 2
1,00	C1orf220	chromosome 1 open reading frame 220
1,00	ERG	v-ets avian erythroblastosis virus E26 oncogene homolog
1,00	FAM43A	family with sequence similarity 43, member A
1,00	CARS	cysteinyl-tRNA synthetase
1,00	SPECC1L-ADORA2A	SPECC1L-ADORA2A readthrough (NMD candidate)
1,00	HAGHL	hydroxyacylglutathione hydrolase-like
1,00	JKAMP	JNK1/MAPK8-associated membrane protein
1,00	NOL8	nucleolar protein 8
1,00	ECI2	enoyl-CoA delta isomerase 2
1,00	SETD6	SET domain containing 6
1,00	ZNF627	zinc finger protein 627
1,00	PADI2	peptidyl arginine deiminase, type II
1,00	DPP9	dipeptidyl-peptidase 9
1,00	MRPL18	mitochondrial ribosomal protein L18
1,00	HMBS	hydroxymethylbilane synthase
1,00	MGAT4B	mannosyl (alpha-1,3-)-glycoprotein beta-1,4-N-acetylglucosaminyltransferase, isozyme B
1,00	WNK4	WNK lysine deficient protein kinase 4
1,00	VCPIP1	valosin containing protein (p97)/p47 complex interacting protein 1
1,00	NUDT8	nudix hydrolase 8
1,00	STARD5	StAR-related lipid transfer domain containing 5
1,00	JUND	jun D proto-oncogene
1,00	SCEL	sciellin
1,00	FAM163A	family with sequence similarity 163, member A
1,00	KLK10	kallikrein related peptidase 10
1,00	KHDC1	KH homology domain containing 1
1,00	BRCC3	BRCA1/BRCA2-containing complex subunit 3
1,00	POLR3B	polymerase (RNA) III (DNA directed) polypeptide B

1,00	CCDC71L	coiled-coil domain containing 71-like
1,00	HNRNPA1	heterogeneous nuclear ribonucleoprotein A1
1,00	OARD1	O-acyl-ADP-ribose deacylase 1
1,00	PBK	PDZ binding kinase
1,00	ANKRD9	ankyrin repeat domain 9
1,00	CDC40	cell division cycle 40
1,00	LSM2	LSM2 homolog, U6 small nuclear RNA and mRNA degradation associated
1,00	TRAM1	translocation associated membrane protein 1
1,00	A2BP1	Transcript Identified by AceView, Entrez Gene ID(s) 54715
1,00	APOM	apolipoprotein M
1,00	ZNF264	zinc finger protein 264
1,00	SNU13	SNU13 homolog, small nuclear ribonucleoprotein (U4/U6.U5)
1,00	OR52K1	olfactory receptor, family 52, subfamily K, member 1
1,00	SEMA3A	sema domain, immunoglobulin domain (Ig), short basic domain, secreted, (semaphorin) 3A
1,00	PRKCZ	protein kinase C, zeta
1,00	TRAF5	TNF receptor-associated factor 5
1,00	LMAN1	lectin, mannose-binding, 1
1,00	CPA3	carboxypeptidase A3 (mast cell)
1,00	BCAP29	B-cell receptor-associated protein 29
1,00	ASCC3	activating signal cointegrator 1 complex subunit 3
1,00	NAA60	N(alpha)-acetyltransferase 60, NatF catalytic subunit
1,00	FGF13; LINC00889	fibroblast growth factor 13; long intergenic non-protein coding RNA 889
1,00	NIPAL2	NIPA-like domain containing 2
1,00	HMOX2	heme oxygenase 2
1,00	YWHAG	tyrosine 3-monooxygenase/tryptophan 5-monooxygenase activation protein, gamma
1,00	OAZ1	ornithine decarboxylase antizyme 1
1,00	ZNF527	zinc finger protein 527
1,00	ABCG2	ATP binding cassette subfamily G member 2 (Junior blood group)
1,00	PTPRCAP	protein tyrosine phosphatase, receptor type, C-associated protein
1,00	UBR3	ubiquitin protein ligase E3 component n-recognin 3 (putative)
1,00	EZR	ezrin
1,00	PHACTR3	Transcript Identified by AceView, Entrez Gene ID(s) 116154
1,00	GATB	glutamyl-tRNA(Gln) amidotransferase, subunit B
1,00	GC	group-specific component (vitamin D binding protein)
1,00	PRR20D; PRR20B	proline rich 20D; proline rich 20B
1,00	PRR20D; PRR20B	proline rich 20D; proline rich 20B
1,00	PRR20C; PRR20E	proline rich 20C; proline rich 20E

1,00	PRR20B; PRR20E; PRR20A; PRR20D	proline rich 20B; proline rich 20E; proline rich 20A; proline rich 20D
1,00	PRR20C; PRR20D	proline rich 20C; proline rich 20D
1,00	OSTC	oligosaccharyltransferase complex subunit (non-catalytic)
1,00	PLSCR4	phospholipid scramblase 4
1,00	SLIT1; ARHGAP19-SLIT1	slit guidance ligand 1; ARHGAP19-SLIT1 readthrough (NMD candidate)
1,00	SNRPB2	small nuclear ribonucleoprotein polypeptide B
1,00	TATDN1; MIR6844	TatD DNase domain containing 1; microRNA 6844
1,00	ACSL3	acyl-CoA synthetase long-chain family member 3
1,00	FEN1	flap structure-specific endonuclease 1
1,00	RPS6KA3	ribosomal protein S6 kinase, 90kDa, polypeptide 3
1,00	BCL6	B-cell CLL/lymphoma 6
1,00	GINS4	GINS complex subunit 4 (Sld5 homolog)
1,00	BLOC1S2	biogenesis of lysosomal organelles complex-1, subunit 2
1,00	IFI44L	interferon-induced protein 44-like
1,00	ABI1	abl-interactor 1
1,00	MRPL42	mitochondrial ribosomal protein L42
1,00	R3HDM1	R3H domain containing 1
1,00	POTED	POTE ankyrin domain family, member D
1,00	ERICH3	glutamate rich 3
1,00	PPAP2B	Transcript Identified by AceView, Entrez Gene ID(s) 8613
1,00	RNF14	ring finger protein 14
1,00	KMO	kynurenine 3-monooxygenase (kynurenine 3-hydroxylase)
1,00	PRPH	peripherin
1,00	C5orf45	chromosome 5 open reading frame 45
1,00	HEXB	hexosaminidase B (beta polypeptide)
1,00	LIPE	lipase, hormone-sensitive
1,00	ACSM1	acyl-CoA synthetase medium-chain family member 1
1,00	TCEAL3	transcription elongation factor A (SII)-like 3
1,00	APBA1	amyloid beta (A4) precursor protein-binding, family A, member 1
1,00	MS4A4A	membrane-spanning 4-domains, subfamily A, member 4A
1,00	NDUFAB1	NADH dehydrogenase (ubiquinone) 1, alpha/beta subcomplex, 1, 8kDa
1,00	ZNF180	zinc finger protein 180
1,00	PSAT1	phosphoserine aminotransferase 1
1,00	OR6C4	olfactory receptor, family 6, subfamily C, member 4
1,00	SSR3	signal sequence receptor, gamma (translocon-associated protein gamma)
1,00	USP19	ubiquitin specific peptidase 19

1,00	CCK	cholecystokinin
1,00	BOLA2; BOLA2B	bolA family member 2; bolA family member 2B
1,00	OR1I1	olfactory receptor, family 1, subfamily I, member 1
1,00	PSMB7	proteasome subunit beta 7
1,00	AURKA	aurora kinase A
1,00	PPP1CC	protein phosphatase 1, catalytic subunit, gamma isozyme
1,00	NXF3	nuclear RNA export factor 3
1,00	REXO4	REX4 homolog, 3'-5' exonuclease [Source:HGNC Symbol;Acc:HGNC:12820]
1,00	ADRB2	adrenoceptor beta 2, surface
1,00	STX1B	syntaxin 1B
1,00	PRAMEF10	PRAME family member 10
1,00	C19orf66	chromosome 19 open reading frame 66
1,00	JUNB	jun B proto-oncogene
1,00	SMUG1	single-strand-selective monofunctional uracil-DNA glycosylase 1
1,00	ZNF780B	zinc finger protein 780B
1,00	ZNF780B	zinc finger protein 780B
1,00	IGF1R	insulin-like growth factor 1 receptor
1,00	ACTR2	ARP2 actin-related protein 2 homolog (yeast)
1,00	DDX18	DEAD (Asp-Glu-Ala-Asp) box polypeptide 18
1,00	ZDHHC5	zinc finger, DHHC-type containing 5
1,00	FAM173A	family with sequence similarity 173, member A
1,00	TWIST2	twist family bHLH transcription factor 2
1,00	GSX2	GS homeobox 2
1,00	DDX24	DEAD (Asp-Glu-Ala-Asp) box helicase 24
1,00	IL1RAPL2	interleukin 1 receptor accessory protein-like 2
1,00	MCU	mitochondrial calcium uniporter
1,00	DBN1	drebrin 1
1,00	TRAF3	TNF receptor-associated factor 3
1,00	EXOSC5	exosome component 5
1,00	ETNK1	ethanolamine kinase 1
1,00	PLA2G4D	phospholipase A2, group IVD (cytosolic)
1,00	RPN1	ribophorin I
1,00	ACBD5	acyl-CoA binding domain containing 5
1,00	TRAPPC4	trafficking protein particle complex 4
1,00	RPN2	ribophorin II
1,00	TTLL6	tubulin tyrosine ligase-like family member 6
1,00	ATP8B2	ATPase, aminophospholipid transporter, class I, type 8B, member 2
1,00	MRPS10	mitochondrial ribosomal protein S10
1,00	LYZL1	lysozyme-like 1
1,00	NDUFS4	NADH dehydrogenase (ubiquinone) Fe-S protein 4, 18kDa (NADH-coenzyme Q reductase)
1,00	GCNT2	glucosaminyl (N-acetyl) transferase 2, I-branching enzyme (I blood group)
1,00	PITPNB	phosphatidylinositol transfer protein, beta
1,00	TMEM168	transmembrane protein 168

1,00	ARHGEF38	Rho guanine nucleotide exchange factor 38
1,00	EHMT2	euchromatic histone-lysine N-methyltransferase 2
1,00	SORBS2	sorbin and SH3 domain containing 2
1,00	TMEM179	transmembrane protein 179
1,00	ANKLE1	ankyrin repeat and LEM domain containing 1
1,00	ALDH18A1	aldehyde dehydrogenase 18 family, member A1
1,00	CNTNAP3B	contactin associated protein-like 3B
1,00	NOL3	nucleolar protein 3 (apoptosis repressor with CARD domain)
1,00	DEPTOR	DEP domain containing MTOR-interacting protein
1,00	NDUFA10	NADH dehydrogenase (ubiquinone) 1 alpha subcomplex, 10, 42kDa
1,00	NPLOC4	NPL4 homolog, ubiquitin recognition factor
1,00	SLC22A13	solute carrier family 22 (organic anion/urate transporter), member 13
1,00	EIF5B	eukaryotic translation initiation factor 5B
1,00	sep-10	septin 10
1,00	DDX20	DEAD (Asp-Glu-Ala-Asp) box polypeptide 20
1,00	ZBTB40	zinc finger and BTB domain containing 40
1,00	HOXC9	homeobox C9
1,00	CMYA5	cardiomyopathy associated 5
1,00	CD96	CD96 molecule
1,00	ZNF354B	Zhang2013 ALT_ACCEPTOR, ALT_DONOR, coding, INTERNAL, intronic best transcript NM_058230
1,00	JMJD1C	jumonji domain containing 1C
1,00	TICRR	TOPBP1-interacting checkpoint and replication regulator
1,00	ISPD	isoprenoid synthase domain containing
1,00	ZDHHC3	zinc finger, DHHC-type containing 3
1,00	ACADSB	acyl-CoA dehydrogenase, short/branched chain
1,00	KRAS	Kirsten rat sarcoma viral oncogene homolog
1,00	PUS1	pseudouridylate synthase 1
1,00	DIP2B	disco-interacting protein 2 homolog B
1,00	ZNF485	zinc finger protein 485
1,00	SP1	Sp1 transcription factor
1,00	VTI1B	vesicle transport through interaction with t-SNAREs 1B
1,00	KRTAP12-4	keratin associated protein 12-4
1,00	MED14	mediator complex subunit 14
1,00	MGC57346-CRHR1; RP11-105N13.4	Homo sapiens MGC57346-CRHR1 readthrough (MGC57346-CRHR1), transcript variant 5, mRNA.; Homo sapiens MGC57346-CRHR1 readthrough (MGC57346-CRHR1), transcript variant 6, mRNA.; novel transcript
1,00	OR5G5P; OR5G3	olfactory receptor, family 5, subfamily G, member 5 pseudogene; olfactory receptor, family 5, subfamily G, member 3 (gene/pseudogene)
1,00	HLTF	helicase-like transcription factor
1,00	CEP170B	centrosomal protein 170B

1,00	C17orf112	chromosome 17 open reading frame 112
1,00	SRBD1	S1 RNA binding domain 1
1,00	SMPD2	sphingomyelin phosphodiesterase 2, neutral membrane (neutral sphingomyelinase)
1,00	ATXN7L1	ataxin 7-like 1
1,00	HAUS8	HAUS augmin like complex subunit 8
1,00	GTF2B	general transcription factor IIB
1,00	TMEM183A	transmembrane protein 183A
1,00	MXI1	MAX interactor 1, dimerization protein
1,00	IGF2BP1	insulin-like growth factor 2 mRNA binding protein 1
1,00	TRIM32	tripartite motif containing 32
1,00	PGA5	pepsinogen 5, group I (pepsinogen A)
1,00	TRMT13	tRNA methyltransferase 13 homolog (S. cerevisiae)
1,00	NWD2	NACHT and WD repeat domain containing 2
1,00	P2RX5-TAX1BP3	P2RX5-TAX1BP3 readthrough (NMD candidate)
1,00	SNRNP70	small nuclear ribonucleoprotein, U1 70kDa subunit
1,00	CD300LB	CD300 molecule-like family member b
1,00	CDX2	caudal type homeobox 2
1,00	AQP11	aquaporin 11
1,00	SCRN2	secernin 2
1,00	DHFR	dihydrofolate reductase
1,00	CST11	cystatin 11
1,00	ZPR1	ZPR1 zinc finger
1,00	SMOX	spermine oxidase
1,00	UBE4A	ubiquitination factor E4A
1,00	SLC52A2	solute carrier family 52 (riboflavin transporter), member 2
1,00	MED12L	mediator complex subunit 12 like
1,00	boyboy; RP4-630A11.3; LEPR	Transcript Identified by AceView; Jeck2013 ALT_ACCEPTOR, ALT_DONOR, coding, INTERNAL, intronic best transcript NM_001003679; putative novel transcript
1,00	RNF103-CHMP3; CHMP3	RNF103-CHMP3 readthrough; charged multivesicular body protein 3
1,00	IL1RAP	interleukin 1 receptor accessory protein
1,00	CBWD1	COBW domain containing 1
1,00	ST8SIA3	ST8 alpha-N-acetyl-neuraminide alpha-2,8-sialyltransferase 3
1,00	TBC1D1	TBC1 (tre-2/USP6, BUB2, cdc16) domain family, member 1
1,00	SUCLA2	succinate-CoA ligase, ADP-forming, beta subunit
1,00	SMPDL3B	sphingomyelin phosphodiesterase, acid-like 3B
1,00	CDH7	cadherin 7, type 2
1,00	OSBPL11	oxysterol binding protein-like 11
1,00	GSTA4	glutathione S-transferase alpha 4
1,00	HHAT	hedgehog acyltransferase
1,00	CDK7	cyclin-dependent kinase 7
1,00	GPC6	glypican 6

1,00	UPK3BL; POLR2J2	uroplakin 3B-like; polymerase (RNA) II (DNA directed) polypeptide J2
1,00	RBL1	retinoblastoma-like 1
1,00	CDCA4	cell division cycle associated 4
1,00	LOC102724279; AC004791.2; nimure	uncharacterized LOC102724279; Transcript Identified by AceView; novel transcript
1,00	PODXL	podocalyxin-like
1,00	PLBD2	phospholipase B domain containing 2
1,00	FZD7	frizzled class receptor 7
1,00	PSG4	pregnancy specific beta-1-glycoprotein 4
1,00	LINC00668	long intergenic non-protein coding RNA 668
1,00	SNORD102; SNORA27; RPL21	small nucleolar RNA, C/D box 102; small nucleolar RNA, H/ACA box 27; ribosomal protein L21
1,00	AGAP1	ArfGAP with GTPase domain, ankyrin repeat and PH domain 1
1,00	IRAK3; MIR6502	interleukin 1 receptor associated kinase 3; microRNA 6502
1,00	HDGFRP3	hepatoma-derived growth factor, related protein 3; Hepatoma-derived growth factor-related protein 3 [Source:UniProtKB/Swiss-Prot;Acc:Q9Y3E1]; Transcript Identified by AceView, Entrez Gene ID(s) 50810; Jeck2013, Memczak2013, Salzman2013 ANNOTATED, CDS, coding, INTERNAL, OVCODE, OVEXON best transcript NM_016073; Jeck2013 ALT_ACCEPTOR, ALT_DONOR, coding, INTERNAL, intronic best transcript NM_016073; Salzman2013 ANNOTATED, CDS, coding, INTERNAL, OVCODE, OVEXON best transcript NM_016073
1,00	TMEM101	transmembrane protein 101
1,00	GPR21; RABGAP1	G protein-coupled receptor 21; RAB GTPase activating protein 1
1,00	SPRTN	SprT-like N-terminal domain
1,00	SMC3	structural maintenance of chromosomes 3
1,00	C1orf159	chromosome 1 open reading frame 159
1,00	DAPP1	dual adaptor of phosphotyrosine and 3-phosphoinositides
1,00	RNF185	ring finger protein 185
1,00	NDUFAF6	NADH dehydrogenase (ubiquinone) complex I, assembly factor 6
1,00	NDUFS1; GCSHP3	NADH dehydrogenase (ubiquinone) Fe-S protein 1, 75kDa (NADH-coenzyme Q reductase); glycine cleavage system protein H (aminomethyl carrier) pseudogene 3
1,00	CACNA1B	calcium channel, voltage-dependent, N type, alpha 1B subunit
1,00	RNF130	ring finger protein 130
1,00	H1FOO	H1 histone family, member O, oocyte-specific

1,00	LRRC16A	Transcript Identified by AceView, Entrez Gene ID(s) 55604
1,00	FH	fumarate hydratase
1,00	LATS2	large tumor suppressor kinase 2
1,00	IFITM2	interferon induced transmembrane protein 2
1,00	C18orf21	chromosome 18 open reading frame 21
1,00	MRPS33	mitochondrial ribosomal protein S33
1,00	MAGEA2; MAGEA2B	MAGE family member A2; MAGE family member A2B
1,00	C10orf10	chromosome 10 open reading frame 10
1,00	SERPING1	serpin peptidase inhibitor, clade G (C1 inhibitor), member 1
1,00	PSMC1	proteasome 26S subunit, ATPase 1
1,00	METTL14	methyltransferase like 14
1,00	GYPA	glycophorin A (MNS blood group)
1,00	SHROOM2	shroom family member 2
1,00	CT45A5	cancer/testis antigen family 45, member A5
1,00	PFDN1	prefoldin subunit 1
1,00	ANXA2	annexin A2
1,00	ANAPC15	anaphase promoting complex subunit 15
1,00	OR2J2	olfactory receptor, family 2, subfamily J, member 2
1,00	ACOT6	acyl-CoA thioesterase 6
1,00	PRR5L	proline rich 5 like
1,00	PDXDC2P	pyridoxal-dependent decarboxylase domain containing 2, pseudogene
1,00	PHACTR4	phosphatase and actin regulator 4
1,00	OSBPL10	oxysterol binding protein-like 10
1,00	HSD17B4	hydroxysteroid (17-beta) dehydrogenase 4
1,00	SSSCA1	Sjogren syndrome/scleroderma autoantigen 1
1,00	MORF4L2	mortality factor 4 like 2
1,00	PCGF2	polycomb group ring finger 2
1,00	ATG9A	autophagy related 9A
1,00	ZMYND8	zinc finger, MYND-type containing 8
1,00	EPB41	Memczak2013 ALT_ACCEPTOR, ALT_DONOR, coding, INTERNAL, intronic best transcript NM_001166007
1,00	SLC9C1	solute carrier family 9, subfamily C (Na+-transporting carboxylic acid decarboxylase), member 1
1,00	RGAG1	retrotransposon gag domain containing 1
1,00	RPS6KA1	ribosomal protein S6 kinase, 90kDa, polypeptide 1
1,00	ADCY3	adenylate cyclase 3
1,00	PIGV	phosphatidylinositol glycan anchor biosynthesis class V
1,00	ZNF613	zinc finger protein 613
1,00	LIFR	leukemia inhibitory factor receptor alpha
1,00	ZNF197	zinc finger protein 197
1,00	HAT1	histone acetyltransferase 1
1,00	METTL2B	methyltransferase like 2B
1,00	ZNF737	zinc finger protein 737

1,00	METTL9	methyltransferase like 9
1,00	PQBP1	polyglutamine binding protein 1
1,00	AARD	alanine and arginine rich domain containing protein
1,00	TMEM89	transmembrane protein 89
1,00	TSKU	tsukushi, small leucine rich proteoglycan
1,00	DNAJC1	DnaJ (Hsp40) homolog, subfamily C, member 1
1,00	PPHLN1	periphilin 1
1,00	SLC10A7	solute carrier family 10, member 7
1,00	LOC389602; AC021218.2	uncharacterized LOC389602; novel transcript
1,00	FAM205C	family with sequence similarity 205, member C
1,00	AURKB	aurora kinase B
1,00	CNBP	CCHC-type zinc finger, nucleic acid binding protein
1,00	TBC1D3L	TBC1 domain family, member 3L
1,00	PPP1R42	protein phosphatase 1, regulatory subunit 42
1,00	MBOAT1	membrane bound O-acyltransferase domain containing 1
1,00	DEPDC7	DEP domain containing 7
1,00	NMRK2	nicotinamide riboside kinase 2
1,00	RNF166	ring finger protein 166
1,00	CCDC137	coiled-coil domain containing 137
1,00	C4orf32	chromosome 4 open reading frame 32
1,00	FTCD	formimidoyltransferase cyclodeaminase
1,00	ZNF780B	zinc finger protein 780B
1,00	TTYH1	tweety family member 1
1,00	TMEM209	transmembrane protein 209
1,00	RPS2; SNORA64; SNORA10	ribosomal protein S2; small nucleolar RNA, H/ACA box 64; small nucleolar RNA, H/ACA box 10
1,00	PPP4R2	protein phosphatase 4, regulatory subunit 2
1,00	MPZL3	myelin protein zero-like 3
1,00	NIPSNAP1	nipsnap homolog 1 (C. elegans)
1,00	SLC25A36	solute carrier family 25 (pyrimidine nucleotide carrier), member 36
1,00	OR2M7	olfactory receptor, family 2, subfamily M, member 7
1,00	PPP3CA	protein phosphatase 3, catalytic subunit, alpha isozyme
1,00	DEFB133	defensin, beta 133
1,00	TTC38	Memczak2013 ALT_ACCEPTOR, ALT_DONOR, coding, INTERNAL, intronic best transcript NM_017931
1,00	CCL25	chemokine (C-C motif) ligand 25
1,00	GTSF1	gametocyte specific factor 1
1,00	ABHD17B	abhydrolase domain containing 17B
1,00	CD74	CD74 molecule, major histocompatibility complex, class II invariant chain
1,00	PVR	poliovirus receptor
1,00	OR1S1	olfactory receptor, family 1, subfamily S, member 1 (gene/pseudogene)
1,00	YIPF5	Yip1 domain family member 5

1,00	PLIN2	perilipin 2
1,00	DFNB31	deafness, autosomal recessive 31
1,00	ZNF471	zinc finger protein 471
1,00	KIAA0895L	KIAA0895-like
1,00	C8orf33	chromosome 8 open reading frame 33
1,00	HDAC2	histone deacetylase 2
1,00	HMGN2	high mobility group nucleosomal binding domain 2
1,00	WDR35	WD repeat domain 35
1,00	SOX5	SRY box 5
1,00	SNCG	synuclein gamma
1,00	IPO7; SNORA23	importin 7; small nucleolar RNA, H/ACA box 23
1,00	ADAD2	adenosine deaminase domain containing 2
1,00	ZER1	zyg-11 related, cell cycle regulator
1,00	VTCN1	V-set domain containing T cell activation inhibitor 1
1,00	STAT2	signal transducer and activator of transcription 2
1,00	SLC22A4	solute carrier family 22 (organic cation/zwitterion transporter), member 4
1,00	GART	phosphoribosylglycinamide formyltransferase, phosphoribosylglycinamide synthetase, phosphoribosylaminoimidazole synthetase
1,00	OR52I1	olfactory receptor, family 52, subfamily I, member 1
1,00	PHRF1	PHD and ring finger domains 1
1,00	SCN2A	sodium channel, voltage gated, type II alpha subunit
1,00	ADGRB2	adhesion G protein-coupled receptor B2
1,00	MROH5	maestro heat-like repeat family member 5
1,00	PFDN2	prefoldin subunit 2
1,00	NPIPB5	nuclear pore complex interacting protein family, member B5
1,00	CT45A10	cancer/testis antigen family 45, member A10
1,00	CCDC51	coiled-coil domain containing 51
1,00	USP15; MIR6125	ubiquitin specific peptidase 15; microRNA 6125
1,00	EBF2	early B-cell factor 2
1,00	HOPX	HOP homeobox
1,00	SOX5	Transcript Identified by AceView, Entrez Gene ID(s) 6660
1,00	MUC2	mucin 2, oligomeric mucus/gel-forming
1,00	CRIP1	cysteine-rich protein 1 (intestinal)
1,00	LAX1	lymphocyte transmembrane adaptor 1
1,00	GOLGA8J; GOLGA8IP	golgin A8 family, member J; golgin A8 family, member I, pseudogene
1,00	FGD5	FYVE, RhoGEF and PH domain containing 5
1,00	CLDN25	claudin 25
1,00	RBMX2	RNA binding motif protein, X-linked 2
1,00	UBXN2B	UBX domain protein 2B
1,00	WEE1	WEE1 G2 checkpoint kinase
1,00	AMELY	amelogenin, Y-linked
1,00	CXCR5	chemokine (C-X-C motif) receptor 5
1,00	GCNT7	glucosaminyl (N-acetyl) transferase family member 7
1,00	DUS1L	dihydrouridine synthase 1-like
1,00	TNRC6A	trinucleotide repeat containing 6A

1,00	ABRACL	ABRA C-terminal like
1,00	ARHGDIG	Rho GDP dissociation inhibitor (GDI) gamma
1,00	IL5RA	interleukin 5 receptor, alpha
1,00	HMBOX1	homeobox containing 1
1,00	CDYL	chromodomain protein, Y-like
1,00	SYN3	synapsin III
1,00	BCS1L	BCS1 homolog, ubiquinol-cytochrome c reductase complex chaperone
1,00	EIF2AK2	eukaryotic translation initiation factor 2-alpha kinase 2
1,00	OLIG3	oligodendrocyte transcription factor 3
1,00	NR3C1	nuclear receptor subfamily 3, group C, member 1 (glucocorticoid receptor)
1,00	EPPIN-WFDC6	EPPIN-WFDC6 readthrough
1,00	CPSF3L; MIR6727	cleavage and polyadenylation specific factor 3-like; microRNA 6727
1,00	KCNH2	potassium channel, voltage gated eag related subfamily H, member 2
1,00	KCNAB2	potassium channel, voltage gated subfamily A regulatory beta subunit 2
1,00	PDCD2	programmed cell death 2
1,00	DGKD	diacylglycerol kinase, delta 130kDa
1,00	KPNA2	karyopherin alpha 2 (RAG cohort 1, importin alpha 1)
1,00	RPS6KA1	ribosomal protein S6 kinase, 90kDa, polypeptide 1
1,00	GALNT5	polypeptide N-acetylgalactosaminyltransferase 5
1,00	UAP1	UDP-N-acetylglucosamine pyrophosphorylase 1
1,00	OCIAD1	Transcript Identified by AceView, Entrez Gene ID(s) 54940
1,00	IKZF2	IKAROS family zinc finger 2
1,00	XBP1	X-box binding protein 1
1,00	VPS29	VPS29 retromer complex component
1,00	EPB41L4A-AS2	EPB41L4A antisense RNA 2 (head to head)
1,00	DCLK3	doublecortin-like kinase 3
1,00	KIAA1614	KIAA1614
1,00	FAM50B	family with sequence similarity 50, member B
1,00	VPS25	vacuolar protein sorting 25 homolog (S. cerevisiae)
1,00	SSU72	SSU72 homolog, RNA polymerase II CTD phosphatase
1,00	MANSC1	MANSC domain containing 1
1,00	CHD5	chromodomain helicase DNA binding protein 5
1,00	SUZ12	SUZ12 polycomb repressive complex 2 subunit
1,00	VCP	valosin containing protein
1,00	RNF139	ring finger protein 139
1,00	CORO1B	coronin, actin binding protein, 1B
1,00	GSK3A	glycogen synthase kinase 3 alpha
1,00	MAN1B1	mannosidase, alpha, class 1B, member 1
1,00	MAGEA10-MAGEA5; RP11-1007I13.4	MAGEA10-MAGEA5 readthrough; novel transcript
1,00	TMEM65	transmembrane protein 65

1,00	TP53BP2	tumor protein p53 binding protein 2
1,00	DUSP9	dual specificity phosphatase 9
1,00	DDX19B	DEAD (Asp-Glu-Ala-Asp) box polypeptide 19B
1,00	FAM72B	family with sequence similarity 72, member B
1,00	ADH6	alcohol dehydrogenase 6 (class V)
1,00	ATP2C1	ATPase, Ca++ transporting, type 2C, member 1
1,00	RCCD1	RCC1 domain containing 1
1,00	SLC9A3R1; MIR3615	solute carrier family 9, subfamily A (NHE3, cation proton antiporter 3), member 3 regulator 1; microRNA 3615
1,00	RPS6KA1	ribosomal protein S6 kinase, 90kDa, polypeptide 1
1,00	GJB7	gap junction protein beta 7
1,00	IQCB1	IQ motif containing B1
1,00	DGKQ	diacylglycerol kinase theta
1,00	ZFY	zinc finger protein, Y-linked
1,00	VMP1; MIR21	vacuole membrane protein 1; microRNA 21
1,00	NPFFR1	neuropeptide FF receptor 1
1,00	ALDH1A1	aldehyde dehydrogenase 1 family, member A1
1,00	B4GAT1	beta-1,4-glucuronyltransferase 1
1,00	KIF20B	kinesin family member 20B
1,00	REN	renin
1,00	YME1L1	YME1-like 1 ATPase
1,00	SLC2A5	solute carrier family 2 (facilitated glucose/fructose transporter), member 5
1,00	SPANXB1	SPANX family, member B1
1,00	PWP2	PWP2 periodic tryptophan protein homolog (yeast)
1,00	RAD51C	RAD51 paralog C
1,00	CCPG1; MIR628	cell cycle progression 1; microRNA 628
1,00	DCN	decorin
1,00	LRRC27	leucine rich repeat containing 27
1,00	RBM15	RNA binding motif protein 15
1,00	DNAI1	Transcript Identified by AceView, Entrez Gene ID(s) 27019
1,00	SNX4	sorting nexin 4
1,00	MRPL28	mitochondrial ribosomal protein L28
1,00	ZNF202	zinc finger protein 202
1,00	MAGEC1	MAGE family member C1
1,00	PFKP	phosphofructokinase, platelet
1,00	TMEM261	transmembrane protein 261
1,00	TMEM91	transmembrane protein 91
1,00	NFX1	nuclear transcription factor, X-box binding 1
1,00	SPHK1	sphingosine kinase 1
1,00	FXR1	fragile X mental retardation, autosomal homolog 1
1,00	ATP6V1B2	ATPase, H+ transporting, lysosomal 56/58kDa, V1 subunit B2
1,00	RPL29	ribosomal protein L29
1,00	KRTAP19-4	keratin associated protein 19-4
1,00	MACROD1	MACRO domain containing 1
1,00	FAM47A	family with sequence similarity 47, member A
1,00	DOK6	docking protein 6

1,00	FKBP7	FK506 binding protein 7
1,00	EFHC1	EF-hand domain (C-terminal) containing 1
1,00	TPT1; SNORA31	tumor protein, translationally-controlled 1; small nucleolar RNA, H/ACA box 31
1,00	APOH	apolipoprotein H (beta-2-glycoprotein I)
1,00	PABPC4	poly(A) binding protein, cytoplasmic 4 (inducible form)
1,00	COPS4	COP9 signalosome subunit 4
1,00	TAS2R8	taste receptor, type 2, member 8
1,00	WDR55	WD repeat domain 55
1,00	MAGOHB	mago homolog B, exon junction complex core component
1,00	STK38	serine/threonine kinase 38
1,00	PDS5B	PDS5 cohesin associated factor B
1,00	CLEC16A	C-type lectin domain family 16, member A
1,00	NCAPG	non-SMC condensin I complex subunit G
1,00	NDUFB2	NADH dehydrogenase (ubiquinone) 1 beta subcomplex, 2, 8kDa
1,00	CWC22	CWC22 homolog, spliceosome-associated protein
1,00	AP4B1	adaptor-related protein complex 4, beta 1 subunit
1,00	TAC4	tachykinin 4 (hemokinin)
1,00	ORC1	origin recognition complex subunit 1
1,00	PLPP5	phospholipid phosphatase 5
1,00	HCAR3	hydroxycarboxylic acid receptor 3
1,00	LRIG3	leucine-rich repeats and immunoglobulin-like domains 3
1,00	ZDHHC3	zinc finger, DHHC-type containing 3
1,00	KRT16	keratin 16, type I
1,00	CLVS1	clavesin 1
1,00	LINC01270	long intergenic non-protein coding RNA 1270
1,00	VPS18	VPS18 CORVET/HOPS core subunit
1,00	HYAL2	hyaluronoglucosaminidase 2
1,00	FCRL2	Fc receptor-like 2
1,00	CHST5	carbohydrate (N-acetylglucosamine 6-O) sulfotransferase 5
1,00	GSPT2	G1 to S phase transition 2
1,00	THUMPD3	THUMP domain containing 3
1,00	GNAQ	guanine nucleotide binding protein (G protein), q polypeptide
1,00	TSKS	testis-specific serine kinase substrate
1,00	LRRC37B	leucine rich repeat containing 37B
1,00	NUP160	nucleoporin 160kDa
1,00	KCNMB4	potassium channel subfamily M regulatory beta subunit 4
1,00	UBE2K	ubiquitin conjugating enzyme E2K
1,00	FFAR1	free fatty acid receptor 1
1,00	OR11H6	olfactory receptor, family 11, subfamily H, member 6
1,00	ZDHHC4	zinc finger, DHHC-type containing 4
1,00	ADPGK	ADP-dependent glucokinase
1,00	MYL6B	myosin light chain 6B

1,00	RND1	Rho family GTPase 1
1,00	MTX3	metaxin 3
1,00	SMLR1	small leucine-rich protein 1
1,00	SLC38A9	Transcript Identified by AceView, Entrez Gene ID(s) 153129
1,00	LRFN4	leucine rich repeat and fibronectin type III domain containing 4
1,00	BSG	basigin (Ok blood group)
1,00	PVRL1	poliovirus receptor-related 1 (herpesvirus entry mediator C)
1,00	COPB2	coatomer protein complex subunit beta 2 (beta prime)
1,00	EPHB6	EPH receptor B6
1,00	STOX2	storkhead box 2
1,00	ACSF2	acyl-CoA synthetase family member 2
1,00	PRRC1	proline-rich coiled-coil 1
1,00	LOC338797; RP13-507P19.2; RP13-507P19.1	uncharacterized LOC338797; novel transcript
0,99	LOC100130370; RP11-1055B8.3	uncharacterized LOC100130370; novel transcript
0,99	PRODH2	proline dehydrogenase (oxidase) 2
0,99	BCL9	B-cell CLL/lymphoma 9
0,99	C15orf40	chromosome 15 open reading frame 40
0,99	THPO	thrombopoietin
0,99	OR2A42; OR2A1	olfactory receptor, family 2, subfamily A, member 42; olfactory receptor, family 2, subfamily A, member 1
0,99	C9orf173	chromosome 9 open reading frame 173
0,99	PRAMEF18	PRAME family member 18
0,99	NAA16	N(alpha)-acetyltransferase 16, NatA auxiliary subunit
0,99	TIPARP	TCDD-inducible poly(ADP-ribose) polymerase
0,99	LOC728554; THOC3	THO complex 3 pseudogene; Homo sapiens THO complex 3, mRNA (cDNA clone MGC:5469 IMAGE:3451612), complete cds.; Homo sapiens THO complex 3, mRNA (cDNA clone MGC:87233 IMAGE:5262880), complete cds.; Salzman2013 ANNOTATED, INTERNAL, ncRNA, OVERLAPTX, OVEXON best transcript NR_003615; Transcript Identified by AceView, Entrez Gene ID(s) 728554
0,99	PRSS8	protease, serine, 8
0,99	C6orf223	chromosome 6 open reading frame 223
0,99	NDUFAF7	NADH dehydrogenase (ubiquinone) complex I, assembly factor 7
0,99	SENP3-EIF4A1	SENP3-EIF4A1 readthrough (NMD candidate)
0,99	ADAMTS18	ADAM metallopeptidase with thrombospondin type 1 motif 18
0,99	TBX21	T-box 21
0,99	PYGL	phosphorylase, glycogen, liver
0,99	PRPF40A	PRP40 pre-mRNA processing factor 40 homolog A
0,99	PNMT	phenylethanolamine N-methyltransferase

0,99	ABTB1	ankyrin repeat and BTB (POZ) domain containing 1
0,99	CRTC1	CREB regulated transcription coactivator 1
0,99	GAMT	guanidinoacetate N-methyltransferase
0,99	PTRF	polymerase I and transcript release factor
0,99	NUDT6	nudix hydrolase 6
0,99	ZNF579	zinc finger protein 579
0,99	HMG20A	Transcript Identified by AceView, Entrez Gene ID(s) 10363
0,99	TECTA	tectorin alpha
0,99	ANKRD33	ankyrin repeat domain 33
0,99	ANXA2R	annexin A2 receptor
0,99	TAX1BP3	Tax1 (human T-cell leukemia virus type I) binding protein 3
0,99	B4GALNT4	beta-1,4-N-acetyl-galactosaminyl transferase 4
0,99	C1orf27	chromosome 1 open reading frame 27
0,99	NOTUM	notum pectinacetylesterase homolog (Drosophila)
0,99	ARHGAP36	Rho GTPase activating protein 36
0,99	SV2C	synaptic vesicle glycoprotein 2C
0,99	IGF2	insulin-like growth factor 2
0,99	RABAC1	Rab acceptor 1 (prenylated)
0,99	WDR82	WD repeat domain 82
0,99	RCSD1	RCSD domain containing 1
0,99	GID8	GID complex subunit 8
0,99	CHAD	chondroadherin
0,99	RBM10	RNA binding motif protein 10
0,99	DYNLRB2	dynein, light chain, roadblock-type 2
0,99	ZNF148	zinc finger protein 148
0,99	GPR35	G protein-coupled receptor 35
0,99	OR10AG1	olfactory receptor, family 10, subfamily AG, member 1
0,99	PAPOLG	poly(A) polymerase gamma
0,99	MANSC1	MANSC domain containing 1
0,99	OR51E1	olfactory receptor, family 51, subfamily E, member 1
0,99	MTMR14	myotubularin related protein 14
0,99	NDUFA6	NADH dehydrogenase (ubiquinone) 1 alpha subcomplex, 6, 14kDa
0,99	DACT1	dishevelled-binding antagonist of beta-catenin 1
0,99	PGS1	phosphatidylglycerophosphate synthase 1
0,99	FASLG	Fas ligand (TNF superfamily, member 6)
0,99	TTC39B	tetratricopeptide repeat domain 39B
0,99	COL23A1	collagen, type XXIII, alpha 1
0,99	SPEG	SPEG complex locus
0,99	RNASEH2B	ribonuclease H2, subunit B
0,99	DCUN1D5	DCN1, defective in cullin neddylation 1, domain containing 5
0,99	LRFN5	leucine rich repeat and fibronectin type III domain containing 5
0,99	LRRC29	leucine rich repeat containing 29
0,99	OPRD1	opioid receptor, delta 1
0,99	SPATA31D4	SPATA31 subfamily D, member 4

0,99	SRSF9	serine/arginine-rich splicing factor 9
0,99	LINC01272	long intergenic non-protein coding RNA 1272
0,99	FHAD1	forkhead-associated (FHA) phosphopeptide binding domain 1
0,99	PRAME	preferentially expressed antigen in melanoma
0,99	RNF186	ring finger protein 186
0,99	HIST1H3H	histone cluster 1, H3h
0,99	ZNF155	zinc finger protein 155
0,99	SUV39H2	suppressor of variegation 3-9 homolog 2 (Drosophila)
0,99	CTC-453G23.8; CARD8; nekera	Memczak2013 ANTISENSE, CDS, coding, INTERNAL best transcript NM_014959; Transcript Identified by AceView; novel transcript, antisense to CARD8
0,99	POU2F1	POU class 2 homeobox 1
0,99	YWHAE	tyrosine 3-monooxygenase/tryptophan 5-monooxygenase activation protein, epsilon
0,99	FAM153A	family with sequence similarity 153, member A
0,99	GSC	goosecoid homeobox
0,99	SCRIB; MIR937	scribbled planar cell polarity protein; microRNA 937
0,99	SYNDIG1	synapse differentiation inducing 1
0,99	CLDN22	claudin 22
0,99	GALNT12	polypeptide N-acetylgalactosaminyltransferase 12
0,99	CCDC85A	Transcript Identified by AceView, Entrez Gene ID(s) 114800
0,99	EEF1A2	eukaryotic translation elongation factor 1 alpha 2
0,99	EXOSC6	exosome component 6
0,99	RIN3	Ras and Rab interactor 3
0,99	GHITM	growth hormone inducible transmembrane protein
0,99	TIMMDC1	translocase of inner mitochondrial membrane domain containing 1
0,99	NPEPL1	aminopeptidase-like 1
0,99	GRID2IP	glutamate receptor, ionotropic, delta 2 (Grid2) interacting protein
0,99	CCDC170	coiled-coil domain containing 170
0,99	SEC61G	Sec61 translocon gamma subunit
0,99	ASB11	ankyrin repeat and SOCS box containing 11, E3 ubiquitin protein ligase
0,99	VCX; VCX3A	variable charge, X-linked; variable charge, X-linked 3A
0,99	CRYAA	crystallin alpha A
0,99	NKX2-8	NK2 homeobox 8
0,99	GIMAP4	GTPase, IMAP family member 4
0,99	MTG2	mitochondrial ribosome-associated GTPase 2
0,99	SUGT1	SGT1 homolog, MIS12 kinetochore complex assembly cochaperone
0,99	CPPED1	calcineurin-like phosphoesterase domain containing 1
0,99	DAZ2; DAZ3	deleted in azoospermia 2; deleted in azoospermia 3
0,99	RPRM	reprimo, TP53 dependent G2 arrest mediator candidate
0,99	UTP3	UTP3, small subunit (SSU) processome component, homolog (S. cerevisiae)

0,99	B3GNT3	UDP-GlcNAc:betaGal beta-1,3-N-acetylglucosaminyltransferase 3
0,99	KCND3	potassium channel, voltage gated Shal related subfamily D, member 3
0,99	LOC115110; RP3-395M20.8	uncharacterized LOC115110; novel transcript
0,99	ST18	suppression of tumorigenicity 18, zinc finger
0,99	FZD3	frizzled class receptor 3
0,99	ALDH1A2	aldehyde dehydrogenase 1 family, member A2
0,99	SLC48A1	solute carrier family 48 (heme transporter), member 1
0,99	MS4A3	membrane-spanning 4-domains, subfamily A, member 3 (hematopoietic cell-specific)
0,99	EMC6	ER membrane protein complex subunit 6
0,99	TXNDC9	thioredoxin domain containing 9
0,99	PTPN1	protein tyrosine phosphatase, non-receptor type 1
0,99	DPYD	dihydropyrimidine dehydrogenase
0,99	SFR1	SWI5-dependent homologous recombination repair protein 1
0,99	LIN7A	lin-7 homolog A (C. elegans)
0,99	GALT	galactose-1-phosphate uridylyltransferase
0,99	MAGEA2	MAGE family member A2
0,99	KRT24	keratin 24, type I
0,99	SCG2	secretogranin II
0,99	LPIN2	lipin 2
0,99	FATE1	fetal and adult testis expressed 1
0,99	VWA3A	von Willebrand factor A domain containing 3A
0,99	STAT1	signal transducer and activator of transcription 1
0,99	ZNF207; MIR632	zinc finger protein 207; microRNA 632
0,99	DNAH3	dynein, axonemal, heavy chain 3
0,99	C8orf87	chromosome 8 open reading frame 87
0,99	ST5	suppression of tumorigenicity 5
0,99	DHX29	DEAH (Asp-Glu-Ala-His) box polypeptide 29
0,99	BMS1P20; IGLV1-51; IGLV5-52	BMS1 ribosome biogenesis factor pseudogene 20; immunoglobulin lambda variable 1-51; immunoglobulin lambda variable 5-52
0,99	CA13	carbonic anhydrase XIII
0,99	RDH11	retinol dehydrogenase 11 (all-trans/9-cis/11-cis)
0,99	GREM1	gremlin 1, DAN family BMP antagonist
0,99	INPP5E	inositol polyphosphate-5-phosphatase E
0,99	TCAF2	TRPM8 channel-associated factor 2
0,99	MC1R	melanocortin 1 receptor (alpha melanocyte stimulating hormone receptor)
0,99	ERCC8	excision repair cross-complementation group 8
0,99	GATA4	GATA binding protein 4
0,99	GTF2H5	general transcription factor IIH subunit 5
0,99	PRIM1	primase, DNA, polypeptide 1 (49kDa)
0,99	CALD1	caldesmon 1
0,99	NIP7	NIP7, nucleolar pre-rRNA processing protein
0,99	CEP112	centrosomal protein 112kDa
0,99	CT45A8	cancer/testis antigen family 45, member A8

0,99	CT45A9	cancer/testis antigen family 45, member A9
0,99	HIST1H4C	histone cluster 1, H4c
0,99	UBE2V2	ubiquitin conjugating enzyme E2 variant 2
0,99	RMND5A	required for meiotic nuclear division 5 homolog A
0,99	LOC100131303; CTC-479C5.6	uncharacterized LOC100131303; novel transcript, antisense to DUS2L
0,99	BHLHE40	basic helix-loop-helix family, member e40
0,99	TDP2	tyrosyl-DNA phosphodiesterase 2
0,99	CITED2	Cbp/p300-interacting transactivator, with Glu/Asp rich carboxy-terminal domain, 2
0,99	TUBD1	tubulin, delta 1
0,99	TCF4	Memczak2013 ANTISENSE, coding, INTERNAL, intronic best transcript NM_001243234
0,99	CAAP1	caspase activity and apoptosis inhibitor 1
0,99	PRELID3B; ATP5E	PRELI domain containing 3B; ATP synthase, H+ transporting, mitochondrial F1 complex, epsilon subunit
0,99	CD22; MIR5196	CD22 molecule; microRNA 5196
0,99	ARHGAP11B	Rho GTPase activating protein 11B
0,99	F2RL2	coagulation factor II (thrombin) receptor-like 2
0,99	IL10RB	interleukin 10 receptor, beta
0,99	HAUS6	HAUS augmin like complex subunit 6
0,99	HTR4	5-hydroxytryptamine (serotonin) receptor 4, G protein-coupled
0,99	TRIM49B	tripartite motif containing 49B
0,99	SALL2	spalt-like transcription factor 2
0,99	NRSN2	neurensin 2
0,99	RPL7A	ribosomal protein L7a
0,99	CCDC14	coiled-coil domain containing 14
0,99	GPKOW	G-patch domain and KOW motifs
0,99	FER1L5	fer-1-like family member 5
0,99	CBLN3	cerebellin 3 precursor
0,99	LMCD1; LINC00312	LIM and cysteine-rich domains 1; long intergenic non-protein coding RNA 312
0,99	TM2D2	TM2 domain containing 2
0,99	OR10A3	olfactory receptor, family 10, subfamily A, member 3
0,99	INHA	inhibin alpha
0,99	LSM8	LSM8 homolog, U6 small nuclear RNA associated
0,99	RAB28	RAB28, member RAS oncogene family
0,99	SPA17	sperm autoantigenic protein 17
0,99	ZFP82	ZFP82 zinc finger protein
0,99	TRIQK	triple QxxK/R motif containing
0,99	OR56A4	olfactory receptor, family 56, subfamily A, member 4
0,99	TTC22	tetratricopeptide repeat domain 22
0,99	PPP4R3A	protein phosphatase 4, regulatory subunit 3A
0,99	GTF2IRD1	GTF2I repeat domain containing 1
0,99	RIMS3	regulating synaptic membrane exocytosis 3
0,99	RGL2	ral guanine nucleotide dissociation stimulator-like 2
0,99	EIF4A3	eukaryotic translation initiation factor 4A3
0,99	COMTD1	catechol-O-methyltransferase domain containing 1

0,99	HYOU1	hypoxia up-regulated 1
0,99	NOTO	notochord homeobox
0,99	MTF2	metal response element binding transcription factor 2
0,99	C5	complement component 5
0,99	PSPC1	paraspeckle component 1
0,99	SLC52A1	solute carrier family 52 (riboflavin transporter), member 1
0,99	CAMK2A	calcium/calmodulin-dependent protein kinase II alpha
0,99	IER5L	immediate early response 5-like
0,99	CHRNA3	cholinergic receptor, nicotinic alpha 3
0,99	PTK6	protein tyrosine kinase 6
0,99	FXR2	fragile X mental retardation, autosomal homolog 2
0,99	C17orf62	chromosome 17 open reading frame 62
0,99	ZNF407	zinc finger protein 407
0,99	MCM6	minichromosome maintenance complex component 6
0,99	COA6	cytochrome c oxidase assembly factor 6
0,99	TP53	tumor protein p53
0,99	PPIB	peptidylprolyl isomerase B (cyclophilin B)
0,99	KLF10	Kruppel-like factor 10
0,99	CLDN6	claudin 6
0,99	STK24	serine/threonine kinase 24
0,99	LSM11	LSM11, U7 small nuclear RNA associated
0,99	ABCA5	ATP binding cassette subfamily A member 5
0,99	OR5P3	olfactory receptor, family 5, subfamily P, member 3
0,99	RARG	retinoic acid receptor, gamma
0,99	RPP30	ribonuclease P/MRP 30kDa subunit
0,99	RNF114	ring finger protein 114
0,99	ZNF701; ZNF137P	zinc finger protein 701; zinc finger protein 137, pseudogene
0,99	RAB5B	RAB5B, member RAS oncogene family
0,99	GATAD2A; MIR640	GATA zinc finger domain containing 2A; microRNA 640
0,99	ATP5I	ATP synthase, H+ transporting, mitochondrial Fo complex subunit E
0,99	LELP1	late cornified envelope-like proline-rich 1
0,99	GNG12	guanine nucleotide binding protein (G protein), gamma 12
0,99	NSMCE4A	NSE4 homolog A, SMC5-SMC6 complex component
0,99	RASSF2	Memczak2013 ANTISENSE, coding, INTERNAL, UTR3 best transcript NM_014737
0,99	HES4	hes family bHLH transcription factor 4
0,99	CDK1	cyclin-dependent kinase 1
0,99	RP11-35G9.5; ATP8B1	Salzman2013 ANTISENSE, coding, INTERNAL, intronic, OVERLAPTX best transcript NM_005603; novel transcript, antisense to ATP8B1
0,99	ATP6V0E1	ATPase, H+ transporting, lysosomal 9kDa, V0 subunit e1
0,99	ZCCHC17	zinc finger, CCHC domain containing 17
0,99	TSPYL1	TSPY-like 1
0,99	LRRC18	leucine rich repeat containing 18

0,99	SMARCC1	SWI/SNF related, matrix associated, actin dependent regulator of chromatin, subfamily c, member 1
0,99	TTC8	Transcript Identified by AceView, Entrez Gene ID(s) 123016
0,99	TEKT4	tektin 4
0,99	MGST1	microsomal glutathione S-transferase 1
0,99	RUNDC1	RUN domain containing 1
0,99	AP1G1	adaptor-related protein complex 1, gamma 1 subunit
0,99	WNT9B	wingless-type MMTV integration site family, member 9B
0,99	PPP5D1	PPP5 tetratricopeptide repeat domain containing 1
0,99	LMBRD1	LMBR1 domain containing 1
0,99	ATP6V1D	ATPase, H+ transporting, lysosomal 34kDa, V1 subunit D
0,99	SGPP2	sphingosine-1-phosphate phosphatase 2
0,99	DERL2	derlin 2
0,99	LIMS1	LIM and senescent cell antigen-like domains 1
0,99	FRMPD1	FERM and PDZ domain containing 1
0,99	OR52N5	olfactory receptor, family 52, subfamily N, member 5
0,99	PLA2G12B	phospholipase A2, group XIIB
0,99	GPR146	G protein-coupled receptor 146
0,99	ZG16B	zymogen granule protein 16B
0,99	OR7A5	olfactory receptor, family 7, subfamily A, member 5
0,99	C5orf51	chromosome 5 open reading frame 51
0,99	GLB1L2	galactosidase beta 1 like 2
0,99	KRT71	keratin 71, type II
0,99	SAP30BP	SAP30 binding protein
0,99	CLDN5	claudin 5
0,99	TMEM14EP	transmembrane protein 14E, pseudogene
0,99	MET	MET proto-oncogene, receptor tyrosine kinase
0,99	PPM1D	protein phosphatase, Mg2+/Mn2+ dependent, 1D
0,99	EFEMP2	EGF containing fibulin-like extracellular matrix protein 2
0,99	NRL	neural retina leucine zipper
0,99	XPOT	exportin, tRNA
0,99	SRSF3	serine/arginine-rich splicing factor 3
0,99	DPH5	diphthamide biosynthesis 5
0,99	GDNF	glial cell derived neurotrophic factor
0,99	MTA2	metastasis associated 1 family member 2
0,99	CDR1; CDR1-AS	Memczak2013 ANTISENSE, CDS, coding, downstream_end, upstream_start, UTR3, UTR5 best transcript NM_004065; CDR1 antisense RNA; CDR1 antisense RNA [Source:HGNC Symbol;Acc:HGNC:48926]
0,99	GPS2	G protein pathway suppressor 2
0,99	POLI	polymerase (DNA directed) iota
0,99	APBA3	amyloid beta (A4) precursor protein-binding, family A, member 3
0,99	MRFAP1L1	Morf4 family associated protein 1-like 1
0,99	ANGPTL2	angiopoietin like 2

0,99	PLA2G1B	phospholipase A2, group IB (pancreas)
0,99	SH3TC1	SH3 domain and tetratricopeptide repeats 1
0,99	NPR2	natriuretic peptide receptor 2
0,99	FKBP8	FK506 binding protein 8
0,99	PRAMEF25	PRAME family member 25
0,99	MIP	major intrinsic protein of lens fiber
0,99	BTN2A3P	butyrophilin, subfamily 2, member A3, pseudogene
0,99	BCL7B	B-cell CLL/lymphoma 7B
0,99	PMM2	phosphomannomutase 2
0,99	UQCR10	ubiquinol-cytochrome c reductase, complex III subunit X
0,99	EIF4ENIF1	eukaryotic translation initiation factor 4E nuclear import factor 1
0,99	TSEN34	TSEN34 tRNA splicing endonuclease subunit
0,99	TMEM213	transmembrane protein 213
0,99	TJAP1	Zhang2013 ALT_ACCEPTOR, ALT_DONOR, coding, INTERNAL, intronic best transcript NM_001146018
0,99	SLCO1A2	Transcript Identified by AceView, Entrez Gene ID(s) 6579
0,99	PNKD; MIR6810	paroxysmal nonkinesigenic dyskinesia; microRNA 6810
0,99	OSTM1	osteopetrosis associated transmembrane protein 1
0,99	VPS33B	vacuolar protein sorting 33 homolog B (yeast)
0,99	CXorf49B	chromosome X open reading frame 49B
0,99	ZNF600	zinc finger protein 600
0,99	MINA	MYC induced nuclear antigen
0,99	ZNF546	zinc finger protein 546
0,99	C9orf85	chromosome 9 open reading frame 85
0,99	FLYWCH1	FLYWCH-type zinc finger 1
0,99	TAF15	TATA box binding protein associated factor 15
0,99	MIA3	melanoma inhibitory activity family, member 3
0,99	MAVS	mitochondrial antiviral signaling protein
0,99	LARP1	La ribonucleoprotein domain family, member 1
0,99	PYDC2	pyrin domain containing 2
0,99	CTHRC1	collagen triple helix repeat containing 1
0,99	STC1	stanniocalcin 1
0,99	SMC4	structural maintenance of chromosomes 4
0,99	SLC16A8	solute carrier family 16 (monocarboxylate transporter), member 8
0,99	CKS2	CDC28 protein kinase regulatory subunit 2
0,99	SOX30	SRY box 30
0,99	UBE2E2	ubiquitin conjugating enzyme E2E 2
0,99	FLII	flightless I actin binding protein
0,99	UBE2M	ubiquitin-conjugating enzyme E2M
0,99	KIAA2022	KIAA2022
0,99	EPS8L3	EPS8-like 3
0,99	ELL	elongation factor RNA polymerase II
0,99	FAM150A	family with sequence similarity 150, member A
0,99	RPGR	retinitis pigmentosa GTPase regulator

0,99	PERM1	PPARGC1 and ESRR induced regulator, muscle 1
0,99	NPHP3	nephronophthisis 3 (adolescent)
0,99	THOP1	thimet oligopeptidase 1
0,99	MRPL36	mitochondrial ribosomal protein L36
0,99	EXD2	exonuclease 3-5 domain containing 2
0,99	TAS1R2	taste receptor, type 1, member 2
0,99	PLPP4	phospholipid phosphatase 4
0,99	KIAA0101; CSNK1G1	KIAA0101; casein kinase 1, gamma 1
0,99	SPIC	Spi-C transcription factor (Spi-1/PU.1 related)
0,99	RARA	retinoic acid receptor, alpha
0,99	DCUN1D4	DCN1, defective in cullin neddylation 1, domain containing 4
0,99	NUTF2	nuclear transport factor 2
0,99	WDR93	WD repeat domain 93
0,99	THSD4	Transcript Identified by AceView, Entrez Gene ID(s) 79875
0,99	ROR2	receptor tyrosine kinase-like orphan receptor 2
0,99	USP48	ubiquitin specific peptidase 48
0,99	C19orf43	chromosome 19 open reading frame 43
0,99	BATF3	basic leucine zipper transcription factor, ATF-like 3
0,99	HIBADH	3-hydroxyisobutyrate dehydrogenase
0,99	IGSF9B	immunoglobulin superfamily, member 9B
0,99	HNRNPAB	heterogeneous nuclear ribonucleoprotein A/B
0,99	TXNDC11	thioredoxin domain containing 11
0,99	LRRC26	leucine rich repeat containing 26
0,99	TPM4	tropomyosin 4
0,99	FAM27L	family with sequence similarity 27-like
0,99	RESP18	regulated endocrine-specific protein 18
0,99	PCMT1	protein-L-isoaspartate (D-aspartate) O-methyltransferase
0,99	NME7	NME/NM23 family member 7
0,99	CREB3L2	cAMP responsive element binding protein 3-like 2
0,99	sep-09	septin 9
0,99	RAD51L3-RFFL	RAD51L3-RFFL readthrough; HCG2039718, isoform CRA_g; Uncharacterized protein [Source:UniProtKB/TrEMBL;Acc:K7EN88]
0,99	EID1	EP300 interacting inhibitor of differentiation 1
0,99	HINT1	histidine triad nucleotide binding protein 1
0,99	TMEM132A	transmembrane protein 132A
0,99	PRAMEF8	PRAME family member 8
0,99	ERP44	endoplasmic reticulum protein 44
0,99	LMOD3	leiomodin 3 (fetal)
0,99	CSDC2	cold shock domain containing C2, RNA binding
0,99	MAP4K2	mitogen-activated protein kinase kinase kinase kinase 2
0,99	SURF2	surfeit 2 [Source:HGNC Symbol;Acc:HGNC:11475]
0,99	HADHA	hydroxyacyl-CoA dehydrogenase/3-ketoacyl-CoA thiolase/enoyl-CoA hydratase (trifunctional protein), alpha subunit

0,99	PRKG1	protein kinase, cGMP-dependent, type I
0,99	RRAGC	Ras-related GTP binding C
0,99	FAM172A; POU5F2; MIR2277	family with sequence similarity 172, member A; POU domain class 5, transcription factor 2; microRNA 2277
0,99	BHLHE23	basic helix-loop-helix family, member e23
0,99	ENPEP	glutamyl aminopeptidase (aminopeptidase A)
0,99	CCDC151	coiled-coil domain containing 151
0,99	C18orf42	chromosome 18 open reading frame 42
0,99	ZNF767P	zinc finger family member 767, pseudogene
0,99	EEF1G; MIR3654	eukaryotic translation elongation factor 1 gamma; microRNA 3654
0,99	IQCC	IQ motif containing C
0,99	HSD17B11	hydroxysteroid (17-beta) dehydrogenase 11
0,99	PRSS57	protease, serine, 57
0,99	SKP1	S-phase kinase-associated protein 1
0,99	GABARAP	GABA(A) receptor-associated protein
0,99	RFC1	replication factor C subunit 1
0,99	BYSL	bystin-like
0,99	DNAJC25-GNG10	DNAJC25-GNG10 readthrough
0,99	SLC15A2	solute carrier family 15 (oligopeptide transporter), member 2
0,99	MRPS36	mitochondrial ribosomal protein S36
0,99	MARCH2	membrane associated ring finger 2
0,99	CANX	calnexin
0,99	CHP2	calcineurin-like EF-hand protein 2
0,99	ST3GAL5	ST3 beta-galactoside alpha-2,3-sialyltransferase 5
0,99	RAD51AP1	RAD51 associated protein 1
0,99	CEP72	centrosomal protein 72kDa
0,99	CC2D1A	coiled-coil and C2 domain containing 1A
0,99	NDUFV1	NADH dehydrogenase (ubiquinone) flavoprotein 1, 51kDa
0,99	NFATC3	nuclear factor of activated T-cells, cytoplasmic, calcineurin-dependent 3
0,99	LHFPL2	lipoma HMGIC fusion partner-like 2
0,99	OTUB1	OTU deubiquitinase, ubiquitin aldehyde binding 1
0,99	CHTF18	chromosome transmission fidelity factor 18
0,99	ZYG11A	zyg-11 family member A, cell cycle regulator
0,99	NEIL3	nei-like DNA glycosylase 3
0,99	ZNF112	zinc finger protein 112
0,99	TAX1BP1	Tax1 (human T-cell leukemia virus type I) binding protein 1
0,99	ATP6AP2	ATPase, H+ transporting, lysosomal accessory protein 2
0,99	GALNT14	polypeptide N-acetylgalactosaminyltransferase 14
0,99	NDUFB9	NADH dehydrogenase (ubiquinone) 1 beta subcomplex, 9, 22kDa
0,99	GEMIN4	gem nuclear organelle associated protein 4
0,99	TBC1D4	TBC1 domain family, member 4

0,99	BORCS7	BLOC-1 related complex subunit 7
0,99	PCDHB14	protocadherin beta 14
0,99	TBCB	tubulin folding cofactor B
0,99	HSPD1	heat shock 60kDa protein 1 (chaperonin)
0,99	TPP2	tripeptidyl peptidase II
0,99	RP1L1	retinitis pigmentosa 1-like 1
0,99	RPP40	ribonuclease P/MRP 40kDa subunit
0,99	PPP2R3A	protein phosphatase 2, regulatory subunit B, alpha
0,99	SRGN	Transcript Identified by AceView, Entrez Gene ID(s) 5552
0,99	COPS3	COP9 signalosome subunit 3
0,99	CYP2A6	cytochrome P450, family 2, subfamily A, polypeptide 6
0,99	OR5I1	olfactory receptor, family 5, subfamily I, member 1
0,99	ROR1-AS1	ROR1 antisense RNA 1
0,99	LSM14B	LSM family member 14B
0,99	MBNL1	muscleblind like splicing regulator 1
0,99	PHF11	PHD finger protein 11
0,99	TAP2	transporter 2, ATP-binding cassette, sub-family B (MDR/TAP)
0,99	ST20-MTHFS	ST20-MTHFS readthrough
0,99	TPMT	thiopurine S-methyltransferase
0,99	RBM4	RNA binding motif protein 4
0,99	KAT5	K(lysine) acetyltransferase 5
0,99	RNF170; MIR4469	ring finger protein 170; microRNA 4469
0,99	CBL	Cbl proto-oncogene, E3 ubiquitin protein ligase
0,99	TRMT112	tRNA methyltransferase 11-2 homolog (S. cerevisiae)
0,99	ID4	inhibitor of DNA binding 4, dominant negative helix-loop-helix protein
0,99	MAFK	v-maf avian musculoaponeurotic fibrosarcoma oncogene homolog K
0,99	RCN2	reticulocalbin 2, EF-hand calcium binding domain
0,99	LRIT2	leucine-rich repeat, immunoglobulin-like and transmembrane domains 2
0,99	ABCD1	Transcript Identified by AceView, Entrez Gene ID(s) 215
0,99	SNX6	sorting nexin 6
0,99	POLE3	polymerase (DNA directed), epsilon 3, accessory subunit
0,99	CDH2	cadherin 2, type 1, N-cadherin (neuronal)
0,99	TLR8	toll-like receptor 8
0,99	LRRC56	leucine rich repeat containing 56
0,99	CYP4F8	cytochrome P450, family 4, subfamily F, polypeptide 8
0,99	PLCXD1	phosphatidylinositol-specific phospholipase C, X domain containing 1
0,99	TMEM62	transmembrane protein 62
0,99	TRUB1	TruB pseudouridine (psi) synthase family member 1
0,99	ZNF593	zinc finger protein 593

0,99	SRSF11	serine/arginine-rich splicing factor 11
0,99	C17orf89	chromosome 17 open reading frame 89
0,99	HNRNPA2B1	heterogeneous nuclear ribonucleoprotein A2/B1
0,99	COPZ1	coatomer protein complex subunit zeta 1
0,99	DYNLT3	dynein, light chain, Tctex-type 3
0,99	IER3IP1	immediate early response 3 interacting protein 1
0,99	AGMAT	agmatinase
0,99	TECR; MIR639	trans-2,3-enoyl-CoA reductase; microRNA 639
0,99	PSMA4	proteasome subunit alpha 4
0,99	RPS20; SNORD54	ribosomal protein S20; small nucleolar RNA, C/D box 54
0,99	EID2B	EP300 interacting inhibitor of differentiation 2B
0,99	ZFAND6	zinc finger, AN1-type domain 6
0,99	CRYGA	crystallin gamma A
0,99	LRRC8A	leucine rich repeat containing 8 family, member A
0,99	IFI27L2	interferon, alpha-inducible protein 27-like 2
0,99	CPNE2	copine II
0,99	CASP12	caspase 12 (gene/pseudogene)
0,99	SPATA19	spermatogenesis associated 19
0,99	PLEKHF2	pleckstrin homology domain containing, family F (with FYVE domain) member 2
0,99	SPTB; MIR7855	spectrin, beta, erythrocytic; microRNA 7855
0,99	KIDINS220	kinase D-interacting substrate 220kDa
0,99	MTERF3	mitochondrial transcription termination factor 3
0,99	LDLRAD3	low density lipoprotein receptor class A domain containing 3
0,99	THAP9-AS1	THAP9 antisense RNA 1
0,99	TP53I11	tumor protein p53 inducible protein 11
0,99	ZNF501	zinc finger protein 501 [Source:HGNC Symbol;Acc:HGNC:23717]
0,99	NUMBL	numb homolog (Drosophila)-like
0,99	ISY1	ISY1 splicing factor homolog
0,99	MRPS18B	mitochondrial ribosomal protein S18B
0,99	ZNF880	zinc finger protein 880
0,99	CD40LG	CD40 ligand
0,99	CDK2AP1	cyclin-dependent kinase 2 associated protein 1
0,99	SLC22A2	solute carrier family 22 (organic cation transporter), member 2
0,99	RTN3	reticulon 3
0,99	SOCS6	suppressor of cytokine signaling 6
0,99	PADI4	peptidyl arginine deiminase, type IV
0,99	SLC25A51	solute carrier family 25, member 51
0,99	ABCA8	ATP binding cassette subfamily A member 8
0,99	GSTM5	glutathione S-transferase mu 5
0,99	SFPQ	splicing factor proline/glutamine-rich
0,99	DISP2	dispatched homolog 2 (Drosophila)
0,99	SLITRK2	SLIT and NTRK-like family, member 2
0,99	UBE2Q1	ubiquitin-conjugating enzyme E2Q family member 1
0,99	NADK2	NAD kinase 2, mitochondrial
0,99	ZNF529-AS1	ZNF529 antisense RNA 1

0,99	ASB1	ankyrin repeat and SOCS box containing 1
0,99	BSCL2	Berardinelli-Seip congenital lipodystrophy 2 (seipin)
0,99	PQLC3	PQ loop repeat containing 3
0,99	OTOP2	otopetrin 2
0,99	TAF15	Transcript Identified by AceView, Entrez Gene ID(s) 8148
0,99	SFT2D1	SFT2 domain containing 1
0,99	ANKRD20A5P; RHOT1P1	ankyrin repeat domain 20 family, member A5, pseudogene; ras homolog family member T1 pseudogene 1
0,99	SEMA4C	sema domain, immunoglobulin domain (Ig), transmembrane domain (TM) and short cytoplasmic domain, (semaphorin) 4C
0,99	HP1BP3	heterochromatin protein 1, binding protein 3
0,99	DENND4B	DENN/MADD domain containing 4B
0,99	CTRL	chymotrypsin-like
0,99	TRH	thyrotropin-releasing hormone
0,99	PHYHIPL	phytanoyl-CoA 2-hydroxylase interacting protein-like
0,99	SERBP1	SERPINE1 mRNA binding protein 1
0,99	RIPK3	receptor-interacting serine-threonine kinase 3
0,99	C21orf33	chromosome 21 open reading frame 33
0,99	MRAP	melanocortin 2 receptor accessory protein
0,99	SKOR1	SKI family transcriptional corepressor 1
0,99	CTSD	cathepsin D
0,99	GTPBP1	GTP binding protein 1
0,99	SQRDL	sulfide quinone reductase-like (yeast)
0,99	FAM155A	Jeck2013 ALT_ACCEPTOR, ALT_DONOR, coding, INTERNAL, intronic best transcript NM_001080396
0,99	OTOGL	otogelin-like
0,99	PRR5-ARHGAP8	PRR5-ARHGAP8 readthrough
0,99	CLDN7	claudin 7
0,99	ULK1	unc-51 like autophagy activating kinase 1
0,99	PPT1	palmitoyl-protein thioesterase 1
0,99	HIST1H4K	histone cluster 1, H4k
0,99	HACD2	3-hydroxyacyl-CoA dehydratase 2
0,99	TNIP1	TNFAIP3 interacting protein 1
0,99	ACTB	actin, beta
0,99	MRPS28	mitochondrial ribosomal protein S28
0,99	KAT6B	K(lysine) acetyltransferase 6B
0,99	TLCD1	TLC domain containing 1
0,99	RACGAP1	Rac GTPase activating protein 1
0,99	HEXIM2	hexamethylene bis-acetamide inducible 2
0,99	TOMM7	translocase of outer mitochondrial membrane 7 homolog (yeast)
0,99	TCEAL6	transcription elongation factor A (SII)-like 6
0,99	CASP16P	caspase 16, pseudogene
0,99	COL18A1	collagen, type XVIII, alpha 1
0,99	YWHAH	tyrosine 3-monooxygenase/tryptophan 5-monooxygenase activation protein, eta
0,99	EMC4	ER membrane protein complex subunit 4

0,99	TOMM20	translocase of outer mitochondrial membrane 20 homolog (yeast)
0,99	PARP1	poly(ADP-ribose) polymerase 1
0,99	TCF3	transcription factor 3
0,99	CDC5L	cell division cycle 5-like
0,99	HRNR	hornerin
0,99	AFTPH	aftiphilin
0,99	ZNF197	zinc finger protein 197
0,99	SLC39A14	solute carrier family 39 (zinc transporter), member 14
0,99	TAGLN2	transgelin 2
0,99	SOX13	SRY box 13
0,99	SPCS2	signal peptidase complex subunit 2
0,99	SHISA6	shisa family member 6
0,99	NOS3	Transcript Identified by AceView, Entrez Gene ID(s) 4846
0,99	ATP7B	ATPase, Cu++ transporting, beta polypeptide
0,99	FBXL4	F-box and leucine-rich repeat protein 4
0,99	SNRNP25	small nuclear ribonucleoprotein, U11/U12 25kDa subunit
0,99	PRMT2	protein arginine methyltransferase 2
0,99	PRMT5	protein arginine methyltransferase 5
0,99	LSG1	large 60S subunit nuclear export GTPase 1
0,99	MKLN1	muskelin 1, intracellular mediator containing kelch motifs
0,99	ERF	Ets2 repressor factor
0,99	MRPL13	mitochondrial ribosomal protein L13
0,99	CD5L	CD5 molecule-like
0,99	TGM7	transglutaminase 7
0,99	CALCOCO1	calcium binding and coiled-coil domain 1
0,99	KRT14	keratin 14, type I
0,99	TMPRSS11D	transmembrane protease, serine 11D
0,99	ZNF229	zinc finger protein 229
0,99	HHAT	hedgehog acyltransferase
0,99	RNASE6	ribonuclease, RNase A family, k6
0,99	AP2M1	adaptor-related protein complex 2, mu 1 subunit
0,99	NPAT	nuclear protein, ataxia-telangiectasia locus
0,99	TMX1	thioredoxin-related transmembrane protein 1
0,99	TMEM231	transmembrane protein 231
0,99	PRAP1	proline-rich acidic protein 1
0,99	WBP4	Transcript Identified by AceView, Entrez Gene ID(s) 11193
0,99	NDUFAF6	NADH dehydrogenase (ubiquinone) complex I, assembly factor 6
0,99	BLOC1S3	biogenesis of lysosomal organelles complex-1, subunit 3
0,99	EPB41L4B	erythrocyte membrane protein band 4.1 like 4B
0,99	DIAPH3	diaphanous-related formin 3
0,99	IFNA5	interferon, alpha 5
0,99	PCDHB10	protocadherin beta 10
0,99	TRIM65	tripartite motif containing 65

0,99	CDK17	cyclin-dependent kinase 17
0,99	CER1	cerberus 1, DAN family BMP antagonist
0,99	C11orf40	chromosome 11 open reading frame 40
0,99	EEF1A1	eukaryotic translation elongation factor 1 alpha 1
0,99	MIR205HG; MIR205	MIR205 host gene; microRNA 205
0,99	MYL12B	myosin light chain 12B
0,99	DIS3	DIS3 homolog, exosome endoribonuclease and 3-5 exoribonuclease
0,99	ZNF131	zinc finger protein 131
0,99	FAM69B	family with sequence similarity 69, member B
0,99	YOD1	YOD1 deubiquitinase
0,99	MBP	myelin basic protein
0,99	KRT3	keratin 3, type II
0,99	SFXN5	sideroflexin 5
0,99	ZNF160	zinc finger protein 160
0,99	ATAD3B	ATPase family, AAA domain containing 3B
0,99	RPL36A	ribosomal protein L36a
0,99	FAM46D	family with sequence similarity 46, member D
0,99	SIX6	SIX homeobox 6
0,99	ZNF287	zinc finger protein 287
0,99	LRP8	LDL receptor related protein 8
0,99	PHB2; SCARNA12	prohibitin 2; small Cajal body-specific RNA 12
0,99	TMEM59	transmembrane protein 59
0,99	NCAPH2	non-SMC condensin II complex subunit H2
0,99	RPS13; SNORD14B	ribosomal protein S13; small nucleolar RNA, C/D box 14B
0,99	DSG2	desmoglein 2
0,99	KHDRBS1	KH domain containing, RNA binding, signal transduction associated 1
0,99	PLXNA1	plexin A1
0,99	ZNF587	zinc finger protein 587
0,99	RANBP1	RAN binding protein 1
0,99	CCDC152	coiled-coil domain containing 152
0,99	GORASP1	golgi reassembly stacking protein 1
0,99	CYB561A3	cytochrome b561 family, member A3
0,99	ACTR10	actin-related protein 10 homolog (S. cerevisiae)
0,99	CLEC2D	C-type lectin domain family 2, member D
0,99	MLX	MLX, MAX dimerization protein
0,99	METTL2A	methyltransferase like 2A
0,99	C1orf158	chromosome 1 open reading frame 158
0,99	FBXO41	F-box protein 41
0,99	VRK3	vaccinia related kinase 3
0,99	PCP4L1	Purkinje cell protein 4 like 1
0,99	PAPOLG	Transcript Identified by AceView, Entrez Gene ID(s) 64895
0,99	NFATC2IP; MIR4517	nuclear factor of activated T-cells, cytoplasmic, calcineurin-dependent 2 interacting protein; microRNA 4517

0,99	EPHX4	epoxide hydrolase 4
0,99	STARD6	StAR-related lipid transfer domain containing 6
0,99	IRG1	immunoresponsive 1 homolog (mouse)
0,99	TUBA3C	tubulin, alpha 3c
0,99	CYGB	cytoglobin
0,99	TFEC	transcription factor EC
0,99	ZNF780B	zinc finger protein 780B
0,99	RNF212	ring finger protein 212
0,99	FAM32A	family with sequence similarity 32, member A
0,99	KDM2B	lysine (K)-specific demethylase 2B
0,99	PURG	purine-rich element binding protein G
0,99	NUP62CL	nucleoporin 62kDa C-terminal like
0,99	PRR19	proline rich 19
0,99	HYOU1	hypoxia up-regulated 1
0,99	SARAF	store-operated calcium entry-associated regulatory factor
0,99	SLC24A4	solute carrier family 24 (sodium/potassium/calcium exchanger), member 4
0,99	KCNC1	potassium channel, voltage gated Shaw related subfamily C, member 1
0,99	CCDC110	coiled-coil domain containing 110
0,99	LRRC9	leucine rich repeat containing 9
0,99	IVNS1ABP	influenza virus NS1A binding protein
0,99	PTCRA	pre T-cell antigen receptor alpha
0,99	NEUROG3	neurogenin 3
0,99	PPM1A	protein phosphatase, Mg2+/Mn2+ dependent, 1A
0,99	ANKRD22	ankyrin repeat domain 22
0,99	DNAL1	dynein, axonemal, light chain 1
0,99	SCN8A	Jeck2013 ANTISENSE, coding, INTERNAL, intronic best transcript NM_014191
0,99	TFAM	transcription factor A, mitochondrial
0,99	LOC100129083; CTD-2616J11.2	uncharacterized LOC100129083; Transcript Identified by AceView, Entrez Gene ID(s) 100129083; novel transcript, antisense to SIGLEC10
0,99	NUCB1	nucleobindin 1
0,99	TRMT5	tRNA methyltransferase 5
0,99	IRX4	iroquois homeobox 4
0,99	OSMR	oncostatin M receptor
0,99	TTC17	tetratricopeptide repeat domain 17
0,99	TNFAIP8L3	tumor necrosis factor, alpha-induced protein 8-like 3
0,99	CSRP1	cysteine and glycine-rich protein 1
0,99	KCNA7	potassium channel, voltage gated shaker related subfamily A, member 7
0,99	RELT	RELT tumor necrosis factor receptor
0,99	SRP68	signal recognition particle 68kDa
0,99	TP63	tumor protein p63
0,99	STRAP	serine/threonine kinase receptor associated protein
0,99	FBL	fibrillarin
0,99	RNF224	ring finger protein 224
0,99	PDPN	podoplanin

0,99	FOXN2	forkhead box N2
0,99	MTRNR2L6	MT-RNR2-like 6
0,99	C15orf38-AP3S2	C15orf38-AP3S2 readthrough
0,99	FSTL3	follistatin-like 3 (secreted glycoprotein)
0,99	CAPN1	calpain 1, (mu/I) large subunit
0,99	NDUFS3	NADH dehydrogenase (ubiquinone) Fe-S protein 3, 30kDa (NADH-coenzyme Q reductase)
0,99	MOGAT1	monoacylglycerol O-acyltransferase 1
0,99	FUZ	fuzzy planar cell polarity protein
0,99	ADORA1	adenosine A1 receptor
0,99	CGGBP1	CGG triplet repeat binding protein 1
0,99	RHOBTB2	Rho-related BTB domain containing 2
0,99	SLC10A1	solute carrier family 10 (sodium/bile acid cotransporter), member 1
0,99	NUP35	nucleoporin 35kDa
0,99	CPNE7	copine VII
0,99	RUNX1	runt-related transcription factor 1
0,99	SEC23B	Sec23 homolog B, COPII coat complex component
0,99	SERPINA11	serpin peptidase inhibitor, clade A (alpha-1 antiproteinase, antitrypsin), member 11
0,99	ABCA9	ATP binding cassette subfamily A member 9
0,99	TFPI	tissue factor pathway inhibitor (lipoprotein-associated coagulation inhibitor)
0,99	POU4F1	POU class 4 homeobox 1
0,99	BDNF	brain-derived neurotrophic factor
0,99	MICAL2	microtubule associated monooxygenase, calponin and LIM domain containing 2
0,99	GLCE	glucuronic acid epimerase
0,99	EDNRB	endothelin receptor type B
0,99	SYNJ1	synaptojanin 1
0,99	ADI1	acireductone dioxygenase 1
0,99	NECAP2	NECAP endocytosis associated 2
0,99	MIIP	Transcript Identified by AceView, Entrez Gene ID(s) 60672
0,99	CRAT	carnitine O-acetyltransferase
0,99	FAM156B; FAM156A	family with sequence similarity 156, member B; family with sequence similarity 156, member A
0,99	CRYBA4	crystallin beta A4
0,99	FOXO4	forkhead box O4
0,99	SMIM7; MED26	small integral membrane protein 7; mediator complex subunit 26
0,99	KDM2A	lysine (K)-specific demethylase 2A
0,99	RPP21	ribonuclease P/MRP 21kDa subunit
0,99	ADPRM	ADP-ribose/CDP-alcohol diphosphatase, manganese-dependent
0,99	VCPKMT	valosin containing protein lysine (K) methyltransferase
0,99	TSPAN1	tetraspanin 1
0,99	ARPC3	actin related protein 2/3 complex subunit 3
0,99	RRAS	related RAS viral (r-ras) oncogene homolog

0,99	SCO1	SCO1 cytochrome c oxidase assembly protein
0,99	BPIFA2	BPI fold containing family A, member 2
0,99	PDGFA	platelet-derived growth factor alpha polypeptide
0,99	RAB10	RAB10, member RAS oncogene family
0,99	ZNF645	zinc finger protein 645
0,99	FRMD8	FERM domain containing 8
0,99	FBL	fibrillarin
0,99	RHEB	Ras homolog enriched in brain
0,99	SECISBP2L	SECIS binding protein 2-like
0,99	sep-07	septin 7
0,99	PHF20L1	PHD finger protein 20-like 1
0,99	CCDC93	coiled-coil domain containing 93
0,99	SMIM13	small integral membrane protein 13
0,99	ERCC6L2	excision repair cross-complementation group 6-like 2
0,99	DAPK3; MIR637	death-associated protein kinase 3; microRNA 637
0,99	PPP6R2	protein phosphatase 6, regulatory subunit 2
0,99	TCEB2	transcription elongation factor B (SIII), polypeptide 2 (18kDa, elongin B)
0,99	CALR	calreticulin
0,99	CDS1	CDP-diacylglycerol synthase 1
0,99	KIRREL	kin of IRRE like (Drosophila)
0,99	USP12	ubiquitin specific peptidase 12
0,99	C21orf59	chromosome 21 open reading frame 59
0,99	CELA3A	chymotrypsin-like elastase family, member 3A
0,99	MAG	myelin associated glycoprotein
0,99	MRPS14	mitochondrial ribosomal protein S14
0,99	PMS2P3	PMS1 homolog 2, mismatch repair system component pseudogene 3
0,99	RPL38	ribosomal protein L38
0,99	CT47A1	cancer/testis antigen family 47, member A1
0,99	ZNF555	zinc finger protein 555
0,99	RAP2A	RAP2A, member of RAS oncogene family
0,99	ERICH5	glutamate rich 5
0,99	TNFSF13	tumor necrosis factor (ligand) superfamily, member 13
0,99	HOXB9	homeobox B9
0,99	CNOT3	CCR4-NOT transcription complex subunit 3
0,99	DDX3Y	DEAD (Asp-Glu-Ala-Asp) box helicase 3, Y-linked
0,99	PATE1	prostate and testis expressed 1
0,99	RAD23B	RAD23 homolog B, nucleotide excision repair protein
0,99	ZBED6CL	ZBED6 C-terminal like
0,99	HIF1A	hypoxia inducible factor 1, alpha subunit (basic helix-loop-helix transcription factor)
0,99	RNF207	ring finger protein 207
0,99	NFKB1	nuclear factor of kappa light polypeptide gene enhancer in B-cells 1
0,99	ASZ1	ankyrin repeat, SAM and basic leucine zipper domain containing 1
0,99	ARNTL	aryl hydrocarbon receptor nuclear translocator-like
0,99	DHX36	DEAH (Asp-Glu-Ala-His) box polypeptide 36

0,99	SLC35D2	solute carrier family 35 (UDP-GlcNAc/UDP-glucose transporter), member D2
0,99	SHKBP1	SH3KBP1 binding protein 1
0,99	PRSS35	protease, serine 35
0,99	PMP22	peripheral myelin protein 22
0,99	OLFM4	olfactomedin 4
0,99	PSMB5	proteasome subunit beta 5
0,99	TBC1D24	TBC1 domain family, member 24
0,99	CCNT2-AS1	CCNT2 antisense RNA 1
0,99	OR3A4P; OR3A5P	olfactory receptor, family 3, subfamily A, member 4 pseudogene; olfactory receptor, family 3, subfamily A, member 5 pseudogene
0,99	KCNB1	potassium channel, voltage gated Shab related subfamily B, member 1
0,99	WSCD1	WSC domain containing 1
0,99	ATP1A4	ATPase, Na+/K+ transporting, alpha 4 polypeptide
0,99	RBM6	RNA binding motif protein 6
0,99	ZNF595	zinc finger protein 595
0,99	VAMP7	vesicle associated membrane protein 7
0,99	C1QC	complement component 1, q subcomponent, C chain
0,99	RDH10-AS1	RDH10 antisense RNA 1
0,99	FSCN2	fascin actin-bundling protein 2, retinal
0,99	TEK	TEK tyrosine kinase, endothelial
0,99	IFT27	intraflagellar transport 27
0,99	ZBTB11	zinc finger and BTB domain containing 11
0,99	IFITM10	interferon induced transmembrane protein 10
0,99	MRPL3	mitochondrial ribosomal protein L3
0,99	SYNGR3	synaptogyrin 3
0,99	GUCA1C	guanylate cyclase activator 1C
0,99	FMR1NB	fragile X mental retardation 1 neighbor
0,99	MPP1	membrane protein, palmitoylated 1
0,99	SGPL1	sphingosine-1-phosphate lyase 1
0,99	ARHGAP15	Rho GTPase activating protein 15
0,99	GIT1	G protein-coupled receptor kinase interacting ArfGAP 1
0,99	IP6K2	inositol hexakisphosphate kinase 2
0,99	OBP2B	odorant binding protein 2B
0,99	FAM89B	family with sequence similarity 89, member B
0,99	DERA	deoxyribose-phosphate aldolase (putative)
0,99	PROX2	prospero homeobox 2
0,99	CCNA1	cyclin A1
0,99	PCGF6	polycomb group ring finger 6
0,99	LIPI	lipase, member I
0,99	PLEKHN1	pleckstrin homology domain containing, family N member 1
0,99	HES1	hes family bHLH transcription factor 1
0,99	GPN1	GPN-loop GTPase 1
0,99	RNF220	ring finger protein 220
0,99	DHRS13	dehydrogenase/reductase (SDR family) member 13
0,99	MYADML2	myeloid-associated differentiation marker-like 2

0,99	TCEANC	transcription elongation factor A (SII) N-terminal and central domain containing
0,99	TMBIM6	transmembrane BAX inhibitor motif containing 6
0,99	SLC25A20	solute carrier family 25 (carnitine/acylcarnitine translocase), member 20
0,99	SPATA32	spermatogenesis associated 32
0,99	GMPR2	guanosine monophosphate reductase 2
0,99	KRIT1	KRIT1, ankyrin repeat containing
0,99	CASP8AP2	caspase 8 associated protein 2
0,99	SNX19	sorting nexin 19
0,99	NNT	nicotinamide nucleotide transhydrogenase
0,99	PRPF4B	pre-mRNA processing factor 4B
0,99	KIAA0100	KIAA0100
0,99	C11orf73	chromosome 11 open reading frame 73
0,99	OSBP2	oxysterol binding protein 2
0,99	SULT1A1	sulfotransferase family 1A member 1
0,99	DCTD	dCMP deaminase
0,99	SERPINB5	serpin peptidase inhibitor, clade B (ovalbumin), member 5
0,99	COX5A	cytochrome c oxidase subunit Va
0,99	MAB21L2	mab-21-like 2 (C. elegans)
0,99	ZC3H14	zinc finger CCCH-type containing 14
0,99	VIT	vitrin
0,99	STARD3	StAR-related lipid transfer domain containing 3
0,99	CCDC167	coiled-coil domain containing 167
0,99	BLOC1S5-TXNDC5	BLOC1S5-TXNDC5 readthrough (NMD candidate)
0,99	CCNF	cyclin F
0,99	GPR12	G protein-coupled receptor 12
0,99	ADGRV1	adhesion G protein-coupled receptor V1
0,99	C1orf198	chromosome 1 open reading frame 198
0,99	MTM1	myotubularin 1
0,99	TDP1	tyrosyl-DNA phosphodiesterase 1
0,99	NBPF7	neuroblastoma breakpoint family, member 7
0,99	C3	complement component 3
0,99	AZI2	5-azacytidine induced 2
0,99	CDC25C	cell division cycle 25C
0,99	GPATCH1	G-patch domain containing 1
0,99	ADCY9	adenylate cyclase 9
0,99	ZSWIM3	zinc finger, SWIM-type containing 3
0,99	GOLPH3	golgi phosphoprotein 3 (coat-protein)
0,99	FANCI	Fanconi anemia complementation group I
0,99	PAXBP1	PAX3 and PAX7 binding protein 1
0,99	GPATCH3	G-patch domain containing 3
0,99	CD151	CD151 molecule (Raph blood group)
0,99	SLC39A4	solute carrier family 39 (zinc transporter), member 4
0,99	KRT31	keratin 31, type I
0,99	ASCL5	achaete-scute family bHLH transcription factor 5
0,99	STIM1; MIR4687	stromal interaction molecule 1; microRNA 4687
0,99	HJURP	Holliday junction recognition protein

0,99	NDUFA1	NADH dehydrogenase (ubiquinone) 1 alpha subcomplex, 1, 7.5kDa
0,99	RPL23A; SNORD4B; SNORD42B; SNORD42A	ribosomal protein L23a; small nucleolar RNA, C/D box 4B; small nucleolar RNA, C/D box 42B; small nucleolar RNA, C/D box 42A
0,99	LINC00961	long intergenic non-protein coding RNA 961
0,99	MAU2	MAU2 sister chromatid cohesion factor
0,99	MECOM	MDS1 and EVI1 complex locus
0,99	OR10J1	olfactory receptor, family 10, subfamily J, member 1
0,99	LAMA2	laminin, alpha 2
0,99	CTSV	cathepsin V
0,99	SLC38A11	solute carrier family 38, member 11
0,99	USP17L2	ubiquitin specific peptidase 17-like family member 2
0,99	FAH	fumarylacetoacetate hydrolase (fumarylacetoacetase)
0,99	EBPL	emopamil binding protein-like
0,99	PPA2	pyrophosphatase (inorganic) 2
0,99	PLLP	plasmolipin
0,99	SPAST	spastin
0,99	ARHGAP4	Rho GTPase activating protein 4
0,99	SPINK4	serine peptidase inhibitor, Kazal type 4
0,99	MRPS23	mitochondrial ribosomal protein S23
0,99	FLOT1	flotillin 1
0,99	MYH4	myosin, heavy chain 4, skeletal muscle
0,99	UBA5	ubiquitin-like modifier activating enzyme 5
0,99	UBE2H	ubiquitin conjugating enzyme E2H
0,99	RNF157	ring finger protein 157
0,99	OR10P1	olfactory receptor, family 10, subfamily P, member 1
0,99	CSPG4P1Y	chondroitin sulfate proteoglycan 4 pseudogene 1, Y-linked
0,99	SEC61A2	Sec61 translocon alpha 2 subunit
0,99	SURF2	surfeit 2 [Source:HGNC Symbol;Acc:HGNC:11475]
0,99	IBSP	integrin-binding sialoprotein
0,99	TUBGCP4	tubulin, gamma complex associated protein 4
0,99	PRL	prolactin
0,99	VIPAS39	VPS33B interacting protein, apical-basolateral polarity regulator, spe-39 homolog
0,99	RAB30	RAB30, member RAS oncogene family
0,99	ADK	Memczak2013 ANTISENSE, coding, INTERNAL, intronic best transcript NM_006721
0,99	DDX1	DEAD (Asp-Glu-Ala-Asp) box helicase 1
0,99	SLC25A38	solute carrier family 25, member 38
0,99	ALAS1	5-aminolevulinate synthase 1
0,99	ZIK1	zinc finger protein interacting with K protein 1
0,99	KCTD20	potassium channel tetramerization domain containing 20
0,99	F11-AS1	F11 antisense RNA 1
0,99	MRPL44	mitochondrial ribosomal protein L44
0,99	PPP1CB	protein phosphatase 1, catalytic subunit, beta isozyme
0,99	LINC00663	long intergenic non-protein coding RNA 663

0,99	EIF1AD	eukaryotic translation initiation factor 1A domain containing
0,99	ZNF57	zinc finger protein 57
0,99	PWWP2A	PWWP domain containing 2A
0,99	RECQL5	RecQ helicase-like 5
0,99	RASA1	RAS p21 protein activator (GTPase activating protein) 1
0,99	FAM163B	family with sequence similarity 163, member B
0,99	ATRX	alpha thalassemia/mental retardation syndrome X-linked
0,99	ATP13A3	ATPase type 13A3
0,99	CCDC42	coiled-coil domain containing 42
0,99	ASB14	ankyrin repeat and SOCS box containing 14
0,99	TRAF3IP1	TNF receptor-associated factor 3 interacting protein 1
0,99	SURF6	surfeit 6
0,99	HYOU1	hypoxia up-regulated 1
0,99	PTPRN2	protein tyrosine phosphatase, receptor type, N polypeptide 2
0,99	CA5A	carbonic anhydrase VA, mitochondrial
0,99	ME1	malic enzyme 1, NADP(+)-dependent, cytosolic
0,99	BST2	bone marrow stromal cell antigen 2
0,99	TTC16	tetratricopeptide repeat domain 16
0,99	POLG	polymerase (DNA directed), gamma
0,99	METTL15	methyltransferase like 15
0,99	HILPDA	hypoxia inducible lipid droplet-associated
0,99	TMEM67	transmembrane protein 67
0,99	DDX28	DEAD (Asp-Glu-Ala-Asp) box polypeptide 28
0,99	IMPA2	inositol(myo)-1(or 4)-monophosphatase 2
0,99	REXO1L2P; REXO1L1; blarbeyby	Homo sapiens REX1, RNA exonuclease 1 homolog-like 2, pseudogene (REXO1L2P), non-coding RNA.; Synthetic construct Homo sapiens clone IMAGE:100068172, MGC:195789 REX1, RNA exonuclease 1 homolog (S. cerevisiae)-like 1 (REXO1L1) mRNA, encodes complete protein.; Transcript Identified by AceView, RefSeq ID(s) NR_003594
0,99	STAG3L2	stromal antigen 3-like 2 (pseudogene)
0,99	ADAM28	ADAM metallopeptidase domain 28
0,99	PPP1R1B	protein phosphatase 1, regulatory (inhibitor) subunit 1B
0,99	PAQR9	progestin and adipoQ receptor family member IX
0,99	BCAT1	branched chain amino-acid transaminase 1, cytosolic
0,99	TCEB3	transcription elongation factor B (SIII), polypeptide 3 (110kDa, elongin A)
0,99	ASH2L	ash2 (absent, small, or homeotic)-like (Drosophila)
0,99	GTF2F1	general transcription factor IIF subunit 1
0,99	KCTD2	potassium channel tetramerization domain containing 2
0,99	GPI	glucose-6-phosphate isomerase
0,99	PPP1R3G	protein phosphatase 1, regulatory subunit 3G
0,99	HYAL1	hyaluronoglucosaminidase 1

0,99	KRTAP10-2	keratin associated protein 10-2
0,99	C15orf61	chromosome 15 open reading frame 61
0,99	VPS51	vacuolar protein sorting 51 homolog (S. cerevisiae)
0,99	MYH7B	myosin, heavy chain 7B, cardiac muscle, beta
0,99	MPL	MPL proto-oncogene, thrombopoietin receptor
0,99	ZNF283	zinc finger protein 283
0,99	ZFAND2B	zinc finger, AN1-type domain 2B
0,99	RAB2A	RAB2A, member RAS oncogene family
0,99	ARHGAP26	Rho GTPase activating protein 26
0,99	GNG5	guanine nucleotide binding protein (G protein), gamma 5
0,99	TSHR	thyroid stimulating hormone receptor
0,99	HMGCS2	3-hydroxy-3-methylglutaryl-CoA synthase 2 (mitochondrial)
0,99	BCCIP	BRCA2 and CDKN1A interacting protein
0,99	PCSK4	proprotein convertase subtilisin/kexin type 4
0,99	NXT2	nuclear transport factor 2-like export factor 2
0,99	TTPAL	tocopherol (alpha) transfer protein-like
0,99	NISCH	nischarin
0,99	GSDMA	gasdermin A
0,99	LCE2A	late cornified envelope 2A
0,99	BZW2	basic leucine zipper and W2 domains 2
0,99	DNHD1	dynein heavy chain domain 1
0,99	SKIL	SKI-like proto-oncogene
0,99	OGFOD1	2-oxoglutarate and iron-dependent oxygenase domain containing 1
0,99	CPOX	coproporphyrinogen oxidase
0,99	SLC39A10	solute carrier family 39 (zinc transporter), member 10
0,99	RABEP1	rabaptin, RAB GTPase binding effector protein 1
0,99	RAB4A; SPHAR	RAB4A, member RAS oncogene family; S-phase response (cyclin related)
0,99	C21orf2	chromosome 21 open reading frame 2
0,99	ASGR1	asialoglycoprotein receptor 1
0,99	C16orf52	chromosome 16 open reading frame 52
0,99	CACNG1	calcium channel, voltage-dependent, gamma subunit 1
0,99	AEN	apoptosis enhancing nuclease
0,99	WDFY3	WD repeat and FYVE domain containing 3
0,99	GREM1	gremlin 1, DAN family BMP antagonist [Source:HGNC Symbol;Acc:HGNC:2001]
0,99	DCUN1D1	DCN1, defective in cullin neddylation 1, domain containing 1
0,99	OR4D6	olfactory receptor, family 4, subfamily D, member 6
0,99	MYH14	myosin, heavy chain 14, non-muscle
0,99	SSBP2	single-stranded DNA binding protein 2
0,99	SLC25A6	solute carrier family 25 (mitochondrial carrier; adenine nucleotide translocator), member 6
0,99	XRCC6	X-ray repair complementing defective repair in Chinese hamster cells 6
0,99	HTR3C	5-hydroxytryptamine (serotonin) receptor 3C, ionotropic

0,99	EDDM3A	epididymal protein 3A
0,99	DUSP26	dual specificity phosphatase 26 (putative)
0,99	COQ10B	coenzyme Q10B
0,99	BRAP	BRCA1 associated protein
0,99	CT45A2	cancer/testis antigen family 45, member A2
0,99	PEBP4	phosphatidylethanolamine-binding protein 4
0,99	SHC2	SHC (Src homology 2 domain containing) transforming protein 2
0,99	HPRT1	hypoxanthine phosphoribosyltransferase 1
0,99	CHEK2	checkpoint kinase 2
0,99	PARP15	poly(ADP-ribose) polymerase family member 15
0,99	ATF4	activating transcription factor 4
0,99	TRAM2	Transcript Identified by AceView, Entrez Gene ID(s) 9697
0,99	LCE2C	late cornified envelope 2C
0,99	HSD17B8	hydroxysteroid (17-beta) dehydrogenase 8
0,99	AP1B1	adaptor-related protein complex 1, beta 1 subunit
0,99	RPA3	replication protein A3
0,99	SAXO2	stabilizer of axonemal microtubules 2
0,99	RPF1	ribosome production factor 1 homolog
0,99	FAM174B	family with sequence similarity 174, member B
0,99	GLRX2	glutaredoxin 2
0,99	FAM220A	family with sequence similarity 220, member A
0,99	SMDT1	single-pass membrane protein with aspartate-rich tail 1
0,99	ABCC3	ATP binding cassette subfamily C member 3
0,99	EIF3F	Eukaryotic translation initiation factor 3 subunit F [Source:UniProtKB/Swiss-Prot;Acc:O00303]
0,99	MRPS31	mitochondrial ribosomal protein S31
0,99	USP5	ubiquitin specific peptidase 5 (isopeptidase T)
0,99	ZNF778	zinc finger protein 778
0,99	LOC102724957; RP11-396O20.1	uncharacterized LOC102724957; novel transcript
0,99	UPF2	UPF2 regulator of nonsense transcripts homolog (yeast)
0,99	IRX5	iroquois homeobox 5
0,99	RPS6KA4	ribosomal protein S6 kinase, 90kDa, polypeptide 4
0,99	MRPS26	mitochondrial ribosomal protein S26
0,99	VPS72	vacuolar protein sorting 72 homolog (S. cerevisiae)
0,99	OR10H2	olfactory receptor, family 10, subfamily H, member 2
0,99	QRSL1	glutaminyl-tRNA synthase (glutamine-hydrolyzing)-like 1
0,99	RNF185	Transcript Identified by AceView, Entrez Gene ID(s) 91445
0,99	MRPL43	mitochondrial ribosomal protein L43
0,99	PTGES3	prostaglandin E synthase 3 (cytosolic)
0,99	SESN2	sestrin 2
0,99	C1GALT1C1L	C1GALT1-specific chaperone 1 like
0,99	CDADC1	cytidine and dCMP deaminase domain containing 1

0,99	GTF2H2; GTF2H2B	general transcription factor IIH subunit 2; general transcription factor IIH subunit 2B (pseudogene)
0,99	ZNF587B	zinc finger protein 587B
0,99	EFCAB11	EF-hand calcium binding domain 11
0,99	ZNF410	zinc finger protein 410
0,98	STK32A	serine/threonine kinase 32A
0,98	AKR1B10	aldo-keto reductase family 1, member B10 (aldose reductase)
0,98	ATIC	5-aminoimidazole-4-carboxamide ribonucleotide formyltransferase/IMP cyclohydrolase
0,98	ABCC1	Jeck2013 ALT_ACCEPTOR, ALT_DONOR, coding, INTERNAL, intronic best transcript NM_004996
0,98	UBTD2	ubiquitin domain containing 2
0,98	TTC34	tetratricopeptide repeat domain 34
0,98	MAPK8IP2	mitogen-activated protein kinase 8 interacting protein 2
0,98	OR8J3	olfactory receptor, family 8, subfamily J, member 3
0,98	BMP1	bone morphogenetic protein 1
0,98	SRGAP3	SLIT-ROBO Rho GTPase activating protein 3
0,98	FST	follistatin
0,98	TAP1	transporter 1, ATP-binding cassette, sub-family B (MDR/TAP)
0,98	FDX1L	ferredoxin 1-like
0,98	EIF4EBP1	eukaryotic translation initiation factor 4E binding protein 1
0,98	FBL	fibrillarin
0,98	DNAH6	dynein, axonemal, heavy chain 6
0,98	LGALS9	lectin, galactoside-binding, soluble, 9
0,98	YIPF3	Yip1 domain family member 3
0,98	ARL6IP4	ADP-ribosylation factor like GTPase 6 interacting protein 4
0,98	MGAT4D	MGAT4 family, member D
0,98	KIRREL2	kin of IRRE like 2 (Drosophila)
0,98	MASP1	mannan-binding lectin serine peptidase 1 (C4/C2 activating component of Ra-reactive factor)
0,98	RNF2	ring finger protein 2
0,98	MLLT3	Jeck2013 ALT_ACCEPTOR, ALT_DONOR, coding, INTERNAL, intronic best transcript NM_004529
0,98	TMEM75	transmembrane protein 75
0,98	NEK9	NIMA-related kinase 9
0,98	SELO	selenoprotein O; selenoprotein O [Source:EntrezGene;Acc:83642]; Salzman2013 ANNOTATED, CDS, coding, INTERNAL, OVCODE, OVERLAPTX, OVEXON best transcript NM_031454; Salzman2013 ANNOTATED, CDS, coding, OVCODE, OVERLAPTX, OVEXON, UTR3 best transcript NM_031454; Transcript Identified by AceView, Entrez Gene ID(s) 83642, RefSeq ID(s) NM_031454
0,98	ZNF652	zinc finger protein 652
0,98	ZNF394	zinc finger protein 394

0,98	FAM104A	family with sequence similarity 104, member A
0,98	KCNH7	potassium channel, voltage gated eag related subfamily H, member 7
0,98	TMEM132C	transmembrane protein 132C
0,98	TPST1	tyrosylprotein sulfotransferase 1
0,98	MAS1L	MAS1 proto-oncogene like, G protein-coupled receptor
0,98	FCGBP	Fc fragment of IgG binding protein
0,98	GCKR	glucokinase (hexokinase 4) regulator
0,98	CA10	carbonic anhydrase X
0,98	LINC00935	long intergenic non-protein coding RNA 935
0,98	NPIPA5	nuclear pore complex interacting protein family, member A5
0,98	IGFN1	immunoglobulin-like and fibronectin type III domain containing 1
0,98	MMP16	matrix metallopeptidase 16 (membrane-inserted)
0,98	PPFIA4	protein tyrosine phosphatase, receptor type, f polypeptide (PTPRF), interacting protein (liprin), alpha 4
0,98	COPA	coatomer protein complex subunit alpha
0,98	UQCC1	ubiquinol-cytochrome c reductase complex assembly factor 1
0,98	C18orf54	chromosome 18 open reading frame 54
0,98	COG3	component of oligomeric golgi complex 3
0,98	CCDC130	coiled-coil domain containing 130
0,98	ZNF674	zinc finger protein 674
0,98	VNN1	vanin 1
0,98	SMIM22	small integral membrane protein 22
0,98	STAT5B	signal transducer and activator of transcription 5B
0,98	TAAR1	trace amine associated receptor 1
0,98	GREB1	growth regulation by estrogen in breast cancer 1
0,98	TRAPPC6B	trafficking protein particle complex 6B
0,98	UFC1	ubiquitin-fold modifier conjugating enzyme 1
0,98	ZDHHC3	zinc finger, DHHC-type containing 3
0,98	HIST1H2AG	histone cluster 1, H2ag
0,98	UQCRQ	ubiquinol-cytochrome c reductase complex III subunit VII
0,98	FHOD3	formin homology 2 domain containing 3
0,98	SLC30A2	solute carrier family 30 (zinc transporter), member 2
0,98	LCE2B	late cornified envelope 2B
0,98	XAB2	XPA binding protein 2
0,98	SDF2L1	stromal cell-derived factor 2-like 1
0,98	ZCCHC9	zinc finger, CCHC domain containing 9
0,98	USP37	ubiquitin specific peptidase 37
0,98	CRISPLD1	cysteine-rich secretory protein LCCL domain containing 1
0,98	CBARP	calcium channel, voltage-dependent, beta subunit associated regulatory protein
0,98	SNRPD2	small nuclear ribonucleoprotein D2 polypeptide
0,98	LAPTM4A	lysosomal protein transmembrane 4 alpha

0,98	FBXW8	F-box and WD repeat domain containing 8
0,98	ACKR2	atypical chemokine receptor 2
0,98	ITGA4	integrin alpha 4
0,98	BCL2L14	BCL2-like 14 (apoptosis facilitator)
0,98	ARIH1; MIR630	ariadne RBR E3 ubiquitin protein ligase 1; microRNA 630
0,98	RASGRP1	RAS guanyl releasing protein 1 (calcium and DAG-regulated)
0,98	ATP2B1	ATPase, Ca++ transporting, plasma membrane 1
0,98	FAM214A	family with sequence similarity 214, member A
0,98	ATP5J2	ATP synthase, H+ transporting, mitochondrial Fo complex subunit F2
0,98	MSS51	MSS51 mitochondrial translational activator
0,98	HENMT1	HEN1 methyltransferase homolog 1 (Arabidopsis)
0,98	CCDC154	coiled-coil domain containing 154
0,98	TSPAN3	tetraspanin 3
0,98	FGG	fibrinogen gamma chain
0,98	MORN5	MORN repeat containing 5
0,98	OR4N5	olfactory receptor, family 4, subfamily N, member 5
0,98	CORO1A	coronin, actin binding protein, 1A
0,98	KNOP1	lysine-rich nucleolar protein 1
0,98	MCOLN1	mucolipin 1
0,98	CLEC18B	C-type lectin domain family 18, member B
0,98	STAP2	signal transducing adaptor family member 2
0,98	SLC8A1	solute carrier family 8 (sodium/calcium exchanger), member 1
0,98	GZMB	granzyme B
0,98	SYT2	synaptotagmin II
0,98	PSMA2	proteasome subunit alpha 2
0,98	CD46	CD46 molecule, complement regulatory protein
0,98	STEAP3	STEAP family member 3, metalloreductase
0,98	TRAPPC4	trafficking protein particle complex 4
0,98	PEAK1	pseudopodium-enriched atypical kinase 1
0,98	MCM5	minichromosome maintenance complex component 5
0,98	IQCJ; SCHIP1; IQCJ-SCHIP1	IQ motif containing J; schwannomin interacting protein 1; IQCJ-SCHIP1 readthrough
0,98	RCC1; SNORA73A; SNHG3; SNORA73B	regulator of chromosome condensation 1; small nucleolar RNA, H/ACA box 73A; small nucleolar RNA host gene 3; small nucleolar RNA, H/ACA box 73B
0,98	ZNF780B	zinc finger protein 780B
0,98	GPR65	G protein-coupled receptor 65
0,98	PINK1; MIR6084	PTEN induced putative kinase 1; microRNA 6084
0,98	SNRPE	small nuclear ribonucleoprotein polypeptide E
0,98	PIK3C3	phosphatidylinositol 3-kinase, catalytic subunit type 3
0,98	ANP32A	acidic nuclear phosphoprotein 32 family member A
0,98	LINC00982	long intergenic non-protein coding RNA 982
0,98	SLC25A26	solute carrier family 25 (S-adenosylmethionine carrier), member 26
0,98	ACO1	aconitase 1, soluble

0,98	TLE4	transducin-like enhancer of split 4
0,98	IL20	interleukin 20
0,98	USP3	ubiquitin specific peptidase 3
0,98	HK1	hexokinase 1
0,98	NKX2-5	NK2 homeobox 5
0,98	APOBEC3A	apolipoprotein B mRNA editing enzyme, catalytic polypeptide-like 3A
0,98	CTBP1	C-terminal binding protein 1
0,98	RPS25	ribosomal protein S25
0,98	LCLAT1	lysocardiolipin acyltransferase 1
0,98	RAB35	RAB35, member RAS oncogene family
0,98	PRSS46	protease, serine 46
0,98	ZNF217	zinc finger protein 217
0,98	ARL8B	ADP-ribosylation factor like GTPase 8B
0,98	NR1H2	nuclear receptor subfamily 1, group H, member 2
0,98	RASL10B	RAS-like, family 10, member B
0,98	TSPYL2	TSPY-like 2
0,98	ACVR1B	activin A receptor type IB
0,98	ZNF420	zinc finger protein 420
0,98	AKIRIN2	akirin 2
0,98	PSMG2	proteasome (prosome, macropain) assembly chaperone 2
0,98	EDRF1	erythroid differentiation regulatory factor 1
0,98	GPR61	G protein-coupled receptor 61
0,98	SPRR2D	small proline-rich protein 2D
0,98	CELSR2	cadherin, EGF LAG seven-pass G-type receptor 2
0,98	FNTB	farnesyltransferase, CAAX box, beta
0,98	DEFB103A; DEFB103B	defensin, beta 103A; defensin, beta 103B
0,98	RRP8	ribosomal RNA processing 8, methyltransferase, homolog (yeast)
0,98	SIRPB2	signal-regulatory protein beta 2
0,98	LUZP6; MTPN	leucine zipper protein 6; myotrophin
0,98	HIST1H1E	histone cluster 1, H1e
0,98	DNAL4	dynein, axonemal, light chain 4
0,98	CD300LG	CD300 molecule-like family member g
0,98	B2M	beta-2-microglobulin
0,98	UBAP2; SNORD121A; SNORD121B	ubiquitin associated protein 2; small nucleolar RNA, C/D box 121A; small nucleolar RNA, C/D box 121B
0,98	STRA6	stimulated by retinoic acid 6
0,98	ST3GAL2	ST3 beta-galactoside alpha-2,3-sialyltransferase 2
0,98	ZNF585A	zinc finger protein 585A
0,98	TSSC4	tumor suppressing subtransferable candidate 4
0,98	ID1	inhibitor of DNA binding 1, dominant negative helix-loop-helix protein
0,98	PITPNA	phosphatidylinositol transfer protein, alpha
0,98	ZBTB7B	zinc finger and BTB domain containing 7B
0,98	NXPH4	neurexophilin 4

0,98	SYDE1	synapse defective 1, Rho GTPase, homolog 1 (C. elegans)
0,98	SYF2	SYF2 pre-mRNA-splicing factor
0,98	NHLRC3	NHL repeat containing 3
0,98	ARVCF	armadillo repeat gene deleted in velocardiofacial syndrome
0,98	RANBP3L	RAN binding protein 3-like
0,98	EIF3I	eukaryotic translation initiation factor 3, subunit I
0,98	ZC3H8	zinc finger CCCH-type containing 8
0,98	YPEL3	yippee like 3
0,98	DLC1	DLC1 Rho GTPase activating protein
0,98	NT5C3B	5-nucleotidase, cytosolic IIIB
0,98	SLC16A13	solute carrier family 16, member 13
0,98	GALNTL6	Salzman2013 ANTISENSE, coding, INTERNAL, intronic best transcript NM_001034845
0,98	FDPS	farnesyl diphosphate synthase
0,98	TCF15	transcription factor 15 (basic helix-loop-helix)
0,98	TIPIN; RPL9P25	TIMELESS interacting protein; ribosomal protein L9 pseudogene 25
0,98	ATHL1	ATH1, acid trehalase-like 1 (yeast)
0,98	GBAS	glioblastoma amplified sequence
0,98	LAMA4	laminin, alpha 4
0,98	POU6F1	POU class 6 homeobox 1
0,98	PTPRC	protein tyrosine phosphatase, receptor type, C
0,98	EFCC1	EF-hand and coiled-coil domain containing 1
0,98	NUS1	NUS1 dehydrodolichyl diphosphate synthase subunit
0,98	ATP5EP2	ATP synthase, H+ transporting, mitochondrial F1 complex, epsilon subunit pseudogene 2
0,98	MICALL1	MICAL-like 1
0,98	BAIAP2	BAI1-associated protein 2
0,98	FAM72D	family with sequence similarity 72, member D
0,98	CCR3	chemokine (C-C motif) receptor 3
0,98	MIS18BP1	MIS18 binding protein 1
0,98	ZC3H13	zinc finger CCCH-type containing 13
0,98	FAM213B	family with sequence similarity 213, member B
0,98	ATP6V1G3	ATPase, H+ transporting, lysosomal 13kDa, V1 subunit G3
0,98	RYR1	ryanodine receptor 1 (skeletal)
0,98	LOC100130705; RP11-309L24.4	uncharacterized LOC100130705; putative novel transcript
0,98	ST3GAL4	ST3 beta-galactoside alpha-2,3-sialyltransferase 4
0,98	PML	promyelocytic leukemia
0,98	HS1BP3	HCLS1 binding protein 3
0,98	PCGF3	polycomb group ring finger 3
0,98	ZCCHC2	zinc finger, CCHC domain containing 2
0,98	CSNK1E	casein kinase 1, epsilon
0,98	PDE6D	phosphodiesterase 6D, cGMP-specific, rod, delta
0,98	TCTN2	tectonic family member 2

0,98	MATR3; SNORA74A; SNHG4	matrin 3; small nucleolar RNA, H/ACA box 74A; small nucleolar RNA host gene 4
0,98	RPL22L1	ribosomal protein L22-like 1
0,98	KLHDC2	kelch domain containing 2
0,98	LMNB2; MIR7108	lamin B2; microRNA 7108
0,98	C2orf47	chromosome 2 open reading frame 47
0,98	C18orf63	chromosome 18 open reading frame 63
0,98	CALML6	calmodulin-like 6
0,98	SNPH	syntaphilin
0,98	TEX13B	testis expressed 13B
0,98	EGFR	epidermal growth factor receptor
0,98	SLCO1A2	solute carrier organic anion transporter family, member 1A2
0,98	TRDN	triadin
0,98	CCDC38	coiled-coil domain containing 38
0,98	CRACR2B	calcium release activated channel regulator 2B
0,98	KANSL1L	KAT8 regulatory NSL complex subunit 1 like
0,98	GZF1	GDNF-inducible zinc finger protein 1
0,98	NR2C2	nuclear receptor subfamily 2, group C, member 2
0,98	OR1F1	olfactory receptor, family 1, subfamily F, member 1
0,98	NEGR1	neuronal growth regulator 1
0,98	ERAP1	endoplasmic reticulum aminopeptidase 1
0,98	KRTAP10-7; KRTAP10-8	keratin associated protein 10-7; keratin associated protein 10-8
0,98	SNRPD2P2	small nuclear ribonucleoprotein D2 pseudogene 2
0,98	IL34	interleukin 34
0,98	PHYHIP	phytanoyl-CoA 2-hydroxylase interacting protein
0,98	KIF21A	kinesin family member 21A
0,98	TRIM54	tripartite motif containing 54
0,98	ADAM17	ADAM metallopeptidase domain 17
0,98	KHDC1L	KH homology domain containing 1-like
0,98	DOLPP1	dolichyldiphosphatase 1
0,98	PSMD2	proteasome 26S subunit, non-ATPase 2
0,98	SAV1	salvador family WW domain containing protein 1
0,98	MSX2	msh homeobox 2
0,98	TFIP11	tuftelin interacting protein 11
0,98	DMWD	dystrophia myotonica, WD repeat containing
0,98	TMEM199; MIR4723	transmembrane protein 199; microRNA 4723
0,98	UBA52	ubiquitin A-52 residue ribosomal protein fusion product 1
0,98	UFD1L	ubiquitin fusion degradation 1 like (yeast)
0,98	MST1L	macrophage stimulating 1-like
0,98	RBM8A	RNA binding motif protein 8A
0,98	PINLYP	phospholipase A2 inhibitor and LY6/PLAUR domain containing
0,98	RUNX1T1	runt-related transcription factor 1; translocated to, 1 (cyclin D-related)

0,98	ASB3	ankyrin repeat and SOCS box containing 3
0,98	ASB13	ankyrin repeat and SOCS box containing 13
0,98	KPNA3	Transcript Identified by AceView, Entrez Gene ID(s) 3839
0,98	AVPR1A	arginine vasopressin receptor 1A
0,98	GLMP	glycosylated lysosomal membrane protein
0,98	CNNM4	cyclin and CBS domain divalent metal cation transport mediator 4
0,98	ASPHD1	aspartate beta-hydroxylase domain containing 1
0,98	FGF23	fibroblast growth factor 23
0,98	MPRIP	myosin phosphatase Rho interacting protein
0,98	DIEXF	digestive organ expansion factor homolog (zebrafish)
0,98	LPAL2	lipoprotein, Lp(a)-like 2, pseudogene
0,98	CDH26	cadherin 26
0,98	CCDC7	coiled-coil domain containing 7
0,98	UPB1	ureidopropionase, beta
0,98	CCDC106	coiled-coil domain containing 106
0,98	SLFN12	schlafen family member 12
0,98	RLF	rearranged L-myc fusion
0,98	PABPC1L2B	poly(A) binding protein, cytoplasmic 1-like 2B
0,98	CHPF	chondroitin polymerizing factor
0,98	FIS1	fission, mitochondrial 1
0,98	GCSAM	germinal center-associated, signaling and motility
0,98	SST	somatostatin
0,98	NRP1	neuropilin 1
0,98	ZBTB44	zinc finger and BTB domain containing 44
0,98	ZNF606	zinc finger protein 606
0,98	EEF1D	eukaryotic translation elongation factor 1 delta (guanine nucleotide exchange protein)
0,98	TFB2M	transcription factor B2, mitochondrial
0,98	CRYGS	crystallin gamma S
0,98	A3GALT2	alpha 1,3-galactosyltransferase 2
0,98	LSM10	LSM10, U7 small nuclear RNA associated
0,98	ORAOV1	oral cancer overexpressed 1
0,98	CNKSR1	connector enhancer of kinase suppressor of Ras 1
0,98	SURF4	surfeit 4
0,98	RSL24D1	ribosomal L24 domain containing 1
0,98	TRO	trophinin
0,98	PUS7L	pseudouridylate synthase 7-like
0,98	ZNF558	zinc finger protein 558
0,98	RPL4; SNORD16; SNORD18A; SNORD18B; SNORD18C	ribosomal protein L4; small nucleolar RNA, C/D box 16; small nucleolar RNA, C/D box 18A; small nucleolar RNA, C/D box 18B; small nucleolar RNA, C/D box 18C
0,98	RARRES1	retinoic acid receptor responder (tazarotene induced) 1
0,98	NHLRC2	NHL repeat containing 2

0,98	PPP6R3	Memczak2013 ALT_ACCEPTOR, ALT_DONOR, coding, INTERNAL, intronic best transcript NM_001164162
0,98	DNAJC10	DnaJ (Hsp40) homolog, subfamily C, member 10
0,98	COL21A1	collagen, type XXI, alpha 1
0,98	BLVRB	biliverdin reductase B
0,98	LMNB1	lamin B1
0,98	SAGE1	sarcoma antigen 1
0,98	CD68	CD68 molecule
0,98	ATP5A1	ATP synthase, H+ transporting, mitochondrial F1 complex, alpha subunit 1, cardiac muscle
0,98	DHCR24	24-dehydrocholesterol reductase
0,98	HSPB9	heat shock protein, alpha-crystallin-related, B9
0,98	HIST1H2AA	histone cluster 1, H2aa
0,98	SERTAD4	SERTA domain containing 4
0,98	ACER2	alkaline ceramidase 2
0,98	SPTBN2	spectrin, beta, non-erythrocytic 2
0,98	TBCCD1	TBCC domain containing 1
0,98	DAP3	death associated protein 3
0,98	SCAF1	SR-related CTD-associated factor 1
0,98	IVD	isovaleryl-CoA dehydrogenase
0,98	TDRD10	tudor domain containing 10
0,98	IFT22	intraflagellar transport 22
0,98	PNKP	polynucleotide kinase 3-phosphatase
0,98	APOC2	apolipoprotein C-II
0,98	ANXA4	annexin A4
0,98	SNAPC5	small nuclear RNA activating complex polypeptide 5
0,98	PELP1	proline, glutamate and leucine rich protein 1
0,98	MSRB1	methionine sulfoxide reductase B1
0,98	CCNA2	cyclin A2
0,98	TEKT5	tektin 5
0,98	PLD5	phospholipase D family, member 5
0,98	OR6C75	olfactory receptor, family 6, subfamily C, member 75
0,98	FBL	fibrillarin
0,98	CEP41	centrosomal protein 41kDa
0,98	ZNF273	zinc finger protein 273
0,98	RAB12	RAB12, member RAS oncogene family
0,98	HBS1L	HBS1-like translational GTPase
0,98	ZBTB26	zinc finger and BTB domain containing 26
0,98	PIGO	phosphatidylinositol glycan anchor biosynthesis class O
0,98	C1orf233	chromosome 1 open reading frame 233
0,98	WDTC1	WD and tetratricopeptide repeats 1
0,98	VAV1	vav 1 guanine nucleotide exchange factor
0,98	RPUSD2	RNA pseudouridylate synthase domain containing 2
0,98	ZNF766; MIR643	zinc finger protein 766; microRNA 643
0,98	HTN1	histatin 1
0,98	FBRS	fibrosin
0,98	SSNA1	Sjogren syndrome nuclear autoantigen 1
0,98	C16orf72	chromosome 16 open reading frame 72

0,98	TBC1D15	TBC1 domain family, member 15
0,98	RAMP2	receptor (G protein-coupled) activity modifying protein 2
0,98	TSPO	translocator protein (18kDa)
0,98	EIF4G3	eukaryotic translation initiation factor 4 gamma, 3
0,98	RPL17-C18orf32	RPL17-C18orf32 readthrough
0,98	POC1A	POC1 centriolar protein A
0,98	IL7	interleukin 7
0,98	KIF18A	kinesin family member 18A
0,98	FAM20B	family with sequence similarity 20, member B
0,98	SPINT1	serine peptidase inhibitor, Kunitz type 1
0,98	ADGRE2	adhesion G protein-coupled receptor E2
0,98	ZNF678	Transcript Identified by AceView, Entrez Gene ID(s) 339500
0,98	LAMP1	lysosomal-associated membrane protein 1
0,98	DAD1	defender against cell death 1
0,98	EMSY	EMSY BRCA2-interacting transcriptional repressor
0,98	PKD2L1	polycystic kidney disease 2-like 1
0,98	RPL3; SNORD83B; SNORD43	ribosomal protein L3; small nucleolar RNA, C/D box 83B; small nucleolar RNA, C/D box 43
0,98	IL21R	interleukin 21 receptor
0,98	MORN2	MORN repeat containing 2
0,98	POLR1E	polymerase (RNA) I polypeptide E
0,98	ARHGDIA	Rho GDP dissociation inhibitor (GDI) alpha
0,98	DNAH9	dynein, axonemal, heavy chain 9
0,98	TNIP2	TNFAIP3 interacting protein 2
0,98	YTHDF1	YTH N(6)-methyladenosine RNA binding protein 1
0,98	CT83	cancer/testis antigen 83
0,98	FPGS	folylpolyglutamate synthase
0,98	FSCN3	fascin actin-bundling protein 3, testicular
0,98	DNAJC15	DnaJ (Hsp40) homolog, subfamily C, member 15
0,98	SPIRE2	spire-type actin nucleation factor 2
0,98	AGTPBP1	ATP/GTP binding protein 1
0,98	PCCA	propionyl-CoA carboxylase alpha subunit
0,98	PAIP1	poly(A) binding protein interacting protein 1
0,98	NSG1; D4S234E	neuron specific gene family member 1; Neuron-specific protein family member 1 [Source:UniProtKB/Swiss-Prot;Acc:P42857]
0,98	STARD8	StAR-related lipid transfer domain containing 8
0,98	MCEE	methylmalonyl CoA epimerase
0,98	GAD1	glutamate decarboxylase 1
0,98	NBPF8	neuroblastoma breakpoint family, member 8
0,98	KIAA1715	KIAA1715
0,98	NAP1L4	nucleosome assembly protein 1-like 4
0,98	PRRG1	proline rich Gla (G-carboxyglutamic acid) 1
0,98	FCRL5	Fc receptor-like 5
0,98	PRR18	proline rich 18
0,98	EXOC6B	exocyst complex component 6B
0,98	DAGLA	diacylglycerol lipase, alpha

0,98	MTHFR	methylenetetrahydrofolate reductase (NAD(P)H)
0,98	NT5DC2	5-nucleotidase domain containing 2
0,98	LPAR4	lysophosphatidic acid receptor 4
0,98	HHAT	hedgehog acyltransferase
0,98	SOX4	SRY box 4
0,98	YRDC	yrdC N(6)-threonylcarbamoyltransferase domain containing
0,98	LRIG1	
0,98	PRDX2	peroxiredoxin 2
0,98	ARF5	ADP-ribosylation factor 5
0,98	ARFGEF3	ARFGEF family member 3
0,98	RAB5C	RAB5C, member RAS oncogene family
0,98	SEC24D	SEC24 homolog D, COPII coat complex component
0,98	EGLN2	egl-9 family hypoxia-inducible factor 2
0,98	AMOTL2; MIR6827; RPL39P5	angiomotin like 2; microRNA 6827; ribosomal protein L39 pseudogene 5
0,98	PRAMEF11	PRAME family member 11
0,98	NR1H2	nuclear receptor subfamily 1, group H, member 2
0,98	ERVW-1	endogenous retrovirus group W, member 1
0,98	SSX4B	synovial sarcoma, X breakpoint 4B
0,98	GPATCH2	G-patch domain containing 2
0,98	ODF2	outer dense fiber of sperm tails 2
0,98	RBM17	RNA binding motif protein 17
0,98	DSCR3	Down syndrome critical region 3
0,98	PGAM1	phosphoglycerate mutase 1 (brain)
0,98	PRND	prion protein 2 (dublet)
0,98	CTU1	cytosolic thiouridylase subunit 1
0,98	DNPH1	2-deoxynucleoside 5-phosphate N-hydrolase 1
0,98	SH2B3	SH2B adaptor protein 3
0,98	HIST1H4D	histone cluster 1, H4d
0,98	MIR650; IGLV3-9; IGLV2-8	microRNA 650; immunoglobulin lambda variable 3-9 (gene/pseudogene); immunoglobulin lambda variable 2-8
0,98	STOM	stomatin
0,98	SCD	stearoyl-CoA desaturase (delta-9-desaturase)
0,98	SPG20	spastic paraplegia 20 (Troyer syndrome)
0,98	CPLX1	complexin 1
0,98	LSM14A	LSM14A mRNA processing body assembly factor
0,98	GMFB	glia maturation factor, beta
0,98	VIPR1	vasoactive intestinal peptide receptor 1
0,98	RCL1	RNA terminal phosphate cyclase-like 1
0,98	DCDC2B	doublecortin domain containing 2B
0,98	NSUN3	NOP2/Sun domain family, member 3
0,98	SURF4	surfeit 4
0,98	STRN3	striatin, calmodulin binding protein 3
0,98	DUSP12	dual specificity phosphatase 12
0,98	MELK	maternal embryonic leucine zipper kinase
0,98	TOMM40L; MIR5187	translocase of outer mitochondrial membrane 40 homolog (yeast)-like; microRNA 5187

0,98	CASP9	caspase 9
0,98	COPS5	COP9 signalosome subunit 5
0,98	SIPA1L3	signal-induced proliferation-associated 1 like 3
0,98	ATP5F1	ATP synthase, H+ transporting, mitochondrial Fo complex subunit B1
0,98	VWA8; MIR5006	von Willebrand factor A domain containing 8; microRNA 5006
0,98	ANKRD52	Zhang2013 ALT_ACCEPTOR, ALT_DONOR, coding, INTERNAL, intronic best transcript NM_173595
0,98	CATSPERG	catsper channel auxiliary subunit gamma
0,98	BIK	BCL2-interacting killer (apoptosis-inducing)
0,98	C4orf33	chromosome 4 open reading frame 33
0,98	TUBB4A	tubulin, beta 4A class IVa
0,98	MICU3	mitochondrial calcium uptake family, member 3
0,98	SUOX	sulfite oxidase
0,98	COQ7	coenzyme Q7 homolog, ubiquinone (yeast)
0,98	TRIM49D2; TRIM49D1	tripartite motif containing 49D2; tripartite motif containing 49D1
0,98	NOC3L	NOC3-like DNA replication regulator
0,98	SOST	sclerostin
0,98	C15orf57; MRPL42P5	chromosome 15 open reading frame 57; mitochondrial ribosomal protein L42 pseudogene 5
0,98	EIF3C; EIF3CL	eukaryotic translation initiation factor 3, subunit C; eukaryotic translation initiation factor 3, subunit C-like
0,98	RBL2	retinoblastoma-like 2
0,98	KY	kyphoscoliosis peptidase
0,98	GNPTG	N-acetylglucosamine-1-phosphate transferase, gamma subunit
0,98	EIF4B	eukaryotic translation initiation factor 4B
0,98	ZNF687	zinc finger protein 687
0,98	PQLC1	PQ loop repeat containing 1
0,98	THBS1	thrombospondin 1
0,98	CGB7	chorionic gonadotropin, beta polypeptide 7
0,98	CTNNA2	catenin (cadherin-associated protein), alpha 2
0,98	FAM178B	family with sequence similarity 178, member B
0,98	SEC22B	SEC22 homolog B, vesicle trafficking protein (gene/pseudogene)
0,98	SPOP	speckle-type POZ protein
0,98	NBPF1	neuroblastoma breakpoint family, member 1
0,98	DCC	DCC netrin 1 receptor
0,98	AMPD2	adenosine monophosphate deaminase 2
0,98	C7orf65	chromosome 7 open reading frame 65
0,98	NPTXR	neuronal pentraxin receptor
0,98	RPL13A; SNORD35A; SNORD34; SNORD33; SNORD32A	ribosomal protein L13a; small nucleolar RNA, C/D box 35A; small nucleolar RNA, C/D box 34; small nucleolar RNA, C/D box 33; small nucleolar RNA, C/D box 32A
0,98	FAM83H-AS1	FAM83H antisense RNA 1 (head to head)

0,98	TTLL5	tubulin tyrosine ligase-like family member 5
0,98	SRPRB	signal recognition particle receptor, B subunit
0,98	RIOK3	RIO kinase 3
0,98	GRPEL2	GrpE-like 2, mitochondrial (E. coli)
0,98	CLDN20	claudin 20
0,98	NDST3	N-deacetylase/N-sulfotransferase (heparan glucosaminyl) 3
0,98	HYPK	huntingtin interacting protein K
0,98	FAM50A	family with sequence similarity 50, member A
0,98	TBX2	T-box 2
0,98	HLA-V	major histocompatibility complex, class I, V (pseudogene)
0,98	CKB	creatine kinase, brain
0,98	FLYWCH1	Transcript Identified by AceView, Entrez Gene ID(s) 84256
0,98	HIST2H3A	histone cluster 2, H3a
0,98	ABHD11	abhydrolase domain containing 11
0,98	M6PR	mannose-6-phosphate receptor (cation dependent)
0,98	DUSP21	dual specificity phosphatase 21
0,98	CSTF1	cleavage stimulation factor, 3 pre-RNA, subunit 1
0,98	CX3CL1	chemokine (C-X3-C motif) ligand 1
0,98	RNF6	ring finger protein (C3H2C3 type) 6
0,98	LRRC28	leucine rich repeat containing 28
0,98	ANKRD40	ankyrin repeat domain 40
0,98	sep-02	septin 2
0,98	CDC20B	cell division cycle 20B
0,98	ZNF256	zinc finger protein 256
0,98	SMCO1	single-pass membrane protein with coiled-coil domains 1
0,98	TMEM115	transmembrane protein 115
0,98	GLIS3	GLIS family zinc finger 3
0,98	BRS3	bombesin-like receptor 3
0,98	FBXO36	F-box protein 36
0,98	EBF4	early B-cell factor 4
0,98	EAF1	ELL associated factor 1
0,98	ZNF580; ZNF581	zinc finger protein 580; zinc finger protein 581
0,98	RELN	reelin
0,98	MED6	mediator complex subunit 6
0,98	RNF17	ring finger protein 17
0,98	sep-12	septin 12
0,98	EHBP1L1	EH domain binding protein 1 like 1
0,98	ZMAT4	zinc finger, matrin-type 4
0,98	RSF1	remodeling and spacing factor 1
0,98	HINFP	histone H4 transcription factor
0,98	C7orf55-LUC7L2; LUC7L2; C7orf55	C7orf55-LUC7L2 readthrough; LUC7-like 2 pre-mRNA splicing factor; chromosome 7 open reading frame 55
0,98	ITPRIPL2	inositol 1,4,5-trisphosphate receptor interacting protein-like 2

0,98	PIEZO1	piezo-type mechanosensitive ion channel component 1
0,98	JTB	jumping translocation breakpoint
0,98	FAM201A	family with sequence similarity 201, member A
0,98	SERPINB10	serpin peptidase inhibitor, clade B (ovalbumin), member 10
0,98	BPY2B	basic charge, Y-linked, 2B
0,98	LILRA1	leukocyte immunoglobulin-like receptor, subfamily A (with TM domain), member 1
0,98	CAT	catalase
0,98	NEDD9	Memczak2013 ALT_ACCEPTOR, ALT_DONOR, coding, INTERNAL, intronic best transcript NM_001142393
0,98	ST6GALNAC4	ST6 (alpha-N-acetyl-neuraminyl-2,3-beta-galactosyl-1,3)-N-acetylgalactosaminide alpha-2,6-sialyltransferase 4
0,98	MESDC2	mesoderm development candidate 2
0,98	LRRC59	leucine rich repeat containing 59
0,98	KLHL20	kelch-like family member 20
0,98	GIPR	gastric inhibitory polypeptide receptor
0,98	MAGEA9; MAGEA9B	MAGE family member A9; MAGE family member A9B
0,98	BIN3	bridging integrator 3
0,98	HS3ST2	heparan sulfate (glucosamine) 3-O-sulfotransferase 2
0,98	RPL36AL	ribosomal protein L36a-like
0,98	DDX6	DEAD (Asp-Glu-Ala-Asp) box helicase 6
0,98	UNC13B	unc-13 homolog B (C. elegans)
0,98	KCNJ6	potassium channel, inwardly rectifying subfamily J, member 6
0,98	NEBL	nebulette
0,98	SLC16A14	solute carrier family 16, member 14
0,98	DHX57	DEAH (Asp-Glu-Ala-Asp/His) box polypeptide 57
0,98	TMPRSS2	transmembrane protease, serine 2
0,98	ART4	ADP-ribosyltransferase 4 (Dombrock blood group)
0,98	NUCB2	nucleobindin 2
0,98	ACOT9	acyl-CoA thioesterase 9
0,98	FOXB2	forkhead box B2
0,98	MANSC1	MANSC domain containing 1
0,98	G3BP1	GTPase activating protein (SH3 domain) binding protein 1
0,98	FBXO7	F-box protein 7
0,98	TDG	thymine DNA glycosylase
0,98	LBP	lipopolysaccharide binding protein
0,98	B4GALNT1	beta-1,4-N-acetyl-galactosaminyl transferase 1
0,98	OR2L5	olfactory receptor, family 2, subfamily L, member 5
0,98	NKAIN3	Jeck2013 ANTISENSE, coding, INTERNAL, intronic best transcript NM_173688
0,98	DRAM1	DNA-damage regulated autophagy modulator 1
0,98	EIF2S3	eukaryotic translation initiation factor 2, subunit 3 gamma, 52kDa

0,98	NOMO1	NODAL modulator 1
0,98	OR8K3	olfactory receptor, family 8, subfamily K, member 3 (gene/pseudogene)
0,98	LOC100287728; RP11-85L21.4; jerva	uncharacterized LOC100287728; Transcript Identified by AceView; novel transcript
0,98	NDUFS6	NADH dehydrogenase (ubiquinone) Fe-S protein 6, 13kDa (NADH-coenzyme Q reductase)
0,98	NAA25	N(alpha)-acetyltransferase 25, NatB auxiliary subunit
0,98	SLC2A1	solute carrier family 2 (facilitated glucose transporter), member 1
0,98	TSNAX-DISC1	TSNAX-DISC1 readthrough (NMD candidate)
0,98	ATPAF1	ATP synthase mitochondrial F1 complex assembly factor 1
0,98	SDAD1	SDA1 domain containing 1
0,98	FBXO22	F-box protein 22
0,98	WDR11	WD repeat domain 11
0,98	COX11	COX11 cytochrome c oxidase copper chaperone
0,98	SLC15A3	solute carrier family 15 (oligopeptide transporter), member 3
0,98	ZNF17	zinc finger protein 17
0,98	ZNF728	zinc finger protein 728
0,98	MARCKSL1	MARCKS-like 1
0,98	LEUTX	leucine twenty homeobox
0,98	OR2A12	olfactory receptor, family 2, subfamily A, member 12
0,98	CT45A3; CT45A4; CT45A5	cancer/testis antigen family 45, member A3; cancer/testis antigen family 45, member A4; cancer/testis antigen family 45, member A5
0,98	PIGY; PYURF	phosphatidylinositol glycan anchor biosynthesis class Y; PIGY upstream reading frame
0,98	DLG4	discs, large homolog 4 (Drosophila)
0,98	CPEB4	cytoplasmic polyadenylation element binding protein 4
0,98	IL12A	interleukin 12A
0,98	SHANK2	SH3 and multiple ankyrin repeat domains 2
0,98	PRIM2	Salzman2013 ANNOTATED, CDS, coding, OVCODE, OVEXON, UTR3 best transcript NM_000947
0,98	CEP85	centrosomal protein 85kDa
0,98	OR5M11	olfactory receptor, family 5, subfamily M, member 11
0,98	MAGEA11	MAGE family member A11
0,98	AKNAD1	AKNA domain containing 1
0,98	NAPSA	napsin A aspartic peptidase
0,98	MAP7D3	MAP7 domain containing 3
0,98	CENPN	centromere protein N
0,98	SPRY4	sprouty RTK signaling antagonist 4
0,98	NR2F6	nuclear receptor subfamily 2, group F, member 6
0,98	GFPT1	glutamine--fructose-6-phosphate transaminase 1
0,98	EMG1	EMG1 N1-specific pseudouridine methyltransferase
0,98	YAP1	Yes-associated protein 1

0,98	NOL11	nucleolar protein 11
0,98	TPRX1	tetra-peptide repeat homeobox 1
0,98	ADGRG2	adhesion G protein-coupled receptor G2
0,98	KCNJ1	potassium channel, inwardly rectifying subfamily J, member 1
0,98	KTN1	kinectin 1 (kinesin receptor)
0,98	HSF2BP	heat shock transcription factor 2 binding protein
0,98	CLPB	ClpB homolog, mitochondrial AAA ATPase chaperonin
0,98	DYNLT1	dynein, light chain, Tctex-type 1
0,98	FOXI1	forkhead box I1
0,98	CLOCK	clock circadian regulator
0,98	TSPO	Memczak2013 ANTISENSE, CDS, coding, INTERNAL best transcript NM_000714
0,98	ALYREF	Aly/REF export factor
0,98	ZNF316	zinc finger protein 316
0,98	BCL2L12	BCL2-like 12 (proline rich)
0,98	SSR2	signal sequence receptor, beta (translocon-associated protein beta)
0,98	GRTP1	growth hormone regulated TBC protein 1
0,98	GDI1	GDP dissociation inhibitor 1
0,98	NOA1	nitric oxide associated 1
0,98	SERPINH1	serpin peptidase inhibitor, clade H (heat shock protein 47), member 1, (collagen binding protein 1)
0,98	CFAP100	cilia and flagella associated protein 100
0,98	DBI	diazepam binding inhibitor (GABA receptor modulator, acyl-CoA binding protein)
0,98	NSUN5P1	NOP2/Sun domain family, member 5 pseudogene 1
0,98	AK1	adenylate kinase 1
0,98	PCNXL4	pecanex-like 4 (Drosophila)
0,98	TNFSF13B	Transcript Identified by AceView, Entrez Gene ID(s) 10673
0,98	ARFGAP3	ADP-ribosylation factor GTPase activating protein 3
0,98	PATE2	prostate and testis expressed 2
0,98	VSTM4	V-set and transmembrane domain containing 4
0,98	ING4	inhibitor of growth family member 4
0,98	C18orf8	chromosome 18 open reading frame 8
0,98	LOC102467081; CTD-2313F11.1; slygee	uncharacterized LOC102467081; novel transcript, antisense to GZMK; Transcript Identified by AceView
0,98	JAM2	junctional adhesion molecule 2
0,98	BLOC1S4	biogenesis of lysosomal organelles complex-1, subunit 4, cappuccino
0,98	MRPS24	mitochondrial ribosomal protein S24
0,98	EXOC8	exocyst complex component 8
0,98	C16orf90	chromosome 16 open reading frame 90
0,98	HBM	hemoglobin, mu
0,98	FAM53B	Memczak2013 ALT_ACCEPTOR, ALT_DONOR, coding, INTERNAL, intronic best transcript NM_014661

0,98	PRR26	proline rich 26
0,98	OR4Q2	olfactory receptor, family 4, subfamily Q, member 2 (gene/pseudogene)
0,98	KLC1	kinesin light chain 1
0,98	ATR	ATR serine/threonine kinase
0,98	CDK8	cyclin-dependent kinase 8
0,98	PRR36	proline rich 36
0,98	CDK2	cyclin-dependent kinase 2
0,98	OR8H3	olfactory receptor, family 8, subfamily H, member 3
0,98	PKD1; MIR6511B2; MIR6511B1	polycystic kidney disease 1 (autosomal dominant); microRNA 6511b-2; microRNA 6511b-1
0,98	SGIP1	SH3-domain GRB2-like (endophilin) interacting protein 1
0,98	ADGRG3	adhesion G protein-coupled receptor G3
0,98	BCL2L2	BCL2-like 2
0,98	PPIAL4F; PPIAL4D	peptidylprolyl isomerase A (cyclophilin A)-like 4F; peptidylprolyl isomerase A (cyclophilin A)-like 4D
0,98	PANK1	pantothenate kinase 1
0,98	GRIN2C	glutamate receptor, ionotropic, N-methyl D-aspartate 2C
0,98	TMEM215	transmembrane protein 215
0,98	ZNF749	zinc finger protein 749
0,98	PFN4	profilin family, member 4
0,98	ZNF644	zinc finger protein 644
0,98	TAB1	TGF-beta activated kinase 1/MAP3K7 binding protein 1
0,98	LETMD1	LETM1 domain containing 1
0,98	ARGLU1	arginine and glutamate rich 1
0,98	CACNG2	calcium channel, voltage-dependent, gamma subunit 2
0,98	THRAP3	thyroid hormone receptor associated protein 3
0,98	NKAIN4	Na+/K+ transporting ATPase interacting 4
0,98	IFNL1	interferon, lambda 1
0,98	AGAP2	ArfGAP with GTPase domain, ankyrin repeat and PH domain 2
0,98	WDR18	WD repeat domain 18
0,98	EIF2D	eukaryotic translation initiation factor 2D
0,98	ATP1A1	ATPase, Na+/K+ transporting, alpha 1 polypeptide
0,98	ASB2	ankyrin repeat and SOCS box containing 2
0,98	C2orf69	chromosome 2 open reading frame 69
0,98	TPD52	tumor protein D52
0,98	LGALS4	lectin, galactoside-binding, soluble, 4
0,98	NDUFA10	NADH dehydrogenase (ubiquinone) 1 alpha subcomplex, 10, 42kDa
0,98	POU3F3	POU class 3 homeobox 3
0,98	RFX1	regulatory factor X, 1 (influences HLA class II expression)
0,98	ENY2	enhancer of yellow 2 homolog (Drosophila)
0,98	ZNF460	zinc finger protein 460

0,98	MTURN	maturin, neural progenitor differentiation regulator homolog (Xenopus)
0,98	EIF3J	eukaryotic translation initiation factor 3, subunit J
0,98	OR10X1	olfactory receptor, family 10, subfamily X, member 1 (gene/pseudogene)
0,98	HAGH	hydroxyacylglutathione hydrolase
0,98	ANO10	anoctamin 10
0,98	FAM210A	family with sequence similarity 210, member A
0,98	ICAM1	intercellular adhesion molecule 1
0,98	GPR108; MIR6791	G protein-coupled receptor 108; microRNA 6791
0,98	AHDC1	AT hook, DNA binding motif, containing 1
0,98	VGLL3	vestigial-like family member 3
0,98	GOLGA2	golgin A2
0,98	PGBD1	piggyBac transposable element derived 1
0,98	MMP24	matrix metallopeptidase 24 (membrane-inserted)
0,98	LRRC31	leucine rich repeat containing 31
0,98	MEMO1	mediator of cell motility 1
0,98	HBEGF	heparin-binding EGF-like growth factor
0,98	IRF6	interferon regulatory factor 6
0,98	LMO7	LIM domain 7
0,98	NASP	nuclear autoantigenic sperm protein (histone-binding)
0,98	PTGS2	prostaglandin-endoperoxide synthase 2 (prostaglandin G/H synthase and cyclooxygenase)
0,98	C19orf18	chromosome 19 open reading frame 18
0,98	REG1B	regenerating islet-derived 1 beta
0,98	TMEM42; MIR564	transmembrane protein 42; microRNA 564
0,98	PRAMEF25	PRAME family member 25
0,98	OR8B2	olfactory receptor, family 8, subfamily B, member 2
0,98	RPS10-NUDT3	RPS10-NUDT3 readthrough
0,98	KIAA1033	KIAA1033
0,98	WDR60	WD repeat domain 60
0,98	CDKN2AIPNL	CDKN2A interacting protein N-terminal like
0,98	MARVELD2	Transcript Identified by AceView, Entrez Gene ID(s) 153562
0,98	NUB1	negative regulator of ubiquitin-like proteins 1
0,98	CDC6	cell division cycle 6
0,98	PPP2R3B	protein phosphatase 2, regulatory subunit B, beta
0,98	PPP2R3B	protein phosphatase 2, regulatory subunit B, beta
0,98	GNS	glucosamine (N-acetyl)-6-sulfatase
0,98	NOC4L	nucleolar complex associated 4 homolog
0,98	KDELC1	KDEL (Lys-Asp-Glu-Leu) containing 1
0,98	KIAA0922	KIAA0922
0,98	GAS8	growth arrest-specific 8
0,98	HIST1H2AE	histone cluster 1, H2ae
0,98	CD163	CD163 molecule
0,98	RHOG	ras homolog family member G
0,98	SF1	splicing factor 1
0,98	RNF141	ring finger protein 141

0,98	USE1	unconventional SNARE in the ER 1 homolog (S. cerevisiae)
0,98	BTN3A2	butyrophilin, subfamily 3, member A2
0,98	SCAMP3	secretory carrier membrane protein 3
0,98	HERPUD1	homocysteine-inducible, endoplasmic reticulum stress-inducible, ubiquitin-like domain member 1
0,98	ZMAT1	zinc finger, matrin-type 1
0,98	PRCD	progressive rod-cone degeneration
0,98	ADARB1	adenosine deaminase, RNA-specific, B1
0,98	OR2T35	olfactory receptor, family 2, subfamily T, member 35
0,98	TMTC4	transmembrane and tetratricopeptide repeat containing 4
0,98	LILRB2	leukocyte immunoglobulin-like receptor, subfamily B (with TM and ITIM domains), member 2
0,98	G0S2	G0/G1 switch 2
0,98	HPS5	Hermansky-Pudlak syndrome 5
0,98	NMI	N-myc (and STAT) interactor
0,98	WWC1	WW and C2 domain containing 1
0,98	LINC00337	long intergenic non-protein coding RNA 337
0,98	ELAC2	elaC ribonuclease Z 2
0,98	PPP4C	protein phosphatase 4, catalytic subunit
0,98	APP	amyloid beta (A4) precursor protein
0,98	SMARCD1	SWI/SNF related, matrix associated, actin dependent regulator of chromatin, subfamily d, member 1
0,98	HIST2H3A; HIST2H3C	histone cluster 2, H3a; histone cluster 2, H3c
0,98	ZNF44	zinc finger protein 44
0,98	TOPORS-AS1; GVQW1	TOPORS antisense RNA 1; GVQW motif containing 1
0,98	CENPBD1	CENPB DNA-binding domain containing 1
0,98	ZNF697	zinc finger protein 697
0,98	PKM	pyruvate kinase, muscle
0,98	C16orf58	chromosome 16 open reading frame 58
0,98	ZMAT2	zinc finger, matrin-type 2
0,98	SLC17A3; HIST1H2APS2	solute carrier family 17 (organic anion transporter), member 3; histone cluster 1, H2a, pseudogene 2
0,98	ASXL2	additional sex combs like transcriptional regulator 2
0,98	WDR1	WD repeat domain 1
0,98	HLA-DPB1	major histocompatibility complex, class II, DP beta 1
0,98	C11orf63	chromosome 11 open reading frame 63
0,98	WNT4	wingless-type MMTV integration site family, member 4
0,98	SLFN13	schlafen family member 13
0,98	CARNS1	carnosine synthase 1
0,98	DBX1	developing brain homeobox 1
0,98	ZNF780A	zinc finger protein 780A
0,98	PRAMEF25	PRAME family member 25
0,98	SCLT1	sodium channel and clathrin linker 1
0,98	RCC2	regulator of chromosome condensation 2
0,98	TXNL4A	thioredoxin-like 4A

0,98	CDK2AP2	cyclin-dependent kinase 2 associated protein 2
0,98	TMSB10	thymosin beta 10
0,98	CES5A	carboxylesterase 5A
0,98	OTUD6B	Transcript Identified by AceView, Entrez Gene ID(s) 51633
0,98	NADK	NAD kinase
0,98	TMEM187	transmembrane protein 187
0,98	PRRT1	proline-rich transmembrane protein 1
0,98	NUP88	nucleoporin 88kDa
0,98	USO1	Transcript Identified by AceView, Entrez Gene ID(s) 8615
0,98	ADAR	adenosine deaminase, RNA-specific
0,98	HIST1H2BL	histone cluster 1, H2bl
0,98	MGAT5	mannosyl (alpha-1,6-)-glycoprotein beta-1,6-N-acetyl-glucosaminyltransferase
0,98	DNAH2	dynein, axonemal, heavy chain 2
0,98	MICAL3	Transcript Identified by AceView, Entrez Gene ID(s) 57553
0,98	TSR3	TSR3, 20S rRNA accumulation, homolog (S. cerevisiae)
0,98	HIST1H2AH	histone cluster 1, H2ah
0,98	NCKAP5L	NCK-associated protein 5-like
0,98	TECRL	trans-2,3-enoyl-CoA reductase-like
0,98	CPSF1; MIR939; MIR1234; MIR6849	cleavage and polyadenylation specific factor 1; microRNA 939; microRNA 1234; microRNA 6849
0,98	EPOR	erythropoietin receptor
0,98	FBXO24	F-box protein 24
0,98	CXXC1	CXXC finger protein 1
0,98	MOB1B	MOB kinase activator 1B
0,98	TMEM64	transmembrane protein 64
0,98	CERS4	ceramide synthase 4
0,98	ROCK1	Rho-associated, coiled-coil containing protein kinase 1
0,98	GLP2R	glucagon-like peptide 2 receptor
0,98	DLG1	discs, large homolog 1 (Drosophila)
0,98	CISD1	CDGSH iron sulfur domain 1
0,98	CACTIN	cactin, spliceosome C complex subunit
0,98	BTBD7	BTB (POZ) domain containing 7
0,98	C17orf74	chromosome 17 open reading frame 74
0,98	FAHD2A	Transcript Identified by AceView, Entrez Gene ID(s) 51011
0,98	LIPN	lipase, family member N
0,98	DECR2	2,4-dienoyl-CoA reductase 2, peroxisomal
0,98	OR5K1	olfactory receptor, family 5, subfamily K, member 1
0,98	CRNN	cornulin
0,98	TXNL1	thioredoxin-like 1
0,98	RSBN1	round spermatid basic protein 1
0,98	KIAA0391	KIAA0391
0,98	GDF11	growth differentiation factor 11

0,98	LAMTOR4	late endosomal/lysosomal adaptor, MAPK and MTOR activator 4
0,98	ATAD3A	ATPase family, AAA domain containing 3A
0,98	RPL35	ribosomal protein L35
0,98	TSPY1	testis specific protein, Y-linked 1
0,98	OR6K6	olfactory receptor, family 6, subfamily K, member 6
0,98	ALDH1B1	aldehyde dehydrogenase 1 family, member B1
0,98	LPO	lactoperoxidase
0,98	CHMP1A	charged multivesicular body protein 1A
0,98	PRPF38A	pre-mRNA processing factor 38A
0,98	RPS6	ribosomal protein S6
0,98	TACR3	tachykinin receptor 3
0,98	ZNF557	zinc finger protein 557
0,98	ITSN2	intersectin 2
0,98	EPYC	epiphycan
0,98	RPS6KC1	ribosomal protein S6 kinase, 52kDa, polypeptide 1
0,98	TMEM181	transmembrane protein 181
0,98	GABARAPL1	GABA(A) receptor-associated protein like 1
0,98	OTX2	orthodenticle homeobox 2
0,98	RSG1	REM2 and RAB-like small GTPase 1
0,98	USP33	ubiquitin specific peptidase 33
0,98	NAGPA	N-acetylglucosamine-1-phosphodiester alpha-N-acetylglucosaminidase
0,98	PVRIG	poliovirus receptor related immunoglobulin domain containing
0,98	DNAJB2	DnaJ (Hsp40) homolog, subfamily B, member 2
0,98	VSX2	visual system homeobox 2
0,98	CCDC127	coiled-coil domain containing 127
0,98	CAPS2	calcyphosine 2
0,98	CCDC58	coiled-coil domain containing 58
0,98	ADH1A	alcohol dehydrogenase 1A (class I), alpha polypeptide
0,98	CHRD	chordin
0,98	C3AR1	complement component 3a receptor 1
0,98	C19orf71	chromosome 19 open reading frame 71
0,98	FGF14; FGF14-IT1	fibroblast growth factor 14; FGF14 intronic transcript 1
0,98	PDYN	prodynorphin
0,98	WDR74	WD repeat domain 74
0,98	TOR3A	torsin family 3, member A
0,98	CA14	carbonic anhydrase XIV
0,98	NCOA3	nuclear receptor coactivator 3
0,98	LRRC15	leucine rich repeat containing 15
0,98	BID	BH3 interacting domain death agonist
0,98	AHCTF1	AT hook containing transcription factor 1
0,98	POLE2	polymerase (DNA directed), epsilon 2, accessory subunit
0,98	KRTAP4-9	keratin associated protein 4-9
0,98	PSORS1C2	psoriasis susceptibility 1 candidate 2
0,98	PPP1R36	protein phosphatase 1, regulatory subunit 36

0,98	C14orf1	chromosome 14 open reading frame 1
0,98	CFAP61	cilia and flagella associated protein 61
0,98	TRA2A	transformer 2 alpha homolog (Drosophila)
0,98	GMPPB	GDP-mannose pyrophosphorylase B
0,98	TIFA	TRAF-interacting protein with forkhead-associated domain
0,98	DTX4	deltex 4, E3 ubiquitin ligase
0,98	CHURC1-FNTB	CHURC1-FNTB readthrough
0,98	SULT1E1	sulfotransferase family 1E member 1
0,98	ZNF831	zinc finger protein 831
0,98	CDC45	cell division cycle 45
0,98	SYK	Memczak2013 ANTISENSE, CDS, coding, INTERNAL best transcript NM_001174167
0,98	CTNND1	catenin (cadherin-associated protein), delta 1
0,98	DYNC1LI1	dynein, cytoplasmic 1, light intermediate chain 1
0,98	DMBX1	diencephalon/mesencephalon homeobox 1
0,98	NAA38	N(alpha)-acetyltransferase 38, NatC auxiliary subunit
0,98	PLA2G15	phospholipase A2, group XV
0,98	RILPL2	Rab interacting lysosomal protein-like 2
0,98	NCL	nucleolin
0,98	TSLP	thymic stromal lymphopoietin
0,98	C9orf114	chromosome 9 open reading frame 114
0,98	LOC102724238; LOC554249	uncharacterized LOC102724238; uncharacterized LOC554249
0,98	TAF7	TAF7 RNA polymerase II, TATA box binding protein (TBP)-associated factor, 55kDa
0,98	TCF24	transcription factor 24
0,98	ZNF569	zinc finger protein 569
0,98	PELO; ITGA1	pelota homolog (Drosophila); integrin alpha 1
0,98	HNRNPUL2-BSCL2	HNRNPUL2-BSCL2 readthrough (NMD candidate)
0,98	CELF4	CUGBP, Elav-like family member 4
0,98	CAPN11	calpain 11
0,98	LOC339803; AC016747.3	uncharacterized LOC339803; Transcript Identified by AceView, Entrez Gene ID(s) 339803; novel transcript
0,98	RSPH4A	radial spoke head 4 homolog A (Chlamydomonas)
0,98	RDH12	retinol dehydrogenase 12 (all-trans/9-cis/11-cis)
0,98	ZNF385A	zinc finger protein 385A
0,98	ANAPC5	anaphase promoting complex subunit 5
0,98	HOMER3	homer scaffolding protein 3
0,98	KIF23	kinesin family member 23
0,98	TMX2-CTNND1	TMX2-CTNND1 readthrough (NMD candidate)
0,98	UBE2I	ubiquitin conjugating enzyme E2I
0,98	C5orf52	chromosome 5 open reading frame 52
0,98	MRPL47	mitochondrial ribosomal protein L47
0,98	SLC16A3; MIR6787	solute carrier family 16 (monocarboxylate transporter), member 3; microRNA 6787
0,98	LRRC20	Transcript Identified by AceView, Entrez Gene ID(s) 55222
0,98	KLHL24	kelch-like family member 24

0,98	ZFHX2	zinc finger homeobox 2
0,98	SNX9	sorting nexin 9
0,98	RPL15	ribosomal protein L15
0,98	EIF4EBP2	eukaryotic translation initiation factor 4E binding protein 2
0,98	CHM	choroideremia (Rab escort protein 1)
0,98	PSTK	phosphoseryl-tRNA kinase
0,98	UBXN10	UBX domain protein 10
0,98	ST6GALNAC6	ST6 (alpha-N-acetyl-neuraminyl-2,3-beta-galactosyl-1,3)-N-acetylgalactosaminide alpha-2,6-sialyltransferase 6
0,98	SBDS	Shwachman-Bodian-Diamond syndrome
0,98	RPL8; MIR6850	ribosomal protein L8; microRNA 6850
0,98	RHOH	ras homolog family member H
0,98	ARMC8	armadillo repeat containing 8
0,98	PRCP	prolylcarboxypeptidase
0,98	DDAH2	dimethylarginine dimethylaminohydrolase 2
0,98	FAM19A4	family with sequence similarity 19 (chemokine (C-C motif)-like), member A4
0,98	TK1	thymidine kinase 1, soluble
0,98	NIN	ninein (GSK3B interacting protein)
0,98	STK3	serine/threonine kinase 3
0,98	TMEM178B	transmembrane protein 178B
0,98	MSH2	mutS homolog 2
0,98	EPHA7	EPH receptor A7
0,98	RFC4	replication factor C subunit 4
0,98	TMBIM6	transmembrane BAX inhibitor motif containing 6
0,98	FZD9	frizzled class receptor 9
0,98	FMO3	flavin containing monooxygenase 3
0,98	MDM2	MDM2 proto-oncogene, E3 ubiquitin protein ligase
0,98	POLR3E	polymerase (RNA) III (DNA directed) polypeptide E (80kD)
0,98	HOOK2	hook microtubule-tethering protein 2
0,98	TNFRSF13B	tumor necrosis factor receptor superfamily, member 13B
0,98	CASC1	cancer susceptibility candidate 1
0,98	MRPL41	mitochondrial ribosomal protein L41
0,98	IGFBP6	insulin like growth factor binding protein 6
0,98	IFIT1	interferon-induced protein with tetratricopeptide repeats 1
0,98	TAB2	TGF-beta activated kinase 1/MAP3K7 binding protein 2
0,98	UST	uronyl-2-sulfotransferase
0,98	NSMAF	neutral sphingomyelinase activation associated factor
0,98	PSMA7	proteasome subunit alpha 7
0,98	TMEM126B	transmembrane protein 126B
0,98	USP11	ubiquitin specific peptidase 11
0,98	OR14A16	olfactory receptor, family 14, subfamily A, member 16
0,98	SCGB1D4	secretoglobin, family 1D, member 4

0,98	GFPT2	glutamine-fructose-6-phosphate transaminase 2
0,98	CASK	calcium/calmodulin-dependent serine protein kinase (MAGUK family)
0,98	RPL9	ribosomal protein L9
0,98	NPAP1	nuclear pore associated protein 1
0,98	LOC389895	chromosome 16 open reading frame 72-like
0,98	TMEM97	transmembrane protein 97
0,98	HTATIP2	HIV-1 Tat interactive protein 2
0,98	CCM2L	cerebral cavernous malformation 2-like
0,98	PRAMEF20	PRAME family member 20
0,98	ATP6V1G2	ATPase, H+ transporting, lysosomal 13kDa, V1 subunit G2
0,98	DLX6	distal-less homeobox 6
0,98	TIMM17B	translocase of inner mitochondrial membrane 17 homolog B (yeast)
0,98	DBH	dopamine beta-hydroxylase (dopamine beta-monooxygenase)
0,98	NXPE4	neurexophilin and PC-esterase domain family, member 4
0,98	HYPM	huntingtin interacting protein M
0,98	MFN1	mitofusin 1
0,98	GET4	golgi to ER traffic protein 4
0,98	SENP3	SUMO1/sentrin/SMT3 specific peptidase 3
0,98	CDKAL1	Jeck2013 ALT_ACCEPTOR, ALT_DONOR, coding, INTERNAL, intronic best transcript NM_017774
0,98	ALG14	ALG14, UDP-N-acetylglucosaminyltransferase subunit
0,98	OR1G1	olfactory receptor, family 1, subfamily G, member 1
0,98	RPL36A	ribosomal protein L36a
0,98	ADAM7	ADAM metallopeptidase domain 7
0,98	LRPPRC	leucine-rich pentatricopeptide repeat containing
0,98	ZMYM4	zinc finger, MYM-type 4
0,98	NKIRAS2	NFKB inhibitor interacting Ras-like 2
0,98	PCNXL2	pecanex-like 2 (Drosophila)
0,98	LRRTM4	leucine rich repeat transmembrane neuronal 4
0,98	FER1L6-AS1	FER1L6 antisense RNA 1
0,98	TNFRSF11A	Transcript Identified by AceView, Entrez Gene ID(s) 8792
0,98	MLC1	megalencephalic leukoencephalopathy with subcortical cysts 1
0,98	R3HDM1	Transcript Identified by AceView, Entrez Gene ID(s) 23518
0,98	ARHGEF18	Rho/Rac guanine nucleotide exchange factor 18
0,98	NGF	nerve growth factor (beta polypeptide)
0,98	HS2ST1	heparan sulfate 2-O-sulfotransferase 1
0,98	EPHX2	epoxide hydrolase 2, cytoplasmic
0,98	ZC3H13	Memczak2013 ANTISENSE, CDS, coding, INTERNAL best transcript NM_015070
0,98	GPR55	G protein-coupled receptor 55
0,98	C2orf50	chromosome 2 open reading frame 50

0,98	RGL1	ral guanine nucleotide dissociation stimulator-like 1
0,98	PCBP2; PCBP2-OT1	poly(rC) binding protein 2; PCBP2 overlapping transcript 1
0,98	SF3B6	splicing factor 3b subunit 6
0,98	C11orf54	chromosome 11 open reading frame 54
0,98	ZNF350	zinc finger protein 350
0,98	FAM46C	family with sequence similarity 46, member C
0,98	HBA1	hemoglobin, alpha 1
0,98	IRX6	iroquois homeobox 6
0,98	ZFAT	zinc finger and AT hook domain containing
0,98	ELOVL3	ELOVL fatty acid elongase 3
0,98	PTPRD	protein tyrosine phosphatase, receptor type, D
0,98	C4orf47	chromosome 4 open reading frame 47
0,98	MUC13	mucin 13, cell surface associated
0,98	ASPN	asporin
0,98	APLF	aprataxin and PNKP like factor
0,98	CLPS	colipase, pancreatic
0,98	C10orf67	chromosome 10 open reading frame 67
0,98	ARAP1	ArfGAP with RhoGAP domain, ankyrin repeat and PH domain 1
0,98	CRTC3	CREB regulated transcription coactivator 3
0,98	OR4K1	olfactory receptor, family 4, subfamily K, member 1
0,98	LYRM9	LYR motif containing 9
0,98	ACAD9	acyl-CoA dehydrogenase family, member 9
0,98	TEAD4	TEA domain family member 4
0,98	PREB	prolactin regulatory element binding
0,98	CADM1	cell adhesion molecule 1
0,98	RNF126	ring finger protein 126
0,98	C9orf135	chromosome 9 open reading frame 135
0,98	MCMBP	minichromosome maintenance complex binding protein
0,98	ZEB2	zinc finger E-box binding homeobox 2
0,98	CIB3	calcium and integrin binding family member 3
0,98	IGSF8	immunoglobulin superfamily, member 8
0,98	SLC17A2	solute carrier family 17, member 2
0,98	PREPL	prolyl endopeptidase-like
0,98	TTK	TTK protein kinase
0,98	CSF2RB	colony stimulating factor 2 receptor, beta, low-affinity (granulocyte-macrophage)
0,98	TMEM88	transmembrane protein 88
0,98	CFAP206	cilia and flagella associated protein 206
0,98	CFAP126	cilia and flagella associated protein 126
0,98	CWC25	CWC25 spliceosome-associated protein homolog
0,97	MRPL20	mitochondrial ribosomal protein L20
0,97	TMEM183B	transmembrane protein 183B
0,97	REEP3	receptor accessory protein 3
0,97	EXOSC9	exosome component 9
0,97	KRTAP5-7	keratin associated protein 5-7
0,97	BASP1	brain abundant, membrane attached signal protein 1
0,97	RGS21	regulator of G-protein signaling 21

0,97	CD109	CD109 molecule
0,97	JAG1	jagged 1
0,97	TSPYL6	TSPY-like 6
0,97	SPATA20	spermatogenesis associated 20
0,97	NME1; NME1-NME2; NME2	NME/NM23 nucleoside diphosphate kinase 1; NME1-NME2 readthrough; NME/NM23 nucleoside diphosphate kinase 2
0,97	LHX5	LIM homeobox 5
0,97	TUBB6	tubulin, beta 6 class V
0,97	FGF11	fibroblast growth factor 11
0,97	LDB3	LIM domain binding 3
0,97	MKX	mohawk homeobox
0,97	COPS7B	COP9 signalosome subunit 7B
0,97	RAB6A	RAB6A, member RAS oncogene family
0,97	LRRC23	leucine rich repeat containing 23
0,97	NRROS	negative regulator of reactive oxygen species
0,97	BLACAT1; LEMD1	bladder cancer associated transcript 1 (non-protein coding); LEM domain containing 1
0,97	SCNN1B	sodium channel, non voltage gated 1 beta subunit
0,97	C1QA	complement component 1, q subcomponent, A chain
0,97	ZNF320	zinc finger protein 320
0,97	POLR2D	polymerase (RNA) II (DNA directed) polypeptide D
0,97	EXOC5	exocyst complex component 5
0,97	COX7A2L	cytochrome c oxidase subunit VIIa polypeptide 2 like
0,97	PLEKHM1; MIR4315-1	pleckstrin homology domain containing, family M (with RUN domain) member 1; microRNA 4315-1
0,97	KAZN	kazrin, periplakin interacting protein
0,97	MAP3K9	mitogen-activated protein kinase kinase kinase 9
0,97	STAMBP	STAM binding protein
0,97	FAM218A	family with sequence similarity 218, member A
0,97	RARS2	arginyl-tRNA synthetase 2, mitochondrial
0,97	SUCNR1	succinate receptor 1
0,97	MLXIP	MLX interacting protein
0,97	OR2A14; OR2A2	olfactory receptor, family 2, subfamily A, member 14; olfactory receptor, family 2, subfamily A, member 2
0,97	LAYN	layilin
0,97	NUBP1	nucleotide binding protein 1
0,97	RIC8A; MIR6743	RIC8 guanine nucleotide exchange factor A; microRNA 6743
0,97	AGAP3	ArfGAP with GTPase domain, ankyrin repeat and PH domain 3
0,97	CYYR1	cysteine/tyrosine-rich 1
0,97	IFNA13	interferon, alpha 13
0,97	TRIM16L	tripartite motif containing 16-like
0,97	GMPPA	GDP-mannose pyrophosphorylase A
0,97	KRTAP19-8	keratin associated protein 19-8
0,97	IYD	iodotyrosine deiodinase
0,97	BIRC8	baculoviral IAP repeat containing 8
0,97	EFR3B	EFR3 homolog B

0,97	MRPS7	mitochondrial ribosomal protein S7
0,97	C10orf91	chromosome 10 open reading frame 91
0,97	FARP2	FERM, ARH/RhoGEF and pleckstrin domain protein 2
0,97	FAM134C	family with sequence similarity 134, member C
0,97	AMIGO1	adhesion molecule with Ig-like domain 1
0,97	CSF2RA	colony stimulating factor 2 receptor, alpha, low-affinity (granulocyte-macrophage)
0,97	SLU7	SLU7 homolog, splicing factor
0,97	GPATCH8	G-patch domain containing 8
0,97	UBE2L6	Transcript Identified by AceView, Entrez Gene ID(s) 9246
0,97	NAA40	N(alpha)-acetyltransferase 40, NatD catalytic subunit
0,97	CLSPN	claspin
0,97	BORCS6	BLOC-1 related complex subunit 6
0,97	PAPOLB	poly(A) polymerase beta
0,97	ARPC1A	actin related protein 2/3 complex subunit 1A
0,97	LINC01565	long intergenic non-protein coding RNA 1565
0,97	OCIAD2	OCIA domain containing 2
0,97	HNRNPK; MIR7-1	heterogeneous nuclear ribonucleoprotein K; microRNA 7-1
0,97	B9D1	B9 protein domain 1
0,97	C16orf45	chromosome 16 open reading frame 45
0,97	C17orf97	chromosome 17 open reading frame 97
0,97	GPR174	G protein-coupled receptor 174
0,97	PBX2	pre-B-cell leukemia homeobox 2
0,97	C8orf59	chromosome 8 open reading frame 59
0,97	WDR5	WD repeat domain 5
0,97	SNAPC4	small nuclear RNA activating complex polypeptide 4
0,97	UNC119B	Memczak2013 ANTISENSE, CDS, coding, INTERNAL, UTR3 best transcript NM_001080533
0,97	AEBP2	AE binding protein 2
0,97	SEPSECS	Sep (O-phosphoserine) tRNA:Sec (selenocysteine) tRNA synthase
0,97	SLC22A18	solute carrier family 22, member 18
0,97	PHYH	phytanoyl-CoA 2-hydroxylase
0,97	LOC101928327; AC007557.1	uncharacterized LOC101928327; novel transcript
0,97	YIPF6	Yip1 domain family member 6
0,97	ABHD10	abhydrolase domain containing 10
0,97	TGIF1	TGFB-induced factor homeobox 1
0,97	RHBDL3	rhomboid, veinlet-like 3 (Drosophila)
0,97	USP2	ubiquitin specific peptidase 2
0,97	C18orf65	chromosome 18 open reading frame 65
0,97	CPNE4	copine IV
0,97	SSRP1	structure specific recognition protein 1
0,97	BBIP1	BBSome interacting protein 1
0,97	DDX10	DEAD (Asp-Glu-Ala-Asp) box polypeptide 10
0,97	ALOX12	arachidonate 12-lipoxygenase
0,97	EIF3H	eukaryotic translation initiation factor 3, subunit H

0,97	RBM26	RNA binding motif protein 26
0,97	HIST1H3E	histone cluster 1, H3e
0,97	XPNPEP1	X-prolyl aminopeptidase (aminopeptidase P) 1, soluble
0,97	ADGRB3	adhesion G protein-coupled receptor B3
0,97	GABRB2	gamma-aminobutyric acid (GABA) A receptor, beta 2
0,97	LPL	lipoprotein lipase
0,97	APBB2	amyloid beta (A4) precursor protein-binding, family B, member 2
0,97	LRRC37B	leucine rich repeat containing 37B
0,97	CHCHD10	coiled-coil-helix-coiled-coil-helix domain containing 10
0,97	HYLS1	hydrolethalus syndrome 1
0,97	BLZF1	basic leucine zipper nuclear factor 1
0,97	DR1	down-regulator of transcription 1
0,97	TOPORS	topoisomerase I binding, arginine/serine-rich, E3 ubiquitin protein ligase
0,97	HOXA10; HOXA9; MIR196B	homeobox A10; homeobox A9; microRNA 196b
0,97	CDC42EP3	CDC42 effector protein (Rho GTPase binding) 3
0,97	SLC28A2	solute carrier family 28 (concentrative nucleoside transporter), member 2
0,97	LOC101927668; AC005062.2; MACC1	uncharacterized LOC101927668; Transcript Identified by AceView, Entrez Gene ID(s) 346389; novel transcript, sense overlapping to MACC1
0,97	ATP6V1C2	ATPase, H+ transporting, lysosomal 42kDa, V1 subunit C2
0,97	TMEM38A	Transcript Identified by AceView, Entrez Gene ID(s) 79041
0,97	RNFT1	ring finger protein, transmembrane 1
0,97	TUBAL3	tubulin, alpha-like 3
0,97	FCAMR	Fc receptor, IgA, IgM, high affinity
0,97	OR4F3	olfactory receptor, family 4, subfamily F, member 3
0,97	TAL2	T-cell acute lymphocytic leukemia 2
0,97	SRL	sarcalumenin
0,97	PRAMEF25	PRAME family member 25
0,97	PRAMEF25	PRAME family member 25
0,97	PPP1R12A	protein phosphatase 1, regulatory subunit 12A
0,97	RAD51B	Jeck2013 ALT_ACCEPTOR, ALT_DONOR, coding, INTERNAL, intronic best transcript NM_133509
0,97	OBP2B	odorant binding protein 2B
0,97	HGSNAT	heparan-alpha-glucosaminide N-acetyltransferase
0,97	PPIL3	peptidylprolyl isomerase (cyclophilin)-like 3
0,97	S100A12	S100 calcium binding protein A12
0,97	DEFB1	defensin, beta 1
0,97	SPACA5	sperm acrosome associated 5
0,97	SNCB	synuclein beta
0,97	KRTAP29-1	keratin associated protein 29-1
0,97	IL17C	interleukin 17C

0,97	UBE2D4	ubiquitin-conjugating enzyme E2D 4 (putative)
0,97	XAGE2	X antigen family, member 2
0,97	HHATL	hedgehog acyltransferase-like
0,97	NFIC	nuclear factor I/C (CCAAT-binding transcription factor)
0,97	BTBD8	BTB (POZ) domain containing 8
0,97	UXT	ubiquitously expressed prefoldin like chaperone
0,97	CHPT1	choline phosphotransferase 1
0,97	ERVV-1	endogenous retrovirus group V, member 1
0,97	RAD54B; FSBP	RAD54 homolog B (S. cerevisiae); fibrinogen silencer binding protein
0,97	NFIL3	nuclear factor, interleukin 3 regulated
0,97	NPIPB11	nuclear pore complex interacting protein family, member B11
0,97	BPTF	bromodomain PHD finger transcription factor
0,97	BRWD1	bromodomain and WD repeat domain containing 1
0,97	ANXA11	annexin A11
0,97	TMEM255B	transmembrane protein 255B
0,97	TTC6	tetratricopeptide repeat domain 6
0,97	ZDHHC3	zinc finger, DHHC-type containing 3
0,97	GLG1	golgi glycoprotein 1
0,97	TIMM8A	translocase of inner mitochondrial membrane 8 homolog A (yeast)
0,97	VAMP7	Homo sapiens vesicle-associated membrane protein 7 (VAMP7), transcript variant 2, mRNA.; Homo sapiens vesicle-associated membrane protein 7 (VAMP7), transcript variant 3, mRNA.; Homo sapiens vesicle-associated membrane protein 7 (VAMP7), transcript variant 1, mRNA.; Homo sapiens vesicle-associated membrane protein 7 (VAMP7), transcript variant 4, non-coding RNA.; Homo sapiens vesicle-associated membrane protein 7 (VAMP7), transcript variant 5, non-coding RNA.; vesicle-associated membrane protein 7 [Source:HGNC Symbol;Acc:HGNC:11486]; Homo sapiens vesicle-associated membrane protein 7, mRNA (cDNA clone MGC:64832 IMAGE:6503665), complete cds.
0,97	MGAT5B	mannosyl (alpha-1,6-)-glycoprotein beta-1,6-N-acetyl-glucosaminyltransferase, isozyme B
0,97	PIGP	phosphatidylinositol glycan anchor biosynthesis class P
0,97	SEC14L3	SEC14-like lipid binding 3
0,97	NFYA	nuclear transcription factor Y subunit alpha
0,97	NTS	neurotensin
0,97	RPS25	ribosomal protein S25
0,97	CD200R1	CD200 receptor 1
0,97	ALDOA	aldolase A, fructose-bisphosphate
0,97	VAC14	Vac14 homolog (S. cerevisiae)
0,97	BSPH1	binder of sperm protein homolog 1
0,97	ACAD8	acyl-CoA dehydrogenase family, member 8

0,97	PUF60	poly-U binding splicing factor 60KDa
0,97	LAS1L	LAS1-like, ribosome biogenesis factor
0,97	BAMBI	BMP and activin membrane-bound inhibitor
0,97	PYROXD1	pyridine nucleotide-disulphide oxidoreductase domain 1
0,97	OR6C6	olfactory receptor, family 6, subfamily C, member 6
0,97	RSPH14	radial spoke head 14 homolog (Chlamydomonas)
0,97	ZNF268	zinc finger protein 268
0,97	GREM2	gremlin 2, DAN family BMP antagonist
0,97	KCTD17	potassium channel tetramerization domain containing 17
0,97	DOCK10	dedicator of cytokinesis 10
0,97	ABCC6	ATP binding cassette subfamily C member 6
0,97	RANBP6	RAN binding protein 6
0,97	SEMA3A	Transcript Identified by AceView, Entrez Gene ID(s) 10371
0,97	TMEM44	transmembrane protein 44
0,97	RAD21L1	RAD21 cohesin complex component like 1
0,97	RNMT	RNA (guanine-7-) methyltransferase
0,97	EVA1B	eva-1 homolog B (C. elegans)
0,97	PNRC2	proline-rich nuclear receptor coactivator 2
0,97	ZNF35	zinc finger protein 35
0,97	MAGEB1; MAGEB4	MAGE family member B1; MAGE family member B4
0,97	DET1	de-etiolated homolog 1 (Arabidopsis)
0,97	TWISTNB	TWIST neighbor
0,97	PTER	phosphotriesterase related
0,97	TKFC	triokinase/FMN cyclase
0,97	POLR2B	polymerase (RNA) II (DNA directed) polypeptide B, 140kDa
0,97	MRPS18C	mitochondrial ribosomal protein S18C
0,97	AKR1C8P	aldo-keto reductase family 1, member C8, pseudogene
0,97	RIC8B	RIC8 guanine nucleotide exchange factor B
0,97	OXCT2P1	3-oxoacid CoA-transferase 2 pseudogene 1
0,97	FERMT2	fermitin family member 2
0,97	LRRC3C	leucine rich repeat containing 3C
0,97	TMOD3	tropomodulin 3 (ubiquitous)
0,97	YWHAB	tyrosine 3-monooxygenase/tryptophan 5-monooxygenase activation protein, beta
0,97	ADAMTS15	ADAM metallopeptidase with thrombospondin type 1 motif 15
0,97	POLR3F	polymerase (RNA) III (DNA directed) polypeptide F, 39 kDa
0,97	FOPNL	FGFR1OP N-terminal like
0,97	GNE	glucosamine (UDP-N-acetyl)-2-epimerase/N-acetylmannosamine kinase
0,97	COX7B	cytochrome c oxidase subunit VIIb
0,97	SPATA31C2	SPATA31 subfamily C, member 2
0,97	TMED2	transmembrane p24 trafficking protein 2

0,97	OTUD3	OTU deubiquitinase 3
0,97	TMEM141	transmembrane protein 141
0,97	ZNF780A	zinc finger protein 780A
0,97	RUBCN; MIR922	RUN domain and cysteine-rich domain containing, Beclin 1-interacting protein; microRNA 922
0,97	TSPAN31	tetraspanin 31
0,97	MEP1A	meprin A, alpha (PABA peptide hydrolase)
0,97	ZNF669	zinc finger protein 669
0,97	TADA1	transcriptional adaptor 1
0,97	ZNF221	zinc finger protein 221
0,97	SEBOX	SEBOX homeobox
0,97	NDUFB3	NADH dehydrogenase (ubiquinone) 1 beta subcomplex, 3, 12kDa
0,97	PAQR4	progestin and adipoQ receptor family member IV
0,97	FOXF1	forkhead box F1
0,97	ACSM4	acyl-CoA synthetase medium-chain family member 4
0,97	RAF1	Raf-1 proto-oncogene, serine/threonine kinase
0,97	KIAA0430	Transcript Identified by AceView, Entrez Gene ID(s) 9665
0,97	TSPYL5	TSPY-like 5
0,97	KIRREL3	Jeck2013 ALT_ACCEPTOR, ALT_DONOR, coding, INTERNAL, intronic best transcript NM_032531
0,97	TRPV2	transient receptor potential cation channel, subfamily V, member 2
0,97	TUBGCP5	tubulin, gamma complex associated protein 5
0,97	UBR7	ubiquitin protein ligase E3 component n-recognin 7 (putative)
0,97	LOC100131107	putative UPF0607 protein ENSP00000383783
0,97	ZNF833P	zinc finger protein 833, pseudogene
0,97	MTX1	metaxin 1
0,97	HBD	hemoglobin, delta
0,97	NT5DC3	5-nucleotidase domain containing 3
0,97	RQCD1	RCD1 required for cell differentiation1 homolog (S. pombe)
0,97	UMPS	uridine monophosphate synthetase
0,97	MAMDC2	MAM domain containing 2
0,97	PCYOX1	prenylcysteine oxidase 1
0,97	EEF2KMT	eukaryotic elongation factor 2 lysine methyltransferase
0,97	NPR3	natriuretic peptide receptor 3
0,97	ZNF33A	zinc finger protein 33A
0,97	CHRNA10; NUP98	cholinergic receptor, nicotinic alpha 10; nucleoporin 98kDa
0,97	HEMGN	hemogen
0,97	ADGRL1	adhesion G protein-coupled receptor L1
0,97	SPOPL	speckle-type POZ protein-like
0,97	DTX2	deltex 2, E3 ubiquitin ligase
0,97	ADD2	adducin 2 (beta)
0,97	HEYL	hes-related family bHLH transcription factor with YRPW motif-like

0,97	PRKAB1	protein kinase, AMP-activated, beta 1 non-catalytic subunit
0,97	LRRC71	leucine rich repeat containing 71
0,97	MYO6	myosin VI
0,97	TMPRSS15	transmembrane protease, serine 15
0,97	SPN	Memczak2013 ANTISENSE, CDS, coding, INTERNAL best transcript NM_003123
0,97	EIF2S1	eukaryotic translation initiation factor 2, subunit 1 alpha, 35kDa
0,97	CERS6	ceramide synthase 6
0,97	EIF3C; EIF3CL	eukaryotic translation initiation factor 3, subunit C; eukaryotic translation initiation factor 3, subunit C-like
0,97	RPL7A	ribosomal protein L7a
0,97	SH3BP1; PDXP	SH3-domain binding protein 1; pyridoxal (pyridoxine, vitamin B6) phosphatase
0,97	METTL13	methyltransferase like 13
0,97	ATP13A4	ATPase type 13A4
0,97	LRRC38	leucine rich repeat containing 38
0,97	ACCSL	1-aminocyclopropane-1-carboxylate synthase (inactive)-like
0,97	MLXIP	MLX interacting protein
0,97	APOA5	apolipoprotein A-V
0,97	SLC35B4	solute carrier family 35 (UDP-xylose/UDP-N-acetylglucosamine transporter), member B4
0,97	PAIP2	poly(A) binding protein interacting protein 2
0,97	ARPC5	actin related protein 2/3 complex subunit 5
0,97	ADNP	activity-dependent neuroprotector homeobox
0,97	USMG5; MIR1307	up-regulated during skeletal muscle growth 5 homolog (mouse); microRNA 1307
0,97	FRMPD3	FERM and PDZ domain containing 3
0,97	UNC93B1	unc-93 homolog B1 (C. elegans)
0,97	SUFU	Transcript Identified by AceView, Entrez Gene ID(s) 51684
0,97	P2RX3	purinergic receptor P2X, ligand gated ion channel, 3
0,97	ADCK5	aarF domain containing kinase 5
0,97	PRMT1	protein arginine methyltransferase 1
0,97	C14orf2	chromosome 14 open reading frame 2
0,97	C10orf105	chromosome 10 open reading frame 105
0,97	RPL34	ribosomal protein L34
0,97	UHMK1	U2AF homology motif (UHM) kinase 1
0,97	OR6M1	olfactory receptor, family 6, subfamily M, member 1
0,97	ORAI2	ORAI calcium release-activated calcium modulator 2
0,97	ZBED2	zinc finger, BED-type containing 2
0,97	VWDE	von Willebrand factor D and EGF domains
0,97	EHHADH	enoyl-CoA, hydratase/3-hydroxyacyl CoA dehydrogenase
0,97	KCP	kielin/chordin-like protein
0,97	CEPT1	choline/ethanolamine phosphotransferase 1
0,97	PCNX	pecanex homolog (Drosophila)

0,97	MGME1	mitochondrial genome maintenance exonuclease 1
0,97	NFKBID	nuclear factor of kappa light polypeptide gene enhancer in B-cells inhibitor, delta
0,97	PRRC2A	proline-rich coiled-coil 2A
0,97	PIGBOS1; RAB27A	PIGB opposite strand 1; RAB27A, member RAS oncogene family
0,97	CAMK2G	calcium/calmodulin-dependent protein kinase II gamma
0,97	LCE1B	late cornified envelope 1B
0,97	TUBGCP3	tubulin, gamma complex associated protein 3
0,97	PABPN1	poly(A) binding protein, nuclear 1
0,97	GNB1	guanine nucleotide binding protein (G protein), beta polypeptide 1
0,97	CPLX2	complexin 2
0,97	ELP4	elongator acetyltransferase complex subunit 4
0,97	ZCCHC16	zinc finger, CCHC domain containing 16
0,97	MRPL49	mitochondrial ribosomal protein L49
0,97	CCDC77	coiled-coil domain containing 77
0,97	ATP6V0A2	ATPase, H+ transporting, lysosomal V0 subunit a2
0,97	NARF	nuclear prelamin A recognition factor
0,97	U2AF2	Memczak2013 ALT_ACCEPTOR, ALT_DONOR, coding, INTERNAL, intronic best transcript NM_001012478
0,97	FAM117B	family with sequence similarity 117, member B
0,97	FKBP5	FK506 binding protein 5
0,97	TSPAN4	tetraspanin 4
0,97	KRTAP19-6	keratin associated protein 19-6
0,97	STK16	serine/threonine kinase 16
0,97	KLB	klotho beta
0,97	STK19	serine/threonine kinase 19
0,97	ROMO1	reactive oxygen species modulator 1
0,97	METRNL	meteorin, glial cell differentiation regulator-like
0,97	PSG6	pregnancy specific beta-1-glycoprotein 6
0,97	CHAF1A	chromatin assembly factor 1, subunit A (p150)
0,97	TRA2B	transformer 2 beta homolog (Drosophila)
0,97	ZBTB8OS	zinc finger and BTB domain containing 8 opposite strand
0,97	UBL5	ubiquitin-like 5
0,97	PDE7B	phosphodiesterase 7B
0,97	NREP	neuronal regeneration related protein
0,97	GUK1	guanylate kinase 1
0,97	DSCAML1	Down syndrome cell adhesion molecule like 1
0,97	QRFP	pyroglutamylated RFamide peptide
0,97	MTMR6	Transcript Identified by AceView, Entrez Gene ID(s) 9107
0,97	SLC9A7	solute carrier family 9, subfamily A (NHE7, cation proton antiporter 7), member 7
0,97	DPP8	dipeptidyl-peptidase 8
0,97	CASP1	caspase 1
0,97	CTDSP1	CTD small phosphatase 1

0,97	PPFIBP1	PTPRF interacting protein, binding protein 1 (liprin beta 1)
0,97	CELA1	chymotrypsin-like elastase family, member 1
0,97	TMEM8A	transmembrane protein 8A
0,97	SV2A	synaptic vesicle glycoprotein 2A
0,97	ACLY	ATP citrate lyase
0,97	MYH9	Memczak2013 ANTISENSE, CDS, coding, INTERNAL best transcript NM_002473
0,97	RER1	retention in endoplasmic reticulum sorting receptor 1
0,97	TMEM30B	transmembrane protein 30B
0,97	ZKSCAN7	zinc finger with KRAB and SCAN domains 7
0,97	CBS	cystathionine-beta-synthase
0,97	AP1AR	adaptor-related protein complex 1 associated regulatory protein
0,97	IPO8	importin 8
0,97	CASP3	caspase 3
0,97	ANKRD13C	ankyrin repeat domain 13C
0,97	PVALB	parvalbumin
0,97	GPR87	G protein-coupled receptor 87
0,97	TMOD4	tropomodulin 4 (muscle)
0,97	VPS37B	vacuolar protein sorting 37 homolog B (S. cerevisiae)
0,97	REP15	RAB15 effector protein
0,97	DNAJB11	DnaJ (Hsp40) homolog, subfamily B, member 11
0,97	CTU2	cytosolic thiouridylase subunit 2 homolog (S. pombe)
0,97	DIO2	deiodinase, iodothyronine, type II
0,97	SYNE4	spectrin repeat containing, nuclear envelope family member 4
0,97	FUT8	fucosyltransferase 8 (alpha (1,6) fucosyltransferase)
0,97	KRT32	keratin 32, type I
0,97	DEFB4A	defensin, beta 4A
0,97	MBNL3	muscleblind-like splicing regulator 3
0,97	DPPA5	developmental pluripotency associated 5
0,97	ACAP2	ArfGAP with coiled-coil, ankyrin repeat and PH domains 2
0,97	FMN1	formin 1
0,97	OR2F1	olfactory receptor, family 2, subfamily F, member 1 (gene/pseudogene)
0,97	BACH1	BTB and CNC homology 1, basic leucine zipper transcription factor 1
0,97	FLOT2	Memczak2013 ANTISENSE, CDS, coding, INTERNAL, intronic best transcript NM_004475
0,97	GOLGA3	golgin A3
0,97	DPRX	divergent-paired related homeobox
0,97	MEI4	meiotic double-stranded break formation protein 4
0,97	TRMO	tRNA methyltransferase O
0,97	PCDH10	protocadherin 10
0,97	ATP6V1G1	ATPase, H+ transporting, lysosomal 13kDa, V1 subunit G1
0,97	PRMT8	protein arginine methyltransferase 8

0,97	SEMA6D	sema domain, transmembrane domain (TM), and cytoplasmic domain, (semaphorin) 6D
0,97	CYTH1	cytohesin 1
0,97	S100A13	S100 calcium binding protein A13
0,97	MCM7	minichromosome maintenance complex component 7
0,97	DDX39A	DEAD (Asp-Glu-Ala-Asp) box polypeptide 39A
0,97	NOM1	nucleolar protein with MIF4G domain 1
0,97	LRRFIP2	leucine rich repeat (in FLII) interacting protein 2
0,97	PATZ1	POZ (BTB) and AT hook containing zinc finger 1
0,97	IFNAR2	interferon (alpha, beta and omega) receptor 2
0,97	CHRNA5	cholinergic receptor, nicotinic alpha 5
0,97	TANGO6	transport and golgi organization 6 homolog
0,97	UTP15	UTP15, U3 small nucleolar ribonucleoprotein, homolog (S. cerevisiae)
0,97	CAMKMT	Memczak2013 ALT_ACCEPTOR, ALT_DONOR, coding, INTERNAL, intronic best transcript NM_024766
0,97	SLAMF1	signaling lymphocytic activation molecule family member 1
0,97	PRPF3	pre-mRNA processing factor 3
0,97	SLIT3	slit guidance ligand 3
0,97	VAPA	VAMP associated protein A
0,97	IFI27L1	interferon, alpha-inducible protein 27-like 1
0,97	FNBP1	formin binding protein 1
0,97	SETDB2	SET domain, bifurcated 2
0,97	ENTPD2	ectonucleoside triphosphate diphosphohydrolase 2
0,97	DUX3	double homeobox 3
0,97	RIC1	RIC1 homolog, RAB6A GEF complex partner 1
0,97	NFE2L2	nuclear factor, erythroid 2-like 2
0,97	AMZ2	archaelysin family metallopeptidase 2
0,97	HTR3D	5-hydroxytryptamine (serotonin) receptor 3D, ionotropic
0,97	TMEM191C	transmembrane protein 191C
0,97	DNAH17	dynein, axonemal, heavy chain 17
0,97	DEPDC1B	DEP domain containing 1B
0,97	CKLF-CMTM1	CKLF-CMTM1 readthrough
0,97	ZFP62	ZFP62 zinc finger protein
0,97	NCOA6	nuclear receptor coactivator 6
0,97	COL8A2	collagen, type VIII, alpha 2
0,97	AKAP4	A kinase (PRKA) anchor protein 4
0,97	SURF4	surfeit 4
0,97	COL25A1	collagen, type XXV, alpha 1
0,97	CALM1	calmodulin 1 (phosphorylase kinase, delta)
0,97	ZNF572	zinc finger protein 572
0,97	SDC2	syndecan 2
0,97	DOCK9	dedicator of cytokinesis 9
0,97	CTBP2	C-terminal binding protein 2
0,97	SLC35B2; MIR4647	solute carrier family 35 (adenosine 3-phospho 5-phosphosulfate transporter), member B2; microRNA 4647

0,97	CCDC146	coiled-coil domain containing 146
0,97	PLA2G4A	phospholipase A2, group IVA (cytosolic, calcium-dependent)
0,97	HMBS	hydroxymethylbilane synthase
0,97	MKKS	McKusick-Kaufman syndrome
0,97	CCDC109B	coiled-coil domain containing 109B
0,97	IQGAP3	IQ motif containing GTPase activating protein 3
0,97	ERI3; ERI3-IT1	ERI1 exoribonuclease family member 3; ERI3 intronic transcript 1
0,97	TOX	thymocyte selection-associated high mobility group box
0,97	BMPR2	bone morphogenetic protein receptor type II
0,97	CHD1L	chromodomain helicase DNA binding protein 1-like
0,97	DUSP22	dual specificity phosphatase 22
0,97	KRTAP4-11	keratin associated protein 4-11
0,97	PTGES3L-AARSD1; PTGES3L; AARSD1	PTGES3L-AARSD1 readthrough; prostaglandin E synthase 3 (cytosolic)-like; alanyl-tRNA synthetase domain containing 1
0,97	LRRC3B	leucine rich repeat containing 3B
0,97	TMEM223	transmembrane protein 223
0,97	CIRBP	cold inducible RNA binding protein
0,97	NUMB	numb homolog (Drosophila)
0,97	PAX8	paired box 8
0,97	ZNF41	zinc finger protein 41
0,97	PIGT	phosphatidylinositol glycan anchor biosynthesis class T
0,97	FBXO9	F-box protein 9
0,97	RBM47	RNA binding motif protein 47
0,97	MAML3	Memczak2013 ALT_ACCEPTOR, ALT_DONOR, coding, INTERNAL, intronic best transcript NM_018717
0,97	ZNF436-AS1	ZNF436 antisense RNA 1
0,97	SPRED2	sprouty-related, EVH1 domain containing 2
0,97	ATG2B	autophagy related 2B
0,97	BRD9	bromodomain containing 9
0,97	EXOC2	exocyst complex component 2
0,97	RBCK1	RanBP-type and C3HC4-type zinc finger containing 1
0,97	UQCC2	ubiquinol-cytochrome c reductase complex assembly factor 2
0,97	PIF1	PIF1 5-to-3 DNA helicase
0,97	BOLL	boule homolog, RNA binding protein
0,97	C21orf58	chromosome 21 open reading frame 58
0,97	SUMO2	small ubiquitin-like modifier 2
0,97	KCNK16	potassium channel, two pore domain subfamily K, member 16
0,97	ARSE	arylsulfatase E (chondrodysplasia punctata 1)
0,97	INPP5D	inositol polyphosphate-5-phosphatase D
0,97	RPS27A	ribosomal protein S27a
0,97	STK11IP	serine/threonine kinase 11 interacting protein

0,97	NR2C2AP	nuclear receptor 2C2-associated protein
0,97	SFTPB	surfactant protein B
0,97	DNAJB11	DnaJ (Hsp40) homolog, subfamily B, member 11
0,97	GNA12	guanine nucleotide binding protein (G protein) alpha 12
0,97	LINC00282	long intergenic non-protein coding RNA 282
0,97	WSB2	WD repeat and SOCS box containing 2
0,97	RHBDL3	Transcript Identified by AceView, Entrez Gene ID(s) 162494
0,97	HHLA3	HERV-H LTR-associating 3
0,97	SIAE	sialic acid acetylesterase
0,97	ZNF638	zinc finger protein 638
0,97	MRPL12	mitochondrial ribosomal protein L12
0,97	GOLIM4	golgi integral membrane protein 4
0,97	PIWIL2	piwi-like RNA-mediated gene silencing 2
0,97	PSMD10	proteasome 26S subunit, non-ATPase 10
0,97	NOMO3; NOMO2	NODAL modulator 3; NODAL modulator 2
0,97	LPAR1	lysophosphatidic acid receptor 1
0,97	KARS	lysyl-tRNA synthetase
0,97	F10	coagulation factor X
0,97	AHCYL1	adenosylhomocysteinase like 1
0,97	SMARCE1	SWI/SNF related, matrix associated, actin dependent regulator of chromatin, subfamily e, member 1
0,97	AHRR; PDCD6	aryl-hydrocarbon receptor repressor; programmed cell death 6
0,97	MAPRE1	microtubule-associated protein, RP/EB family, member 1
0,97	GBA3	glucosidase, beta, acid 3 (gene/pseudogene)
0,97	C22orf31	chromosome 22 open reading frame 31
0,97	ZMYND11	zinc finger, MYND-type containing 11
0,97	KRTAP9-4	keratin associated protein 9-4
0,97	TAS2R50	taste receptor, type 2, member 50
0,97	LBR	lamin B receptor
0,97	TIMM23	translocase of inner mitochondrial membrane 23 homolog (yeast)
0,97	PPP1R35	protein phosphatase 1, regulatory subunit 35
0,97	TPM1	tropomyosin 1 (alpha)
0,97	SLC2A7	solute carrier family 2 (facilitated glucose transporter), member 7
0,97	TBPL2	TATA box binding protein like 2
0,97	ZNF706	zinc finger protein 706
0,97	POU5F1	POU class 5 homeobox 1
0,97	SRSF7	serine/arginine-rich splicing factor 7
0,97	NBPF10	neuroblastoma breakpoint family, member 10
0,97	ZDHHC17	zinc finger, DHHC-type containing 17
0,97	SRR	serine racemase
0,97	OFCC1	orofacial cleft 1 candidate 1
0,97	SPRED1	sprouty-related, EVH1 domain containing 1
0,97	ZFAND1	zinc finger, AN1-type domain 1

0,97	TK2	thymidine kinase 2, mitochondrial
0,97	SELT; SELT.1	selenoprotein T; SELT protein; Selenoprotein T; cDNA FLJ90525 fis, clone NT2RP4001001, highly similar to Selenoprotein T [Source:UniProtKB/TrEMBL;Acc:Q6IAK0]; Transcript Identified by AceView, Entrez Gene ID(s) 51714
0,97	CAPN13	calpain 13
0,97	MPHOSPH8	M-phase phosphoprotein 8
0,97	CACFD1	calcium channel flower domain containing 1
0,97	PJA1	praja ring finger 1, E3 ubiquitin protein ligase
0,97	PHF7	PHD finger protein 7
0,97	OR4K3	olfactory receptor family 4 subfamily K member 3 (gene/pseudogene) [Source:HGNC Symbol;Acc:HGNC:14731]
0,97	OR6C2	olfactory receptor, family 6, subfamily C, member 2
0,97	NQO2	NAD(P)H dehydrogenase, quinone 2
0,97	PRR23D2	proline rich 23 domain containing 2
0,97	CAPNS1	calpain, small subunit 1
0,97	CTD-3032H12.2; IRX5	Transcript Identified by AceView, Entrez Gene ID(s) 10265; novel transcript
0,97	PDE4DIP	phosphodiesterase 4D interacting protein
0,97	ZNF620	zinc finger protein 620
0,97	SERPINB8	serpin peptidase inhibitor, clade B (ovalbumin), member 8
0,97	FMN1	formin 1
0,97	FAM217A	family with sequence similarity 217, member A
0,97	EMC3	ER membrane protein complex subunit 3
0,97	DEGS2	delta(4)-desaturase, sphingolipid 2
0,97	VWA1	von Willebrand factor A domain containing 1
0,97	VPS45	vacuolar protein sorting 45 homolog (S. cerevisiae)
0,97	OSR2	odd-skipped related transciption factor 2
0,97	TBC1D5	TBC1 domain family, member 5
0,97	CAPN9	calpain 9
0,97	DHX35	Memczak2013 ANTISENSE, coding, INTERNAL, intronic best transcript NM_021931
0,97	CLEC9A	C-type lectin domain family 9, member A
0,97	MMP14	matrix metallopeptidase 14 (membrane-inserted)
0,97	CERS3	ceramide synthase 3
0,97	CELF1	CUGBP, Elav-like family member 1
0,97	PRDM11	PR domain containing 11
0,97	KLHL28	kelch-like family member 28
0,97	SGSM2	small G protein signaling modulator 2
0,97	C5orf49	chromosome 5 open reading frame 49
0,97	NOMO2	NODAL modulator 2
0,97	RNF20	ring finger protein 20, E3 ubiquitin protein ligase
0,97	LTN1	listerin E3 ubiquitin protein ligase 1
0,97	SI	sucrase-isomaltase (alpha-glucosidase)
0,97	BBS10	Bardet-Biedl syndrome 10

0,97	FAM89A; MIR1182	family with sequence similarity 89, member A; microRNA 1182
0,97	ACVRL1	Memczak2013 ANTISENSE, CDS, coding, INTERNAL, intronic best transcript NM_001077401
0,97	CCT7	chaperonin containing TCP1, subunit 7 (eta)
0,97	RUSC1	RUN and SH3 domain containing 1
0,97	GFRA1	GDNF family receptor alpha 1
0,97	FAM83A	family with sequence similarity 83, member A
0,97	AF131215.3; XKR6	Memczak2013 ALT_ACCEPTOR, ALT_DONOR, coding, INTERNAL, intronic best transcript NM_173683; novel transcript
0,97	FAM120AOS	family with sequence similarity 120A opposite strand
0,97	UQCRFS1	ubiquinol-cytochrome c reductase, Rieske iron-sulfur polypeptide 1
0,97	N4BP2L2	NEDD4 binding protein 2-like 2
0,97	FAM122C	family with sequence similarity 122C
0,97	TPGS2	tubulin polyglutamylase complex subunit 2
0,97	GOLGA6D	golgin A6 family, member D
0,97	C17orf99	chromosome 17 open reading frame 99
0,97	ERGIC1	endoplasmic reticulum-golgi intermediate compartment 1
0,97	PLA2G12A	Memczak2013 ANTISENSE, CDS, coding, INTERNAL best transcript NM_030821
0,97	ADAMTS10	ADAM metallopeptidase with thrombospondin type 1 motif 10
0,97	ACOX1	acyl-CoA oxidase 1, palmitoyl
0,97	SF3B2	splicing factor 3b subunit 2
0,97	INHBC	inhibin beta C
0,97	SDHC	succinate dehydrogenase complex, subunit C, integral membrane protein, 15kDa
0,97	TRPC4AP	transient receptor potential cation channel, subfamily C, member 4 associated protein
0,97	IGSF23	immunoglobulin superfamily, member 23

0,97	HDGFRP2	hepatoma-derived growth factor-related protein 2; Hepatoma-derived growth factor-related protein 2 [Source:UniProtKB/Swiss-Prot;Acc:Q7Z4V5]; Salzman2013 ANNOTATED, CDS, coding, OVCODE, OVEXON, UTR5 best transcript NM_001001520; Salzman2013 ANNOTATED, CDS, coding, OVCODE, OVERLAPTX, OVEXON, UTR3, UTR5 best transcript NM_001001520; Salzman2013 ANNOTATED, CDS, coding, INTERNAL, OVCODE, OVEXON best transcript NM_001001520; Salzman2013 ALT_ACCEPTOR, CDS, coding, INTERNAL, intronic, OVCODE, OVEXON best transcript NM_001001520; Salzman2013 ANNOTATED, CDS, coding, OVCODE, OVERLAPTX, OVEXON, UTR3 best transcript NM_001001520; Salzman2013 ALT_DONOR, CDS, coding, INTERNAL, OVCODE, OVERLAPTX, OVEXON, UTR3 best transcript NM_001001520
0,97	HNRNPF	heterogeneous nuclear ribonucleoprotein F
0,97	CD207	CD207 molecule, langerin
0,97	TSPAN32	tetraspanin 32
0,97	EPHB1	EPH receptor B1
0,97	OR1L6	olfactory receptor, family 1, subfamily L, member 6
0,97	GPHN	gephyrin
0,97	AHI1	Abelson helper integration site 1
0,97	NUBPL	nucleotide binding protein-like
0,97	OR10J3	olfactory receptor, family 10, subfamily J, member 3
0,97	BPNT1	3(2), 5-bisphosphate nucleotidase 1
0,97	TRIM56	tripartite motif containing 56
0,97	BUB3	BUB3 mitotic checkpoint protein
0,97	SCARA3	scavenger receptor class A, member 3
0,97	HEATR1	HEAT repeat containing 1
0,97	CAMSAP3	calmodulin regulated spectrin-associated protein family, member 3
0,97	CETP	cholesteryl ester transfer protein, plasma
0,97	TMEM200B	transmembrane protein 200B
0,97	CALCB	calcitonin-related polypeptide beta
0,97	AAR2	AAR2 splicing factor homolog
0,97	DAZAP1	DAZ associated protein 1
0,97	ATG7	autophagy related 7
0,97	ST8SIA1	ST8 alpha-N-acetyl-neuraminide alpha-2,8-sialyltransferase 1
0,97	C7orf73	chromosome 7 open reading frame 73
0,97	RGS3	regulator of G-protein signaling 3
0,97	GABRG3	gamma-aminobutyric acid (GABA) A receptor, gamma 3
0,97	SLC39A5	solute carrier family 39 (zinc transporter), member 5
0,97	TMEM14A	transmembrane protein 14A
0,97	LRPAP1	LDL receptor related protein associated protein 1
0,97	IRF2BP2	interferon regulatory factor 2 binding protein 2

0,97	NSFL1C	NSFL1 (p97) cofactor (p47)
0,97	TCTE3	t-complex-associated-testis-expressed 3
0,97	ALDH7A1	aldehyde dehydrogenase 7 family, member A1
0,97	CRIP3	cysteine-rich protein 3
0,97	C4orf45	chromosome 4 open reading frame 45
0,97	ALS2CL	ALS2 C-terminal like
0,97	CTNNAL1	catenin (cadherin-associated protein), alpha-like 1
0,97	RFX2	regulatory factor X, 2 (influences HLA class II expression)
0,97	GPD1L	glycerol-3-phosphate dehydrogenase 1-like
0,97	MAPK13	mitogen-activated protein kinase 13
0,97	MCIDAS	multiciliate differentiation and DNA synthesis associated cell cycle protein
0,97	B3GNT9	UDP-GlcNAc:betaGal beta-1,3-N-acetylglucosaminyltransferase 9
0,97	MAMDC4	MAM domain containing 4
0,97	AMACR	alpha-methylacyl-CoA racemase
0,97	OR4F29; OR4F3; OR4F16	olfactory receptor, family 4, subfamily F, member 29; olfactory receptor, family 4, subfamily F, member 3; olfactory receptor, family 4, subfamily F, member 16
0,97	OR4F16; OR4F29	olfactory receptor, family 4, subfamily F, member 16; olfactory receptor, family 4, subfamily F, member 29
0,97	PRRX2	paired related homeobox 2
0,97	ICOSLG	inducible T-cell co-stimulator ligand
0,97	OR4X2	olfactory receptor, family 4, subfamily X, member 2 (gene/pseudogene)
0,97	CD8A	CD8a molecule
0,97	DGKD	diacylglycerol kinase, delta 130kDa
0,97	ANKHD1; EIF4EBP3; ANKHD1-EIF4EBP3	ankyrin repeat and KH domain containing 1; eukaryotic translation initiation factor 4E binding protein 3; ANKHD1-EIF4EBP3 readthrough
0,97	UBA6	ubiquitin-like modifier activating enzyme 6
0,97	PARP10	poly(ADP-ribose) polymerase family member 10
0,97	GSE1	Gse1 coiled-coil protein
0,97	ZNF254	zinc finger protein 254
0,97	PHLDA3	pleckstrin homology-like domain, family A, member 3
0,97	BVES	blood vessel epicardial substance
0,97	RAB11B	RAB11B, member RAS oncogene family
0,97	NDUFAF2	NADH dehydrogenase (ubiquinone) complex I, assembly factor 2
0,97	NKIRAS2	Memczak2013 ANTISENSE, coding, INTERNAL, UTR3 best transcript NM_001001349
0,97	DEF8	differentially expressed in FDCP 8 homolog (mouse)
0,97	CSE1L	CSE1 chromosome segregation 1-like (yeast)
0,97	CD200	CD200 molecule
0,97	SERINC5	serine incorporator 5
0,97	DYRK1B	dual specificity tyrosine-(Y)-phosphorylation regulated kinase 1B

0,97	TIMM13	translocase of inner mitochondrial membrane 13 homolog (yeast)
0,97	HCCS	holocytochrome c synthase
0,97	ZBTB33	zinc finger and BTB domain containing 33
0,97	DNAJC19	DnaJ (Hsp40) homolog, subfamily C, member 19
0,97	B4GALT1	UDP-Gal:betaGlcNAc beta 1,4- galactosyltransferase, polypeptide 1
0,97	HSD17B2	hydroxysteroid (17-beta) dehydrogenase 2
0,97	DEFB110	defensin, beta 110
0,97	OLIG1	oligodendrocyte transcription factor 1
0,97	CTBP1-AS2	CTBP1 antisense RNA 2 (head to head)
0,97	HIVEP3	human immunodeficiency virus type I enhancer binding protein 3
0,97	PRAMEF7	PRAME family member 7
0,97	SEMA7A	semaphorin 7A, GPI membrane anchor (John Milton Hagen blood group)
0,97	CAPZA1	capping protein (actin filament) muscle Z-line, alpha 1
0,97	PTGER2	prostaglandin E receptor 2
0,97	TUBB8	tubulin, beta 8 class VIII
0,97	MSI2	musashi RNA binding protein 2
0,97	PGAM2	phosphoglycerate mutase 2 (muscle)
0,97	SGSM1	small G protein signaling modulator 1
0,97	TAS2R13	taste receptor, type 2, member 13
0,97	FAM227B	family with sequence similarity 227, member B
0,97	TMEM165	transmembrane protein 165
0,97	PKP4	plakophilin 4
0,97	SBF2	SET binding factor 2
0,97	ARID1B	AT rich interactive domain 1B (SWI1-like)
0,97	PTPRQ	protein tyrosine phosphatase, receptor type, Q
0,97	GSK3B	glycogen synthase kinase 3 beta
0,97	PITRM1	pitrilysin metallopeptidase 1
0,97	ZDHHC3	zinc finger, DHHC-type containing 3
0,97	BNIP3L	BCL2/adenovirus E1B 19kDa interacting protein 3-like
0,97	KRT38	keratin 38, type I
0,97	ADCK4	aarF domain containing kinase 4
0,97	NFKBIZ	nuclear factor of kappa light polypeptide gene enhancer in B-cells inhibitor, zeta
0,97	CIDEA	cell death-inducing DFFA-like effector a
0,97	COPS6	COP9 signalosome subunit 6
0,97	PCDHB2	protocadherin beta 2
0,97	PRAMEF7	PRAME family member 7
0,97	ACVR1	activin A receptor type I
0,97	CLEC2A	C-type lectin domain family 2, member A
0,97	PTGDR	prostaglandin D2 receptor (DP)
0,97	ZBTB38	zinc finger and BTB domain containing 38
0,97	OR51S1	olfactory receptor, family 51, subfamily S, member 1
0,97	LRP6	LDL receptor related protein 6
0,97	FDCSP	follicular dendritic cell secreted protein

0,97	ZNF721	Salzman2013 ALT_ACCEPTOR, ALT_DONOR, coding, INTERNAL, intronic best transcript NM_133474
0,97	MKRN1	makorin ring finger protein 1
0,97	ARL6IP1	ADP-ribosylation factor like GTPase 6 interacting protein 1
0,97	DHX8	DEAH (Asp-Glu-Ala-His) box polypeptide 8
0,97	WDR4	WD repeat domain 4
0,97	BAIAP2L1	BAI1-associated protein 2-like 1
0,97	SLC25A6	solute carrier family 25 (mitochondrial carrier; adenine nucleotide translocator), member 6
0,97	NSRP1; MIR423	nuclear speckle splicing regulatory protein 1; microRNA 423
0,97	ELMO3	engulfment and cell motility 3
0,97	MID1	midline 1
0,97	FLVCR1	feline leukemia virus subgroup C cellular receptor 1
0,97	FHOD1	formin homology 2 domain containing 1
0,97	FBXO47	F-box protein 47
0,97	COLCA1	colorectal cancer associated 1
0,97	DCTN2	dynactin 2 (p50)
0,97	KRT79	keratin 79, type II
0,97	STEAP4	STEAP family member 4
0,97	TRABD2A	TraB domain containing 2A
0,97	PRKCSH	protein kinase C substrate 80K-H
0,97	UCKL1; MIR1914	uridine-cytidine kinase 1-like 1; microRNA 1914
0,97	SEC14L6	SEC14-like lipid binding 6
0,97	GATC	glutamyl-tRNA(Gln) amidotransferase, subunit C
0,97	ASAP2	ArfGAP with SH3 domain, ankyrin repeat and PH domain 2
0,97	ZNF735	zinc finger protein 735
0,97	EMC2	ER membrane protein complex subunit 2
0,97	SLC16A10	solute carrier family 16 (aromatic amino acid transporter), member 10
0,97	C12orf29	chromosome 12 open reading frame 29
0,97	FOXD4	forkhead box D4
0,97	USP35	ubiquitin specific peptidase 35
0,97	KAT6B	K(lysine) acetyltransferase 6B
0,97	ZNF33B	zinc finger protein 33B
0,97	RDX	radixin
0,97	NSL1	NSL1, MIS12 kinetochore complex component
0,97	PRKD3	protein kinase D3
0,97	ZKSCAN4	zinc finger with KRAB and SCAN domains 4
0,97	NBPF9	neuroblastoma breakpoint family, member 9
0,97	TNFAIP2	tumor necrosis factor, alpha-induced protein 2
0,97	MPDZ	multiple PDZ domain protein
0,97	OR51J1	olfactory receptor, family 51, subfamily J, member 1 (gene/pseudogene)
0,97	LEFTY2	left-right determination factor 2
0,97	OR10W1	olfactory receptor, family 10, subfamily W, member 1

0,97	ICK	intestinal cell (MAK-like) kinase
0,97	PTPN13	protein tyrosine phosphatase, non-receptor type 13 (APO-1/CD95 (Fas)-associated phosphatase)
0,97	STPG1	sperm-tail PG-rich repeat containing 1
0,97	PXDC1	PX domain containing 1
0,97	SIN3A	SIN3 transcription regulator family member A
0,97	RGS10	regulator of G-protein signaling 10
0,97	LRCH2	leucine-rich repeats and calponin homology (CH) domain containing 2
0,97	DDX54	DEAD (Asp-Glu-Ala-Asp) box polypeptide 54
0,97	ITGA2B	integrin alpha 2b
0,97	APOBEC1	apolipoprotein B mRNA editing enzyme, catalytic polypeptide 1
0,97	ABHD5	abhydrolase domain containing 5
0,97	PQLC2	PQ loop repeat containing 2
0,97	ATG4D	autophagy related 4D, cysteine peptidase
0,97	L2HGDH	L-2-hydroxyglutarate dehydrogenase
0,97	ZBTB8A	zinc finger and BTB domain containing 8A
0,97	RMI2	RecQ mediated genome instability 2
0,97	TMPRSS3	transmembrane protease, serine 3
0,97	C1orf52	chromosome 1 open reading frame 52
0,97	ZDHHC3	zinc finger, DHHC-type containing 3
0,97	MUC5B	mucin 5B, oligomeric mucus/gel-forming
0,97	COBL	cordon-bleu WH2 repeat protein
0,97	H2AFV	H2A histone family, member V
0,97	C17orf47	chromosome 17 open reading frame 47
0,97	PIK3CB	phosphatidylinositol-4,5-bisphosphate 3-kinase, catalytic subunit beta
0,97	KRTAP6-2	keratin associated protein 6-2
0,97	HMSD	histocompatibility (minor) serpin domain containing
0,97	MRPL48	mitochondrial ribosomal protein L48
0,97	TUBGCP6	tubulin, gamma complex associated protein 6
0,97	MYOM1	myomesin 1
0,97	PARP4	poly(ADP-ribose) polymerase family member 4
0,97	TMEM160	transmembrane protein 160
0,97	APOA1BP	apolipoprotein A-I binding protein
0,97	ZNF780A	zinc finger protein 780A
0,97	CRB3	crumbs family member 3
0,97	TLX2	T-cell leukemia homeobox 2
0,97	CECR6	cat eye syndrome chromosome region, candidate 6
0,97	ZNF546	zinc finger protein 546
0,97	TOP3A	topoisomerase (DNA) III alpha
0,97	PABPC4L	poly(A) binding protein, cytoplasmic 4-like
0,97	ZNF536	zinc finger protein 536
0,97	EMILIN1	elastin microfibril interfacer 1
0,97	CTSL3P	cathepsin L family member 3, pseudogene
0,97	GTF3A	general transcription factor IIIA
0,97	JMY	junction mediating and regulatory protein, p53 cofactor
0,97	CHD1	chromodomain helicase DNA binding protein 1

0,97	APH1A	APH1A gamma secretase subunit
0,97	SFMBT2	Scm-like with four mbt domains 2
0,97	TRAFD1	TRAF-type zinc finger domain containing 1
0,97	RPS23	ribosomal protein S23
0,97	SIGLEC16	sialic acid binding Ig-like lectin 16 (gene/pseudogene)
0,97	HSF4; FBXL8	heat shock transcription factor 4; F-box and leucine-rich repeat protein 8
0,97	CARD10	caspase recruitment domain family, member 10
0,97	OR13C3	olfactory receptor, family 13, subfamily C, member 3
0,97	DNAI1	dynein, axonemal, intermediate chain 1
0,97	COPS2	COP9 signalosome subunit 2
0,97	SETDB1	SET domain, bifurcated 1
0,97	LAMTOR5	late endosomal/lysosomal adaptor, MAPK and MTOR activator 5
0,97	CXCR4	chemokine (C-X-C motif) receptor 4
0,97	DDX56	DEAD (Asp-Glu-Ala-Asp) box helicase 56
0,97	KLRB1	killer cell lectin-like receptor subfamily B, member 1
0,97	HTRA3	HtrA serine peptidase 3
0,97	DNAJB12	DnaJ (Hsp40) homolog, subfamily B, member 12
0,97	ICMT	isoprenylcysteine carboxyl methyltransferase
0,97	ACP1	acid phosphatase 1, soluble
0,97	PRKRA	protein kinase, interferon-inducible double stranded RNA dependent activator
0,97	KRTAP5-11	keratin associated protein 5-11
0,97	UBALD2	UBA-like domain containing 2
0,97	RMND1	required for meiotic nuclear division 1 homolog
0,97	PDILT	protein disulfide isomerase-like, testis expressed
0,97	SP100	SP100 nuclear antigen
0,97	MED7	mediator complex subunit 7
0,97	NPIPB9	nuclear pore complex interacting protein family, member B9
0,97	ZIC2	Zic family member 2
0,97	LIMD1	LIM domains containing 1
0,97	PSG11	pregnancy specific beta-1-glycoprotein 11
0,97	HTRA4	HtrA serine peptidase 4
0,97	NUP205	nucleoporin 205kDa
0,97	AKT3	v-akt murine thymoma viral oncogene homolog 3
0,97	GP9	glycoprotein IX (platelet)
0,97	KCNG2	potassium channel, voltage gated modifier subfamily G, member 2
0,97	APLP2	amyloid beta (A4) precursor-like protein 2
0,97	KPNA7	karyopherin alpha 7 (importin alpha 8)
0,97	C17orf82	chromosome 17 open reading frame 82
0,97	TMTC1	transmembrane and tetratricopeptide repeat containing 1
0,97	TMEM38A	transmembrane protein 38A
0,97	GLI1	GLI family zinc finger 1
0,97	BMP3	bone morphogenetic protein 3
0,97	ARHGEF5	Rho guanine nucleotide exchange factor 5
0,97	SCAND1	SCAN domain containing 1

0,97	KRTAP10-6	keratin associated protein 10-6
0,97	DDX43	DEAD (Asp-Glu-Ala-Asp) box polypeptide 43
0,97	FKBP9	FK506 binding protein 9
0,97	SLC22A10	solute carrier family 22, member 10
0,97	RNF168	ring finger protein 168, E3 ubiquitin protein ligase
0,97	ICAM4	intercellular adhesion molecule 4 (Landsteiner-Wiener blood group)
0,97	PPP2R1A	protein phosphatase 2, regulatory subunit A, alpha
0,97	SPN	sialophorin
0,97	FAM58A	family with sequence similarity 58, member A
0,97	MOSPD2	motile sperm domain containing 2
0,97	HACL1	2-hydroxyacyl-CoA lyase 1
0,97	ANAPC7	anaphase promoting complex subunit 7
0,97	KIF15	kinesin family member 15
0,97	TRIM73	tripartite motif containing 73
0,97	OR6S1	olfactory receptor, family 6, subfamily S, member 1
0,97	LARP4	La ribonucleoprotein domain family, member 4
0,97	SLC2A4RG	SLC2A4 regulator
0,97	ZNF624	zinc finger protein 624
0,97	LINC01578	long intergenic non-protein coding RNA 1578
0,97	ISLR	immunoglobulin superfamily containing leucine-rich repeat
0,97	TMEM240	transmembrane protein 240
0,97	TMUB1	transmembrane and ubiquitin-like domain containing 1
0,97	MMP9	matrix metallopeptidase 9
0,97	PPP2CB	protein phosphatase 2, catalytic subunit, beta isozyme
0,97	BARX2	BARX homeobox 2
0,97	FXYD1	FXYD domain containing ion transport regulator 1
0,97	RYBP	RING1 and YY1 binding protein
0,97	C1GALT1C1	C1GALT1 specific chaperone 1
0,97	DDX53	DEAD (Asp-Glu-Ala-Asp) box polypeptide 53
0,97	SURF4	surfeit 4
0,97	MAP2K4; MIR744	mitogen-activated protein kinase kinase 4; microRNA 744
0,97	TPSG1	tryptase gamma 1
0,97	LCE3B	late cornified envelope 3B
0,97	CT45A6; CT45A7	cancer/testis antigen family 45, member A6; cancer/testis antigen family 45, member A7
0,97	SPAG9	sperm associated antigen 9
0,97	OR52E6	olfactory receptor, family 52, subfamily E, member 6
0,97	SUMF1	sulfatase modifying factor 1
0,97	DYRK1B	dual specificity tyrosine-(Y)-phosphorylation regulated kinase 1B
0,97	ZBED5	zinc finger, BED-type containing 5
0,97	KRTAP3-1	keratin associated protein 3-1
0,97	CYTH3	cytohesin 3
0,97	ARID4B	AT rich interactive domain 4B (RBP1-like)
0,97	DYNAP	dynactin associated protein
0,97	KRTAP16-1	keratin associated protein 16-1

0,97	UBB	ubiquitin B
0,97	TMEM200A	transmembrane protein 200A
0,97	PRB3	proline-rich protein BstNI subfamily 3
0,97	REXO4	REX4 homolog, 3'-5' exonuclease [Source:HGNC Symbol;Acc:HGNC:12820]
0,97	UBE2W	ubiquitin-conjugating enzyme E2W (putative)
0,97	AK5	adenylate kinase 5
0,97	PPP1R3D	protein phosphatase 1, regulatory subunit 3D
0,97	JPH1	junctophilin 1
0,97	LINC00260	long intergenic non-protein coding RNA 260
0,96	SNORD89; RNF149	small nucleolar RNA, C/D box 89; ring finger protein 149
0,96	CDC42EP4	CDC42 effector protein (Rho GTPase binding) 4
0,96	LONP2	lon peptidase 2, peroxisomal
0,96	DOK2	docking protein 2
0,96	UTP23	UTP23, small subunit (SSU) processome component, homolog (yeast)
0,96	CGB8	chorionic gonadotropin, beta polypeptide 8
0,96	DPM1	dolichyl-phosphate mannosyltransferase polypeptide 1, catalytic subunit
0,96	OR2J1	olfactory receptor, family 2, subfamily J, member 1 (gene/pseudogene)
0,96	ITSN1	intersectin 1
0,96	SNRPB	small nuclear ribonucleoprotein polypeptides B and B1
0,96	TEN1; CDK3; TEN1-CDK3	TEN1 CST complex subunit; cyclin-dependent kinase 3; TEN1-CDK3 readthrough (NMD candidate)
0,96	DHRS1	dehydrogenase/reductase (SDR family) member 1
0,96	TIMP4	TIMP metallopeptidase inhibitor 4
0,96	PRDM2	PR domain containing 2, with ZNF domain
0,96	HKR1	Memczak2013 ANTISENSE, CDS, coding, INTERNAL best transcript NM_181786
0,96	ZMAT3	zinc finger, matrin-type 3
0,96	ZFP92	ZFP92 zinc finger protein
0,96	TRAM1L1	translocation associated membrane protein 1-like 1
0,96	CCZ1; CCZ1B	CCZ1 homolog, vacuolar protein trafficking and biogenesis associated; CCZ1 homolog B, vacuolar protein trafficking and biogenesis associated
0,96	CAGE1	cancer antigen 1
0,96	CYP4F2	cytochrome P450, family 4, subfamily F, polypeptide 2
0,96	FOS	FBJ murine osteosarcoma viral oncogene homolog
0,96	TIMM23B	translocase of inner mitochondrial membrane 23 homolog B (yeast)
0,96	XKR7	X-linked Kx blood group related 7
0,96	NLGN3	neuroligin 3
0,96	MCM3AP	minichromosome maintenance complex component 3 associated protein
0,96	CCNYL1; MIR4775	cyclin Y like 1; microRNA 4775

0,96	TCL1B; TCL6	T-cell leukemia/lymphoma 1B; T-cell leukemia/lymphoma 6 (non-protein coding)
0,96	SCRN1	secernin 1
0,96	CCT3	chaperonin containing TCP1, subunit 3 (gamma)
0,96	CAT	Memczak2013 ANTISENSE, CDS, coding, INTERNAL, UTR3 best transcript NM_001752
0,96	LCMT2	leucine carboxyl methyltransferase 2
0,96	RABL3	RAB, member of RAS oncogene family-like 3
0,96	RGS9BP	regulator of G protein signaling 9 binding protein
0,96	RHOD	ras homolog family member D
0,96	NABP2	nucleic acid binding protein 2
0,96	LSM6	Transcript Identified by AceView, Entrez Gene ID(s) 11157
0,96	GJA4	gap junction protein alpha 4
0,96	ZWILCH	zwilch kinetochore protein
0,96	CMPK2	cytidine monophosphate (UMP-CMP) kinase 2, mitochondrial
0,96	SLC12A4	solute carrier family 12 (potassium/chloride transporter), member 4
0,96	GPR107	G protein-coupled receptor 107
0,96	CNTD2	cyclin N-terminal domain containing 2
0,96	STOML2	stomatin (EPB72)-like 2
0,96	S1PR4	sphingosine-1-phosphate receptor 4
0,96	KIF13A	kinesin family member 13A
0,96	SSX8	synovial sarcoma, X breakpoint 8
0,96	TARDBP	TAR DNA binding protein
0,96	PLA2G4F	phospholipase A2, group IVF
0,96	CCDC85B	coiled-coil domain containing 85B
0,96	ZC3H7A	zinc finger CCCH-type containing 7A
0,96	SLC1A5	solute carrier family 1 (neutral amino acid transporter), member 5
0,96	CGRRF1	cell growth regulator with ring finger domain 1
0,96	TPK1	thiamin pyrophosphokinase 1
0,96	HLCS	holocarboxylase synthetase (biotin-(proprionyl-CoA-carboxylase (ATP-hydrolysing)) ligase)
0,96	FASTK	Fas-activated serine/threonine kinase
0,96	MYO1F	Jeck2013 ANTISENSE, CDS, coding, INTERNAL, intronic, OVCODE, OVEXON best transcript NM_012335
0,96	ADIRF; AGAP11; BMS1P3	adipogenesis regulatory factor; ankyrin repeat and GTPase domain Arf GTPase activating protein 11; BMS1 ribosome biogenesis factor pseudogene 3
0,96	HDAC6	histone deacetylase 6
0,96	MGAM	maltase-glucoamylase
0,96	SERPINA3	serpin peptidase inhibitor, clade A (alpha-1 antiproteinase, antitrypsin), member 3
0,96	FLVCR2	feline leukemia virus subgroup C cellular receptor family, member 2
0,96	STK39	serine threonine kinase 39
0,96	INTS3	integrator complex subunit 3
0,96	HEXA	hexosaminidase A (alpha polypeptide)

0,96	LIMA1	LIM domain and actin binding 1
0,96	ATP5H	ATP synthase, H+ transporting, mitochondrial Fo complex subunit D
0,96	ETFA	electron-transfer-flavoprotein, alpha polypeptide
0,96	TMEM222	transmembrane protein 222
0,96	DDX52	DEAD (Asp-Glu-Ala-Asp) box polypeptide 52
0,96	RGS20	regulator of G-protein signaling 20
0,96	THOC2	THO complex 2
0,96	POFUT1; MIR1825	protein O-fucosyltransferase 1; microRNA 1825
0,96	FRMPD2	FERM and PDZ domain containing 2
0,96	LINGO1	leucine rich repeat and Ig domain containing 1
0,96	PI4KAP2	phosphatidylinositol 4-kinase, catalytic, alpha pseudogene 2
0,96	OR13C2	olfactory receptor, family 13, subfamily C, member 2
0,96	GRB2	growth factor receptor bound protein 2
0,96	RASGRP2	RAS guanyl releasing protein 2 (calcium and DAG-regulated)
0,96	KRT20	keratin 20, type I
0,96	ABHD15	abhydrolase domain containing 15
0,96	SCGB1D1	secretoglobin, family 1D, member 1
0,96	YY1AP1	YY1 associated protein 1
0,96	RPS10	ribosomal protein S10
0,96	CENPM	centromere protein M
0,96	POLD3	polymerase (DNA-directed), delta 3, accessory subunit
0,96	SLFNL1	schlafen like 1
0,96	ITPKB	inositol-trisphosphate 3-kinase B
0,96	INPP5D	inositol polyphosphate-5-phosphatase D
0,96	COQ9	coenzyme Q9
0,96	KRT18	keratin 18, type I
0,96	TRIM7	tripartite motif containing 7
0,96	RSAD2	radical S-adenosyl methionine domain containing 2
0,96	OPRL1	opiate receptor-like 1
0,96	PHF13	PHD finger protein 13
0,96	MED13	mediator complex subunit 13
0,96	TIGD4	tigger transposable element derived 4
0,96	WDYHV1	WDYHV motif containing 1
0,96	TCF25	transcription factor 25 (basic helix-loop-helix)
0,96	CTSL	cathepsin L
0,96	DPY19L4	dpy-19-like 4 (C. elegans)
0,96	LRIG1	leucine-rich repeats and immunoglobulin-like domains 1
0,96	S100A4	S100 calcium binding protein A4
0,96	UBR3	Transcript Identified by AceView, Entrez Gene ID(s) 130507
0,96	HIPK4	homeodomain interacting protein kinase 4
0,96	RPS7	ribosomal protein S7
0,96	ZDHHC3	zinc finger, DHHC-type containing 3
0,96	MTF1	metal-regulatory transcription factor 1

0,96	KLK13	kallikrein related peptidase 13
0,96	CNTROB	centrobin, centrosomal BRCA2 interacting protein
0,96	TNFRSF25	tumor necrosis factor receptor superfamily, member 25
0,96	NTPCR	nucleoside-triphosphatase, cancer-related
0,96	PPARD	peroxisome proliferator-activated receptor delta
0,96	DBNDD2	dysbindin (dystrobrevin binding protein 1) domain containing 2
0,96	SDHB	succinate dehydrogenase complex subunit B, iron sulfur (Ip)
0,96	LMO3	LIM domain only 3 (rhombotin-like 2)
0,96	RPS15A	ribosomal protein S15a
0,96	SERPINB2	serpin peptidase inhibitor, clade B (ovalbumin), member 2
0,96	PIP	prolactin-induced protein
0,96	SOS1	SOS Ras/Rac guanine nucleotide exchange factor 1
0,96	CDRT15	CMT1A duplicated region transcript 15
0,96	RBAK; RBAK-RBAKDN; RBAKDN	RB-associated KRAB zinc finger; RBAK-RBAKDN readthrough; RBAK downstream neighbor (non-protein coding)
0,96	DAXX	death-domain associated protein
0,96	PER1; MIR6883	period circadian clock 1; microRNA 6883
0,96	SMPD1	sphingomyelin phosphodiesterase 1, acid lysosomal
0,96	DDAH1	dimethylarginine dimethylaminohydrolase 1
0,96	MAP1LC3B2	microtubule-associated protein 1 light chain 3 beta 2
0,96	EML5	Transcript Identified by AceView, Entrez Gene ID(s) 161436
0,96	ICE1	interactor of little elongation complex ELL subunit 1
0,96	RPL7	ribosomal protein L7
0,96	HSF1	heat shock transcription factor 1
0,96	MCMDC2	minichromosome maintenance domain containing 2
0,96	ATAD1	ATPase family, AAA domain containing 1
0,96	SLC1A6	solute carrier family 1 (high affinity aspartate/glutamate transporter), member 6
0,96	GGCX	gamma-glutamyl carboxylase
0,96	TTC31	tetratricopeptide repeat domain 31
0,96	TIMELESS	timeless circadian clock
0,96	RPL24	ribosomal protein L24
0,96	PDZD3	PDZ domain containing 3
0,96	ANKRD34C	ankyrin repeat domain 34C
0,96	IARS2	isoleucyl-tRNA synthetase 2, mitochondrial
0,96	LSM4	LSM4 homolog, U6 small nuclear RNA and mRNA degradation associated
0,96	CIAPIN1	cytokine induced apoptosis inhibitor 1
0,96	CDY1B; CDY1	chromodomain protein, Y-linked, 1B; chromodomain protein, Y-linked, 1
0,96	TAS2R3	taste receptor, type 2, member 3
0,96	HTR7	5-hydroxytryptamine (serotonin) receptor 7, adenylate cyclase-coupled
0,96	FAM81A	family with sequence similarity 81, member A

0,96	SNX17	sorting nexin 17
0,96	TUFM; MIR4721	Tu translation elongation factor, mitochondrial; microRNA 4721
0,96	DNASE2	deoxyribonuclease II, lysosomal
0,96	RHEBL1	Ras homolog enriched in brain like 1
0,96	PREX1	Memczak2013 ALT_ACCEPTOR, ALT_DONOR, coding, INTERNAL, intronic best transcript NM_020820
0,96	CRBN	cereblon
0,96	SAR1A	secretion associated, Ras related GTPase 1A
0,96	OXR1	oxidation resistance 1
0,96	MTMR1	myotubularin related protein 1
0,96	OLFM3	olfactomedin 3
0,96	TMPRSS11A	transmembrane protease, serine 11A
0,96	TRAPPC4	trafficking protein particle complex 4
0,96	WIPF1	WAS/WASL interacting protein family, member 1
0,96	WTAP	Wilms tumor 1 associated protein
0,96	ANXA3	annexin A3
0,96	YKT6	YKT6 v-SNARE homolog (S. cerevisiae)
0,96	ACPT	acid phosphatase, testicular
0,96	SKIV2L	SKI2 homolog, superkiller viralicidic activity 2-like
0,96	AK8	adenylate kinase 8
0,96	FAM21A	family with sequence similarity 21, member A
0,96	DNAJB6	DnaJ (Hsp40) homolog, subfamily B, member 6
0,96	CENPP	centromere protein P
0,96	NDUFA6	NADH dehydrogenase (ubiquinone) 1 alpha subcomplex, 6, 14kDa
0,96	ZNF800	zinc finger protein 800
0,96	EDF1	endothelial differentiation-related factor 1
0,96	CTNNA1	catenin (cadherin-associated protein), alpha 1
0,96	PSIP1	PC4 and SFRS1 interacting protein 1
0,96	SLC16A5	solute carrier family 16 (monocarboxylate transporter), member 5
0,96	PCDH11Y	protocadherin 11 Y-linked
0,96	TPST2	tyrosylprotein sulfotransferase 2
0,96	EMX1	empty spiracles homeobox 1
0,96	TSPY3; LOC728395	Homo sapiens testis specific protein, Y-linked 3, mRNA (cDNA clone MGC:103998 IMAGE:30915406), complete cds.; Homo sapiens similar to testis specific protein, Y-linked 1, mRNA (cDNA clone MGC:150413 IMAGE:40120433), complete cds.; Homo sapiens testis specific protein, Y-linked 3, mRNA (cDNA clone MGC:150414 IMAGE:40120435), complete cds.
0,96	VSIG4	V-set and immunoglobulin domain containing 4
0,96	ZP1	zona pellucida glycoprotein 1 (sperm receptor)
0,96	SOX7	SRY box 7
0,96	TMEM41B	transmembrane protein 41B
0,96	MRPL50	mitochondrial ribosomal protein L50
0,96	SLC35G3	solute carrier family 35, member G3

0,96	TMEM63C	transmembrane protein 63C
0,96	MTHFS	5,10-methenyltetrahydrofolate synthetase (5-formyltetrahydrofolate cyclo-ligase)
0,96	MRM1	mitochondrial rRNA methyltransferase 1
0,96	UTP18	UTP18 small subunit (SSU) processome component
0,96	AMBN	ameloblastin
0,96	GCM2	glial cells missing homolog 2 (Drosophila)
0,96	FAM212A	family with sequence similarity 212, member A
0,96	PIK3C2B	phosphatidylinositol-4-phosphate 3-kinase, catalytic subunit type 2 beta
0,96	UGT2A2; UGT2A1	UDP glucuronosyltransferase 2 family, polypeptide A2; UDP glucuronosyltransferase 2 family, polypeptide A1, complex locus
0,96	PTP4A3	protein tyrosine phosphatase type IVA, member 3
0,96	CCND3	Memczak2013 ALT_ACCEPTOR, ALT_DONOR, coding, INTERNAL, intronic best transcript NM_001136017
0,96	FBXO11	F-box protein 11
0,96	RASA4B; RASA4	RAS p21 protein activator 4B; RAS p21 protein activator 4
0,96	CNTNAP3	contactin associated protein-like 3
0,96	LPCAT4	lysophosphatidylcholine acyltransferase 4
0,96	SRGAP2C	SLIT-ROBO Rho GTPase activating protein 2C
0,96	OR52B6	olfactory receptor, family 52, subfamily B, member 6
0,96	GLRA1	glycine receptor alpha 1
0,96	C9orf129	chromosome 9 open reading frame 129
0,96	C1orf111	chromosome 1 open reading frame 111
0,96	C1orf216	chromosome 1 open reading frame 216
0,96	STX3	syntaxin 3
0,96	RNASEL	ribonuclease L (2,5-oligoisoadenylate synthetase-dependent)
0,96	GGTLC2	gamma-glutamyltransferase light chain 2
0,96	LOC101928841; RP11-102K13.5	collagen alpha-1(II) chain-like; novel transcript
0,96	CSF3R	colony stimulating factor 3 receptor
0,96	USP17L4	ubiquitin specific peptidase 17-like family member 4
0,96	SLC20A1	solute carrier family 20 (phosphate transporter), member 1
0,96	DAO	D-amino-acid oxidase
0,96	PNOC	prepronociceptin
0,96	NCBP2	nuclear cap binding protein subunit 2
0,96	C20orf203	chromosome 20 open reading frame 203
0,96	CD37	CD37 molecule
0,96	EPHB2	EPH receptor B2
0,96	TRERF1	transcriptional regulating factor 1
0,96	COX17	COX17 cytochrome c oxidase copper chaperone
0,96	EBF1	early B-cell factor 1
0,96	TRIM37	tripartite motif containing 37
0,96	S100P	S100 calcium binding protein P
0,96	RERE	arginine-glutamic acid dipeptide (RE) repeats

0,96	ATXN10	ataxin 10
0,96	PIK3R4	phosphoinositide-3-kinase, regulatory subunit 4
0,96	SIDT1	SID1 transmembrane family, member 1
0,96	UGT2B17	UDP glucuronosyltransferase 2 family, polypeptide B17
0,96	REPS2	RALBP1 associated Eps domain containing 2
0,96	STUB1	STIP1 homology and U-box containing protein 1, E3 ubiquitin protein ligase
0,96	USP17L13	ubiquitin specific peptidase 17-like family member 13
0,96	PEX10	peroxisomal biogenesis factor 10
0,96	PTMS	parathymosin
0,96	U2AF1	U2 small nuclear RNA auxiliary factor 1
0,96	NAT6	N-acetyltransferase 6 (GCN5-related)
0,96	OR10A5	olfactory receptor, family 10, subfamily A, member 5
0,96	MTRF1	mitochondrial translational release factor 1
0,96	FBXO3	F-box protein 3
0,96	TRAPPC12	trafficking protein particle complex 12
0,96	ZNF492	zinc finger protein 492
0,96	EPHA4	Transcript Identified by AceView, Entrez Gene ID(s) 2043
0,96	RPL41	ribosomal protein L41
0,96	CDK5RAP2	CDK5 regulatory subunit associated protein 2
0,96	LOC103611081; LOC100507431; RP11-890B15.3; RP11-890B15.2; klerglaw	uncharacterized LOC103611081; uncharacterized LOC100507431; Transcript Identified by AceView; novel transcript
0,96	EXOC4	exocyst complex component 4
0,96	MFAP2	microfibrillar associated protein 2
0,96	SMG7	SMG7 nonsense mediated mRNA decay factor
0,96	ADIRF-AS1; RP11-96C23.15; MMRN2	ADIRF antisense RNA 1 [Source:HGNC Symbol;Acc:HGNC:45127]; Transcript Identified by AceView, Entrez Gene ID(s) 79812; ADIRF antisense RNA 1; novel transcript, antisense to ADIRF
0,96	LPP	LIM domain containing preferred translocation partner in lipoma
0,96	HACD1	3-hydroxyacyl-CoA dehydratase 1
0,96	ZDHHC3	zinc finger, DHHC-type containing 3
0,96	HEG1	heart development protein with EGF-like domains 1
0,96	TXLNGY	taxilin gamma pseudogene, Y-linked
0,96	C1orf61	chromosome 1 open reading frame 61
0,96	DZIP1	Transcript Identified by AceView, Entrez Gene ID(s) 22873
0,96	CCDC40	coiled-coil domain containing 40
0,96	ST3GAL1	ST3 beta-galactoside alpha-2,3-sialyltransferase 1
0,96	PIK3CG	Memczak2013 ANTISENSE, CDS, coding, INTERNAL best transcript NM_002649
0,96	CCT6A	chaperonin containing TCP1, subunit 6A (zeta 1)
0,96	ZNF860	zinc finger protein 860
0,96	CTAGE9	CTAGE family, member 9

0,96	TGM2	transglutaminase 2
0,96	AQR	aquarius intron-binding spliceosomal factor
0,96	CYC1	cytochrome c-1
0,96	TMEM219	transmembrane protein 219
0,96	LOC653653; AC025048.1	adaptor-related protein complex 1, sigma 2 subunit pseudogene
0,96	ANKRD18A; FAM95C	ankyrin repeat domain 18A; family with sequence similarity 95, member C
0,96	DPYSL2	dihydropyrimidinase-like 2
0,96	ZMYND15	zinc finger, MYND-type containing 15
0,96	UBN2	ubinuclein 2
0,96	KIAA0895	KIAA0895
0,96	PRRC2C	proline-rich coiled-coil 2C
0,96	ACADM	acyl-CoA dehydrogenase, C-4 to C-12 straight chain
0,96	SERPINA1	serpin peptidase inhibitor, clade A (alpha-1 antiproteinase, antitrypsin), member 1
0,96	CCL2	chemokine (C-C motif) ligand 2
0,96	RICTOR	RPTOR independent companion of MTOR, complex 2
0,96	TGM4	transglutaminase 4
0,96	TAF12	TAF12 RNA polymerase II, TATA box binding protein (TBP)-associated factor, 20kDa
0,96	GLT8D1	glycosyltransferase 8 domain containing 1
0,96	RAD18	RAD18 E3 ubiquitin protein ligase
0,96	SNX1	sorting nexin 1
0,96	ERMP1	endoplasmic reticulum metallopeptidase 1
0,96	COG2	component of oligomeric golgi complex 2
0,96	BUB1B	BUB1 mitotic checkpoint serine/threonine kinase B
0,96	DEAF1	DEAF1 transcription factor
0,96	CALM3	calmodulin 3 (phosphorylase kinase, delta)
0,96	RAB11FIP2	RAB11 family interacting protein 2 (class I)
0,96	CKS1B	CDC28 protein kinase regulatory subunit 1B
0,96	MAD2L1	MAD2 mitotic arrest deficient-like 1 (yeast)
0,96	CRYGD	crystallin gamma D
0,96	SBK1	SH3 domain binding kinase 1
0,96	SZRD1	SUZ RNA binding domain containing 1
0,96	GNL3; SNORD19B	guanine nucleotide binding protein-like 3 (nucleolar); small nucleolar RNA, C/D box 19B
0,96	FUT9	fucosyltransferase 9 (alpha (1,3) fucosyltransferase)
0,96	TRIM10	tripartite motif containing 10
0,96	ZNF367	zinc finger protein 367
0,96	SSTR2	somatostatin receptor 2
0,96	ZNF322	zinc finger protein 322
0,96	TTC8	tetratricopeptide repeat domain 8
0,96	HSPB1	heat shock 27kDa protein 1
0,96	TSFM	Ts translation elongation factor, mitochondrial
0,96	FKBP1A; MIR6869	FK506 binding protein 1A; microRNA 6869
0,96	TMEM50A	transmembrane protein 50A
0,96	P2RX2	purinergic receptor P2X, ligand gated ion channel, 2

0,96	TMC2	transmembrane channel like 2
0,96	PRKACA	protein kinase, cAMP-dependent, catalytic, alpha
0,96	MRGPRD	MAS-related GPR, member D
0,96	FFAR3	free fatty acid receptor 3
0,96	ATP2A3	ATPase, Ca++ transporting, ubiquitous
0,96	MDGA1	MAM domain containing glycosylphosphatidylinositol anchor 1
0,96	FRMPD2B; FRMPD2	FERM and PDZ domain containing 2B, pseudogene; FERM and PDZ domain containing 2
0,96	ARPP19	cAMP-regulated phosphoprotein 19kDa
0,96	TSTA3	tissue specific transplantation antigen P35B
0,96	ADAMTS9	ADAM metallopeptidase with thrombospondin type 1 motif 9
0,96	WDR5B	WD repeat domain 5B
0,96	CDHR2	cadherin-related family member 2
0,96	LRIT1	leucine-rich repeat, immunoglobulin-like and transmembrane domains 1
0,96	OR8U1; OR8U8	olfactory receptor, family 8, subfamily U, member 1; olfactory receptor, family 8, subfamily U, member 8
0,96	UTP6	UTP6, small subunit (SSU) processome component, homolog (yeast)
0,96	KCNK12	potassium channel, two pore domain subfamily K, member 12
0,96	INO80	INO80 complex subunit
0,96	PODXL2	podocalyxin-like 2
0,96	KPNA1	Transcript Identified by AceView, Entrez Gene ID(s) 3836
0,96	CLIP1	CAP-GLY domain containing linker protein 1
0,96	KCNE2	potassium channel, voltage gated subfamily E regulatory beta subunit 2
0,96	TULP3	tubby like protein 3
0,96	GADD45G	growth arrest and DNA-damage-inducible, gamma
0,96	RP1	retinitis pigmentosa 1 (autosomal dominant)
0,96	EIF3F	Eukaryotic translation initiation factor 3 subunit F [Source:UniProtKB/Swiss-Prot;Acc:O00303]
0,96	TGFBI	transforming growth factor, beta-induced, 68kDa
0,96	NDUFS2	NADH dehydrogenase (ubiquinone) Fe-S protein 2, 49kDa (NADH-coenzyme Q reductase)
0,96	ASCC1	activating signal cointegrator 1 complex subunit 1
0,96	ANKRD16	ankyrin repeat domain 16
0,96	NVL	nuclear VCP-like
0,96	SAFB	scaffold attachment factor B
0,96	NACA2	nascent polypeptide-associated complex alpha subunit 2
0,96	AURKAIP1	aurora kinase A interacting protein 1
0,96	CHRNA2	cholinergic receptor, nicotinic alpha 2
0,96	LACTB2	lactamase, beta 2
0,96	ZMPSTE24	zinc metallopeptidase STE24
0,96	C1orf100	chromosome 1 open reading frame 100
0,96	SYS1-DBNDD2	SYS1-DBNDD2 readthrough (NMD candidate)

0,96	TEX19	testis expressed 19
0,96	LYRM2	LYR motif containing 2
0,96	VAV2	vav 2 guanine nucleotide exchange factor
0,96	sep-08	septin 8
0,96	ASF1A	anti-silencing function 1A histone chaperone
0,96	RNF34	ring finger protein 34, E3 ubiquitin protein ligase
0,96	SYTL2	synaptotagmin-like 2
0,96	ANXA8	annexin A8
0,96	HPGD	hydroxyprostaglandin dehydrogenase 15-(NAD)
0,96	TAOK2	TAO kinase 2
0,96	TOP1	topoisomerase (DNA) I
0,96	CACUL1	CDK2-associated, cullin domain 1
0,96	RASSF5	Ras association (RalGDS/AF-6) domain family member 5
0,96	NSF	N-ethylmaleimide-sensitive factor
0,96	ZNF575	zinc finger protein 575
0,96	PRPF38B	pre-mRNA processing factor 38B
0,96	OPN1LW	opsin 1 (cone pigments), long-wave-sensitive
0,96	TTC37	tetratricopeptide repeat domain 37
0,96	POF1B	premature ovarian failure, 1B
0,96	ARHGAP8	Rho GTPase activating protein 8
0,96	INCENP	inner centromere protein
0,96	TECPR1	tectonin beta-propeller repeat containing 1
0,96	POU3F4	POU class 3 homeobox 4
0,96	INPPL1	inositol polyphosphate phosphatase-like 1
0,96	HTATSF1	HIV-1 Tat specific factor 1
0,96	UNC13D	unc-13 homolog D (C. elegans)
0,96	ATP2A1	ATPase, Ca++ transporting, cardiac muscle, fast twitch 1
0,96	STAU1	staufen double-stranded RNA binding protein 1
0,96	COMMD9	COMM domain containing 9
0,96	HIST1H4I	histone cluster 1, H4i
0,96	PRDX4	peroxiredoxin 4
0,96	UPF1	UPF1 regulator of nonsense transcripts homolog (yeast)
0,96	MRPL39	mitochondrial ribosomal protein L39
0,96	NXNL1	nucleoredoxin-like 1
0,96	FRY	FRY microtubule binding protein
0,96	PRUNE2	prune homolog 2 (Drosophila)
0,96	ACTRT2	actin-related protein T2
0,96	RPL41	Synthetic construct Homo sapiens clone IMAGE:100063377, MGC:190767 ribosomal protein L41 (RPL41) mRNA, encodes complete protein.
0,96	TIGD2	tigger transposable element derived 2
0,96	BPY2C	basic charge, Y-linked, 2C
0,96	PALM	paralemmin
0,96	DUSP15	dual specificity phosphatase 15
0,96	CORIN	corin, serine peptidase
0,96	PABPC1L	poly(A) binding protein, cytoplasmic 1-like
0,96	UFL1	UFM1-specific ligase 1

0,96	STK33	serine/threonine kinase 33
0,96	KRT80	keratin 80, type II
0,96	HTT	huntingtin
0,96	MSTN	myostatin
0,96	ZNF197	zinc finger protein 197
0,96	PBRM1	polybromo 1
0,96	NUF2	NUF2, NDC80 kinetochore complex component
0,96	LYPLA2	lysophospholipase II
0,96	PHOSPHO1	phosphatase, orphan 1
0,96	LOC101060389; TBC1D3D	TBC1 domain family member-like; TBC1 domain family, member 3D [Source:HGNC Symbol;Acc:HGNC:28944]
0,96	PIN4	peptidylprolyl cis/trans isomerase, NIMA-interacting 4
0,96	GADD45GIP1	growth arrest and DNA-damage-inducible, gamma interacting protein 1
0,96	OR14I1	olfactory receptor, family 14, subfamily I, member 1
0,96	OR10A3	olfactory receptor, family 10, subfamily A, member 3
0,96	LPIN1; MIR548S	lipin 1; microRNA 548s
0,96	NPM3	nucleophosmin/nucleoplasmin 3
0,96	IQCF3	Transcript Identified by AceView, Entrez Gene ID(s) 401067
0,96	VANGL1	VANGL planar cell polarity protein 1
0,96	KLHL11	kelch-like family member 11
0,96	C14orf142	chromosome 14 open reading frame 142
0,96	TTC9C	tetratricopeptide repeat domain 9C
0,96	SORBS1	Transcript Identified by AceView, Entrez Gene ID(s) 10580
0,96	NDUFA7	NADH dehydrogenase (ubiquinone) 1 alpha subcomplex, 7, 14.5kDa
0,96	MRPS16	mitochondrial ribosomal protein S16
0,96	PDXDC1	pyridoxal-dependent decarboxylase domain containing 1
0,96	TPGS1	tubulin polyglutamylase complex subunit 1
0,96	ZNF114	zinc finger protein 114
0,96	CEACAM3	carcinoembryonic antigen-related cell adhesion molecule 3
0,96	RAG2	recombination activating gene 2
0,96	MAPK8	mitogen-activated protein kinase 8
0,96	PDSS1	prenyl (decaprenyl) diphosphate synthase, subunit 1
0,96	HSPBAP1	HSPB (heat shock 27kDa) associated protein 1
0,96	CD200; RP11-90K6.1	Transcript Identified by AceView, Entrez Gene ID(s) 4345; novel transcript
0,96	ZNF281	zinc finger protein 281
0,96	FAM198A	family with sequence similarity 198, member A
0,96	ELMOD1	ELMO/CED-12 domain containing 1
0,96	NOSIP	nitric oxide synthase interacting protein
0,96	KCNQ3	potassium channel, voltage gated KQT-like subfamily Q, member 3
0,96	DBX2	developing brain homeobox 2

0,96	PLPP1; RNF138P1	phospholipid phosphatase 1; ring finger protein 138, E3 ubiquitin protein ligase pseudogene 1
0,96	EBP	emopamil binding protein (sterol isomerase)
0,96	ZNF273	zinc finger protein 273
0,96	C1orf137	chromosome 1 open reading frame 137
0,96	XYLT2	xylosyltransferase II
0,96	SRSF12	serine/arginine-rich splicing factor 12
0,96	ENG	endoglin
0,96	ZNF780B	zinc finger protein 780B
0,96	GNAT3	guanine nucleotide binding protein, alpha transducing 3
0,96	OR6A2	olfactory receptor, family 6, subfamily A, member 2
0,96	XPO7	exportin 7
0,96	RALY	RALY heterogeneous nuclear ribonucleoprotein
0,96	GRK6	G protein-coupled receptor kinase 6
0,96	C2orf81	chromosome 2 open reading frame 81
0,96	HIST1H3G	histone cluster 1, H3g
0,96	C19orf48; SNORD88B; SNORD88C	chromosome 19 open reading frame 48; small nucleolar RNA, C/D box 88B; small nucleolar RNA, C/D box 88C
0,96	FOXM1	forkhead box M1
0,96	PRSS27	protease, serine 27
0,96	THAP3	THAP domain containing, apoptosis associated protein 3
0,96	MOBP	myelin-associated oligodendrocyte basic protein
0,96	CFAP36	cilia and flagella associated protein 36
0,96	DDHD1	DDHD domain containing 1
0,96	PGPEP1	pyroglutamyl-peptidase I
0,96	C8orf49	chromosome 8 open reading frame 49
0,96	FTSJ2	FtsJ RNA methyltransferase homolog 2 (E. coli)
0,96	EYA4	EYA transcriptional coactivator and phosphatase 4
0,96	RSRC1	arginine/serine-rich coiled-coil 1
0,96	KRTAP21-2	keratin associated protein 21-2
0,96	UBXN4	UBX domain protein 4
0,96	ZNF70	zinc finger protein 70
0,96	LACTBL1	lactamase, beta-like 1
0,96	EIF4E1B	eukaryotic translation initiation factor 4E family member 1B
0,96	MDGA2	MAM domain containing glycosylphosphatidylinositol anchor 2
0,96	SORCS1	sortilin-related VPS10 domain containing receptor 1
0,96	CACNA1I	calcium channel, voltage-dependent, T type, alpha 1I subunit
0,96	PRDM14	PR domain containing 14
0,96	SLC33A1	solute carrier family 33 (acetyl-CoA transporter), member 1
0,96	RIMS4	regulating synaptic membrane exocytosis 4
0,96	DBF4B	DBF4 zinc finger B
0,96	FAM71F1	family with sequence similarity 71, member F1
0,96	FOXN4	forkhead box N4

0,96	C5orf64	chromosome 5 open reading frame 64
0,96	LRRC8C	leucine rich repeat containing 8 family, member C
0,96	MAP1LC3B	microtubule-associated protein 1 light chain 3 beta
0,96	KHNYN	KH and NYN domain containing
0,96	TALDO1	transaldolase 1
0,96	C16orf91	chromosome 16 open reading frame 91
0,96	LMAN2	lectin, mannose-binding 2
0,96	PRR27	proline rich 27
0,96	ENSA	endosulfine alpha
0,96	MIR22HG	MIR22 host gene
0,96	BTBD3	BTB (POZ) domain containing 3
0,96	MGAT4C	MGAT4 family, member C
0,96	RPL14	Transcript Identified by AceView, Entrez Gene ID(s) 9045
0,96	SYNGAP1; MIR5004	synaptic Ras GTPase activating protein 1; microRNA 5004
0,96	GPX8	glutathione peroxidase 8 (putative)
0,96	HEMK1	HemK methyltransferase family member 1
0,96	SLAIN1	SLAIN motif family member 1
0,96	C19orf45	chromosome 19 open reading frame 45
0,96	GTF2E1	general transcription factor IIE subunit 1
0,96	RSAD1	radical S-adenosyl methionine domain containing 1
0,96	GNG8	guanine nucleotide binding protein (G protein), gamma 8
0,96	OR2AK2	olfactory receptor, family 2, subfamily AK, member 2
0,96	TLDC2	TBC/LysM-associated domain containing 2
0,96	PTGES2	prostaglandin E synthase 2
0,96	ARID5B	AT rich interactive domain 5B (MRF1-like)
0,96	SLC7A9	solute carrier family 7 (amino acid transporter light chain, bo,+ system), member 9
0,96	FAM96A	family with sequence similarity 96, member A
0,96	BTNL9	butyrophilin-like 9
0,96	PRMT3	protein arginine methyltransferase 3
0,96	CKM	creatine kinase, muscle
0,96	FCER1A	Fc fragment of IgE, high affinity I, receptor for; alpha polypeptide
0,96	ZDHHC20	zinc finger, DHHC-type containing 20
0,96	NRXN1	neurexin 1
0,96	OR4D10	olfactory receptor, family 4, subfamily D, member 10
0,96	CD2AP	CD2-associated protein
0,96	PRH2	proline-rich protein HaeIII subfamily 2
0,96	ERLEC1	endoplasmic reticulum lectin 1
0,96	TRMT1L	tRNA methyltransferase 1 like
0,96	SCUBE1	signal peptide, CUB domain, EGF-like 1
0,96	MYO3A	myosin IIIA
0,96	CORO2A	coronin, actin binding protein, 2A
0,96	BRPF1	bromodomain and PHD finger containing 1
0,96	SOD1	superoxide dismutase 1, soluble
0,96	ABCF1	ATP binding cassette subfamily F member 1
0,96	REC114	REC114 meiotic recombination protein

0,96	SRGAP2B	SLIT-ROBO Rho GTPase activating protein 2B
0,96	PGK1	phosphoglycerate kinase 1
0,96	ZBTB9	zinc finger and BTB domain containing 9
0,96	SLC26A8	solute carrier family 26 (anion exchanger), member 8
0,96	SLC25A32	solute carrier family 25 (mitochondrial folate carrier), member 32
0,96	LRRC7	leucine rich repeat containing 7
0,96	LYRM1	LYR motif containing 1
0,96	CD180	CD180 molecule
0,96	ETS2	v-ets avian erythroblastosis virus E26 oncogene homolog 2
0,96	PAH	phenylalanine hydroxylase
0,96	PPM1G	protein phosphatase, Mg2+/Mn2+ dependent, 1G
0,96	PSMG3	proteasome (prosome, macropain) assembly chaperone 3
0,96	ZNF93	zinc finger protein 93
0,96	CEP97	Transcript Identified by AceView, Entrez Gene ID(s) 79598
0,96	GYPB	glycophorin B (MNS blood group)
0,96	TIGAR	TP53 induced glycolysis regulatory phosphatase
0,96	TPM2	tropomyosin 2 (beta)
0,96	SYCP1	synaptonemal complex protein 1
0,96	C12orf42	chromosome 12 open reading frame 42
0,96	TBX19	T-box 19
0,96	SDHA	succinate dehydrogenase complex subunit A, flavoprotein (Fp)
0,96	CIAO1	cytosolic iron-sulfur assembly component 1
0,96	EGLN3	egl-9 family hypoxia-inducible factor 3
0,96	PTPRN	protein tyrosine phosphatase, receptor type, N
0,96	ITGB6	integrin beta 6
0,96	UROS	uroporphyrinogen III synthase
0,96	SCARF2	scavenger receptor class F, member 2
0,96	SETD1B	SET domain containing 1B
0,96	AQP5	aquaporin 5
0,96	LIPC	lipase, hepatic
0,96	SRP14	signal recognition particle 14kDa
0,96	ABHD14A-ACY1	ABHD14A-ACY1 readthrough
0,96	CCZ1B	CCZ1 homolog B, vacuolar protein trafficking and biogenesis associated
0,96	GPX4	glutathione peroxidase 4
0,96	RFESD	Rieske (Fe-S) domain containing
0,96	GABARAPL2	GABA(A) receptor-associated protein like 2
0,96	PELI3	pellino E3 ubiquitin protein ligase family member 3
0,96	GJA8	gap junction protein alpha 8
0,96	UHRF1	ubiquitin-like with PHD and ring finger domains 1
0,96	RPS3A; SNORD73A	ribosomal protein S3A; small nucleolar RNA, C/D box 73A
0,96	MZB1	marginal zone B and B1 cell-specific protein
0,96	HIST1H2AJ	histone cluster 1, H2aj

0,96	PYGO2	pygopus family PHD finger 2
0,96	GPRC6A	G protein-coupled receptor, class C, group 6, member A
0,96	ANKRD62	ankyrin repeat domain 62
0,96	FAM167B	family with sequence similarity 167, member B
0,96	NAP1L6	nucleosome assembly protein 1-like 6
0,96	DTYMK	deoxythymidylate kinase
0,96	SMPX	small muscle protein, X-linked
0,96	UBQLNL	ubiquilin-like
0,96	ZRANB1	zinc finger, RAN-binding domain containing 1
0,96	TFDP3	transcription factor Dp family, member 3
0,96	RPS8; SNORD55; SNORD46; SNORD38A; SNORD38B	ribosomal protein S8; small nucleolar RNA, C/D box 55; small nucleolar RNA, C/D box 46; small nucleolar RNA, C/D box 38A; small nucleolar RNA, C/D box 38B
0,96	TMEM154	transmembrane protein 154
0,96	S100A6	S100 calcium binding protein A6
0,96	UBE2E3	ubiquitin-conjugating enzyme E2E 3
0,96	CHCHD3	coiled-coil-helix-coiled-coil-helix domain containing 3
0,96	TMEM81	transmembrane protein 81
0,96	TGM1	transglutaminase 1
0,96	SEL1L3	sel-1 suppressor of lin-12-like 3 (C. elegans)
0,96	ABI3BP	ABI family, member 3 (NESH) binding protein
0,96	PHF2	PHD finger protein 2
0,96	CACNG8; MIR935	calcium channel, voltage-dependent, gamma subunit 8; microRNA 935
0,96	MED31	mediator complex subunit 31
0,96	ZNF74	zinc finger protein 74
0,96	DDX51	DEAD (Asp-Glu-Ala-Asp) box polypeptide 51
0,96	C10orf126	chromosome 10 open reading frame 126
0,96	INSM1	insulinoma-associated 1
0,96	IGSF21	immunoglobin superfamily, member 21
0,96	TLK1	tousled-like kinase 1
0,96	CHSY3	chondroitin sulfate synthase 3
0,96	OR2V1	olfactory receptor, family 2, subfamily V, member 1
0,96	LEO1	LEO1 homolog, Paf1/RNA polymerase II complex component
0,96	CECR2	cat eye syndrome chromosome region, candidate 2
0,96	RANBP9	RAN binding protein 9
0,96	PPP2R5D	protein phosphatase 2, regulatory subunit B, delta
0,96	ADRA1B	adrenoceptor alpha 1B
0,96	ITGB1	integrin beta 1
0,96	CHRNA4	cholinergic receptor, nicotinic alpha 4
0,96	PHF23	PHD finger protein 23
0,96	TDRKH	tudor and KH domain containing
0,96	B3GALNT2	beta-1,3-N-acetylgalactosaminyltransferase 2
0,96	USP13	ubiquitin specific peptidase 13 (isopeptidase T-3)

0,96	PPP1R16B	Transcript Identified by AceView, Entrez Gene ID(s) 26051
0,96	UGT8	UDP glycosyltransferase 8
0,96	NCAPD2	non-SMC condensin I complex subunit D2
0,96	SLC16A4	solute carrier family 16, member 4
0,96	UPK2	uroplakin 2
0,96	APLN	apelin
0,96	EFNB2	ephrin-B2
0,96	OR1N1	olfactory receptor, family 1, subfamily N, member 1
0,96	SGK3	serum/glucocorticoid regulated kinase family, member 3
0,96	HP	haptoglobin
0,96	HBZ	hemoglobin, zeta
0,96	HSBP1	heat shock factor binding protein 1
0,96	LRIF1	ligand dependent nuclear receptor interacting factor 1
0,96	PRKD1	protein kinase D1
0,96	DACT3	dishevelled-binding antagonist of beta-catenin 3
0,96	ZNF888	zinc finger protein 888
0,96	OR4F4; OR4F17	olfactory receptor, family 4, subfamily F, member 4; olfactory receptor, family 4, subfamily F, member 17
0,96	HRH3	histamine receptor H3
0,96	POLK	polymerase (DNA directed) kappa
0,96	HTRA3	Transcript Identified by AceView, Entrez Gene ID(s) 94031
0,96	ZNF654	zinc finger protein 654
0,96	B3GLCT	beta 3-glucosyltransferase
0,96	OSR1	odd-skipped related transciption factor 1
0,96	L3MBTL3	l(3)mbt-like 3 (Drosophila)
0,96	IL4R	interleukin 4 receptor
0,96	RNF208	ring finger protein 208
0,96	SURF4	surfeit 4
0,96	KRTAP9-2	keratin associated protein 9-2
0,96	ZNF658	zinc finger protein 658
0,96	HMBS	hydroxymethylbilane synthase
0,96	NCKIPSD	NCK interacting protein with SH3 domain
0,96	HIST2H2AB	histone cluster 2, H2ab
0,96	DUSP16	dual specificity phosphatase 16
0,96	MCM4	minichromosome maintenance complex component 4
0,96	LGALS12	lectin, galactoside-binding, soluble, 12
0,96	C4orf3	chromosome 4 open reading frame 3

0,96	LOC389831; TCONS_00030034; TCONS_00030035; AC011043.1	uncharacterized LOC389831; Salzman2013 ANNOTATED, CDS, coding, OVCODE, OVEXON, UTR3 best transcript NM_001242480; Salzman2013 ANNOTATED, CDS, coding, OVCODE, OVEXON, UTR3, UTR5 best transcript NM_001242480; Salzman2013 ANNOTATED, ncRNA, OVEXON best transcript TCONS_00030034; Salzman2013 ANNOTATED, ncRNA, OVCODE, OVEXON best transcript TCONS_00030034; Salzman2013 ALT_ACCEPTOR, ncRNA, OVCODE, OVEXON, upstream_start best transcript TCONS_00030034; Salzman2013 ALT_DONOR, downstream_end, ncRNA, OVEXON best transcript TCONS_00030035; Salzman2013 ALT_DONOR, coding, intronic, OVEXON, UTR5 best transcript NM_001242480; uncharacterized protein LOC389831 [Source:RefSeq peptide;Acc:NP_001229409]
0,96	SLC7A4	solute carrier family 7, member 4
0,96	NUP188	nucleoporin 188kDa
0,96	MED8	mediator complex subunit 8
0,96	ESAM	endothelial cell adhesion molecule
0,96	TGM4	transglutaminase 4
0,96	CD69	CD69 molecule
0,96	NUP155	nucleoporin 155kDa
0,96	NDUFA10	NADH dehydrogenase (ubiquinone) 1 alpha subcomplex, 10, 42kDa
0,96	DNAJC16	DnaJ (Hsp40) homolog, subfamily C, member 16
0,96	ALPK1	alpha kinase 1
0,96	ARMT1	acidic residue methyltransferase 1
0,96	DMBT1	deleted in malignant brain tumors 1
0,96	EHBP1	EH domain binding protein 1
0,96	OR8I2	olfactory receptor, family 8, subfamily I, member 2
0,96	KAZALD1	Kazal-type serine peptidase inhibitor domain 1
0,96	CHMP3	charged multivesicular body protein 3
0,96	UHRF2	ubiquitin-like with PHD and ring finger domains 2, E3 ubiquitin protein ligase
0,96	LCMT1	leucine carboxyl methyltransferase 1
0,96	LOC151121; AC079586.1	uncharacterized LOC151121; novel transcript
0,96	YTHDC2	YTH domain containing 2
0,96	CDK5R1	cyclin-dependent kinase 5, regulatory subunit 1 (p35)
0,96	ATP1B3	ATPase, Na+/K+ transporting, beta 3 polypeptide
0,96	PHKB	Transcript Identified by AceView, Entrez Gene ID(s) 5257
0,96	C6orf58	chromosome 6 open reading frame 58
0,96	TM9SF1	transmembrane 9 superfamily member 1
0,96	TRAK1	trafficking protein, kinesin binding 1
0,96	OR52I2	olfactory receptor, family 52, subfamily I, member 2
0,96	PET100	PET100 homolog
0,96	UBXN7	UBX domain protein 7

0,96	GPIHBP1	glycosylphosphatidylinositol anchored high density lipoprotein binding protein 1
0,96	C8G	complement component 8, gamma polypeptide
0,96	SF1	Memczak2013 ALT_ACCEPTOR, ALT_DONOR, coding, INTERNAL, intronic best transcript NM_201998
0,96	PKN1	protein kinase N1
0,96	ONECUT2	one cut homeobox 2
0,96	TMEM95	transmembrane protein 95
0,96	HIST1H4H	histone cluster 1, H4h
0,96	ZNF852	zinc finger protein 852
0,96	SKA2	spindle and kinetochore associated complex subunit 2
0,96	STYK1	serine/threonine/tyrosine kinase 1
0,96	CRB1	crumbs family member 1, photoreceptor morphogenesis associated
0,96	CAMTA2	calmodulin binding transcription activator 2
0,96	MRPS15	mitochondrial ribosomal protein S15
0,96	MAGT1	magnesium transporter 1
0,96	SFN	stratifin
0,96	KDM3B	lysine (K)-specific demethylase 3B
0,96	TRIM69	tripartite motif containing 69
0,96	SNX11	sorting nexin 11
0,96	LIMCH1	LIM and calponin homology domains 1
0,96	GINS2	GINS complex subunit 2 (Psf2 homolog)
0,96	RREB1	ras responsive element binding protein 1
0,96	CETN2	centrin 2
0,96	CNOT11	CCR4-NOT transcription complex subunit 11
0,96	TOR1B	torsin family 1, member B (torsin B)
0,96	SRD5A3	steroid 5 alpha-reductase 3
0,96	CCDC159	coiled-coil domain containing 159
0,96	TSPAN6	tetraspanin 6
0,96	LTA	lymphotoxin alpha
0,96	RPS6KA1	ribosomal protein S6 kinase, 90kDa, polypeptide 1
0,96	CMC2	C-x(9)-C motif containing 2
0,96	GABRA3	gamma-aminobutyric acid (GABA) A receptor, alpha 3
0,96	AGTRAP	angiotensin II receptor-associated protein
0,96	ZNRF2	zinc and ring finger 2, E3 ubiquitin protein ligase
0,96	NTNG2	netrin G2
0,96	FLCN	folliculin
0,96	AAGAB	alpha- and gamma-adaptin binding protein
0,96	HLA-DQA2	major histocompatibility complex, class II, DQ alpha 2
0,96	MORF4L1	mortality factor 4 like 1
0,96	SESN1	sestrin 1
0,96	KIAA1143	KIAA1143

0,96	IGLL5; MIR5571; IGLC1; IGLC2; IGLV2-5; IGLV3-1; IGLJ1	immunoglobulin lambda-like polypeptide 5; microRNA 5571; immunoglobulin lambda constant 1 (Mcg marker); immunoglobulin lambda constant 2 (Kern-Oz-marker); immunoglobulin lambda variable 2-5 (pseudogene); immunoglobulin lambda variable 3-1; immunoglobulin lambda joining 1
0,96	VWF	von Willebrand factor
0,96	FAM173B	family with sequence similarity 173, member B
0,96	ADK; MRPL35P3	adenosine kinase; mitochondrial ribosomal protein L35 pseudogene 3
0,96	TMEFF2	transmembrane protein with EGF-like and two follistatin-like domains 2
0,96	KLHL36	kelch-like family member 36
0,96	ATP5O	ATP synthase, H+ transporting, mitochondrial F1 complex, O subunit
0,96	QSER1	glutamine and serine rich 1
0,96	SAP18	Sin3A associated protein 18kDa
0,96	PIP5K1C	Zhang2013 ALT_ACCEPTOR, ALT_DONOR, coding, INTERNAL, intronic best transcript NM_012398
0,96	OAS1	2-5-oligoadenylate synthetase 1
0,96	TTF2	transcription termination factor, RNA polymerase II
0,96	ADGRA2	adhesion G protein-coupled receptor A2
0,96	COMMD3-BMI1; BMI1; COMMD3	COMMD3-BMI1 readthrough; BMI1 proto-oncogene, polycomb ring finger; COMM domain containing 3
0,96	FBXO42	F-box protein 42
0,96	TRIM4	tripartite motif containing 4
0,96	CD80	CD80 molecule
0,96	CMTM2	CKLF-like MARVEL transmembrane domain containing 2
0,96	LRRC42	leucine rich repeat containing 42
0,96	ZNF705B	zinc finger protein 705B
0,96	OSER1	oxidative stress responsive serine-rich 1
0,96	BCL2L2-PABPN1	BCL2L2-PABPN1 readthrough
0,96	SOWAHC	sosondowah ankyrin repeat domain family member C
0,96	APBA1	amyloid beta (A4) precursor protein-binding, family A, member 1
0,96	EXO1	exonuclease 1
0,96	SURF4	surfeit 4
0,96	ZNF141	zinc finger protein 141
0,96	POLE	Transcript Identified by AceView, Entrez Gene ID(s) 5426
0,96	KIAA1841	KIAA1841
0,96	OGDHL	oxoglutarate dehydrogenase-like
0,96	CRADD	CASP2 and RIPK1 domain containing adaptor with death domain
0,96	SPATA7	spermatogenesis associated 7
0,96	NGB	neuroglobin
0,96	C4orf48	chromosome 4 open reading frame 48
0,96	RTTN	rotatin

0,96	ZCCHC6	zinc finger, CCHC domain containing 6
0,96	KNSTRN	kinetochore-localized astrin/SPAG5 binding protein
0,96	CCDC50	coiled-coil domain containing 50
0,96	WDR13	WD repeat domain 13
0,96	RASSF7	Ras association (RalGDS/AF-6) domain family (N-terminal) member 7
0,96	PTPRT	protein tyrosine phosphatase, receptor type, T
0,96	FBXO39	F-box protein 39
0,96	TRPM7	transient receptor potential cation channel, subfamily M, member 7
0,96	CYP27C1	cytochrome P450, family 27, subfamily C, polypeptide 1
0,96	EIF2B2	eukaryotic translation initiation factor 2B, subunit 2 beta, 39kDa
0,96	WWC2	WW and C2 domain containing 2
0,96	TGM4	transglutaminase 4
0,96	NBPF15	neuroblastoma breakpoint family, member 15
0,96	SLC1A7	solute carrier family 1 (glutamate transporter), member 7
0,96	TPH1	tryptophan hydroxylase 1
0,96	LOC100130451; AC079610.2; RP11-105N14.2	uncharacterized LOC100130451; novel transcript (LOC100130451); novel transcript
0,96	PRADC1	protease-associated domain containing 1
0,96	TRHDE	thyrotropin-releasing hormone degrading enzyme
0,96	ATG2A	autophagy related 2A
0,96	DHFR; DHFRP1	dihydrofolate reductase; dihydrofolate reductase pseudogene 1
0,96	DNM1L	dynamin 1-like
0,96	ZAK; pk	sterile alpha motif and leucine zipper containing kinase AZK; Mitogen-activated protein kinase kinase kinase MLT [Source:UniProtKB/Swiss-Prot;Acc:Q9NYL2]; Salzman2013 ANNOTATED, CDS, coding, INTERNAL, OVCODE, OVERLAPTX, OVEXON best transcript NM_133646; Salzman2013 ANNOTATED, CDS, coding, OVCODE, OVERLAPTX, OVEXON, UTR3 best transcript NM_016653; Salzman2013 ANNOTATED, CDS, coding, OVCODE, OVERLAPTX, OVEXON, UTR3 best transcript NM_133646; Salzman2013 ANNOTATED, CDS, coding, INTERNAL, OVCODE, OVERLAPTX, OVEXON best transcript NM_016653; Transcript Identified by AceView, Entrez Gene ID(s) 51776
0,96	DHX38	DEAH (Asp-Glu-Ala-His) box polypeptide 38
0,96	BLMH	bleomycin hydrolase
0,96	WISP1	WNT1 inducible signaling pathway protein 1
0,96	MXD3	MAX dimerization protein 3
0,96	RCBTB1	regulator of chromosome condensation (RCC1) and BTB (POZ) domain containing protein 1
0,96	APEH	acylaminoacyl-peptide hydrolase

0,96	TLE1	transducin-like enhancer of split 1 (E(sp1) homolog, Drosophila)
0,96	HAX1	HCLS1 associated protein X-1
0,96	TXN2	thioredoxin 2
0,96	LRIG1	
0,96	OR1C1	olfactory receptor, family 1, subfamily C, member 1
0,96	FAM221B	family with sequence similarity 221, member B
0,96	ROM1	retinal outer segment membrane protein 1
0,96	TCP11L1	t-complex 11, testis-specific-like 1
0,96	CHST4	carbohydrate (N-acetylglucosamine 6-O) sulfotransferase 4
0,96	SCN9A	sodium channel, voltage gated, type IX alpha subunit
0,96	SLIT2	Jeck2013 ALT_ACCEPTOR, ALT_DONOR, coding, INTERNAL, intronic best transcript NM_004787
0,96	ZNF212	zinc finger protein 212
0,96	AKAP1	A kinase (PRKA) anchor protein 1
0,96	PLEKHA2	pleckstrin homology domain containing, family A (phosphoinositide binding specific) member 2
0,96	CTNNA3	catenin (cadherin-associated protein), alpha 3
0,96	DFNB59	deafness, autosomal recessive 59
0,96	CYB5A	cytochrome b5 type A (microsomal)
0,96	RASSF10	Ras association (RalGDS/AF-6) domain family (N-terminal) member 10
0,96	RPS25	ribosomal protein S25
0,96	MGARP	mitochondria localized glutamic acid rich protein
0,96	ARHGEF17	Rho guanine nucleotide exchange factor 17
0,96	EEF1B2; SNORA41	eukaryotic translation elongation factor 1 beta 2; small nucleolar RNA, H/ACA box 41
0,96	SNORD49A; SNORD65; LRRC75A-AS1	small nucleolar RNA, C/D box 49A; small nucleolar RNA, C/D box 65; LRRC75A antisense RNA 1
0,96	LINC01359	long intergenic non-protein coding RNA 1359
0,96	ZNF623	zinc finger protein 623
0,96	OR8B4	olfactory receptor, family 8, subfamily B, member 4 (gene/pseudogene)
0,96	NUDT12	nudix hydrolase 12
0,96	NUP54	nucleoporin 54kDa
0,96	CDKL1	cyclin-dependent kinase-like 1 (CDC2-related kinase)
0,96	NUDT5	nudix hydrolase 5
0,96	OR4K2	olfactory receptor, family 4, subfamily K, member 2
0,96	ADAMTS13	ADAM metallopeptidase with thrombospondin type 1 motif 13
0,96	PXDNL	peroxidasin like
0,96	CACNG6	calcium channel, voltage-dependent, gamma subunit 6
0,96	AASDHPPT	aminoadipate-semialdehyde dehydrogenase-phosphopantetheinyl transferase
0,96	NRTN	neurturin
0,96	PTN	pleiotrophin
0,96	ACSM3	acyl-CoA synthetase medium-chain family member 3
0,96	PPP4R3B	protein phosphatase 4, regulatory subunit 3B

0,96	CDCA7L	cell division cycle associated 7-like
0,96	RPL10L	ribosomal protein L10-like
0,96	LDB2	LIM domain binding 2
0,96	NIF3L1	NIF3 NGG1 interacting factor 3-like 1
0,96	COX7A1	cytochrome c oxidase subunit VIIa polypeptide 1 (muscle)
0,96	NDUFB1	NADH dehydrogenase (ubiquinone) 1 beta subcomplex, 1, 7kDa
0,96	SNRPA1	small nuclear ribonucleoprotein polypeptide A
0,96	LMNTD2	lamin tail domain containing 2
0,96	KRT6C	keratin 6C, type II
0,96	TNS3	tensin 3
0,96	SPINK9	serine peptidase inhibitor, Kazal type 9
0,96	ALDH3A2	aldehyde dehydrogenase 3 family, member A2
0,96	HNRNPCL1	heterogeneous nuclear ribonucleoprotein C-like 1
0,96	ILF2	interleukin enhancer binding factor 2
0,96	ALDH1L2	aldehyde dehydrogenase 1 family, member L2
0,96	PRAMEF4	PRAME family member 4
0,96	ZNF541	zinc finger protein 541
0,96	GMNN	geminin, DNA replication inhibitor
0,96	SOX3	SRY box 3
0,96	WWTR1	WW domain containing transcription regulator 1
0,96	IGFBP3	insulin like growth factor binding protein 3
0,96	TAF7L	TATA box binding protein associated factor 7 like
0,96	BCL2L11	BCL2-like 11 (apoptosis facilitator)
0,96	OR2T8	olfactory receptor, family 2, subfamily T, member 8
0,96	EIF3F	eukaryotic translation initiation factor 3, subunit F
0,96	C20orf27	chromosome 20 open reading frame 27
0,96	SULF1	sulfatase 1
0,96	C16orf87	chromosome 16 open reading frame 87
0,96	UBE2S; UBE2SP1	ubiquitin-conjugating enzyme E2S; ubiquitin conjugating enzyme E2S pseudogene 1
0,96	ALDH3B1	aldehyde dehydrogenase 3 family, member B1
0,96	ALAS2	5-aminolevulinate synthase 2
0,96	PRKAG1	protein kinase, AMP-activated, gamma 1 non-catalytic subunit
0,96	TMEM108	transmembrane protein 108
0,96	SLC4A3	solute carrier family 4 (anion exchanger), member 3
0,96	OR6C3	olfactory receptor, family 6, subfamily C, member 3
0,96	CDH6	cadherin 6, type 2, K-cadherin (fetal kidney)
0,96	SBSN	suprabasin
0,96	VAMP4	vesicle associated membrane protein 4
0,96	SNX13	sorting nexin 13
0,96	OCLM	Transcript Identified by AceView, Entrez Gene ID(s) 10896
0,96	BMPR1A	bone morphogenetic protein receptor type IA
0,96	SURF2	surfeit 2
0,96	OR1L3	olfactory receptor, family 1, subfamily L, member 3
0,96	OR4C6	olfactory receptor, family 4, subfamily C, member 6

0,96	RPS29; RPL32P29	ribosomal protein S29; ribosomal protein L32 pseudogene 29
0,96	T	T brachyury transcription factor
0,96	SH2B2	SH2B adaptor protein 2
0,96	SPINK13	serine peptidase inhibitor, Kazal type 13 (putative)
0,96	KLRF1	killer cell lectin-like receptor subfamily F, member 1
0,96	SMIM12	small integral membrane protein 12
0,96	CROCC	Zhang2013 ALT_ACCEPTOR, ALT_DONOR, coding, INTERNAL, intronic best transcript NM_014675
0,96	PPM1F	protein phosphatase, Mg2+/Mn2+ dependent, 1F
0,96	TBRG4; SNORA5B	transforming growth factor beta regulator 4; small nucleolar RNA, H/ACA box 5B
0,96	COMMD7	COMM domain containing 7
0,96	MAPK3	mitogen-activated protein kinase 3
0,96	OR13J1	olfactory receptor, family 13, subfamily J, member 1
0,96	PAGE3	P antigen family, member 3 (prostate associated)
0,96	ACSS3	acyl-CoA synthetase short-chain family member 3
0,96	SH3YL1	SH3 and SYLF domain containing 1
0,96	HERC3	HECT and RLD domain containing E3 ubiquitin protein ligase 3
0,96	ZSCAN9	zinc finger and SCAN domain containing 9
0,96	THSD1	thrombospondin type 1 domain containing 1
0,96	RHOXF1	Rhox homeobox family, member 1
0,96	LYZL2	lysozyme-like 2
0,96	SYN2	synapsin II
0,96	FLJ38576; RP11-173M11.6	uncharacterized LOC651430; uncharacterized LOC651430 [Source:EntrezGene;Acc:651430]; novel transcript
0,96	AIG1	androgen-induced 1
0,96	EIF3E	eukaryotic translation initiation factor 3, subunit E
0,96	CD59	CD59 molecule, complement regulatory protein
0,96	ACRBP	acrosin binding protein
0,96	SBK3	SH3 domain binding kinase family, member 3
0,96	FAM129B	family with sequence similarity 129, member B
0,96	PTGR2	prostaglandin reductase 2
0,96	SLC3A1	solute carrier family 3 (amino acid transporter heavy chain), member 1
0,96	LOC283788; AL592183.1	FSHD region gene 1 pseudogene
0,96	DPY30	dpy-30 histone methyltransferase complex regulatory subunit
0,96	TAF1	TAF1 RNA polymerase II, TATA box binding protein (TBP)-associated factor, 250kDa
0,96	C14orf159	chromosome 14 open reading frame 159
0,96	FAM207A	family with sequence similarity 207, member A
0,96	EPHA3	EPH receptor A3
0,96	CCDC172	coiled-coil domain containing 172
0,95	ZNF436	zinc finger protein 436
0,95	ISL2	ISL LIM homeobox 2

0,95	FAM81B	family with sequence similarity 81, member B
0,95	TFDP2	transcription factor Dp-2 (E2F dimerization partner 2)
0,95	HPS6	Hermansky-Pudlak syndrome 6
0,95	ZNF417	zinc finger protein 417
0,95	sep-15	15 kDa selenoprotein; Salzman2013 ANNOTATED, CDS, coding, INTERNAL, OVCODE, OVEXON best transcript NM_004261; Transcript Identified by AceView, Entrez Gene ID(s) 9403, RefSeq ID(s) NM_203341; Transcript Identified by AceView, Entrez Gene ID(s) 9403, RefSeq ID(s) NM_004261; 15 kDa selenoprotein [Source:EntrezGene;Acc:9403]
0,95	CDAN1	codanin 1
0,95	SNTB1	syntrophin, beta 1 (dystrophin-associated protein A1, 59kDa, basic component 1)
0,95	P4HB	prolyl 4-hydroxylase, beta polypeptide
0,95	PQLC2L	PQ loop repeat containing 2-like
0,95	MYDGF	myeloid-derived growth factor
0,95	SAMD13	sterile alpha motif domain containing 13
0,95	DSN1	DSN1 homolog, MIS12 kinetochore complex component
0,95	RAB15	RAB15, member RAS oncogene family
0,95	TYW1B	tRNA-yW synthesizing protein 1 homolog B (S. cerevisiae)
0,95	RPL39; SNORA69	ribosomal protein L39; small nucleolar RNA, H/ACA box 69
0,95	DESI2	desumoylating isopeptidase 2
0,95	SNCA	synuclein alpha
0,95	TBC1D20	TBC1 domain family, member 20
0,95	VARS	valyl-tRNA synthetase
0,95	LOC646938	TBC1 domain family, member 2B pseudogene; Transcript Identified by AceView, Entrez Gene ID(s) 646938
0,95	GSDMD	gasdermin D
0,95	USP47	ubiquitin specific peptidase 47
0,95	MNDA	myeloid cell nuclear differentiation antigen
0,95	OGN	osteoglycin
0,95	ZNF786	zinc finger protein 786
0,95	KLHL9	kelch-like family member 9
0,95	EHD4	EH domain containing 4
0,95	BLVRA	biliverdin reductase A
0,95	COA4	cytochrome c oxidase assembly factor 4 homolog
0,95	PSMD8	proteasome 26S subunit, non-ATPase 8
0,95	IL17F	interleukin 17F
0,95	C20orf24	chromosome 20 open reading frame 24
0,95	PDIA6	protein disulfide isomerase family A, member 6
0,95	EPB41	erythrocyte membrane protein band 4.1
0,95	ZNF141	Transcript Identified by AceView, Entrez Gene ID(s) 100288237; 7700
0,95	CAMK4	calcium/calmodulin-dependent protein kinase IV
0,95	GJD4	gap junction protein delta 4

0,95	C2orf15	chromosome 2 open reading frame 15
0,95	DVL2	dishevelled segment polarity protein 2
0,95	NPEPPS	aminopeptidase puromycin sensitive
0,95	CISH	cytokine inducible SH2-containing protein
0,95	PRMT9	protein arginine methyltransferase 9
0,95	HRH4	histamine receptor H4
0,95	IMP3	IMP3, U3 small nucleolar ribonucleoprotein
0,95	MRGBP	MRG/MORF4L binding protein
0,95	ZC3H6	zinc finger CCCH-type containing 6
0,95	PLAG1	pleiomorphic adenoma gene 1
0,95	GSTT2	glutathione S-transferase theta 2 (gene/pseudogene)
0,95	LEMD2	LEM domain containing 2
0,95	PP14571; AC110619.2	uncharacterized LOC100130449; novel transcript, antisense to GPC1; Transcript Identified by AceView, Entrez Gene ID(s) 100130449; uncharacterized LOC100130449 [Source:EntrezGene;Acc:100130449]
0,95	C5orf56	chromosome 5 open reading frame 56
0,95	IL3RA	Jeck2013 ANTISENSE, CDS, coding, INTERNAL, intronic, OVCODE, OVEXON best transcript NM_002183
0,95	SYNPO	synaptopodin
0,95	OR11G2	olfactory receptor, family 11, subfamily G, member 2
0,95	LYZL6	lysozyme-like 6
0,95	GOPC	golgi-associated PDZ and coiled-coil motif containing
0,95	FPR1	formyl peptide receptor 1
0,95	NAT9	N-acetyltransferase 9 (GCN5-related, putative)
0,95	AGPAT5	1-acylglycerol-3-phosphate O-acyltransferase 5
0,95	PHF1	PHD finger protein 1
0,95	STATH	statherin
0,95	SPR	sepiapterin reductase (7,8-dihydrobiopterin:NADP+ oxidoreductase)
0,95	SHCBP1	SHC SH2-domain binding protein 1
0,95	GAB4	GRB2-associated binding protein family, member 4
0,95	BCL2L14	BCL2-like 14 (apoptosis facilitator)
0,95	UBE2U	ubiquitin-conjugating enzyme E2U (putative)
0,95	GPS1	G protein pathway suppressor 1
0,95	HIST1H2BD	histone cluster 1, H2bd
0,95	CA4	carbonic anhydrase IV
0,95	USH2A	Usher syndrome 2A (autosomal recessive, mild)
0,95	CFC1	cripto, FRL-1, cryptic family 1
0,95	RNF169	ring finger protein 169
0,95	ING3	inhibitor of growth family member 3
0,95	ARL5A	ADP-ribosylation factor like GTPase 5A
0,95	TMEM129	transmembrane protein 129, E3 ubiquitin protein ligase
0,95	C1orf116	chromosome 1 open reading frame 116
0,95	USP17L17	ubiquitin specific peptidase 17-like family member 17
0,95	TWF1	twinfilin actin binding protein 1

0,95	RANGAP1	Ran GTPase activating protein 1
0,95	OTC	ornithine carbamoyltransferase
0,95	ABCB6	ATP binding cassette subfamily B member 6 (Langereis blood group)
0,95	POLR3A	polymerase (RNA) III (DNA directed) polypeptide A, 155kDa
0,95	PTPRO	Transcript Identified by AceView, Entrez Gene ID(s) 5800
0,95	USP13	Memczak2013 ANTISENSE, coding, INTERNAL, intronic best transcript NM_003940
0,95	PLCD1	phospholipase C, delta 1
0,95	PNMA5	paraneoplastic Ma antigen family member 5
0,95	ZNF878	zinc finger protein 878
0,95	CD86	CD86 molecule
0,95	ZNF625-ZNF20; ZNF625	ZNF625-ZNF20 readthrough (NMD candidate); zinc finger protein 625
0,95	AKTIP	AKT interacting protein
0,95	FYTTD1	forty-two-three domain containing 1
0,95	LCN15	lipocalin 15
0,95	CDCA2	cell division cycle associated 2
0,95	ZNF615	zinc finger protein 615
0,95	OXER1	oxoeicosanoid (OXE) receptor 1
0,95	EFNA5	ephrin-A5
0,95	KRT4	keratin 4, type II
0,95	GPR119	G protein-coupled receptor 119
0,95	GLOD5	glyoxalase domain containing 5
0,95	WBP1L	WW domain binding protein 1-like
0,95	PPP6R1	protein phosphatase 6, regulatory subunit 1
0,95	RAD1	RAD1 checkpoint DNA exonuclease
0,95	SMC6	structural maintenance of chromosomes 6
0,95	LHCGR	luteinizing hormone/choriogonadotropin receptor
0,95	LCE1F	late cornified envelope 1F
0,95	GPR33	G protein-coupled receptor 33 (gene/pseudogene)
0,95	AATK	apoptosis-associated tyrosine kinase
0,95	RBMY1J; RBMY1F	RNA binding motif protein, Y-linked, family 1, member J; RNA binding motif protein, Y-linked, family 1, member F
0,95	RUNX3	runt-related transcription factor 3
0,95	PEX1	peroxisomal biogenesis factor 1
0,95	RNF41	ring finger protein 41, E3 ubiquitin protein ligase
0,95	TMEM186	transmembrane protein 186
0,95	OR8A1	olfactory receptor, family 8, subfamily A, member 1
0,95	LASP1	LIM and SH3 protein 1
0,95	TATDN3	TatD DNase domain containing 3
0,95	PCMTD1	protein-L-isoaspartate (D-aspartate) O-methyltransferase domain containing 1
0,95	CFL1	cofilin 1 (non-muscle)
0,95	FICD	FIC domain containing
0,95	ARHGAP11B	Rho GTPase activating protein 11B
0,95	HAND2-AS1	HAND2 antisense RNA 1 (head to head)

0,95	HMGB4	high mobility group box 4
0,95	CARKD	carbohydrate kinase domain containing
0,95	KCTD9	potassium channel tetramerization domain containing 9
0,95	PON2	paraoxonase 2
0,95	FLYWCH2	FLYWCH family member 2
0,95	ERP29	endoplasmic reticulum protein 29
0,95	RRM1	ribonucleotide reductase M1
0,95	SLC25A52	solute carrier family 25, member 52
0,95	CLCC1	chloride channel CLIC-like 1
0,95	RPS4Y1	ribosomal protein S4, Y-linked 1
0,95	TRMT12	tRNA methyltransferase 12 homolog (S. cerevisiae)
0,95	WWOX	WW domain containing oxidoreductase
0,95	MARCH1	membrane associated ring finger 1
0,95	MAN2C1	mannosidase, alpha, class 2C, member 1
0,95	NPHP3-ACAD11	NPHP3-ACAD11 readthrough (NMD candidate)
0,95	OR1Q1	olfactory receptor, family 1, subfamily Q, member 1
0,95	NKAIN2	Na+/K+ transporting ATPase interacting 2
0,95	CDK5RAP3	CDK5 regulatory subunit associated protein 3
0,95	TRIM48	tripartite motif containing 48
0,95	KDM5B	lysine (K)-specific demethylase 5B
0,95	POLR2H	polymerase (RNA) II (DNA directed) polypeptide H
0,95	KIF4B	kinesin family member 4B
0,95	CYP39A1	cytochrome P450, family 39, subfamily A, polypeptide 1
0,95	CAPN15; MIR5587	calpain 15; microRNA 5587
0,95	SCYL3	SCY1-like, kinase-like 3
0,95	GPR152	G protein-coupled receptor 152
0,95	SMIM24	small integral membrane protein 24
0,95	RPA2	replication protein A2
0,95	MICU1	mitochondrial calcium uptake 1
0,95	C17orf102	chromosome 17 open reading frame 102
0,95	SP3	Sp3 transcription factor
0,95	FAM65B	family with sequence similarity 65, member B
0,95	CCT6B	chaperonin containing TCP1, subunit 6B (zeta 2)
0,95	TIMM10B	translocase of inner mitochondrial membrane 10 homolog B (yeast)
0,95	HSFX1	heat shock transcription factor family, X-linked 1
0,95	OC90	otoconin 90
0,95	REG3G	regenerating islet-derived 3 gamma
0,95	ACTN1; HMGN1P3	actinin, alpha 1; high mobility group nucleosome binding domain 1 pseudogene 3
0,95	GHRHR	growth hormone releasing hormone receptor
0,95	HLA-C	major histocompatibility complex, class I, C
0,95	PPM1H	protein phosphatase, Mg2+/Mn2+ dependent, 1H
0,95	PLXDC1	plexin domain containing 1
0,95	KHSRP; MIR3940	KH-type splicing regulatory protein; microRNA 3940
0,95	CNOT6	CCR4-NOT transcription complex subunit 6

0,95	NPSR1	neuropeptide S receptor 1
0,95	FAM84B	family with sequence similarity 84, member B
0,95	OR4K15	olfactory receptor, family 4, subfamily K, member 15
0,95	VPS9D1	VPS9 domain containing 1
0,95	UCN3	urocortin 3
0,95	HAS3	hyaluronan synthase 3
0,95	C7orf13	chromosome 7 open reading frame 13
0,95	IMMT	inner membrane protein, mitochondrial
0,95	LCN10	lipocalin 10
0,95	SHC1	SHC (Src homology 2 domain containing) transforming protein 1
0,95	SOX21	SRY box 21
0,95	DOC2B	double C2-like domains, beta
0,95	POP7	POP7 homolog, ribonuclease P/MRP subunit
0,95	RNASEH2C	ribonuclease H2, subunit C
0,95	FNBP1L	formin binding protein 1-like
0,95	ING3	Transcript Identified by AceView, Entrez Gene ID(s) 54556
0,95	TBCA	tubulin folding cofactor A
0,95	NUPR2	nuclear protein 2, transcriptional regulator
0,95	GIT2	G protein-coupled receptor kinase interacting ArfGAP 2
0,95	PARP11	poly(ADP-ribose) polymerase family member 11
0,95	DHRS9	dehydrogenase/reductase (SDR family) member 9
0,95	TP53BP1	tumor protein p53 binding protein 1
0,95	CMIP	Memczak2013 ANTISENSE, coding, INTERNAL, intronic best transcript NM_198390
0,95	FANCC	Fanconi anemia complementation group C
0,95	RP11-54C4.3; LOC100652758	uncharacterized protein LOC100652758 [Source:RefSeq peptide;Acc:NP_001265011]; uncharacterized LOC100652758; novel transcript
0,95	DDT	D-dopachrome tautomerase
0,95	PTPRS	protein tyrosine phosphatase, receptor type, S
0,95	NUP50	nucleoporin 50kDa
0,95	BLM	Bloom syndrome, RecQ helicase-like
0,95	STX10	syntaxin 10
0,95	ITGAV	integrin alpha V
0,95	RMDN1	regulator of microtubule dynamics 1
0,95	BEND3	BEN domain containing 3
0,95	RCC2	regulator of chromosome condensation 2
0,95	MRPS34	mitochondrial ribosomal protein S34
0,95	ZBTB43	zinc finger and BTB domain containing 43
0,95	SLC7A3	solute carrier family 7 (cationic amino acid transporter, y+ system), member 3
0,95	TEX30	testis expressed 30
0,95	ASXL1	additional sex combs like transcriptional regulator 1
0,95	SLC41A2	solute carrier family 41 (magnesium transporter), member 2
0,95	CLCA1	chloride channel accessory 1
0,95	MRPS25	mitochondrial ribosomal protein S25

0,95	PSMC3	proteasome 26S subunit, ATPase 3
0,95	PRR9	proline rich 9
0,95	WHSC1L1	Wolf-Hirschhorn syndrome candidate 1-like 1
0,95	CNBD2	cyclic nucleotide binding domain containing 2
0,95	SRPK1	SRSF protein kinase 1
0,95	SEMA4B	sema domain, immunoglobulin domain (Ig), transmembrane domain (TM) and short cytoplasmic domain, (semaphorin) 4B
0,95	DIDO1	death inducer-obliterator 1
0,95	RAB40A	RAB40A, member RAS oncogene family
0,95	AAMP	angio-associated migratory cell protein
0,95	PPY	pancreatic polypeptide
0,95	SH3PXD2B	SH3 and PX domains 2B
0,95	IFT20	intraflagellar transport 20
0,95	LGR6	leucine-rich repeat containing G protein-coupled receptor 6
0,95	WNT3A	wingless-type MMTV integration site family, member 3A
0,95	PAX3	paired box 3
0,95	HAPLN2	hyaluronan and proteoglycan link protein 2
0,95	ETAA1	Ewing tumor-associated antigen 1
0,95	DDX4	DEAD (Asp-Glu-Ala-Asp) box polypeptide 4
0,95	OR2W1	olfactory receptor, family 2, subfamily W, member 1
0,95	SYNPO2	synaptopodin 2
0,95	GRXCR2	glutaredoxin, cysteine rich 2
0,95	ORM2	orosomucoid 2
0,95	ZNF440	zinc finger protein 440
0,95	IL31RA	interleukin 31 receptor A
0,95	SCARB2	scavenger receptor class B, member 2
0,95	MARCH6	membrane associated ring finger 6
0,95	TEKT2	tektin 2 (testicular)
0,95	PCED1B	PC-esterase domain containing 1B
0,95	RPL13; SNORD68	ribosomal protein L13; small nucleolar RNA, C/D box 68
0,95	MTMR2	myotubularin related protein 2
0,95	C2orf15andLIPT1 andMRPL30; C2orf15	Transcript Identified by AceView, Entrez Gene ID(s) 150590; 51601; 51263; chromosome 2 open reading frame 15 [Source:HGNC Symbol;Acc:HGNC:28436]
0,95	LSM6	LSM6 homolog, U6 small nuclear RNA and mRNA degradation associated
0,95	FBXO25	F-box protein 25
0,95	CASQ1	calsequestrin 1 (fast-twitch, skeletal muscle)
0,95	TEX2	testis expressed 2
0,95	RBM45	RNA binding motif protein 45
0,95	DEFB112	defensin, beta 112
0,95	ATG16L1	autophagy related 16-like 1
0,95	TMPRSS7	transmembrane protease, serine 7
0,95	POLR2I	polymerase (RNA) II (DNA directed) polypeptide I, 14.5kDa

0,95	SLC7A1	solute carrier family 7 (cationic amino acid transporter, y+ system), member 1
0,95	GALNT15	polypeptide N-acetylgalactosaminyltransferase 15
0,95	WDR26; MIR4742	WD repeat domain 26; microRNA 4742
0,95	XRN1	5-3 exoribonuclease 1
0,95	NLRP5	NLR family, pyrin domain containing 5
0,95	ARRDC3	arrestin domain containing 3
0,95	MYO10	myosin X
0,95	FAM200B	family with sequence similarity 200, member B
0,95	SSH3	slingshot protein phosphatase 3
0,95	GRHPR	glyoxylate reductase/hydroxypyruvate reductase
0,95	AKAP10	A kinase (PRKA) anchor protein 10
0,95	CBLN2	cerebellin 2 precursor
0,95	KLHL6	kelch-like family member 6
0,95	HVCN1	hydrogen voltage gated channel 1
0,95	TMEM30A	transmembrane protein 30A
0,95	B3GNT6	UDP-GlcNAc:betaGal beta-1,3-N-acetylglucosaminyltransferase 6
0,95	NAGK	N-acetylglucosamine kinase
0,95	TUB	tubby bipartite transcription factor
0,95	ARHGEF39	Rho guanine nucleotide exchange factor 39
0,95	WAPL	WAPL cohesin release factor
0,95	WDR83OS	WD repeat domain 83 opposite strand
0,95	FMN1	Jeck2013 ANTISENSE, coding, INTERNAL, intronic best transcript NM_001103184
0,95	PREP	prolyl endopeptidase
0,95	HNRNPL	heterogeneous nuclear ribonucleoprotein L
0,95	CTCF	CCCTC-binding factor (zinc finger protein)
0,95	ZNF273	zinc finger protein 273
0,95	SLC2A14	solute carrier family 2 (facilitated glucose transporter), member 14
0,95	SKOR2	SKI family transcriptional corepressor 2
0,95	UBE3C	ubiquitin protein ligase E3C
0,95	FBXW5	F-box and WD repeat domain containing 5
0,95	SPPL2A	signal peptide peptidase like 2A
0,95	RPA1	replication protein A1
0,95	MED4	mediator complex subunit 4
0,95	OR10K1	olfactory receptor, family 10, subfamily K, member 1
0,95	OR4K5	olfactory receptor, family 4, subfamily K, member 5
0,95	MAPKAPK5; ADAM1A	mitogen-activated protein kinase-activated protein kinase 5; ADAM metallopeptidase domain 1A (pseudogene)
0,95	LRRC57	leucine rich repeat containing 57
0,95	KIF26A	kinesin family member 26A
0,95	SIK2	salt-inducible kinase 2
0,95	HEATR5B	HEAT repeat containing 5B
0,95	NAT2	N-acetyltransferase 2 (arylamine N-acetyltransferase)
0,95	PAX5	paired box 5
0,95	AFM	afamin

0,95	WDR6	WD repeat domain 6
0,95	PITHD1	PITH (C-terminal proteasome-interacting domain of thioredoxin-like) domain containing 1
0,95	RPL9	ribosomal protein L9
0,95	GRN	granulin
0,95	NFIB	nuclear factor I/B
0,95	CHN1	chimerin 1
0,95	LGALS8-AS1	LGALS8 antisense RNA 1
0,95	USP32	ubiquitin specific peptidase 32
0,95	MAGEA8	MAGE family member A8
0,95	JMJD1C-AS1	JMJD1C antisense RNA 1
0,95	C1orf56	chromosome 1 open reading frame 56
0,95	CLEC18A	C-type lectin domain family 18, member A
0,95	PHTF1	putative homeodomain transcription factor 1
0,95	SIGLEC14; SIGLEC5	sialic acid binding Ig-like lectin 14; sialic acid binding Ig-like lectin 5
0,95	GPANK1	G-patch domain and ankyrin repeats 1
0,95	SUMO3	small ubiquitin-like modifier 3
0,95	EPHA6	EPH receptor A6
0,95	ZNF501	zinc finger protein 501 [Source:HGNC Symbol;Acc:HGNC:23717]
0,95	SCD5	stearoyl-CoA desaturase 5
0,95	TTI2	TELO2 interacting protein 2
0,95	CXCR6	chemokine (C-X-C motif) receptor 6
0,95	CLTC	clathrin, heavy chain (Hc)
0,95	GEMIN8	gem nuclear organelle associated protein 8
0,95	FSCN1	fascin actin-bundling protein 1
0,95	MCM2	minichromosome maintenance complex component 2
0,95	MAZ	MYC-associated zinc finger protein (purine-binding transcription factor)
0,95	TLX1	T-cell leukemia homeobox 1
0,95	MOCOS	molybdenum cofactor sulfurase
0,95	CPSF2	cleavage and polyadenylation specific factor 2
0,95	PRAMEF9	PRAME family member 9
0,95	RAD17	RAD17 checkpoint clamp loader component
0,95	TASP1	taspase 1
0,95	NAB2	NGFI-A binding protein 2 (EGR1 binding protein 2)
0,95	CCT8	chaperonin containing TCP1, subunit 8 (theta)
0,95	TDRD3	tudor domain containing 3
0,95	UXS1	UDP-glucuronate decarboxylase 1
0,95	ZBTB24	zinc finger and BTB domain containing 24
0,95	KCNN4	potassium channel, calcium activated intermediate/small conductance subfamily N alpha, member 4
0,95	LINC01588	long intergenic non-protein coding RNA 1588
0,95	ZSCAN32	zinc finger and SCAN domain containing 32
0,95	EN1	engrailed homeobox 1
0,95	BCO2; RPS12P21	beta-carotene oxygenase 2; ribosomal protein S12 pseudogene 21
0,95	CPSF3	cleavage and polyadenylation specific factor 3

0,95	CCBE1	Jeck2013 ALT_ACCEPTOR, ALT_DONOR, coding, INTERNAL, intronic best transcript NM_133459
0,95	CHMP5	charged multivesicular body protein 5
0,95	LGR4	leucine-rich repeat containing G protein-coupled receptor 4
0,95	DIO1	deiodinase, iodothyronine, type I
0,95	MDFIC	MyoD family inhibitor domain containing
0,95	SERF1B; SERF1A	small EDRK-rich factor 1B (centromeric); small EDRK-rich factor 1A (telomeric)
0,95	SERF1A	small EDRK-rich factor 1A (telomeric)
0,95	HERC1	HECT and RLD domain containing E3 ubiquitin protein ligase family member 1
0,95	RP11-67L3.5; RP11-67L3.4; TMEM61	novel transcript; Transcript Identified by AceView, Entrez Gene ID(s) 199964
0,95	TIGD5	tigger transposable element derived 5
0,95	SHMT1; MIR6778	serine hydroxymethyltransferase 1 (soluble); microRNA 6778
0,95	DEPDC1	DEP domain containing 1
0,95	DNALI1	dynein, axonemal, light intermediate chain 1
0,95	FOXI3	forkhead box I3
0,95	SAMM50	SAMM50 sorting and assembly machinery component
0,95	IL22RA1	interleukin 22 receptor, alpha 1
0,95	FOXE1	forkhead box E1
0,95	BCL2L13	BCL2-like 13 (apoptosis facilitator)
0,95	ZNF730	zinc finger protein 730
0,95	RBP4	retinol binding protein 4, plasma
0,95	DEFB106A	defensin, beta 106A
0,95	SPAG8	sperm associated antigen 8
0,95	GGA2	golgi-associated, gamma adaptin ear containing, ARF binding protein 2
0,95	SGCE	sarcoglycan epsilon
0,95	PLEKHA8	pleckstrin homology domain containing, family A (phosphoinositide binding specific) member 8
0,95	MTHFD1	methylenetetrahydrofolate dehydrogenase (NADP+ dependent) 1, methenyltetrahydrofolate cyclohydrolase, formyltetrahydrofolate synthetase
0,95	ASS1	argininosuccinate synthase 1
0,95	CRISPLD2	cysteine-rich secretory protein LCCL domain containing 2
0,95	SP6	Sp6 transcription factor
0,95	HLA-DMB	major histocompatibility complex, class II, DM beta
0,95	TRAPPC3	trafficking protein particle complex 3
0,95	COL4A3	collagen, type IV, alpha 3 (Goodpasture antigen)
0,95	HIC1	hypermethylated in cancer 1
0,95	PYM1	PYM homolog 1, exon junction complex associated factor
0,95	ATP4A	ATPase, H+/K+ exchanging, alpha polypeptide
0,95	SPG7	spastic paraplegia 7 (pure and complicated autosomal recessive)

0,95	C6orf118	chromosome 6 open reading frame 118
0,95	ING5	inhibitor of growth family member 5
0,95	TCF19	transcription factor 19
0,95	PIAS2	protein inhibitor of activated STAT 2
0,95	UMODL1-AS1	UMODL1 antisense RNA 1
0,95	RASSF8	Ras association (RalGDS/AF-6) domain family (N-terminal) member 8
0,95	PASD1	PAS domain containing 1
0,95	LINC00272	long intergenic non-protein coding RNA 272
0,95	SAG	S-antigen; retina and pineal gland (arrestin)
0,95	MYO3B	myosin IIIB
0,95	COL17A1; MIR936	collagen, type XVII, alpha 1; microRNA 936
0,95	DUSP10	dual specificity phosphatase 10
0,95	TRIAP1	TP53 regulated inhibitor of apoptosis 1
0,95	TMEM190	transmembrane protein 190
0,95	TRIM27	tripartite motif containing 27
0,95	MAPRE3	microtubule-associated protein, RP/EB family, member 3
0,95	ERH	enhancer of rudimentary homolog (Drosophila)
0,95	ZNF423	zinc finger protein 423
0,95	SPEM1	spermatid maturation 1
0,95	MR1	major histocompatibility complex, class I-related
0,95	SURF2	surfeit 2 [Source:HGNC Symbol;Acc:HGNC:11475]
0,95	PLEKHF1	pleckstrin homology domain containing, family F (with FYVE domain) member 1
0,95	FAM122B	family with sequence similarity 122B
0,95	RIBC2	RIB43A domain with coiled-coils 2
0,95	FOXQ1	forkhead box Q1
0,95	PHKA2	phosphorylase kinase, alpha 2 (liver)
0,95	TBC1D22A	TBC1 domain family, member 22A
0,95	IFT81	intraflagellar transport 81
0,95	NMB	neuromedin B
0,95	CADM3	cell adhesion molecule 3
0,95	OAZ2	ornithine decarboxylase antizyme 2
0,95	SUN5	Sad1 and UNC84 domain containing 5
0,95	RFTN1	raftlin, lipid raft linker 1
0,95	ZNF546	zinc finger protein 546
0,95	PDCD10	programmed cell death 10
0,95	FDX1	ferredoxin 1
0,95	CENPI	centromere protein I
0,95	NEK3	NIMA-related kinase 3
0,95	ICOSLG	inducible T-cell co-stimulator ligand
0,95	PDCD7	programmed cell death 7
0,95	SREK1IP1	SREK1-interacting protein 1
0,95	SLC38A8	solute carrier family 38, member 8
0,95	ZNF257	zinc finger protein 257
0,95	TMBIM4	transmembrane BAX inhibitor motif containing 4
0,95	WDR44	WD repeat domain 44
0,95	BOK	BCL2-related ovarian killer

0,95	EVI5L	ecotropic viral integration site 5-like
0,95	C11orf58	chromosome 11 open reading frame 58
0,95	RPS25	ribosomal protein S25
0,95	P2RY2	purinergic receptor P2Y, G-protein coupled, 2
0,95	KIF5B	kinesin family member 5B
0,95	WDR90	WD repeat domain 90
0,95	PRKCA	Jeck2013 ALT_ACCEPTOR, ALT_DONOR, coding, INTERNAL, intronic best transcript NM_002737
0,95	SORCS2	sortilin-related VPS10 domain containing receptor 2
0,95	LEXM	lymphocyte expansion molecule
0,95	AP2S1	adaptor-related protein complex 2 sigma 1 subunit
0,95	ALPL	alkaline phosphatase, liver/bone/kidney
0,95	PAXIP1	PAX interacting (with transcription-activation domain) protein 1
0,95	KLF2	Kruppel-like factor 2
0,95	ELAVL3	ELAV like neuron-specific RNA binding protein 3
0,95	CLUAP1	clusterin associated protein 1
0,95	FLJ33534; AC062028.1	uncharacterized LOC285150; uncharacterized LOC285150 [Source:EntrezGene;Acc:285150]; novel transcript
0,95	NPIPB4	nuclear pore complex interacting protein family, member B4
0,95	CT45A7; CT45A6	cancer/testis antigen family 45, member A7; cancer/testis antigen family 45, member A6
0,95	CCR8	chemokine (C-C motif) receptor 8
0,95	XXYLT1	xyloside xylosyltransferase 1
0,95	STK31	serine/threonine kinase 31
0,95	OR1A1	olfactory receptor, family 1, subfamily A, member 1
0,95	HIST3H2A	histone cluster 3, H2a
0,95	EHD1	EH domain containing 1
0,95	HCN3	hyperpolarization activated cyclic nucleotide gated potassium channel 3
0,95	INVS	inversin
0,95	SLC4A1	solute carrier family 4 (anion exchanger), member 1 (Diego blood group)
0,95	BRI3BP	BRI3 binding protein
0,95	C2orf76	chromosome 2 open reading frame 76
0,95	OTUD1	OTU deubiquitinase 1
0,95	SNUPN	snurportin 1
0,95	RAPGEF1	Rap guanine nucleotide exchange factor 1
0,95	SNRPG	small nuclear ribonucleoprotein polypeptide G
0,95	REXO1L11P	REX1, RNA exonuclease 1 homolog-like 11, pseudogene
0,95	SMAD1	SMAD family member 1
0,95	DROSHA	drosha, ribonuclease type III
0,95	C16orf72	Memczak2013 ANTISENSE, coding, INTERNAL, intronic best transcript NM_014117
0,95	PPP1R12C	protein phosphatase 1, regulatory subunit 12C
0,95	GNAL	guanine nucleotide binding protein (G protein), alpha activating activity polypeptide, olfactory type

0,95	FAR1	fatty acyl CoA reductase 1
0,95	MAP3K7	mitogen-activated protein kinase kinase kinase 7
0,95	HELB	helicase (DNA) B
0,95	ZNF627	zinc finger protein 627
0,95	RNPEPL1	arginyl aminopeptidase (aminopeptidase B)-like 1
0,95	CRIM1	cysteine rich transmembrane BMP regulator 1 (chordin-like)
0,95	ARL14EP	ADP-ribosylation factor like GTPase 14 effector protein
0,95	GEMIN6	gem nuclear organelle associated protein 6
0,95	WBSCR17	Williams-Beuren syndrome chromosome region 17
0,95	SCMH1	sex comb on midleg homolog 1 (Drosophila)
0,95	DEFB106B; DEFB106A	defensin, beta 106B; defensin, beta 106A
0,95	MAGEA3	MAGE family member A3
0,95	CDR2L	cerebellar degeneration-related protein 2-like
0,95	ALDH9A1	aldehyde dehydrogenase 9 family, member A1
0,95	CTNNB1	catenin (cadherin-associated protein), beta 1
0,95	FAM53C	family with sequence similarity 53, member C
0,95	MFSD12	major facilitator superfamily domain containing 12
0,95	DAZ3	deleted in azoospermia 3
0,95	RPL41P1	ribosomal protein L41 pseudogene 1
0,95	CADM2	cell adhesion molecule 2
0,95	PGA4	pepsinogen 4, group I (pepsinogen A)
0,95	LOC100129924; C1orf50; RP5-994D16.11	uncharacterized LOC100129924; Transcript Identified by AceView, Entrez Gene ID(s) 79078; novel transcript
0,95	TLK2	tousled-like kinase 2
0,95	GDF2	growth differentiation factor 2
0,95	UBTF	upstream binding transcription factor, RNA polymerase I
0,95	MRPS9	mitochondrial ribosomal protein S9
0,95	PTCH2	patched 2
0,95	PNMAL2	paraneoplastic Ma antigen family-like 2
0,95	ADSL	adenylosuccinate lyase
0,95	GPR135	G protein-coupled receptor 135
0,95	CLCA4	chloride channel accessory 4
0,95	NEK4	NIMA-related kinase 4
0,95	GPRIN1	G protein regulated inducer of neurite outgrowth 1
0,95	KNCN	kinocilin
0,95	HNRNPA3; MIR4444-2; MIR4444-1	heterogeneous nuclear ribonucleoprotein A3; microRNA 4444-2; microRNA 4444-1
0,95	SIKE1	suppressor of IKBKE 1
0,95	TMED1	transmembrane p24 trafficking protein 1
0,95	FAM219B	family with sequence similarity 219, member B
0,95	SLC22A14	solute carrier family 22, member 14
0,95	FAM175B	family with sequence similarity 175, member B
0,95	ZNF597	zinc finger protein 597
0,95	RPL7A	ribosomal protein L7a

0,95	SNX16	sorting nexin 16
0,95	SSX2IP	synovial sarcoma, X breakpoint 2 interacting protein
0,95	RRM2	ribonucleotide reductase M2
0,95	ATPIF1	ATPase inhibitory factor 1
0,95	FMR1	fragile X mental retardation 1
0,95	NDUFA8	NADH dehydrogenase (ubiquinone) 1 alpha subcomplex, 8, 19kDa
0,95	EN2	engrailed homeobox 2
0,95	ITGA8	integrin alpha 8
0,95	CDK5	cyclin-dependent kinase 5
0,95	RNF225	ring finger protein 225
0,95	TMEM14C	transmembrane protein 14C
0,95	NQO1	NAD(P)H dehydrogenase, quinone 1
0,95	CLIC6	chloride intracellular channel 6
0,95	WRB	tryptophan rich basic protein
0,95	PCCB	propionyl-CoA carboxylase beta subunit
0,95	PPFIA1	protein tyrosine phosphatase, receptor type, f polypeptide (PTPRF), interacting protein (liprin), alpha 1
0,95	OCIAD1	OCIA domain containing 1
0,95	IP6K1	inositol hexakisphosphate kinase 1
0,95	MAGI3	membrane associated guanylate kinase, WW and PDZ domain containing 3
0,95	TRIML2	tripartite motif family like 2
0,95	DGCR8	Memczak2013 ALT_ACCEPTOR, ALT_DONOR, coding, INTERNAL, intronic best transcript NM_022720
0,95	USP44	ubiquitin specific peptidase 44
0,95	MBLAC2	metallo-beta-lactamase domain containing 2
0,95	SLC35G5	solute carrier family 35, member G5
0,95	CMAS	cytidine monophosphate N-acetylneuraminic acid synthetase
0,95	CAMKK1	calcium/calmodulin-dependent protein kinase kinase 1, alpha
0,95	MED22	mediator complex subunit 22
0,95	RP11-517A5.5; NOMO3; RP11-517A5.4	NODAL modulator 3 [Source:HGNC Symbol;Acc:HGNC:25242]; novel transcript, antisense to NOMO3; RP11-517A5.4 (from geneSymbol)
0,95	MLEC	malectin
0,95	SEC61A1	Sec61 translocon alpha 1 subunit
0,95	ZCCHC12	zinc finger, CCHC domain containing 12
0,95	ZC3HAV1L	zinc finger CCCH-type, antiviral 1-like
0,95	SECISBP2	SECIS binding protein 2
0,95	LRRC53	leucine rich repeat containing 53
0,95	ANO8	anoctamin 8
0,95	CNPY3	Memczak2013 ALT_ACCEPTOR, ALT_DONOR, coding, INTERNAL, intronic best transcript NM_006586
0,95	DNAJC24	DnaJ (Hsp40) homolog, subfamily C, member 24

0,95	DDOST	dolichyl-diphosphooligosaccharide--protein glycosyltransferase subunit (non-catalytic)
0,95	RUSC2	RUN and SH3 domain containing 2
0,95	CDC25B	cell division cycle 25B
0,95	KRTAP2-4	keratin associated protein 2-4
0,95	VWC2	von Willebrand factor C domain containing 2
0,95	CXCL17	chemokine (C-X-C motif) ligand 17
0,95	MPC2	mitochondrial pyruvate carrier 2
0,95	PAQR7	progestin and adipoQ receptor family member VII
0,95	RBFOX1	RNA binding protein, fox-1 homolog (C. elegans) 1
0,95	ARPP21	Transcript Identified by AceView, Entrez Gene ID(s) 10777
0,95	TMED4	transmembrane p24 trafficking protein 4
0,95	MB21D1	Mab-21 domain containing 1
0,95	CDH22	cadherin 22, type 2
0,95	RPS14	ribosomal protein S14
0,95	PFN2	profilin 2
0,95	TIE1	tyrosine kinase with immunoglobulin-like and EGF-like domains 1
0,95	EFTUD1	elongation factor Tu GTP binding domain containing 1
0,95	TSPAN12	tetraspanin 12
0,95	FLT3	fms-related tyrosine kinase 3
0,95	ZNF562	zinc finger protein 562
0,95	WLS	wntless Wnt ligand secretion mediator
0,95	IER5	immediate early response 5
0,95	CRH	corticotropin releasing hormone
0,95	DCXR	dicarbonyl/L-xylulose reductase
0,95	UGP2	UDP-glucose pyrophosphorylase 2
0,95	PDE8B	phosphodiesterase 8B
0,95	GOLM1	golgi membrane protein 1
0,95	FSTL4	follistatin-like 4
0,95	FAM155A	family with sequence similarity 155, member A
0,95	PPP1R14B	protein phosphatase 1, regulatory (inhibitor) subunit 14B
0,95	PPIL4	peptidylprolyl isomerase (cyclophilin)-like 4
0,95	C1orf109	chromosome 1 open reading frame 109
0,95	PIK3CD-AS2	PIK3CD antisense RNA 2
0,95	PDXK	pyridoxal (pyridoxine, vitamin B6) kinase
0,95	ZNF747	zinc finger protein 747
0,95	IGFBPL1	insulin like growth factor binding protein-like 1
0,95	MIIP	migration and invasion inhibitory protein
0,95	MEAF6	MYST/Esa1-associated factor 6
0,95	TXNDC17	thioredoxin domain containing 17
0,95	FCHO1	FCH domain only 1
0,95	SYT8	synaptotagmin VIII
0,95	GPR75	G protein-coupled receptor 75
0,95	MEA1	male-enhanced antigen 1
0,95	KDM4C	lysine (K)-specific demethylase 4C

0,95	GNAI2	guanine nucleotide binding protein (G protein), alpha inhibiting activity polypeptide 2
0,95	DPM3	dolichyl-phosphate mannosyltransferase polypeptide 3
0,95	GIMAP7	GTPase, IMAP family member 7
0,95	PUM3	pumilio RNA binding family member 3
0,95	CRYAA	crystallin alpha A
0,95	CYR61	cysteine-rich, angiogenic inducer, 61
0,95	CCAR2	cell cycle and apoptosis regulator 2
0,95	UGT2B4	UDP glucuronosyltransferase 2 family, polypeptide B4
0,95	DMRTC1B; DMRTC1	DMRT-like family C1B; DMRT-like family C1
0,95	JPH4	junctophilin 4
0,95	DUSP19	dual specificity phosphatase 19
0,95	QDPR	quinoid dihydropteridine reductase
0,95	COX5B	cytochrome c oxidase subunit Vb
0,95	GTF3C6	general transcription factor IIIC subunit 6
0,95	PBX1	pre-B-cell leukemia homeobox 1
0,95	GAB1	GRB2-associated binding protein 1
0,95	CCDC71	coiled-coil domain containing 71
0,95	DKKL1	dickkopf-like 1
0,95	PRPF31	pre-mRNA processing factor 31
0,95	POLL	polymerase (DNA directed), lambda
0,95	AZU1	azurocidin 1
0,95	NAIF1	nuclear apoptosis inducing factor 1
0,95	RPL41P2	ribosomal protein L41 pseudogene 2
0,95	ABL1	ABL proto-oncogene 1, non-receptor tyrosine kinase
0,95	ULBP1	UL16 binding protein 1
0,95	GMEB1	glucocorticoid modulatory element binding protein 1
0,95	ANAPC16	anaphase promoting complex subunit 16
0,95	PIKFYVE	phosphoinositide kinase, FYVE finger containing
0,95	RFC2	replication factor C subunit 2
0,95	GTDC1	glycosyltransferase like domain containing 1
0,95	FLT3LG	fms-related tyrosine kinase 3 ligand
0,95	KIF22	kinesin family member 22
0,95	EIF6	eukaryotic translation initiation factor 6
0,95	MGST3	microsomal glutathione S-transferase 3
0,95	FOXD1	forkhead box D1
0,95	MFNG	MFNG O-fucosylpeptide 3-beta-N-acetylglucosaminyltransferase
0,95	TXK	TXK tyrosine kinase
0,95	TSPY10	testis specific protein, Y-linked 10
0,95	DEPDC5	DEP domain containing 5
0,95	IKBKB	inhibitor of kappa light polypeptide gene enhancer in B-cells, kinase beta
0,95	RHOV	ras homolog family member V
0,95	YEATS2	YEATS domain containing 2
0,95	OSBPL2	oxysterol binding protein-like 2
0,95	CYBRD1	cytochrome b reductase 1

0,95	ZNF19	zinc finger protein 19
0,95	OR51A2	olfactory receptor, family 51, subfamily A, member 2
0,95	MEX3C	mex-3 RNA binding family member C
0,95	SRRT	serrate, RNA effector molecule
0,95	CCDC67	coiled-coil domain containing 67
0,95	SEC14L5	SEC14-like lipid binding 5
0,95	SLC12A9	solute carrier family 12, member 9
0,95	PRKRIR	protein-kinase, interferon-inducible double stranded RNA dependent inhibitor, repressor of (P58 repressor)
0,95	EEF2; SNORD37	eukaryotic translation elongation factor 2; small nucleolar RNA, C/D box 37
0,95	SAPCD2	suppressor APC domain containing 2
0,95	ANKS6	ankyrin repeat and sterile alpha motif domain containing 6
0,95	KCND2	potassium channel, voltage gated Shal related subfamily D, member 2
0,95	C7orf25	chromosome 7 open reading frame 25
0,95	POLR3D	polymerase (RNA) III (DNA directed) polypeptide D, 44kDa
0,95	PPCS; CCDC30	phosphopantothenoylcysteine synthetase; coiled-coil domain containing 30
0,95	RAP1GDS1	RAP1, GTP-GDP dissociation stimulator 1
0,95	C8A	complement component 8, alpha polypeptide
0,95	ZNF439	zinc finger protein 439
0,95	CDKL2	cyclin-dependent kinase-like 2 (CDC2-related kinase)
0,95	CTSG	cathepsin G
0,95	C4orf50	chromosome 4 open reading frame 50
0,95	MAT1A	methionine adenosyltransferase I, alpha
0,95	SURF4	surfeit 4
0,95	ATP10D	ATPase, class V, type 10D
0,95	HEXDC	hexosaminidase (glycosyl hydrolase family 20, catalytic domain) containing
0,95	BBX	Memczak2013 ANTISENSE, coding, INTERNAL, intronic best transcript NM_020235
0,95	TRIM63	tripartite motif containing 63, E3 ubiquitin protein ligase
0,95	NUDT13	nudix hydrolase 13
0,95	E2F4	E2F transcription factor 4, p107/p130-binding
0,95	CABS1	calcium-binding protein, spermatid-specific 1
0,95	PITX1	paired-like homeodomain 1
0,95	UBE2D3	ubiquitin conjugating enzyme E2D 3
0,95	SLC41A1	solute carrier family 41 (magnesium transporter), member 1
0,95	PRPF40B	PRP40 homolog, pre-mRNA processing factor B
0,95	RAB9A	RAB9A, member RAS oncogene family
0,95	SNX5	Jeck2013 ALT_DONOR, coding, INTERNAL, intronic best transcript NM_152227
0,95	CLU; MIR6843	clusterin; microRNA 6843
0,95	ATXN7L2	ataxin 7-like 2

0,95	STX18	syntaxin 18
0,95	FAAP24	Fanconi anemia core complex associated protein 24
0,95	TRIM44	tripartite motif containing 44
0,95	TMEM135	transmembrane protein 135
0,95	EXTL3	exostosin-like glycosyltransferase 3
0,95	SLC2A3	solute carrier family 2 (facilitated glucose transporter), member 3
0,95	PRPF18	pre-mRNA processing factor 18
0,95	RWDD1	RWD domain containing 1
0,95	FBL	fibrillarin
0,95	ZNF484	zinc finger protein 484
0,95	SLC25A5	solute carrier family 25 (mitochondrial carrier; adenine nucleotide translocator), member 5
0,95	ZNF649	zinc finger protein 649
0,95	NDUFA11	NADH dehydrogenase (ubiquinone) 1 alpha subcomplex, 11, 14.7kDa
0,95	ZNF91	zinc finger protein 91
0,95	CRLS1	cardiolipin synthase 1
0,95	KDELC2	KDEL (Lys-Asp-Glu-Leu) containing 2
0,95	MOV10L1	Mov10 RISC complex RNA helicase like 1
0,95	KIAA1257	KIAA1257
0,95	HTRA2	HtrA serine peptidase 2
0,95	FCHO2	FCH domain only 2
0,95	TXNDC15	thioredoxin domain containing 15
0,95	FOXD4L1	forkhead box D4-like 1
0,95	GPD2	glycerol-3-phosphate dehydrogenase 2
0,95	ALDH8A1	aldehyde dehydrogenase 8 family, member A1
0,95	LINC00269	long intergenic non-protein coding RNA 269
0,95	TMEM9	transmembrane protein 9
0,95	WNT8B	wingless-type MMTV integration site family, member 8B
0,95	FOXK2	forkhead box K2
0,95	KCTD5	potassium channel tetramerization domain containing 5
0,95	BAALC	brain and acute leukemia, cytoplasmic
0,95	SOX11	SRY box 11
0,95	MPHOSPH10	M-phase phosphoprotein 10 (U3 small nucleolar ribonucleoprotein)
0,95	KPNA6	karyopherin alpha 6 (importin alpha 7)
0,95	PHF21B	PHD finger protein 21B
0,95	ESYT1	extended synaptotagmin-like protein 1
0,95	SSX3	synovial sarcoma, X breakpoint 3
0,95	NDC80	NDC80 kinetochore complex component
0,95	GGN	gametogenetin
0,95	PDLIM7	PDZ and LIM domain 7 (enigma)
0,95	BRCA1	breast cancer 1, early onset
0,95	THAP5	THAP domain containing 5
0,95	TCEB3C	transcription elongation factor B polypeptide 3C (elongin A3)
0,95	ZNF20	zinc finger protein 20

0,95	MED19	mediator complex subunit 19
0,95	RWDD4	RWD domain containing 4
0,95	B4GALT7	xylosylprotein beta 1,4-galactosyltransferase, polypeptide 7
0,95	OR2M3	olfactory receptor, family 2, subfamily M, member 3
0,95	SMYD2	SET and MYND domain containing 2
0,95	FMO1	flavin containing monooxygenase 1
0,95	CSNK1G2	casein kinase 1, gamma 2
0,95	RPS6KA1	ribosomal protein S6 kinase, 90kDa, polypeptide 1
0,95	RPTN	repetin
0,95	TRIM49C	tripartite motif containing 49C
0,95	OR5V1	olfactory receptor, family 5, subfamily V, member 1
0,95	CLTA	clathrin, light chain A
0,95	KANK2	KN motif and ankyrin repeat domains 2
0,95	KIFC3	kinesin family member C3
0,95	ZWINT	ZW10 interacting kinetochore protein
0,95	POLR2K	polymerase (RNA) II (DNA directed) polypeptide K, 7.0kDa
0,95	RIPK4	receptor-interacting serine-threonine kinase 4
0,95	FAU	Finkel-Biskis-Reilly murine sarcoma virus (FBR-MuSV) ubiquitously expressed
0,95	EDIL3	EGF-like repeats and discoidin I-like domains 3
0,95	ITM2B	integral membrane protein 2B
0,95	CEACAM7	carcinoembryonic antigen-related cell adhesion molecule 7
0,95	CLDND1	claudin domain containing 1
0,95	PIGB	phosphatidylinositol glycan anchor biosynthesis class B
0,95	RPL7A	ribosomal protein L7a
0,95	C2orf82	chromosome 2 open reading frame 82
0,95	PSD	pleckstrin and Sec7 domain containing
0,95	THNSL1	threonine synthase-like 1
0,95	CASP2	caspase 2
0,95	CENPO	centromere protein O
0,95	LINC00518	long intergenic non-protein coding RNA 518
0,95	HS3ST5	heparan sulfate (glucosamine) 3-O-sulfotransferase 5
0,95	MIEN1	migration and invasion enhancer 1
0,95	MRPL14	mitochondrial ribosomal protein L14
0,95	ZNF138	zinc finger protein 138
0,95	AP3D1	adaptor-related protein complex 3, delta 1 subunit
0,95	FOSL1	FOS-like antigen 1
0,95	TMEM174	transmembrane protein 174
0,95	RAP1A	RAP1A, member of RAS oncogene family
0,95	TYMP; SCO2	thymidine phosphorylase; SCO2 cytochrome c oxidase assembly protein
0,95	S100A1	S100 calcium binding protein A1
0,95	SAG	S-antigen; retina and pineal gland (arrestin)
0,95	TADA2B	Transcript Identified by AceView, Entrez Gene ID(s) 93624

0,95	PTPRG	Jeck2013 ALT_ACCEPTOR, ALT_DONOR, coding, INTERNAL, intronic best transcript NM_002841
0,95	ADTRP	androgen-dependent TFPI-regulating protein
0,95	EGLN3	egl-9 family hypoxia-inducible factor 3
0,95	EPAS1	endothelial PAS domain protein 1
0,95	SENP2	SUMO1/sentrin/SMT3 specific peptidase 2
0,95	BPIFB1	BPI fold containing family B, member 1
0,95	USP34	Memczak2013 ANTISENSE, CDS, coding, INTERNAL best transcript NM_014709
0,95	USP31	ubiquitin specific peptidase 31
0,95	PHF8	PHD finger protein 8
0,95	C1orf122	chromosome 1 open reading frame 122
0,95	EFCAB3	EF-hand calcium binding domain 3
0,95	ARL1	ADP-ribosylation factor like GTPase 1
0,95	ADAMTS1	ADAM metallopeptidase with thrombospondin type 1 motif 1
0,95	ADAMTS13	ADAM metallopeptidase with thrombospondin type 1 motif 13
0,95	ZNF879	zinc finger protein 879
0,95	CKLF; CKLF-CMTM1	chemokine-like factor; CKLF-CMTM1 readthrough
0,95	NIM1K	NIM1 serine/threonine protein kinase
0,95	HCRTR2	hypocretin (orexin) receptor 2
0,95	ARPC4-TTLL3	ARPC4-TTLL3 readthrough
0,95	LY6K	lymphocyte antigen 6 complex, locus K
0,95	ACTRT1	actin-related protein T1
0,95	CLIC1	chloride intracellular channel 1
0,95	SLC37A3	solute carrier family 37, member 3
0,95	PARG	poly (ADP-ribose) glycohydrolase
0,95	TDGF1	teratocarcinoma-derived growth factor 1
0,95	SLITRK4	SLIT and NTRK-like family, member 4
0,95	SRI	sorcin
0,95	SPATA5L1	spermatogenesis associated 5-like 1
0,95	C14orf93	chromosome 14 open reading frame 93
0,95	CSRNP1	cysteine-serine-rich nuclear protein 1
0,95	LHX2	LIM homeobox 2
0,95	KIAA1143	KIAA1143 [Source:HGNC Symbol;Acc:HGNC:29198]
0,95	RPL41P5	ribosomal protein L41 pseudogene 5
0,95	MTERF1	mitochondrial transcription termination factor 1
0,95	ZFP42	ZFP42 zinc finger protein
0,95	ZC3HAV1	zinc finger CCCH-type, antiviral 1
0,95	GMCL1	germ cell-less, spermatogenesis associated 1
0,95	CRP	C-reactive protein, pentraxin-related
0,95	PIR	pirin
0,95	COX19	COX19 cytochrome c oxidase assembly factor
0,95	CTAGE6	CTAGE family, member 6
0,95	PFKM	phosphofructokinase, muscle
0,95	ZNF16	zinc finger protein 16
0,95	SAFB2	scaffold attachment factor B2

0,95	ARHGAP32	Rho GTPase activating protein 32
0,95	OR4M1	olfactory receptor, family 4, subfamily M, member 1
0,95	R3HDM4	R3H domain containing 4
0,95	HMCES	5-hydroxymethylcytosine (hmC) binding, ES cell-specific
0,95	SURF4	surfeit 4
0,95	CCL13	chemokine (C-C motif) ligand 13
0,95	BCL2A1	BCL2-related protein A1
0,95	SPATA2	spermatogenesis associated 2
0,95	CCDC114	coiled-coil domain containing 114
0,95	FOXRED2	FAD-dependent oxidoreductase domain containing 2
0,95	CHD2; MIR3175	chromodomain helicase DNA binding protein 2; microRNA 3175
0,95	ADCY6; MIR4701	adenylate cyclase 6; microRNA 4701
0,95	PHTF2	putative homeodomain transcription factor 2
0,95	CKAP5	Transcript Identified by AceView, Entrez Gene ID(s) 9793
0,95	STOML1	stomatin (EPB72)-like 1
0,95	GBE1	glucan (1,4-alpha-), branching enzyme 1
0,95	CCRL2	chemokine (C-C motif) receptor-like 2
0,95	LOC100133669; RP11-273G15.2	Homo sapiens uncharacterized LOC100133669 (LOC100133669), long non-coding RNA.; Transcript Identified by AceView, Entrez Gene ID(s) 100133669; novel transcript
0,95	AFP	alpha-fetoprotein
0,95	NIPAL1	NIPA-like domain containing 1
0,95	NAGLU	N-acetylglucosaminidase, alpha
0,95	PPP1R3F	protein phosphatase 1, regulatory subunit 3F
0,95	MS4A14	membrane-spanning 4-domains, subfamily A, member 14
0,95	FRG2B	FSHD region gene 2 family, member B
0,95	HTR1A	5-hydroxytryptamine (serotonin) receptor 1A, G protein-coupled
0,95	ADAMTSL3	ADAMTS like 3
0,95	SLAMF9	SLAM family member 9
0,95	ZNF250	zinc finger protein 250
0,94	LRRC37A2	leucine rich repeat containing 37, member A2
0,94	PLA2G12A	phospholipase A2, group XIIA
0,94	PRRT2	proline-rich transmembrane protein 2
0,94	CHL1	cell adhesion molecule L1-like
0,94	FHL1	four and a half LIM domains 1
0,94	COL6A2	Jeck2013 ALT_ACCEPTOR, ALT_DONOR, coding, INTERNAL, intronic best transcript NM_058175
0,94	DDX26B	DEAD/H (Asp-Glu-Ala-Asp/His) box polypeptide 26B
0,94	ARL8A	ADP-ribosylation factor like GTPase 8A
0,94	GSG1L	GSG1-like
0,94	ZBTB7A	zinc finger and BTB domain containing 7A
0,94	ZC3H4	zinc finger CCCH-type containing 4

0,94	ATP13A1	ATPase type 13A1
0,94	CXorf21	chromosome X open reading frame 21
0,94	C8orf58	chromosome 8 open reading frame 58
0,94	MUC1	mucin 1, cell surface associated
0,94	GLOD4	glyoxalase domain containing 4
0,94	FGF3	fibroblast growth factor 3
0,94	MTRNR2L13	MT-RNR2-like 13
0,94	VTN	vitronectin
0,94	LGALS3BP	lectin, galactoside-binding, soluble, 3 binding protein
0,94	ZFR2	zinc finger RNA binding protein 2
0,94	CCDC96	coiled-coil domain containing 96
0,94	FN3KRP	fructosamine 3 kinase related protein
0,94	RNPC3	RNA binding region (RNP1, RRM) containing 3
0,94	NAB1	NGFI-A binding protein 1
0,94	NUCKS1	nuclear casein kinase and cyclin-dependent kinase substrate 1
0,94	GSKIP	GSK3B interacting protein
0,94	TSPAN9	tetraspanin 9
0,94	SLC27A4	solute carrier family 27 (fatty acid transporter), member 4
0,94	H2AFY	H2A histone family, member Y
0,94	HIPK2	homeodomain interacting protein kinase 2
0,94	MIS12	MIS12 kinetochore complex component
0,94	CARD8	caspase recruitment domain family, member 8
0,94	DGKZ	diacylglycerol kinase, zeta
0,94	CREB3L1	cAMP responsive element binding protein 3-like 1
0,94	HM13; MCTS2P	histocompatibility (minor) 13; malignant T-cell amplified sequence 2, pseudogene
0,94	NPIPB3	nuclear pore complex interacting protein family, member B3
0,94	NR6A1	nuclear receptor subfamily 6, group A, member 1
0,94	PEX6	peroxisomal biogenesis factor 6
0,94	MYBPHL	myosin binding protein H-like
0,94	PIH1D1	PIH1 domain containing 1
0,94	BSX	brain-specific homeobox
0,94	RPL41	Synthetic construct Homo sapiens clone IMAGE:100063377, MGC:190767 ribosomal protein L41 (RPL41) mRNA, encodes complete protein.
0,94	ESD	esterase D
0,94	CCNDBP1	cyclin D-type binding-protein 1
0,94	BTBD9	Memczak2013 ALT_ACCEPTOR, ALT_DONOR, coding, INTERNAL, intronic best transcript NM_001099272
0,94	FREM2	FRAS1 related extracellular matrix protein 2
0,94	COMMD6	COMM domain containing 6
0,94	USP17L15	ubiquitin specific peptidase 17-like family member 15
0,94	PHACTR2	phosphatase and actin regulator 2
0,94	H3F3A; H3F3AP4	H3 histone, family 3A; H3 histone, family 3A, pseudogene 4

0,94	BCL10	B-cell CLL/lymphoma 10
0,94	NUDT14	nudix hydrolase 14
0,94	USP17L11; USP17L18; USP17L20	ubiquitin specific peptidase 17-like family member 11; ubiquitin specific peptidase 17-like family member 18; ubiquitin specific peptidase 17-like family member 20
0,94	TMEM201	transmembrane protein 201
0,94	ESRP1	epithelial splicing regulatory protein 1
0,94	NKPD1	NTPase, KAP family P-loop domain containing 1
0,94	KIF2C	kinesin family member 2C
0,94	RPL35A	ribosomal protein L35a
0,94	PGLYRP2	peptidoglycan recognition protein 2
0,94	C9orf69	chromosome 9 open reading frame 69
0,94	KCTD15	potassium channel tetramerization domain containing 15
0,94	SNX14	sorting nexin 14
0,94	ERN2	endoplasmic reticulum to nucleus signaling 2
0,94	SLC37A2	solute carrier family 37 (glucose-6-phosphate transporter), member 2
0,94	GTSE1	G-2 and S-phase expressed 1
0,94	ANKRD44	Memczak2013 ANTISENSE, coding, INTERNAL, intronic best transcript NM_001195144
0,94	ZBTB4	zinc finger and BTB domain containing 4
0,94	ATG4A	autophagy related 4A, cysteine peptidase
0,94	CHRM4	cholinergic receptor, muscarinic 4
0,94	IPO5	importin 5
0,94	CSNK1A1	casein kinase 1, alpha 1
0,94	SAAL1	serum amyloid A-like 1
0,94	GJB6	gap junction protein beta 6
0,94	ATP6AP1L	ATPase, H+ transporting, lysosomal accessory protein 1-like
0,94	TMCO1	transmembrane and coiled-coil domains 1
0,94	RAE1	ribonucleic acid export 1
0,94	ZNF311	zinc finger protein 311
0,94	OR5AS1	olfactory receptor, family 5, subfamily AS, member 1
0,94	AGT	angiotensinogen (serpin peptidase inhibitor, clade A, member 8)
0,94	B4GALT2	UDP-Gal:betaGlcNAc beta 1,4- galactosyltransferase, polypeptide 2
0,94	CRTAM	cytotoxic and regulatory T-cell molecule
0,94	PRORY	proline rich, Y-linked
0,94	F13A1	coagulation factor XIII, A1 polypeptide
0,94	CWF19L2	CWF19-like 2, cell cycle control (S. pombe)
0,94	SORBS3	sorbin and SH3 domain containing 3
0,94	PPP1R14D	protein phosphatase 1, regulatory (inhibitor) subunit 14D
0,94	PLEKHA7	pleckstrin homology domain containing, family A member 7
0,94	CASP8	caspase 8, apoptosis-related cysteine peptidase

0,94	DHRS3; MIR6730	dehydrogenase/reductase (SDR family) member 3; microRNA 6730
0,94	AHSP	alpha hemoglobin stabilizing protein
0,94	GREM1	gremlin 1, DAN family BMP antagonist [Source:HGNC Symbol;Acc:HGNC:2001]
0,94	ZDHHC16	zinc finger, DHHC-type containing 16
0,94	METTL25	methyltransferase like 25
0,94	RBM3	RNA binding motif (RNP1, RRM) protein 3
0,94	ZBED9	zinc finger, BED-type containing 9
0,94	TGFB2; TGFB2-OT1	transforming growth factor beta 2; TGFB2 overlapping transcript 1
0,94	KMT5A	lysine (K)-specific methyltransferase 5A
0,94	ATP2A2	ATPase, Ca++ transporting, cardiac muscle, slow twitch 2
0,94	NAGA	N-acetylgalactosaminidase, alpha-
0,94	NUP85	nucleoporin 85kDa
0,94	RUVBL1	RuvB-like AAA ATPase 1
0,94	LZTS1	leucine zipper, putative tumor suppressor 1
0,94	FEM1A	fem-1 homolog a (C. elegans)
0,94	CTH	cystathionine gamma-lyase
0,94	EIF2S2	eukaryotic translation initiation factor 2, subunit 2 beta, 38kDa
0,94	MIB2	mindbomb E3 ubiquitin protein ligase 2
0,94	MIF	macrophage migration inhibitory factor (glycosylation-inhibiting factor)
0,94	HOXC12	homeobox C12
0,94	FLG2	filaggrin family member 2
0,94	PPIE	peptidylprolyl isomerase E (cyclophilin E)
0,94	DPEP3	dipeptidase 3
0,94	SWAP70	SWAP switching B-cell complex 70kDa subunit
0,94	NCBP1	nuclear cap binding protein subunit 1
0,94	TRIM67	tripartite motif containing 67
0,94	GATSL3	GATS protein-like 3
0,94	KNG1	kininogen 1
0,94	SERPINA10	serpin peptidase inhibitor, clade A (alpha-1 antiproteinase, antitrypsin), member 10
0,94	KRR1	KRR1, small subunit (SSU) processome component, homolog (yeast)
0,94	RBM27	RNA binding motif protein 27
0,94	PCOLCE-AS1	PCOLCE antisense RNA 1
0,94	NPIPB8	nuclear pore complex interacting protein family, member B8
0,94	ATP6V0B	ATPase, H+ transporting, lysosomal 21kDa, V0 subunit b
0,94	SBNO1; MIR8072	strawberry notch homolog 1 (Drosophila); microRNA 8072
0,94	C19orf38	chromosome 19 open reading frame 38
0,94	SLC15A4	solute carrier family 15 (oligopeptide transporter), member 4
0,94	REXO1L2P	REX1, RNA exonuclease 1 homolog-like 2, pseudogene

0,94	REXO1L4P	REX1, RNA exonuclease 1 homolog-like 4, pseudogene
0,94	MPHOSPH9	M-phase phosphoprotein 9
0,94	KIF12	kinesin family member 12
0,94	HIST1H2BO	histone cluster 1, H2bo
0,94	TERT	Zhang2013 ALT_ACCEPTOR, ALT_DONOR, coding, INTERNAL, intronic best transcript NM_198253
0,94	NELFE; MIR1236	negative elongation factor complex member E; microRNA 1236
0,94	MORC2	MORC family CW-type zinc finger 2
0,94	HHLA2	HERV-H LTR-associating 2
0,94	KLRF2	killer cell lectin-like receptor subfamily F, member 2
0,94	HLA-G	major histocompatibility complex, class I, G
0,94	IL4	interleukin 4
0,94	SLC25A37	solute carrier family 25 (mitochondrial iron transporter), member 37
0,94	ZEB1	zinc finger E-box binding homeobox 1
0,94	PPP4R1	protein phosphatase 4, regulatory subunit 1
0,94	MLST8	MTOR associated protein, LST8 homolog
0,94	SLC25A40	solute carrier family 25, member 40
0,94	HIST1H2BB	histone cluster 1, H2bb
0,94	ACE2	angiotensin I converting enzyme 2
0,94	C11orf42	chromosome 11 open reading frame 42
0,94	AADACL4	arylacetamide deacetylase-like 4
0,94	MIER3	mesoderm induction early response 1, family member 3
0,94	PARK7	parkinson protein 7
0,94	CLCF1	cardiotrophin-like cytokine factor 1
0,94	UVRAG	UV radiation resistance associated
0,94	ZBTB37	zinc finger and BTB domain containing 37
0,94	E2F2	E2F transcription factor 2
0,94	TOR2A	torsin family 2, member A
0,94	SLC4A2	solute carrier family 4 (anion exchanger), member 2
0,94	KLHL15	kelch-like family member 15
0,94	AMPD1	adenosine monophosphate deaminase 1
0,94	MTX2	metaxin 2
0,94	OXCT1	3-oxoacid CoA-transferase 1
0,94	EPHX1	epoxide hydrolase 1, microsomal (xenobiotic)
0,94	VASN	vasorin
0,94	DET1	de-etiolated homolog 1 (Arabidopsis)
0,94	GSTA5	glutathione S-transferase alpha 5
0,94	PIH1D2	PIH1 domain containing 2
0,94	SLC25A26	solute carrier family 25 (S-adenosylmethionine carrier), member 26
0,94	HLA-DRB5	major histocompatibility complex, class II, DR beta 5
0,94	RPS21	ribosomal protein S21
0,94	SKP2	Transcript Identified by AceView, Entrez Gene ID(s) 6502
0,94	CATSPERB	catsper channel auxiliary subunit beta

0,94	FBXL5	F-box and leucine-rich repeat protein 5
0,94	CT47A9; CT47A4; CT47A11	cancer/testis antigen family 47, member A9; cancer/testis antigen family 47, member A4; cancer/testis antigen family 47, member A11
0,94	CT47A3; CT47A10	cancer/testis antigen family 47, member A3; cancer/testis antigen family 47, member A10
0,94	CT47A10; CT47A8; CT47A9	cancer/testis antigen family 47, member A10; cancer/testis antigen family 47, member A8; cancer/testis antigen family 47, member A9
0,94	CT47A6; CT47A4	cancer/testis antigen family 47, member A6; cancer/testis antigen family 47, member A4
0,94	CT47A3	cancer/testis antigen family 47, member A3
0,94	CT47A2	cancer/testis antigen family 47, member A2
0,94	CT47A12; CT47A8	cancer/testis antigen family 47, member A12; cancer/testis antigen family 47, member A8
0,94	OR5A1	olfactory receptor, family 5, subfamily A, member 1
0,94	HCFC1	host cell factor C1
0,94	GDF3	growth differentiation factor 3
0,94	OR2A5	olfactory receptor, family 2, subfamily A, member 5
0,94	ALOX15	arachidonate 15-lipoxygenase
0,94	GLYCTK	glycerate kinase
0,94	C19orf67	chromosome 19 open reading frame 67
0,94	FPR3	formyl peptide receptor 3
0,94	PSMD13	proteasome 26S subunit, non-ATPase 13
0,94	RAB3D	RAB3D, member RAS oncogene family
0,94	CACNG2	calcium channel, voltage-dependent, gamma subunit 2
0,94	TXNDC12; KTI12	thioredoxin domain containing 12 (endoplasmic reticulum); KTI12 chromatin associated homolog
0,94	AURKC	aurora kinase C
0,94	VEZF1	vascular endothelial zinc finger 1
0,94	FAM53B	family with sequence similarity 53, member B
0,94	ALPK3	alpha kinase 3
0,94	MTMR6	myotubularin related protein 6
0,94	ZC3H3	Transcript Identified by AceView, Entrez Gene ID(s) 23144
0,94	ULK3	unc-51 like kinase 3
0,94	SIAH3	siah E3 ubiquitin protein ligase family member 3
0,94	LINC00504	long intergenic non-protein coding RNA 504
0,94	ZNF304	zinc finger protein 304
0,94	LYSMD4	LysM, putative peptidoglycan-binding, domain containing 4
0,94	WARS	tryptophanyl-tRNA synthetase
0,94	SLC5A1	solute carrier family 5 (sodium/glucose cotransporter), member 1
0,94	HNF4G	hepatocyte nuclear factor 4, gamma
0,94	EXO5	exonuclease 5
0,94	MAP6D1	MAP6 domain containing 1
0,94	CRISP2	cysteine-rich secretory protein 2
0,94	EIF2B4	eukaryotic translation initiation factor 2B, subunit 4 delta, 67kDa

0,94	ARHGAP21	Rho GTPase activating protein 21
0,94	GOLGA8F; GOLGA8G	golgin A8 family, member F; golgin A8 family, member G
0,94	PF4	platelet factor 4
0,94	ZNF799	zinc finger protein 799
0,94	HTR1E	5-hydroxytryptamine (serotonin) receptor 1E, G protein-coupled
0,94	C7orf61	chromosome 7 open reading frame 61
0,94	ZNF502	zinc finger protein 502
0,94	FAM13A	family with sequence similarity 13, member A
0,94	ANAPC10	anaphase promoting complex subunit 10
0,94	LINC01547	long intergenic non-protein coding RNA 1547
0,94	ZFP91-CNTF	ZFP91-CNTF readthrough (NMD candidate)
0,94	WDR6	Memczak2013 ANTISENSE, CDS, coding, INTERNAL best transcript NM_018031
0,94	POLE4	polymerase (DNA-directed), epsilon 4, accessory subunit
0,94	TSTD2	thiosulfate sulfurtransferase (rhodanese)-like domain containing 2
0,94	RHOQ	ras homolog family member Q
0,94	IFNE; MIR31HG	interferon, epsilon; MIR31 host gene
0,94	XXbac-BPG154L12.4; RNF5	putative novel transcript; Transcript Identified by AceView, Entrez Gene ID(s) 6048
0,94	OR52B4	olfactory receptor, family 52, subfamily B, member 4 (gene/pseudogene)
0,94	DHRS7C	dehydrogenase/reductase (SDR family) member 7C
0,94	DICER1	dicer 1, ribonuclease type III
0,94	DEFB132	defensin, beta 132
0,94	ZNF362	zinc finger protein 362
0,94	ABLIM2	actin binding LIM protein family, member 2
0,94	RPL30	ribosomal protein L30
0,94	DGKD	diacylglycerol kinase, delta 130kDa
0,94	FZD6	frizzled class receptor 6
0,94	MIPEP	mitochondrial intermediate peptidase
0,94	NR4A1	nuclear receptor subfamily 4, group A, member 1
0,94	ARFIP2	ADP-ribosylation factor interacting protein 2
0,94	C6orf229	chromosome 6 open reading frame 229
0,94	FSTL1; MIR198	follistatin like 1; microRNA 198
0,94	RBPMS2	RNA binding protein with multiple splicing 2
0,94	RETSAT	retinol saturase (all-trans-retinol 13,14-reductase)
0,94	ARPC4	actin related protein 2/3 complex subunit 4
0,94	CBS	cystathionine-beta-synthase
0,94	RP3-370M22.8; GRAP2	Transcript Identified by AceView, Entrez Gene ID(s) 9402; putative novel transcript
0,94	FAM83F	family with sequence similarity 83, member F
0,94	HES7	hes family bHLH transcription factor 7
0,94	MTMR12	myotubularin related protein 12
0,94	FAM187B	family with sequence similarity 187, member B
0,94	KRTAP12-2	keratin associated protein 12-2

0,94	CD300C	CD300c molecule
0,94	PDE4D	phosphodiesterase 4D, cAMP-specific
0,94	GPT	glutamic-pyruvate transaminase (alanine aminotransferase)
0,94	PCM1	pericentriolar material 1
0,94	MSX1	msh homeobox 1
0,94	LINC00934	long intergenic non-protein coding RNA 934
0,94	XRCC5	Transcript Identified by AceView, Entrez Gene ID(s) 7520
0,94	ASTN1	astrotactin 1
0,94	CSNK1G2-AS1	CSNK1G2 antisense RNA 1
0,94	NAPA	N-ethylmaleimide-sensitive factor attachment protein, alpha
0,94	CA13	carbonic anhydrase XIII
0,94	LSP1P3	lymphocyte-specific protein 1 pseudogene 3
0,94	BRD4	bromodomain containing 4
0,94	IHH	indian hedgehog
0,94	CEP89	centrosomal protein 89kDa
0,94	TMEM114	transmembrane protein 114
0,94	SMARCA1	SWI/SNF related, matrix associated, actin dependent regulator of chromatin, subfamily a, member 1
0,94	CHMP1B	charged multivesicular body protein 1B
0,94	SACS	sacsin molecular chaperone
0,94	SSX9	synovial sarcoma, X breakpoint 9
0,94	TRAF4	TNF receptor-associated factor 4
0,94	MCM10	minichromosome maintenance 10 replication initiation factor
0,94	MED27	mediator complex subunit 27
0,94	PRR12	proline rich 12
0,94	STAT6	signal transducer and activator of transcription 6, interleukin-4 induced
0,94	CNOT6L	CCR4-NOT transcription complex subunit 6-like
0,94	KRTAP19-2	keratin associated protein 19-2
0,94	IRF2BPL	interferon regulatory factor 2 binding protein-like
0,94	LRMP	lymphoid-restricted membrane protein
0,94	AUP1	ancient ubiquitous protein 1
0,94	FMN1	formin 1
0,94	ORMDL2	ORMDL sphingolipid biosynthesis regulator 2
0,94	GLRX5	glutaredoxin 5
0,94	TNFAIP8L1	tumor necrosis factor, alpha-induced protein 8-like 1
0,94	FLJ30679; RP11-463O9.8	uncharacterized protein FLJ30679; uncharacterized protein FLJ30679 [Source:EntrezGene;Acc:146512]; novel transcript
0,94	TCFL5	transcription factor-like 5 (basic helix-loop-helix)
0,94	ATG14	autophagy related 14
0,94	DUSP16	dual specificity phosphatase 16
0,94	CALB1	calbindin 1
0,94	MKRN2OS	MKRN2 opposite strand
0,94	MAML1	mastermind-like transcriptional coactivator 1
0,94	PRDM9	PR domain containing 9

0,94	KRT81	keratin 81, type II
0,94	ATP4B	ATPase, H+/K+ exchanging, beta polypeptide
0,94	GAPDHS	glyceraldehyde-3-phosphate dehydrogenase, spermatogenic
0,94	CAMP	cathelicidin antimicrobial peptide
0,94	ODF3	outer dense fiber of sperm tails 3
0,94	TMLHE	trimethyllysine hydroxylase, epsilon
0,94	CCL24	chemokine (C-C motif) ligand 24
0,94	LRRCC1	leucine rich repeat and coiled-coil centrosomal protein 1
0,94	AMMECR1	Alport syndrome, mental retardation, midface hypoplasia and elliptocytosis chromosomal region gene 1
0,94	OR10A6	olfactory receptor, family 10, subfamily A, member 6 (gene/pseudogene)
0,94	OR10A6	olfactory receptor family 10 subfamily A member 6 (gene/pseudogene) [Source:HGNC Symbol;Acc:HGNC:15132]
0,94	MKL2; TVP23CP2	MKL/myocardin-like 2; TVP23C pseudogene 2
0,94	PRC1	protein regulator of cytokinesis 1
0,94	HDAC7	histone deacetylase 7
0,94	HARS	histidyl-tRNA synthetase
0,94	RD3L	retinal degeneration 3-like
0,94	NOL4	nucleolar protein 4
0,94	TIFAB	TRAF-interacting protein with forkhead-associated domain, family member B
0,94	DNAJC9	DnaJ (Hsp40) homolog, subfamily C, member 9
0,94	SDR39U1	short chain dehydrogenase/reductase family 39U, member 1
0,94	KCNK18	potassium channel, two pore domain subfamily K, member 18
0,94	TIMM10	translocase of inner mitochondrial membrane 10 homolog (yeast)
0,94	DOCK1	dedicator of cytokinesis 1
0,94	TRIM27	Transcript Identified by AceView, Entrez Gene ID(s) 5987
0,94	PACRGL	PARK2 co-regulated like
0,94	TOP2A	topoisomerase (DNA) II alpha
0,94	LILRB1	leukocyte immunoglobulin-like receptor, subfamily B (with TM and ITIM domains), member 1
0,94	SCPEP1	serine carboxypeptidase 1
0,94	HOXC11	homeobox C11
0,94	TNFRSF1B; MIR4632; MIR7846	tumor necrosis factor receptor superfamily, member 1B; microRNA 4632; microRNA 7846
0,94	SPATA13; C1QTNF9	spermatogenesis associated 13; C1q and tumor necrosis factor related protein 9
0,94	RNF185	Transcript Identified by AceView, Entrez Gene ID(s) 91445
0,94	UBE3B	ubiquitin protein ligase E3B

0,94	HTR6	5-hydroxytryptamine (serotonin) receptor 6, G protein-coupled
0,94	MTUS1	microtubule associated tumor suppressor 1
0,94	CD63	CD63 molecule
0,94	SEMA5B	sema domain, seven thrombospondin repeats (type 1 and type 1-like), transmembrane domain (TM) and short cytoplasmic domain, (semaphorin) 5B
0,94	ASB17	ankyrin repeat and SOCS box containing 17
0,94	PHKB	phosphorylase kinase, beta
0,94	ST6GALNAC5	ST6 (alpha-N-acetyl-neuraminyl-2,3-beta-galactosyl-1,3)-N-acetylgalactosaminide alpha-2,6-sialyltransferase 5
0,94	NMRAL1	NmrA-like family domain containing 1
0,94	SPAM1	sperm adhesion molecule 1 (PH-20 hyaluronidase, zona pellucida binding)
0,94	LRIT3	leucine-rich repeat, immunoglobulin-like and transmembrane domains 3
0,94	PTGDS	prostaglandin D2 synthase 21kDa (brain)
0,94	AANAT	aralkylamine N-acetyltransferase
0,94	RXFP3	relaxin/insulin-like family peptide receptor 3
0,94	RFXAP	regulatory factor X-associated protein
0,94	RPS6KA1	ribosomal protein S6 kinase, 90kDa, polypeptide 1
0,94	ELN	elastin
0,94	OR5C1	olfactory receptor, family 5, subfamily C, member 1
0,94	RPP25L	ribonuclease P/MRP 25kDa subunit-like
0,94	SAT2	spermidine/spermine N1-acetyltransferase family member 2
0,94	ZNF432	zinc finger protein 432
0,94	IFIT1B	interferon-induced protein with tetratricopeptide repeats 1B
0,94	GTF3C3	general transcription factor IIIC subunit 3
0,94	INPP5F	inositol polyphosphate-5-phosphatase F
0,94	LINC01219	long intergenic non-protein coding RNA 1219
0,94	ZNF354A	zinc finger protein 354A
0,94	FBXL13	F-box and leucine-rich repeat protein 13
0,94	CAMLG	calcium modulating ligand
0,94	KCNS3	potassium voltage-gated channel, modifier subfamily S, member 3
0,94	NEMP1	nuclear envelope integral membrane protein 1
0,94	MRPL24	mitochondrial ribosomal protein L24
0,94	LINC01387	long intergenic non-protein coding RNA 1387
0,94	IAH1	isoamyl acetate-hydrolyzing esterase 1 homolog
0,94	ALDH2	aldehyde dehydrogenase 2 family (mitochondrial)
0,94	MYL9	myosin light chain 9
0,94	OTUD5	OTU deubiquitinase 5
0,94	ASCL1	achaete-scute family bHLH transcription factor 1
0,94	ZNF660	zinc finger protein 660
0,94	IMPACT	impact RWD domain protein
0,94	USP36	ubiquitin specific peptidase 36

0,94	CHST15	carbohydrate (N-acetylgalactosamine 4-sulfate 6-O) sulfotransferase 15
0,94	CYB5D2	cytochrome b5 domain containing 2
0,94	IL21	interleukin 21
0,94	GORAB	golgin, RAB6-interacting
0,94	ADAMTS2	ADAM metallopeptidase with thrombospondin type 1 motif 2
0,94	TARBP1	TAR (HIV-1) RNA binding protein 1
0,94	IFITM1	interferon induced transmembrane protein 1
0,94	OR8K1	olfactory receptor, family 8, subfamily K, member 1
0,94	TTF1	transcription termination factor, RNA polymerase I
0,94	ANKRD10	ankyrin repeat domain 10
0,94	ADGRF5	adhesion G protein-coupled receptor F5
0,94	CCDC171	coiled-coil domain containing 171
0,94	ZNF32	zinc finger protein 32
0,94	KIF3A	kinesin family member 3A
0,94	LRRC37A11P	leucine rich repeat containing 37, member A11, pseudogene
0,94	CENPU	centromere protein U
0,94	G6PC3	glucose 6 phosphatase, catalytic, 3
0,94	PCYT1A	phosphate cytidylyltransferase 1, choline, alpha
0,94	ABHD14A	abhydrolase domain containing 14A
0,94	GTF2A1	general transcription factor IIA 1
0,94	RAB1A	RAB1A, member RAS oncogene family
0,94	SLC11A1	solute carrier family 11 (proton-coupled divalent metal ion transporter), member 1
0,94	ABLIM3	actin binding LIM protein family, member 3
0,94	FBXO45	F-box protein 45
0,94	ZRSR2	zinc finger (CCCH type), RNA binding motif and serine/arginine rich 2
0,94	ZNF716	zinc finger protein 716
0,94	HIST1H4L	histone cluster 1, H4l
0,94	HMG20B	high mobility group 20B
0,94	ZSCAN30	zinc finger and SCAN domain containing 30
0,94	CHRAC1	Transcript Identified by AceView, Entrez Gene ID(s) 54108
0,94	MGAT4A	mannosyl (alpha-1,3-)-glycoprotein beta-1,4-N-acetylglucosaminyltransferase, isozyme A
0,94	ZNF286B; FOXO3B	zinc finger protein 286B; forkhead box O3B pseudogene
0,94	CAPS	calcyphosine
0,94	HNF4A	hepatocyte nuclear factor 4, alpha
0,94	COL4A2	collagen, type IV, alpha 2
0,94	PIP4K2A	phosphatidylinositol-5-phosphate 4-kinase, type II, alpha
0,94	TTC1	tetratricopeptide repeat domain 1
0,94	CSTF3	cleavage stimulation factor, 3 pre-RNA, subunit 3
0,94	FAM166B	family with sequence similarity 166, member B
0,94	HOXB8	homeobox B8
0,94	MGST2	microsomal glutathione S-transferase 2

0,94	HAUS7; TREX2	HAUS augmin like complex subunit 7; three prime repair exonuclease 2
0,94	SLK	STE20-like kinase
0,94	KRTAP5-1	keratin associated protein 5-1
0,94	CT47A5; CT47A2; CT47A1; CT47A11; CT47A12	cancer/testis antigen family 47, member A5; cancer/testis antigen family 47, member A2; cancer/testis antigen family 47, member A1; cancer/testis antigen family 47, member A11; cancer/testis antigen family 47, member A12
0,94	SLC39A9	solute carrier family 39, member 9
0,94	MEX3B	mex-3 RNA binding family member B
0,94	CYP4X1	cytochrome P450, family 4, subfamily X, polypeptide 1
0,94	FUT6	fucosyltransferase 6 (alpha (1,3) fucosyltransferase)
0,94	GRIP2	glutamate receptor interacting protein 2
0,94	KIF15	kinesin family member 15
0,94	ELAVL4	ELAV like neuron-specific RNA binding protein 4
0,94	KRTAP13-4	keratin associated protein 13-4
0,94	ZNF92	zinc finger protein 92
0,94	FNIP2	folliculin interacting protein 2
0,94	CHRNA6	cholinergic receptor, nicotinic alpha 6
0,94	NDRG3	NDRG family member 3
0,94	BRCA2	breast cancer 2, early onset
0,94	GEN1	GEN1 Holliday junction 5 flap endonuclease
0,94	SLC35A2	solute carrier family 35 (UDP-galactose transporter), member A2
0,94	KIAA0040	KIAA0040
0,94	LOC100129940; flanu; RP11-366L20.2	uncharacterized LOC100129940; Transcript Identified by AceView; novel transcript, antisense to HMGA2
0,94	GPRC5B	G protein-coupled receptor, class C, group 5, member B
0,94	IL3RA	interleukin 3 receptor, alpha (low affinity)
0,94	MSH3	mutS homolog 3
0,94	STAB1	stabilin 1
0,94	TRAPPC3L	trafficking protein particle complex 3-like
0,94	ZNF37A	zinc finger protein 37A
0,94	GRIA3	glutamate receptor, ionotropic, AMPA 3
0,94	ACTL7B	actin-like 7B
0,94	SSR4	signal sequence receptor, delta
0,94	PDCD2L	programmed cell death 2-like
0,94	ECE1	endothelin converting enzyme 1
0,94	CRTAC1	cartilage acidic protein 1
0,94	AKR7A3	aldo-keto reductase family 7, member A3 (aflatoxin aldehyde reductase)
0,94	MMS19	MMS19 homolog, cytosolic iron-sulfur assembly component
0,94	TMEM40	transmembrane protein 40
0,94	ATE1	arginyltransferase 1
0,94	SIM1	single-minded family bHLH transcription factor 1

0,94	MNT	MAX network transcriptional repressor
0,94	KLHL8	kelch-like family member 8
0,94	FCGR2A	Fc fragment of IgG, low affinity IIa, receptor (CD32)
0,94	GPAM	glycerol-3-phosphate acyltransferase, mitochondrial
0,94	MTRF1L	mitochondrial translational release factor 1-like
0,94	ZNF780B	zinc finger protein 780B
0,94	VARS2	valyl-tRNA synthetase 2, mitochondrial
0,94	CUL1	cullin 1
0,94	PYY	peptide YY
0,94	KCNC3	potassium channel, voltage gated Shaw related subfamily C, member 3
0,94	APC	adenomatous polyposis coli
0,94	TBL3	transducin (beta)-like 3
0,94	SH2B2	Memczak2013 ANTISENSE, CDS, coding, INTERNAL best transcript NM_020979
0,94	SLC35E2	solute carrier family 35, member E2
0,94	RPS25	ribosomal protein S25
0,94	PPP1R9A	protein phosphatase 1, regulatory subunit 9A
0,94	PDSS2	prenyl (decaprenyl) diphosphate synthase, subunit 2
0,94	MIA; RAB4B; MIA-RAB4B	melanoma inhibitory activity; RAB4B, member RAS oncogene family; MIA-RAB4B readthrough (NMD candidate)
0,94	PRIM2	primase, DNA, polypeptide 2 (58kDa)
0,94	CHRNA7	cholinergic receptor, nicotinic alpha 7
0,94	FCRL3	Fc receptor-like 3
0,94	DNAJB9	DnaJ (Hsp40) homolog, subfamily B, member 9
0,94	ETS1	v-ets avian erythroblastosis virus E26 oncogene homolog 1
0,94	OCEL1	occludin/ELL domain containing 1
0,94	CAMKK2	calcium/calmodulin-dependent protein kinase kinase 2, beta
0,94	ANKRD17	ankyrin repeat domain 17
0,94	ZNF12	zinc finger protein 12
0,94	GNB4	guanine nucleotide binding protein (G protein), beta polypeptide 4
0,94	MRPS11	mitochondrial ribosomal protein S11
0,94	MRPL45	mitochondrial ribosomal protein L45
0,94	RPLP0	ribosomal protein, large, P0
0,94	METTL4	methyltransferase like 4
0,94	C6orf89	chromosome 6 open reading frame 89
0,94	CD320	CD320 molecule
0,94	PRMT6	protein arginine methyltransferase 6
0,94	ZCCHC10	zinc finger, CCHC domain containing 10
0,94	CBLN4	cerebellin 4 precursor
0,94	PEX5	peroxisomal biogenesis factor 5
0,94	MYO1C	myosin IC
0,94	NAMPT	nicotinamide phosphoribosyltransferase
0,94	GLTP	glycolipid transfer protein
0,94	PLK4	polo-like kinase 4
0,94	SEC11A	SEC11 homolog A, signal peptidase complex subunit

0,94	NPY4R; CH17-360D5.1; PPYR1	Homo sapiens neuropeptide Y receptor Y4 (NPY4R), transcript variant 2, mRNA.; neuropeptide Y receptor type 4-like; Homo sapiens pancreatic polypeptide receptor 1, mRNA (cDNA clone MGC:116895 IMAGE:40005502), complete cds.; Homo sapiens pancreatic polypeptide receptor 1, mRNA (cDNA clone MGC:116897 IMAGE:40005506), complete cds.; novel transcript
0,94	SNRNP48	small nuclear ribonucleoprotein, U11/U12 48KDa subunit
0,94	PDCL	phosducin like
0,94	DBT	dihydrolipoamide branched chain transacylase E2
0,94	CAST	calpastatin
0,94	EPHA2	EPH receptor A2
0,94	CUL7	cullin 7
0,94	ARMC10	armadillo repeat containing 10
0,94	E2F1	E2F transcription factor 1
0,94	APOBEC3D	apolipoprotein B mRNA editing enzyme, catalytic polypeptide-like 3D
0,94	SMG1	SMG1 phosphatidylinositol 3-kinase-related kinase
0,94	PHF3	PHD finger protein 3
0,94	KDELR3	KDEL (Lys-Asp-Glu-Leu) endoplasmic reticulum protein retention receptor 3
0,94	KIF11	kinesin family member 11
0,94	ST6GALNAC3	ST6 (alpha-N-acetyl-neuraminyl-2,3-beta-galactosyl-1,3)-N-acetylgalactosaminide alpha-2,6-sialyltransferase 3
0,94	AKIP1	A kinase (PRKA) interacting protein 1
0,94	XCR1	chemokine (C motif) receptor 1
0,94	HSF2	heat shock transcription factor 2
0,94	SENP8	SUMO/sentrin peptidase family member, NEDD8 specific
0,94	DYRK3	dual specificity tyrosine-(Y)-phosphorylation regulated kinase 3
0,94	PLEKHG7	pleckstrin homology domain containing, family G (with RhoGef domain) member 7
0,94	CYB5RL	cytochrome b5 reductase-like
0,94	S100A5	S100 calcium binding protein A5
0,94	LACRT	lacritin
0,94	RAB3IP	RAB3A interacting protein
0,94	MSMB	microseminoprotein, beta-
0,94	LIPA	Transcript Identified by AceView, Entrez Gene ID(s) 3988
0,94	ACSL5	acyl-CoA synthetase long-chain family member 5
0,94	RAB42	RAB42, member RAS oncogene family
0,94	SRRM1	serine/arginine repetitive matrix 1
0,94	LINC01219	long intergenic non-protein coding RNA 1219
0,94	CCDC28B	coiled-coil domain containing 28B
0,94	LEFTY1	left-right determination factor 1
0,94	KIAA1147	KIAA1147
0,94	PANX2	pannexin 2

0,94	PDK4	pyruvate dehydrogenase kinase, isozyme 4
0,94	JSRP1	junctional sarcoplasmic reticulum protein 1
0,94	POLR2M	polymerase (RNA) II (DNA directed) polypeptide M
0,94	SLC30A9	solute carrier family 30 (zinc transporter), member 9
0,94	GLIPR2	GLI pathogenesis-related 2
0,94	ARPP21	Transcript Identified by AceView, Entrez Gene ID(s) 10777
0,94	CUL3	cullin 3
0,94	CHTF8	Jeck2013 ANTISENSE, coding, INTERNAL, OVEXON, UTR3 best transcript NM_001039690
0,94	MBD5	methyl-CpG binding domain protein 5
0,94	ARL3	ADP-ribosylation factor like GTPase 3
0,94	PDE10A	phosphodiesterase 10A
0,94	KIAA1429	KIAA1429
0,94	BIN1	bridging integrator 1
0,94	CXorf58	chromosome X open reading frame 58
0,94	PGM2	phosphoglucomutase 2
0,94	HDHD2	haloacid dehalogenase-like hydrolase domain containing 2
0,94	HSD17B14	hydroxysteroid (17-beta) dehydrogenase 14
0,94	SLC37A4	solute carrier family 37 (glucose-6-phosphate transporter), member 4
0,94	CMTM8	CKLF-like MARVEL transmembrane domain containing 8
0,94	KCNJ10	potassium channel, inwardly rectifying subfamily J, member 10
0,94	SLN	sarcolipin
0,94	PSG7	pregnancy specific beta-1-glycoprotein 7 (gene/pseudogene)
0,94	HNRNPCL2	heterogeneous nuclear ribonucleoprotein C-like 2
0,94	SLC25A4	solute carrier family 25 (mitochondrial carrier; adenine nucleotide translocator), member 4
0,94	ARHGAP19-SLIT1	ARHGAP19-SLIT1 readthrough (NMD candidate)
0,94	MYL3	myosin, light chain 3, alkali; ventricular, skeletal, slow
0,94	RNF123	ring finger protein 123
0,94	CYP2D6	cytochrome P450, family 2, subfamily D, polypeptide 6
0,94	RPS11; SNORD35B	ribosomal protein S11; small nucleolar RNA, C/D box 35B
0,94	MED21	mediator complex subunit 21
0,94	SIL1	SIL1 nucleotide exchange factor
0,94	FAM92A1	family with sequence similarity 92, member A1
0,94	ZNF330	zinc finger protein 330
0,94	CBR1	carbonyl reductase 1
0,94	ZNF7	zinc finger protein 7
0,94	ANGPTL7	angiopoietin like 7
0,94	ERBB3	erb-b2 receptor tyrosine kinase 3
0,94	ABCB10	ATP binding cassette subfamily B member 10

0,94	CERKL	ceramide kinase like
0,94	FAM71A	family with sequence similarity 71, member A
0,94	TMTC2	transmembrane and tetratricopeptide repeat containing 2
0,94	CYP4Z1	cytochrome P450, family 4, subfamily Z, polypeptide 1
0,94	NAV3	neuron navigator 3
0,94	LIPT1	lipoyltransferase 1
0,94	OR10A6	olfactory receptor, family 10, subfamily A, member 6 (gene/pseudogene)
0,94	GALNT8	polypeptide N-acetylgalactosaminyltransferase 8
0,94	C3orf58	chromosome 3 open reading frame 58
0,94	RYK	receptor-like tyrosine kinase
0,94	GLYATL2	glycine-N-acyltransferase-like 2
0,94	FOXI2	forkhead box I2
0,94	TRMT10A	tRNA methyltransferase 10A
0,94	UTS2B	urotensin 2B
0,94	OR5AL1	olfactory receptor, family 5, subfamily AL, member 1 (gene/pseudogene)
0,94	PYCR1	pyrroline-5-carboxylate reductase 1
0,94	CMTR1	cap methyltransferase 1
0,94	IGF2-AS	IGF2 antisense RNA
0,94	DCAF12L2	DDB1 and CUL4 associated factor 12-like 2
0,94	DPYSL3	dihydropyrimidinase-like 3
0,94	SF3B1	splicing factor 3b, subunit 1, 155kDa
0,94	INO80B-WBP1	INO80B-WBP1 readthrough (NMD candidate)
0,94	C16orf78	chromosome 16 open reading frame 78
0,94	STAM	Memczak2013 ALT_ACCEPTOR, ALT_DONOR, INTERNAL, intronic, ncRNA best transcript NR_037774
0,94	TREM2	triggering receptor expressed on myeloid cells 2
0,94	TDRD1	tudor domain containing 1
0,94	RP4-728D4.2; PSMB2	novel transcript; Transcript Identified by AceView, Entrez Gene ID(s) 5690
0,94	GPR144; ADGRD2	Transcript Identified by AceView, Entrez Gene ID(s) 347088; adhesion G protein-coupled receptor D2 [Source:HGNC Symbol;Acc:HGNC:18651]
0,94	GNA11	guanine nucleotide binding protein (G protein), alpha 11 (Gq class)
0,94	NFIA	nuclear factor I/A
0,94	PARL	presenilin associated, rhomboid-like
0,94	MAEA	macrophage erythroblast attacher
0,94	SUSD5	sushi domain containing 5
0,94	MDFI	MyoD family inhibitor
0,94	NUBPL	nucleotide binding protein-like
0,94	NDST2	N-deacetylase/N-sulfotransferase (heparan glucosaminyl) 2
0,94	TBC1D16	TBC1 domain family, member 16
0,94	DCPS	decapping enzyme, scavenger
0,94	DENND1B	DENN/MADD domain containing 1B

0,94	LYRM7	LYR motif containing 7
0,94	NDUFAF5	NADH dehydrogenase (ubiquinone) complex I, assembly factor 5
0,94	TENM4	teneurin transmembrane protein 4
0,94	SEL1L2	sel-1 suppressor of lin-12-like 2 (C. elegans)
0,94	ARPC3	actin related protein 2/3 complex subunit 3
0,94	TLE2	transducin-like enhancer of split 2
0,94	MAP3K4	mitogen-activated protein kinase kinase kinase 4
0,94	HCAR1	hydroxycarboxylic acid receptor 1
0,94	SPATA4	spermatogenesis associated 4
0,94	IKBIP	IKBKB interacting protein
0,94	USP17L18; USP17L11	ubiquitin specific peptidase 17-like family member 18; ubiquitin specific peptidase 17-like family member 11
0,94	RAB41	RAB41, member RAS oncogene family
0,94	SPAG16	sperm associated antigen 16
0,94	GTF2A1L; STON1-GTF2A1L; STON1	general transcription factor IIA 1-like; STON1-GTF2A1L readthrough; stonin 1
0,94	GPRC5C	G protein-coupled receptor, class C, group 5, member C
0,94	LOC441155	zinc finger CCCH-type domain-containing-like
0,94	MMP23B; MMP23A	matrix metallopeptidase 23B; matrix metallopeptidase 23A (pseudogene)
0,94	SMARCC2	SWI/SNF related, matrix associated, actin dependent regulator of chromatin, subfamily c, member 2
0,94	OR10Z1	olfactory receptor, family 10, subfamily Z, member 1
0,94	PLOD2	procollagen-lysine, 2-oxoglutarate 5-dioxygenase 2
0,94	ARMC2	armadillo repeat containing 2
0,94	SHBG	sex hormone-binding globulin
0,94	FADS1; MIR1908	fatty acid desaturase 1; microRNA 1908
0,94	DEF6	Memczak2013 ANTISENSE, CDS, coding, INTERNAL best transcript NM_022047
0,94	ALG9	ALG9, alpha-1,2-mannosyltransferase
0,94	NPIPB15	nuclear pore complex interacting protein family, member B15
0,94	WDR24	WD repeat domain 24
0,94	SLC17A1	solute carrier family 17 (organic anion transporter), member 1
0,94	CST4	cystatin S
0,94	KATNB1	katanin p80 (WD repeat containing) subunit B 1
0,94	APCDD1	adenomatosis polyposis coli down-regulated 1
0,94	ZBED6; ZC3H11A	zinc finger, BED-type containing 6; zinc finger CCCH-type containing 11A
0,94	VANGL2	VANGL planar cell polarity protein 2
0,94	POP5	POP5 homolog, ribonuclease P/MRP subunit
0,94	TRIM61	tripartite motif containing 61
0,94	FGFR2	fibroblast growth factor receptor 2

0,94	ATP6V1H	ATPase, H+ transporting, lysosomal 50/57kDa, V1 subunit H
0,94	CCDC12	coiled-coil domain containing 12
0,94	OR10K2	olfactory receptor, family 10, subfamily K, member 2
0,94	NFYB	nuclear transcription factor Y subunit beta
0,94	RPARP-AS1	RPARP antisense RNA 1
0,94	OR52E4	olfactory receptor, family 52, subfamily E, member 4
0,94	TTC23	tetratricopeptide repeat domain 23
0,94	ADAMTS13	ADAM metallopeptidase with thrombospondin type 1 motif 13
0,94	ZNF234	zinc finger protein 234
0,94	GRIPAP1	GRIP1 associated protein 1
0,94	CPEB1	cytoplasmic polyadenylation element binding protein 1
0,94	EFNA4	ephrin-A4
0,94	NYX	nyctalopin
0,94	PTP4A1	protein tyrosine phosphatase type IVA, member 1
0,94	ZFP69	ZFP69 zinc finger protein
0,94	PPP1R37	protein phosphatase 1, regulatory subunit 37
0,94	MYLPF	myosin light chain, phosphorylatable, fast skeletal muscle
0,94	XPNPEP2	X-prolyl aminopeptidase (aminopeptidase P) 2, membrane-bound
0,94	COX7B2	cytochrome c oxidase subunit VIIb2
0,94	C10orf111	chromosome 10 open reading frame 111
0,94	AACSP1	acetoacetyl-CoA synthetase pseudogene 1
0,94	NMNAT3	nicotinamide nucleotide adenylyltransferase 3
0,94	ITIH5	inter-alpha-trypsin inhibitor heavy chain family, member 5
0,94	TNNC1	troponin C type 1 (slow)
0,94	MAGED1	MAGE family member D1
0,94	SH3RF2	SH3 domain containing ring finger 2
0,94	PRR33	proline rich 33
0,94	TLR7	toll-like receptor 7
0,94	SEC24B	SEC24 homolog B, COPII coat complex component
0,94	MSANTD4	Myb/SANT-like DNA-binding domain containing 4 with coiled-coils
0,94	CEP120	centrosomal protein 120kDa
0,94	CENPK	centromere protein K
0,94	MIER1	mesoderm induction early response 1, transcriptional regulator
0,94	PKD1L1	polycystic kidney disease 1 like 1
0,94	ARHGEF9; ARHGEF9-IT1	Cdc42 guanine nucleotide exchange factor 9; ARHGEF9 intronic transcript 1
0,94	FBXO21	F-box protein 21
0,94	DCD	dermcidin
0,94	PI4KAP1	phosphatidylinositol 4-kinase, catalytic, alpha pseudogene 1
0,94	LCA5	Leber congenital amaurosis 5

0,94	TMEM110-MUSTN1; TMEM110	TMEM110-MUSTN1 readthrough; transmembrane protein 110
0,94	RPL41	Synthetic construct Homo sapiens clone IMAGE:100063377, MGC:190767 ribosomal protein L41 (RPL41) mRNA, encodes complete protein.; 60S ribosomal protein L41 [Source:UniProtKB/Swiss-Prot;Acc:P62945]
0,94	SLC12A5	solute carrier family 12 (potassium/chloride transporter), member 5
0,94	ZNF280D	zinc finger protein 280D
0,94	HYOU1	hypoxia up-regulated 1
0,94	PIP4K2C	phosphatidylinositol-5-phosphate 4-kinase, type II, gamma
0,94	VPS11	VPS11, CORVET/HOPS core subunit [Source:HGNC Symbol;Acc:HGNC:14583]
0,94	ZNF835	zinc finger protein 835
0,94	KIAA1191	KIAA1191
0,94	WNT8A	wingless-type MMTV integration site family, member 8A
0,94	OR5H2	olfactory receptor, family 5, subfamily H, member 2
0,94	IKZF3	IKAROS family zinc finger 3
0,94	OR6B3	olfactory receptor, family 6, subfamily B, member 3
0,94	MOB3C	MOB kinase activator 3C
0,94	ZSCAN16	zinc finger and SCAN domain containing 16
0,94	TLDC1	TBC/LysM-associated domain containing 1
0,94	RPL7A; SNORD36C; SNORD36B; SNORD24; SNORD36A	ribosomal protein L7a; small nucleolar RNA, C/D box 36C; small nucleolar RNA, C/D box 36B; small nucleolar RNA, C/D box 24; small nucleolar RNA, C/D box 36A
0,94	DMTN	dematin actin binding protein
0,94	SDF2	stromal cell-derived factor 2
0,94	MYT1L	Transcript Identified by AceView, Entrez Gene ID(s) 23040
0,94	SARNP	SAP domain containing ribonucleoprotein
0,94	NAT1	N-acetyltransferase 1 (arylamine N-acetyltransferase)
0,94	RNASEH2A	ribonuclease H2, subunit A
0,94	PLXDC2	plexin domain containing 2
0,94	JOSD2	Josephin domain containing 2
0,94	KIFC3	Jeck2013 ANTISENSE, coding, INTERNAL, intronic best transcript NM_001130100
0,94	CSNK2A2	casein kinase 2, alpha prime polypeptide
0,94	HTR5A	5-hydroxytryptamine (serotonin) receptor 5A, G protein-coupled
0,94	URB2	URB2 ribosome biogenesis 2 homolog (S. cerevisiae)
0,94	C3orf80	chromosome 3 open reading frame 80
0,94	GOLGA1	golgin A1
0,94	ACTR1B	ARP1 actin-related protein 1 homolog B, centractin beta (yeast)
0,94	FAM132A	family with sequence similarity 132, member A

0,94	TUBG2	tubulin, gamma 2
0,94	SCRN3	secernin 3
0,94	ZFR	zinc finger RNA binding protein
0,94	GTF3C4	general transcription factor IIIC subunit 4
0,94	LOXL4	lysyl oxidase-like 4
0,94	CLIC4	chloride intracellular channel 4
0,94	MISP	mitotic spindle positioning
0,94	C7orf72	chromosome 7 open reading frame 72
0,94	LARP4B	La ribonucleoprotein domain family, member 4B
0,94	FTMT	ferritin mitochondrial
0,94	CD1D	CD1d molecule
0,94	CXorf67	chromosome X open reading frame 67
0,94	MYOD1	myogenic differentiation 1
0,94	HELZ	helicase with zinc finger
0,94	ADIPOQ	adiponectin, C1Q and collagen domain containing
0,94	FAM160B2	family with sequence similarity 160, member B2
0,94	PFDN6; MIR6834	prefoldin subunit 6; microRNA 6834
0,94	SBF1	SET binding factor 1
0,94	KCNK15	potassium channel, two pore domain subfamily K, member 15
0,94	SUDS3	SDS3 homolog, SIN3A corepressor complex component
0,94	ARHGEF4	Rho guanine nucleotide exchange factor 4
0,94	FAM78A	family with sequence similarity 78, member A
0,94	GJA10	gap junction protein alpha 10
0,94	sep-01	septin 1
0,94	C2orf27B	chromosome 2 open reading frame 27B
0,94	ZNF236	zinc finger protein 236
0,94	SERPINA6	serpin peptidase inhibitor, clade A (alpha-1 antiproteinase, antitrypsin), member 6
0,94	PAFAH1B1	platelet-activating factor acetylhydrolase 1b, regulatory subunit 1 (45kDa)
0,94	TMEM229B	transmembrane protein 229B
0,94	RECK	reversion-inducing-cysteine-rich protein with kazal motifs
0,94	sep-14	septin 14
0,94	VPRBP	Vpr (HIV-1) binding protein
0,94	MICA	MHC class I polypeptide-related sequence A
0,94	DNMT3A	DNA (cytosine-5-)-methyltransferase 3 alpha
0,94	ZNF211	zinc finger protein 211
0,94	IRF9	interferon regulatory factor 9
0,94	CA7	carbonic anhydrase VII
0,94	LRRK1	leucine-rich repeat kinase 1
0,94	LCE5A	late cornified envelope 5A
0,94	SEMA4F	sema domain, immunoglobulin domain (Ig), transmembrane domain (TM) and short cytoplasmic domain, (semaphorin) 4F
0,94	DNM1	dynamin 1
0,94	CDK13	cyclin-dependent kinase 13

0,94	GMEB2	glucocorticoid modulatory element binding protein 2
0,94	ATG101	autophagy related 101
0,94	SMAD2	SMAD family member 2
0,94	MNX1	motor neuron and pancreas homeobox 1
0,94	GOLGA8J	golgin A8 family, member J
0,94	VSX1	visual system homeobox 1
0,94	GAL3ST2	galactose-3-O-sulfotransferase 2
0,94	CCDC84	coiled-coil domain containing 84
0,94	PI4K2B	phosphatidylinositol 4-kinase type 2 beta
0,94	EXOG	endo/exonuclease (5-3), endonuclease G-like
0,94	NBPF26	neuroblastoma breakpoint family, member 26
0,94	OR5K4	olfactory receptor, family 5, subfamily K, member 4
0,94	BTG2	BTG family, member 2
0,94	BIVM	basic, immunoglobulin-like variable motif containing
0,94	LOC101929319; TNFAIP6	uncharacterized LOC101929319; Salzman2013 ANTISENSE, CDS, coding, INTERNAL, intronic, OVCODE, OVEXON best transcript NM_007115
0,94	MRPL55	mitochondrial ribosomal protein L55
0,94	SH3GLB1	SH3-domain GRB2-like endophilin B1
0,93	TRAF2	TNF receptor-associated factor 2
0,93	DERL3	derlin 3
0,93	ZNF385D	zinc finger protein 385D
0,93	P3H2	prolyl 3-hydroxylase 2
0,93	ITGB6	integrin beta 6
0,93	LAMC1	laminin, gamma 1 (formerly LAMB2)
0,93	HIST1H1B	histone cluster 1, H1b
0,93	NDUFA4L2	NADH dehydrogenase (ubiquinone) 1 alpha subcomplex, 4-like 2
0,93	CLCNKA	chloride channel, voltage-sensitive Ka
0,93	C12orf49	chromosome 12 open reading frame 49
0,93	ARHGAP17	Memczak2013 ALT_ACCEPTOR, ALT_DONOR, coding, INTERNAL, intronic best transcript NM_001006634
0,93	CTAGE5	CTAGE family, member 5
0,93	SIGLEC1	sialic acid binding Ig-like lectin 1, sialoadhesin
0,93	POGZ	pogo transposable element with ZNF domain
0,93	TCEAL2	transcription elongation factor A (SII)-like 2
0,93	B3GAT3	beta-1,3-glucuronyltransferase 3
0,93	TAPT1	transmembrane anterior posterior transformation 1
0,93	TRIP12	Transcript Identified by AceView, Entrez Gene ID(s) 9320
0,93	PCSK2	Transcript Identified by AceView, Entrez Gene ID(s) 5126
0,93	CEP192	centrosomal protein 192kDa
0,93	TTC7A	tetratricopeptide repeat domain 7A
0,93	CYP2E1	cytochrome P450, family 2, subfamily E, polypeptide 1
0,93	RBBP7	retinoblastoma binding protein 7
0,93	ARL9	ADP-ribosylation factor like GTPase 9
0,93	FAIM	Fas apoptotic inhibitory molecule

0,93	FAM103A1	family with sequence similarity 103, member A1
0,93	CHD4	chromodomain helicase DNA binding protein 4
0,93	COQ10A	coenzyme Q10A
0,93	TBX10	T-box 10
0,93	ZNF511	zinc finger protein 511
0,93	OR4S1	olfactory receptor, family 4, subfamily S, member 1
0,93	DUSP6	dual specificity phosphatase 6
0,93	CLDN17	claudin 17
0,93	POMK	protein-O-mannose kinase
0,93	PPP2R2C	protein phosphatase 2, regulatory subunit B, gamma
0,93	GTF2IRD2B	GTF2I repeat domain containing 2B
0,93	FAM134A	family with sequence similarity 134, member A
0,93	AC010894.3; SCRN3	novel transcript; Transcript Identified by AceView, Entrez Gene ID(s) 79634
0,93	RAET1E	retinoic acid early transcript 1E
0,93	TRIM69	tripartite motif containing 69
0,93	ATP6AP1	ATPase, H+ transporting, lysosomal accessory protein 1
0,93	PODN	podocan
0,93	FAM193A	family with sequence similarity 193, member A
0,93	PHGR1	proline/histidine/glycine-rich 1
0,93	GSTO1	glutathione S-transferase omega 1
0,93	ABCA3	ATP binding cassette subfamily A member 3
0,93	MRO	maestro
0,93	ENOSF1	enolase superfamily member 1
0,93	FAM195B	family with sequence similarity 195, member B
0,93	PCNA	proliferating cell nuclear antigen
0,93	SH2D2A	SH2 domain containing 2A
0,93	ABHD6	abhydrolase domain containing 6
0,93	NUDT16L1	nudix hydrolase 16 like 1
0,93	DHX34	DEAH (Asp-Glu-Ala-His) box polypeptide 34
0,93	AQP3	aquaporin 3 (Gill blood group)
0,93	TSHZ1	teashirt zinc finger homeobox 1
0,93	PLEKHA3	pleckstrin homology domain containing, family A (phosphoinositide binding specific) member 3
0,93	ARFGEF1	ADP-ribosylation factor guanine nucleotide-exchange factor 1 (brefeldin A-inhibited)
0,93	CDKN1B	cyclin-dependent kinase inhibitor 1B (p27, Kip1)
0,93	RAB38	RAB38, member RAS oncogene family
0,93	HGSNAT	Transcript Identified by AceView, Entrez Gene ID(s) 138050
0,93	RUNDC3B	RUN domain containing 3B
0,93	HMGN3	high mobility group nucleosomal binding domain 3
0,93	SERTM1	serine-rich and transmembrane domain containing 1
0,93	FBL	fibrillarin
0,93	C5orf24	chromosome 5 open reading frame 24
0,93	PRPSAP1	phosphoribosyl pyrophosphate synthetase-associated protein 1
0,93	GLS2	glutaminase 2 (liver, mitochondrial)

0,93	CHCHD5	coiled-coil-helix-coiled-coil-helix domain containing 5
0,93	NCR3LG1	natural killer cell cytotoxicity receptor 3 ligand 1
0,93	ZNF653	zinc finger protein 653
0,93	CRMP1	collapsin response mediator protein 1
0,93	CFHR4	complement factor H-related 4
0,93	HKDC1	hexokinase domain containing 1
0,93	TTC21A; MIR6822	tetratricopeptide repeat domain 21A; microRNA 6822
0,93	KRT28	keratin 28, type I
0,93	FAM104B	family with sequence similarity 104, member B
0,93	IDI2	isopentenyl-diphosphate delta isomerase 2
0,93	NCSTN	nicastrin
0,93	PCED1A	PC-esterase domain containing 1A
0,93	NBPF14	neuroblastoma breakpoint family, member 14
0,93	TEX29	testis expressed 29
0,93	PSG9	pregnancy specific beta-1-glycoprotein 9
0,93	C1orf123	chromosome 1 open reading frame 123
0,93	LRR1; RHOQP1	leucine rich repeat protein 1; ras homolog family member Q pseudogene 1
0,93	SERTAD1	SERTA domain containing 1
0,93	NRDE2	NRDE-2, necessary for RNA interference, domain containing
0,93	MZT2A; MIR4784	mitotic spindle organizing protein 2A; microRNA 4784
0,93	TRAPPC2L	trafficking protein particle complex 2-like
0,93	TNRC6B	trinucleotide repeat containing 6B
0,93	TMEM52B	transmembrane protein 52B
0,93	KIF21B	kinesin family member 21B
0,93	MEF2A	myocyte enhancer factor 2A
0,93	TECTB	tectorin beta
0,93	EIF2B3	eukaryotic translation initiation factor 2B, subunit 3 gamma, 58kDa
0,93	CREBZF	CREB/ATF bZIP transcription factor
0,93	FAM103A1	Homo sapiens family with sequence similarity 103, member A1, mRNA (cDNA clone MGC:2560 IMAGE:2989772), complete cds.; Homo sapiens family with sequence similarity 103, member A1, mRNA (cDNA clone MGC:18029 IMAGE:3924570), complete cds.; Homo sapiens family with sequence similarity 103, member A1, mRNA (cDNA clone MGC:102778 IMAGE:5578103), complete cds.
0,93	RTEL1; TNFRSF6B; RTEL1-TNFRSF6B	regulator of telomere elongation helicase 1; tumor necrosis factor receptor superfamily, member 6b, decoy; RTEL1-TNFRSF6B readthrough (NMD candidate)
0,93	FAM101A	family with sequence similarity 101, member A
0,93	MRPL33	mitochondrial ribosomal protein L33
0,93	GLRA3	glycine receptor alpha 3
0,93	BAZ1A	bromodomain adjacent to zinc finger domain 1A
0,93	LINC00346	long intergenic non-protein coding RNA 346

0,93	ZNF22	zinc finger protein 22
0,93	DPY19L1	dpy-19-like 1 (C. elegans)
0,93	SCCPDH	saccharopine dehydrogenase (putative)
0,93	FBXO10	F-box protein 10
0,93	ZMYM2	zinc finger, MYM-type 2
0,93	ING1	inhibitor of growth family member 1
0,93	TCIRG1	T-cell, immune regulator 1, ATPase, H+ transporting, lysosomal V0 subunit A3
0,93	SYCN	syncollin
0,93	RARRES2	retinoic acid receptor responder (tazarotene induced) 2
0,93	CCDC13	coiled-coil domain containing 13
0,93	FAM120A	family with sequence similarity 120A
0,93	XIAP	X-linked inhibitor of apoptosis, E3 ubiquitin protein ligase
0,93	DTWD2	DTW domain containing 2
0,93	OR1E3	olfactory receptor, family 1, subfamily E, member 3 (gene/pseudogene)
0,93	DOLK	dolichol kinase
0,93	TRIM39-RPP21	TRIM39-RPP21 readthrough
0,93	HOXB-AS3	HOXB cluster antisense RNA 3
0,93	KEAP1	kelch-like ECH-associated protein 1
0,93	PRSS36	protease, serine 36
0,93	QRICH2	Jeck2013 ALT_DONOR, coding, INTERNAL, intronic best transcript NM_032134
0,93	KIF27	kinesin family member 27
0,93	LRRC14	leucine rich repeat containing 14
0,93	TAOK3	TAO kinase 3
0,93	CPXCR1	CPX chromosome region, candidate 1
0,93	GLT8D2	glycosyltransferase 8 domain containing 2
0,93	CYB561D2	cytochrome b561 family, member D2
0,93	MEGF10	multiple EGF-like-domains 10
0,93	NAA35	N(alpha)-acetyltransferase 35, NatC auxiliary subunit
0,93	EPB41L4A	erythrocyte membrane protein band 4.1 like 4A
0,93	MLLT6	myeloid/lymphoid or mixed-lineage leukemia; translocated to, 6
0,93	SLC17A7	solute carrier family 17 (vesicular glutamate transporter), member 7
0,93	TRIM28	tripartite motif containing 28
0,93	LSM5	LSM5 homolog, U6 small nuclear RNA and mRNA degradation associated
0,93	DPP7	dipeptidyl-peptidase 7
0,93	P2RY1	purinergic receptor P2Y, G-protein coupled, 1
0,93	ACVR2B	activin A receptor type IIB
0,93	EXOC3	exocyst complex component 3
0,93	CNNM2	cyclin and CBS domain divalent metal cation transport mediator 2
0,93	AK4	adenylate kinase 4
0,93	KCNK6	potassium channel, two pore domain subfamily K, member 6

0,93	ZNF341	zinc finger protein 341
0,93	MATK	megakaryocyte-associated tyrosine kinase
0,93	ATP5C1	ATP synthase, H+ transporting, mitochondrial F1 complex, gamma polypeptide 1
0,93	FBXO4	F-box protein 4
0,93	ZMYND19	zinc finger, MYND-type containing 19
0,93	IFIT5	interferon-induced protein with tetratricopeptide repeats 5
0,93	LYPLAL1	lysophospholipase-like 1
0,93	MYNN	myoneurin
0,93	ESCO1	establishment of sister chromatid cohesion N-acetyltransferase 1
0,93	RRAGA	Ras-related GTP binding A
0,93	DCTN4	dynactin 4 (p62)
0,93	GNRHR	gonadotropin releasing hormone receptor
0,93	FRMPD3-AS1	FRMPD3 antisense RNA 1
0,93	LSS	lanosterol synthase (2,3-oxidosqualene-lanosterol cyclase)
0,93	NUSAP1	nucleolar and spindle associated protein 1
0,93	NTMT1	N-terminal Xaa-Pro-Lys N-methyltransferase 1
0,93	ZNF222	zinc finger protein 222
0,93	MARC2	mitochondrial amidoxime reducing component 2
0,93	TEX9	testis expressed 9
0,93	SPATS2	spermatogenesis associated, serine-rich 2
0,93	KBTBD6	kelch repeat and BTB (POZ) domain containing 6
0,93	ZNF107; MIR6839	zinc finger protein 107; microRNA 6839
0,93	RFC5	replication factor C subunit 5
0,93	CIDEB	cell death-inducing DFFA-like effector b
0,93	MMP17	matrix metallopeptidase 17 (membrane-inserted)
0,93	USH1G	Usher syndrome 1G (autosomal recessive)
0,93	CSTA	cystatin A (stefin A)
0,93	GUSB	glucuronidase, beta
0,93	VSIG2	V-set and immunoglobulin domain containing 2
0,93	OR1K1	olfactory receptor, family 1, subfamily K, member 1
0,93	EIF3G	eukaryotic translation initiation factor 3, subunit G
0,93	DDX23	DEAD (Asp-Glu-Ala-Asp) box polypeptide 23
0,93	WBP1	WW domain binding protein 1
0,93	MOB3B	MOB kinase activator 3B
0,93	RDM1	RAD52 motif containing 1
0,93	C6orf136	chromosome 6 open reading frame 136
0,93	TMEM204	transmembrane protein 204
0,93	HIST1H2BE	Memczak2013 ANTISENSE, CDS, coding, upstream_start, UTR3, UTR5 best transcript NM_003523
0,93	POLD1	polymerase (DNA directed), delta 1, catalytic subunit
0,93	NEDD4	neural precursor cell expressed, developmentally down-regulated 4, E3 ubiquitin protein ligase
0,93	OR10Q1	olfactory receptor, family 10, subfamily Q, member 1
0,93	RBX1	ring-box 1, E3 ubiquitin protein ligase

0,93	PAG1	phosphoprotein membrane anchor with glycosphingolipid microdomains 1
0,93	DRC3	dynein regulatory complex subunit 3
0,93	LCN12	lipocalin 12
0,93	PHC2; MIR3605	polyhomeotic homolog 2 (Drosophila); microRNA 3605
0,93	PTDSS1	Memczak2013 ANTISENSE, coding, INTERNAL, intronic best transcript NM_014754
0,93	ASIC3	acid sensing ion channel 3
0,93	CTDSPL2	CTD small phosphatase like 2
0,93	CHI3L1	chitinase 3-like 1 (cartilage glycoprotein-39)
0,93	RAD54L2	RAD54-like 2 (S. cerevisiae)
0,93	INTS5	integrator complex subunit 5
0,93	NDUFB6	NADH dehydrogenase (ubiquinone) 1 beta subcomplex, 6, 17kDa
0,93	KRTAP2-2	keratin associated protein 2-2
0,93	GAL3ST4	galactose-3-O-sulfotransferase 4
0,93	ARMCX3	armadillo repeat containing, X-linked 3
0,93	PDE4B	phosphodiesterase 4B, cAMP-specific
0,93	C15orf54	chromosome 15 open reading frame 54
0,93	PRLHR	prolactin releasing hormone receptor
0,93	AGRP	agouti related neuropeptide
0,93	ANKS1A	ankyrin repeat and sterile alpha motif domain containing 1A
0,93	ACIN1	Transcript Identified by AceView, Entrez Gene ID(s) 22985
0,93	SERPINB3	serpin peptidase inhibitor, clade B (ovalbumin), member 3
0,93	PRB2	proline-rich protein BstNI subfamily 2
0,93	MGAM2	maltase-glucoamylase 2 (putative)
0,93	ZNRD1	zinc ribbon domain containing 1
0,93	MAPK1	mitogen-activated protein kinase 1
0,93	KLHL41	kelch-like family member 41
0,93	FAM57B	family with sequence similarity 57, member B
0,93	DCHS2	dachsous cadherin-related 2
0,93	ZNF607	zinc finger protein 607
0,93	H3F3AP4; H3F3A; H3F3B	Homo sapiens H3 histone, family 3A, pseudogene 4 (H3F3AP4), non-coding RNA.; Homo sapiens H3 histone, family 3A, mRNA (cDNA clone MGC:47761 IMAGE:6014954), complete cds.; Homo sapiens H3 histone, family 3B (H3.3B), mRNA (cDNA clone MGC:87782 IMAGE:3884607), complete cds.; Homo sapiens H3 histone, family 3A, mRNA (cDNA clone MGC:87783 IMAGE:5162817), complete cds.
0,93	RPL31	ribosomal protein L31
0,93	RPS6KA1	ribosomal protein S6 kinase, 90kDa, polypeptide 1
0,93	DUSP16	dual specificity phosphatase 16
0,93	RNF40	ring finger protein 40, E3 ubiquitin protein ligase
0,93	DONSON; CRYZL1	downstream neighbor of SON; crystallin zeta like 1

0,93	SUPT6H	SPT6 homolog, histone chaperone
0,93	CCM2	cerebral cavernous malformation 2
0,93	OR10AC1	olfactory receptor, family 10, subfamily AC, member 1 (gene/pseudogene) [Source:HGNC Symbol;Acc:HGNC:14758]
0,93	UBL4A	ubiquitin-like 4A
0,93	MFSD8	major facilitator superfamily domain containing 8
0,93	C4orf27	chromosome 4 open reading frame 27
0,93	XAGE1E; XAGE1B	X antigen family, member 1E; X antigen family, member 1B
0,93	MS4A6A	membrane-spanning 4-domains, subfamily A, member 6A
0,93	VRK1	vaccinia related kinase 1
0,93	APBB3; MIR6831	amyloid beta (A4) precursor protein-binding, family B, member 3; microRNA 6831
0,93	SMPD3	sphingomyelin phosphodiesterase 3, neutral membrane (neutral sphingomyelinase II)
0,93	CDK18	cyclin-dependent kinase 18
0,93	FUNDC1	FUN14 domain containing 1
0,93	FIZ1	FLT3-interacting zinc finger 1
0,93	SERHL	serine hydrolase-like (pseudogene)
0,93	ARL15	ADP-ribosylation factor like GTPase 15
0,93	LIMK2	LIM domain kinase 2
0,93	GNA14	guanine nucleotide binding protein (G protein), alpha 14
0,93	STX5	syntaxin 5
0,93	GAA	glucosidase, alpha; acid
0,93	TRIM49	tripartite motif containing 49
0,93	OR9G4	olfactory receptor, family 9, subfamily G, member 4
0,93	CXCL5	chemokine (C-X-C motif) ligand 5
0,93	KDM5C	lysine (K)-specific demethylase 5C
0,93	EMB	embigin
0,93	TFEB	transcription factor EB
0,93	PRTFDC1	phosphoribosyl transferase domain containing 1
0,93	TST	thiosulfate sulfurtransferase (rhodanese)
0,93	ADIG	adipogenin
0,93	ARHGAP17	Rho GTPase activating protein 17
0,93	NDUFB5	NADH dehydrogenase (ubiquinone) 1 beta subcomplex, 5, 16kDa
0,93	PGBD2	piggyBac transposable element derived 2
0,93	CD5	CD5 molecule
0,93	ZNF622	zinc finger protein 622
0,93	RBBP9	retinoblastoma binding protein 9
0,93	PCBP1	poly(rC) binding protein 1
0,93	IFNL2	interferon, lambda 2
0,93	FBXL14	F-box and leucine-rich repeat protein 14
0,93	TANK	TRAF family member-associated NFKB activator
0,93	OTULIN	OTU deubiquitinase with linear linkage specificity
0,93	ATG10	autophagy related 10
0,93	C8orf34	chromosome 8 open reading frame 34

0,93	FAM83E	family with sequence similarity 83, member E
0,93	SYMPK	symplekin
0,93	AP1S1	adaptor-related protein complex 1 sigma 1 subunit
0,93	TRAPPC4	trafficking protein particle complex 4
0,93	CHD8	chromodomain helicase DNA binding protein 8
0,93	LENEP	lens epithelial protein
0,93	NCCRP1	non-specific cytotoxic cell receptor protein 1 homolog (zebrafish)
0,93	NACC2	NACC family member 2, BEN and BTB (POZ) domain containing
0,93	IFNW1	interferon, omega 1
0,93	MYCT1	myc target 1
0,93	RNF25	ring finger protein 25
0,93	IMPG2	interphotoreceptor matrix proteoglycan 2
0,93	EIF3F	Eukaryotic translation initiation factor 3 subunit F [Source:UniProtKB/Swiss-Prot;Acc:O00303]
0,93	MLLT4	myeloid/lymphoid or mixed-lineage leukemia; translocated to, 4
0,93	KRT40	keratin 40, type I
0,93	CT47A7; CT47A5; CT47A6	cancer/testis antigen family 47, member A7; cancer/testis antigen family 47, member A5; cancer/testis antigen family 47, member A6
0,93	ENDOV	endonuclease V
0,93	IP6K3	inositol hexakisphosphate kinase 3
0,93	HPS1; MIR4685	Hermansky-Pudlak syndrome 1; microRNA 4685
0,93	KRTAP4-1	keratin associated protein 4-1
0,93	LIN28A	lin-28 homolog A (C. elegans)
0,93	RIF1	replication timing regulatory factor 1
0,93	CLASRP	CLK4-associating serine/arginine rich protein
0,93	AR	androgen receptor
0,93	TNFAIP1	tumor necrosis factor, alpha-induced protein 1 (endothelial)
0,93	GANAB	glucosidase, alpha; neutral AB
0,93	NPPB	natriuretic peptide B
0,93	TMEM45A	transmembrane protein 45A
0,93	ENPP5	ectonucleotide pyrophosphatase/phosphodiesterase 5 (putative)
0,93	ARHGAP22	Rho GTPase activating protein 22
0,93	FGF4	fibroblast growth factor 4
0,93	FAM132B	family with sequence similarity 132, member B
0,93	ZGLP1	zinc finger, GATA-like protein 1
0,93	CCNG2	cyclin G2
0,93	ADAMTS3	ADAM metallopeptidase with thrombospondin type 1 motif 3
0,93	LYST	lysosomal trafficking regulator
0,93	SV2B	synaptic vesicle glycoprotein 2B
0,93	COPZ2	coatomer protein complex subunit zeta 2
0,93	TDRD15	tudor domain containing 15
0,93	HMG20A	high mobility group 20A
0,93	TACC1	transforming, acidic coiled-coil containing protein 1

0,93	PDZD8	PDZ domain containing 8
0,93	AP4E1	adaptor-related protein complex 4, epsilon 1 subunit
0,93	KIF6	kinesin family member 6
0,93	MANSC4	MANSC domain containing 4
0,93	GLRX	glutaredoxin
0,93	UBC	ubiquitin C
0,93	PHF20	PHD finger protein 20
0,93	NSMCE2	NSE2/MMS21 homolog, SMC5-SMC6 complex SUMO ligase
0,93	GRIK1-AS2; BACH1	GRIK1 antisense RNA 2; BTB and CNC homology 1, basic leucine zipper transcription factor 1
0,93	KNDC1	kinase non-catalytic C-lobe domain (KIND) containing 1
0,93	TRIM26	tripartite motif containing 26
0,93	PCDH12	protocadherin 12
0,93	GNAT1	guanine nucleotide binding protein (G protein), alpha transducing activity polypeptide 1
0,93	PDK1	pyruvate dehydrogenase kinase, isozyme 1
0,93	CFDP1	craniofacial development protein 1
0,93	METTL12; SNORA57	methyltransferase like 12; small nucleolar RNA, H/ACA box 57
0,93	TUSC3	tumor suppressor candidate 3
0,93	CBX4	chromobox homolog 4
0,93	SEC22A	SEC22 homolog A, vesicle trafficking protein
0,93	CMA1	chymase 1, mast cell
0,93	IKZF4	IKAROS family zinc finger 4
0,93	SCAP	SREBF chaperone
0,93	LOC728485; CTD-2162K18.4	uncharacterized LOC728485; novel transcript
0,93	SLC22A25	solute carrier family 22, member 25
0,93	RNF26	ring finger protein 26
0,93	C17orf80	chromosome 17 open reading frame 80
0,93	RBMS1	Jeck2013 ALT_ACCEPTOR, ALT_DONOR, coding, INTERNAL, intronic best transcript NM_016836
0,93	LMTK2	lemur tyrosine kinase 2
0,93	CHPT1	choline phosphotransferase 1
0,93	C12orf54	chromosome 12 open reading frame 54
0,93	STXBP1	syntaxin binding protein 1
0,93	DEFB105A	defensin, beta 105A
0,93	ARHGEF40	Rho guanine nucleotide exchange factor (GEF) 40
0,93	NDUFA4	NDUFA4, mitochondrial complex associated
0,93	CLRN3	clarin 3
0,93	PPP1R1A	protein phosphatase 1, regulatory (inhibitor) subunit 1A
0,93	CLTB	clathrin, light chain B
0,93	TMEM80	transmembrane protein 80
0,93	CKAP4	cytoskeleton-associated protein 4
0,93	ADCY1	adenylate cyclase 1 (brain)
0,93	SPCS2	signal peptidase complex subunit 2
0,93	OXCT2	3-oxoacid CoA-transferase 2

0,93	SURF4	surfeit 4
0,93	PPP1R27	protein phosphatase 1, regulatory subunit 27
0,93	USP17L19	ubiquitin specific peptidase 17-like family member 19
0,93	ZNF516	zinc finger protein 516
0,93	MLXIP	MLX interacting protein
0,93	KIF7	Memczak2013 ANTISENSE, CDS, coding, INTERNAL best transcript NM_198525
0,93	PSMB8	proteasome subunit beta 8
0,93	EVX2	even-skipped homeobox 2
0,93	CA6	carbonic anhydrase VI
0,93	ATG9B	autophagy related 9B
0,93	C9orf62	chromosome 9 open reading frame 62
0,93	ANAPC11	anaphase promoting complex subunit 11
0,93	NELL2	neural EGFL like 2
0,93	DEFB104A; DEFB104B	defensin, beta 104A; defensin, beta 104B
0,93	UBAP1	ubiquitin associated protein 1
0,93	ZNF773	zinc finger protein 773
0,93	SON; MIR6501	SON DNA binding protein; microRNA 6501
0,93	DGKD	diacylglycerol kinase, delta 130kDa
0,93	OR10H4	olfactory receptor, family 10, subfamily H, member 4
0,93	INF2	inverted formin, FH2 and WH2 domain containing
0,93	RFX7	regulatory factor X, 7
0,93	DGKE	diacylglycerol kinase, epsilon 64kDa
0,93	TBCEL	tubulin folding cofactor E-like
0,93	HMGB2	high mobility group box 2
0,93	CENPB	centromere protein B
0,93	MAN1A2	mannosidase, alpha, class 1A, member 2
0,93	LLGL2	lethal giant larvae homolog 2 (Drosophila)
0,93	XKR3	X-linked Kx blood group related 3
0,93	LRRIQ3	leucine-rich repeats and IQ motif containing 3
0,93	TSC22D1	TSC22 domain family, member 1
0,93	GATAD2B	GATA zinc finger domain containing 2B
0,93	APITD1-CORT; CORT; APITD1	APITD1-CORT readthrough; cortistatin; apoptosis-inducing, TAF9-like domain 1
0,93	GYPC	Memczak2013 ANTISENSE, coding, INTERNAL, UTR3 best transcript NM_016815
0,93	LRGUK	leucine-rich repeats and guanylate kinase domain containing
0,93	NKTR	natural killer cell triggering receptor
0,93	IGSF6	immunoglobulin superfamily, member 6
0,93	GRINA	glutamate receptor, ionotropic, N-methyl D-aspartate-associated protein 1 (glutamate binding)
0,93	ZBED4	zinc finger, BED-type containing 4
0,93	MAGEB18	MAGE family member B18
0,93	HHAT	hedgehog acyltransferase
0,93	C1orf167	chromosome 1 open reading frame 167
0,93	GREB1L	growth regulation by estrogen in breast cancer-like

0,93	NINL	Zhang2013 ALT_ACCEPTOR, ALT_DONOR, coding, INTERNAL, intronic best transcript NM_025176
0,93	GTPBP2	GTP binding protein 2
0,93	PTCHD2	patched domain containing 2
0,93	KIAA1549	KIAA1549
0,93	ST8SIA1	ST8 alpha-N-acetyl-neuraminide alpha-2,8-sialyltransferase 1
0,93	TOR1AIP2	torsin A interacting protein 2
0,93	SSC5D	scavenger receptor cysteine rich family, 5 domains
0,93	JCHAIN	joining chain of multimeric IgA and IgM
0,93	MROH6	maestro heat-like repeat family member 6
0,93	DCLK1	doublecortin-like kinase 1
0,93	HOXB6	homeobox B6
0,93	ANKFY1	ankyrin repeat and FYVE domain containing 1
0,93	CELF5	CUGBP, Elav-like family member 5
0,93	BEGAIN	brain-enriched guanylate kinase-associated
0,93	ZRANB3	zinc finger, RAN-binding domain containing 3
0,93	SOWAHA	sosondowah ankyrin repeat domain family member A
0,93	BRAF	B-Raf proto-oncogene, serine/threonine kinase
0,93	ARFRP1	ADP-ribosylation factor related protein 1
0,93	UPRT	uracil phosphoribosyltransferase (FUR1) homolog (S. cerevisiae)
0,93	ZNF705A; FAM66C	zinc finger protein 705A; family with sequence similarity 66, member C
0,93	TAS1R1	taste receptor, type 1, member 1
0,93	ADAMTS16	ADAM metallopeptidase with thrombospondin type 1 motif 16
0,93	SDC1	syndecan 1
0,93	ERBB4	erb-b2 receptor tyrosine kinase 4
0,93	COX7A2	cytochrome c oxidase subunit VIIa polypeptide 2 (liver)
0,93	ATP13A2	ATPase type 13A2
0,93	TISP43; LOC646743; AC013269.5	uncharacterized LOC150527; uncharacterized LOC646743; Transcript Identified by AceView, Entrez Gene ID(s) 646743, RefSeq ID(s) NR_033930; Uncharacterized protein [Source:UniProtKB/TrEMBL;Acc:B9A039]
0,93	USP17L23	ubiquitin specific peptidase 17-like family member 23 [Source:HGNC Symbol;Acc:HGNC:44451]
0,93	FNIP1	folliculin interacting protein 1
0,93	OR6C76	olfactory receptor, family 6, subfamily C, member 76
0,93	GPR63	G protein-coupled receptor 63
0,93	UROD	uroporphyrinogen decarboxylase
0,93	KCNE4	potassium channel, voltage gated subfamily E regulatory beta subunit 4
0,93	HIST1H2BJ	histone cluster 1, H2bj
0,93	PKP1	plakophilin 1
0,93	FFAR2	free fatty acid receptor 2
0,93	GIPC1	GIPC PDZ domain containing family, member 1

0,93	ZNF689	zinc finger protein 689
0,93	CENPA	centromere protein A
0,93	SNX12	sorting nexin 12
0,93	TERF2IP	telomeric repeat binding factor 2, interacting protein
0,93	NOS1AP	nitric oxide synthase 1 (neuronal) adaptor protein
0,93	MAP1LC3A	microtubule-associated protein 1 light chain 3 alpha
0,93	SMPDL3A	sphingomyelin phosphodiesterase, acid-like 3A
0,93	ANKEF1	ankyrin repeat and EF-hand domain containing 1
0,93	ZNF8	zinc finger protein 8
0,93	DLK2	delta-like 2 homolog (Drosophila)
0,93	NDUFA10	NADH dehydrogenase (ubiquinone) 1 alpha subcomplex, 10, 42kDa
0,93	SCML1	sex comb on midleg-like 1 (Drosophila)
0,93	RHPN2	rhophilin, Rho GTPase binding protein 2
0,93	CCDC53	coiled-coil domain containing 53
0,93	C16orf46	chromosome 16 open reading frame 46
0,93	NPIPB4	nuclear pore complex interacting protein family, member B4
0,93	THEM4	thioesterase superfamily member 4
0,93	GPALPP1	GPALPP motifs containing 1
0,93	PIP5K1C	phosphatidylinositol-4-phosphate 5-kinase, type I, gamma
0,93	NHEJ1	nonhomologous end-joining factor 1
0,93	PPP1R3C	protein phosphatase 1, regulatory subunit 3C
0,93	TEAD1	TEA domain family member 1 (SV40 transcriptional enhancer factor)
0,93	C3orf62	chromosome 3 open reading frame 62
0,93	ACOT2	acyl-CoA thioesterase 2
0,93	KRT12	keratin 12, type I
0,93	F2RL3	coagulation factor II (thrombin) receptor-like 3
0,93	TUBB1	tubulin, beta 1 class VI
0,93	EFCAB6	EF-hand calcium binding domain 6
0,93	KIF2A	kinesin heavy chain member 2A
0,93	TIAL1	TIA1 cytotoxic granule-associated RNA binding protein-like 1
0,93	SURF1	surfeit 1
0,93	TMCC2	transmembrane and coiled-coil domain family 2
0,93	AVIL	advillin
0,93	SND1; SND1-IT1	staphylococcal nuclease and tudor domain containing 1; SND1 intronic transcript 1
0,93	ZSCAN12	zinc finger and SCAN domain containing 12
0,93	RPS5	ribosomal protein S5
0,93	LGALS13	lectin, galactoside-binding, soluble, 13
0,93	TATDN2; GHRLOS	TatD DNase domain containing 2; ghrelin opposite strand/antisense RNA
0,93	KPNB1	karyopherin (importin) beta 1
0,93	RPS3; SNORD15A	ribosomal protein S3; small nucleolar RNA, C/D box 15A
0,93	ST13	suppression of tumorigenicity 13 (colon carcinoma) (Hsp70 interacting protein)

0,93	SGK223	homolog of rat pragma of Rnd2; Tyrosine-protein kinase SgK223 [Source:UniProtKB/Swiss-Prot;Acc:Q86YV5]
0,93	E2F6	E2F transcription factor 6
0,93	C19orf12	chromosome 19 open reading frame 12
0,93	MPPE1	metallophosphoesterase 1
0,93	NPPC	natriuretic peptide C
0,93	TAAR3	trace amine associated receptor 3 (gene/pseudogene)
0,93	ODF3L2	outer dense fiber of sperm tails 3-like 2
0,93	TTC39C	tetratricopeptide repeat domain 39C
0,93	VPS13C	vacuolar protein sorting 13 homolog C (S. cerevisiae)
0,93	FAM126B	family with sequence similarity 126, member B
0,93	LCN2	lipocalin 2
0,93	PDPK1	3-phosphoinositide dependent protein kinase 1
0,93	POLE	polymerase (DNA directed), epsilon, catalytic subunit
0,93	TBC1D13	TBC1 domain family, member 13
0,93	PRKCE	protein kinase C, epsilon
0,93	PADI6	peptidyl arginine deiminase, type VI
0,93	RPAP2	RNA polymerase II associated protein 2
0,93	SNN	stannin
0,93	GZMM	granzyme M
0,93	PLSCR1	phospholipid scramblase 1
0,93	ARHGAP11B; ARHGAP11A	Rho GTPase activating protein 11B; Rho GTPase activating protein 11A
0,93	HCN4	hyperpolarization activated cyclic nucleotide gated potassium channel 4
0,93	CTSF	cathepsin F
0,93	YLPM1	YLP motif containing 1
0,93	HSPA12A	heat shock 70kDa protein 12A
0,93	POLA1	polymerase (DNA directed), alpha 1, catalytic subunit
0,93	TENM2	teneurin transmembrane protein 2
0,93	SLC39A12	solute carrier family 39 (zinc transporter), member 12
0,93	WDFY1	WD repeat and FYVE domain containing 1
0,93	MFAP1	microfibrillar associated protein 1
0,93	IRAK4	interleukin 1 receptor associated kinase 4
0,93	ZNF501	zinc finger protein 501 [Source:HGNC Symbol;Acc:HGNC:23717]
0,93	C1orf194	chromosome 1 open reading frame 194
0,93	FBXO34	F-box protein 34
0,93	SLC16A1	solute carrier family 16 (monocarboxylate transporter), member 1
0,93	UFSP1	UFM1-specific peptidase 1 (inactive)
0,93	C6orf203	chromosome 6 open reading frame 203
0,93	H2BFM	H2B histone family, member M
0,93	LOC100134391; CTD-2532D12.4	uncharacterized LOC100134391; novel transcript
0,93	ADRBK1	adrenergic, beta, receptor kinase 1
0,93	PF4V1	platelet factor 4 variant 1

0,93	TCERG1	Transcript Identified by AceView, Entrez Gene ID(s) 10915
0,93	DNLZ	DNL-type zinc finger
0,93	MTMR3	myotubularin related protein 3
0,93	METTL23	methyltransferase like 23
0,93	MAPKAPK2	mitogen-activated protein kinase-activated protein kinase 2
0,93	POLH	polymerase (DNA directed), eta
0,93	TBATA	thymus, brain and testes associated
0,93	INO80D	INO80 complex subunit D
0,93	LCE3A	late cornified envelope 3A
0,93	DENND4A	DENN/MADD domain containing 4A
0,93	ALOX5AP	arachidonate 5-lipoxygenase-activating protein
0,93	C3orf70	chromosome 3 open reading frame 70
0,93	GLO1	glyoxalase I
0,93	INPP5D	inositol polyphosphate-5-phosphatase D
0,93	NAT14	N-acetyltransferase 14 (GCN5-related, putative)
0,93	SLC25A18	solute carrier family 25 (glutamate carrier), member 18
0,93	MPC1	mitochondrial pyruvate carrier 1
0,93	TTL	tubulin tyrosine ligase
0,93	MSL2	male-specific lethal 2 homolog (Drosophila)
0,93	C6orf106	chromosome 6 open reading frame 106
0,93	FMOD	fibromodulin
0,93	PLCG1; RPL23AP81	phospholipase C, gamma 1; ribosomal protein L23a pseudogene 81
0,93	POLQ	polymerase (DNA directed), theta
0,93	GPR68	G protein-coupled receptor 68
0,93	TYRO3	TYRO3 protein tyrosine kinase
0,93	FBXO6	F-box protein 6
0,93	SETD5	SET domain containing 5
0,93	MAD1L1	MAD1 mitotic arrest deficient-like 1 (yeast)
0,93	CHD9	Transcript Identified by AceView, Entrez Gene ID(s) 80205
0,93	YTHDC1	YTH domain containing 1
0,93	KLRAP1	killer cell lectin-like receptor subfamily A pseudogene 1
0,93	HSD17B10	hydroxysteroid (17-beta) dehydrogenase 10
0,93	FLOT2	flotillin 2
0,93	ITGB1BP2	integrin beta 1 binding protein (melusin) 2
0,93	LLPH	LLP homolog, long-term synaptic facilitation (Aplysia)
0,93	TBL1Y	transducin (beta)-like 1, Y-linked
0,93	SMIM4	small integral membrane protein 4
0,93	CDC37L1	cell division cycle 37-like 1
0,93	FKBP10	FK506 binding protein 10
0,93	AP3B1	adaptor-related protein complex 3, beta 1 subunit
0,93	PHLPP1	PH domain and leucine rich repeat protein phosphatase 1

0,93	SLC22A3	solute carrier family 22 (organic cation transporter), member 3
0,93	TYMS	thymidylate synthetase
0,93	CSNK1E	casein kinase 1, epsilon
0,93	SORL1	sortilin-related receptor, L(DLR class) A repeats containing
0,93	DDIT4	DNA damage inducible transcript 4
0,93	NDUFA6	NADH dehydrogenase (ubiquinone) 1 alpha subcomplex, 6, 14kDa
0,93	LOC729159; RP11-457D20.2	UPF0607 protein ENSP00000381418-like; Putative UPF0607 protein ENSP00000381418 [Source:UniProtKB/Swiss-Prot;Acc:A8MU76]
0,93	PRKAR2B	protein kinase, cAMP-dependent, regulatory, type II, beta
0,93	PIGC	phosphatidylinositol glycan anchor biosynthesis class C
0,93	LRRC17	leucine rich repeat containing 17
0,93	RHOJ	ras homolog family member J
0,93	TMEM237	transmembrane protein 237
0,93	FAM160A1	family with sequence similarity 160, member A1
0,93	NDUFA12	NADH dehydrogenase (ubiquinone) 1 alpha subcomplex, 12
0,93	NR5A2	nuclear receptor subfamily 5, group A, member 2
0,93	THOC6	THO complex 6
0,93	FAM153B	family with sequence similarity 153, member B
0,93	FEM1B	fem-1 homolog b (C. elegans)
0,93	BRI3	brain protein I3
0,93	MRI1	methylthioribose-1-phosphate isomerase 1
0,93	LOC643355; RP11-88H9.2; toyfawbu	uncharacterized LOC643355; putative novel transcript; Transcript Identified by AceView
0,93	LCE1A	late cornified envelope 1A
0,93	SLFN5	schlafen family member 5
0,93	C1orf112	chromosome 1 open reading frame 112
0,93	PSAP	prosaposin
0,93	H2AFB3	H2A histone family, member B3
0,93	AAMDC	adipogenesis associated, Mth938 domain containing
0,93	SLC24A3	solute carrier family 24 (sodium/potassium/calcium exchanger), member 3
0,93	OR1L1	olfactory receptor, family 1, subfamily L, member 1
0,93	GIPC3	GIPC PDZ domain containing family, member 3
0,93	MORC4	MORC family CW-type zinc finger 4
0,93	PACRG	PARK2 co-regulated
0,93	GPR42	G protein-coupled receptor 42 (gene/pseudogene)
0,93	SSC4D	scavenger receptor cysteine rich family, 4 domains
0,93	SMCR8	Smith-Magenis syndrome chromosome region, candidate 8
0,93	ZNF200	Transcript Identified by AceView, Entrez Gene ID(s) 7752
0,93	FEZF1	FEZ family zinc finger 1

0,93	SLC12A2	solute carrier family 12 (sodium/potassium/chloride transporter), member 2
0,93	EPB42	erythrocyte membrane protein band 4.2
0,93	EDEM3	ER degradation enhancer, mannosidase alpha-like 3
0,93	ITLN2	intelectin 2
0,93	FUT3	fucosyltransferase 3 (galactoside 3(4)-L-fucosyltransferase, Lewis blood group)
0,93	HUS1B	HUS1 checkpoint clamp component B
0,93	BCL2L10	BCL2-like 10 (apoptosis facilitator)
0,93	SH2B1	SH2B adaptor protein 1
0,93	VIPR2	vasoactive intestinal peptide receptor 2
0,93	CCDC183; RABL6	coiled-coil domain containing 183; RAB, member RAS oncogene family-like 6
0,93	SMYD3	SET and MYND domain containing 3
0,93	MCHR1	melanin-concentrating hormone receptor 1
0,93	KATNAL1	katanin p60 subunit A-like 1
0,93	MON1B	MON1 secretory trafficking family member B
0,93	EML3	echinoderm microtubule associated protein like 3
0,93	ST8SIA4	ST8 alpha-N-acetyl-neuraminide alpha-2,8-sialyltransferase 4
0,93	SLC32A1	solute carrier family 32 (GABA vesicular transporter), member 1
0,93	MYSM1	Myb-like, SWIRM and MPN domains 1
0,93	PAK1	p21 protein (Cdc42/Rac)-activated kinase 1
0,93	ESR2	estrogen receptor 2 (ER beta)
0,93	TRAK2	Jeck2013 ALT_ACCEPTOR, ALT_DONOR, coding, INTERNAL, intronic best transcript NM_015049
0,93	MTR	5-methyltetrahydrofolate-homocysteine methyltransferase
0,93	UBE2QL1	ubiquitin conjugating enzyme E2Q family-like 1
0,93	DYRK1B	dual specificity tyrosine-(Y)-phosphorylation regulated kinase 1B
0,93	GSG2	germ cell associated 2 (haspin)
0,93	FAM73B	family with sequence similarity 73, member B
0,93	OSBPL3	oxysterol binding protein-like 3
0,93	SELK	selenoprotein K; selenoprotein K [Source:EntrezGene;Acc:58515]; Transcript Identified by AceView, Entrez Gene ID(s) 58515, RefSeq ID(s) NM_021237
0,93	IL31	interleukin 31
0,93	ZKSCAN1	zinc finger with KRAB and SCAN domains 1
0,93	CYP4F11	cytochrome P450, family 4, subfamily F, polypeptide 11
0,93	TBL1XR1	transducin (beta)-like 1 X-linked receptor 1
0,93	RRAGC	Memczak2013 ANTISENSE, coding, INTERNAL, intronic best transcript NM_022157
0,93	CCNB2	cyclin B2
0,93	KIAA0430; MIR6506	KIAA0430; microRNA 6506
0,93	WBP4	WW domain binding protein 4
0,93	NEURL1	neuralized E3 ubiquitin protein ligase 1

0,93	HCAR2	hydroxycarboxylic acid receptor 2
0,93	CCDC22	coiled-coil domain containing 22
0,93	CDC37; MIR1181	cell division cycle 37; microRNA 1181
0,93	IFIT2	interferon-induced protein with tetratricopeptide repeats 2
0,93	CPD	carboxypeptidase D
0,93	RPS4Y2	ribosomal protein S4, Y-linked 2
0,93	OR5H6	olfactory receptor, family 5, subfamily H, member 6 (gene/pseudogene)
0,93	DSCAM	Down syndrome cell adhesion molecule
0,93	WFDC13	WAP four-disulfide core domain 13
0,93	YARS	Transcript Identified by AceView, Entrez Gene ID(s) 8565
0,93	EHMT1	euchromatic histone-lysine N-methyltransferase 1
0,93	PRR15L	proline rich 15-like
0,93	UNC45A	unc-45 myosin chaperone A
0,93	FOXK1	forkhead box K1
0,93	MMP25	matrix metallopeptidase 25
0,93	RNF103	ring finger protein 103
0,93	ATP5G3	ATP synthase, H+ transporting, mitochondrial Fo complex subunit C3 (subunit 9)
0,93	FANCD2	Fanconi anemia complementation group D2
0,93	PRTG	protogenin
0,93	TBC1D22B	TBC1 domain family, member 22B
0,93	SPDYC	speedy/RINGO cell cycle regulator family member C
0,93	EFS	embryonal Fyn-associated substrate
0,93	NYNRIN	NYN domain and retroviral integrase containing
0,93	RBM42	RNA binding motif protein 42
0,93	MAPK15	mitogen-activated protein kinase 15
0,93	DLEC1	deleted in lung and esophageal cancer 1
0,93	ACOT13	acyl-CoA thioesterase 13
0,93	CCDC112	coiled-coil domain containing 112
0,93	KDM6B	lysine (K)-specific demethylase 6B
0,93	PRSS41	protease, serine 41
0,93	ZNF574	zinc finger protein 574
0,93	RNASE7	ribonuclease, RNase A family, 7
0,93	PAAF1	proteasomal ATPase-associated factor 1
0,93	TMA7	translation machinery associated 7 homolog
0,93	SDF4	stromal cell derived factor 4
0,93	EAPP	E2F-associated phosphoprotein
0,93	OR7C1	olfactory receptor, family 7, subfamily C, member 1
0,93	GAPVD1	GTPase activating protein and VPS9 domains 1
0,93	TMEM2	Transcript Identified by AceView, Entrez Gene ID(s) 23670
0,93	SNF8	SNF8, ESCRT-II complex subunit
0,93	MRPL27	mitochondrial ribosomal protein L27
0,93	ARRB1	arrestin, beta 1
0,92	KRT19	keratin 19, type I
0,92	NCAPG2	non-SMC condensin II complex subunit G2

0,92	TTC3	tetratricopeptide repeat domain 3
0,92	NEFL	neurofilament, light polypeptide
0,92	NDUFA10	NADH dehydrogenase (ubiquinone) 1 alpha subcomplex, 10, 42kDa
0,92	SELPLG	selectin P ligand
0,92	ITPR2	inositol 1,4,5-trisphosphate receptor, type 2
0,92	PMEPA1	prostate transmembrane protein, androgen induced 1
0,92	NDUFA13	NADH dehydrogenase (ubiquinone) 1 alpha subcomplex, 13
0,92	ARHGAP5	Rho GTPase activating protein 5
0,92	RC3H2	ring finger and CCCH-type domains 2
0,92	ECSIT	ECSIT signalling integrator
0,92	P4HTM	prolyl 4-hydroxylase, transmembrane (endoplasmic reticulum)
0,92	RALGAPB	Ral GTPase activating protein, beta subunit (non-catalytic)
0,92	RPL10A	ribosomal protein L10a
0,92	ATP6V1E1	ATPase, H+ transporting, lysosomal 31kDa, V1 subunit E1
0,92	RB1	retinoblastoma 1
0,92	IL13RA1	interleukin 13 receptor, alpha 1
0,92	FAM13C	family with sequence similarity 13, member C
0,92	FAM117A	family with sequence similarity 117, member A
0,92	HDDC3	HD domain containing 3
0,92	ZNF266	zinc finger protein 266
0,92	LOC55338; CTC-242N15.1	uncharacterized LOC55338; novel transcript
0,92	UPP2	uridine phosphorylase 2
0,92	SPACA1	sperm acrosome associated 1
0,92	MSI2	Memczak2013 ALT_ACCEPTOR, ALT_DONOR, coding, INTERNAL, intronic best transcript NM_170721
0,92	TDRD7	tudor domain containing 7
0,92	RGSL1	regulator of G-protein signaling like 1
0,92	CTLA4	cytotoxic T-lymphocyte-associated protein 4
0,92	UBE2C	ubiquitin-conjugating enzyme E2C
0,92	LAT	linker for activation of T-cells
0,92	UNC50	unc-50 homolog (C. elegans)
0,92	TNKS1BP1	tankyrase 1 binding protein 1
0,92	CD3D	CD3d molecule, delta (CD3-TCR complex)
0,92	ALG1L2	ALG1, chitobiosyldiphosphodolichol beta-mannosyltransferase-like 2
0,92	TRIM64C	tripartite motif containing 64C
0,92	SNX25	sorting nexin 25
0,92	PEX11A	peroxisomal biogenesis factor 11 alpha
0,92	UBXN6	UBX domain protein 6
0,92	SMIM20	small integral membrane protein 20
0,92	NEU1	sialidase 1 (lysosomal sialidase)
0,92	ADCY4	adenylate cyclase 4
0,92	LINC00598	long intergenic non-protein coding RNA 598

0,92	SLC39A7	solute carrier family 39 (zinc transporter), member 7
0,92	PAK4	p21 protein (Cdc42/Rac)-activated kinase 4
0,92	CCDC166	coiled-coil domain containing 166
0,92	SUGCT	succinyl-CoA:glutarate-CoA transferase
0,92	WIF1	WNT inhibitory factor 1
0,92	STX19	syntaxin 19
0,92	SPDYE1	speedy/RINGO cell cycle regulator family member E1
0,92	ALDH16A1	aldehyde dehydrogenase 16 family, member A1
0,92	ATRAID	all-trans retinoic acid-induced differentiation factor
0,92	GABPA	GA binding protein transcription factor alpha subunit
0,92	SOD2	superoxide dismutase 2, mitochondrial
0,92	RNF144A	ring finger protein 144A
0,92	ZFYVE27	zinc finger, FYVE domain containing 27
0,92	DNAJC21	DnaJ (Hsp40) homolog, subfamily C, member 21
0,92	RAPGEF2	Rap guanine nucleotide exchange factor 2
0,92	WDR59	WD repeat domain 59
0,92	METTL21A	methyltransferase like 21A
0,92	IFT122	intraflagellar transport 122
0,92	AP1M1	adaptor-related protein complex 1, mu 1 subunit
0,92	CKAP5; SNORD67	cytoskeleton associated protein 5; small nucleolar RNA, C/D box 67
0,92	ZNF521	zinc finger protein 521
0,92	AMMECR1L	AMMECR1 like
0,92	BRPF3	bromodomain and PHD finger containing 3
0,92	GJB3	gap junction protein beta 3
0,92	SH3BP4	SH3-domain binding protein 4
0,92	HSPB11	heat shock protein family B (small), member 11
0,92	NUDC	nudC nuclear distribution protein
0,92	ZNF714; VN1R81P	zinc finger protein 714; vomeronasal 1 receptor 81 pseudogene
0,92	PCDHB11	protocadherin beta 11
0,92	PCGF1	polycomb group ring finger 1
0,92	C11orf57	chromosome 11 open reading frame 57
0,92	WIPI2	WD repeat domain, phosphoinositide interacting 2
0,92	ATL3	atlastin GTPase 3
0,92	DENND6A	DENN/MADD domain containing 6A
0,92	FAM219A	family with sequence similarity 219, member A
0,92	ZYG11B	zyg-11 family member B, cell cycle regulator
0,92	TM7SF3	transmembrane 7 superfamily member 3
0,92	DNAJC5; MIR941-1; MIR941-2; MIR941-3; MIR941-4; MIR941-5	DnaJ (Hsp40) homolog, subfamily C, member 5; microRNA 941-1; microRNA 941-2; microRNA 941-3; microRNA 941-4; microRNA 941-5
0,92	ELANE	elastase, neutrophil expressed
0,92	TKT	transketolase
0,92	LINC00552	long intergenic non-protein coding RNA 552
0,92	ERICH1	glutamate rich 1

0,92	CCNJ	cyclin J
0,92	SRGAP2	SLIT-ROBO Rho GTPase activating protein 2
0,92	CCDC187	coiled-coil domain containing 187
0,92	NKX3-2	NK3 homeobox 2
0,92	CMIP	c-Maf inducing protein
0,92	LAPTM4A	Memczak2013 ALT_ACCEPTOR, ALT_DONOR, coding, INTERNAL, intronic best transcript NM_014713
0,92	PDCD11	programmed cell death 11
0,92	NEIL1	nei-like DNA glycosylase 1
0,92	HYOU1	hypoxia up-regulated 1
0,92	RALBP1	ralA binding protein 1
0,92	WRAP73	WD repeat containing, antisense to TP73
0,92	PLEKHO1	pleckstrin homology domain containing, family O member 1
0,92	SPSB1	splA/ryanodine receptor domain and SOCS box containing 1
0,92	LOC101928436; RP11-153F1.1; RP11-347K2.2; teyfawbu	uncharacterized LOC101928436; novel transcript; Transcript Identified by AceView
0,92	ZNF444	zinc finger protein 444
0,92	SLC1A4	solute carrier family 1 (glutamate/neutral amino acid transporter), member 4
0,92	LRP5L	LDL receptor related protein 5 like
0,92	SERGEF	secretion regulating guanine nucleotide exchange factor
0,92	NBEAL1	neurobeachin like 1
0,92	KCNA2	potassium channel, voltage gated shaker related subfamily A, member 2
0,92	TOPBP1	topoisomerase (DNA) II binding protein 1
0,92	LMOD2	leiomodin 2 (cardiac)
0,92	COMMD4	COMM domain containing 4
0,92	AKR1A1	aldo-keto reductase family 1, member A1 (aldehyde reductase)
0,92	QKI	QKI, KH domain containing, RNA binding
0,92	ZSCAN4	zinc finger and SCAN domain containing 4
0,92	RABGGTB	Zhang2013 ALT_ACCEPTOR, ALT_DONOR, coding, INTERNAL, intronic best transcript NM_004582
0,92	OR11H12	olfactory receptor, family 11, subfamily H, member 12
0,92	SAC3D1	SAC3 domain containing 1
0,92	RMDN3	regulator of microtubule dynamics 3
0,92	RNF167	ring finger protein 167
0,92	PWP2	PWP2 periodic tryptophan protein homolog (yeast)
0,92	ADCYAP1R1	adenylate cyclase activating polypeptide 1 (pituitary) receptor type I
0,92	SERPINF1	serpin peptidase inhibitor, clade F (alpha-2 antiplasmin, pigment epithelium derived factor), member 1

0,92	GPR171	G protein-coupled receptor 171
0,92	FNTA	farnesyltransferase, CAAX box, alpha
0,92	RPLP1	ribosomal protein, large, P1
0,92	MRGPRF	MAS-related GPR, member F
0,92	GGA1	golgi-associated, gamma adaptin ear containing, ARF binding protein 1
0,92	AFF1	AF4/FMR2 family, member 1
0,92	TMEM9B	TMEM9 domain family, member B
0,92	ABR	active BCR-related
0,92	C1RL	complement component 1, r subcomponent-like
0,92	SERF2	small EDRK-rich factor 2
0,92	SNAP47	synaptosome associated protein 47kDa
0,92	C9orf152	chromosome 9 open reading frame 152
0,92	LAG3	lymphocyte-activation gene 3
0,92	EFCAB14	EF-hand calcium binding domain 14
0,92	ACVRL1	activin A receptor type IL
0,92	SLC36A4	solute carrier family 36 (proton/amino acid symporter), member 4
0,92	MROH2B	maestro heat-like repeat family member 2B
0,92	SPEG	SPEG complex locus
0,92	LCN9	lipocalin 9
0,92	KPNB1	Memczak2013 ANTISENSE, CDS, coding, INTERNAL best transcript NM_002265
0,92	AIMP2	aminoacyl tRNA synthetase complex-interacting multifunctional protein 2
0,92	CCDC91	coiled-coil domain containing 91
0,92	MTNR1A	melatonin receptor 1A
0,92	GTPBP6	Homo sapiens GTP binding protein 6 (putative) (GTPBP6), mRNA.; GTP binding protein 6 (putative) [Source:HGNC Symbol;Acc:HGNC:30189]; Homo sapiens GTP binding protein 6 (putative), mRNA (cDNA clone MGC:1170 IMAGE:3528598), complete cds.; Salzman2013 ANNOTATED, CDS, coding, INTERNAL, OVCODE, OVERLAPTX, OVEXON best transcript NM_012227
0,92	SHOC2	SHOC2 leucine-rich repeat scaffold protein
0,92	CHRNA1	cholinergic receptor, nicotinic alpha 1
0,92	FANCB	Fanconi anemia complementation group B
0,92	MICAL1	Jeck2013 ANTISENSE, coding, INTERNAL, intronic best transcript NM_022765
0,92	HYOU1	hypoxia up-regulated 1
0,92	R3HDM2	R3H domain containing 2
0,92	PHACTR1	phosphatase and actin regulator 1
0,92	SRXN1	sulfiredoxin 1
0,92	ATMIN	ATM interactor
0,92	HLA-F	major histocompatibility complex, class I, F
0,92	ZNF488	zinc finger protein 488
0,92	TMUB2	transmembrane and ubiquitin-like domain containing 2
0,92	LRP6	LDL receptor related protein 6

0,92	CENPE	centromere protein E
0,92	BAZ1B	bromodomain adjacent to zinc finger domain 1B
0,92	TRAIP	TRAF interacting protein
0,92	CENPW	centromere protein W
0,92	CHMP2A	charged multivesicular body protein 2A
0,92	C12orf65	chromosome 12 open reading frame 65
0,92	KRTAP5-6	keratin associated protein 5-6
0,92	TTC25	tetratricopeptide repeat domain 25
0,92	CPT1A	carnitine palmitoyltransferase 1A (liver)
0,92	ATXN3	ataxin 3
0,92	DHX32	DEAH (Asp-Glu-Ala-His) box polypeptide 32
0,92	CENPV	centromere protein V
0,92	AHCY	adenosylhomocysteinase
0,92	FGD6	FYVE, RhoGEF and PH domain containing 6
0,92	C22orf23	chromosome 22 open reading frame 23
0,92	COL11A2	collagen, type XI, alpha 2
0,92	RPS18	ribosomal protein S18
0,92	FAM96B	family with sequence similarity 96, member B
0,92	CRX	cone-rod homeobox
0,92	KCNC4	potassium channel, voltage gated Shaw related subfamily C, member 4
0,92	PLD3	phospholipase D family, member 3
0,92	TAS2R9	taste receptor, type 2, member 9
0,92	DDR1; MIR4640	discoidin domain receptor tyrosine kinase 1; microRNA 4640
0,92	OR5AU1	olfactory receptor, family 5, subfamily AU, member 1
0,92	TBC1D8	TBC1 domain family, member 8 (with GRAM domain)
0,92	RPL9P7	ribosomal protein L9 pseudogene 7
0,92	KRTAP8-1	keratin associated protein 8-1
0,92	STARD9	StAR-related lipid transfer domain containing 9
0,92	FAM110B	family with sequence similarity 110, member B
0,92	ARSD	arylsulfatase D
0,92	LRRC66	leucine rich repeat containing 66
0,92	GMPR	Zhang2013 ALT_ACCEPTOR, ALT_DONOR, coding, INTERNAL, intronic best transcript NM_006877
0,92	SNRNP200	small nuclear ribonucleoprotein, U5 200kDa subunit
0,92	R3HCC1L	R3H domain and coiled-coil containing 1-like
0,92	GOLGA5	golgin A5
0,92	SRGN	serglycin
0,92	RBFOX2	RNA binding protein, fox-1 homolog (C. elegans) 2
0,92	EIF2B5	eukaryotic translation initiation factor 2B, subunit 5 epsilon, 82kDa
0,92	FZD1	frizzled class receptor 1
0,92	HYOU1	hypoxia up-regulated 1
0,92	UBXN8	UBX domain protein 8
0,92	PRKAR1A	protein kinase, cAMP-dependent, regulatory, type I, alpha
0,92	ADD1	adducin 1 (alpha)

0,92	PAX2	paired box 2
0,92	PLXNC1	plexin C1
0,92	C11orf16	chromosome 11 open reading frame 16
0,92	TUSC2	tumor suppressor candidate 2
0,92	ZP3	zona pellucida glycoprotein 3 (sperm receptor)
0,92	EYA3	EYA transcriptional coactivator and phosphatase 3
0,92	GAS2	growth arrest-specific 2
0,92	SYNJ2	synaptojanin 2
0,92	TMEM116	transmembrane protein 116
0,92	CRNDE	colorectal neoplasia differentially expressed (non-protein coding)
0,92	AKR7L	aldo-keto reductase family 7-like (gene/pseudogene)
0,92	MCAM; MIR6756	melanoma cell adhesion molecule; microRNA 6756
0,92	CCS	copper chaperone for superoxide dismutase
0,92	PADI4	peptidyl arginine deiminase, type IV
0,92	CDKN1C	cyclin-dependent kinase inhibitor 1C (p57, Kip2)
0,92	PUM2	pumilio RNA binding family member 2
0,92	ALLC	allantoicase
0,92	FOXP1	forkhead box P1
0,92	ZSCAN5B	zinc finger and SCAN domain containing 5B
0,92	NBPF19	neuroblastoma breakpoint family, member 19
0,92	P2RY8	purinergic receptor P2Y, G-protein coupled, 8
0,92	P2RY8	Homo sapiens purinergic receptor P2Y, G-protein coupled, 8 (P2RY8), mRNA.; purinergic receptor P2Y, G-protein coupled, 8 [Source:HGNC Symbol;Acc:HGNC:15524]; Homo sapiens purinergic receptor P2Y, G-protein coupled, 8, mRNA (cDNA clone MGC:50878 IMAGE:5787268), complete cds.
0,92	HYOU1	hypoxia up-regulated 1
0,92	COL6A2	collagen, type VI, alpha 2
0,92	CRB2	crumbs family member 2
0,92	AARS	alanyl-tRNA synthetase
0,92	EPB41L2	erythrocyte membrane protein band 4.1-like 2
0,92	MFGE8	milk fat globule-EGF factor 8 protein
0,92	ABTB2	ankyrin repeat and BTB (POZ) domain containing 2
0,92	MOB2	MOB kinase activator 2
0,92	OXSM	3-oxoacyl-ACP synthase, mitochondrial
0,92	ENO3	enolase 3 (beta, muscle)
0,92	CD177	CD177 molecule
0,92	PDHX	pyruvate dehydrogenase complex, component X
0,92	RNF32	ring finger protein 32
0,92	MAD2L1BP	MAD2L1 binding protein
0,92	ITM2C	integral membrane protein 2C
0,92	MAGED2	MAGE family member D2
0,92	HMGCS1	3-hydroxy-3-methylglutaryl-CoA synthase 1 (soluble)
0,92	LSMEM1	leucine-rich single-pass membrane protein 1
0,92	BTBD16	BTB (POZ) domain containing 16
0,92	DVL3	dishevelled segment polarity protein 3

0,92	DAB2	Dab, mitogen-responsive phosphoprotein, homolog 2 (Drosophila)
0,92	EREG	epiregulin
0,92	TOP2B; MIR4442	topoisomerase (DNA) II beta; microRNA 4442
0,92	SCN3B	sodium channel, voltage gated, type III beta subunit
0,92	PLOD3	procollagen-lysine, 2-oxoglutarate 5-dioxygenase 3
0,92	CCDC158	coiled-coil domain containing 158
0,92	CNTNAP2	contactin associated protein-like 2
0,92	CARD19	caspase recruitment domain family, member 19
0,92	SLC25A28	solute carrier family 25 (mitochondrial iron transporter), member 28
0,92	DDX12P; DDX11	DEAD/H (Asp-Glu-Ala-Asp/His) box polypeptide 12, pseudogene; DEAD/H (Asp-Glu-Ala-Asp/His) box helicase 11
0,92	TTC13	tetratricopeptide repeat domain 13
0,92	ANKRD13D	ankyrin repeat domain 13 family, member D
0,92	ASRGL1	asparaginase like 1
0,92	VPS13A	vacuolar protein sorting 13 homolog A (S. cerevisiae)
0,92	IFNA8	interferon, alpha 8
0,92	ZNF576	zinc finger protein 576
0,92	HNMT	histamine N-methyltransferase
0,92	RAD21-AS1	RAD21 antisense RNA 1
0,92	CITED4	Cbp/p300-interacting transactivator, with Glu/Asp rich carboxy-terminal domain, 4
0,92	TET3	tet methylcytosine dioxygenase 3
0,92	OR52M1	olfactory receptor, family 52, subfamily M, member 1
0,92	HYAL3	hyaluronoglucosaminidase 3
0,92	UBTFL1	upstream binding transcription factor, RNA polymerase I-like 1
0,92	SERPINF2	serpin peptidase inhibitor, clade F (alpha-2 antiplasmin, pigment epithelium derived factor), member 2
0,92	F7	coagulation factor VII (serum prothrombin conversion accelerator)
0,92	DSCR4	Down syndrome critical region 4
0,92	CENPC	centromere protein C
0,92	GBP2	guanylate binding protein 2, interferon-inducible
0,92	PICALM	phosphatidylinositol binding clathrin assembly protein
0,92	ENOX1	ecto-NOX disulfide-thiol exchanger 1
0,92	FLI1	Fli-1 proto-oncogene, ETS transcription factor
0,92	ZBED1	zinc finger, BED-type containing 1
0,92	SLC25A1	solute carrier family 25 (mitochondrial carrier; citrate transporter), member 1
0,92	DEFB109P1B	defensin, beta 109, pseudogene 1B
0,92	ZNF260	zinc finger protein 260
0,92	UBR1	ubiquitin protein ligase E3 component n-recognin 1
0,92	OR4C45	olfactory receptor, family 4, subfamily C, member 45
0,92	EFNA1	ephrin-A1

0,92	SEMA3D	sema domain, immunoglobulin domain (Ig), short basic domain, secreted, (semaphorin) 3D
0,92	BECN1	beclin 1, autophagy related
0,92	MPP6	membrane protein, palmitoylated 6
0,92	MYBBP1A	MYB binding protein (P160) 1a
0,92	NDUFB8	NADH dehydrogenase (ubiquinone) 1 beta subcomplex, 8, 19kDa
0,92	PER2	period circadian clock 2
0,92	CETN1	centrin 1
0,92	VSIG10	V-set and immunoglobulin domain containing 10
0,92	PITPNM1	phosphatidylinositol transfer protein, membrane-associated 1
0,92	SUMF1	sulfatase modifying factor 1
0,92	PRR23D2; PRR23D1	proline rich 23 domain containing 2; proline rich 23 domain containing 1
0,92	S100A3	S100 calcium binding protein A3
0,92	KMT5C	lysine (K)-specific methyltransferase 5C
0,92	RHOB	ras homolog family member B
0,92	SEL1L	sel-1 suppressor of lin-12-like (C. elegans)
0,92	RBBP4	retinoblastoma binding protein 4
0,92	HMX3	H6 family homeobox 3
0,92	TMEM121	Memczak2013 ANTISENSE, CDS, coding, INTERNAL best transcript NM_025268
0,92	LMX1B	LIM homeobox transcription factor 1, beta
0,92	MARVELD2	MARVEL domain containing 2
0,92	CEP19	centrosomal protein 19kDa
0,92	TCN2	transcobalamin II
0,92	CYFIP1	cytoplasmic FMR1 interacting protein 1
0,92	SIDT2	SID1 transmembrane family, member 2
0,92	GOLGA7	golgin A7
0,92	ZNF302	zinc finger protein 302
0,92	STKLD1	serine/threonine kinase-like domain containing 1 [Source:HGNC Symbol;Acc:HGNC:28669]
0,92	ERVH48-1	endogenous retrovirus group 48, member 1
0,92	HLA-E	major histocompatibility complex, class I, E
0,92	KIF15	kinesin family member 15
0,92	DNTTIP1	deoxynucleotidyltransferase, terminal, interacting protein 1
0,92	TRMT61B	tRNA methyltransferase 61B
0,92	PTPRM	protein tyrosine phosphatase, receptor type, M
0,92	OIP5	Opa interacting protein 5
0,92	FLNC	filamin C, gamma
0,92	COL5A1	collagen, type V, alpha 1
0,92	TRABD2A	TraB domain containing 2A
0,92	MED28	mediator complex subunit 28
0,92	PRSS48	protease, serine, 48
0,92	OAF	out at first homolog
0,92	ANXA8L1; ANXA8	annexin A8-like 1; annexin A8
0,92	AAED1	AhpC/TSA antioxidant enzyme domain containing 1

0,92	METTL20	methyltransferase like 20
0,92	DLGAP5	discs, large (Drosophila) homolog-associated protein 5
0,92	PDE1A	phosphodiesterase 1A, calmodulin-dependent
0,92	NDST4	N-deacetylase/N-sulfotransferase (heparan glucosaminyl) 4
0,92	WDR38	WD repeat domain 38
0,92	RAB31	RAB31, member RAS oncogene family
0,92	MGA	MGA, MAX dimerization protein
0,92	NGFRAP1	nerve growth factor receptor (TNFRSF16) associated protein 1
0,92	COA5	cytochrome c oxidase assembly factor 5
0,92	MYO1F	myosin IF
0,92	FRYL	FRY like transcription coactivator
0,92	OPN1MW	opsin 1 (cone pigments), medium-wave-sensitive
0,92	MGEA5	meningioma expressed antigen 5 (hyaluronidase)
0,92	NDUFA10	NADH dehydrogenase (ubiquinone) 1 alpha subcomplex, 10, 42kDa
0,92	MAP2K1	mitogen-activated protein kinase kinase 1
0,92	NHS	Nance-Horan syndrome (congenital cataracts and dental anomalies)
0,92	FBXW10	F-box and WD repeat domain containing 10
0,92	SLC39A13	solute carrier family 39 (zinc transporter), member 13
0,92	OR4D1	olfactory receptor, family 4, subfamily D, member 1
0,92	C4orf26	chromosome 4 open reading frame 26
0,92	HDAC4	Transcript Identified by AceView, Entrez Gene ID(s) 9759
0,92	MGP	matrix Gla protein
0,92	NAP1L2	Transcript Identified by AceView, Entrez Gene ID(s) 4674
0,92	FFAR4	free fatty acid receptor 4
0,92	GPR37L1	G protein-coupled receptor 37 like 1
0,92	KLHL42	kelch-like family member 42
0,92	CEP295	centrosomal protein 295kDa
0,92	AP1G2	adaptor-related protein complex 1, gamma 2 subunit
0,92	MPRIP	myosin phosphatase Rho interacting protein
0,92	UBAC2	UBA domain containing 2
0,92	KRT82	keratin 82, type II
0,92	SEMA3G	sema domain, immunoglobulin domain (Ig), short basic domain, secreted, (semaphorin) 3G
0,92	SMG5	SMG5 nonsense mediated mRNA decay factor
0,92	EPHB4	EPH receptor B4
0,92	MFSD4	major facilitator superfamily domain containing 4
0,92	ZSCAN29	zinc finger and SCAN domain containing 29
0,92	RASSF2	Ras association (RalGDS/AF-6) domain family member 2
0,92	ST8SIA5	ST8 alpha-N-acetyl-neuraminide alpha-2,8-sialyltransferase 5
0,92	CISD3	CDGSH iron sulfur domain 3

0,92	LOC403312; AP000708.1	putative uncharacterized protein MGC39545; novel transcript, antisense to FEZ1
0,92	SRGAP1	SLIT-ROBO Rho GTPase activating protein 1
0,92	SERPINA4	serpin peptidase inhibitor, clade A (alpha-1 antiproteinase, antitrypsin), member 4
0,92	WDR70	WD repeat domain 70
0,92	GPR158	G protein-coupled receptor 158
0,92	ERGIC3	ERGIC and golgi 3
0,92	CLASP2	cytoplasmic linker associated protein 2
0,92	TMEM265; SRCAP	transmembrane protein 265; Snf2-related CREBBP activator protein
0,92	LRRC10B	leucine rich repeat containing 10B
0,92	ULBP3	UL16 binding protein 3
0,92	FMN1	formin 1
0,92	PNCK	pregnancy up-regulated nonubiquitous CaM kinase
0,92	ARHGEF4	Rho guanine nucleotide exchange factor 4
0,92	CEBPZ	CCAAT/enhancer binding protein (C/EBP), zeta
0,92	ITGAL	integrin alpha L
0,92	C7orf66	chromosome 7 open reading frame 66
0,92	PTPN20	protein tyrosine phosphatase, non-receptor type 20
0,92	CSF2	colony stimulating factor 2 (granulocyte-macrophage)
0,92	TTYH2	tweety family member 2
0,92	FOXJ1	forkhead box J1
0,92	FMN1	formin 1
0,92	NDUFB10	NADH dehydrogenase (ubiquinone) 1 beta subcomplex, 10, 22kDa
0,92	OR6P1	olfactory receptor, family 6, subfamily P, member 1
0,92	PALB2	partner and localizer of BRCA2
0,92	PIGK	phosphatidylinositol glycan anchor biosynthesis class K
0,92	QRSL1	Transcript Identified by AceView, Entrez Gene ID(s) 55278
0,92	DSTYK	dual serine/threonine and tyrosine protein kinase
0,92	SVIL	supervillin
0,92	RASA2	RAS p21 protein activator 2
0,92	PTH2	parathyroid hormone 2
0,92	SEC13	SEC13 homolog, nuclear pore and COPII coat complex component
0,92	HHAT	hedgehog acyltransferase
0,92	LETM1	leucine zipper-EF-hand containing transmembrane protein 1
0,92	PIM1	Pim-1 proto-oncogene, serine/threonine kinase
0,92	OR5L2	olfactory receptor, family 5, subfamily L, member 2
0,92	LDHAL6A	lactate dehydrogenase A-like 6A
0,92	TGFBR1	transforming growth factor, beta receptor 1
0,92	OR52R1	olfactory receptor, family 52, subfamily R, member 1 (gene/pseudogene)
0,92	CC2D2B	coiled-coil and C2 domain containing 2B
0,92	ACSS1	acyl-CoA synthetase short-chain family member 1
0,92	CEP57	centrosomal protein 57kDa

0,92	CSAG3	CSAG family, member 3
0,92	YY1; MIR6764	YY1 transcription factor; microRNA 6764
0,92	CYSTM1	cysteine-rich transmembrane module containing 1
0,92	MST1	macrophage stimulating 1
0,92	C9orf64	chromosome 9 open reading frame 64
0,92	DEFB127	defensin, beta 127
0,92	TMEM5	transmembrane protein 5
0,92	BCL2L14	BCL2-like 14 (apoptosis facilitator)
0,92	FBXW12	F-box and WD repeat domain containing 12
0,92	KLF13	Kruppel-like factor 13
0,92	NCAM2	Salzman2013 ALT_ACCEPTOR, ALT_DONOR, coding, INTERNAL, intronic best transcript NM_004540
0,92	MN1	meningioma (disrupted in balanced translocation) 1
0,92	ITGB3BP	integrin beta 3 binding protein (beta3-endonexin)
0,92	ABL2	ABL proto-oncogene 2, non-receptor tyrosine kinase
0,92	CRELD2	cysteine rich with EGF-like domains 2
0,92	GOLGA8M	golgin A8 family, member M
0,92	SMIM17	small integral membrane protein 17
0,92	DOCK7	dedicator of cytokinesis 7
0,92	FAM110A	family with sequence similarity 110, member A
0,92	TET2	tet methylcytosine dioxygenase 2
0,92	MYEF2	Transcript Identified by AceView, Entrez Gene ID(s) 50804
0,92	ARPC1B	actin related protein 2/3 complex subunit 1B
0,92	ATP6V1D	ATPase, H+ transporting, lysosomal 34kDa, V1 subunit D
0,92	CYBA	cytochrome b-245, alpha polypeptide
0,92	NDN	necdin, MAGE family member
0,92	FBLN7	fibulin 7
0,92	BICC1	BicC family RNA binding protein 1
0,92	NPVF	neuropeptide VF precursor
0,92	CACNB4	calcium channel, voltage-dependent, beta 4 subunit
0,92	OR4K13	olfactory receptor, family 4, subfamily K, member 13
0,92	SMOC2	SPARC related modular calcium binding 2
0,92	PDF; COG8; TMED6	peptide deformylase (mitochondrial); component of oligomeric golgi complex 8; transmembrane p24 trafficking protein 6
0,92	MGAT4EP	MGAT4 family, member E, pseudogene
0,92	FRK	fyn-related Src family tyrosine kinase
0,92	MYEOV2	myeloma overexpressed 2
0,92	THAP2	THAP domain containing, apoptosis associated protein 2
0,92	DGCR8; MIR1306; MIR3618	DGCR8 microprocessor complex subunit; microRNA 1306; microRNA 3618
0,92	AQP4	aquaporin 4
0,92	ROR1	receptor tyrosine kinase-like orphan receptor 1
0,92	HOXA5	homeobox A5
0,92	NBPF11	neuroblastoma breakpoint family, member 11

0,92	FUT11	fucosyltransferase 11 (alpha (1,3) fucosyltransferase)
0,92	PYGB	phosphorylase, glycogen; brain
0,92	CAST	calpastatin
0,92	PHF5A	PHD finger protein 5A
0,92	ANKRD35	ankyrin repeat domain 35
0,92	HGFAC	HGF activator
0,92	RPL27	ribosomal protein L27
0,92	GFRA2	GDNF family receptor alpha 2
0,92	ITGAE	integrin alpha E
0,92	PECAM1	platelet/endothelial cell adhesion molecule 1
0,92	CBFA2T3	core-binding factor, runt domain, alpha subunit 2; translocated to, 3
0,92	RNF113A	ring finger protein 113A
0,92	ARHGAP23	Rho GTPase activating protein 23
0,92	ARID5A	AT rich interactive domain 5A (MRF1-like)
0,92	NOXRED1	NADP-dependent oxidoreductase domain containing 1
0,92	LRRC45	leucine rich repeat containing 45
0,92	GPRC5B	G protein-coupled receptor, class C, group 5, member B
0,92	RNF187	ring finger protein 187
0,92	KLHDC4	Transcript Identified by AceView, Entrez Gene ID(s) 54758; 100129637
0,92	NR1D2	nuclear receptor subfamily 1, group D, member 2
0,92	PLXNB1	plexin B1
0,92	C2CD2	C2 calcium-dependent domain containing 2
0,92	BORCS8; MEF2B; MEF2BNB-MEF2B	BLOC-1 related complex subunit 8; myocyte enhancer factor 2B; MEF2BNB-MEF2B readthrough
0,92	ARID4A	AT rich interactive domain 4A (RBP1-like)
0,92	INA	internexin neuronal intermediate filament protein, alpha
0,92	HYOU1	hypoxia up-regulated 1
0,92	MAN2A1	mannosidase, alpha, class 2A, member 1
0,92	PSD2	pleckstrin and Sec7 domain containing 2
0,92	DPPA2	developmental pluripotency associated 2
0,92	C11orf70	chromosome 11 open reading frame 70
0,92	DOK4	docking protein 4
0,92	CYTH2	cytohesin 2
0,92	VASP	vasodilator-stimulated phosphoprotein
0,92	ANKRD50	ankyrin repeat domain 50
0,92	WDR49	WD repeat domain 49
0,92	FBXL16	F-box and leucine-rich repeat protein 16
0,92	ZNF346	zinc finger protein 346
0,92	RELB	v-rel avian reticuloendotheliosis viral oncogene homolog B
0,92	NTN3	netrin 3
0,92	ZNF284	zinc finger protein 284
0,92	CROT	carnitine O-octanoyltransferase

0,92	MROH8	maestro heat-like repeat family member 8
0,92	JADE1	jade family PHD finger 1
0,92	LYZL4	lysozyme-like 4
0,92	FRMD7	FERM domain containing 7
0,92	PTCHD1	patched domain containing 1
0,92	ZNF396	zinc finger protein 396
0,92	TKTL2	transketolase-like 2
0,92	TPD52L3	tumor protein D52-like 3
0,92	MMRN2	multimerin 2
0,92	RAB43	Memczak2013 ALT_ACCEPTOR, ALT_DONOR, coding, INTERNAL, intronic best transcript NM_001204883
0,92	CD82	CD82 molecule
0,92	HID1	HID1 domain containing
0,92	EML4	echinoderm microtubule associated protein like 4
0,92	SOCS7	suppressor of cytokine signaling 7
0,92	C1orf158	Transcript Identified by AceView, Entrez Gene ID(s) 93190
0,92	C5AR1	complement component 5a receptor 1
0,92	PC	pyruvate carboxylase
0,92	OR5J2	olfactory receptor, family 5, subfamily J, member 2
0,92	NOP14	NOP14 nucleolar protein
0,92	TM2D3	TM2 domain containing 3
0,92	MIR99AHG	mir-99a-let-7c cluster host gene
0,92	MEGF8	multiple EGF-like-domains 8
0,92	HEATR3	HEAT repeat containing 3
0,92	FMN1	formin 1
0,92	GYG2	glycogenin 2
0,92	FAM161A	family with sequence similarity 161, member A
0,92	OR8K5	olfactory receptor, family 8, subfamily K, member 5
0,92	ANKIB1	ankyrin repeat and IBR domain containing 1
0,92	PLEKHA6	pleckstrin homology domain containing, family A member 6
0,92	INTS7	integrator complex subunit 7
0,92	FOXN3	forkhead box N3
0,92	DDX50	DEAD (Asp-Glu-Ala-Asp) box polypeptide 50
0,92	PDE8A	phosphodiesterase 8A
0,92	CXCL11	chemokine (C-X-C motif) ligand 11
0,92	LGALS1	lectin, galactoside-binding, soluble, 1
0,92	DEFB125	defensin, beta 125
0,92	LOC283710; RP11-16E12.2; rukama	uncharacterized LOC283710; novel transcript; Transcript Identified by AceView
0,92	BIRC7	baculoviral IAP repeat containing 7
0,92	TAAR2	trace amine associated receptor 2
0,92	IL23A	interleukin 23, alpha subunit p19
0,92	CDC42EP5	CDC42 effector protein (Rho GTPase binding) 5
0,92	MGC45922; CTD-2568A17.5	uncharacterized LOC284365; uncharacterized LOC284365 [Source:EntrezGene;Acc:284365]; novel transcript

0,92	OR51V1	olfactory receptor, family 51, subfamily V, member 1
0,92	CNKSR2	connector enhancer of kinase suppressor of Ras 2
0,92	PPP3R2	protein phosphatase 3, regulatory subunit B, beta
0,92	HDAC9	histone deacetylase 9
0,92	KRT36	keratin 36, type I
0,92	SELE	selectin E
0,92	EZH2	Transcript Identified by AceView, Entrez Gene ID(s) 2146
0,92	MTERF2	mitochondrial transcription termination factor 2
0,92	LRTM1	leucine-rich repeats and transmembrane domains 1
0,92	LOC101928877; RP11-386B13.3; SLC25A4	uncharacterized LOC101928877; novel transcript; Transcript Identified by AceView, Entrez Gene ID(s) 291
0,92	HDAC3	histone deacetylase 3
0,92	ADAM12	ADAM metallopeptidase domain 12
0,92	CLN8	ceroid-lipofuscinosis, neuronal 8
0,92	BEND4	BEN domain containing 4
0,92	CCDC27	coiled-coil domain containing 27
0,92	STAT5B	Transcript Identified by AceView, Entrez Gene ID(s) 6777
0,92	WDR66	WD repeat domain 66
0,92	CDK15	cyclin-dependent kinase 15
0,92	SCAF8; TIAM2	SR-related CTD-associated factor 8; T-cell lymphoma invasion and metastasis 2
0,92	PRAMEF19	PRAME family member 19
0,92	TBPL1	TBP-like 1
0,92	TAS2R1	taste receptor, type 2, member 1
0,92	DNAJC4	DnaJ (Hsp40) homolog, subfamily C, member 4
0,92	SNRPN; SNURF; SNORD107; PWARSN	small nuclear ribonucleoprotein polypeptide N; SNRPN upstream reading frame; small nucleolar RNA, C/D box 107; Prader Willi/Angelman region RNA, SNRPN neighbor
0,92	FUCA1	fucosidase, alpha-L- 1, tissue
0,92	CYSLTR2	cysteinyl leukotriene receptor 2
0,92	MSH5; MSH5-SAPCD1; SAPCD1	mutS homolog 5; MSH5-SAPCD1 readthrough (NMD candidate); suppressor APC domain containing 1
0,92	FOXD2	forkhead box D2
0,92	MPP2	membrane protein, palmitoylated 2
0,92	EIF3D	eukaryotic translation initiation factor 3, subunit D
0,92	GULP1	GULP, engulfment adaptor PTB domain containing 1
0,92	OR5M10	olfactory receptor, family 5, subfamily M, member 10
0,92	MEIS1	Meis homeobox 1
0,92	MEX3D	mex-3 RNA binding family member D
0,92	EDN2	endothelin 2
0,92	RHPN1	rhophilin, Rho GTPase binding protein 1
0,92	OR10V1	olfactory receptor, family 10, subfamily V, member 1
0,92	HMBS	hydroxymethylbilane synthase
0,92	AHSG	alpha-2-HS-glycoprotein
0,92	SGCB	sarcoglycan beta

0,92	DENND5B	DENN/MADD domain containing 5B
0,92	PHPT1	phosphohistidine phosphatase 1
0,92	TNS4	tensin 4
0,92	PEX16	peroxisomal biogenesis factor 16
0,92	MS4A5	membrane-spanning 4-domains, subfamily A, member 5
0,92	GAB2	GRB2-associated binding protein 2
0,92	ENDOG	endonuclease G
0,92	PPAN-P2RY11; P2RY11; PPAN	PPAN-P2RY11 readthrough; purinergic receptor P2Y, G-protein coupled, 11; peter pan homolog (Drosophila)
0,92	MAPKBP1	mitogen-activated protein kinase binding protein 1
0,92	GALNT10	polypeptide N-acetylgalactosaminyltransferase 10
0,92	C1QL1	complement component 1, q subcomponent-like 1
0,92	JMJD6	jumonji domain containing 6
0,92	MAPK7	mitogen-activated protein kinase 7
0,92	OR10H3	olfactory receptor, family 10, subfamily H, member 3
0,92	DSEL	dermatan sulfate epimerase-like
0,92	OR2D2	olfactory receptor, family 2, subfamily D, member 2
0,92	GP2	glycoprotein 2 (zymogen granule membrane)
0,92	LMBR1	Transcript Identified by AceView, Entrez Gene ID(s) 64327
0,92	N4BP2	NEDD4 binding protein 2
0,92	GTPBP6	GTP binding protein 6 (putative)
0,92	TNRC18	trinucleotide repeat containing 18
0,92	BCL2L14	BCL2-like 14 (apoptosis facilitator)
0,92	VWC2L	von Willebrand factor C domain containing protein 2-like
0,92	GPR78	G protein-coupled receptor 78
0,92	PBLD	phenazine biosynthesis-like protein domain containing
0,92	ZNF501	zinc finger protein 501
0,92	LOC389199; AC097381.1	uncharacterized LOC389199; Transcript Identified by AceView, Entrez Gene ID(s) 389199; novel transcript
0,92	ZSCAN26	zinc finger and SCAN domain containing 26
0,92	FOXP4; MIR4641	forkhead box P4; microRNA 4641
0,92	SIRPB1	signal-regulatory protein beta 1
0,92	ZNF276	zinc finger protein 276
0,92	SERPINB6	serpin peptidase inhibitor, clade B (ovalbumin), member 6
0,92	NLRP6	NLR family, pyrin domain containing 6
0,92	TSPAN7	tetraspanin 7
0,92	RTP3	receptor (chemosensory) transporter protein 3
0,92	BNIP3	BCL2/adenovirus E1B 19kDa interacting protein 3
0,92	ANKRD36	Transcript Identified by AceView, Entrez Gene ID(s) 375248
0,92	KIFC1	kinesin family member C1
0,92	GABPB2	GA binding protein transcription factor, beta subunit 2
0,92	TDGF1	teratocarcinoma-derived growth factor 1

0,92	SF3B3	splicing factor 3b subunit 3
0,92	C2orf70	chromosome 2 open reading frame 70
0,92	ZNF79	zinc finger protein 79
0,92	WDR81	WD repeat domain 81
0,92	PEX5L	Transcript Identified by AceView, Entrez Gene ID(s) 51555
0,92	MON1A	MON1 secretory trafficking family member A
0,92	SLC37A1	solute carrier family 37 (glucose-6-phosphate transporter), member 1
0,92	SPECC1L	sperm antigen with calponin homology and coiled-coil domains 1-like
0,92	RC3H1	ring finger and CCCH-type domains 1
0,92	KANSL2; SNORA2B	KAT8 regulatory NSL complex subunit 2; small nucleolar RNA, H/ACA box 2B
0,92	IQGAP1	IQ motif containing GTPase activating protein 1
0,92	ZNF487	zinc finger protein 487
0,92	PCDHB8	protocadherin beta 8
0,92	BTBD9	BTB (POZ) domain containing 9
0,92	DGKD	diacylglycerol kinase, delta 130kDa
0,92	NMBR	neuromedin B receptor
0,92	ZNF175	zinc finger protein 175
0,92	SHOX2	short stature homeobox 2
0,92	IRF5	interferon regulatory factor 5
0,92	OR4K14	olfactory receptor, family 4, subfamily K, member 14
0,92	GNPDA1	glucosamine-6-phosphate deaminase 1
0,92	CPVL	carboxypeptidase, vitellogenic-like
0,92	KCNJ12	potassium channel, inwardly rectifying subfamily J, member 12
0,91	KIAA1755	KIAA1755
0,91	GCA	grancalcin, EF-hand calcium binding protein
0,91	ITGB2	integrin, beta 2 (complement component 3 receptor 3 and 4 subunit)
0,91	DRD2	dopamine receptor D2
0,91	STARD5	StAR-related lipid transfer domain containing 5
0,91	MARCH8	Transcript Identified by AceView, Entrez Gene ID(s) 220972
0,91	SERPINB1	serpin peptidase inhibitor, clade B (ovalbumin), member 1
0,91	KLHL21	kelch-like family member 21
0,91	PTPN14	protein tyrosine phosphatase, non-receptor type 14
0,91	PDE4DIP	Transcript Identified by AceView, Entrez Gene ID(s) 9659
0,91	C17orf67	chromosome 17 open reading frame 67
0,91	AOC1	amine oxidase, copper containing 1
0,91	FCGR3B	Fc fragment of IgG, low affinity IIIb, receptor (CD16b)
0,91	UBAP1L	ubiquitin associated protein 1 like
0,91	PPIAL4E	peptidylprolyl isomerase A (cyclophilin A)-like 4E
0,91	GPAT4	glycerol-3-phosphate acyltransferase 4

0,91	ADAMTS20	ADAM metallopeptidase with thrombospondin type 1 motif 20
0,91	CIB1	calcium and integrin binding 1 (calmyrin)
0,91	ROPN1	rhophilin associated tail protein 1
0,91	CCDC125	coiled-coil domain containing 125
0,91	PLIN1	perilipin 1
0,91	DNM2	dynamin 2
0,91	ISG20	interferon stimulated exonuclease gene 20kDa
0,91	TAS2R40	taste receptor, type 2, member 40
0,91	ZNF660	zinc finger protein 660
0,91	ACTA2	actin, alpha 2, smooth muscle, aorta
0,91	SLC25A41	solute carrier family 25, member 41
0,91	MATN1	matrilin 1, cartilage matrix protein
0,91	GCNT6	glucosaminyl (N-acetyl) transferase 6 [Source:HGNC Symbol;Acc:HGNC:21623]
0,91	TPI1	triosephosphate isomerase 1
0,91	ARGFX	arginine-fifty homeobox
0,91	PFAS	phosphoribosylformylglycinamidine synthase
0,91	SEC62	SEC62 homolog, preprotein translocation factor
0,91	DCP2	decapping mRNA 2
0,91	MCL1	myeloid cell leukemia 1
0,91	PRAMEF18	PRAME family member 18
0,91	BRMS1	breast cancer metastasis suppressor 1
0,91	PHOX2A	paired-like homeobox 2a
0,91	LRRIQ1	leucine-rich repeats and IQ motif containing 1
0,91	BMP2	bone morphogenetic protein 2
0,91	DNAH7	dynein, axonemal, heavy chain 7
0,91	HMBS	hydroxymethylbilane synthase
0,91	SLA2	Src-like-adaptor 2
0,91	PAM	peptidylglycine alpha-amidating monooxygenase
0,91	COA1	cytochrome c oxidase assembly factor 1 homolog
0,91	IFT140	intraflagellar transport 140
0,91	PDCD6	programmed cell death 6
0,91	SIGLEC11	sialic acid binding Ig-like lectin 11
0,91	TCF12	transcription factor 12
0,91	APBB2	Jeck2013 ALT_ACCEPTOR, ALT_DONOR, coding, INTERNAL, intronic best transcript NM_004307
0,91	NEUROD1	neuronal differentiation 1
0,91	SLC10A5	solute carrier family 10, member 5
0,91	FAM157A	family with sequence similarity 157, member A
0,91	BCAS4	breast carcinoma amplified sequence 4
0,91	FKBP2	FK506 binding protein 2
0,91	SLC22A5	solute carrier family 22 (organic cation/carnitine transporter), member 5
0,91	KIF1B	kinesin family member 1B
0,91	CMC4; MTCP1	C-x(9)-C motif containing 4; mature T-cell proliferation 1
0,91	LHX1	LIM homeobox 1
0,91	TACR2	tachykinin receptor 2

0,91	LOC101928034; S100A3	uncharacterized LOC101928034; Salzman2013 ANTISENSE, CDS, coding, INTERNAL, intronic, OVCODE, OVEXON, UTR5 best transcript NM_002960
0,91	ARL14EPL	ADP-ribosylation factor like GTPase 14 effector protein like
0,91	ODF2L	outer dense fiber of sperm tails 2-like
0,91	FAM109A	family with sequence similarity 109, member A
0,91	DKK4	dickkopf WNT signaling pathway inhibitor 4
0,91	COL9A3	collagen, type IX, alpha 3
0,91	KDM5A	lysine (K)-specific demethylase 5A
0,91	PM20D2	peptidase M20 domain containing 2
0,91	TMEM257	transmembrane protein 257
0,91	TMEM106C	transmembrane protein 106C
0,91	RGMB	repulsive guidance molecule family member b
0,91	OSGIN1	oxidative stress induced growth inhibitor 1
0,91	BLOC1S1	biogenesis of lysosomal organelles complex-1, subunit 1
0,91	FCRLA	Fc receptor-like A
0,91	HNRNPLL	heterogeneous nuclear ribonucleoprotein L-like
0,91	PSMB4	proteasome subunit beta 4
0,91	IFNA14	interferon, alpha 14
0,91	SYTL5	synaptotagmin-like 5
0,91	REST	RE1-silencing transcription factor
0,91	WDR83	WD repeat domain 83
0,91	ANOS1	anosmin 1
0,91	TRMT61A	tRNA methyltransferase 61A
0,91	MAP3K1	mitogen-activated protein kinase kinase kinase 1, E3 ubiquitin protein ligase
0,91	NDUFA10	NADH dehydrogenase (ubiquinone) 1 alpha subcomplex, 10, 42kDa
0,91	KRT10	keratin 10, type I
0,91	COL6A2	Jeck2013 ANTISENSE, CDS, coding, INTERNAL, intronic, OVCODE, OVEXON best transcript NM_058175
0,91	CES3; CES2	carboxylesterase 3; carboxylesterase 2
0,91	KIAA0232	KIAA0232
0,91	CHTOP	chromatin target of PRMT1
0,91	NRBP2; MIR6845	nuclear receptor binding protein 2; microRNA 6845
0,91	DCUN1D3	DCN1, defective in cullin neddylation 1, domain containing 3
0,91	NUP133	nucleoporin 133kDa
0,91	SLC22A31	solute carrier family 22, member 31
0,91	CASKIN1	CASK interacting protein 1
0,91	TRAPPC4	trafficking protein particle complex 4
0,91	CCL18	chemokine (C-C motif) ligand 18
0,91	PDX1	pancreatic and duodenal homeobox 1
0,91	GTF2IRD2; GTF2IRD2B	GTF2I repeat domain containing 2; GTF2I repeat domain containing 2B

0,91	TRIM36	tripartite motif containing 36
0,91	ATXN1	ataxin 1
0,91	GNB2	guanine nucleotide binding protein (G protein), beta polypeptide 2
0,91	CXCL9	chemokine (C-X-C motif) ligand 9
0,91	BPIFB4	BPI fold containing family B, member 4
0,91	SLC25A14	solute carrier family 25 (mitochondrial carrier, brain), member 14
0,91	NIT1	nitrilase 1
0,91	YIPF1	Yip1 domain family member 1
0,91	FAM149B1	family with sequence similarity 149, member B1
0,91	PALLD	palladin, cytoskeletal associated protein
0,91	SERPIND1	serpin peptidase inhibitor, clade D (heparin cofactor), member 1
0,91	NUPR1	nuclear protein 1, transcriptional regulator
0,91	ACSM2B	acyl-CoA synthetase medium-chain family member 2B
0,91	GUCA2A	guanylate cyclase activator 2A (guanylin)
0,91	DSE	dermatan sulfate epimerase
0,91	DUPD1	dual specificity phosphatase and pro isomerase domain containing 1
0,91	CABLES1	Cdk5 and Abl enzyme substrate 1
0,91	FAR2	fatty acyl-CoA reductase 2
0,91	ZFYVE26	zinc finger, FYVE domain containing 26
0,91	LONP1	lon peptidase 1, mitochondrial
0,91	PRKAR1B	protein kinase, cAMP-dependent, regulatory, type I, beta
0,91	MPI	mannose phosphate isomerase
0,91	ACOX2	acyl-CoA oxidase 2, branched chain
0,91	TTLL11	tubulin tyrosine ligase-like family member 11
0,91	ATG5	autophagy related 5
0,91	SFPQ	splicing factor proline/glutamine-rich
0,91	PPM1B	protein phosphatase, Mg2+/Mn2+ dependent, 1B
0,91	DNAJC13	DnaJ (Hsp40) homolog, subfamily C, member 13
0,91	TMEM52	transmembrane protein 52
0,91	KRTAP10-1	keratin associated protein 10-1
0,91	IGF1	insulin-like growth factor 1 (somatomedin C)
0,91	FADS2	fatty acid desaturase 2
0,91	DMXL1	Dmx-like 1
0,91	C2orf66	chromosome 2 open reading frame 66
0,91	RET	ret proto-oncogene
0,91	ALDH3B2	aldehyde dehydrogenase 3 family, member B2
0,91	CDC42SE2	CDC42 small effector 2
0,91	COG5	component of oligomeric golgi complex 5
0,91	ZNF292	zinc finger protein 292
0,91	C11orf96	chromosome 11 open reading frame 96
0,91	OR2T29	olfactory receptor, family 2, subfamily T, member 29
0,91	CFH	complement factor H
0,91	OR51H1	olfactory receptor, family 51, subfamily H, member 1
0,91	ZBTB46	zinc finger and BTB domain containing 46

0,91	THSD4	thrombospondin type 1 domain containing 4
0,91	SERPINA2	serpin peptidase inhibitor, clade A (alpha-1 antiproteinase, antitrypsin), member 2 (gene/pseudogene)
0,91	IGDCC4	immunoglobulin superfamily, DCC subclass, member 4
0,91	OMP	olfactory marker protein
0,91	SPIN4	spindlin family, member 4
0,91	JMJD7; PLA2G4B; JMJD7-PLA2G4B	jumonji domain containing 7; phospholipase A2, group IVB (cytosolic); JMJD7-PLA2G4B readthrough
0,91	BACE1	beta-site APP-cleaving enzyme 1
0,91	C19orf84	chromosome 19 open reading frame 84
0,91	ZNF700	zinc finger protein 700
0,91	SLC36A2	solute carrier family 36 (proton/amino acid symporter), member 2
0,91	SPATA45	spermatogenesis associated 45
0,91	MTA3	metastasis associated 1 family member 3
0,91	MAGI2	membrane associated guanylate kinase, WW and PDZ domain containing 2
0,91	PIFO	primary cilia formation
0,91	PPM1L	protein phosphatase, Mg2+/Mn2+ dependent, 1L
0,91	OXT	oxytocin/neurophysin I prepropeptide
0,91	GNPTAB	N-acetylglucosamine-1-phosphate transferase, alpha and beta subunits
0,91	PCDH9	Memczak2013 ALT_ACCEPTOR, ALT_DONOR, coding, INTERNAL, intronic best transcript NM_203487
0,91	GRB10	growth factor receptor bound protein 10
0,91	ATP6V1E2	ATPase, H+ transporting, lysosomal 31kDa, V1 subunit E2
0,91	CHRM3	cholinergic receptor, muscarinic 3
0,91	FAM169B	family with sequence similarity 169, member B
0,91	B4GALT5	UDP-Gal:betaGlcNAc beta 1,4- galactosyltransferase, polypeptide 5
0,91	SORCS3	sortilin-related VPS10 domain containing receptor 3
0,91	ARID3B	AT rich interactive domain 3B (BRIGHT-like)
0,91	RAB32	RAB32, member RAS oncogene family
0,91	MS4A7	membrane-spanning 4-domains, subfamily A, member 7
0,91	SIRT5	sirtuin 5
0,91	ZFP37	ZFP37 zinc finger protein
0,91	SDCCAG3	serologically defined colon cancer antigen 3
0,91	CEBPB	CCAAT/enhancer binding protein (C/EBP), beta
0,91	LGI3	leucine-rich repeat LGI family, member 3
0,91	REV1	REV1, DNA directed polymerase
0,91	CHCHD6	coiled-coil-helix-coiled-coil-helix domain containing 6
0,91	INPP4A	inositol polyphosphate-4-phosphatase type I A
0,91	E2F3	E2F transcription factor 3

0,91	PRLH	prolactin releasing hormone
0,91	CFI	complement factor I
0,91	SNTB2	syntrophin, beta 2 (dystrophin-associated protein A1, 59kDa, basic component 2)
0,91	TRIP12	thyroid hormone receptor interactor 12
0,91	EXOC3-AS1	EXOC3 antisense RNA 1
0,91	ZNF596	zinc finger protein 596
0,91	PRPF6	pre-mRNA processing factor 6
0,91	COL10A1	collagen, type X, alpha 1
0,91	AFF2	AF4/FMR2 family, member 2
0,91	WAS	Wiskott-Aldrich syndrome
0,91	SPAG5	sperm associated antigen 5
0,91	UBXN11	UBX domain protein 11
0,91	KCNK9	potassium channel, two pore domain subfamily K, member 9
0,91	RHBDL1	rhomboid, veinlet-like 1 (Drosophila)
0,91	GCNT4	glucosaminyl (N-acetyl) transferase 4, core 2
0,91	HYOU1	hypoxia up-regulated 1
0,91	SH3GL3	SH3-domain GRB2-like 3
0,91	DBNL; MIR6837	drebrin-like; microRNA 6837
0,91	CDKN1A	cyclin-dependent kinase inhibitor 1A (p21, Cip1)
0,91	TXLNA	taxilin alpha
0,91	GCK	glucokinase (hexokinase 4)
0,91	RHOC	ras homolog family member C
0,91	GALK1	galactokinase 1
0,91	ITPKC	inositol-trisphosphate 3-kinase C
0,91	GPC1	glypican 1
0,91	IFNA21	interferon, alpha 21
0,91	TRIM43B	tripartite motif containing 43B
0,91	ERMN	ermin
0,91	OR4A47	olfactory receptor, family 4, subfamily A, member 47
0,91	ARMCX6	armadillo repeat containing, X-linked 6
0,91	DEFB107B; DEFB107A	defensin, beta 107B; defensin, beta 107A
0,91	HIST1H1A	histone cluster 1, H1a
0,91	OPTC	opticin
0,91	LZTFL1	leucine zipper transcription factor like 1
0,91	OR6X1	olfactory receptor, family 6, subfamily X, member 1
0,91	TACO1	translational activator of mitochondrially encoded cytochrome c oxidase I
0,91	ZNF573	Transcript Identified by AceView, Entrez Gene ID(s) 126231
0,91	BCL9L	B-cell CLL/lymphoma 9-like
0,91	KSR1	kinase suppressor of ras 1
0,91	EMP2	epithelial membrane protein 2
0,91	DIAPH2	diaphanous-related formin 2
0,91	TMEM214	transmembrane protein 214
0,91	GNAZ	guanine nucleotide binding protein (G protein), alpha z polypeptide
0,91	ADCY7	adenylate cyclase 7

0,91	ZNF141	zinc finger protein 141
0,91	RIMKLA	ribosomal modification protein rimK-like family member A
0,91	RSRC2	arginine/serine-rich coiled-coil 2
0,91	GPCPD1	glycerophosphocholine phosphodiesterase 1
0,91	LIMS2	LIM and senescent cell antigen-like domains 2
0,91	IQUB	IQ motif and ubiquitin domain containing
0,91	PLXND1	plexin D1
0,91	CXXC4	CXXC finger protein 4
0,91	CNTN1	contactin 1
0,91	DUS4L	dihydrouridine synthase 4-like
0,91	ATP6V1B1	ATPase, H+ transporting, lysosomal 56/58kDa, V1 subunit B1
0,91	OR52E8	olfactory receptor, family 52, subfamily E, member 8
0,91	CPSF4L	cleavage and polyadenylation specific factor 4-like
0,91	ARMC9	armadillo repeat containing 9
0,91	KMT2A	lysine (K)-specific methyltransferase 2A
0,91	PRR23C	proline rich 23C
0,91	TCF20	transcription factor 20 (AR1)
0,91	TCF20	transcription factor 20 (AR1)
0,91	CXorf51A; CXorf51B	chromosome X open reading frame 51A; chromosome X open reading frame 51B
0,91	TSC22D4	TSC22 domain family, member 4
0,91	TRPC1	transient receptor potential cation channel, subfamily C, member 1
0,91	C9orf135-AS1	C9orf135 antisense RNA 1 (head to head)
0,91	CCSAP	centriole, cilia and spindle-associated protein
0,91	CRYAB	crystallin alpha B
0,91	LCE1C	late cornified envelope 1C
0,91	PTAFR	platelet-activating factor receptor
0,91	C7orf76; SHFM1	chromosome 7 open reading frame 76; split hand/foot malformation (ectrodactyly) type 1
0,91	BUD31	BUD31 homolog
0,91	C2orf73	chromosome 2 open reading frame 73
0,91	SPATA25	spermatogenesis associated 25
0,91	ZNF524	zinc finger protein 524
0,91	HDX	highly divergent homeobox
0,91	MC3R	melanocortin 3 receptor
0,91	ACSL1	acyl-CoA synthetase long-chain family member 1
0,91	CCNY	cyclin Y
0,91	TPR	translocated promoter region, nuclear basket protein
0,91	FBXO2	F-box protein 2
0,91	MALSU1	mitochondrial assembly of ribosomal large subunit 1
0,91	TCTE1	t-complex-associated-testis-expressed 1
0,91	CHIA	chitinase, acidic
0,91	TCEB3B	transcription elongation factor B polypeptide 3B (elongin A2)
0,91	CCDC74A	coiled-coil domain containing 74A
0,91	OBSCN	obscurin, cytoskeletal calmodulin and titin-interacting RhoGEF

0,91	DENND2D	DENN/MADD domain containing 2D
0,91	MUM1	melanoma associated antigen (mutated) 1
0,91	RNF7	ring finger protein 7
0,91	SMC5	structural maintenance of chromosomes 5
0,91	LXN	latexin
0,91	RSPO3	R-spondin 3
0,91	EPHA1	EPH receptor A1
0,91	MBD3	methyl-CpG binding domain protein 3
0,91	ABCD2	Transcript Identified by AceView, Entrez Gene ID(s) 225
0,91	CDON	cell adhesion associated, oncogene regulated
0,91	SDC3	syndecan 3
0,91	PANX3	pannexin 3
0,91	UBE2S	ubiquitin-conjugating enzyme E2S
0,91	PAN2; CNPY2	PAN2 poly(A) specific ribonuclease subunit; canopy FGF signaling regulator 2
0,91	TMEM191B	transmembrane protein 191B
0,91	SMAD7	Memczak2013 ALT_ACCEPTOR, ALT_DONOR, coding, INTERNAL, intronic best transcript NM_001190821
0,91	MRPL52	mitochondrial ribosomal protein L52
0,91	MACF1; KIAA0754	microtubule-actin crosslinking factor 1; KIAA0754
0,91	SHARPIN	SHANK-associated RH domain interactor
0,91	ZBTB1	zinc finger and BTB domain containing 1
0,91	EED; MIR6755	embryonic ectoderm development; microRNA 6755
0,91	FCRLB	Fc receptor-like B
0,91	UBE3D	ubiquitin protein ligase E3D
0,91	FBRSL1	fibrosin-like 1
0,91	EFCAB13	EF-hand calcium binding domain 13
0,91	CCDC186; MIR2110	coiled-coil domain containing 186; microRNA 2110
0,91	POLR3GL	polymerase (RNA) III (DNA directed) polypeptide G (32kD)-like
0,91	NTF3	neurotrophin 3
0,91	PARP2	poly(ADP-ribose) polymerase 2
0,91	TAS2R5	taste receptor, type 2, member 5
0,91	EGLN1	egl-9 family hypoxia-inducible factor 1
0,91	PPIAL4A	peptidylprolyl isomerase A (cyclophilin A)-like 4A
0,91	ZNF710	zinc finger protein 710
0,91	OR5M8	olfactory receptor, family 5, subfamily M, member 8
0,91	CBFA2T2	core-binding factor, runt domain, alpha subunit 2; translocated to, 2
0,91	RRP7A	ribosomal RNA processing 7 homolog A
0,91	PFDN4	prefoldin subunit 4
0,91	SEC16A	SEC16 homolog A, endoplasmic reticulum export factor
0,91	SPINT2	serine peptidase inhibitor, Kunitz type, 2
0,91	BPI	bactericidal/permeability-increasing protein

0,91	RP5-1091N2.9; IL2RG	Transcript Identified by AceView, Entrez Gene ID(s) 3561; putative novel transcript
0,91	FAM170A	family with sequence similarity 170, member A
0,91	PKIA	protein kinase (cAMP-dependent, catalytic) inhibitor alpha
0,91	FAM46A	family with sequence similarity 46, member A
0,91	SYNE1	Memczak2013 ALT_ACCEPTOR, ALT_DONOR, coding, INTERNAL, intronic best transcript NM_033071
0,91	OR10J5	olfactory receptor, family 10, subfamily J, member 5
0,91	CRIP2	cysteine-rich protein 2
0,91	PRG2	proteoglycan 2, bone marrow (natural killer cell activator, eosinophil granule major basic protein)
0,91	GGH	gamma-glutamyl hydrolase (conjugase, folylpolygammaglutamyl hydrolase)
0,91	ANGPTL1	angiopoietin like 1
0,91	KCNK4	potassium channel, two pore domain subfamily K, member 4
0,91	CD58	CD58 molecule
0,91	ACTL8	actin-like 8
0,91	PYCR2; MIR6741	pyrroline-5-carboxylate reductase family, member 2; microRNA 6741
0,91	LACE1	lactation elevated 1
0,91	MFSD7	major facilitator superfamily domain containing 7
0,91	COL4A1	collagen, type IV, alpha 1
0,91	STK35	serine/threonine kinase 35
0,91	PNPLA1	patatin-like phospholipase domain containing 1
0,91	RANBP1	Zhang2013 ALT_ACCEPTOR, ALT_DONOR, coding, INTERNAL, intronic best transcript NM_002882
0,91	PRR22	proline rich 22
0,91	FOXN1	forkhead box N1
0,91	OTUD6B	OTU domain containing 6B
0,91	ZSWIM5	zinc finger, SWIM-type containing 5
0,91	CLPSL2	colipase-like 2
0,91	GRID1	glutamate receptor, ionotropic, delta 1
0,91	PCTP	phosphatidylcholine transfer protein
0,91	TMEM79	transmembrane protein 79
0,91	FAM73A	family with sequence similarity 73, member A
0,91	IL17RC	interleukin 17 receptor C
0,91	ZC3H12D	zinc finger CCCH-type containing 12D
0,91	HYOU1	hypoxia up-regulated 1
0,91	PIN1	peptidylprolyl cis/trans isomerase, NIMA-interacting 1
0,91	ZNF282	zinc finger protein 282
0,91	DCAF8L1	DDB1 and CUL4 associated factor 8-like 1
0,91	AHNAK2	AHNAK nucleoprotein 2
0,91	RORA	RAR-related orphan receptor A
0,91	IL1R2	interleukin 1 receptor, type II
0,91	AP3S1	adaptor-related protein complex 3, sigma 1 subunit

0,91	HYOU1	hypoxia up-regulated 1
0,91	C16orf86	chromosome 16 open reading frame 86
0,91	CXXC5	CXXC finger protein 5
0,91	FAM179B	family with sequence similarity 179, member B
0,91	CCDC177	coiled-coil domain containing 177
0,91	BHLHE22	basic helix-loop-helix family, member e22
0,91	PRR21	proline rich 21
0,91	ASAP3	ArfGAP with SH3 domain, ankyrin repeat and PH domain 3
0,91	RCAN1	regulator of calcineurin 1
0,91	SLC22A20	solute carrier family 22, member 20
0,91	AJAP1	adherens junctions associated protein 1
0,91	MS4A1	membrane-spanning 4-domains, subfamily A, member 1
0,91	SLC10A4	solute carrier family 10, member 4
0,91	C12orf60	chromosome 12 open reading frame 60
0,91	UNC79	unc-79 homolog (C. elegans)
0,91	HOXB2	homeobox B2
0,91	HIPK1	Memczak2013 ALT_DONOR, coding, INTERNAL, intronic best transcript NM_198268
0,91	goyborbu; MCM2	Transcript Identified by AceView; Memczak2013 ANTISENSE, CDS, coding, INTERNAL, UTR3 best transcript NM_004526
0,91	SNRK	SNF related kinase
0,91	DDX5	Memczak2013 ANTISENSE, CDS, coding, INTERNAL best transcript NM_004396
0,91	AKR1E2	aldo-keto reductase family 1, member E2
0,91	GPSM2	G-protein signaling modulator 2
0,91	OR4C3	olfactory receptor, family 4, subfamily C, member 3
0,91	ASPM	abnormal spindle microtubule assembly
0,91	SMKR1	small lysine-rich protein 1
0,91	PHF10	PHD finger protein 10
0,91	ZNF771	zinc finger protein 771
0,91	ADH4	alcohol dehydrogenase 4 (class II), pi polypeptide
0,91	PLCG2	phospholipase C, gamma 2 (phosphatidylinositol-specific)
0,91	PLSCR2	phospholipid scramblase 2
0,91	OR2AP1	olfactory receptor, family 2, subfamily AP, member 1
0,91	QSOX2	quiescin Q6 sulfhydryl oxidase 2
0,91	SRA1	steroid receptor RNA activator 1
0,91	SIRT2	sirtuin 2
0,91	ECM2	extracellular matrix protein 2, female organ and adipocyte specific
0,91	MEIS3	Meis homeobox 3
0,91	RBKS; BRE-AS1	ribokinase; BRE antisense RNA 1
0,91	ZFP91	ZFP91 zinc finger protein
0,91	ARHGEF2	Rho/Rac guanine nucleotide exchange factor 2
0,91	RNF182	ring finger protein 182
0,91	LARGE	like-glycosyltransferase
0,91	SIK1	salt-inducible kinase 1

0,91	HSF5	heat shock transcription factor family member 5
0,91	SESN3	sestrin 3
0,91	AP4M1	adaptor-related protein complex 4, mu 1 subunit
0,91	TEDDM1	transmembrane epididymal protein 1
0,91	CDKN2C	cyclin-dependent kinase inhibitor 2C (p18, inhibits CDK4)
0,91	ITGB6	integrin beta 6
0,91	PNMA2	paraneoplastic Ma antigen 2
0,91	SNAPIN	SNAP-associated protein
0,91	WBSCR16	Williams-Beuren syndrome chromosome region 16
0,91	RPS6KA1	ribosomal protein S6 kinase, 90kDa, polypeptide 1
0,91	VASH1	vasohibin 1
0,91	C1orf53	chromosome 1 open reading frame 53
0,91	NAT8L	N-acetyltransferase 8-like (GCN5-related, putative)
0,91	LITAF	lipopolysaccharide-induced TNF factor
0,91	SLC6A20	solute carrier family 6 (proline IMINO transporter), member 20
0,91	DEFB135	defensin, beta 135
0,91	COBLL1	cordon-bleu WH2 repeat protein like 1
0,91	C2orf71	chromosome 2 open reading frame 71
0,91	TPD52	tumor protein D52
0,91	STAT3	signal transducer and activator of transcription 3 (acute-phase response factor)
0,91	HLA-DOB	major histocompatibility complex, class II, DO beta
0,91	CAPN12	calpain 12
0,91	HBP1	HMG-box transcription factor 1
0,91	ANTXR1	anthrax toxin receptor 1
0,91	SLC25A42	solute carrier family 25, member 42
0,91	APOA1	apolipoprotein A-I
0,91	FAM102B	family with sequence similarity 102, member B
0,91	RNF181	ring finger protein 181
0,91	CNTRL	centriolin
0,91	MPG	N-methylpurine DNA glycosylase
0,91	HSPA13	heat shock protein 70kDa family, member 13
0,91	DAZ4; DAZ1	deleted in azoospermia 4; deleted in azoospermia 1
0,91	SAG	S-antigen; retina and pineal gland (arrestin)
0,91	HS3ST1	heparan sulfate (glucosamine) 3-O-sulfotransferase 1
0,91	SMARCA5	SWI/SNF related, matrix associated, actin dependent regulator of chromatin, subfamily a, member 5
0,91	OPTN	optineurin
0,91	CD1E	CD1e molecule
0,91	LILRA6	leukocyte immunoglobulin-like receptor, subfamily A (with TM domain), member 6
0,91	TMEM150A	transmembrane protein 150A
0,91	MYH10	myosin, heavy chain 10, non-muscle
0,91	OR1L8	olfactory receptor, family 1, subfamily L, member 8
0,91	MYRIP	myosin VIIA and Rab interacting protein
0,91	LONRF2	LON peptidase N-terminal domain and ring finger 2
0,91	SLC10A6	solute carrier family 10 (sodium/bile acid cotransporter), member 6

0,91	NIPSNAP3A; NIPSNAP3B	nipsnap homolog 3A (C. elegans); nipsnap homolog 3B (C. elegans)
0,91	SERPINA13P	serpin peptidase inhibitor, clade A (alpha-1 antiproteinase, antitrypsin), member 13, pseudogene
0,91	APOPT1	apoptogenic 1, mitochondrial
0,91	TMEM133	transmembrane protein 133
0,91	MVK	mevalonate kinase
0,91	NAV2	neuron navigator 2
0,91	SUPT3H	SPT3 homolog, SAGA and STAGA complex component
0,91	APAF1	apoptotic peptidase activating factor 1
0,91	DZIP3	DAZ interacting zinc finger protein 3
0,91	GGT7	gamma-glutamyltransferase 7
0,91	MBTPS1	membrane bound transcription factor peptidase, site 1
0,91	CATSPER4	cation channel, sperm associated 4
0,91	SEMA3F	sema domain, immunoglobulin domain (Ig), short basic domain, secreted, (semaphorin) 3F
0,91	TRIB3	tribbles pseudokinase 3
0,91	OR5AN1	olfactory receptor, family 5, subfamily AN, member 1
0,91	SLC8B1	solute carrier family 8 (sodium/lithium/calcium exchanger), member B1
0,91	FAM3B	family with sequence similarity 3, member B
0,91	TEAD2	TEA domain family member 2
0,91	PPIC	peptidylprolyl isomerase C (cyclophilin C)
0,91	IL24	interleukin 24
0,91	GBX1	gastrulation brain homeobox 1
0,91	RABGAP1L	RAB GTPase activating protein 1-like
0,91	PFKL	phosphofructokinase, liver
0,91	HGF	hepatocyte growth factor (hepapoietin A; scatter factor)
0,91	MSLN	mesothelin
0,91	SLX4IP	SLX4 interacting protein
0,91	SDPR	serum deprivation response
0,91	GOLGA6L22	golgin A6 family-like 22
0,91	BTBD17	BTB (POZ) domain containing 17
0,91	LRRTM1	leucine rich repeat transmembrane neuronal 1
0,91	FABP6	fatty acid binding protein 6, ileal
0,91	CGB1	chorionic gonadotropin, beta polypeptide 1
0,91	EFNB3	ephrin-B3
0,91	SGCZ	sarcoglycan zeta
0,91	NUMA1	nuclear mitotic apparatus protein 1
0,91	AADAC	arylacetamide deacetylase
0,91	CSN3	casein kappa
0,91	UGT2A3	UDP glucuronosyltransferase 2 family, polypeptide A3
0,91	GPR84	G protein-coupled receptor 84
0,91	RPS6KA1	ribosomal protein S6 kinase, 90kDa, polypeptide 1
0,91	TCAP	titin-cap
0,91	ADAT1	adenosine deaminase, tRNA-specific 1

0,91	IRF7	interferon regulatory factor 7
0,91	MON2	MON2 homolog, regulator of endosome-to-Golgi trafficking
0,91	KRTAP3-2	keratin associated protein 3-2
0,91	CHIT1	chitinase 1 (chitotriosidase)
0,91	FGB	fibrinogen beta chain
0,91	PGM1	phosphoglucomutase 1
0,91	SAP30	Sin3A associated protein 30kDa
0,91	HP09025; CTD-2116F7.1	uncharacterized LOC100652929; novel transcript; uncharacterized LOC100652929 [Source:EntrezGene;Acc:100652929]
0,91	GPR137C	G protein-coupled receptor 137C
0,91	OR4M2; AC171558.2	Homo sapiens olfactory receptor, family 4, subfamily M, member 2 (OR4M2), mRNA.; Homo sapiens olfactory receptor, family 4, subfamily M, member 2, mRNA (cDNA clone MGC:168600 IMAGE:9020977), complete cds.
0,91	MALRD1	MAM and LDL receptor class A domain containing 1
0,91	KIAA0141	KIAA0141
0,91	PIGX	phosphatidylinositol glycan anchor biosynthesis class X
0,91	ACTR3C	ARP3 actin-related protein 3 homolog C (yeast)
0,91	KRT72	keratin 72, type II
0,91	ZNF592	zinc finger protein 592
0,91	NBPF20	neuroblastoma breakpoint family, member 20
0,91	RHCE	Rh blood group, CcEe antigens
0,91	OR4F5	olfactory receptor, family 4, subfamily F, member 5
0,91	FAM47E; FAM47E-STBD1; STBD1	family with sequence similarity 47, member E; FAM47E-STBD1 readthrough; starch binding domain 1
0,91	BNIP1	BCL2/adenovirus E1B 19kDa interacting protein 1
0,91	FBXL20	F-box and leucine-rich repeat protein 20
0,91	MTSS1	metastasis suppressor 1
0,91	TTC7B	tetratricopeptide repeat domain 7B
0,91	SLC7A7	solute carrier family 7 (amino acid transporter light chain, y+L system), member 7
0,91	OR51B2	olfactory receptor, family 51, subfamily B, member 2 (gene/pseudogene)
0,91	EGR3	early growth response 3
0,91	SPACA3	sperm acrosome associated 3
0,91	KRTAP25-1	keratin associated protein 25-1
0,91	JADE3	jade family PHD finger 3
0,91	AGPAT4	Transcript Identified by AceView, Entrez Gene ID(s) 56895
0,91	BIRC5	baculoviral IAP repeat containing 5
0,91	TIAF1; MYO18A	TGFB1-induced anti-apoptotic factor 1; myosin XVIIIA
0,91	LRRC75A	leucine rich repeat containing 75A
0,91	FIGLA	folliculogenesis specific bHLH transcription factor
0,91	EXOC3L4	exocyst complex component 3-like 4
0,91	FBLIM1	filamin binding LIM protein 1

0,91	PEX26	peroxisomal biogenesis factor 26
0,91	CNIH2	cornichon family AMPA receptor auxiliary protein 2
0,91	GAN; MIR4720	gigaxonin; microRNA 4720
0,91	KCNA10	potassium channel, voltage gated shaker related subfamily A, member 10
0,91	CASP7	caspase 7
0,91	RAB8B	RAB8B, member RAS oncogene family
0,91	LOC730159; RP11-360D2.1	uncharacterized LOC730159; putative novel transcript
0,91	FER	fer (fps/fes related) tyrosine kinase
0,91	CLGN	calmegin
0,91	CCL4L2; CCL4L1	chemokine (C-C motif) ligand 4-like 2; chemokine (C-C motif) ligand 4-like 1
0,91	POLR2F; MIR4534; MIR6820	polymerase (RNA) II (DNA directed) polypeptide F; microRNA 4534; microRNA 6820
0,91	B3GNT8	UDP-GlcNAc:betaGal beta-1,3-N-acetylglucosaminyltransferase 8
0,91	RRBP1	ribosome binding protein 1
0,91	MAN2B2	mannosidase, alpha, class 2B, member 2
0,91	MTCL1	microtubule crosslinking factor 1
0,90	EMC7	ER membrane protein complex subunit 7
0,90	PLA2G3	phospholipase A2, group III
0,90	CARNS1	carnosine synthase 1
0,90	S100A2	S100 calcium binding protein A2
0,90	PPIP5K1	diphosphoinositol pentakisphosphate kinase 1
0,90	PSMG4	proteasome (prosome, macropain) assembly chaperone 4
0,90	ARFGAP2	ADP-ribosylation factor GTPase activating protein 2
0,90	FMN1	formin 1
0,90	TCF20	transcription factor 20 (AR1)
0,90	TCF20	transcription factor 20 (AR1)
0,90	CEP350	centrosomal protein 350kDa
0,90	PKDCC	protein kinase domain containing, cytoplasmic
0,90	LILRA5	Memczak2013 ANTISENSE, coding, INTERNAL, intronic best transcript NM_181985
0,90	KIAA1671	KIAA1671
0,90	MUS81	MUS81 structure-specific endonuclease subunit
0,90	PRTN3	proteinase 3
0,90	COG4	component of oligomeric golgi complex 4
0,90	MRPL17	mitochondrial ribosomal protein L17
0,90	C22orf42	chromosome 22 open reading frame 42
0,90	LMNA	lamin A/C
0,90	MTERF4	mitochondrial transcription termination factor 4
0,90	SFXN4	sideroflexin 4
0,90	TRIM40	tripartite motif containing 40
0,90	DCTN3	dynactin 3 (p22)
0,90	FAM162A	family with sequence similarity 162, member A
0,90	MAP3K12	mitogen-activated protein kinase kinase kinase 12
0,90	ARPC5L	actin related protein 2/3 complex subunit 5-like

0,90	RPL7A	ribosomal protein L7a
0,90	KRTAP4-4	keratin associated protein 4-4
0,90	ANKRD36	ankyrin repeat domain 36
0,90	AGPAT4-IT1	AGPAT4 intronic transcript 1
0,90	INSIG2	insulin induced gene 2
0,90	CXADR	coxsackie virus and adenovirus receptor
0,90	CHSY1	chondroitin sulfate synthase 1
0,90	VKORC1	vitamin K epoxide reductase complex subunit 1
0,90	ZHX1-C8orf76	ZHX1-C8orf76 readthrough
0,90	DNMBP	dynamin binding protein
0,90	ZNF385C	zinc finger protein 385C
0,90	HMOX1	heme oxygenase 1
0,90	URB1	URB1 ribosome biogenesis 1 homolog (S. cerevisiae)
0,90	TPCN2	two pore segment channel 2
0,90	OR5T3	olfactory receptor, family 5, subfamily T, member 3
0,90	AQP12B	aquaporin 12B
0,90	ADAP2	ArfGAP with dual PH domains 2
0,90	PNLDC1	poly(A)-specific ribonuclease (PARN)-like domain containing 1
0,90	ZNF239	zinc finger protein 239
0,90	GNG11	guanine nucleotide binding protein (G protein), gamma 11
0,90	OOEP	oocyte expressed protein
0,90	SCRIB	Memczak2013 ANTISENSE, CDS, coding, INTERNAL best transcript NM_015356
0,90	HAUS1	HAUS augmin like complex subunit 1
0,90	CBX5; MIR3198-2	chromobox homolog 5; microRNA 3198-2
0,90	MKNK2	MAP kinase interacting serine/threonine kinase 2
0,90	MUC7	mucin 7, secreted
0,90	RRH	retinal pigment epithelium-derived rhodopsin homolog
0,90	CEBPA	CCAAT/enhancer binding protein (C/EBP), alpha
0,90	SPTLC1	Transcript Identified by AceView, Entrez Gene ID(s) 10558
0,90	EPHA8	EPH receptor A8
0,90	COPG1	coatomer protein complex subunit gamma 1
0,90	VWA5B1	von Willebrand factor A domain containing 5B1
0,90	MSL1	male-specific lethal 1 homolog (Drosophila)
0,90	PIK3R1	phosphoinositide-3-kinase, regulatory subunit 1 (alpha)
0,90	COQ2	coenzyme Q2 4-hydroxybenzoate polyprenyltransferase
0,90	FDXR	Zhang2013 ALT_ACCEPTOR, ALT_DONOR, coding, INTERNAL, intronic best transcript NM_004110
0,90	KRT34	keratin 34, type I
0,90	NBR1	neighbor of BRCA1 gene 1
0,90	GRIK1	glutamate receptor, ionotropic, kainate 1
0,90	LPA	lipoprotein, Lp(a)
0,90	CERK	ceramide kinase

0,90	SMC1A	structural maintenance of chromosomes 1A
0,90	DUX4	double homeobox 4
0,90	ADPRHL1	ADP-ribosylhydrolase like 1
0,90	CACHD1	cache domain containing 1
0,90	PPIH	peptidylprolyl isomerase H (cyclophilin H)
0,90	RLIM	ring finger protein, LIM domain interacting
0,90	SH3PXD2A	SH3 and PX domains 2A
0,90	GAS2L1	growth arrest-specific 2 like 1
0,90	CLEC14A	C-type lectin domain family 14, member A
0,90	SH3BGRL	SH3 domain binding glutamate-rich protein like
0,90	TBL2	transducin (beta)-like 2
0,90	CHD6	chromodomain helicase DNA binding protein 6
0,90	THY1	Thy-1 cell surface antigen
0,90	TAOK1; MIR4523	TAO kinase 1; microRNA 4523
0,90	GBF1	golgi brefeldin A resistant guanine nucleotide exchange factor 1
0,90	PPP2R5B	protein phosphatase 2, regulatory subunit B, beta
0,90	NLRP7	NLR family, pyrin domain containing 7
0,90	NUDCD3	NudC domain containing 3
0,90	RALGAPA1	Ral GTPase activating protein, alpha subunit 1 (catalytic)
0,90	APOBEC3G	apolipoprotein B mRNA editing enzyme, catalytic polypeptide-like 3G
0,90	HADH	hydroxyacyl-CoA dehydrogenase
0,90	TLR2	toll-like receptor 2
0,90	C1orf174	chromosome 1 open reading frame 174
0,90	NIPBL	Nipped-B homolog (Drosophila)
0,90	BCL2L14	BCL2-like 14 (apoptosis facilitator)
0,90	MYO5C	myosin VC
0,90	AKR1D1	aldo-keto reductase family 1, member D1
0,90	ABHD16A	abhydrolase domain containing 16A
0,90	TMEM106A	transmembrane protein 106A
0,90	KIF17	kinesin family member 17
0,90	MEI1	meiotic double-stranded break formation protein 1
0,90	AKAP3	A kinase (PRKA) anchor protein 3
0,90	FAT2	FAT atypical cadherin 2
0,90	OVOL2	ovo-like zinc finger 2
0,90	TCF20	transcription factor 20 (AR1)
0,90	TCF20	transcription factor 20 (AR1)
0,90	RD3	retinal degeneration 3
0,90	HRAS	Harvey rat sarcoma viral oncogene homolog
0,90	C5orf67	chromosome 5 open reading frame 67
0,90	NFRKB	Transcript Identified by AceView, Entrez Gene ID(s) 4798
0,90	FCAR	Fc fragment of IgA receptor
0,90	PRRC2B	proline-rich coiled-coil 2B
0,90	C16orf89	chromosome 16 open reading frame 89
0,90	KRTAP9-7	keratin associated protein 9-7
0,90	SLC16A11	solute carrier family 16, member 11

0,90	C11orf86	chromosome 11 open reading frame 86
0,90	OLFML2A	olfactomedin like 2A
0,90	GDPD1	glycerophosphodiester phosphodiesterase domain containing 1
0,90	POMZP3	POM121 and ZP3 fusion
0,90	SETD2	SET domain containing 2
0,90	H2AFJ	H2A histone family, member J
0,90	TCAF1	TRPM8 channel-associated factor 1
0,90	DGCR6; LOC102724770	Homo sapiens DiGeorge syndrome critical region gene 6 (DGCR6), mRNA.; protein DGCR6; Homo sapiens DiGeorge syndrome critical region gene 6, mRNA (cDNA clone MGC:54086 IMAGE:5229172), complete cds.; Protein DGCR6 [Source:UniProtKB/Swiss-Prot;Acc:Q14129]
0,90	C12orf57; RNU7-1	chromosome 12 open reading frame 57; RNA, U7 small nuclear 1
0,90	NEK6	NIMA-related kinase 6
0,90	FNDC4	fibronectin type III domain containing 4
0,90	KRT8	keratin 8, type II
0,90	DCT	dopachrome tautomerase
0,90	CCDC88A	coiled-coil domain containing 88A
0,90	BARHL1	BarH-like homeobox 1
0,90	TMEM262	transmembrane protein 262
0,90	FOXJ2	forkhead box J2
0,90	CLEC11A	C-type lectin domain family 11, member A
0,90	OR52N4	olfactory receptor, family 52, subfamily N, member 4 (gene/pseudogene)
0,90	LRP4	LDL receptor related protein 4
0,90	PRKY	protein kinase, Y-linked, pseudogene
0,90	ZNF546	zinc finger protein 546
0,90	ZNF474	zinc finger protein 474
0,90	DOCK4	dedicator of cytokinesis 4
0,90	MGST3	microsomal glutathione S-transferase 3
0,90	CHRNB4	cholinergic receptor, nicotinic beta 4
0,90	C6orf120	chromosome 6 open reading frame 120
0,90	HTR3B	5-hydroxytryptamine (serotonin) receptor 3B, ionotropic
0,90	RPL26L1	ribosomal protein L26-like 1
0,90	C1orf101	chromosome 1 open reading frame 101
0,90	MIB1	mindbomb E3 ubiquitin protein ligase 1
0,90	ZUFSP	zinc finger with UFM1-specific peptidase domain
0,90	TSSK2	testis-specific serine kinase 2
0,90	CACTIN-AS1	CACTIN antisense RNA 1
0,90	GPR155	G protein-coupled receptor 155
0,90	KIAA1328	KIAA1328
0,90	COA3	cytochrome c oxidase assembly factor 3
0,90	SAMHD1	SAM domain and HD domain 1
0,90	PNPLA8	patatin-like phospholipase domain containing 8
0,90	C11orf1	chromosome 11 open reading frame 1
0,90	FAM122A	family with sequence similarity 122A

0,90	MOV10	Mov10 RISC complex RNA helicase
0,90	KRTAP5-10	keratin associated protein 5-10
0,90	LPXN	leupaxin
0,90	GYPC	glycophorin C (Gerbich blood group)
0,90	ZNF655	zinc finger protein 655
0,90	METTL22	methyltransferase like 22
0,90	MYCBPAP	MYCBP associated protein
0,90	BAZ2B	bromodomain adjacent to zinc finger domain 2B
0,90	RPS6KA1	ribosomal protein S6 kinase, 90kDa, polypeptide 1
0,90	HMBS	hydroxymethylbilane synthase
0,90	GRM6	glutamate receptor, metabotropic 6
0,90	PGLYRP1	peptidoglycan recognition protein 1
0,90	OR4N4	olfactory receptor, family 4, subfamily N, member 4
0,90	PTDSS2	phosphatidylserine synthase 2
0,90	TPBGL	trophoblast glycoprotein-like
0,90	TMED9	transmembrane p24 trafficking protein 9
0,90	TAF6L	TAF6-like RNA polymerase II, p300/CBP-associated factor (PCAF)-associated factor, 65kDa
0,90	ZNF441	zinc finger protein 441
0,90	GNLY	granulysin
0,90	ITPKB	Transcript Identified by AceView, Entrez Gene ID(s) 3707
0,90	C5orf42	chromosome 5 open reading frame 42
0,90	TULP4	tubby like protein 4
0,90	BLOC1S5	biogenesis of lysosomal organelles complex-1, subunit 5, muted
0,90	ZNF334	zinc finger protein 334
0,90	NCF4	neutrophil cytosolic factor 4
0,90	NPIPA1	nuclear pore complex interacting protein family, member A1
0,90	IL20RB	interleukin 20 receptor beta
0,90	KRT37	keratin 37, type I
0,90	PRDM6	PR domain containing 6
0,90	SF3B5	splicing factor 3b subunit 5
0,90	RNF150	ring finger protein 150
0,90	CEP170	centrosomal protein 170kDa
0,90	TCTEX1D2	Tctex1 domain containing 2
0,90	DSPP	dentin sialophosphoprotein
0,90	NLRC5	NLR family, CARD domain containing 5
0,90	VRK2	vaccinia related kinase 2
0,90	MAMSTR	MEF2 activating motif and SAP domain containing transcriptional regulator
0,90	HEBP2	heme binding protein 2
0,90	KMT2E	lysine (K)-specific methyltransferase 2E
0,90	HOMER2	homer scaffolding protein 2
0,90	HCRTR1	hypocretin (orexin) receptor 1
0,90	COQ5	coenzyme Q5, methyltransferase
0,90	JMJD4	jumonji domain containing 4
0,90	CD27	CD27 molecule
0,90	ENKD1	enkurin domain containing 1

0,90	KLHL22	kelch-like family member 22
0,90	LOC102724238; LOC554249; RP11-111F5.4	Homo sapiens uncharacterized LOC102724238 (LOC102724238), long non-coding RNA.; Homo sapiens uncharacterized LOC554249 (LOC554249), long non-coding RNA.; Homo sapiens hypothetical LOC554249, mRNA (cDNA clone MGC:99799 IMAGE:6378300), complete cds.; novel transcript
0,90	OR10A7	olfactory receptor, family 10, subfamily A, member 7
0,90	PCBP3	poly(rC) binding protein 3
0,90	F12	coagulation factor XII (Hageman factor)
0,90	C9orf66	chromosome 9 open reading frame 66
0,90	C8orf44	chromosome 8 open reading frame 44
0,90	CBWD6; CBWD5; CBWD7	COBW domain containing 6; COBW domain containing 5; COBW domain containing 7
0,90	SHROOM3	shroom family member 3
0,90	USP54	ubiquitin specific peptidase 54
0,90	GLYATL1	glycine-N-acyltransferase-like 1
0,90	GZMK	granzyme K
0,90	SP5	Sp5 transcription factor
0,90	LCP2	lymphocyte cytosolic protein 2
0,90	OR10T2	olfactory receptor, family 10, subfamily T, member 2
0,90	LINC01561	long intergenic non-protein coding RNA 1561
0,90	MASTL	microtubule associated serine/threonine kinase-like
0,90	ADCY8	adenylate cyclase 8 (brain)
0,90	MTHFD1L	methylenetetrahydrofolate dehydrogenase (NADP+ dependent) 1-like
0,90	PGP	phosphoglycolate phosphatase
0,90	PRDX5	peroxiredoxin 5
0,90	PARD6A	par-6 family cell polarity regulator alpha
0,90	KRTAP5-2	keratin associated protein 5-2
0,90	FCGBP	Fc fragment of IgG binding protein
0,90	PRDM8	PR domain containing 8
0,90	KRT26	keratin 26, type I
0,90	CCDC81	coiled-coil domain containing 81
0,90	FKTN	fukutin
0,90	NFIX	nuclear factor I/X (CCAAT-binding transcription factor)
0,90	GPN3	GPN-loop GTPase 3
0,90	ICOS	inducible T-cell co-stimulator
0,90	GATA5	GATA binding protein 5
0,90	PTGER3	prostaglandin E receptor 3 (subtype EP3)
0,90	ZNF582	zinc finger protein 582
0,90	AMN1	antagonist of mitotic exit network 1 homolog
0,90	CNTNAP3P2; CNTNAP3	contactin associated protein-like 3 pseudogene 2; contactin associated protein-like 3
0,90	STK32B	serine/threonine kinase 32B
0,90	ZNF619	zinc finger protein 619
0,90	HOXB5	homeobox B5

0,90	UGT1A1; UGT1A6; UGT1A4; UGT1A10; UGT1A8; UGT1A7; UGT1A5; UGT1A3; UGT1A9	UDP glucuronosyltransferase 1 family, polypeptide A1; UDP glucuronosyltransferase 1 family, polypeptide A6; UDP glucuronosyltransferase 1 family, polypeptide A4; UDP glucuronosyltransferase 1 family, polypeptide A10; UDP glucuronosyltransferase 1 family, polypeptide A8; UDP glucuronosyltransferase 1 family, polypeptide A7; UDP glucuronosyltransferase 1 family, polypeptide A5; UDP glucuronosyltransferase 1 family, polypeptide A3; UDP glucuronosyltransferase 1 family, polypeptide A9
0,90	STON2	stonin 2
0,90	EXT1	exostosin glycosyltransferase 1
0,90	CENPJ	centromere protein J
0,90	PMVK	phosphomevalonate kinase
0,90	NFXL1	nuclear transcription factor, X-box binding-like 1
0,90	ERLIN2	ER lipid raft associated 2
0,90	RNASE13	ribonuclease, RNase A family, 13 (non-active)
0,90	AFF4	AF4/FMR2 family, member 4
0,90	KRTAP5-4	keratin associated protein 5-4
0,90	ENTPD8	ectonucleoside triphosphate diphosphohydrolase 8
0,90	SMCHD1	structural maintenance of chromosomes flexible hinge domain containing 1
0,90	TPRG1	tumor protein p63 regulated 1
0,90	PTH	parathyroid hormone
0,90	APBB1IP	amyloid beta (A4) precursor protein-binding, family B, member 1 interacting protein
0,90	PER3	period circadian clock 3
0,90	TDRD9	tudor domain containing 9
0,90	VPS4A	vacuolar protein sorting 4 homolog A (S. cerevisiae)
0,90	KIF14	kinesin family member 14
0,90	ARG2	arginase 2
0,90	TLN2	talin 2
0,90	SLC25A47	solute carrier family 25, member 47
0,90	NDST1	N-deacetylase/N-sulfotransferase (heparan glucosaminyl) 1
0,90	SLC17A4	solute carrier family 17, member 4
0,90	ROBO1	roundabout guidance receptor 1
0,90	DEFB114	defensin, beta 114
0,90	MFSD6	major facilitator superfamily domain containing 6
0,90	PTPN21	protein tyrosine phosphatase, non-receptor type 21
0,90	SLC25A35	solute carrier family 25, member 35
0,90	THYN1	thymocyte nuclear protein 1
0,90	HIST1H3A	histone cluster 1, H3a
0,90	SLC2A13	solute carrier family 2 (facilitated glucose transporter), member 13
0,90	ZFPL1	zinc finger protein-like 1
0,90	SGOL1	shugoshin-like 1 (S. pombe)
0,90	PARPBP	PARP1 binding protein
0,90	CNP	2,3-cyclic nucleotide 3 phosphodiesterase

0,90	RNF151	ring finger protein 151
0,90	ZGPAT; LIME1	zinc finger, CCCH-type with G-patch domain; Lck interacting transmembrane adaptor 1
0,90	FASN	fatty acid synthase
0,90	VWA3B	von Willebrand factor A domain containing 3B
0,90	KIF1C	kinesin family member 1C
0,90	NDUFA10	NADH dehydrogenase (ubiquinone) 1 alpha subcomplex, 10, 42kDa
0,90	MICAL3	microtubule associated monooxygenase, calponin and LIM domain containing 3
0,90	FLOT1	Transcript Identified by AceView, Entrez Gene ID(s) 10211
0,90	ATRNL1	attractin-like 1
0,90	ZNF274	zinc finger protein 274
0,90	STPG2	sperm-tail PG-rich repeat containing 2
0,90	IGIP	IgA-inducing protein
0,90	SEPHS1	selenophosphate synthetase 1
0,90	TPD52L1	tumor protein D52-like 1
0,90	TRAPPC13	trafficking protein particle complex 13
0,90	CDCA5	cell division cycle associated 5
0,90	GFAP	glial fibrillary acidic protein
0,90	ABCD1	ATP binding cassette subfamily D member 1
0,90	DYRK1B	dual specificity tyrosine-(Y)-phosphorylation regulated kinase 1B
0,90	ITPRIP	inositol 1,4,5-trisphosphate receptor interacting protein
0,90	RFPL3	ret finger protein-like 3
0,90	KLHL4	kelch-like family member 4
0,90	VIMP	VCP-interacting membrane selenoprotein
0,90	LINC01276	long intergenic non-protein coding RNA 1276
0,90	MEFV	Mediterranean fever
0,90	VPS26B	VPS26 retromer complex component B
0,90	CCDC82	coiled-coil domain containing 82
0,90	ZW10	zw10 kinetochore protein
0,90	HAND1	heart and neural crest derivatives expressed 1
0,90	FGF20	fibroblast growth factor 20
0,90	BMP8B	bone morphogenetic protein 8b
0,90	PIK3R2; IFI30	phosphoinositide-3-kinase, regulatory subunit 2 (beta); interferon, gamma-inducible protein 30
0,90	SHCBP1L	SHC SH2-domain binding protein 1-like
0,90	ACADVL	acyl-CoA dehydrogenase, very long chain
0,90	SKI	SKI proto-oncogene
0,90	P3H1	prolyl 3-hydroxylase 1
0,90	GHSR	growth hormone secretagogue receptor
0,90	MAP2K2	mitogen-activated protein kinase kinase 2
0,90	MARC1	mitochondrial amidoxime reducing component 1
0,90	CPT1B	carnitine palmitoyltransferase 1B (muscle)
0,90	HEBP1	heme binding protein 1
0,90	HCK	HCK proto-oncogene, Src family tyrosine kinase
0,90	ANKRD18B	ankyrin repeat domain 18B

0,90	TRIM6-TRIM34	TRIM6-TRIM34 readthrough
0,90	KDF1	keratinocyte differentiation factor 1
0,90	KRTAP2-1	keratin associated protein 2-1
0,90	CUBN	cubilin (intrinsic factor-cobalamin receptor)
0,90	SLC6A18	solute carrier family 6 (neutral amino acid transporter), member 18
0,90	UHRF1BP1	UHRF1 binding protein 1
0,90	APOBEC3B	apolipoprotein B mRNA editing enzyme, catalytic polypeptide-like 3B
0,90	TLR9	toll-like receptor 9
0,90	NOD1	nucleotide-binding oligomerization domain containing 1
0,90	ROCK2	Rho-associated, coiled-coil containing protein kinase 2
0,90	DENND5A	DENN/MADD domain containing 5A
0,90	RPS3A	Zhang2013 ALT_DONOR, coding, INTERNAL, intronic best transcript NM_001006
0,90	NBN	Transcript Identified by AceView, Entrez Gene ID(s) 4683
0,90	FAM208A	family with sequence similarity 208, member A
0,90	PARVB	parvin, beta
0,90	TMEM220	transmembrane protein 220
0,90	RERG	RAS-like, estrogen-regulated, growth inhibitor
0,90	ZCCHC14	zinc finger, CCHC domain containing 14
0,90	RIN2	Transcript Identified by AceView, Entrez Gene ID(s) 54453
0,90	HLA-L	major histocompatibility complex, class I, L (pseudogene)
0,90	PLCD3	phospholipase C, delta 3
0,90	DNAI2	dynein, axonemal, intermediate chain 2
0,90	FAM200A	family with sequence similarity 200, member A
0,90	AGL	amylo-alpha-1, 6-glucosidase, 4-alpha-glucanotransferase
0,90	WNT3	wingless-type MMTV integration site family, member 3
0,90	CUL4A	Memczak2013 ALT_ACCEPTOR, ALT_DONOR, coding, INTERNAL, intronic best transcript NM_001008895
0,90	CORO7; PAM16; CORO7-PAM16	coronin 7; presequence translocase-associated motor 16 homolog (S. cerevisiae); CORO7-PAM16 readthrough
0,90	MZT2B	mitotic spindle organizing protein 2B
0,90	NDUFA10	NADH dehydrogenase (ubiquinone) 1 alpha subcomplex, 10, 42kDa
0,90	HMBS	hydroxymethylbilane synthase
0,90	HR	hair growth associated
0,90	CMC1	C-x(9)-C motif containing 1
0,90	ARHGEF33	Rho guanine nucleotide exchange factor 33
0,90	HPSE2	heparanase 2 (inactive)
0,90	WWC3	WWC family member 3
0,90	SMC2	structural maintenance of chromosomes 2

0,90	MNS1	meiosis-specific nuclear structural 1
0,90	TUBGCP2	tubulin, gamma complex associated protein 2
0,90	ZSWIM7	zinc finger, SWIM-type containing 7
0,90	TCEB3CL2; TCEB3CL	transcription elongation factor B polypeptide 3C-like 2; transcription elongation factor B polypeptide 3C-like
0,90	PRKAR1A; ARSG	protein kinase, cAMP-dependent, regulatory, type I, alpha; arylsulfatase G
0,90	ZBP1	Z-DNA binding protein 1
0,90	TICAM1	toll-like receptor adaptor molecule 1
0,90	MUC20; SDHAP2; MIR570; LINC00969	mucin 20, cell surface associated; succinate dehydrogenase complex subunit A, flavoprotein pseudogene 2; microRNA 570; long intergenic non-protein coding RNA 969
0,90	FBXL19	F-box and leucine-rich repeat protein 19
0,90	OR2T5	olfactory receptor, family 2, subfamily T, member 5
0,90	WFDC9	WAP four-disulfide core domain 9
0,90	SAMD1	sterile alpha motif domain containing 1
0,90	VGLL4	vestigial-like family member 4
0,90	ABHD17C	abhydrolase domain containing 17C
0,90	NOTCH2NL	notch 2 N-terminal like
0,90	TP73	tumor protein p73
0,90	PLEKHA1	pleckstrin homology domain containing, family A (phosphoinositide binding specific) member 1
0,90	OR1E2	olfactory receptor, family 1, subfamily E, member 2
0,90	HLA-A	major histocompatibility complex, class I, A
0,90	DDIAS	DNA damage-induced apoptosis suppressor
0,90	WDR87	WD repeat domain 87
0,90	PSMC4	proteasome 26S subunit, ATPase 4 [Source:HGNC Symbol;Acc:HGNC:9551]
0,90	COL1A2	collagen, type I, alpha 2
0,90	TRAF7	TNF receptor-associated factor 7, E3 ubiquitin protein ligase
0,90	SH3D19	SH3 domain containing 19
0,90	CASC4	cancer susceptibility candidate 4
0,90	CLDN8	claudin 8
0,90	CFHR2	complement factor H-related 2
0,90	TIMM9	translocase of inner mitochondrial membrane 9 homolog (yeast)
0,90	ST8SIA6	ST8 alpha-N-acetyl-neuraminide alpha-2,8-sialyltransferase 6
0,90	GPR32	G protein-coupled receptor 32
0,90	HOXD8	homeobox D8
0,90	NEMP2	nuclear envelope integral membrane protein 2
0,90	NEIL2	nei-like DNA glycosylase 2
0,90	LOXL3	lysyl oxidase-like 3
0,90	UBE2J2	ubiquitin-conjugating enzyme E2, J2
0,90	CLDN18	claudin 18
0,90	FANCG	Fanconi anemia complementation group G
0,90	ICAM5	intercellular adhesion molecule 5

0,90	IFT43	intraflagellar transport 43
0,90	EPHX3	epoxide hydrolase 3
0,90	MIR17HG; MIR17; MIR18A; MIR19A; MIR19B1; MIR20A; MIR92A1	miR-17-92 cluster host gene; microRNA 17; microRNA 18a; microRNA 19a; microRNA 19b-1; microRNA 20a; microRNA 92a-1
0,90	TSHZ3	teashirt zinc finger homeobox 3
0,90	MMAB	methylmalonic aciduria (cobalamin deficiency) cblB type
0,90	BCL2L14	BCL2-like 14 (apoptosis facilitator)
0,90	C2orf68	chromosome 2 open reading frame 68
0,90	NOL4L	nucleolar protein 4-like
0,90	GOLGA8B	golgin A8 family, member B
0,90	WASF2	Memczak2013 ANTISENSE, coding, INTERNAL, intronic best transcript NM_006990
0,90	BLOC1S1-RDH5; RP11-644F5.10	BLOC1S1-RDH5 readthrough; Uncharacterized protein [Source:UniProtKB/TrEMBL;Acc:F8VNQ1]
0,90	MYOCD	myocardin
0,90	OR10G7	olfactory receptor, family 10, subfamily G, member 7
0,90	FBXL17	F-box and leucine-rich repeat protein 17
0,90	CCDC24	coiled-coil domain containing 24
0,90	SYT12	synaptotagmin XII
0,90	CYP2C9	cytochrome P450, family 2, subfamily C, polypeptide 9
0,90	CAMK1D	calcium/calmodulin-dependent protein kinase ID
0,90	PSME2; MIR7703	proteasome activator subunit 2; microRNA 7703
0,90	NHLRC4; PIGQ	NHL repeat containing 4; phosphatidylinositol glycan anchor biosynthesis class Q
0,90	THNSL2	threonine synthase-like 2
0,90	TBC1D2	TBC1 domain family, member 2
0,90	HIST1H1D	histone cluster 1, H1d
0,90	PTPN22	protein tyrosine phosphatase, non-receptor type 22 (lymphoid)
0,90	VPS39	vacuolar protein sorting 39 homolog (S. cerevisiae)
0,90	ETFB	electron-transfer-flavoprotein, beta polypeptide
0,90	MYH15	myosin, heavy chain 15
0,90	OR8G2	olfactory receptor, family 8, subfamily G, member 2
0,90	UBXN2A	UBX domain protein 2A
0,90	LOC100129520	testis expressed sequence 13-like

0,90	CD99	Homo sapiens CD99 molecule (CD99), transcript variant 2, mRNA.; Homo sapiens CD99 molecule (CD99), transcript variant 3, mRNA.; Homo sapiens CD99 molecule (CD99), transcript variant 1, mRNA.; CD99 molecule [Source:HGNC Symbol;Acc:HGNC:7082]; Homo sapiens CD99 molecule, mRNA (cDNA clone MGC:2282 IMAGE:3161673), complete cds.; Homo sapiens CD99 molecule, mRNA (cDNA clone MGC:3938 IMAGE:2906143), complete cds.; Homo sapiens CD99 molecule, mRNA (cDNA clone MGC:19734 IMAGE:3606974), complete cds.; Homo sapiens CD99 molecule, mRNA (cDNA clone MGC:4214 IMAGE:2957883), complete cds.; Homo sapiens CD99 molecule, mRNA (cDNA clone MGC:14521 IMAGE:4094268), complete cds.; Salzman2013 ANNOTATED, CDS, coding, INTERNAL, OVCODE, OVEXON best transcript NM_002414; Salzman2013 ANNOTATED, CDS, coding, OVCODE, OVEXON, UTR3 best transcript NM_002414
0,90	NFAT5	nuclear factor of activated T-cells 5, tonicity-responsive
0,90	LOXL1	lysyl oxidase-like 1
0,90	NTSR1	neurotensin receptor 1 (high affinity)
0,90	LOC100652768; PCSK7	uncharacterized LOC100652768; Salzman2013 ANNOTATED, ncRNA, OVCODE, OVERLAPTX, OVEXON best transcript NR_045215; Transcript Identified by AceView, Entrez Gene ID(s) 9159
0,90	TMEM132D	transmembrane protein 132D
0,90	ABHD17A	abhydrolase domain containing 17A
0,90	LRP2	LDL receptor related protein 2
0,90	NPIPB6	nuclear pore complex interacting protein family, member B6
0,90	NRG3	neuregulin 3
0,90	CEACAM4	carcinoembryonic antigen-related cell adhesion molecule 4
0,90	TMEM245	transmembrane protein 245
0,90	GALNT11	polypeptide N-acetylgalactosaminyltransferase 11
0,90	MUC19	mucin 19, oligomeric
0,90	PPP1R16A	protein phosphatase 1, regulatory subunit 16A
0,90	DUT	deoxyuridine triphosphatase
0,90	ABHD4	abhydrolase domain containing 4
0,90	FARP1; MIR3170	FERM, ARH/RhoGEF and pleckstrin domain protein 1; microRNA 3170
0,90	PLEKHM2	pleckstrin homology domain containing, family M (with RUN domain) member 2
0,90	NKAPL	NFKB activating protein-like
0,90	RBMS1; MIR4785	RNA binding motif, single stranded interacting protein 1; microRNA 4785
0,90	TMEM203	transmembrane protein 203

0,90	TMEM107; SNORD118	transmembrane protein 107; small nucleolar RNA, C/D box 118
0,90	MED12	mediator complex subunit 12
0,90	OR4X1	olfactory receptor, family 4, subfamily X, member 1 (gene/pseudogene)
0,90	CNN1	calponin 1, basic, smooth muscle
0,90	RAB26	RAB26, member RAS oncogene family
0,90	KHDRBS3	KH domain containing, RNA binding, signal transduction associated 3
0,90	PDZK1	PDZ domain containing 1
0,90	TTC39A	tetratricopeptide repeat domain 39A
0,90	PDCD5	programmed cell death 5
0,90	AASS	aminoadipate-semialdehyde synthase
0,90	WISP2	WNT1 inducible signaling pathway protein 2
0,90	LZTR1	leucine-zipper-like transcription regulator 1
0,90	SLPI	secretory leukocyte peptidase inhibitor
0,90	NT5DC4	5-nucleotidase domain containing 4
0,90	LMO1	LIM domain only 1 (rhombotin 1)
0,90	ZNF2	zinc finger protein 2
0,90	ago-02	argonaute RISC catalytic component 2
0,90	TSC2	tuberous sclerosis 2
0,90	PRR14L	proline rich 14-like
0,90	CADM4	cell adhesion molecule 4
0,90	MYO1H	myosin IH
0,90	LRRC8B	leucine rich repeat containing 8 family, member B
0,90	SAMD14	sterile alpha motif domain containing 14
0,90	HIST1H3C	histone cluster 1, H3c
0,90	SLITRK1	SLIT and NTRK-like family, member 1
0,90	TRHDE-AS1	TRHDE antisense RNA 1
0,90	SH3BP5	SH3-domain binding protein 5 (BTK-associated)
0,90	AKAP7	A kinase (PRKA) anchor protein 7
0,90	CD72	CD72 molecule
0,90	MROH9	maestro heat-like repeat family member 9
0,90	NEK2	NIMA-related kinase 2
0,90	KIF15	kinesin family member 15
0,90	KLHDC3	kelch domain containing 3
0,90	DNAJC6	DnaJ (Hsp40) homolog, subfamily C, member 6
0,90	PAIP2B	poly(A) binding protein interacting protein 2B
0,90	PALM2	paralemmin 2
0,90	RELL1	RELT-like 1
0,90	DCAF7	DDB1 and CUL4 associated factor 7
0,90	NECAB2	N-terminal EF-hand calcium binding protein 2
0,90	C22orf15	chromosome 22 open reading frame 15
0,90	ASNA1	arsA arsenite transporter, ATP-binding, homolog 1 (bacterial)
0,90	AXIN1	Memczak2013 ALT_ACCEPTOR, ALT_DONOR, coding, INTERNAL, intronic best transcript NM_003502
0,90	PGA3; PGA4	pepsinogen 3, group I (pepsinogen A); pepsinogen 4, group I (pepsinogen A)

0,90	CLUH	clustered mitochondria (cluA/CLU1) homolog
0,90	ADGRF1	adhesion G protein-coupled receptor F1
0,90	PIGR	polymeric immunoglobulin receptor
0,90	CACNB1	calcium channel, voltage-dependent, beta 1 subunit
0,90	BRWD3	bromodomain and WD repeat domain containing 3
0,90	SPRR2F	small proline-rich protein 2F
0,90	SHTN1	shootin 1
0,90	TRIM15	tripartite motif containing 15
0,90	KLHDC10	kelch domain containing 10
0,90	OR5T1	olfactory receptor, family 5, subfamily T, member 1
0,90	THAP11	THAP domain containing 11
0,90	DNM3	dynamin 3
0,89	RBM7	RNA binding motif protein 7
0,89	FAM180A	family with sequence similarity 180, member A
0,89	LY6H	lymphocyte antigen 6 complex, locus H
0,89	ITCH	itchy E3 ubiquitin protein ligase
0,89	DSC2	desmocollin 2
0,89	OR6C1	olfactory receptor, family 6, subfamily C, member 1
0,89	TRIM42	tripartite motif containing 42
0,89	TMIE	transmembrane inner ear
0,89	CNST	consortin, connexin sorting protein
0,89	FAM217A	family with sequence similarity 217, member A
0,89	RILP	Rab interacting lysosomal protein
0,89	MOAP1	modulator of apoptosis 1
0,89	PCDH15	protocadherin-related 15
0,89	STMN3	stathmin-like 3
0,89	DCSTAMP	dendrocyte expressed seven transmembrane protein
0,89	TCEB3CL; TCEB3C	transcription elongation factor B polypeptide 3C-like; transcription elongation factor B polypeptide 3C (elongin A3)
0,89	HACE1	HECT domain and ankyrin repeat containing E3 ubiquitin protein ligase 1
0,89	KIAA0586	KIAA0586
0,89	SNTA1	syntrophin, alpha 1
0,89	NELFCD	negative elongation factor complex member C/D
0,89	VPS11	VPS11, CORVET/HOPS core subunit [Source:HGNC Symbol;Acc:HGNC:14583]
0,89	ZNF500	zinc finger protein 500
0,89	DES	desmin
0,89	NEFM	neurofilament, medium polypeptide
0,89	PLA2G7	phospholipase A2, group VII (platelet-activating factor acetylhydrolase, plasma)
0,89	C1orf21	chromosome 1 open reading frame 21
0,89	USH1C	Usher syndrome 1C
0,89	OR51D1	olfactory receptor, family 51, subfamily D, member 1
0,89	ZFYVE19	zinc finger, FYVE domain containing 19
0,89	SPACA7	sperm acrosome associated 7
0,89	UNC5B	unc-5 netrin receptor B
0,89	VEGFA	vascular endothelial growth factor A
0,89	TMEM18	transmembrane protein 18

0,89	APCS	amyloid P component, serum
0,89	PDE1C	phosphodiesterase 1C, calmodulin-dependent 70kDa
0,89	SLBP	stem-loop binding protein
0,89	FICD	FIC domain containing
0,89	SPATA24	spermatogenesis associated 24
0,89	FDXR	ferredoxin reductase
0,89	CPLX4	complexin 4
0,89	MAP4K4	mitogen-activated protein kinase kinase kinase kinase 4
0,89	TBC1D10B	TBC1 domain family, member 10B
0,89	SLC37A4	solute carrier family 37 (glucose-6-phosphate transporter), member 4 [Source:HGNC Symbol;Acc:HGNC:4061]
0,89	ZSCAN1	zinc finger and SCAN domain containing 1
0,89	LRRC4	leucine rich repeat containing 4
0,89	UGT3A1	UDP glycosyltransferase 3 family, polypeptide A1
0,89	TNFRSF4	tumor necrosis factor receptor superfamily, member 4
0,89	CHMP4C	charged multivesicular body protein 4C
0,89	RHBDD3	rhomboid domain containing 3
0,89	SIK1	salt-inducible kinase 1
0,89	ST6GALNAC1	ST6 (alpha-N-acetyl-neuraminyl-2,3-beta-galactosyl-1,3)-N-acetylgalactosaminide alpha-2,6-sialyltransferase 1
0,89	GIGYF1	GRB10 interacting GYF protein 1
0,89	FAM111A	family with sequence similarity 111, member A
0,89	CCDC54	coiled-coil domain containing 54
0,89	RPS9	ribosomal protein S9
0,89	TTC33	tetratricopeptide repeat domain 33
0,89	FUT4	fucosyltransferase 4 (alpha (1,3) fucosyltransferase, myeloid-specific)
0,89	ITK	IL2-inducible T-cell kinase
0,89	ADAMTS4	ADAM metallopeptidase with thrombospondin type 1 motif 4
0,89	TRIM5	tripartite motif containing 5
0,89	PLEKHO2	pleckstrin homology domain containing, family O member 2
0,89	CCND3	cyclin D3
0,89	NOP9	NOP9 nucleolar protein
0,89	IRGM	immunity-related GTPase family, M
0,89	ZC3H12C	zinc finger CCCH-type containing 12C
0,89	ACER1	alkaline ceramidase 1
0,89	ZNF506	Transcript Identified by AceView, Entrez Gene ID(s) 284440; 440515
0,89	CRY2	Memczak2013 ANTISENSE, CDS, coding, INTERNAL, UTR3 best transcript NM_001127457
0,89	ANKRD24	ankyrin repeat domain 24
0,89	GALNT2	polypeptide N-acetylgalactosaminyltransferase 2
0,89	CINP	cyclin-dependent kinase 2 interacting protein
0,89	ACAP1	ArfGAP with coiled-coil, ankyrin repeat and PH domains 1

0,89	ZIC4	Zic family member 4
0,89	MS4A18	membrane-spanning 4-domains, subfamily A, member 18
0,89	IL1R1	interleukin 1 receptor, type I
0,89	RIPPLY1	ripply transcriptional repressor 1
0,89	USP20	ubiquitin specific peptidase 20
0,89	DLG5	discs, large homolog 5 (Drosophila)
0,89	STMN1; MIR3917	stathmin 1; microRNA 3917
0,89	ZNF780A	zinc finger protein 780A
0,89	ITFG1	integrin alpha FG-GAP repeat containing 1
0,89	CDK12	cyclin-dependent kinase 12
0,89	XPO4	exportin 4
0,89	DPH7	diphthamide biosynthesis 7
0,89	PPP2R5B	protein phosphatase 2, regulatory subunit B, beta
0,89	DEFA4	defensin, alpha 4, corticostatin
0,89	NPL	N-acetylneuraminate pyruvate lyase (dihydrodipicolinate synthase)
0,89	C8orf76	chromosome 8 open reading frame 76
0,89	CLEC12B	C-type lectin domain family 12, member B
0,89	PGAP1	post-GPI attachment to proteins 1
0,89	FRMD4B	FERM domain containing 4B
0,89	PBX3	pre-B-cell leukemia homeobox 3
0,89	HEY2	hes-related family bHLH transcription factor with YRPW motif 2
0,89	TMOD1	tropomodulin 1
0,89	HTR3E	5-hydroxytryptamine (serotonin) receptor 3E, ionotropic
0,89	KIAA1161	KIAA1161
0,89	RPS6KA1	ribosomal protein S6 kinase, 90kDa, polypeptide 1
0,89	DFFB	DNA fragmentation factor, 40kDa, beta polypeptide (caspase-activated DNase)
0,89	APOL5	apolipoprotein L, 5
0,89	TBCC	tubulin folding cofactor C
0,89	SCUBE1	signal peptide, CUB domain, EGF-like 1
0,89	WBSCR17	Memczak2013 ALT_ACCEPTOR, ALT_DONOR, coding, INTERNAL, intronic best transcript NM_022479
0,89	LY96	lymphocyte antigen 96
0,89	PAX6	paired box 6
0,89	ZSCAN20	zinc finger and SCAN domain containing 20
0,89	MRPL57	mitochondrial ribosomal protein L57
0,89	LDOC1L	leucine zipper, down-regulated in cancer 1-like
0,89	MAGEB2	MAGE family member B2
0,89	SOBP	sine oculis binding protein homolog
0,89	FNDC3A	fibronectin type III domain containing 3A
0,89	BLID; MIR100HG	BH3-like motif containing, cell death inducer; mir-100-let-7a-2 cluster host gene
0,89	OXLD1	oxidoreductase-like domain containing 1
0,89	NLRP1	NLR family, pyrin domain containing 1

0,89	ETV2	ets variant 2
0,89	HHAT	hedgehog acyltransferase
0,89	GCNT1	glucosaminyl (N-acetyl) transferase 1, core 2
0,89	ITGB8	integrin beta 8
0,89	AUTS2	autism susceptibility candidate 2
0,89	MTRR	5-methyltetrahydrofolate-homocysteine methyltransferase reductase
0,89	GOLGA8R	golgin A8 family, member R
0,89	DNASE1L1	deoxyribonuclease I-like 1
0,89	HRH2	histamine receptor H2
0,89	LTBP3	latent transforming growth factor beta binding protein 3
0,89	KLHDC9	kelch domain containing 9
0,89	DNAJC17	DnaJ (Hsp40) homolog, subfamily C, member 17
0,89	TRPM8	transient receptor potential cation channel, subfamily M, member 8
0,89	HSD17B1	hydroxysteroid (17-beta) dehydrogenase 1
0,89	EVI5	ecotropic viral integration site 5
0,89	DOCK11	dedicator of cytokinesis 11
0,89	PXDN	peroxidasin
0,89	GOLGA6L4	Homo sapiens golgin A6 family-like 4 (GOLGA6L4), mRNA.; golgin A6 family-like 4; golgin A6 family-like 4 [Source:HGNC Symbol;Acc:HGNC:27256]
0,89	SLC29A3	Transcript Identified by AceView, Entrez Gene ID(s) 55315
0,89	ZNF443	zinc finger protein 443
0,89	RNF128	ring finger protein 128, E3 ubiquitin protein ligase
0,89	EFCAB8	EF-hand calcium binding domain 8
0,89	DDX59	DEAD (Asp-Glu-Ala-Asp) box polypeptide 59
0,89	UBN1	ubinuclein 1
0,89	THSD7B	thrombospondin type 1 domain containing 7B
0,89	IQCA1L	IQ motif containing with AAA domain 1 like
0,89	HECA	hdc homolog, cell cycle regulator
0,89	FAM63A	family with sequence similarity 63, member A
0,89	MYH8	myosin, heavy chain 8, skeletal muscle, perinatal
0,89	NCR3	natural cytotoxicity triggering receptor 3
0,89	PITPNM2	phosphatidylinositol transfer protein, membrane-associated 2
0,89	HUNK	hormonally up-regulated Neu-associated kinase
0,89	LRRC36	leucine rich repeat containing 36
0,89	PDE3B	phosphodiesterase 3B, cGMP-inhibited
0,89	SUSD4	sushi domain containing 4
0,89	ZNF688	zinc finger protein 688
0,89	FGF1	fibroblast growth factor 1 (acidic)
0,89	LARS2	leucyl-tRNA synthetase 2, mitochondrial
0,89	RIT1	Ras-like without CAAX 1
0,89	LRFN1	leucine rich repeat and fibronectin type III domain containing 1
0,89	YBEY	ybeY metallopeptidase (putative)
0,89	ZHX1	zinc fingers and homeoboxes 1

0,89	ZNF181	zinc finger protein 181
0,89	RGS2	regulator of G-protein signaling 2
0,89	LPAR1	Jeck2013 ALT_ACCEPTOR, ALT_DONOR, coding, INTERNAL, intronic best transcript NM_057159; Salzman2013 ALT_ACCEPTOR, ALT_DONOR, coding, INTERNAL, intronic best transcript NM_057159
0,89	ARMC6	armadillo repeat containing 6
0,89	TP53TG3D; LOC102723655; TP53TG3; AC136612.1	Homo sapiens TP53 target 3D (TP53TG3D), transcript variant 2, non-coding RNA.; TP53-target gene 3 protein; Homo sapiens TP53 target 3, mRNA (cDNA clone MGC:119889 IMAGE:40015196), complete cds.; Homo sapiens TP53 target 3, mRNA (cDNA clone MGC:119888 IMAGE:40015195), complete cds.
0,89	TPSD1	tryptase delta 1
0,89	KCNJ16	potassium channel, inwardly rectifying subfamily J, member 16
0,89	CORO2B	coronin, actin binding protein, 2B
0,89	OR2Y1	olfactory receptor, family 2, subfamily Y, member 1
0,89	SOGA3; KIAA0408	SOGA family member 3; KIAA0408
0,89	TTPA	tocopherol (alpha) transfer protein
0,89	CABP7	calcium binding protein 7
0,89	SLC5A8	solute carrier family 5 (sodium/monocarboxylate cotransporter), member 8
0,89	IRF3	interferon regulatory factor 3
0,89	GLTSCR2; SNORD23	glioma tumor suppressor candidate region gene 2; small nucleolar RNA, C/D box 23
0,89	TARS2; MIR6878	threonyl-tRNA synthetase 2, mitochondrial (putative); microRNA 6878
0,89	YBX1	Zhang2013 ALT_ACCEPTOR, ALT_DONOR, coding, INTERNAL, intronic best transcript NM_004559
0,89	TRABD2B	TraB domain containing 2B
0,89	TCF20	transcription factor 20 (AR1)
0,89	TCF20	transcription factor 20 (AR1)
0,89	C9orf47; S1PR3	chromosome 9 open reading frame 47; sphingosine-1-phosphate receptor 3
0,89	ITGA5	integrin alpha 5
0,89	LRIG2	leucine-rich repeats and immunoglobulin-like domains 2
0,89	ZNF461	zinc finger protein 461
0,89	PENK	proenkephalin
0,89	COL6A5	collagen, type VI, alpha 5
0,89	UIMC1	ubiquitin interaction motif containing 1
0,89	JDP2	Jun dimerization protein 2
0,89	PRC1-AS1	PRC1 antisense RNA 1
0,89	C3orf30	chromosome 3 open reading frame 30
0,89	FANCL	Fanconi anemia complementation group L
0,89	ESR1	estrogen receptor 1

0,89	ADRBK2	adrenergic, beta, receptor kinase 2
0,89	SPAG4	sperm associated antigen 4
0,89	CHKB-CPT1B	CHKB-CPT1B readthrough (NMD candidate)
0,89	ARFGEF2	ADP-ribosylation factor guanine nucleotide-exchange factor 2 (brefeldin A-inhibited)
0,89	ZC3H12A; MIR6732	zinc finger CCCH-type containing 12A; microRNA 6732
0,89	SHISA8	shisa family member 8
0,89	RGS1	regulator of G-protein signaling 1
0,89	GCOM1; MYZAP; POLR2M	GRINL1A complex locus 1; myocardial zonula adherens protein; polymerase (RNA) II (DNA directed) polypeptide M
0,89	STK36	serine/threonine kinase 36
0,89	LRRC72	leucine rich repeat containing 72
0,89	THAP7-AS1	THAP7 antisense RNA 1
0,89	SGTB	small glutamine-rich tetratricopeptide repeat (TPR)-containing, beta
0,89	IFT46	intraflagellar transport 46
0,89	ZNF821	zinc finger protein 821
0,89	SEMA6A	sema domain, transmembrane domain (TM), and cytoplasmic domain, (semaphorin) 6A
0,89	ASIC4	acid sensing ion channel family member 4
0,89	UCN	urocortin
0,89	ZNF852	zinc finger protein 852
0,89	KLK8	kallikrein related peptidase 8
0,89	SLC16A6	solute carrier family 16, member 6
0,89	LYG2	lysozyme G-like 2
0,89	STRN4	striatin, calmodulin binding protein 4
0,89	SLC35E3	solute carrier family 35, member E3
0,89	TWF2	twinfilin actin binding protein 2
0,89	CLCN6	chloride channel, voltage-sensitive 6
0,89	PRAMEF27	PRAME family member 27
0,89	SLC34A2	solute carrier family 34 (type II sodium/phosphate cotransporter), member 2
0,89	MARCH8	membrane associated ring finger 8
0,89	OPA3	optic atrophy 3 (autosomal recessive, with chorea and spastic paraplegia)
0,89	PLXNA3	plexin A3
0,89	NES	nestin
0,89	KRTAP1-5	keratin associated protein 1-5
0,89	TLE6	transducin-like enhancer of split 6
0,89	RRP9	ribosomal RNA processing 9, small subunit (SSU) processome component, homolog (yeast)
0,89	WDR73	WD repeat domain 73
0,89	ASF1B	anti-silencing function 1B histone chaperone
0,89	INSL4	insulin-like 4 (placenta)
0,89	GPR52	G protein-coupled receptor 52
0,89	PIGN	phosphatidylinositol glycan anchor biosynthesis class N
0,89	EEF2K	eukaryotic elongation factor 2 kinase

0,89	ATP7A	ATPase, Cu++ transporting, alpha polypeptide
0,89	VSTM2A; VSTM2A-OT1	V-set and transmembrane domain containing 2A; VSTM2A overlapping transcript 1
0,89	NAPB	N-ethylmaleimide-sensitive factor attachment protein, beta
0,89	CD99	CD99 molecule
0,89	KLHDC4	kelch domain containing 4
0,89	CFAP44	cilia and flagella associated protein 44
0,89	OSBPL7	oxysterol binding protein-like 7
0,89	TXNDC16	thioredoxin domain containing 16
0,89	ADAM22	ADAM metallopeptidase domain 22
0,89	UBE2J1	ubiquitin-conjugating enzyme E2, J1
0,89	TSTD1	thiosulfate sulfurtransferase (rhodanese)-like domain containing 1
0,89	ARHGAP30	Rho GTPase activating protein 30
0,89	GVQW2	GVQW motif containing 2
0,89	EIF2AK4	eukaryotic translation initiation factor 2 alpha kinase 4
0,89	CRNKL1	crooked neck pre-mRNA splicing factor 1
0,89	SYNE3	spectrin repeat containing, nuclear envelope family member 3
0,89	E4F1	E4F transcription factor 1
0,89	SDHAF3	succinate dehydrogenase complex assembly factor 3
0,89	ADAMTS6	ADAM metallopeptidase with thrombospondin type 1 motif 6
0,89	ZNF544	zinc finger protein 544
0,89	DGKG	diacylglycerol kinase gamma
0,89	ANKRD60	ankyrin repeat domain 60
0,89	MFAP3	microfibrillar associated protein 3
0,89	CBY1	chibby homolog 1 (Drosophila)
0,89	ZBED1; DHRSX	zinc finger, BED-type containing 1; dehydrogenase/reductase (SDR family) X-linked
0,89	MTOR	mechanistic target of rapamycin (serine/threonine kinase)
0,89	SAMD7	sterile alpha motif domain containing 7
0,89	DUSP28	dual specificity phosphatase 28
0,89	ERGIC1	Memczak2013 ALT_ACCEPTOR, ALT_DONOR, coding, INTERNAL, intronic best transcript NM_001031711
0,89	ANXA6	annexin A6
0,89	PTPN5	protein tyrosine phosphatase, non-receptor type 5 (striatum-enriched)
0,89	UBE2T	ubiquitin conjugating enzyme E2T
0,89	SUMF2	sulfatase modifying factor 2
0,89	GSAP	gamma-secretase activating protein
0,89	ELF1	Memczak2013 ALT_ACCEPTOR, ALT_DONOR, coding, INTERNAL, intronic best transcript NM_172373
0,89	ARHGEF2	Rho/Rac guanine nucleotide exchange factor 2
0,89	GOLGA2P5	golgin A2 pseudogene 5
0,89	DNAH12	dynein, axonemal, heavy chain 12

0,89	HEY1	hes-related family bHLH transcription factor with YRPW motif 1
0,89	CKAP2L	cytoskeleton associated protein 2-like
0,89	IL12RB2	interleukin 12 receptor, beta 2
0,89	KLC4	kinesin light chain 4
0,89	NOL6	Memczak2013 ANTISENSE, CDS, coding, INTERNAL, intronic best transcript NM_139235
0,89	RWDD2A	RWD domain containing 2A
0,89	TMEM155	transmembrane protein 155
0,89	KIAA0319	KIAA0319
0,89	NCOA5	nuclear receptor coactivator 5
0,89	TPRN	taperin
0,89	C11orf85	chromosome 11 open reading frame 85
0,89	PADI4	peptidyl arginine deiminase, type IV
0,89	MFSD12	major facilitator superfamily domain containing 12
0,89	CR1	complement component (3b/4b) receptor 1 (Knops blood group)
0,89	ZBTB18	zinc finger and BTB domain containing 18
0,89	PAK2	p21 protein (Cdc42/Rac)-activated kinase 2
0,89	PSMB8-AS1	PSMB8 antisense RNA 1 (head to head)
0,89	LINS1	lines homolog 1
0,89	NPHP4	nephronophthisis 4
0,89	PEX11G	peroxisomal biogenesis factor 11 gamma
0,89	C11orf53	chromosome 11 open reading frame 53
0,89	F10	coagulation factor X
0,89	TESPA1	thymocyte expressed, positive selection associated 1
0,89	FOXO3	forkhead box O3
0,89	APEX2	APEX nuclease (apurinic/apyrimidinic endonuclease) 2
0,89	SMYD1	SET and MYND domain containing 1
0,89	OBSL1	obscurin-like 1
0,89	IDH3B	isocitrate dehydrogenase 3 (NAD+) beta
0,89	CYP3A43	cytochrome P450, family 3, subfamily A, polypeptide 43
0,89	L1CAM	L1 cell adhesion molecule
0,89	SIRT1	sirtuin 1
0,89	CXCR2	chemokine (C-X-C motif) receptor 2
0,89	CCDC144A	coiled-coil domain containing 144A
0,89	SPTY2D1	SPT2 chromatin protein domain containing 1
0,89	VN1R10P	vomeronasal 1 receptor 10 pseudogene
0,89	ADCK3	aarF domain containing kinase 3
0,89	ARSA	arylsulfatase A
0,89	HAPLN3	hyaluronan and proteoglycan link protein 3
0,89	DGKI	diacylglycerol kinase, iota
0,89	GRASP	GRP1 (general receptor for phosphoinositides 1)-associated scaffold protein
0,89	SLC26A3	solute carrier family 26 (anion exchanger), member 3
0,89	ANKRD53	ankyrin repeat domain 53
0,89	TYK2	tyrosine kinase 2
0,89	MAP1A	microtubule associated protein 1A

0,89	DCDC2	doublecortin domain containing 2
0,89	SLC35E2B	solute carrier family 35, member E2B
0,89	RGS6	regulator of G-protein signaling 6
0,89	CALCOCO2	calcium binding and coiled-coil domain 2
0,89	SMG8	SMG8 nonsense mediated mRNA decay factor
0,89	TAF3	TATA box binding protein associated factor 3
0,89	CNIH4	cornichon family AMPA receptor auxiliary protein 4
0,89	CEP295NL; TIMP2	CEP295 N-terminal like; TIMP metallopeptidase inhibitor 2
0,89	MST1R	macrophage stimulating 1 receptor
0,89	MEDAG	mesenteric estrogen-dependent adipogenesis
0,89	CITED1	Cbp/p300-interacting transactivator, with Glu/Asp rich carboxy-terminal domain, 1
0,89	TAF4B	TAF4b RNA polymerase II, TATA box binding protein (TBP)-associated factor, 105kDa
0,89	WNT9A	wingless-type MMTV integration site family, member 9A
0,89	E2F7	E2F transcription factor 7
0,89	DOCK5	dedicator of cytokinesis 5
0,89	SYNC	syncoilin, intermediate filament protein
0,89	RNASE2	ribonuclease, RNase A family, 2 (liver, eosinophil-derived neurotoxin)
0,89	CHRNA9	cholinergic receptor, nicotinic alpha 9
0,89	UXT-AS1	UXT antisense RNA 1
0,89	APOBEC3F	apolipoprotein B mRNA editing enzyme, catalytic polypeptide-like 3F
0,89	FRAS1	Fraser extracellular matrix complex subunit 1
0,89	GKN2	gastrokine 2
0,89	SURF1	surfeit 1
0,89	PEX12	peroxisomal biogenesis factor 12
0,89	SUSD2	sushi domain containing 2
0,89	CCKBR	cholecystokinin B receptor
0,89	ZNF783	zinc finger family member 783
0,89	MNAT1	MNAT CDK-activating kinase assembly factor 1
0,89	CYLC1	cylicin, basic protein of sperm head cytoskeleton 1
0,89	VN1R4	vomeronasal 1 receptor 4
0,89	PSMB10	proteasome subunit beta 10
0,89	SLC24A5	solute carrier family 24 (sodium/potassium/calcium exchanger), member 5
0,89	CFHR3	complement factor H-related 3
0,89	STK17A	serine/threonine kinase 17a
0,89	NMU	neuromedin U
0,89	FUNDC2	FUN14 domain containing 2
0,89	ZDBF2	zinc finger, DBF-type containing 2
0,89	CARD18	caspase recruitment domain family, member 18
0,89	ADAD1	adenosine deaminase domain containing 1
0,89	PCYOX1L	prenylcysteine oxidase 1 like
0,89	PDZD11	PDZ domain containing 11
0,89	GPR179	G protein-coupled receptor 179
0,89	ACOT11	acyl-CoA thioesterase 11

0,89	USP9X	ubiquitin specific peptidase 9, X-linked
0,89	ARHGAP27	Rho GTPase activating protein 27
0,89	HNRNPA1P33; LINC00842; ANXA8L1	heterogeneous nuclear ribonucleoprotein A1 pseudogene 33; long intergenic non-protein coding RNA 842; annexin A8-like 1
0,89	ZNF512B	zinc finger protein 512B
0,89	KLHL13	kelch-like family member 13
0,89	NAA20	N(alpha)-acetyltransferase 20, NatB catalytic subunit
0,89	SH3TC2	SH3 domain and tetratricopeptide repeats 2
0,89	TSSK4	testis-specific serine kinase 4
0,89	CEP72	centrosomal protein 72kDa
0,89	PLPPR5	phospholipid phosphatase related 5
0,89	MRGPRX3	MAS-related GPR, member X3
0,89	CTSZ	cathepsin Z
0,89	RINL	Ras and Rab interactor like
0,89	PEG3; ZIM2	paternally expressed 3; zinc finger, imprinted 2
0,89	IL17RD	interleukin 17 receptor D
0,89	FRAT1	frequently rearranged in advanced T-cell lymphomas 1
0,89	HYOU1	hypoxia up-regulated 1
0,89	CNTFR	ciliary neurotrophic factor receptor
0,89	HECW2	HECT, C2 and WW domain containing E3 ubiquitin protein ligase 2
0,89	STAT4	signal transducer and activator of transcription 4
0,89	BTAF1	BTAF1 RNA polymerase II, B-TFIID transcription factor-associated, 170kDa
0,89	CHST14	carbohydrate (N-acetylgalactosamine 4-0) sulfotransferase 14
0,89	WRNIP1	Werner helicase interacting protein 1
0,89	FLNB	filamin B, beta
0,89	SDR42E2	short chain dehydrogenase/reductase family 42E, member 2
0,89	SREBF2	sterol regulatory element binding transcription factor 2
0,89	NEDD9	neural precursor cell expressed, developmentally down-regulated 9
0,89	PKMYT1	protein kinase, membrane associated tyrosine/threonine 1
0,89	ORM1	orosomucoid 1
0,89	NAALADL2	N-acetylated alpha-linked acidic dipeptidase-like 2
0,89	SOCS2	suppressor of cytokine signaling 2
0,89	CADPS2	Ca++-dependent secretion activator 2
0,89	ARPIN	actin-related protein 2/3 complex inhibitor
0,89	KCNK17	potassium channel, two pore domain subfamily K, member 17
0,89	IGFBP7	insulin like growth factor binding protein 7
0,89	CNOT1; SNORA50A	CCR4-NOT transcription complex subunit 1; small nucleolar RNA, H/ACA box 50A
0,89	KIF15	kinesin family member 15
0,89	ACSBG1	acyl-CoA synthetase bubblegum family member 1

0,89	ZNF883	zinc finger protein 883
0,89	ATRN	attractin
0,89	UPP1	uridine phosphorylase 1
0,89	SSUH2	ssu-2 homolog (C. elegans)
0,89	RERGL	RERG/RAS-like
0,89	SSBP3	single stranded DNA binding protein 3
0,89	IL17D	interleukin 17D
0,89	FABP12	fatty acid binding protein 12
0,89	H1F0	H1 histone family, member 0
0,89	ANKRD39	ankyrin repeat domain 39
0,89	MAMLD1	mastermind-like domain containing 1
0,89	ZNF84	zinc finger protein 84
0,89	FAM221A	family with sequence similarity 221, member A
0,89	SNAP47	synaptosome associated protein 47kDa
0,89	CASC5	cancer susceptibility candidate 5
0,89	LOC730183; RP11-146F11.1; hitema	uncharacterized LOC730183; Transcript Identified by AceView; novel transcript, antisense to SRCAP
0,89	RAPGEF6	Rap guanine nucleotide exchange factor 6
0,89	CSRP2	cysteine and glycine-rich protein 2
0,89	GPR20	G protein-coupled receptor 20
0,89	RAET1G	retinoic acid early transcript 1G
0,89	AIFM3	apoptosis-inducing factor, mitochondrion-associated, 3
0,89	ANXA4	Salzman2013 ANTISENSE, coding, INTERNAL, intronic best transcript NM_001153
0,89	HSD17B3	hydroxysteroid (17-beta) dehydrogenase 3
0,89	CCDC83	coiled-coil domain containing 83
0,89	AFAP1L1	actin filament associated protein 1-like 1
0,89	CCDC191	coiled-coil domain containing 191
0,89	SLC10A2	solute carrier family 10 (sodium/bile acid cotransporter), member 2
0,89	WNT5B	wingless-type MMTV integration site family, member 5B
0,89	TESK2	testis-specific kinase 2
0,89	DGKD	diacylglycerol kinase, delta 130kDa
0,89	LOC283731; RP11-247C2.2	uncharacterized LOC283731; novel transcript
0,89	F5	coagulation factor V (proaccelerin, labile factor)
0,89	XPC	xeroderma pigmentosum, complementation group C
0,89	KBTBD11	kelch repeat and BTB (POZ) domain containing 11
0,89	KLK6	kallikrein related peptidase 6
0,89	FGF22	fibroblast growth factor 22
0,89	GALNT13	polypeptide N-acetylgalactosaminyltransferase 13
0,89	INSIG1	insulin induced gene 1
0,89	CLEC4D	C-type lectin domain family 4, member D
0,89	LRRC63	leucine rich repeat containing 63
0,89	PET117; CSRP2BP	PET117 homolog; CSRP2 binding protein
0,89	TRPS1	trichorhinophalangeal syndrome I

0,89	BCLAF1	Memczak2013 ALT_DONOR, coding, INTERNAL, intronic best transcript NM_014739
0,89	STK17B	serine/threonine kinase 17b
0,88	BTBD2	BTB (POZ) domain containing 2
0,88	PHC1	polyhomeotic homolog 1 (Drosophila)
0,88	CLSTN1	calsyntenin 1
0,88	ATG16L1	autophagy related 16-like 1
0,88	GUSBP2	glucuronidase, beta pseudogene 2
0,88	MMP25-AS1	MMP25 antisense RNA 1
0,88	TRIM49D1; TRIM49D2	tripartite motif containing 49D1; tripartite motif containing 49D2
0,88	SEC61B	Sec61 translocon beta subunit
0,88	FAM83H	family with sequence similarity 83, member H
0,88	TRIM43	tripartite motif containing 43
0,88	ZFHX4	zinc finger homeobox 4
0,88	SMAP1	small ArfGAP 1
0,88	SIX4	SIX homeobox 4
0,88	IDH2	isocitrate dehydrogenase 2 (NADP+), mitochondrial
0,88	CREM	cAMP responsive element modulator
0,88	ADCK2	aarF domain containing kinase 2
0,88	MARK4	MAP/microtubule affinity-regulating kinase 4
0,88	IRAK2	interleukin 1 receptor associated kinase 2
0,88	WSCD2	WSC domain containing 2
0,88	GLB1L	galactosidase beta 1 like
0,88	PPEF1	protein phosphatase, EF-hand calcium binding domain 1
0,88	KIF18B	Memczak2013 ALT_ACCEPTOR, coding, INTERNAL, intronic best transcript NM_001080443
0,88	SPTLC2	serine palmitoyltransferase, long chain base subunit 2
0,88	PLXNA4	plexin A4
0,88	SIGLEC9	sialic acid binding Ig-like lectin 9
0,88	IL13RA2	interleukin 13 receptor, alpha 2
0,88	ZNF48	zinc finger protein 48
0,88	ACSF3	acyl-CoA synthetase family member 3
0,88	CXorf36	chromosome X open reading frame 36
0,88	LILRA5	leukocyte immunoglobulin-like receptor, subfamily A (with TM domain), member 5
0,88	PURB; MIR4657	purine-rich element binding protein B; microRNA 4657
0,88	GP1BA	glycoprotein Ib (platelet), alpha polypeptide
0,88	PLEKHA4	pleckstrin homology domain containing, family A (phosphoinositide binding specific) member 4
0,88	ANO6	Transcript Identified by AceView, Entrez Gene ID(s) 196527
0,88	MAGEC2	MAGE family member C2
0,88	TMEM263	transmembrane protein 263
0,88	PDE4A	phosphodiesterase 4A, cAMP-specific
0,88	GH1	growth hormone 1
0,88	CREBBP	CREB binding protein
0,88	CD84	CD84 molecule

0,88	NOS2	nitric oxide synthase 2, inducible
0,88	ZC2HC1A	zinc finger, C2HC-type containing 1A
0,88	DTNB	dystrobrevin beta
0,88	LLGL1	lethal giant larvae homolog 1 (Drosophila)
0,88	RAB39B	Transcript Identified by AceView, Entrez Gene ID(s) 116442
0,88	MTRNR2L7	MT-RNR2-like 7
0,88	ALKBH5	alkB homolog 5, RNA demethylase
0,88	MYO15A	myosin XVA
0,88	BICD2	bicaudal D homolog 2 (Drosophila)
0,88	OR4C11	olfactory receptor, family 4, subfamily C, member 11
0,88	EFCAB2	EF-hand calcium binding domain 2
0,88	SPTBN1	spectrin, beta, non-erythrocytic 1
0,88	PTPN7	protein tyrosine phosphatase, non-receptor type 7
0,88	HAPLN4	hyaluronan and proteoglycan link protein 4
0,88	SAP30L	SAP30-like
0,88	RDH8	retinol dehydrogenase 8 (all-trans)
0,88	PRM1	protamine 1
0,88	CD8B	CD8b molecule
0,88	BCL2L14	BCL2-like 14 (apoptosis facilitator)
0,88	RCN1	reticulocalbin 1, EF-hand calcium binding domain
0,88	KIAA1958	KIAA1958
0,88	ZBTB20; MIR568	zinc finger and BTB domain containing 20; microRNA 568
0,88	SCAI; GOLGA1	suppressor of cancer cell invasion; golgin A1
0,88	ZNF827	zinc finger protein 827
0,88	NDRG4	NDRG family member 4
0,88	EMILIN3	elastin microfibril interfacer 3
0,88	GRAMD1B	GRAM domain containing 1B
0,88	ACR	acrosin
0,88	CTSB	cathepsin B
0,88	RPS9	Jeck2013 ANTISENSE, CDS, coding, INTERNAL, OVCODE, OVEXON, UTR3 best transcript NM_001013
0,88	GRAMD2	GRAM domain containing 2
0,88	CAD	carbamoyl-phosphate synthetase 2, aspartate transcarbamylase, and dihydroorotase
0,88	C2CD3	C2 calcium-dependent domain containing 3
0,88	LAMA5; MIR4758	laminin, alpha 5; microRNA 4758
0,88	C14orf79	chromosome 14 open reading frame 79
0,88	HIPK1	homeodomain interacting protein kinase 1
0,88	FAAP100	Fanconi anemia core complex associated protein 100
0,88	CNNM3	cyclin and CBS domain divalent metal cation transport mediator 3
0,88	MAP3K3	mitogen-activated protein kinase kinase kinase 3
0,88	RAP1GAP	RAP1 GTPase activating protein
0,88	MAP3K13	mitogen-activated protein kinase kinase kinase 13
0,88	RGR	retinal G protein coupled receptor
0,88	C19orf44	chromosome 19 open reading frame 44

0,88	DIAPH1	diaphanous-related formin 1
0,88	KRT17	keratin 17, type I
0,88	DCLRE1B	DNA cross-link repair 1B
0,88	ZP4	zona pellucida glycoprotein 4
0,88	CCDC103	coiled-coil domain containing 103
0,88	SYCE1L	synaptonemal complex central element protein 1-like
0,88	PCDHB4	protocadherin beta 4
0,88	ARRDC4	arrestin domain containing 4
0,88	ZNF564	zinc finger protein 564
0,88	MARK3	MAP/microtubule affinity-regulating kinase 3
0,88	HEXIM1	hexamethylene bis-acetamide inducible 1
0,88	RNF111	ring finger protein 111
0,88	PITPNM3	PITPNM family member 3
0,88	ITGB4	integrin beta 4
0,88	RASSF3	Ras association (RalGDS/AF-6) domain family member 3
0,88	PKD2	polycystic kidney disease 2 (autosomal dominant)
0,88	ZNF843	zinc finger protein 843
0,88	DHRS7	dehydrogenase/reductase (SDR family) member 7
0,88	RAB28	Jeck2013 ALT_ACCEPTOR, ALT_DONOR, coding, INTERNAL, intronic best transcript NM_004249
0,88	CD99L2	CD99 molecule-like 2
0,88	REL	v-rel avian reticuloendotheliosis viral oncogene homolog
0,88	C1orf168	chromosome 1 open reading frame 168
0,88	CILP2	cartilage intermediate layer protein 2
0,88	TOP3B	topoisomerase (DNA) III beta
0,88	EVI2A; EVI2B	ecotropic viral integration site 2A; ecotropic viral integration site 2B
0,88	FAM174B	family with sequence similarity 174, member B
0,88	TRIM46	tripartite motif containing 46
0,88	STK25	serine/threonine kinase 25
0,88	CCDC142; MRPL53	coiled-coil domain containing 142; mitochondrial ribosomal protein L53
0,88	MAPT	microtubule associated protein tau
0,88	CDKN3	cyclin-dependent kinase inhibitor 3
0,88	UQCR11	ubiquinol-cytochrome c reductase, complex III subunit XI
0,88	KLHDC1	kelch domain containing 1
0,88	RAB34	RAB34, member RAS oncogene family
0,88	BIRC3	Memczak2013 ANTISENSE, CDS, coding, INTERNAL best transcript NM_182962
0,88	PIK3C2A	phosphatidylinositol-4-phosphate 3-kinase, catalytic subunit type 2 alpha
0,88	DDRGK1	DDRGK domain containing 1
0,88	MAFB	v-maf avian musculoaponeurotic fibrosarcoma oncogene homolog B
0,88	ZNF551	zinc finger protein 551
0,88	CXCL12	chemokine (C-X-C motif) ligand 12
0,88	PLAUR	plasminogen activator, urokinase receptor

0,88	CCDC126	coiled-coil domain containing 126
0,88	POU2AF1	POU class 2 associating factor 1
0,88	APOA4	apolipoprotein A-IV
0,88	SERPINB7	serpin peptidase inhibitor, clade B (ovalbumin), member 7
0,88	SLC22A23	solute carrier family 22, member 23
0,88	FMNL2	formin like 2
0,88	SLC35G2	solute carrier family 35, member G2
0,88	TRIP4	thyroid hormone receptor interactor 4
0,88	FAM133A	family with sequence similarity 133, member A
0,88	C1orf74	chromosome 1 open reading frame 74
0,88	METRN	meteorin, glial cell differentiation regulator
0,88	ITGA3	integrin alpha 3
0,88	NPIPA7; NPIPA8; PKD1P1	nuclear pore complex interacting protein family, member A7; nuclear pore complex interacting protein family, member A8; polycystic kidney disease 1 (autosomal dominant) pseudogene 1
0,88	KRTAP13-1	keratin associated protein 13-1
0,88	DCAF8	DDB1 and CUL4 associated factor 8
0,88	ATP5S	ATP synthase, H+ transporting, mitochondrial Fo complex subunit s (factor B)
0,88	CPEB3	Transcript Identified by AceView, Entrez Gene ID(s) 22849
0,88	C9orf24	chromosome 9 open reading frame 24
0,88	PAPPA	pregnancy-associated plasma protein A, pappalysin 1
0,88	SSMEM1	serine-rich single-pass membrane protein 1
0,88	ERO1B	endoplasmic reticulum oxidoreductase beta
0,88	BTN2A2	butyrophilin, subfamily 2, member A2
0,88	PCMTD2	protein-L-isoaspartate (D-aspartate) O-methyltransferase domain containing 2
0,88	SLC28A3	solute carrier family 28 (concentrative nucleoside transporter), member 3
0,88	GPR82	G protein-coupled receptor 82
0,88	SLC26A9	solute carrier family 26 (anion exchanger), member 9
0,88	TACC3	transforming, acidic coiled-coil containing protein 3
0,88	A4GNT	alpha-1,4-N-acetylglucosaminyltransferase
0,88	CEMP1	cementum protein 1
0,88	NACAD	NAC alpha domain containing
0,88	SSH2	slingshot protein phosphatase 2
0,88	PRR7	proline rich 7 (synaptic)
0,88	IL15	interleukin 15
0,88	DMXL2	Dmx-like 2
0,88	RASAL2	RAS protein activator like 2
0,88	GPR139	G protein-coupled receptor 139
0,88	LRRC37A4P	leucine rich repeat containing 37, member A4, pseudogene
0,88	TRPM4	transient receptor potential cation channel, subfamily M, member 4

0,88	KIAA1804; MLK4	mixed lineage kinase 4; Mitogen-activated protein kinase kinase kinase MLK4 [Source:UniProtKB/Swiss-Prot;Acc:Q5TCX8]; Salzman2013 ANNOTATED, CDS, coding, INTERNAL, OVCODE, OVEXON best transcript NM_032435
0,88	SLC25A25	solute carrier family 25 (mitochondrial carrier; phosphate carrier), member 25
0,88	HCST	hematopoietic cell signal transducer
0,88	NPB	neuropeptide B
0,88	SPTSSA	serine palmitoyltransferase, small subunit A
0,88	GP1BB; SEPT5	glycoprotein Ib (platelet), beta polypeptide; septin 5
0,88	BICD1	bicaudal D homolog 1 (Drosophila)
0,88	ALG12	ALG12, alpha-1,6-mannosyltransferase
0,88	PHC1	Homo sapiens polyhomeotic homolog 1 (Drosophila), mRNA (cDNA clone MGC:87926 IMAGE:5788132), complete cds.; Homo sapiens polyhomeotic homolog 1 (Drosophila), mRNA (cDNA clone MGC:189745 IMAGE:9057069), complete cds.
0,88	SLC2A12	solute carrier family 2 (facilitated glucose transporter), member 12
0,88	CACFD1	calcium channel flower domain containing 1
0,88	CAMSAP1	calmodulin regulated spectrin-associated protein 1
0,88	KLK2	kallikrein related peptidase 2
0,88	C17orf105	chromosome 17 open reading frame 105
0,88	ISCA2	iron-sulfur cluster assembly 2
0,88	LPCAT1	lysophosphatidylcholine acyltransferase 1
0,88	DSC3	desmocollin 3
0,88	CDT1	chromatin licensing and DNA replication factor 1
0,88	GSTA2	glutathione S-transferase alpha 2
0,88	GIMAP2	GTPase, IMAP family member 2
0,88	CYP8B1	cytochrome P450, family 8, subfamily B, polypeptide 1
0,88	MC4R	melanocortin 4 receptor
0,88	CSF2RA	colony stimulating factor 2 receptor, alpha, low-affinity (granulocyte-macrophage)
0,88	OR10C1	olfactory receptor, family 10, subfamily C, member 1 (gene/pseudogene)
0,88	CMTM4	CKLF-like MARVEL transmembrane domain containing 4
0,88	SERTAD3	SERTA domain containing 3
0,88	GOLGA4	golgin A4
0,88	MRGPRG	MAS-related GPR, member G
0,88	HYOU1	hypoxia up-regulated 1
0,88	SMOC1	SPARC related modular calcium binding 1
0,88	SEMA4D	Memczak2013 ANTISENSE, coding, INTERNAL, UTR3 best transcript NM_006378
0,88	MUSK	muscle, skeletal, receptor tyrosine kinase
0,88	GDF5	growth differentiation factor 5
0,88	TANC1	tetratricopeptide repeat, ankyrin repeat and coiled-coil containing 1

0,88	ARTN	artemin
0,88	NBPF19	neuroblastoma breakpoint family, member 19
0,88	PPM1J	protein phosphatase, Mg2+/Mn2+ dependent, 1J
0,88	FAM24A	family with sequence similarity 24, member A
0,88	MTFP1	mitochondrial fission process 1
0,88	CGB; CGB5	chorionic gonadotropin, beta polypeptide; chorionic gonadotropin, beta polypeptide 5
0,88	CNN3	calponin 3, acidic
0,88	GTF2E2	general transcription factor IIE subunit 2
0,88	CHRND	cholinergic receptor, nicotinic delta
0,88	MIR6809; TNS1	microRNA 6809; tensin 1
0,88	MYL6	myosin light chain 6
0,88	DGKD	diacylglycerol kinase, delta 130kDa
0,88	SHPRH	SNF2 histone linker PHD RING helicase, E3 ubiquitin protein ligase
0,88	PCDH19	protocadherin 19
0,88	PDE3A	phosphodiesterase 3A, cGMP-inhibited
0,88	UTP20	UTP20 small subunit (SSU) processome component
0,88	ARSJ	arylsulfatase family, member J
0,88	DHX33	DEAH (Asp-Glu-Ala-His) box polypeptide 33
0,88	RAB13	RAB13, member RAS oncogene family
0,88	SPAG7	sperm associated antigen 7
0,88	ANKRD36C	ankyrin repeat domain 36C
0,88	GPX1	glutathione peroxidase 1
0,88	MLXIP	MLX interacting protein
0,88	DUSP23	dual specificity phosphatase 23
0,88	CHST8	carbohydrate (N-acetylgalactosamine 4-0) sulfotransferase 8
0,88	LRP6	LDL receptor related protein 6
0,88	GRIK3	glutamate receptor, ionotropic, kainate 3
0,88	TRIM33	tripartite motif containing 33
0,88	PRICKLE2	prickle homolog 2
0,88	OGFOD3	2-oxoglutarate and iron-dependent oxygenase domain containing 3
0,88	FAM135A	family with sequence similarity 135, member A
0,88	PAOX	polyamine oxidase (exo-N4-amino)
0,88	WDR63	WD repeat domain 63
0,88	FAHD1	fumarylacetoacetate hydrolase domain containing 1
0,88	OR13H1	olfactory receptor, family 13, subfamily H, member 1
0,88	SCN10A	sodium channel, voltage gated, type X alpha subunit
0,88	GSN	gelsolin
0,88	ESPN	espin
0,88	PAH	phenylalanine hydroxylase
0,88	BAZ2A	bromodomain adjacent to zinc finger domain 2A
0,88	ZNF556	zinc finger protein 556
0,88	ATAD2	ATPase family, AAA domain containing 2
0,88	LOC340074; CTC-321K16.4	uncharacterized LOC340074; Transcript Identified by AceView, Entrez Gene ID(s) 340074, RefSeq ID(s) NR_027127; novel transcript
0,88	ARHGAP19	Rho GTPase activating protein 19

0,88	SLC38A7	solute carrier family 38, member 7
0,88	RASA4	RAS p21 protein activator 4
0,88	GJC1	gap junction protein gamma 1
0,88	OR4S2	olfactory receptor, family 4, subfamily S, member 2
0,88	LRRC37A3	leucine rich repeat containing 37, member A3
0,88	UNC5C	unc-5 netrin receptor C
0,88	CD200R1L	CD200 receptor 1 like
0,88	STX16-NPEPL1	STX16-NPEPL1 readthrough (NMD candidate)
0,88	ZBED8	zinc finger, BED-type containing 8
0,88	HMCN2	hemicentin 2
0,88	PARD6G	par-6 family cell polarity regulator gamma
0,88	BEST3	bestrophin 3
0,88	PCDHB13	protocadherin beta 13
0,88	TMF1	TATA element modulatory factor 1
0,88	CXCR3	chemokine (C-X-C motif) receptor 3
0,88	HDDC2	HD domain containing 2
0,88	CWF19L1; SNORA12	CWF19-like 1, cell cycle control (S. pombe); small nucleolar RNA, H/ACA box 12
0,88	SMARCA4	SWI/SNF related, matrix associated, actin dependent regulator of chromatin, subfamily a, member 4
0,88	ZNF507	zinc finger protein 507
0,88	DLEU1	deleted in lymphocytic leukemia 1 (non-protein coding)
0,88	C1orf186	chromosome 1 open reading frame 186
0,88	HMBS	hydroxymethylbilane synthase
0,88	C11orf87	chromosome 11 open reading frame 87
0,88	CORO2B	Memczak2013 ALT_ACCEPTOR, ALT_DONOR, coding, INTERNAL, intronic best transcript NM_001190457
0,88	HSPB2; C11orf52; HSPB2-C11orf52	heat shock 27kDa protein 2; chromosome 11 open reading frame 52; HSPB2-C11orf52 readthrough (NMD candidate)
0,88	SLC25A26	solute carrier family 25 (S-adenosylmethionine carrier), member 26
0,88	ECHS1	enoyl-CoA hydratase, short chain, 1, mitochondrial
0,88	OR52D1	olfactory receptor, family 52, subfamily D, member 1
0,88	PPARGC1A	peroxisome proliferator-activated receptor gamma, coactivator 1 alpha
0,88	GUCY2F	guanylate cyclase 2F, retinal
0,88	HELLS	helicase, lymphoid-specific
0,88	C9orf163	chromosome 9 open reading frame 163
0,88	LOC650293	seven transmembrane helix receptor; Transcript Identified by AceView, Entrez Gene ID(s) 650293, RefSeq ID(s) NM_001040071
0,88	FXYD4	FXYD domain containing ion transport regulator 4
0,88	NCKAP5	NCK-associated protein 5
0,88	KCNJ14	potassium channel, inwardly rectifying subfamily J, member 14
0,88	KIF1A	kinesin family member 1A
0,88	MRPS12	mitochondrial ribosomal protein S12

0,88	GLRA2	glycine receptor alpha 2
0,88	SCAPER	S-phase cyclin A-associated protein in the ER
0,88	EPHB1	EPH receptor B1
0,88	KRT76	keratin 76, type II
0,88	TMEM254	transmembrane protein 254
0,88	ZNF224	zinc finger protein 224
0,88	PLCXD3	phosphatidylinositol-specific phospholipase C, X domain containing 3
0,88	STK32C	serine/threonine kinase 32C
0,88	ANGPTL3	angiopoietin like 3
0,88	HSD11B1L; RPL36	hydroxysteroid (11-beta) dehydrogenase 1-like; ribosomal protein L36
0,88	ALKBH4	alkB homolog 4, lysine demthylase
0,88	KBTBD7	kelch repeat and BTB (POZ) domain containing 7
0,88	TNFRSF19	tumor necrosis factor receptor superfamily, member 19
0,88	CNFN	cornifelin
0,88	TTC9	tetratricopeptide repeat domain 9
0,88	RORC	RAR-related orphan receptor C
0,88	C3orf20	chromosome 3 open reading frame 20
0,88	RFX3	regulatory factor X, 3 (influences HLA class II expression)
0,88	NPTN	neuroplastin
0,88	SSFA2	sperm specific antigen 2
0,88	LINC00336	long intergenic non-protein coding RNA 336
0,88	UBR5	ubiquitin protein ligase E3 component n-recognin 5
0,88	CARS2	cysteinyl-tRNA synthetase 2, mitochondrial (putative)
0,88	VPS8	Transcript Identified by AceView, Entrez Gene ID(s) 23355
0,88	STRBP; MIR600HG; MIR600	spermatid perinuclear RNA binding protein; MIR600 host gene; microRNA 600
0,88	KRTAP12-1	keratin associated protein 12-1
0,88	FAM210B	family with sequence similarity 210, member B
0,88	RAB20	RAB20, member RAS oncogene family
0,88	NEUROG1	neurogenin 1
0,88	LRRC19	leucine rich repeat containing 19
0,88	PICK1	protein interacting with PRKCA 1
0,88	FBLN1	fibulin 1
0,88	SPATA41	spermatogenesis associated 41 (non-protein coding)
0,88	FBXW11	F-box and WD repeat domain containing 11
0,88	GSTO2	glutathione S-transferase omega 2
0,88	PGAM5	PGAM family member 5, serine/threonine protein phosphatase, mitochondrial
0,88	C3orf38	chromosome 3 open reading frame 38
0,88	BACE2	beta-site APP-cleaving enzyme 2
0,88	CERCAM	cerebral endothelial cell adhesion molecule
0,88	SUN1	Sad1 and UNC84 domain containing 1
0,88	ZBTB25	zinc finger and BTB domain containing 25
0,88	RXFP4	relaxin/insulin-like family peptide receptor 4

0,88	SRF	serum response factor
0,88	PRAMEF8	PRAME family member 8
0,88	PELI2	pellino E3 ubiquitin protein ligase family member 2
0,88	ZNF746	zinc finger protein 746
0,88	C15orf62	chromosome 15 open reading frame 62
0,88	GALR2	galanin receptor 2
0,88	MTCH1	mitochondrial carrier 1
0,88	HOXD13	homeobox D13
0,88	ALDH1L1-AS1	ALDH1L1 antisense RNA 1
0,87	PIK3R3	phosphoinositide-3-kinase, regulatory subunit 3 (gamma)
0,87	ONECUT1	one cut homeobox 1
0,87	APCDD1L	adenomatosis polyposis coli down-regulated 1-like
0,87	FAM131B	family with sequence similarity 131, member B
0,87	ZNF184	zinc finger protein 184
0,87	LOC101927322; RP11-126O1.5	uncharacterized LOC101927322; novel transcript, antisense to MALT1
0,87	ZNF638	Transcript Identified by AceView, Entrez Gene ID(s) 27332
0,87	DECR1	2,4-dienoyl-CoA reductase 1, mitochondrial
0,87	CACFD1	calcium channel flower domain containing 1
0,87	LHFPL1	lipoma HMGIC fusion partner-like 1
0,87	KCNMB2	potassium channel subfamily M regulatory beta subunit 2
0,87	C1orf226	chromosome 1 open reading frame 226
0,87	ATP8B4	ATPase, class I, type 8B, member 4
0,87	NELFB	negative elongation factor complex member B
0,87	CASR	calcium-sensing receptor
0,87	NOTCH2NL	notch 2 N-terminal like
0,87	USP32P2; FAM106A; CCDC144B	ubiquitin specific peptidase 32 pseudogene 2; family with sequence similarity 106, member A; coiled-coil domain containing 144B (pseudogene)
0,87	TRMU	tRNA 5-methylaminomethyl-2-thiouridylate methyltransferase
0,87	RFWD2	ring finger and WD repeat domain 2, E3 ubiquitin protein ligase
0,87	SH3RF1	SH3 domain containing ring finger 1
0,87	KAT2B	K(lysine) acetyltransferase 2B
0,87	NUP210	nucleoporin 210kDa
0,87	RPP25	ribonuclease P/MRP 25kDa subunit
0,87	ZNF846	zinc finger protein 846
0,87	HAVCR1	hepatitis A virus cellular receptor 1
0,87	P3H4	prolyl 3-hydroxylase family member 4 (non-enzymatic)
0,87	ago-03	argonaute RISC catalytic component 3
0,87	SVBP	small vasohibin binding protein
0,87	HESX1	HESX homeobox 1
0,87	NRIP1	nuclear receptor interacting protein 1
0,87	SUCO	SUN domain containing ossification factor

0,87	ZMYM6NB; ZMYM6	ZMYM6 neighbor; zinc finger, MYM-type 6
0,87	C2orf16	chromosome 2 open reading frame 16
0,87	DEFB103A	defensin, beta 103A
0,87	PIANP	PILR alpha associated neural protein
0,87	GJC3	gap junction protein gamma 3
0,87	ZNF609	zinc finger protein 609
0,87	COX18	COX18 cytochrome c oxidase assembly factor
0,87	MCC	mutated in colorectal cancers
0,87	NFU1	NFU1 iron-sulfur cluster scaffold
0,87	RPS29	ribosomal protein S29
0,87	SSUH2	ssu-2 homolog (C. elegans)
0,87	PANK2	pantothenate kinase 2
0,87	IFT52	intraflagellar transport 52
0,87	DUPD1	dual specificity phosphatase and pro isomerase domain containing 1
0,87	TRIM45	tripartite motif containing 45
0,87	NINL	ninein-like
0,87	NLK	nemo-like kinase
0,87	RPL7A	ribosomal protein L7a
0,87	TBKBP1	TBK1 binding protein 1
0,87	ZNF853	zinc finger protein 853
0,87	RBP2	retinol binding protein 2, cellular
0,87	CPB1	carboxypeptidase B1 (tissue)
0,87	PRIMA1	proline rich membrane anchor 1
0,87	SMARCD2	SWI/SNF related, matrix associated, actin dependent regulator of chromatin, subfamily d, member 2
0,87	ART1	ADP-ribosyltransferase 1
0,87	SPINT3	serine peptidase inhibitor, Kunitz type, 3
0,87	ELFN1	extracellular leucine-rich repeat and fibronectin type III domain containing 1
0,87	MIR659; ANKRD54	microRNA 659; ankyrin repeat domain 54
0,87	FAM175A	family with sequence similarity 175, member A
0,87	KIAA1522	KIAA1522
0,87	GSTM2	glutathione S-transferase mu 2 (muscle)
0,87	NR0B2	nuclear receptor subfamily 0, group B, member 2
0,87	P2RY12	purinergic receptor P2Y, G-protein coupled, 12
0,87	GPR148	G protein-coupled receptor 148
0,87	SMR3B	submaxillary gland androgen regulated protein 3B
0,87	CEP70	centrosomal protein 70kDa
0,87	SREBF1	sterol regulatory element binding transcription factor 1
0,87	TCEA3	transcription elongation factor A (SII), 3
0,87	IGFBP4	insulin like growth factor binding protein 4
0,87	NAPRT	nicotinate phosphoribosyltransferase
0,87	PPM1E	Transcript Identified by AceView, Entrez Gene ID(s) 22843
0,87	SERP1	stress-associated endoplasmic reticulum protein 1
0,87	MRGPRX1	MAS-related GPR, member X1

0,87	ZKSCAN7	zinc finger with KRAB and SCAN domains 7
0,87	HLA-B	major histocompatibility complex, class I, B
0,87	SWI5	SWI5 homologous recombination repair protein
0,87	CCDC113	coiled-coil domain containing 113
0,87	CENPH	centromere protein H
0,87	SIPA1L1	signal-induced proliferation-associated 1 like 1
0,87	ATXN3	ataxin 3
0,87	DNAJC7	DnaJ (Hsp40) homolog, subfamily C, member 7
0,87	TMSB15A	thymosin beta 15a
0,87	TRPC3	transient receptor potential cation channel, subfamily C, member 3
0,87	MPPED1	metallophosphoesterase domain containing 1
0,87	RAB23	RAB23, member RAS oncogene family
0,87	HECTD2	HECT domain containing E3 ubiquitin protein ligase 2
0,87	SLC14A1	solute carrier family 14 (urea transporter), member 1 (Kidd blood group)
0,87	SQSTM1	Transcript Identified by AceView, Entrez Gene ID(s) 8878
0,87	GHR	growth hormone receptor
0,87	ESX1	ESX homeobox 1
0,87	LIF	leukemia inhibitory factor
0,87	DPYSL4	dihydropyrimidinase-like 4
0,87	RNF214	ring finger protein 214
0,87	CCDC65	coiled-coil domain containing 65
0,87	TSHZ2	teashirt zinc finger homeobox 2
0,87	ARHGAP42	Rho GTPase activating protein 42
0,87	PDK3	pyruvate dehydrogenase kinase, isozyme 3
0,87	CNPY4	canopy FGF signaling regulator 4
0,87	RP11-499E18.1; SLC39A8	novel transcript; Transcript Identified by AceView, Entrez Gene ID(s) 64116
0,87	KLHL40	kelch-like family member 40
0,87	NFKBIL1	nuclear factor of kappa light polypeptide gene enhancer in B-cells inhibitor-like 1
0,87	RBM26	Transcript Identified by AceView, Entrez Gene ID(s) 64062
0,87	SPACA6	sperm acrosome associated 6
0,87	ANKK1	ankyrin repeat and kinase domain containing 1
0,87	C2orf91	chromosome 2 open reading frame 91
0,87	POLR2G	polymerase (RNA) II (DNA directed) polypeptide G
0,87	GCN1; MIR4498	GCN1 eIF2 alpha kinase activator homolog; microRNA 4498
0,87	KRT222	keratin 222, type II
0,87	TBCD	tubulin folding cofactor D
0,87	ZNF135	zinc finger protein 135
0,87	LCN6	lipocalin 6
0,87	SRD5A2	steroid-5-alpha-reductase, alpha polypeptide 2 (3-oxo-5 alpha-steroid delta 4-dehydrogenase alpha 2)
0,87	PCNXL3	pecanex-like 3 (Drosophila)
0,87	NSUN6	NOP2/Sun domain family, member 6

0,87	CDH20	cadherin 20, type 2
0,87	MAP1LC3B	microtubule-associated protein 1 light chain 3 beta
0,87	FIBIN	fin bud initiation factor homolog (zebrafish)
0,87	PGLYRP4	peptidoglycan recognition protein 4
0,87	HAUS4; MIR4707	HAUS augmin like complex subunit 4; microRNA 4707
0,87	GPR153	G protein-coupled receptor 153
0,87	ZHX3	zinc fingers and homeoboxes 3
0,87	TAS2R31	taste receptor, type 2, member 31
0,87	ATP11A	ATPase, class VI, type 11A
0,87	AC171558.1	Olfactory receptor 4N4 [Source:UniProtKB/Swiss-Prot;Acc:Q8N0Y3]
0,87	GPR88	G protein-coupled receptor 88
0,87	CARD11	caspase recruitment domain family, member 11
0,87	LIN54	lin-54 DREAM MuvB core complex component
0,87	DHRS12	dehydrogenase/reductase (SDR family) member 12
0,87	CLTCL1	clathrin, heavy chain-like 1
0,87	MYO1G	myosin IG
0,87	IGSF3	immunoglobulin superfamily, member 3
0,87	CYLD	cylindromatosis (turban tumor syndrome)
0,87	CHST3	carbohydrate (chondroitin 6) sulfotransferase 3
0,87	WDR33	WD repeat domain 33
0,87	NUDT16	nudix hydrolase 16
0,87	LYL1	lymphoblastic leukemia associated hematopoiesis regulator 1
0,87	BAHD1	bromo adjacent homology domain containing 1
0,87	HECTD1	HECT domain containing E3 ubiquitin protein ligase 1
0,87	CAPSL	calcyphosine-like
0,87	OR13G1	olfactory receptor, family 13, subfamily G, member 1
0,87	PTGER1	prostaglandin E receptor 1
0,87	MFI2	antigen p97 (melanoma associated) identified by monoclonal antibodies 133.2 and 96.5
0,87	SAG	S-antigen; retina and pineal gland (arrestin)
0,87	MECP2	methyl-CpG binding protein 2
0,87	DEFB124	defensin, beta 124
0,87	LINC01620	long intergenic non-protein coding RNA 1620
0,87	DNMT1	DNA (cytosine-5-)-methyltransferase 1
0,87	LACC1	laccase (multicopper oxidoreductase) domain containing 1
0,87	DGKD	diacylglycerol kinase, delta 130kDa
0,87	AKAP13; MIR7706	A kinase (PRKA) anchor protein 13; microRNA 7706
0,87	FAM212B	family with sequence similarity 212, member B
0,87	FAM64A	family with sequence similarity 64, member A
0,87	ATXN7L3	ataxin 7-like 3
0,87	UCN2	urocortin 2
0,87	PAN3; RNU6-82P	PAN3 poly(A) specific ribonuclease subunit; RNA, U6 small nuclear 82, pseudogene
0,87	LAMB1	laminin, beta 1

0,87	FAM209A	family with sequence similarity 209, member A
0,87	FOXP1; RP11-298C2.1	Transcript Identified by AceView, Entrez Gene ID(s) 27086; novel transcript, sense intronic to FOXP1
0,87	KPNA6	Jeck2013 ALT_ACCEPTOR, ALT_DONOR, coding, INTERNAL, intronic best transcript NM_012316
0,87	GM2A	GM2 ganglioside activator
0,87	RRP36	ribosomal RNA processing 36
0,87	RAD51AP2	Transcript Identified by AceView, Entrez Gene ID(s) 729475
0,87	CD300E	CD300e molecule
0,87	DCHS1	dachsous cadherin-related 1
0,87	ZNF526	zinc finger protein 526
0,87	C1QTNF6	C1q and tumor necrosis factor related protein 6
0,87	MS4A15	membrane-spanning 4-domains, subfamily A, member 15
0,87	BBS7	Bardet-Biedl syndrome 7
0,87	PLEKHM3	pleckstrin homology domain containing, family M, member 3
0,87	GOLGA6L5P; GOLGA6L17P	golgin A6 family-like 5, pseudogene; golgin A6 family-like 17, pseudogene
0,87	MAEL	maelstrom spermatogenic transposon silencer
0,87	KCTD4	potassium channel tetramerization domain containing 4
0,87	PLEC	plectin
0,87	PPFIA2	protein tyrosine phosphatase, receptor type, f polypeptide (PTPRF), interacting protein (liprin), alpha 2
0,87	C1S	complement component 1, s subcomponent
0,87	OTUB2	OTU deubiquitinase, ubiquitin aldehyde binding 2
0,87	ZNF740	zinc finger protein 740
0,87	ELF2	E74-like factor 2 (ets domain transcription factor)
0,87	WDFY2	WD repeat and FYVE domain containing 2
0,87	EP400; SNORA49	E1A binding protein p400; small nucleolar RNA, H/ACA box 49
0,87	MROH2A	maestro heat-like repeat family member 2A
0,87	ADCY2	Transcript Identified by AceView, Entrez Gene ID(s) 108
0,87	ZNFX1	zinc finger, NFX1-type containing 1
0,87	ENPP2	ectonucleotide pyrophosphatase/phosphodiesterase 2
0,87	ONECUT3	one cut homeobox 3
0,87	PC	Memczak2013 ALT_ACCEPTOR, ALT_DONOR, coding, INTERNAL, intronic best transcript NM_022172
0,87	UBA1; tydee	Memczak2013 ANTISENSE, CDS, coding, INTERNAL best transcript NM_153280; Transcript Identified by AceView
0,87	CSAG1	chondrosarcoma associated gene 1
0,87	SRGN	Memczak2013 ANTISENSE, coding, INTERNAL, UTR3 best transcript NM_002727
0,87	LILRB4	leukocyte immunoglobulin-like receptor, subfamily B (with TM and ITIM domains), member 4

0,87	GAREM1	GRB2 associated regulator of MAPK1 1
0,87	TSC22D3	TSC22 domain family, member 3
0,87	UGGT1	UDP-glucose glycoprotein glucosyltransferase 1
0,87	RABL2A	RAB, member of RAS oncogene family-like 2A
0,87	GPR142	G protein-coupled receptor 142
0,87	KNTC1	kinetochore associated 1
0,87	HIST1H4F	histone cluster 1, H4f
0,87	DUXA	double homeobox A
0,87	CCBL1	cysteine conjugate-beta lyase, cytoplasmic
0,87	SLC5A12	solute carrier family 5 (sodium/monocarboxylate cotransporter), member 12
0,87	PTK2	protein tyrosine kinase 2
0,87	INO80C	INO80 complex subunit C
0,87	SPINK14	serine peptidase inhibitor, Kazal type 14 (putative)
0,87	EMD	emerin
0,87	CHD9	chromodomain helicase DNA binding protein 9
0,87	ZSWIM4	zinc finger, SWIM-type containing 4
0,87	CCNG2	cyclin G2
0,87	C2CD4B	C2 calcium-dependent domain containing 4B
0,87	PSME4	proteasome activator subunit 4
0,87	ERC1	ELKS/RAB6-interacting/CAST family member 1
0,87	MFHAS1	malignant fibrous histiocytoma amplified sequence 1
0,87	SPECC1	sperm antigen with calponin homology and coiled-coil domains 1
0,87	TLL1	tolloid like 1
0,87	VRTN	vertebrae development associated
0,87	SCRIB	Zhang2013 ALT_ACCEPTOR, ALT_DONOR, coding, INTERNAL, intronic best transcript NM_182706
0,87	ZXDA	zinc finger, X-linked, duplicated A
0,87	SUPT20HL2	SPT20 homolog, SAGA complex component-like 2
0,87	S100A7	S100 calcium binding protein A7
0,87	ARID2	AT rich interactive domain 2 (ARID, RFX-like)
0,87	ALKBH3; SEC14L1P1	alkB homolog 3, alpha-ketoglutarate-dependent dioxygenase; SEC14-like 1 pseudogene 1
0,87	SPRR2G	small proline-rich protein 2G
0,87	WEE2	WEE1 homolog 2 (S. pombe)
0,87	MAP2K5	mitogen-activated protein kinase kinase 5
0,87	DPCD	deleted in primary ciliary dyskinesia homolog (mouse)
0,87	SETX	senataxin
0,87	KLHDC7B	kelch domain containing 7B
0,87	CCL20	chemokine (C-C motif) ligand 20
0,87	COL12A1	collagen, type XII, alpha 1
0,87	NGDN	neuroguidin, EIF4E binding protein
0,87	PLEKHG3	pleckstrin homology domain containing, family G (with RhoGef domain) member 3
0,87	EFNA3	ephrin-A3
0,87	KIAA1324	KIAA1324

0,87	PAMR1	peptidase domain containing associated with muscle regeneration 1
0,87	CDKN2D	cyclin-dependent kinase inhibitor 2D (p19, inhibits CDK4)
0,87	EFHB	EF-hand domain family, member B
0,87	FGD1	FYVE, RhoGEF and PH domain containing 1
0,87	OSCAR	osteoclast associated, immunoglobulin-like receptor
0,87	ST5	Transcript Identified by AceView, Entrez Gene ID(s) 6764
0,87	TRIP12	Transcript Identified by AceView, Entrez Gene ID(s) 9320
0,87	CELF2	Transcript Identified by AceView, Entrez Gene ID(s) 10659
0,87	KRTAP13-2	keratin associated protein 13-2
0,87	AKT1	v-akt murine thymoma viral oncogene homolog 1
0,87	PLET1	placenta expressed transcript 1
0,87	ERC2	ELKS/RAB6-interacting/CAST family member 2
0,87	OR4D2	olfactory receptor, family 4, subfamily D, member 2
0,87	DLGAP3	discs, large (Drosophila) homolog-associated protein 3
0,87	C16orf59	chromosome 16 open reading frame 59
0,87	DHX16	DEAH (Asp-Glu-Ala-His) box polypeptide 16
0,87	SPATA22	spermatogenesis associated 22
0,87	ASB12	ankyrin repeat and SOCS box containing 12
0,87	ADGRG7	adhesion G protein-coupled receptor G7
0,87	PTPRG	protein tyrosine phosphatase, receptor type, G
0,87	KLHL29	kelch-like family member 29
0,87	HIGD1C	HIG1 hypoxia inducible domain family, member 1C
0,87	APOC4	apolipoprotein C-IV
0,87	MAGEB17	MAGE family member B17
0,87	AFAP1	actin filament associated protein 1
0,87	LINC00473	long intergenic non-protein coding RNA 473
0,87	KHDC3L	KH domain containing 3-like, subcortical maternal complex member
0,87	UBE2Q2	ubiquitin-conjugating enzyme E2Q family member 2
0,87	C16orf97	chromosome 16 open reading frame 97
0,87	MB21D2	Mab-21 domain containing 2
0,87	MAP3K19	mitogen-activated protein kinase kinase kinase 19
0,87	AXIN2	axin 2
0,87	PLEKHG6	pleckstrin homology domain containing, family G (with RhoGef domain) member 6
0,87	CD53	Memczak2013 ALT_ACCEPTOR, ALT_DONOR, coding, INTERNAL, intronic best transcript NM_001040033
0,87	VPS13B	vacuolar protein sorting 13 homolog B (yeast)
0,87	PARP14	poly(ADP-ribose) polymerase family member 14
0,87	ZNF503	zinc finger protein 503
0,87	TMEM150C	transmembrane protein 150C
0,87	AMFR	autocrine motility factor receptor, E3 ubiquitin protein ligase

0,87	NRBP1	nuclear receptor binding protein 1
0,87	TEC	tec protein tyrosine kinase
0,87	COL1A1	collagen, type I, alpha 1
0,87	FAM171B	family with sequence similarity 171, member B
0,87	OTP	orthopedia homeobox
0,87	RNF180	ring finger protein 180
0,87	DIP2A	disco-interacting protein 2 homolog A
0,87	GTSCR1	Gilles de la Tourette syndrome chromosome region, candidate 1 (non-protein coding)
0,87	ATP1B4	ATPase, Na+/K+ transporting, beta 4 polypeptide
0,87	MAP2K7	mitogen-activated protein kinase kinase 7
0,87	FLOT2	Memczak2013 ANTISENSE, coding, INTERNAL, UTR3 best transcript NM_004475
0,87	TENM3	teneurin transmembrane protein 3
0,87	CDK11A	cyclin-dependent kinase 11A
0,87	VCAN	versican
0,87	NDP	Norrie disease (pseudoglioma)
0,87	RNF145	ring finger protein 145
0,87	IQCE	IQ motif containing E
0,87	TP53I3	tumor protein p53 inducible protein 3
0,86	PABPC1L2A	poly(A) binding protein, cytoplasmic 1-like 2A
0,86	PMCH	pro-melanin-concentrating hormone
0,86	KIAA1109	KIAA1109
0,86	LINC01126	long intergenic non-protein coding RNA 1126
0,86	PRPF8	pre-mRNA processing factor 8
0,86	NUDT9	nudix hydrolase 9
0,86	MYO7A	myosin VIIA
0,86	RBPMS	RNA binding protein with multiple splicing
0,86	CUL9	cullin 9
0,86	ZNF143	zinc finger protein 143
0,86	KRTAP20-4	keratin associated protein 20-4
0,86	C8orf37	chromosome 8 open reading frame 37
0,86	APPL1	adaptor protein, phosphotyrosine interaction, PH domain and leucine zipper containing 1
0,86	INPP4B	inositol polyphosphate-4-phosphatase type II B
0,86	RP5-1198O20.4; KLF17	Transcript Identified by AceView, Entrez Gene ID(s) 128209; novel transcript
0,86	CDC42BPA	CDC42 binding protein kinase alpha (DMPK-like)
0,86	ZFP30	ZFP30 zinc finger protein
0,86	LAMB2	laminin, beta 2 (laminin S)
0,86	DPH3P1	diphthamide biosynthesis 3 pseudogene 1
0,86	USP4	ubiquitin specific peptidase 4 (proto-oncogene)
0,86	CFAP54	cilia and flagella associated 54
0,86	GOLGA8A; GOLGA8B	golgin A8 family, member A; golgin A8 family, member B
0,86	HNRNPUL2	heterogeneous nuclear ribonucleoprotein U-like 2
0,86	AZIN2	antizyme inhibitor 2
0,86	MSANTD1	Myb/SANT-like DNA-binding domain containing 1
0,86	ZCCHC18	zinc finger, CCHC domain containing 18
0,86	SURF1	surfeit 1

0,86	ST7-AS1	ST7 antisense RNA 1
0,86	LHFPL3	lipoma HMGIC fusion partner-like 3
0,86	CHST1	carbohydrate (keratan sulfate Gal-6) sulfotransferase 1
0,86	CSRNP2	cysteine-serine-rich nuclear protein 2
0,86	ZNF18	zinc finger protein 18
0,86	FBXL7	F-box and leucine-rich repeat protein 7
0,86	GCH1	GTP cyclohydrolase 1
0,86	ADAM11	ADAM metallopeptidase domain 11
0,86	TP53INP2	tumor protein p53 inducible nuclear protein 2
0,86	C10orf120	chromosome 10 open reading frame 120
0,86	BAD	BCL2-associated agonist of cell death
0,86	XIRP2	xin actin binding repeat containing 2
0,86	OR4D9	olfactory receptor, family 4, subfamily D, member 9
0,86	SPATA21	spermatogenesis associated 21
0,86	FZR1	fizzy/cell division cycle 20 related 1
0,86	CYP4F12	cytochrome P450, family 4, subfamily F, polypeptide 12
0,86	MAP6	microtubule associated protein 6
0,86	LOC654841; AC097662.2; COL4A3	uncharacterized LOC654841; Salzman2013 ANTISENSE, CDS, coding, INTERNAL, intronic, OVCODE, OVERLAPTX, OVEXON best transcript NM_000091; novel transcript, antisense to COL4A3
0,86	TBC1D7	TBC1 domain family, member 7
0,86	AACS	acetoacetyl-CoA synthetase
0,86	C17orf64	chromosome 17 open reading frame 64
0,86	FTHL17	ferritin, heavy polypeptide-like 17
0,86	THBS2	thrombospondin 2
0,86	WWC2-AS2	WWC2 antisense RNA 2
0,86	ODF3L1	outer dense fiber of sperm tails 3-like 1
0,86	EPX	eosinophil peroxidase
0,86	PDZK1IP1	PDZK1 interacting protein 1
0,86	LMO4	LIM domain only 4
0,86	TNKS2	tankyrase, TRF1-interacting ankyrin-related ADP-ribose polymerase 2
0,86	SIX1	SIX homeobox 1
0,86	JPH3	junctophilin 3
0,86	AMZ1	archaelysin family metallopeptidase 1
0,86	USP34	ubiquitin specific peptidase 34
0,86	NSD1	nuclear receptor binding SET domain protein 1
0,86	ZNF419	zinc finger protein 419
0,86	MFSD2B	major facilitator superfamily domain containing 2B
0,86	MAGEB10	MAGE family member B10
0,86	ACBD6	acyl-CoA binding domain containing 6
0,86	PLD1	phospholipase D1, phosphatidylcholine-specific
0,86	EIF3F	Eukaryotic translation initiation factor 3 subunit F [Source:UniProtKB/Swiss-Prot;Acc:O00303]
0,86	GPR19	G protein-coupled receptor 19
0,86	BCORL1	BCL6 corepressor-like 1
0,86	ZFPM2	zinc finger protein, FOG family member 2

0,86	OR51M1	olfactory receptor, family 51, subfamily M, member 1
0,86	DDR2	discoidin domain receptor tyrosine kinase 2
0,86	SMG6	SMG6 nonsense mediated mRNA decay factor
0,86	SEMA3B; MIR6872	sema domain, immunoglobulin domain (Ig), short basic domain, secreted, (semaphorin) 3B; microRNA 6872
0,86	ALDH4A1	aldehyde dehydrogenase 4 family, member A1
0,86	GFER	growth factor, augmenter of liver regeneration
0,86	CREB3L3	cAMP responsive element binding protein 3-like 3
0,86	ZNF383	zinc finger protein 383
0,86	ARHGAP31	Rho GTPase activating protein 31
0,86	POGZ	Transcript Identified by AceView, Entrez Gene ID(s) 23126
0,86	THADA	Transcript Identified by AceView, Entrez Gene ID(s) 63892
0,86	LILRB3	leukocyte immunoglobulin-like receptor, subfamily B (with TM and ITIM domains), member 3
0,86	SYT6	synaptotagmin VI
0,86	ARVCF	Zhang2013 ALT_ACCEPTOR, ALT_DONOR, coding, INTERNAL, intronic best transcript NM_001670
0,86	ADGRL2	adhesion G protein-coupled receptor L2
0,86	DLGAP2	discs, large (Drosophila) homolog-associated protein 2
0,86	REXO4	REX4 homolog, 3'-5' exonuclease [Source:HGNC Symbol;Acc:HGNC:12820]
0,86	SPINK6	serine peptidase inhibitor, Kazal type 6
0,86	ZNF154	zinc finger protein 154
0,86	POTEA	POTE ankyrin domain family, member A
0,86	PRRG2	proline rich Gla (G-carboxyglutamic acid) 2
0,86	HLA-DOA	major histocompatibility complex, class II, DO alpha
0,86	RUSC1-AS1	RUSC1 antisense RNA 1
0,86	LNX1	ligand of numb-protein X 1, E3 ubiquitin protein ligase
0,86	MAL	mal, T-cell differentiation protein
0,86	PPIL6	peptidylprolyl isomerase (cyclophilin)-like 6
0,86	OR56A5	olfactory receptor, family 56, subfamily A, member 5
0,86	DRICH1	aspartate-rich 1
0,86	CYP7B1	cytochrome P450, family 7, subfamily B, polypeptide 1
0,86	PPM1M	protein phosphatase, Mg2+/Mn2+ dependent, 1M
0,86	ZNF76	zinc finger protein 76
0,86	CCNE2	cyclin E2
0,86	DGKD	diacylglycerol kinase, delta 130kDa
0,86	CHRNB1	cholinergic receptor, nicotinic beta 1
0,86	CENPF	centromere protein F
0,86	NME3	NME/NM23 nucleoside diphosphate kinase 3
0,86	THRSP	thyroid hormone responsive
0,86	TMCO2	transmembrane and coiled-coil domains 2
0,86	DOC2A	double C2-like domains, alpha

0,86	INSC	inscuteable homolog (Drosophila)
0,86	C5orf58	chromosome 5 open reading frame 58
0,86	PLCL2; MIR3714	phospholipase C-like 2; microRNA 3714
0,86	INHBE	inhibin beta E
0,86	INPP5D	inositol polyphosphate-5-phosphatase D
0,86	CX3CR1	chemokine (C-X3-C motif) receptor 1
0,86	SLC25A2	solute carrier family 25 (mitochondrial carrier; ornithine transporter) member 2
0,86	KRTAP24-1	keratin associated protein 24-1
0,86	MKI67	marker of proliferation Ki-67
0,86	FPR2	formyl peptide receptor 2
0,86	VN1R2	vomeronasal 1 receptor 2
0,86	CC2D1B	coiled-coil and C2 domain containing 1B
0,86	ZNF529	zinc finger protein 529
0,86	MAB21L3	mab-21-like 3 (C. elegans)
0,86	PI4KA	phosphatidylinositol 4-kinase, catalytic, alpha
0,86	MYOT	myotilin
0,86	FKBP6	FK506 binding protein 6
0,86	NAP1L5	nucleosome assembly protein 1-like 5
0,86	WDFY4	WDFY family member 4
0,86	HINT2	histidine triad nucleotide binding protein 2
0,86	MATN3	matrilin 3
0,86	CMKLR1	chemerin chemokine-like receptor 1
0,86	SNX10	sorting nexin 10
0,86	TNFRSF14	tumor necrosis factor receptor superfamily, member 14
0,86	ARL5C	ADP-ribosylation factor like GTPase 5C
0,86	SERPINI1	serpin peptidase inhibitor, clade I (neuroserpin), member 1
0,86	DUSP16	dual specificity phosphatase 16
0,86	GANC	glucosidase, alpha; neutral C
0,86	SGSH	N-sulfoglucosamine sulfohydrolase
0,86	YPEL4	yippee like 4
0,86	SIPA1L2	signal-induced proliferation-associated 1 like 2
0,86	TRAPPC4	trafficking protein particle complex 4
0,86	NEB	nebulin
0,86	WFDC8	WAP four-disulfide core domain 8
0,86	MMD	monocyte to macrophage differentiation-associated
0,86	APOD	apolipoprotein D
0,86	TMEM94; MIR6785	transmembrane protein 94; microRNA 6785
0,86	NOTCH2	notch 2
0,86	GPR37	G protein-coupled receptor 37 (endothelin receptor type B-like)
0,86	FKRP	fukutin related protein
0,86	SIRPD	signal-regulatory protein delta
0,86	DYNLL2	Memczak2013 ANTISENSE, coding, INTERNAL, UTR3 best transcript NM_080677
0,86	HLA-DRA	major histocompatibility complex, class II, DR alpha
0,86	PTCHD3	patched domain containing 3

0,86	HMCN1	hemicentin 1
0,86	ZIC1	Zic family member 1
0,86	OR2F2	olfactory receptor, family 2, subfamily F, member 2
0,86	MCEMP1	mast cell-expressed membrane protein 1
0,86	ETV7	ets variant 7
0,86	SLFN12L	schlafen family member 12-like
0,86	DDIT4L	DNA-damage-inducible transcript 4-like
0,86	DYDC2	DPY30 domain containing 2
0,86	MICAL1	microtubule associated monooxygenase, calponin and LIM domain containing 1
0,86	HLA-DMA	major histocompatibility complex, class II, DM alpha
0,86	TRPM5	transient receptor potential cation channel, subfamily M, member 5
0,86	SERAC1	serine active site containing 1
0,86	ACVR1C	activin A receptor type IC
0,86	ILVBL	ilvB (bacterial acetolactate synthase)-like
0,86	PMAIP1	phorbol-12-myristate-13-acetate-induced protein 1
0,86	SPATA18	spermatogenesis associated 18
0,86	ASCL2	achaete-scute family bHLH transcription factor 2
0,86	FAM3A	family with sequence similarity 3, member A
0,86	GTPBP3	Memczak2013 ANTISENSE, CDS, coding, INTERNAL best transcript NM_001195422
0,86	C6orf99	chromosome 6 open reading frame 99
0,86	RFX4	Transcript Identified by AceView, Entrez Gene ID(s) 5992
0,86	DYRK2	dual specificity tyrosine-(Y)-phosphorylation regulated kinase 2
0,86	MAP2	microtubule associated protein 2
0,86	CCDC34	coiled-coil domain containing 34
0,86	PLBD1	phospholipase B domain containing 1
0,86	CDCP2	CUB domain containing protein 2
0,86	ZNF691	zinc finger protein 691
0,86	BCOR	BCL6 corepressor
0,86	DUX1	double homeobox 1
0,86	PACRG-AS3	PACRG antisense RNA 3
0,86	MESP1	mesoderm posterior bHLH transcription factor 1
0,86	KLHL2	kelch-like family member 2
0,86	KPTN	kaptin (actin binding protein)
0,86	COL4A6	collagen, type IV, alpha 6
0,86	ANKRD26	ankyrin repeat domain 26
0,86	DSP	desmoplakin
0,86	STRIP1	striatin interacting protein 1
0,86	TPRG1	tumor protein p63 regulated 1
0,86	DTX3L	deltex 3 like, E3 ubiquitin ligase
0,86	MAST2	microtubule associated serine/threonine kinase 2
0,86	IKBKAP	inhibitor of kappa light polypeptide gene enhancer in B-cells, kinase complex-associated protein
0,86	P4HA2	prolyl 4-hydroxylase, alpha polypeptide II
0,86	CRIPAK	cysteine-rich PAK1 inhibitor
0,86	OVOL1	ovo-like zinc finger 1

0,86	CACNA1C	calcium channel, voltage-dependent, L type, alpha 1C subunit
0,86	QTRT1	queuine tRNA-ribosyltransferase 1
0,86	RAB3C	RAB3C, member RAS oncogene family
0,86	MAP1B	microtubule associated protein 1B
0,86	TMEM150B	transmembrane protein 150B
0,86	MRC2	mannose receptor, C type 2
0,86	TTLL7	tubulin tyrosine ligase-like family member 7
0,86	CCDC121	coiled-coil domain containing 121
0,86	LAIR1	leukocyte-associated immunoglobulin-like receptor 1
0,86	SLC6A17	solute carrier family 6 (neutral amino acid transporter), member 17
0,86	SYNPO2L	synaptopodin 2-like
0,86	LIPJ	lipase, family member J
0,86	NLGN2	neuroligin 2
0,86	OR9I1	olfactory receptor, family 9, subfamily I, member 1
0,86	INPP5D	inositol polyphosphate-5-phosphatase D
0,86	SYNM	synemin, intermediate filament protein
0,86	BRMS1L	breast cancer metastasis-suppressor 1-like
0,86	HACD4	3-hydroxyacyl-CoA dehydratase 4
0,86	LRP11	LDL receptor related protein 11
0,86	SCGN	secretagogin, EF-hand calcium binding protein
0,86	HMX2	H6 family homeobox 2
0,86	TNKS	tankyrase, TRF1-interacting ankyrin-related ADP-ribose polymerase
0,86	HMBS	hydroxymethylbilane synthase
0,86	MCUR1	mitochondrial calcium uniporter regulator 1
0,86	TM6SF2	transmembrane 6 superfamily member 2
0,86	BCR	breakpoint cluster region
0,86	ZNF106	zinc finger protein 106
0,86	PAFAH2	platelet-activating factor acetylhydrolase 2
0,86	OGFR	opioid growth factor receptor
0,86	TMEM171	transmembrane protein 171
0,86	DNAJB13	DnaJ (Hsp40) homolog, subfamily B, member 13
0,86	SEMA6C	sema domain, transmembrane domain (TM), and cytoplasmic domain, (semaphorin) 6C
0,86	SETD4	SET domain containing 4
0,85	CST1	cystatin SN
0,85	CDC42BPB	CDC42 binding protein kinase beta (DMPK-like)
0,85	SLC38A8	Transcript Identified by AceView, Entrez Gene ID(s) 146167
0,85	KL	klotho
0,85	ADAM20	ADAM metallopeptidase domain 20
0,85	CACFD1	calcium channel flower domain containing 1
0,85	OMG	oligodendrocyte myelin glycoprotein
0,85	SDHAF2	Memczak2013 ALT_ACCEPTOR, ALT_DONOR, coding, INTERNAL, intronic best transcript NM_017841
0,85	OSGEPL1	O-sialoglycoprotein endopeptidase-like 1
0,85	ATP11B	ATPase, class VI, type 11B

0,85	PIGF	phosphatidylinositol glycan anchor biosynthesis class F
0,85	PRSS55	protease, serine, 55
0,85	MYLK3	myosin light chain kinase 3
0,85	ZNF414	zinc finger protein 414
0,85	WDR7	WD repeat domain 7
0,85	ISG15	ISG15 ubiquitin-like modifier
0,85	TMEM88B	transmembrane protein 88B
0,85	PCDH9	protocadherin 9
0,85	PTK2	Transcript Identified by AceView, Entrez Gene ID(s) 5747
0,85	ERICH6B	glutamate rich 6B
0,85	GDF9	growth differentiation factor 9
0,85	CTSH	cathepsin H
0,85	OR52E1	olfactory receptor, family 52, subfamily E, member 1 (gene/pseudogene)
0,85	TRAF1	TNF receptor-associated factor 1
0,85	SRRM3	serine/arginine repetitive matrix 3
0,85	CSGALNACT2	chondroitin sulfate N-acetylgalactosaminyltransferase 2
0,85	ZNF398	zinc finger protein 398
0,85	FRZB	frizzled-related protein
0,85	RNF24	ring finger protein 24
0,85	EPCAM	epithelial cell adhesion molecule
0,85	STK38L	serine/threonine kinase 38 like
0,85	CTNND2	catenin (cadherin-associated protein), delta 2
0,85	ADAL	adenosine deaminase-like
0,85	PTGER4	prostaglandin E receptor 4 (subtype EP4)
0,85	IMMP2L	inner mitochondrial membrane peptidase subunit 2
0,85	C9orf57	chromosome 9 open reading frame 57
0,85	NUBP2	nucleotide binding protein 2
0,85	ZNF709	zinc finger protein 709
0,85	LPAR5	lysophosphatidic acid receptor 5
0,85	CDH4	cadherin 4, type 1, R-cadherin (retinal)
0,85	CCL19	chemokine (C-C motif) ligand 19
0,85	PDE4C	phosphodiesterase 4C, cAMP-specific
0,85	PKLR	pyruvate kinase, liver and RBC
0,85	TBC1D9B	TBC1 domain family, member 9B (with GRAM domain)
0,85	KIF15	kinesin family member 15
0,85	ACKR3	atypical chemokine receptor 3
0,85	SERP1	stress-associated endoplasmic reticulum protein 1
0,85	SLC7A2	solute carrier family 7 (cationic amino acid transporter, y+ system), member 2
0,85	AK9	adenylate kinase 9
0,85	RRP8	Transcript Identified by AceView, Entrez Gene ID(s) 23378
0,85	EVA1C	eva-1 homolog C (C. elegans)
0,85	PPP1R13B	protein phosphatase 1, regulatory subunit 13B
0,85	TCEA2	transcription elongation factor A (SII), 2

0,85	NFATC2	nuclear factor of activated T-cells, cytoplasmic, calcineurin-dependent 2
0,85	OLFML2B	olfactomedin like 2B
0,85	ST3GAL3; MIR6079	ST3 beta-galactoside alpha-2,3-sialyltransferase 3; microRNA 6079
0,85	ATP2A3	Memczak2013 ANTISENSE, CDS, coding, INTERNAL best transcript NM_174956
0,85	PCOLCE	procollagen C-endopeptidase enhancer
0,85	LRCH1	leucine-rich repeats and calponin homology (CH) domain containing 1
0,85	KCNT1	potassium channel, sodium activated subfamily T, member 1
0,85	GPM6B	glycoprotein M6B
0,85	BAG1	BCL2-associated athanogene
0,85	SLC2A10	solute carrier family 2 (facilitated glucose transporter), member 10
0,85	NHLH1	nescient helix-loop-helix 1
0,85	TMEM74	transmembrane protein 74
0,85	NEO1	neogenin 1
0,85	KATNAL2	katanin p60 subunit A-like 2
0,85	KCNQ2	potassium channel, voltage gated KQT-like subfamily Q, member 2
0,85	KRT33A	keratin 33A, type I
0,85	ARMS2	age-related maculopathy susceptibility 2
0,85	KBTBD2	kelch repeat and BTB (POZ) domain containing 2
0,85	KRTAP2-3	keratin associated protein 2-3
0,85	LDLRAD4	low density lipoprotein receptor class A domain containing 4
0,85	B3GALT1	UDP-Gal:betaGlcNAc beta 1,3-galactosyltransferase 1
0,85	MYH9	myosin, heavy chain 9, non-muscle
0,85	NCBP2-AS2	NCBP2 antisense RNA 2 (head to head)
0,85	SLC6A2	solute carrier family 6 (neurotransmitter transporter), member 2
0,85	MANF	mesencephalic astrocyte-derived neurotrophic factor
0,85	ATP9B	ATPase, class II, type 9B
0,85	CHPF2; MIR671	chondroitin polymerizing factor 2; microRNA 671
0,85	SERF2	small EDRK-rich factor 2
0,85	TET1	tet methylcytosine dioxygenase 1
0,85	IFNA16	interferon, alpha 16
0,85	ADGRD1	adhesion G protein-coupled receptor D1
0,85	KDM4C	Memczak2013 ANTISENSE, coding, INTERNAL, intronic best transcript NM_001146694
0,85	GUCY1B3	guanylate cyclase 1, soluble, beta 3
0,85	MATN2	matrilin 2
0,85	CHRDL1	chordin-like 1
0,85	FOXA2	forkhead box A2
0,85	SMC1B	structural maintenance of chromosomes 1B
0,85	RYR3	ryanodine receptor 3
0,85	FMO6P	flavin containing monooxygenase 6 pseudogene

0,85	ABCA1	ATP binding cassette subfamily A member 1
0,85	PDPR	pyruvate dehydrogenase phosphatase regulatory subunit
0,85	CACNA2D3	calcium channel, voltage-dependent, alpha 2/delta subunit 3
0,85	SUGP2	SURP and G-patch domain containing 2
0,85	SLC38A9	Transcript Identified by AceView, Entrez Gene ID(s) 153129
0,85	FAM63B	family with sequence similarity 63, member B
0,85	AMOTL1	angiomotin like 1
0,85	PHYKPL	5-phosphohydroxy-L-lysine phospho-lyase
0,85	COL14A1	collagen, type XIV, alpha 1
0,85	SPX	spexin hormone
0,85	POLM; MIR6838	polymerase (DNA directed), mu; microRNA 6838
0,85	PKD2L2	polycystic kidney disease 2-like 2
0,85	SP9	Sp9 transcription factor
0,85	CGN	cingulin
0,85	ACYP2	acylphosphatase 2, muscle type
0,85	LTBP2	latent transforming growth factor beta binding protein 2
0,85	ZNF469	zinc finger protein 469
0,85	ZNF285	zinc finger protein 285
0,85	TMEM159	transmembrane protein 159
0,85	CPNE5	copine V
0,85	ANG; RNASE4	angiogenin, ribonuclease, RNase A family, 5; ribonuclease, RNase A family, 4
0,85	DIRC3	disrupted in renal carcinoma 3
0,85	FEM1C	Jeck2013 ALT_ACCEPTOR, ALT_DONOR, coding, INTERNAL, intronic best transcript NM_020177
0,85	HNRNPCL3	heterogeneous nuclear ribonucleoprotein C-like 3 [Source:HGNC Symbol;Acc:HGNC:51235]
0,85	CHD7	chromodomain helicase DNA binding protein 7
0,85	POTEF	POTE ankyrin domain family, member F
0,85	ZNF543	zinc finger protein 543
0,85	NUTM2G	NUT family member 2G
0,85	ALG6	ALG6, alpha-1,3-glucosyltransferase
0,85	BMP6	bone morphogenetic protein 6
0,85	BORCS5	BLOC-1 related complex subunit 5
0,85	PIDD1	p53-induced death domain protein 1
0,85	PRKCH	protein kinase C, eta
0,85	ACACA	acetyl-CoA carboxylase alpha
0,85	TGDS	TDP-glucose 4,6-dehydratase
0,85	DST	dystonin
0,85	MYB-AS1; MYB; RP1-32B1.3; rawjer	MYB antisense RNA 1 [Source:HGNC Symbol;Acc:HGNC:37457]; Memczak2013 ANTISENSE, coding, INTERNAL, intronic best transcript NM_005375; putative novel transcript; Transcript Identified by AceView
0,85	TFPT	TCF3 (E2A) fusion partner (in childhood Leukemia)

0,85	VTI1A	vesicle transport through interaction with t-SNAREs 1A
0,85	ZBTB41	zinc finger and BTB domain containing 41
0,85	IL9	interleukin 9
0,85	ACSL6	acyl-CoA synthetase long-chain family member 6
0,85	ATP6V0D2	ATPase, H+ transporting, lysosomal 38kDa, V0 subunit d2
0,85	AHCYL2	adenosylhomocysteinase-like 2
0,85	PTPRF	protein tyrosine phosphatase, receptor type, F
0,85	DHDH	dihydrodiol dehydrogenase (dimeric)
0,85	COMMD10	COMM domain containing 10
0,85	TRIM68	tripartite motif containing 68
0,85	RPS6KA1	ribosomal protein S6 kinase, 90kDa, polypeptide 1
0,85	FCGR1A	Fc fragment of IgG, high affinity Ia, receptor (CD64)
0,85	UCP2	uncoupling protein 2 (mitochondrial, proton carrier)
0,85	TRIM23	tripartite motif containing 23
0,85	OR1B1	olfactory receptor, family 1, subfamily B, member 1 (gene/pseudogene)
0,85	ATP2C2	ATPase, Ca++ transporting, type 2C, member 2
0,85	TINF2	TERF1 (TRF1)-interacting nuclear factor 2
0,85	MORC1	MORC family CW-type zinc finger 1
0,85	SYT11	synaptotagmin XI
0,85	STARD7-AS1	STARD7 antisense RNA 1
0,85	NOX4	NADPH oxidase 4
0,85	RASGEF1B	RasGEF domain family member 1B
0,85	C12orf40	chromosome 12 open reading frame 40
0,85	RNASE3	ribonuclease, RNase A family, 3
0,85	FURIN	furin (paired basic amino acid cleaving enzyme)
0,85	C9orf72	chromosome 9 open reading frame 72
0,85	NDUFS7	NADH dehydrogenase (ubiquinone) Fe-S protein 7, 20kDa (NADH-coenzyme Q reductase)
0,85	PTX4	pentraxin 4, long
0,85	ARID1A	AT rich interactive domain 1A (SWI-like)
0,85	ZNF594	zinc finger protein 594
0,85	C1orf105	chromosome 1 open reading frame 105
0,85	MUM1L1	melanoma associated antigen (mutated) 1-like 1
0,85	ATAD5	ATPase family, AAA domain containing 5
0,85	OR2Z1	olfactory receptor, family 2, subfamily Z, member 1
0,85	GOLGA6L10	golgin A6 family-like 10
0,85	KCNMB1	potassium channel subfamily M regulatory beta subunit 1
0,85	WWP1	Transcript Identified by AceView, Entrez Gene ID(s) 11059
0,85	RPS6KL1	ribosomal protein S6 kinase-like 1
0,85	NEUROD4	neuronal differentiation 4
0,85	AKAP2	A kinase (PRKA) anchor protein 2
0,85	SAG	S-antigen; retina and pineal gland (arrestin)
0,85	TTC12	tetratricopeptide repeat domain 12
0,85	GRM1	glutamate receptor, metabotropic 1
0,85	SPATA2L	spermatogenesis associated 2-like

0,85	SLC16A1	solute carrier family 16 (monocarboxylate transporter), member 1
0,85	HECTD4; MIR6861	HECT domain containing E3 ubiquitin protein ligase 4; microRNA 6861
0,85	C10orf71	chromosome 10 open reading frame 71
0,85	MRAS	muscle RAS oncogene homolog
0,85	VIM-AS1	VIM antisense RNA 1
0,85	ZNF565	zinc finger protein 565
0,85	TRPM3	transient receptor potential cation channel, subfamily M, member 3
0,85	PPARGC1B	peroxisome proliferator-activated receptor gamma, coactivator 1 beta
0,85	ESYT2	extended synaptotagmin-like protein 2
0,85	DLX3	distal-less homeobox 3
0,85	PHIP	pleckstrin homology domain interacting protein
0,85	CALCA	calcitonin-related polypeptide alpha
0,85	H2BFWT	H2B histone family, member W, testis-specific
0,85	KCNH5	potassium channel, voltage gated eag related subfamily H, member 5
0,85	ZNF704	zinc finger protein 704
0,85	FRMD1	FERM domain containing 1
0,85	MLYCD	malonyl-CoA decarboxylase
0,85	MAN1C1	mannosidase, alpha, class 1C, member 1
0,85	POM121L12	POM121 transmembrane nucleoporin-like 12
0,85	FBN1	fibrillin 1
0,85	ST6GAL2	ST6 beta-galactosamide alpha-2,6-sialyltranferase 2
0,85	MSRB2	methionine sulfoxide reductase B2
0,85	OSTCP1	oligosaccharyltransferase complex subunit pseudogene 1
0,85	BUD13	BUD13 homolog
0,85	NNMT	nicotinamide N-methyltransferase
0,85	TSPAN18	tetraspanin 18
0,85	KRTAP26-1	keratin associated protein 26-1
0,85	ADAMTSL4-AS1	ADAMTSL4 antisense RNA 1
0,85	AIFM2	apoptosis-inducing factor, mitochondrion-associated, 2
0,85	NF1	neurofibromin 1
0,85	ENDOD1	endonuclease domain containing 1
0,85	DUSP13	dual specificity phosphatase 13
0,85	STK32C	serine/threonine kinase 32C
0,85	ISL1	Transcript Identified by AceView, Entrez Gene ID(s) 3670
0,85	TTC28	tetratricopeptide repeat domain 28
0,85	DIRC2	disrupted in renal carcinoma 2
0,85	HRASLS2	HRAS-like suppressor 2
0,85	CDRT1	CMT1A duplicated region transcript 1
0,85	KLLN	killin, p53-regulated DNA replication inhibitor
0,85	ZNF391	zinc finger protein 391
0,85	CHODL	chondrolectin

0,85	SENP5	Transcript Identified by AceView, Entrez Gene ID(s) 205564
0,84	GREM1	gremlin 1, DAN family BMP antagonist [Source:HGNC Symbol;Acc:HGNC:2001]
0,84	SLC30A10	solute carrier family 30, member 10
0,84	NFKBIE	nuclear factor of kappa light polypeptide gene enhancer in B-cells inhibitor, epsilon
0,84	TRPV5	transient receptor potential cation channel, subfamily V, member 5
0,84	LUZP2	leucine zipper protein 2
0,84	BARD1	BRCA1 associated RING domain 1
0,84	FAM228B	family with sequence similarity 228, member B
0,84	IFI6	interferon, alpha-inducible protein 6
0,84	OR51L1	olfactory receptor, family 51, subfamily L, member 1
0,84	HAL	histidine ammonia-lyase
0,84	SCIN	scinderin
0,84	TMEM128	transmembrane protein 128
0,84	MYCBP2	MYC binding protein 2, E3 ubiquitin protein ligase
0,84	FAM26F	family with sequence similarity 26, member F
0,84	NR1H4	nuclear receptor subfamily 1, group H, member 4
0,84	ARHGEF10	Rho guanine nucleotide exchange factor 10
0,84	CACNG5	calcium channel, voltage-dependent, gamma subunit 5
0,84	DGKH	diacylglycerol kinase, eta
0,84	MAP3K8	mitogen-activated protein kinase kinase kinase 8
0,84	LOC100288748; RP11-22C11.2	uncharacterized LOC100288748; novel transcript, antisense to ESRP1
0,84	MYO1E	Transcript Identified by AceView, Entrez Gene ID(s) 4643
0,84	CFAP221	cilia and flagella associated protein 221
0,84	FAM149A	family with sequence similarity 149, member A
0,84	TIRAP	toll-interleukin 1 receptor (TIR) domain containing adaptor protein
0,84	BACH2	BTB and CNC homology 1, basic leucine zipper transcription factor 2
0,84	LRRC39	leucine rich repeat containing 39
0,84	BCL2L14	BCL2-like 14 (apoptosis facilitator)
0,84	ADAMTS8	ADAM metallopeptidase with thrombospondin type 1 motif 8
0,84	RAB6B	RAB6B, member RAS oncogene family
0,84	AMER2	APC membrane recruitment protein 2
0,84	CD274	CD274 molecule
0,84	SLC23A2	solute carrier family 23 (ascorbic acid transporter), member 2
0,84	TFRC	Transcript Identified by AceView, Entrez Gene ID(s) 7037
0,84	SNX22	sorting nexin 22
0,84	CYP3A4	cytochrome P450, family 3, subfamily A, polypeptide 4
0,84	TMEM151A	transmembrane protein 151A
0,84	HDAC5	histone deacetylase 5

0,84	TMEM8B	transmembrane protein 8B
0,84	TRIM22	tripartite motif containing 22
0,84	RAB36	RAB36, member RAS oncogene family
0,84	TNIP3	TNFAIP3 interacting protein 3
0,84	GABRR3	gamma-aminobutyric acid (GABA) A receptor, rho 3 (gene/pseudogene)
0,84	GPR143	G protein-coupled receptor 143
0,84	CNTNAP1	contactin associated protein 1
0,84	OCRL	oculocerebrorenal syndrome of Lowe
0,84	FAM216A	family with sequence similarity 216, member A
0,84	REPIN1	replication initiator 1
0,84	PELI1	pellino E3 ubiquitin protein ligase 1
0,84	GBP1	guanylate binding protein 1, interferon-inducible
0,84	PCSK1	proprotein convertase subtilisin/kexin type 1
0,84	KCTD7	potassium channel tetramerization domain containing 7
0,84	RAD21	Transcript Identified by AceView, Entrez Gene ID(s) 5885
0,84	GPX6	glutathione peroxidase 6
0,84	LGSN	lengsin, lens protein with glutamine synthetase domain
0,84	LRRC8E	leucine rich repeat containing 8 family, member E
0,84	PSTPIP2	proline-serine-threonine phosphatase interacting protein 2
0,84	FYCO1	FYVE and coiled-coil domain containing 1
0,84	CCDC15	coiled-coil domain containing 15
0,84	GPR161	G protein-coupled receptor 161
0,84	CEBPD	CCAAT/enhancer binding protein (C/EBP), delta
0,84	TTC23L	tetratricopeptide repeat domain 23-like
0,84	SAP25	Sin3A associated protein 25kDa
0,84	C8orf86	chromosome 8 open reading frame 86
0,84	TPO	thyroid peroxidase
0,84	COX4I1	cytochrome c oxidase subunit IV isoform 1
0,84	WDPCP	WD repeat containing planar cell polarity effector
0,84	RASL11B	RAS-like, family 11, member B
0,84	TMEM82	transmembrane protein 82
0,84	C3orf67	chromosome 3 open reading frame 67
0,84	PDS5B	Memczak2013 ALT_ACCEPTOR, ALT_DONOR, coding, INTERNAL, intronic best transcript NM_015032
0,84	ZDHHC3	zinc finger, DHHC-type containing 3
0,84	BACH2	Memczak2013 ANTISENSE, coding, INTERNAL, intronic best transcript NM_001170794
0,84	MRPL23; LOC102724828; AC004556.1	Homo sapiens mitochondrial ribosomal protein L23 (MRPL23), mRNA.; 39S ribosomal protein L23, mitochondrial-like; Homo sapiens mitochondrial ribosomal protein L23, mRNA (cDNA clone MGC:12795 IMAGE:4298000), complete cds.
0,84	NOTCH3	notch 3
0,84	ADPRHL2	ADP-ribosylhydrolase like 2

0,84	CSPG4	chondroitin sulfate proteoglycan 4
0,84	CRABP2	cellular retinoic acid binding protein 2
0,84	NEURL1B	neuralized E3 ubiquitin protein ligase 1B
0,84	HGD	homogentisate 1,2-dioxygenase
0,84	FAHD2A	fumarylacetoacetate hydrolase domain containing 2A
0,84	NR2F1	nuclear receptor subfamily 2, group F, member 1
0,84	TGS1	trimethylguanosine synthase 1
0,84	PCDHA9; PCDHAC1; PCDHAC2; PCDHA1; PCDHA10; PCDHA11; PCDHA12; PCDHA13; PCDHA2; PCDHA3; PCDHA4; PCDHA5; PCDHA6; PCDHA7; PCDHA8; PCDHA14	protocadherin alpha 9; protocadherin alpha subfamily C, 1; protocadherin alpha subfamily C, 2; protocadherin alpha 1; protocadherin alpha 10; protocadherin alpha 11; protocadherin alpha 12; protocadherin alpha 13; protocadherin alpha 2; protocadherin alpha 3; protocadherin alpha 4; protocadherin alpha 5; protocadherin alpha 6; protocadherin alpha 7; protocadherin alpha 8; protocadherin alpha 14 pseudogene
0,84	GRIN2A	Transcript Identified by AceView, Entrez Gene ID(s) 2903
0,84	AS3MT	arsenite methyltransferase
0,84	EXT1; hunera	Jeck2013 ALT_ACCEPTOR, ALT_DONOR, coding, INTERNAL, intronic best transcript NM_000127; Transcript Identified by AceView
0,84	MLXIP	Memczak2013 ALT_ACCEPTOR, ALT_DONOR, coding, INTERNAL, intronic best transcript NM_014938
0,84	ETNK2	ethanolamine kinase 2
0,84	MYLK2	myosin light chain kinase 2
0,84	OSBP	oxysterol binding protein
0,84	CRYBG3	crystallin beta-gamma domain containing 3
0,84	MBOAT4	membrane bound O-acyltransferase domain containing 4
0,84	CNGA1	cyclic nucleotide gated channel alpha 1
0,84	H1FX	H1 histone family, member X
0,84	DSG4	desmoglein 4
0,84	HDAC10	histone deacetylase 10
0,84	GGACT	gamma-glutamylamine cyclotransferase
0,84	PADI3	peptidyl arginine deiminase, type III
0,84	HEATR5A	HEAT repeat containing 5A
0,84	HDAC11	histone deacetylase 11
0,84	DNAJC12	DnaJ (Hsp40) homolog, subfamily C, member 12
0,84	SERPINB4	serpin peptidase inhibitor, clade B (ovalbumin), member 4
0,84	ZNF711	zinc finger protein 711
0,84	KIFC2	kinesin family member C2

0,84	SNX30	sorting nexin family member 30
0,84	CD14	CD14 molecule
0,84	NMRK1	nicotinamide riboside kinase 1
0,84	CD300A	CD300a molecule
0,84	SNX5	Zhang2013 ALT_ACCEPTOR, ALT_DONOR, coding, INTERNAL, intronic best transcript NM_152227
0,84	ZNF627	Memczak2013 ANTISENSE, CDS, coding, INTERNAL best transcript NM_145295
0,84	BRINP1	bone morphogenetic protein/retinoic acid inducible neural-specific 1
0,84	PPP1R21	protein phosphatase 1, regulatory subunit 21
0,84	FLNA	Jeck2013 ANTISENSE, CDS, coding, INTERNAL, OVCODE, OVEXON best transcript NM_001110556
0,84	FAM166A	family with sequence similarity 166, member A
0,84	OR4C16	olfactory receptor, family 4, subfamily C, member 16 (gene/pseudogene)
0,84	CFLAR	CASP8 and FADD like apoptosis regulator
0,84	MTL5	metallothionein-like 5, testis-specific (tesmin)
0,84	RAPGEFL1	Rap guanine nucleotide exchange factor like 1
0,84	SUCLG2	succinate-CoA ligase, GDP-forming, beta subunit
0,84	PSMB9	proteasome subunit beta 9
0,84	SLC35E4	solute carrier family 35, member E4
0,84	GALE	UDP-galactose-4-epimerase
0,84	ABCC11	ATP binding cassette subfamily C member 11
0,84	FAM229A	family with sequence similarity 229, member A
0,84	ATP8B1	ATPase, aminophospholipid transporter, class I, type 8B, member 1
0,84	TMEM51	transmembrane protein 51
0,84	WDR64	WD repeat domain 64
0,84	PPP1R9B	protein phosphatase 1, regulatory subunit 9B
0,84	PTPRB	protein tyrosine phosphatase, receptor type, B
0,84	TESC	tescalcin
0,84	MTRNR2L10	MT-RNR2-like 10
0,84	TSPEAR	thrombospondin-type laminin G domain and EAR repeats
0,84	PFKFB2	6-phosphofructo-2-kinase/fructose-2,6-biphosphatase 2
0,84	ZNF841	zinc finger protein 841
0,84	MINK1	misshapen-like kinase 1
0,84	OR14C36	olfactory receptor, family 14, subfamily C, member 36
0,84	ZNF546	zinc finger protein 546
0,84	CDY1	chromodomain protein, Y-linked, 1
0,84	MVB12B	multivesicular body subunit 12B
0,84	OR6J1	olfactory receptor, family 6, subfamily J, member 1 (gene/pseudogene)
0,84	ST6GALNAC2	ST6 (alpha-N-acetyl-neuraminyl-2,3-beta-galactosyl-1,3)-N-acetylgalactosaminide alpha-2,6-sialyltransferase 2
0,84	C1orf68	chromosome 1 open reading frame 68

0,84	ACYP1	acylphosphatase 1, erythrocyte (common) type
0,84	POGZ	Transcript Identified by AceView, Entrez Gene ID(s) 23126
0,84	TMEM184A	transmembrane protein 184A
0,84	C15orf59	chromosome 15 open reading frame 59
0,84	SOWAHB	sosondowah ankyrin repeat domain family member B
0,84	SPRR3	small proline-rich protein 3
0,84	EBLN2	endogenous Bornavirus-like nucleoprotein 2
0,84	ZNF707	zinc finger protein 707
0,84	ACSS3	acyl-CoA synthetase short-chain family member 3
0,84	SUN3	Sad1 and UNC84 domain containing 3
0,84	RPS6KA1	ribosomal protein S6 kinase, 90kDa, polypeptide 1
0,84	LOC100133315; RP11-849H4.2	transient receptor potential cation channel, subfamily C, member 2-like; Salzman2013 ANNOTATED, INTERNAL, ncRNA, OVEXON best transcript NR_029192; Transcript Identified by AceView, Entrez Gene ID(s) 100133315, RefSeq ID(s) NR_029192; Transcript Identified by AceView, Entrez Gene ID(s) 100133315; Putative short transient receptor potential channel 2-like protein [Source:UniProtKB/Swiss-Prot;Acc:Q6ZNB5]
0,84	P3H3	prolyl 3-hydroxylase 3
0,84	LRFN3	leucine rich repeat and fibronectin type III domain containing 3
0,84	AGRN	agrin
0,84	ELMSAN1	ELM2 and Myb/SANT-like domain containing 1
0,84	GJC2	gap junction protein gamma 2
0,84	SCGB2A2	secretoglobin, family 2A, member 2
0,84	ARHGEF15	Rho guanine nucleotide exchange factor 15
0,84	NOS3	nitric oxide synthase 3 (endothelial cell)
0,84	LRBA	LPS-responsive vesicle trafficking, beach and anchor containing
0,84	MMD2	monocyte to macrophage differentiation-associated 2
0,84	DNMT3B	DNA (cytosine-5-)-methyltransferase 3 beta
0,84	CHGA	chromogranin A
0,84	LRRC30	leucine rich repeat containing 30
0,84	CDC34	cell division cycle 34
0,84	MAN2A2	mannosidase, alpha, class 2A, member 2
0,84	SERPINC1	serpin peptidase inhibitor, clade C (antithrombin), member 1
0,84	C1R	complement component 1, r subcomponent
0,84	LINC00525	long intergenic non-protein coding RNA 525
0,84	MS4A6E	membrane-spanning 4-domains, subfamily A, member 6E
0,84	TRAPPC4	trafficking protein particle complex 4
0,84	MAGI1	membrane associated guanylate kinase, WW and PDZ domain containing 1
0,84	DGKD	diacylglycerol kinase, delta 130kDa
0,83	KIAA1683	KIAA1683
0,83	TRIO	trio Rho guanine nucleotide exchange factor
0,83	TMEM63B	transmembrane protein 63B

0,83	HFE2	hemochromatosis type 2 (juvenile)
0,83	XCL2	chemokine (C motif) ligand 2
0,83	PRR25	proline rich 25
0,83	FSHB	follicle stimulating hormone, beta polypeptide
0,83	NTHL1	nth-like DNA glycosylase 1
0,83	WFDC1	WAP four-disulfide core domain 1
0,83	GMPR	guanosine monophosphate reductase
0,83	GXYLT1	glucoside xylosyltransferase 1
0,83	C2orf80	chromosome 2 open reading frame 80
0,83	MUSTN1	musculoskeletal, embryonic nuclear protein 1
0,83	HYOU1	hypoxia up-regulated 1
0,83	EXPH5	exophilin 5
0,83	TEX264	testis expressed 264
0,83	ITGA10	integrin alpha 10
0,83	FRRS1	ferric-chelate reductase 1
0,83	KDM8	lysine (K)-specific demethylase 8
0,83	TPPP3	tubulin polymerization-promoting protein family member 3
0,83	FBN2	fibrillin 2
0,83	RPL39L	ribosomal protein L39-like
0,83	OR11H7	olfactory receptor, family 11, subfamily H, member 7 (gene/pseudogene)
0,83	ZSWIM2	zinc finger, SWIM-type containing 2
0,83	LCAT	lecithin-cholesterol acyltransferase
0,83	NPR1	natriuretic peptide receptor 1
0,83	VSTM2B	V-set and transmembrane domain containing 2B
0,83	AP2A1	adaptor-related protein complex 2, alpha 1 subunit
0,83	LRRTM3	leucine rich repeat transmembrane neuronal 3
0,83	BCAM	basal cell adhesion molecule (Lutheran blood group)
0,83	SYT17	synaptotagmin XVII
0,83	ZBTB12	zinc finger and BTB domain containing 12
0,83	MAST4	microtubule associated serine/threonine kinase family member 4
0,83	STS	steroid sulfatase (microsomal), isozyme S
0,83	CCDC130	coiled-coil domain containing 130
0,83	NATD1	N-acetyltransferase domain containing 1
0,83	GSTZ1	glutathione S-transferase zeta 1
0,83	IFT172	intraflagellar transport 172
0,83	TSPAN11	tetraspanin 11
0,83	GPHA2	glycoprotein hormone alpha 2
0,83	MID2	midline 2
0,83	VPS8	Transcript Identified by AceView, Entrez Gene ID(s) 23355
0,83	SHROOM1	shroom family member 1
0,83	EML1	echinoderm microtubule associated protein like 1
0,83	KDM3A	lysine (K)-specific demethylase 3A
0,83	KLK12	kallikrein related peptidase 12
0,83	CA9	carbonic anhydrase IX
0,83	BRF2	BRF2, RNA polymerase III transcription initiation factor 50 kDa subunit

0,83	SNAP25	synaptosome associated protein 25kDa
0,83	PRSS22	protease, serine, 22
0,83	SSTR3	somatostatin receptor 3
0,83	C10orf11	chromosome 10 open reading frame 11
0,83	DNAJB8	DnaJ (Hsp40) homolog, subfamily B, member 8
0,83	HIVEP2	human immunodeficiency virus type I enhancer binding protein 2
0,83	SNX20	sorting nexin 20
0,83	FAM120B	family with sequence similarity 120B
0,83	GABRA1	gamma-aminobutyric acid (GABA) A receptor, alpha 1
0,83	C19orf68	chromosome 19 open reading frame 68
0,83	LYG1	lysozyme G-like 1
0,83	CPN2	carboxypeptidase N, polypeptide 2
0,83	ZNF517	zinc finger protein 517
0,83	ASCC1	Transcript Identified by AceView, Entrez Gene ID(s) 51008
0,83	DOPEY1	dopey family member 1
0,83	TTBK2	tau tubulin kinase 2
0,83	RAMP3	receptor (G protein-coupled) activity modifying protein 3
0,83	ARHGAP39	Rho GTPase activating protein 39
0,83	PLXNB3	plexin B3
0,83	UBE3B	Transcript Identified by AceView, Entrez Gene ID(s) 89910
0,83	OBFC1	oligonucleotide/oligosaccharide-binding fold containing 1
0,83	GPR162	G protein-coupled receptor 162
0,83	LBH	limb bud and heart development
0,83	CYP17A1	cytochrome P450, family 17, subfamily A, polypeptide 1
0,83	ALDOC	aldolase C, fructose-bisphosphate
0,83	FABP1	fatty acid binding protein 1, liver
0,83	TRAPPC10	trafficking protein particle complex 10
0,83	IMMP1L	inner mitochondrial membrane peptidase subunit 1
0,83	LRRC55	leucine rich repeat containing 55
0,83	CBR3	carbonyl reductase 3
0,83	PRDM16	PR domain containing 16
0,83	SH2D4A	SH2 domain containing 4A
0,83	OR4K17	olfactory receptor, family 4, subfamily K, member 17
0,83	ZNF804A	zinc finger protein 804A
0,83	CNR2	cannabinoid receptor 2
0,83	CR2	complement component (3d/Epstein Barr virus) receptor 2
0,83	CACFD1	calcium channel flower domain containing 1
0,83	MARK1	MAP/microtubule affinity-regulating kinase 1
0,83	KCNU1	potassium channel, subfamily U, member 1
0,83	EFHB	EF-hand domain family, member B
0,83	SLC25A30	solute carrier family 25, member 30
0,83	DCAF4L2	DDB1 and CUL4 associated factor 4-like 2

0,83	PLPPR4	phospholipid phosphatase related 4
0,83	PRSS16	protease, serine, 16 (thymus)
0,83	SDCBP2	syndecan binding protein (syntenin) 2
0,83	ZSCAN31	zinc finger and SCAN domain containing 31
0,83	OR2A7; ARHGEF34P	olfactory receptor, family 2, subfamily A, member 7; Rho guanine nucleotide exchange factor 34, pseudogene
0,83	IFNK	interferon, kappa
0,83	TSSK3	testis-specific serine kinase 3
0,83	TBR1	T-box, brain, 1
0,83	WBSCR27	Williams Beuren syndrome chromosome region 27
0,83	MRPL54	mitochondrial ribosomal protein L54
0,83	HSP90B1	Transcript Identified by AceView, Entrez Gene ID(s) 7184
0,83	BAIAP2L2	BAI1-associated protein 2-like 2
0,83	OFD1	oral-facial-digital syndrome 1
0,83	PAPLN	papilin, proteoglycan-like sulfated glycoprotein
0,83	LRP6	LDL receptor related protein 6
0,83	ST14	suppression of tumorigenicity 14 (colon carcinoma)
0,83	COL2A1	collagen, type II, alpha 1
0,83	TOR4A	torsin family 4, member A
0,83	CD44	Memczak2013 ALT_ACCEPTOR, ALT_DONOR, coding, INTERNAL, intronic best transcript NM_001202557
0,83	PREX2	phosphatidylinositol-3,4,5-trisphosphate-dependent Rac exchange factor 2
0,83	MUC6	mucin 6, oligomeric mucus/gel-forming
0,83	ESYT3	extended synaptotagmin-like protein 3
0,83	SH2D3C	SH2 domain containing 3C
0,83	SPAG11B	sperm associated antigen 11B
0,83	MAN2B1	mannosidase, alpha, class 2B, member 1
0,83	C16orf96	chromosome 16 open reading frame 96
0,83	ZNF605	zinc finger protein 605
0,83	ECEL1	endothelin converting enzyme-like 1
0,83	MYH1	myosin, heavy chain 1, skeletal muscle, adult
0,83	KCTD12	potassium channel tetramerization domain containing 12
0,83	ZZZ3	Transcript Identified by AceView, Entrez Gene ID(s) 26009
0,83	PARD3B	par-3 family cell polarity regulator beta
0,83	KCNIP3	Kv channel interacting protein 3, calsenilin
0,83	IL37	interleukin 37
0,83	HMGXB3	HMG box domain containing 3
0,83	FAR2P1	fatty acyl-CoA reductase 2 pseudogene 1
0,83	ZNF275	zinc finger protein 275
0,83	ADAM2	ADAM metallopeptidase domain 2
0,83	ATM	ATM serine/threonine kinase
0,83	EXT1; spawla	Jeck2013 ALT_ACCEPTOR, ALT_DONOR, coding, INTERNAL, intronic best transcript NM_000127; Transcript Identified by AceView

0,83	PLD1	phospholipase D1, phosphatidylcholine-specific
0,83	DRD1	dopamine receptor D1
0,83	ZNF324	zinc finger protein 324
0,83	GFY	golgi-associated, olfactory signaling regulator
0,83	HMBS	hydroxymethylbilane synthase
0,83	ITPR1	inositol 1,4,5-trisphosphate receptor, type 1
0,83	CYP2U1	cytochrome P450, family 2, subfamily U, polypeptide 1
0,83	LGI4	leucine-rich repeat LGI family, member 4
0,83	TRIM39	tripartite motif containing 39
0,83	MTMR7	myotubularin related protein 7
0,83	RDH16	retinol dehydrogenase 16 (all-trans)
0,83	FBXO8	F-box protein 8
0,83	LCOR	ligand dependent nuclear receptor corepressor
0,83	LRP1B	LDL receptor related protein 1B
0,83	SLC35F4	solute carrier family 35, member F4
0,83	PSD3	pleckstrin and Sec7 domain containing 3
0,83	CHST6	carbohydrate (N-acetylglucosamine 6-O) sulfotransferase 6
0,83	EPB42	erythrocyte membrane protein band 4.2
0,83	SLC7A10	solute carrier family 7 (neutral amino acid transporter light chain, asc system), member 10
0,83	MIR7162; GOLGA2P6; MTPAP	microRNA 7162; golgin A2 pseudogene 6; mitochondrial poly(A) polymerase
0,83	CYP26B1	cytochrome P450, family 26, subfamily B, polypeptide 1
0,83	CACNG4	calcium channel, voltage-dependent, gamma subunit 4
0,83	CACNA1E	calcium channel, voltage-dependent, R type, alpha 1E subunit
0,83	SYNPR	synaptoporin
0,83	IGFBP5	insulin like growth factor binding protein 5
0,83	DRD4	dopamine receptor D4
0,83	GLI2	Jeck2013 ALT_ACCEPTOR, ALT_DONOR, coding, INTERNAL, intronic best transcript NM_005270
0,83	PIEZO2	piezo-type mechanosensitive ion channel component 2
0,83	IZUMO4	IZUMO family member 4
0,83	PTK7	protein tyrosine kinase 7 (inactive)
0,83	TXNRD3	thioredoxin reductase 3
0,83	TM4SF4	transmembrane 4 L six family member 4
0,83	C10orf131	chromosome 10 open reading frame 131
0,83	PHF24	PHD finger protein 24
0,83	CYFIP2	cytoplasmic FMR1 interacting protein 2
0,83	C9orf147; HSDL2	Transcript Identified by AceView, Entrez Gene ID(s) 100133204; Salzman2013 ANTISENSE, CDS, coding, downstream_end, intronic, OVCODE, OVERLAPTX, OVEXON, UTR3 best transcript NM_032303; chromosome 9 open reading frame 147; chromosome 9 open reading frame 147 [Source:HGNC Symbol;Acc:HGNC:31438]

0,83	SLC22A9	solute carrier family 22 (organic anion transporter), member 9
0,83	PLEKHA5	pleckstrin homology domain containing, family A member 5
0,83	PRB1	proline-rich protein BstNI subfamily 1
0,83	AGA	aspartylglucosaminidase
0,83	FAM215A	family with sequence similarity 215, member A (non-protein coding)
0,83	KCNG3	potassium channel, voltage gated modifier subfamily G, member 3
0,83	RTN1	reticulon 1
0,83	QRICH1	Transcript Identified by AceView, Entrez Gene ID(s) 54870
0,83	ITPKA	inositol-trisphosphate 3-kinase A
0,83	IPP	intracisternal A particle-promoted polypeptide
0,82	OR5M1	olfactory receptor, family 5, subfamily M, member 1
0,82	CPS1	carbamoyl-phosphate synthase 1
0,82	PADI4	peptidyl arginine deiminase, type IV
0,82	DENND4C	DENN/MADD domain containing 4C
0,82	PRAMEF19	PRAME family member 19 [Source:EntrezGene;Acc:645414]
0,82	PKP2	plakophilin 2
0,82	DOT1L	DOT1-like histone H3K79 methyltransferase
0,82	HCN2	hyperpolarization activated cyclic nucleotide gated potassium channel 2
0,82	CYB561D2	cytochrome b561 family, member D2
0,82	MYBPC2	myosin binding protein C, fast type
0,82	NDUFA10	NADH dehydrogenase (ubiquinone) 1 alpha subcomplex, 10, 42kDa
0,82	EFCAB9	EF-hand calcium binding domain 9
0,82	BMP10	bone morphogenetic protein 10
0,82	PDE3B	phosphodiesterase 3B, cGMP-inhibited
0,82	TMEM200C	transmembrane protein 200C
0,82	POLR2A	polymerase (RNA) II (DNA directed) polypeptide A, 220kDa
0,82	LRCOL1	leucine rich colipase-like 1
0,82	AKAP11	A kinase (PRKA) anchor protein 11
0,82	ZNF682	zinc finger protein 682
0,82	ZDHHC19	zinc finger, DHHC-type containing 19
0,82	PALMD	palmdelphin
0,82	PROM1	prominin 1
0,82	ADRA2A	adrenoceptor alpha 2A
0,82	SAG	S-antigen; retina and pineal gland (arrestin)
0,82	ZCCHC11	zinc finger, CCHC domain containing 11
0,82	EPPK1	epiplakin 1
0,82	PNPLA7	Transcript Identified by AceView, Entrez Gene ID(s) 375775
0,82	RGS17	regulator of G-protein signaling 17
0,82	MOXD1	monooxygenase, DBH-like 1
0,82	MMP11	matrix metallopeptidase 11

0,82	ADAM23	ADAM metallopeptidase domain 23
0,82	GINS1	GINS complex subunit 1 (Psf1 homolog)
0,82	NUP88	nucleoporin 88kDa
0,82	MGC27345	uncharacterized protein MGC27345
0,82	OR4F15	olfactory receptor, family 4, subfamily F, member 15
0,82	GCC2	Jeck2013 ANTISENSE, CDS, coding, INTERNAL, intronic, OVCODE, OVEXON best transcript NM_181453
0,82	BDP1	B double prime 1, subunit of RNA polymerase III transcription initiation factor IIIB
0,82	KAT6B	K(lysine) acetyltransferase 6B
0,82	CETN3	centrin 3
0,82	NTF4	neurotrophin 4
0,82	ZCCHC24	zinc finger, CCHC domain containing 24
0,82	DCAF10	DDB1 and CUL4 associated factor 10
0,82	LGALS16	lectin, galactoside-binding, soluble, 16
0,82	OR52A1	olfactory receptor, family 52, subfamily A, member 1
0,82	ZNF446	zinc finger protein 446
0,82	ZNF846	zinc finger protein 846
0,82	CCDC157	coiled-coil domain containing 157
0,82	SLC22A6	solute carrier family 22 (organic anion transporter), member 6
0,82	KIF16B	kinesin family member 16B
0,82	FES	FES proto-oncogene, tyrosine kinase
0,82	GOLGB1	golgin B1
0,82	OR5AR1	olfactory receptor, family 5, subfamily AR, member 1 (gene/pseudogene)
0,82	OOSP2	oocyte secreted protein 2
0,82	PCNT	pericentrin
0,82	SGSM3	small G protein signaling modulator 3
0,82	CDKL5	cyclin-dependent kinase-like 5
0,82	TRRAP	transformation/transcription domain-associated protein
0,82	ANKRD52	ankyrin repeat domain 52
0,82	MSC	musculin
0,82	TCF20	transcription factor 20 (AR1)
0,82	SLCO1C1	solute carrier organic anion transporter family, member 1C1
0,82	FAM151B	Memczak2013 ANTISENSE, coding, INTERNAL, intronic best transcript NM_205548
0,82	RFTN2	raftlin family member 2
0,82	PKIB	protein kinase (cAMP-dependent, catalytic) inhibitor beta
0,82	FAHD2B	fumarylacetoacetate hydrolase domain containing 2B
0,82	CHGB	chromogranin B
0,82	C4orf22	chromosome 4 open reading frame 22
0,82	SURF1	surfeit 1
0,82	AKAP12	A kinase (PRKA) anchor protein 12
0,82	ACSS2	acyl-CoA synthetase short-chain family member 2

0,82	PREX1	phosphatidylinositol-3,4,5-trisphosphate-dependent Rac exchange factor 1
0,82	IFNA1	interferon, alpha 1
0,82	DKK1	Jeck2013 ANTISENSE, CDS, coding, INTERNAL, intronic, OVCODE, OVEXON best transcript NM_012242
0,82	TMEM210	transmembrane protein 210
0,82	COL8A1	collagen, type VIII, alpha 1
0,82	IKBKG	inhibitor of kappa light polypeptide gene enhancer in B-cells, kinase gamma
0,82	GNMT	glycine N-methyltransferase
0,82	PROKR1	prokineticin receptor 1
0,82	NR4A3	nuclear receptor subfamily 4, group A, member 3
0,82	LCE6A	late cornified envelope 6A
0,82	CFTR	cystic fibrosis transmembrane conductance regulator
0,82	LARGE	Transcript Identified by AceView, Entrez Gene ID(s) 9215
0,82	KRT77	keratin 77, type II
0,82	CPED1	cadherin-like and PC-esterase domain containing 1
0,82	GATM	glycine amidinotransferase (L-arginine:glycine amidinotransferase)
0,82	NCOR2	nuclear receptor corepressor 2
0,82	CUEDC2	CUE domain containing 2
0,82	GH2	growth hormone 2
0,82	PBX4	pre-B-cell leukemia homeobox 4
0,82	OPCML	opioid binding protein/cell adhesion molecule-like
0,82	PADI3	peptidyl arginine deiminase, type III
0,82	ABCC8	ATP binding cassette subfamily C member 8
0,82	SUSD6	sushi domain containing 6
0,82	CHD3	chromodomain helicase DNA binding protein 3
0,82	AIM1L	absent in melanoma 1-like
0,82	BBS2	Bardet-Biedl syndrome 2
0,82	SLX4	SLX4 structure-specific endonuclease subunit
0,82	GLI3	GLI family zinc finger 3
0,82	ICAM2	intercellular adhesion molecule 2
0,82	FAM167A	family with sequence similarity 167, member A
0,82	PCDHB15	protocadherin beta 15
0,82	HBQ1	hemoglobin, theta 1
0,82	C9orf92	chromosome 9 open reading frame 92
0,82	INPP5D	inositol polyphosphate-5-phosphatase D
0,82	MAGED4B; MAGED4; SNORA11D	melanoma antigen family D4B; melanoma antigen family D4; small nucleolar RNA, H/ACA box 11D
0,82	HOXD4	homeobox D4
0,82	TSPAN5	tetraspanin 5
0,82	ZNF483	zinc finger protein 483
0,82	OCLN	occludin
0,82	GUCY1A3	guanylate cyclase 1, soluble, alpha 3
0,82	SETBP1	SET binding protein 1
0,82	GPX7	glutathione peroxidase 7

0,82	HIST1H4A	histone cluster 1, H4a
0,82	BET1L	Bet1 golgi vesicular membrane trafficking protein-like
0,82	BAZ2B	bromodomain adjacent to zinc finger domain 2B
0,82	CST5	cystatin D
0,82	SPP1	secreted phosphoprotein 1
0,82	RAG1	recombination activating gene 1
0,82	SPNS2	spinster homolog 2 (Drosophila)
0,81	GOLGA8K; ULK4P1	golgin A8 family, member K; ULK4 pseudogene 1
0,81	SP4	Sp4 transcription factor
0,81	RGS12	regulator of G-protein signaling 12
0,81	TMEM72	transmembrane protein 72
0,81	CHST7	carbohydrate (N-acetylglucosamine 6-O) sulfotransferase 7
0,81	KRT33B	keratin 33B, type I
0,81	FBN3; mumari	Zhang2013 ALT_ACCEPTOR, ALT_DONOR, coding, INTERNAL, intronic best transcript NM_032447; Transcript Identified by AceView
0,81	WDR25	WD repeat domain 25
0,81	FBXW9	F-box and WD repeat domain containing 9
0,81	PGM2L1	phosphoglucomutase 2-like 1
0,81	ALMS1	Alstrom syndrome protein 1
0,81	AKAP9	A kinase (PRKA) anchor protein 9
0,81	CUX1	cut-like homeobox 1
0,81	GXYLT2	glucoside xylosyltransferase 2
0,81	MAGEE1	MAGE family member E1
0,81	ALPI	alkaline phosphatase, intestinal
0,81	SPANXN4	SPANX family, member N4
0,81	TMEM2	transmembrane protein 2
0,81	AHNAK	AHNAK nucleoprotein
0,81	SDR9C7	short chain dehydrogenase/reductase family 9C, member 7
0,81	PLXNB2	plexin B2
0,81	CREB3L4	cAMP responsive element binding protein 3-like 4
0,81	PROL1	proline rich, lacrimal 1
0,81	LRP1	LDL receptor related protein 1
0,81	ZNF780A	zinc finger protein 780A
0,81	CD19	CD19 molecule
0,81	GBX2	gastrulation brain homeobox 2
0,81	FLNA	filamin A, alpha
0,81	PDIA5; MIR7110	protein disulfide isomerase family A, member 5; microRNA 7110
0,81	OLFM1	olfactomedin 1
0,81	TDRD12	tudor domain containing 12
0,81	UBA7; MIR5193	ubiquitin-like modifier activating enzyme 7; microRNA 5193
0,81	RNF19A	ring finger protein 19A, RBR E3 ubiquitin protein ligase
0,81	FRRS1L	ferric-chelate reductase 1-like

0,81	ARIH2OS	ariadne homolog 2 opposite strand
0,81	NDUFA10	NADH dehydrogenase (ubiquinone) 1 alpha subcomplex, 10, 42kDa
0,81	ERVV-2	endogenous retrovirus group V, member 2
0,81	JAK1	Janus kinase 1
0,81	DNA2	DNA replication helicase/nuclease 2
0,81	CCL14; CCL15; CCL15-CCL14	chemokine (C-C motif) ligand 14; chemokine (C-C motif) ligand 15; CCL15-CCL14 readthrough (NMD candidate)
0,81	C10orf12	chromosome 10 open reading frame 12
0,81	SEMA4A	sema domain, immunoglobulin domain (Ig), transmembrane domain (TM) and short cytoplasmic domain, (semaphorin) 4A
0,81	PLAC4	placenta specific 4
0,81	NDEL1	nudE neurodevelopment protein 1-like 1
0,81	PHLPP2	PH domain and leucine rich repeat protein phosphatase 2
0,81	CCDC84	coiled-coil domain containing 84
0,81	TAF11	TAF11 RNA polymerase II, TATA box binding protein (TBP)-associated factor, 28kDa
0,81	SAT1	spermidine/spermine N1-acetyltransferase 1
0,81	GNAO1	guanine nucleotide binding protein (G protein), alpha activating activity polypeptide O
0,81	PRKDC	protein kinase, DNA-activated, catalytic polypeptide
0,81	ZFYVE1	zinc finger, FYVE domain containing 1
0,81	CLEC5A	C-type lectin domain family 5, member A
0,81	SPAG1	sperm associated antigen 1
0,81	ZNF165	zinc finger protein 165
0,81	HYKK	hydroxylysine kinase
0,81	BIRC6	baculoviral IAP repeat containing 6
0,81	GRIN2D	glutamate receptor, ionotropic, N-methyl D-aspartate 2D
0,81	ZC3H12B	zinc finger CCCH-type containing 12B
0,81	PMEL	premelanosome protein
0,81	SLC18A2	solute carrier family 18 (vesicular monoamine transporter), member 2
0,81	ZNF354C	zinc finger protein 354C
0,81	NBAS	neuroblastoma amplified sequence
0,81	ROBO2	roundabout guidance receptor 2
0,81	ZKSCAN7	zinc finger with KRAB and SCAN domains 7
0,81	DACH1	Memczak2013 ANTISENSE, coding, INTERNAL, intronic best transcript NM_080760
0,81	DNAJC5B	DnaJ (Hsp40) homolog, subfamily C, member 5 beta
0,81	PTPDC1	protein tyrosine phosphatase domain containing 1
0,81	HSD3B2	hydroxy-delta-5-steroid dehydrogenase, 3 beta- and steroid delta-isomerase 2
0,81	TSPAN17	tetraspanin 17
0,81	PRKAR1B	protein kinase, cAMP-dependent, regulatory, type I, beta
0,81	DPY19L3	dpy-19-like 3 (C. elegans)
0,81	CBLC	Cbl proto-oncogene C, E3 ubiquitin protein ligase

0,81	SPRR2A	small proline-rich protein 2A
0,81	CD83	CD83 molecule
0,81	MIER2	Zhang2013 ALT_ACCEPTOR, ALT_DONOR, coding, INTERNAL, intronic, OVERLAPTX best transcript NM_017550
0,81	INPP5A	Transcript Identified by AceView, Entrez Gene ID(s) 3632
0,81	OGFOD2	2-oxoglutarate and iron-dependent oxygenase domain containing 2
0,81	OGFRL1	opioid growth factor receptor-like 1
0,81	EDEM1	Transcript Identified by AceView, Entrez Gene ID(s) 9695
0,81	SLC9A4	solute carrier family 9, subfamily A (NHE4, cation proton antiporter 4), member 4
0,81	KCNS2	potassium voltage-gated channel, modifier subfamily S, member 2
0,81	SLC6A14	solute carrier family 6 (amino acid transporter), member 14
0,81	ALOX5	Transcript Identified by AceView, Entrez Gene ID(s) 240
0,81	AIP	aryl hydrocarbon receptor interacting protein
0,81	ACADS	acyl-CoA dehydrogenase, C-2 to C-3 short chain
0,81	LRP6	LDL receptor related protein 6
0,81	STEAP2	STEAP family member 2, metalloreductase
0,81	HIPK3	homeodomain interacting protein kinase 3
0,81	MAD2L2	MAD2 mitotic arrest deficient-like 2 (yeast)
0,81	MAP2K1	Transcript Identified by AceView, Entrez Gene ID(s) 5604
0,81	C20orf85	chromosome 20 open reading frame 85
0,81	B3GALT4	UDP-Gal:betaGlcNAc beta 1,3-galactosyltransferase 4
0,81	KCNV2	potassium channel, voltage gated modifier subfamily V, member 2
0,81	DLG2	discs, large homolog 2 (Drosophila)
0,81	SLC12A7	solute carrier family 12 (potassium/chloride transporter), member 7
0,81	KIAA1644	KIAA1644
0,81	PLVAP	plasmalemma vesicle associated protein
0,81	REV3L	REV3 like, DNA directed polymerase zeta catalytic subunit
0,81	OTUD7B	OTU deubiquitinase 7B
0,81	KAT6A	K(lysine) acetyltransferase 6A
0,81	ERBB4	Transcript Identified by AceView, Entrez Gene ID(s) 2066
0,81	ELOVL4	ELOVL fatty acid elongase 4
0,81	CD33	CD33 molecule
0,81	AQP1	aquaporin 1 (Colton blood group)
0,81	RASAL1	RAS protein activator like 1 (GAP1 like)
0,81	RMDN2	regulator of microtubule dynamics 2
0,81	TMEM71	transmembrane protein 71
0,81	TAAR5	trace amine associated receptor 5

0,81	NAIP	NLR family, apoptosis inhibitory protein
0,81	NCOA2	nuclear receptor coactivator 2
0,81	KMT2D	lysine (K)-specific methyltransferase 2D
0,81	MED13L	mediator complex subunit 13-like
0,81	LOC100506388; RP11-1260E13.4; hosaru	uncharacterized LOC100506388; Transcript Identified by AceView; novel transcript, atisense to RPH3AL
0,81	IQGAP2	IQ motif containing GTPase activating protein 2
0,81	ZNF546	zinc finger protein 546
0,81	HTRA1	HtrA serine peptidase 1
0,81	TIAL1	Transcript Identified by AceView, Entrez Gene ID(s) 7073
0,81	HERC2	HECT and RLD domain containing E3 ubiquitin protein ligase 2
0,81	PPL	periplakin
0,81	PDE9A	phosphodiesterase 9A
0,81	ILF2	Jeck2013 ALT_DONOR, coding, INTERNAL, intronic best transcript NM_004515
0,81	METTL16	methyltransferase like 16
0,81	CRHR1	corticotropin releasing hormone receptor 1
0,81	PRRT4	proline-rich transmembrane protein 4
0,81	KRTAP9-1	keratin associated protein 9-1
0,81	EFCAB8	EF-hand calcium binding domain 8
0,81	KLF12	Kruppel-like factor 12
0,80	HUWE1	HECT, UBA and WWE domain containing 1, E3 ubiquitin protein ligase
0,80	C7orf60	chromosome 7 open reading frame 60
0,80	REG3A	regenerating islet-derived 3 alpha
0,80	XPNPEP1	Transcript Identified by AceView, Entrez Gene ID(s) 7511
0,80	PLEKHG2	pleckstrin homology domain containing, family G (with RhoGef domain) member 2
0,80	MGMT	O-6-methylguanine-DNA methyltransferase
0,80	ACBD4	acyl-CoA binding domain containing 4
0,80	PNMA6A	paraneoplastic Ma antigen family member 6A
0,80	CTPS2	Transcript Identified by AceView, Entrez Gene ID(s) 56474
0,80	PPFIBP2	PTPRF interacting protein, binding protein 2 (liprin beta 2)
0,80	TNMD	tenomodulin
0,80	DENND3	DENN/MADD domain containing 3
0,80	ASB16	ankyrin repeat and SOCS box containing 16
0,80	TREML1	triggering receptor expressed on myeloid cells-like 1
0,80	UNC45B	unc-45 myosin chaperone B
0,80	SPEN	spen family transcriptional repressor
0,80	CHIC2	cysteine rich hydrophobic domain 2
0,80	SOCS3	suppressor of cytokine signaling 3
0,80	LOC730668; CITF22-92A6.2	dynein heavy chain -like pseudogene; TEC
0,80	FAN1	FANCD2/FANCI-associated nuclease 1

0,80	DAB1	Dab, reelin signal transducer, homolog 1 (Drosophila)
0,80	ADCY10	adenylate cyclase 10 (soluble)
0,80	RSPH6A	radial spoke head 6 homolog A (Chlamydomonas)
0,80	TMEM26	transmembrane protein 26
0,80	SLC25A27	solute carrier family 25, member 27
0,80	PALD1	phosphatase domain containing, paladin 1
0,80	INAFM1	InaF-motif containing 1
0,80	REXO1L1P; REXO1L3P	REX1, RNA exonuclease 1 homolog-like 1, pseudogene; REX1, RNA exonuclease 1 homolog-like 3, pseudogene
0,80	SASH1	SAM and SH3 domain containing 1
0,80	MDN1	midasin AAA ATPase 1
0,80	DAPK2	death-associated protein kinase 2
0,80	IFFO2	intermediate filament family orphan 2
0,80	SNAPC4	Zhang2013 ALT_ACCEPTOR, ALT_DONOR, coding, INTERNAL, intronic best transcript NM_003086
0,80	DIP2C	disco-interacting protein 2 homolog C
0,80	P4HA3	prolyl 4-hydroxylase, alpha polypeptide III
0,80	FAM84A	family with sequence similarity 84, member A
0,80	COL4A4	collagen, type IV, alpha 4
0,80	GALR3	galanin receptor 3
0,80	RAB27A	RAB27A, member RAS oncogene family
0,80	FAM83B	family with sequence similarity 83, member B
0,80	KRTAP10-10	keratin associated protein 10-10
0,80	CCDC120	coiled-coil domain containing 120
0,80	NHSL1	NHS-like 1
0,80	NEURL4	neuralized E3 ubiquitin protein ligase 4
0,80	GRK5	Memczak2013 ANTISENSE, coding, INTERNAL, intronic best transcript NM_005308
0,80	KMT2C	lysine (K)-specific methyltransferase 2C
0,80	MCOLN3	mucolipin 3
0,80	SLC6A16	solute carrier family 6, member 16
0,80	NPY4R	neuropeptide Y receptor Y4
0,80	CYP21A2	cytochrome P450, family 21, subfamily A, polypeptide 2
0,80	KNDC1	Transcript Identified by AceView, Entrez Gene ID(s) 85442
0,80	RBP1	retinol binding protein 1, cellular
0,80	KIF24	kinesin family member 24
0,80	SBSPON	somatomedin B and thrombospondin type 1 domain containing
0,80	MYO9A	myosin IXA
0,80	ZKSCAN7	zinc finger with KRAB and SCAN domains 7
0,80	GLYAT	glycine-N-acyltransferase
0,80	APPL2	adaptor protein, phosphotyrosine interaction, PH domain and leucine zipper containing 2
0,80	WNK1	WNK lysine deficient protein kinase 1
0,80	KRTAP15-1	keratin associated protein 15-1

0,80	RPS4X	Zhang2013 ALT_ACCEPTOR, ALT_DONOR, coding, INTERNAL, intronic best transcript NM_001007
0,80	KRTAP10-9	keratin associated protein 10-9
0,80	MAP7	microtubule-associated protein 7
0,80	SVOPL	SVOP-like
0,80	KLF9	Kruppel-like factor 9
0,80	TPH2	tryptophan hydroxylase 2
0,80	CPEB2	cytoplasmic polyadenylation element binding protein 2
0,80	flawmawbo; GBP3	Transcript Identified by AceView; Memczak2013 ANTISENSE, CDS, coding, INTERNAL, intronic, UTR5 best transcript NM_018284
0,80	KCND1	potassium channel, voltage gated Shal related subfamily D, member 1
0,80	TBC1D30	TBC1 domain family, member 30
0,80	KCNK1	Transcript Identified by AceView, Entrez Gene ID(s) 3775
0,80	FAP	fibroblast activation protein alpha
0,80	ADCY2	adenylate cyclase 2 (brain)
0,80	MCTP2	multiple C2 domains, transmembrane 2
0,80	HERC2P3	hect domain and RLD 2 pseudogene 3
0,80	PRSS38	protease, serine 38
0,80	DIRC1	disrupted in renal carcinoma 1
0,80	CWH43	cell wall biogenesis 43 C-terminal homolog
0,80	UBL4B	ubiquitin-like 4B
0,80	PLA1A	phospholipase A1 member A
0,80	RRBP1	Zhang2013 ALT_ACCEPTOR, ALT_DONOR, coding, INTERNAL, intronic best transcript NM_001042576
0,80	MOCS1	molybdenum cofactor synthesis 1
0,80	ELAVL2	ELAV like neuron-specific RNA binding protein 2
0,80	FAM173B	Transcript Identified by AceView, Entrez Gene ID(s) 134145
0,80	SLC16A2	solute carrier family 16, member 2 (thyroid hormone transporter)
0,80	ZNF660	zinc finger protein 660
0,80	KLHDC8B	kelch domain containing 8B
0,80	BNC2	basonuclin 2
0,80	POU1F1	POU class 1 homeobox 1
0,80	ITPRIPL1	inositol 1,4,5-trisphosphate receptor interacting protein-like 1
0,80	LRRC37A	leucine rich repeat containing 37A
0,80	SPAG11A; SPAG11B	sperm associated antigen 11A; sperm associated antigen 11B
0,80	PRLR	prolactin receptor
0,80	SLC8A3	solute carrier family 8 (sodium/calcium exchanger), member 3
0,80	SIGLEC15	sialic acid binding Ig-like lectin 15
0,80	CTXN3	cortexin 3
0,80	SCNN1G	sodium channel, non voltage gated 1 gamma subunit

0,80	KIAA0825	KIAA0825
0,80	PHF20L1	Transcript Identified by AceView, Entrez Gene ID(s) 51105
0,80	CLMN	calmin (calponin-like, transmembrane)
0,80	KCTD19	potassium channel tetramerization domain containing 19
0,80	YEATS4	Transcript Identified by AceView, Entrez Gene ID(s) 8089
0,80	PON3	paraoxonase 3
0,80	SH3BP5L	SH3-binding domain protein 5-like
0,80	KLHL34	kelch-like family member 34
0,80	PDZD2	PDZ domain containing 2
0,80	S1PR2	sphingosine-1-phosphate receptor 2
0,80	KIRREL3-AS3	KIRREL3 antisense RNA 3
0,80	CCDC159	coiled-coil domain containing 159
0,80	DOCK3	dedicator of cytokinesis 3
0,80	CBY3	chibby homolog 3 (Drosophila)
0,80	TAAR9	trace amine associated receptor 9 (gene/pseudogene)
0,80	PTGFRN	prostaglandin F2 receptor inhibitor
0,80	MAPK8IP1	mitogen-activated protein kinase 8 interacting protein 1
0,80	XAGE3	X antigen family, member 3
0,80	DNASE2B	deoxyribonuclease II beta
0,80	SNX33	sorting nexin 33
0,80	FAM118A	family with sequence similarity 118, member A
0,79	TAS2R20	taste receptor, type 2, member 20
0,79	LOC100129216	beta-defensin 131-like
0,79	AMDHD1	amidohydrolase domain containing 1
0,79	ARHGEF10L	Rho guanine nucleotide exchange factor 10 like
0,79	PBXIP1	pre-B-cell leukemia homeobox interacting protein 1
0,79	VEPH1	ventricular zone expressed PH domain containing 1
0,79	KIAA1549L	KIAA1549-like
0,79	HCP5	HLA complex P5 (non-protein coding)
0,79	FAM198B	family with sequence similarity 198, member B
0,79	RALGAPA2	Ral GTPase activating protein, alpha subunit 2 (catalytic)
0,79	LGI2	leucine-rich repeat LGI family, member 2
0,79	CHFR	checkpoint with forkhead and ring finger domains, E3 ubiquitin protein ligase
0,79	HK2	hexokinase 2
0,79	GLT6D1	glycosyltransferase 6 domain containing 1
0,79	LOC339059; RP5-1142A6.8	uncharacterized LOC339059; novel transcript, antisense to FAM38A
0,79	COL4A5	collagen, type IV, alpha 5
0,79	BPIFB3	BPI fold containing family B, member 3
0,79	MEIS1	Transcript Identified by AceView, Entrez Gene ID(s) 4211
0,79	BPIFB6	BPI fold containing family B, member 6
0,79	TTC30B	tetratricopeptide repeat domain 30B

0,79	APBB3	amyloid beta (A4) precursor protein-binding, family B, member 3
0,79	PRR30	proline rich 30
0,79	NPTX1	neuronal pentraxin I
0,79	EPHA5	EPH receptor A5
0,79	KRBA2	KRAB-A domain containing 2
0,79	UBR4	ubiquitin protein ligase E3 component n-recognin 4
0,79	GRIN2A	glutamate receptor, ionotropic, N-methyl D-aspartate 2A
0,79	MFAP5	microfibrillar associated protein 5
0,79	LDHAL6B	lactate dehydrogenase A-like 6B
0,79	PCSK6	proprotein convertase subtilisin/kexin type 6
0,79	SYBU	syntabulin (syntaxin-interacting)
0,79	ADSSL1	adenylosuccinate synthase like 1
0,79	FAM107B	family with sequence similarity 107, member B
0,79	SSPN	sarcospan
0,79	CCDC149	coiled-coil domain containing 149
0,79	ATP9A	ATPase, class II, type 9A
0,79	ARHGEF12	Rho guanine nucleotide exchange factor (GEF) 12
0,79	TMEM218	transmembrane protein 218
0,79	INTS1	integrator complex subunit 1
0,79	ATP10A	ATPase, class V, type 10A
0,79	SPTAN1	spectrin, alpha, non-erythrocytic 1
0,79	OCM2	oncomodulin 2
0,79	ASH1L; MIR555	ash1 (absent, small, or homeotic)-like (Drosophila); microRNA 555
0,79	LOC100144595; AC009227.2; tikure	uncharacterized LOC100144595; novel transcript; Transcript Identified by AceView
0,79	ABHD1	abhydrolase domain containing 1
0,79	DEFA5	defensin, alpha 5, Paneth cell-specific
0,79	CGREF1	cell growth regulator with EF-hand domain 1
0,79	PIWIL4	piwi-like RNA-mediated gene silencing 4
0,79	SPATA3	spermatogenesis associated 3
0,79	PITPNC1	phosphatidylinositol transfer protein, cytoplasmic 1
0,79	LCA5L	Leber congenital amaurosis 5-like
0,79	COQ6	coenzyme Q6 monooxygenase
0,79	CCDC34	Transcript Identified by AceView, Entrez Gene ID(s) 91057
0,79	CLRN2	clarin 2
0,79	TMX4	thioredoxin-related transmembrane protein 4
0,79	UCK2	Jeck2013 ALT_ACCEPTOR, ALT_DONOR, coding, INTERNAL, intronic best transcript NM_012474
0,79	FAM129A	family with sequence similarity 129, member A
0,79	TTLL8	tubulin tyrosine ligase-like family member 8
0,79	ANKRD34B	ankyrin repeat domain 34B
0,79	POLD4	polymerase (DNA-directed), delta 4, accessory subunit
0,79	COCH	cochlin
0,79	STXBP5L	syntaxin binding protein 5-like

0,79	CNTN3	contactin 3 (plasmacytoma associated)
0,79	NAV1	neuron navigator 1
0,79	PLCE1	phospholipase C, epsilon 1
0,79	BACH2	Memczak2013 ALT_ACCEPTOR, ALT_DONOR, coding, INTERNAL, intronic best transcript NM_001170794
0,79	C20orf196	chromosome 20 open reading frame 196
0,79	SAMD4A	Jeck2013 ALT_ACCEPTOR, ALT_DONOR, coding, INTERNAL, intronic best transcript NM_015589
0,79	ZNF77	zinc finger protein 77
0,79	TMEM71	transmembrane protein 71
0,79	PLCB4	phospholipase C, beta 4
0,79	DTHD1	death domain containing 1
0,79	HNF1B	HNF1 homeobox B
0,79	EFCAB12	EF-hand calcium binding domain 12
0,79	SYT1	synaptotagmin I
0,79	CCL3	chemokine (C-C motif) ligand 3
0,79	LINC00656	long intergenic non-protein coding RNA 656
0,79	HTR2C	5-hydroxytryptamine (serotonin) receptor 2C, G protein-coupled
0,79	KATNA1	katanin p60 (ATPase containing) subunit A 1
0,79	MMP10	matrix metallopeptidase 10
0,79	ABCD3	Transcript Identified by AceView, Entrez Gene ID(s) 5825
0,79	FOXR2	forkhead box R2
0,79	ZFYVE21	zinc finger, FYVE domain containing 21
0,79	SPC25	SPC25, NDC80 kinetochore complex component
0,79	KRTAP9-6	keratin associated protein 9-6
0,79	SLC44A5	solute carrier family 44, member 5
0,79	RGN	regucalcin
0,79	RIPPLY3	ripply transcriptional repressor 3
0,79	CHST12	carbohydrate (chondroitin 4) sulfotransferase 12
0,79	STAU2-AS1	STAU2 antisense RNA 1
0,79	TTLL1	tubulin tyrosine ligase-like family member 1
0,79	ATP2B4	ATPase, Ca++ transporting, plasma membrane 4
0,79	RNF217	ring finger protein 217
0,79	KHDC1	KH homology domain containing 1
0,79	RIMS1	regulating synaptic membrane exocytosis 1
0,78	TMEM207	transmembrane protein 207
0,78	SERPINB13	serpin peptidase inhibitor, clade B (ovalbumin), member 13
0,78	ARHGAP15	Rho GTPase activating protein 15
0,78	PLA2R1	phospholipase A2 receptor 1
0,78	ALKBH7	alkB homolog 7
0,78	NBEA	neurobeachin
0,78	HFE	hemochromatosis
0,78	EP300; MIR1281	E1A binding protein p300; microRNA 1281
0,78	FOXO1	forkhead box O1
0,78	STAC2	SH3 and cysteine rich domain 2
0,78	KLF15	Kruppel-like factor 15

0,78	SLF1	SMC5-SMC6 complex localization factor 1
0,78	DIXDC1	DIX domain containing 1
0,78	CST9L	cystatin 9-like
0,78	CIT; MIR1178	citron rho-interacting serine/threonine kinase; microRNA 1178
0,78	COL15A1	collagen, type XV, alpha 1
0,78	CYP2J2	cytochrome P450, family 2, subfamily J, polypeptide 2
0,78	BSND	barttin CLCNK-type chloride channel accessory beta subunit
0,78	TBXAS1	thromboxane A synthase 1 (platelet)
0,78	EPM2A	epilepsy, progressive myoclonus type 2A, Lafora disease (laforin)
0,78	SBK2	SH3 domain binding kinase family, member 2
0,78	LSAMP	limbic system-associated membrane protein
0,78	RGCC	regulator of cell cycle
0,78	TTC30A	tetratricopeptide repeat domain 30A
0,78	DEFB107A; DEFB107B	defensin, beta 107A; defensin, beta 107B
0,78	APBB1	amyloid beta (A4) precursor protein-binding, family B, member 1 (Fe65)
0,78	DOCK10	Transcript Identified by AceView, Entrez Gene ID(s) 55619
0,78	MYO7B	myosin VIIB
0,78	MIOX	myo-inositol oxygenase
0,78	PDE4D	Memczak2013 ANTISENSE, coding, INTERNAL, intronic best transcript NM_001165899
0,78	LAMB3; MIR4260	laminin, beta 3; microRNA 4260
0,78	TAGLN	transgelin
0,78	CCDC84	coiled-coil domain containing 84
0,78	FGF12	fibroblast growth factor 12
0,78	PHLDB2; PLCXD2	pleckstrin homology-like domain, family B, member 2; phosphatidylinositol-specific phospholipase C, X domain containing 2
0,78	PTPRR	protein tyrosine phosphatase, receptor type, R
0,78	SPSB2	splA/ryanodine receptor domain and SOCS box containing 2
0,78	TM4SF20	transmembrane 4 L six family member 20
0,78	ELF1	Transcript Identified by AceView, Entrez Gene ID(s) 1997; 100128628
0,78	CPE	carboxypeptidase E
0,78	RAPH1	Ras association (RalGDS/AF-6) and pleckstrin homology domains 1
0,78	SLC9A1	solute carrier family 9, subfamily A (NHE1, cation proton antiporter 1), member 1
0,78	TMEM25	transmembrane protein 25
0,78	LRP12	LDL receptor related protein 12
0,78	ITGAM	integrin, alpha M (complement component 3 receptor 3 subunit)
0,78	C19orf57	chromosome 19 open reading frame 57

0,78	GPC2	glypican 2
0,78	TP53I13	tumor protein p53 inducible protein 13
0,78	FAM134B	family with sequence similarity 134, member B
0,78	FOXS1	forkhead box S1
0,78	SYPL2	synaptophysin-like 2
0,78	FANK1	fibronectin type III and ankyrin repeat domains 1
0,78	FGF10	fibroblast growth factor 10
0,78	VLDLR	very low density lipoprotein receptor
0,78	CCDC60	coiled-coil domain containing 60
0,78	RNF44	ring finger protein 44
0,78	ABCC1	ATP binding cassette subfamily C member 1
0,78	CARD9	caspase recruitment domain family, member 9
0,78	ADPRH	ADP-ribosylarginine hydrolase
0,78	APTX	aprataxin
0,77	CGA	glycoprotein hormones, alpha polypeptide
0,77	C3orf22	chromosome 3 open reading frame 22
0,77	AVL9	AVL9 homolog (S. cerevisiase)
0,77	TRPV4	transient receptor potential cation channel, subfamily V, member 4
0,77	WRN	Werner syndrome, RecQ helicase-like
0,77	SMIM19	small integral membrane protein 19
0,77	SKIDA1	SKI/DACH domain containing 1
0,77	CCDC80; LINC01279	coiled-coil domain containing 80; long intergenic non-protein coding RNA 1279
0,77	SLC44A3	solute carrier family 44, member 3
0,77	C5AR2	complement component 5a receptor 2
0,77	INADL	InaD-like (Drosophila)
0,77	ADAM32	ADAM metallopeptidase domain 32
0,77	PARP9	poly(ADP-ribose) polymerase family member 9
0,77	SLC26A4	solute carrier family 26 (anion exchanger), member 4
0,77	CXCL10	chemokine (C-X-C motif) ligand 10
0,77	LAMP3	lysosomal-associated membrane protein 3
0,77	VMAC	vimentin-type intermediate filament associated coiled-coil protein
0,77	TRIM38	tripartite motif containing 38
0,77	B3GNTL1	UDP-GlcNAc:betaGal beta-1,3-N-acetylglucosaminyltransferase-like 1
0,77	MAP2K6	mitogen-activated protein kinase kinase 6
0,77	WDR1	Jeck2013 ANTISENSE, CDS, coding, INTERNAL, intronic, OVCODE, OVEXON best transcript NM_017491
0,77	FANCM	Fanconi anemia complementation group M
0,77	AP5Z1	adaptor-related protein complex 5, zeta 1 subunit
0,77	WDR27	WD repeat domain 27
0,77	ZNF462	zinc finger protein 462
0,77	DNAJB5	DnaJ (Hsp40) homolog, subfamily B, member 5
0,77	CKMT1B; CKMT1A	creatine kinase, mitochondrial 1B; creatine kinase, mitochondrial 1A
0,77	SYT10	synaptotagmin X

0,77	BICD2	Memczak2013 ALT_ACCEPTOR, ALT_DONOR, coding, INTERNAL, intronic best transcript NM_001003800
0,77	GOLGA6C	golgin A6 family, member C
0,77	PODN	Transcript Identified by AceView, Entrez Gene ID(s) 127435
0,77	JARID2	jumonji, AT rich interactive domain 2
0,77	TRIP11	thyroid hormone receptor interactor 11
0,77	TLCD2	TLC domain containing 2
0,77	OTOP3	otopetrin 3
0,77	IGF2R	insulin-like growth factor 2 receptor
0,77	ARHGEF11	Rho guanine nucleotide exchange factor 11
0,77	CRLF1	cytokine receptor-like factor 1
0,77	TC2N	tandem C2 domains, nuclear
0,77	PDGFRB	platelet-derived growth factor receptor, beta polypeptide
0,77	SORT1	sortilin 1
0,77	PALM2-AKAP2	PALM2-AKAP2 readthrough
0,77	SERPINA5	serpin peptidase inhibitor, clade A (alpha-1 antiproteinase, antitrypsin), member 5
0,77	MYBPH	myosin binding protein H
0,77	GPR85	G protein-coupled receptor 85
0,77	FTH1P18	ferritin, heavy polypeptide 1 pseudogene 18
0,77	LRRTM2	leucine rich repeat transmembrane neuronal 2
0,77	SCRT2	scratch family zinc finger 2
0,77	DNASE1L2	deoxyribonuclease I-like 2
0,77	ITGB3	integrin beta 3
0,77	PAGE5	P antigen family, member 5 (prostate associated)
0,76	DDO	D-aspartate oxidase
0,76	LRP6	LDL receptor related protein 6
0,76	KCNK5	potassium channel, two pore domain subfamily K, member 5
0,76	EIF2AK3	eukaryotic translation initiation factor 2-alpha kinase 3
0,76	SULT2A1	sulfotransferase family 2A member 1
0,76	ASB4	ankyrin repeat and SOCS box containing 4
0,76	CFAP52	cilia and flagella associated protein 52
0,76	CLEC3B	C-type lectin domain family 3, member B
0,76	ZNF713	zinc finger protein 713
0,76	KCNG4	potassium channel, voltage gated modifier subfamily G, member 4
0,76	FRAT2	frequently rearranged in advanced T-cell lymphomas 2
0,76	CNTNAP5	contactin associated protein-like 5
0,76	TRIM55	tripartite motif containing 55
0,76	C5orf46	chromosome 5 open reading frame 46
0,76	GABRQ	gamma-aminobutyric acid (GABA) A receptor, theta
0,76	SCUBE3	signal peptide, CUB domain, EGF-like 3
0,76	STXBP5	syntaxin binding protein 5 (tomosyn)
0,76	TAS2R14	taste receptor, type 2, member 14

0,76	TTC32	tetratricopeptide repeat domain 32
0,76	C2orf54	chromosome 2 open reading frame 54
0,76	LYSMD4	LysM, putative peptidoglycan-binding, domain containing 4
0,76	SLAMF7	SLAM family member 7
0,76	MRPL23	mitochondrial ribosomal protein L23
0,76	DYNC1H1	dynein, cytoplasmic 1, heavy chain 1
0,76	FAM20C	family with sequence similarity 20, member C
0,76	TMEM205	transmembrane protein 205
0,76	GCC1	GRIP and coiled-coil domain containing 1
0,76	MMP12	matrix metallopeptidase 12
0,76	POM121L2	POM121 transmembrane nucleoporin-like 2
0,76	GRAMD4	GRAM domain containing 4
0,76	NANOS1	nanos homolog 1 (Drosophila)
0,76	RNF213	ring finger protein 213
0,76	ETV5	ets variant 5
0,76	POGZ	Transcript Identified by AceView, Entrez Gene ID(s) 23126
0,76	NAIP	NLR family, apoptosis inhibitory protein
0,76	BOD1L1	biorientation of chromosomes in cell division 1-like 1
0,76	ANKRD6	ankyrin repeat domain 6
0,76	NID1	nidogen 1
0,76	OR52E2	olfactory receptor, family 52, subfamily E, member 2
0,75	PPBP	pro-platelet basic protein
0,75	PPM1K	protein phosphatase, Mg2+/Mn2+ dependent, 1K
0,75	CNTF	ciliary neurotrophic factor
0,75	GPR183	G protein-coupled receptor 183
0,75	KDM5D	lysine (K)-specific demethylase 5D
0,75	PYGO1	pygopus family PHD finger 1
0,75	THBS3	thrombospondin 3
0,75	HGF	Transcript Identified by AceView, Entrez Gene ID(s) 3082
0,75	WNT1	wingless-type MMTV integration site family, member 1
0,75	STAMBPL1	STAM binding protein-like 1
0,75	XRCC6BP1	XRCC6 binding protein 1
0,75	CELSR1	cadherin, EGF LAG seven-pass G-type receptor 1
0,75	EDC4	enhancer of mRNA decapping 4
0,75	GTF3C1	general transcription factor IIIC subunit 1
0,75	ZNF503-AS2	ZNF503 antisense RNA 2
0,75	HSPB3	heat shock 27kDa protein 3
0,75	FAT1	FAT atypical cadherin 1
0,75	SERPINE3	serpin peptidase inhibitor, clade E (nexin, plasminogen activator inhibitor type 1), member 3
0,75	POLB	polymerase (DNA directed), beta
0,75	C14orf178	chromosome 14 open reading frame 178
0,75	CDKL3	cyclin-dependent kinase-like 3
0,75	MYL6	Memczak2013 ANTISENSE, coding, INTERNAL, intronic best transcript NM_079423

0,75	ESD	Transcript Identified by AceView, Entrez Gene ID(s) 2098
0,75	LRRC1	leucine rich repeat containing 1
0,75	CNNM1	cyclin and CBS domain divalent metal cation transport mediator 1
0,75	PCDHB16	protocadherin beta 16
0,75	NANOS2	nanos homolog 2 (Drosophila)
0,75	GRIK2	glutamate receptor, ionotropic, kainate 2
0,75	NPNT	nephronectin
0,75	LTBP1	latent transforming growth factor beta binding protein 1
0,75	NLGN1	neuroligin 1
0,75	PARP16	poly(ADP-ribose) polymerase family member 16
0,75	NAP1L1	Memczak2013 ALT_ACCEPTOR, ALT_DONOR, coding, INTERNAL, intronic best transcript NM_139207
0,75	NPBWR1	neuropeptides B/W receptor 1
0,75	ABCG1	ATP binding cassette subfamily G member 1
0,75	RNF215	ring finger protein 215
0,75	MLXIP	MLX interacting protein
0,75	MYLK	myosin light chain kinase
0,75	ZFHX3	zinc finger homeobox 3
0,75	ZBTB10	zinc finger and BTB domain containing 10
0,75	C4orf36	chromosome 4 open reading frame 36
0,74	ZNF554	zinc finger protein 554
0,74	TLN1; MIR6852	talin 1; microRNA 6852
0,74	TCF23	transcription factor 23
0,74	IGSF11	immunoglobulin superfamily, member 11
0,74	CLUL1	clusterin-like 1 (retinal)
0,74	TANC2	tetratricopeptide repeat, ankyrin repeat and coiled-coil containing 2
0,74	C1QTNF9B-AS1	C1QTNF9B antisense RNA 1
0,74	GOLGA6L9	golgin A6 family-like 9
0,74	ZFYVE9	zinc finger, FYVE domain containing 9
0,74	FAM150B	family with sequence similarity 150, member B
0,74	CRELD1	cysteine rich with EGF-like domains 1
0,74	MAML2	mastermind-like transcriptional coactivator 2
0,74	FBXO32	F-box protein 32
0,74	THAP9	THAP domain containing 9
0,74	CLCN5	chloride channel, voltage-sensitive 5
0,74	NR3C2	nuclear receptor subfamily 3, group C, member 2
0,74	TSPAN15	tetraspanin 15
0,74	LCK	LCK proto-oncogene, Src family tyrosine kinase
0,74	TEX35	testis expressed 35
0,74	ADAMTS13	ADAM metallopeptidase with thrombospondin type 1 motif 13
0,74	CTRC	chymotrypsin C (caldecrin)
0,74	TFAP2D	transcription factor AP-2 delta (activating enhancer binding protein 2 delta)

0,74	GNG7	Transcript Identified by AceView, Entrez Gene ID(s) 2788
0,74	ANKRD44	ankyrin repeat domain 44
0,73	DZIP1L	DAZ interacting zinc finger protein 1-like
0,73	NLRP4	NLR family, pyrin domain containing 4
0,73	HPD	4-hydroxyphenylpyruvate dioxygenase
0,73	RNF222	ring finger protein 222
0,73	SFRP5	secreted frizzled-related protein 5
0,73	FAM213B	Memczak2013 ANTISENSE, coding, INTERNAL, UTR3 best transcript NM_001195737
0,73	SULT1B1	sulfotransferase family 1B member 1
0,73	POTEI	POTE ankyrin domain family, member I
0,73	DGKD	diacylglycerol kinase, delta 130kDa
0,73	CSMD3	CUB and Sushi multiple domains 3
0,73	TSNAX	Memczak2013 ANTISENSE, CDS, coding, INTERNAL, UTR3 best transcript NM_005999
0,73	FAT4	FAT atypical cadherin 4
0,73	ITPR3	inositol 1,4,5-trisphosphate receptor, type 3
0,73	IL13	interleukin 13
0,73	ZNF518A	zinc finger protein 518A
0,73	TRIM66	tripartite motif containing 66
0,73	LAMA3	laminin, alpha 3
0,73	ANKRD29	ankyrin repeat domain 29
0,73	SERINC5	Memczak2013 ALT_ACCEPTOR, ALT_DONOR, coding, INTERNAL, intronic best transcript NM_001174072
0,73	UTRN	utrophin
0,73	ANKFN1	ankyrin-repeat and fibronectin type III domain containing 1
0,73	CFB	complement factor B
0,73	FGL2	fibrinogen-like 2
0,73	TRIM6	tripartite motif containing 6
0,73	OCRL	Transcript Identified by AceView, Entrez Gene ID(s) 4952
0,73	LAMA1	laminin, alpha 1
0,73	HBE1	hemoglobin, epsilon 1
0,73	SCAMP5	secretory carrier membrane protein 5
0,73	TTC28	Jeck2013 ALT_ACCEPTOR, ALT_DONOR, coding, INTERNAL, intronic best transcript NM_001145418
0,72	HSPG2	heparan sulfate proteoglycan 2
0,72	ZNF318	zinc finger protein 318
0,72	SMCR5	Smith-Magenis syndrome chromosome region, candidate 5 (non-protein coding)
0,72	COL3A1; MIR3606	collagen, type III, alpha 1; microRNA 3606
0,72	IGSF10	immunoglobulin superfamily, member 10
0,72	NASP	Zhang2013 ALT_ACCEPTOR, ALT_DONOR, coding, INTERNAL, intronic best transcript NM_002482
0,72	SYNE2	spectrin repeat containing, nuclear envelope 2

0,72	PAK3	p21 protein (Cdc42/Rac)-activated kinase 3
0,72	BTBD19	BTB (POZ) domain containing 19
0,72	FN1	fibronectin 1
0,72	DGKD	diacylglycerol kinase, delta 130kDa
0,72	LINC00494	long intergenic non-protein coding RNA 494
0,72	CKMT1A	creatine kinase, mitochondrial 1A
0,72	FBLN5	fibulin 5
0,72	VN1R1	vomeronasal 1 receptor 1
0,72	ABCA7	ATP binding cassette subfamily A member 7
0,72	MLIP	muscular LMNA-interacting protein
0,72	KCNH1	potassium channel, voltage gated eag related subfamily H, member 1
0,72	LINC00092	long intergenic non-protein coding RNA 92
0,72	KMT2B	lysine (K)-specific methyltransferase 2B
0,72	HFM1	HFM1, ATP-dependent DNA helicase homolog
0,72	TRAPPC4	trafficking protein particle complex 4
0,71	RCBTB2	regulator of chromosome condensation (RCC1) and BTB (POZ) domain containing protein 2
0,71	DGKD	diacylglycerol kinase, delta 130kDa
0,71	OXCT1	Transcript Identified by AceView, Entrez Gene ID(s) 5019
0,71	RSPH3	radial spoke 3 homolog (Chlamydomonas)
0,71	C10orf62	chromosome 10 open reading frame 62
0,71	ACAP2; ACAP2-IT1; AC090018.3	Transcript Identified by AceView, Entrez Gene ID(s) 23527; ACAP2 intronic transcript 1 (non-protein coding) [Source:HGNC Symbol;Acc:HGNC:41426]; novel transcript; ACAP2 intronic transcript 1 [Source:HGNC Symbol;Acc:HGNC:41426]
0,71	SORBS1	Transcript Identified by AceView, Entrez Gene ID(s) 10580
0,71	C2CD4A	C2 calcium-dependent domain containing 4A
0,71	HDLBP	Transcript Identified by AceView, Entrez Gene ID(s) 3069
0,71	ZNF660	zinc finger protein 660
0,71	QPCT	glutaminyl-peptide cyclotransferase
0,71	LYSMD2	LysM, putative peptidoglycan-binding, domain containing 2
0,71	PRAMEF16; PRAMEF17	PRAME family member 16; PRAME family member 17
0,71	FBXO43	F-box protein 43
0,70	TMX4	Memczak2013 ALT_ACCEPTOR, ALT_DONOR, coding, INTERNAL, intronic best transcript NM_021156
0,70	IFT88	intraflagellar transport 88
0,70	MAGIX	MAGI family member, X-linked
0,70	ACSM6	acyl-CoA synthetase medium-chain family member 6
0,70	AMOT; MIR4329	angiomotin; microRNA 4329
0,70	ZZEF1	zinc finger, ZZ-type with EF-hand domain 1
0,69	BBOF1	basal body orientation factor 1
0,69	LOC389834; AL354822.1	ankyrin repeat domain 57 pseudogene

0,69	KLF13	Memczak2013 ANTISENSE, coding, INTERNAL, intronic best transcript NM_015995
0,69	LRRC3	leucine rich repeat containing 3
0,69	DLX4	distal-less homeobox 4
0,69	TBC1D2B	TBC1 domain family, member 2B
0,69	DGCR8	Zhang2013 ALT_ACCEPTOR, ALT_DONOR, coding, INTERNAL, intronic best transcript NM_022720
0,68	SGCA	sarcoglycan alpha
0,68	POU3F1	POU class 3 homeobox 1
0,68	C20orf195	chromosome 20 open reading frame 195
0,67	TNC	tenascin C
0,67	CYP24A1	cytochrome P450, family 24, subfamily A, polypeptide 1
0,67	CHRNB4	cholinergic receptor, nicotinic beta 4
0,67	PRKAR1A	Memczak2013 ALT_ACCEPTOR, ALT_DONOR, coding, INTERNAL, intronic best transcript NM_212471
0,67	RCOR2	REST corepressor 2
0,67	RADIL	Ras association and DIL domains
0,67	PCDHB3	protocadherin beta 3
0,66	TM4SF19-TCTEX1D2	TM4SF19-TCTEX1D2 readthrough (NMD candidate)
0,66	ITGA11	integrin alpha 11
0,66	MARCH9	membrane associated ring finger 9
0,65	PDE5A	phosphodiesterase 5A, cGMP-specific
0,65	DUSP16	dual specificity phosphatase 16
0,65	PTBP2	Transcript Identified by AceView, Entrez Gene ID(s) 58155
0,65	MUC16	mucin 16, cell surface associated
0,65	VPS13D	vacuolar protein sorting 13 homolog D (S. cerevisiae)
0,64	LRRC58	Memczak2013 ANTISENSE, CDS, coding, INTERNAL, intronic, UTR3 best transcript NM_001099678
0,64	FITM1	fat storage-inducing transmembrane protein 1
0,62	TAC3	tachykinin 3
0,61	BGN	biglycan
0,61	ZNF658B	zinc finger protein 658B, pseudogene
0,60	BUD31	Transcript Identified by AceView, Entrez Gene ID(s) 8896
0,59	PPP1R3B	protein phosphatase 1, regulatory subunit 3B
0,57	GPR141	G protein-coupled receptor 141
