# Supplementary material for: Integration of Transcriptomics and Metabolomics to Reveal the Molecular Mechanisms Underlying Rhodium Nanoparticles-Based Photodynamic Cancer Therapy
Source: Pharmaceutics. 2021 Oct 6;13(10):1629. doi: 10.3390/pharmaceutics13101629 (PMC8539937; doi:10.3390/pharmaceutics13101629)
Supplement: Supplementary file 1 [file pharmaceutics-13-01629-s001.zip › Supplementary information.pdf]

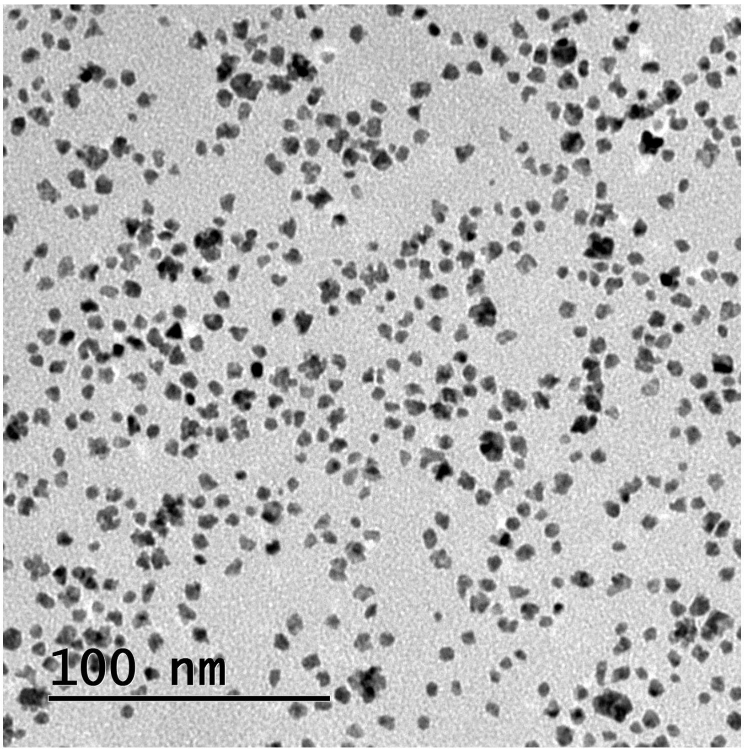


**Supplemental Figure 1.** TEM micrograph of RhNPs on aqueous suspension. Image was obtained using a JEOL JEM 1400 PLUS microscope.

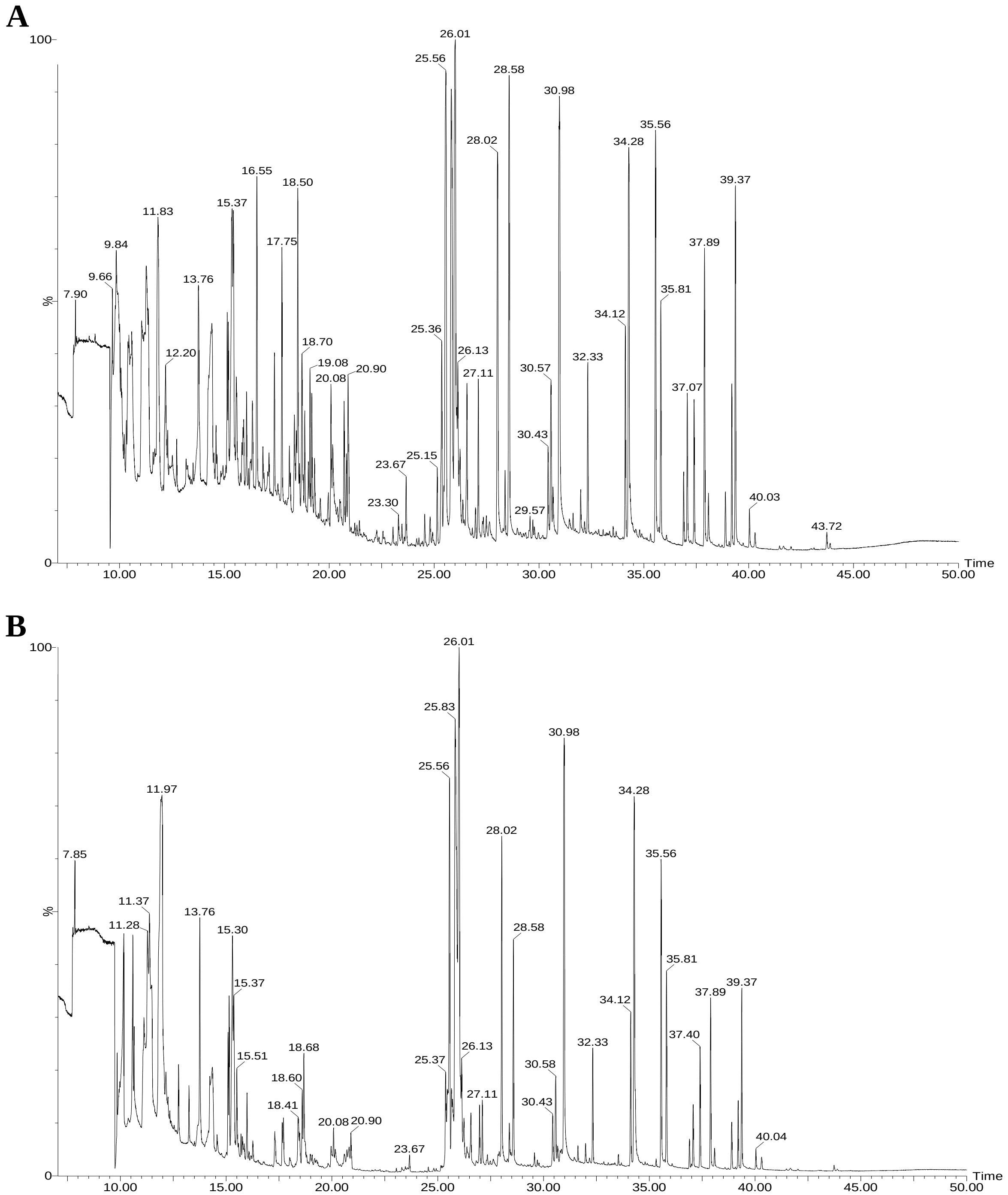


**Supplemental Figure 2.** Representative chromatograms obtained in TIC mode for metabolite extracts from control (A) and treated (B) samples.

A

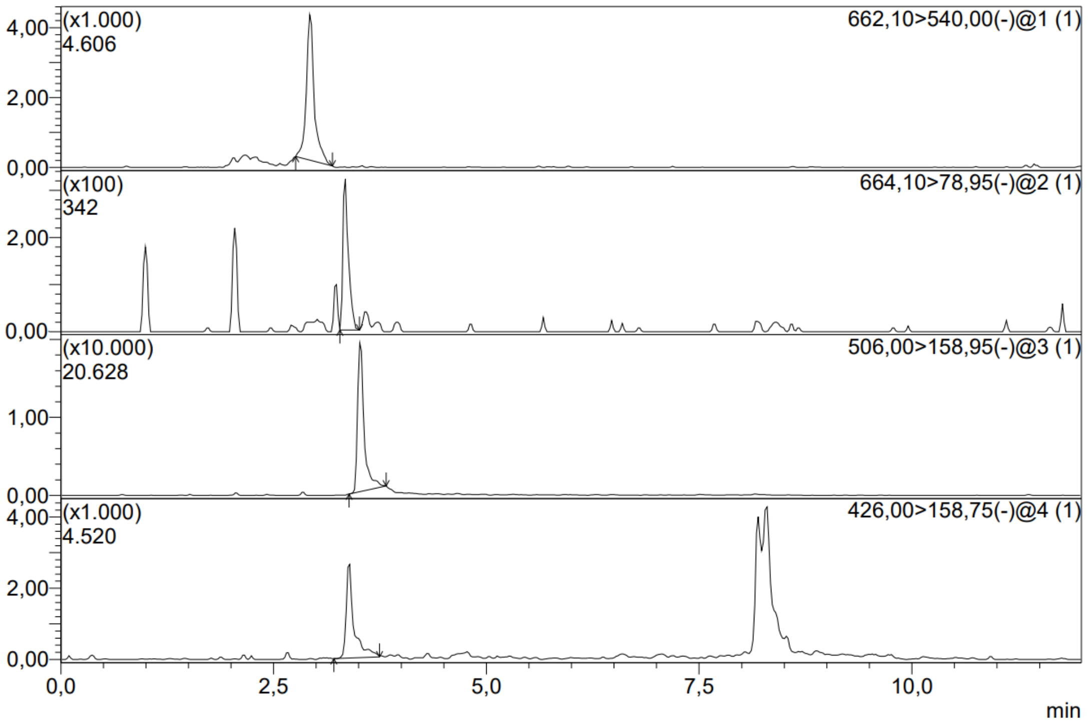


B

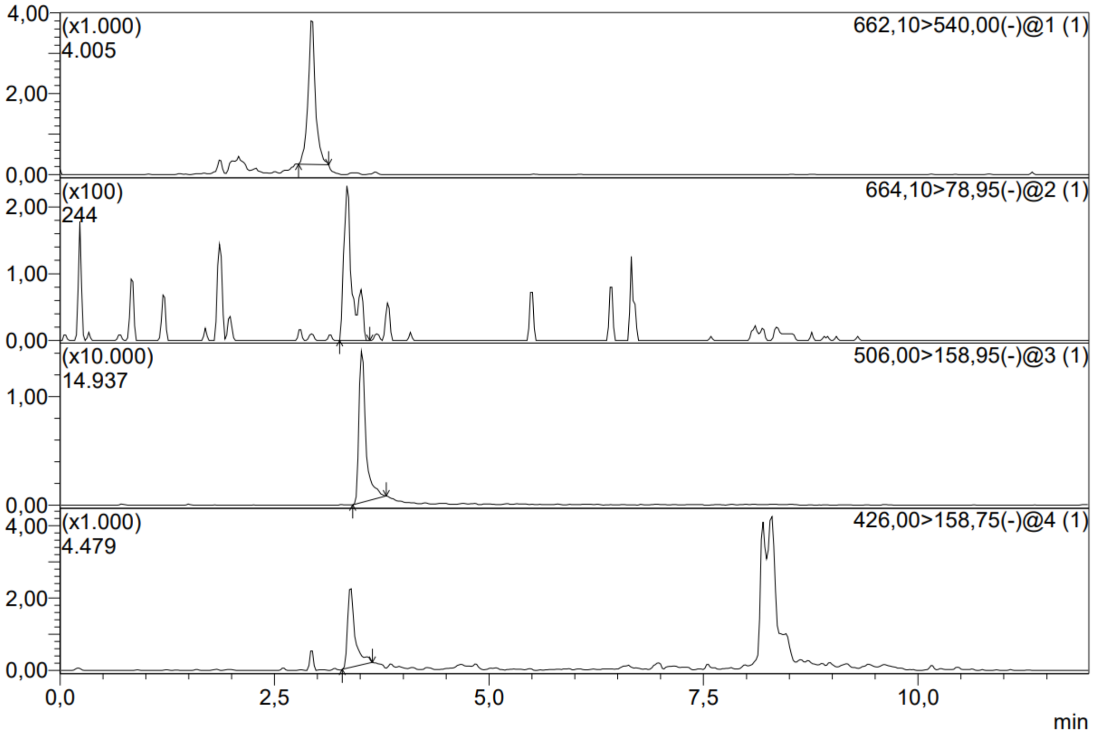


**Supplemental Figure 3.** Representative chromatograms from control (A) and treated (B) samples obtained by LC-QqQ-MS operating in MRM mode for $NAD^+$, NADH, ATP and ADP, respectively.

**Supplemental table S1.** Transitions and collision energies used for the quantification of ATP, ADP, NADH and $NAD^+$ by LC-QqQ-MS working in MRM mode.

| **Metabolite** | **Precursor ion $[M-H]^+$ (m/z)** | **Product ion (m/z)** | **Collision energy (eV)** |
|---|---|---|---|
| ATP | 506.00 | 426.00 | 30 |
| ADP | 426.00 | 158.75 | 26 |
| NADH | 664.10 | 78.95 | 54 |
| $NAD^+$ | 662.10 | 540.00 | 16 |

**Supplemental table S3.** Common metabolites identified in control and treated samples by GC-TOF-MS with a minimum NIST Rmatch value of 700.

| Number | Metabolite | Retention time (min) | NIST Rmatch |
|---|---|---|---|
| 1 | Acetate | 12.293 | 709 |
| 2 | L-valine | 12.371 | 902 |
| 3 | Serine | 13.446 | 769 |
| 4 | L-isoleucine | 14.144 | 907 |
| 5 | Pyruvate | 14.484 | 778 |
| 6 | Propanedionate | 14.743 | 813 |
| 7 | Propionate | 14.872 | 835 |
| 8 | Butanoate | 15.047 | 739 |
| 9 | Nonanoate | 15.425 | 778 |
| 10 | L-threonine | 15.978 | 833 |
| 11 | Pentanedionate | 16.319 | 801 |
| 12 | Cyclohexanamine | 16.587 | 921 |
| 13 | B-alanine | 16.795 | 811 |
| 14 | Decanoate | 17.451 | 805 |
| 15 | Malate | 18.056 | 865 |
| 16 | L-proline | 18.468 | 888 |
| 17 | L-aspartate | 18.665 | 890 |
| 18 | Creatinine | 19.198 | 735 |
| 19 | Cytein | 19.394 | 750 |
| 20 | Dodecanoate | 21.197 | 852 |
| 21 | D-xylofuranose | 21.684 | 851 |
| 22 | Pyrimidine | 21.766 | 733 |
| 23 | Phosphate | 23.296 | 905 |
| 24 | Azelate | 23.807 | 826 |
| 25 | Propanetricarboxylate | 24.278 | 823 |
| 26 | Tetradecanoate | 24.696 | 865 |
| 27 | Fructose | 25.022 | 837 |
| 28 | Galactose | 25.430 | 857 |
| 29 | Glucose | 25.575 | 709 |
| 30 | Hexadecanenitrile | 25.580 | 900 |
| 31 | D-mannitol | 25.864 | 713 |
| 32 | N-pentadecanoate | 26.324 | 823 |
| 33 | Gluconate | 26.562 | 778 |
| 34 | Hexadecanoate | 27.885 | 826 |
| 35 | Oleanitrile | 28.417 | 930 |
| 36 | Myo-inositol | 28.438 | 814 |
| 37 | Octadecanenitrile | 28.830 | 880 |

| | | | |
|---|---|---|---|
| 38 | Heptadecanoate | 28.975 | 863 |
| 39 | 11-trans-octadecenoate | 30.546 | 864 |
| 40 | Oleate | 30.671 | 791 |
| 41 | Dehydroabietate | 32.711 | 748 |
| 42 | Mystirate | 32.938 | 889 |
| 42 | Eicosanoate | 33.553 | 845 |
| 44 | Monopalmitoglycerol | 35.026 | 826 |
| 45 | 2- monostearin | 37.367 | 831 |
| 46 | Octadecanoate | 37.574 | 879 |
| 47 | Decanedionate | 38.081 | 844 |
| 48 | Cholesterol | 41.615 | 895 |

**Supplemental Table S4.** Pearson’s correlation matrix data for the 48 identified metabolites.

| | 1 | 2 | 3 | 4 | 5 | 6 | 7 | 8 | 9 | 10 | 11 | 12 | 13 | 14 | 15 | 16 | 17 | 18 | 19 | 20 | 21 | 22 | 23 | 24 | 25 | 26 | 27 | 28 | 29 | 30 | 31 | 32 | 33 | 34 | 35 | 36 | 37 | 38 | 39 | 40 | 41 | 42 | 43 | 44 | 45 | 46 | 47 | 48 |
|---|---|---|---|---|---|---|---|---|---|---|---|---|---|---|---|---|---|---|---|---|---|---|---|---|---|---|---|---|---|---|---|---|---|---|---|---|---|---|---|---|---|---|---|---|---|---|---|---|
| **1** | **1** | | | | | | | | | | | | | | | | | | | | | | | | | | | | | | | | | | | | | | | | | | | | | | | |
| **2** | **0,7885** | **1** | | | | | | | | | | | | | | | | | | | | | | | | | | | | | | | | | | | | | | | | | | | | | | |
| **3** | -0,1752 | -0,0524 | **1** | | | | | | | | | | | | | | | | | | | | | | | | | | | | | | | | | | | | | | | | | | | | | |
| **4** | 0,5042 | **0,6641** | **0,2869** | **1** | | | | | | | | | | | | | | | | | | | | | | | | | | | | | | | | | | | | | | | | | | | | |
| **5** | 0,1986 | 0,3224 | 0,3033 | 0,3247 | **1** | | | | | | | | | | | | | | | | | | | | | | | | | | | | | | | | | | | | | | | | | | | |
| **6** | -0,0647 | -0,0720 | -0,0784 | -0,3252 | 0,1201 | **1** | | | | | | | | | | | | | | | | | | | | | | | | | | | | | | | | | | | | | | | | | | |
| **7** | 0,1918 | 0,2615 | -0,4781 | -0,1908 | 0,2586 | **0,6497** | **1** | | | | | | | | | | | | | | | | | | | | | | | | | | | | | | | | | | | | | | | | | |
| **8** | -0,0649 | 0,1360 | -0,0796 | 0,1246 | 0,0462 | 0,4306 | **0,6288** | **1** | | | | | | | | | | | | | | | | | | | | | | | | | | | | | | | | | | | | | | | | |
| **9** | -0,4384 | -0,1702 | **0,6791** | 0,0812 | -0,0723 | -0,1230 | -0,3929 | 0,2223 | **1** | | | | | | | | | | | | | | | | | | | | | | | | | | | | | | | | | | | | | | | |
| **10** | -0,1079 | 0,0532 | **0,7602** | 0,3227 | 0,4298 | 0,4470 | 0,0422 | 0,2424 | 0,3341 | **1** | | | | | | | | | | | | | | | | | | | | | | | | | | | | | | | | | | | | | | |
| **11** | 0,1707 | -0,0369 | 0,0239 | 0,1522 | 0,0902 | 0,4299 | 0,1892 | 0,1984 | -0,0221 | 0,3173 | **1** | | | | | | | | | | | | | | | | | | | | | | | | | | | | | | | | | | | | | |
| **12** | 0,0818 | 0,0533 | -0,0380 | 0,0220 | -0,0492 | -0,1189 | -0,2114 | -0,5502 | -0,1471 | -0,1139 | -0,2582 | **1** | | | | | | | | | | | | | | | | | | | | | | | | | | | | | | | | | | | | |
| **13** | 0,0443 | 0,0692 | 0,1746 | 0,1653 | **0,5902** | 0,3683 | 0,1449 | -0,0562 | 0,0059 | 0,4081 | **0,6404** | 0,2094 | **1** | | | | | | | | | | | | | | | | | | | | | | | | | | | | | | | | | | | |
| **14** | 0,2380 | 0,4135 | 0,3323 | 0,3869 | **0,6377** | **0,5478** | 0,2812 | 0,1853 | 0,0107 | **0,6922** | 0,4258 | -0,1717 | **0,5644** | **1** | | | | | | | | | | | | | | | | | | | | | | | | | | | | | | | | | | |
| **15** | -0,2261 | -0,0924 | -0,0966 | -0,2470 | 0,2084 | 0,3999 | 0,5523 | 0,3609 | -0,0353 | 0,2043 | 0,1983 | -0,2564 | 0,1988 | 0,1419 | **1** | | | | | | | | | | | | | | | | | | | | | | | | | | | | | | | | | |
| **16** | 0,0642 | 0,4941 | 0,4764 | **0,5515** | 0,4112 | -0,0249 | 0,2086 | **0,5496** | 0,4251 | 0,5222 | -0,2332 | -0,2933 | -0,0962 | 0,3671 | 0,2262 | **1** | | | | | | | | | | | | | | | | | | | | | | | | | | | | | | | | |
| **17** | 0,2462 | 0,4233 | 0,4703 | 0,3189 | **0,6051** | 0,2801 | 0,5287 | 0,4801 | 0,1457 | **0,6601** | 0,0960 | -0,3011 | 0,1892 | 0,4902 | 0,4152 | **0,7628** | **1** | | | | | | | | | | | | | | | | | | | | | | | | | | | | | | | |
| **18** | 0,2148 | **0,5711** | -0,0532 | 0,1769 | 0,2925 | 0,4191 | **0,5938** | 0,4966 | 0,0160 | 0,2735 | 0,0075 | -0,1743 | 0,0948 | 0,5027 | **0,6282** | **0,5765** | **0,5491** | **1** | | | | | | | | | | | | | | | | | | | | | | | | | | | | | | |
| **19** | 0,0051 | 0,1328 | 0,2783 | 0,1682 | **0,6456** | 0,4835 | 0,2332 | 0,2401 | -0,0406 | **0,6246** | 0,4267 | -0,3678 | **0,6303** | **0,7176** | 0,4251 | 0,2959 | 0,4963 | 0,3828 | **1** | | | | | | | | | | | | | | | | | | | | | | | | | | | | | |
| **20** | 0,2304 | 0,1229 | -0,1414 | -0,2732 | 0,1539 | 0,4350 | 0,4429 | -0,0761 | -0,1608 | 0,0376 | **0,5493** | -0,0537 | 0,4136 | 0,3515 | 0,4192 | -0,2717 | 0,1754 | 0,3397 | 0,1525 | **1** | | | | | | | | | | | | | | | | | | | | | | | | | | | | |
| **21** | 0,1581 | 0,3725 | 0,0693 | 0,3083 | **0,5900** | 0,4960 | 0,4265 | 0,4674 | 0,0445 | 0,4066 | 0,3903 | -0,4524 | 0,4642 | **0,8143** | 0,0846 | 0,3941 | 0,4091 | 0,4019 | **0,6781** | 0,1902 | **1** | | | | | | | | | | | | | | | | | | | | | | | | | | | |
| **22** | -0,2106 | -0,0609 | -0,4518 | -0,4467 | 0,0449 | 0,0546 | 0,5011 | 0,1271 | -0,3837 | -0,3260 | -0,4088 | -0,0944 | -0,2619 | -0,2164 | 0,1027 | -0,0386 | 0,0633 | 0,0658 | -0,1766 | 0,0830 | -0,0076 | **1** | | | | | | | | | | | | | | | | | | | | | | | | | | |
| **23** | 0,2342 | 0,4949 | -0,0480 | 0,2538 | **0,6338** | 0,3264 | **0,5532** | 0,4871 | -0,0064 | 0,2032 | 0,1280 | -0,5262 | 0,2286 | **0,6550** | 0,3364 | **0,5460** | 0,5220 | **0,6288** | **0,5806** | 0,2266 | **0,8647** | 0,1240 | **1** | | | | | | | | | | | | | | | | | | | | | | | | | |
| **24** | -0,0399 | 0,1472 | 0,0852 | 0,2906 | 0,3030 | **0,5511** | 0,4176 | 0,4275 | -0,0164 | **0,5938** | **0,7289** | -0,2971 | **0,6121** | **0,6727** | 0,4881 | 0,2394 | 0,3948 | 0,4478 | **0,6758** | 0,3981 | **0,6017** | -0,1306 | 0,4201 | **1** | | | | | | | | | | | | | | | | | | | | | | | | |
| **25** | 0,3279 | 0,3887 | 0,3155 | 0,2155 | 0,3451 | 0,1293 | -0,1099 | -0,0169 | 0,3123 | 0,1874 | 0,1178 | -0,2917 | 0,1551 | 0,5301 | -0,1430 | 0,2771 | 0,2126 | 0,2002 | 0,3437 | 0,1715 | **0,6040** | -0,3917 | **0,6157** | 0,0227 | **1** | | | | | | | | | | | | | | | | | | | | | | | |
| **26** | 0,1976 | 0,2322 | -0,1495 | 0,1695 | 0,5096 | **0,7071** | **0,6523** | 0,3939 | -0,3527 | 0,4235 | **0,5610** | -0,2188 | **0,5928** | **0,7252** | 0,2265 | 0,1165 | 0,3822 | 0,3025 | **0,6721** | 0,3198 | **0,8105** | 0,1036 | **0,6075** | **0,7409** | 0,2129 | **1** | | | | | | | | | | | | | | | | | | | | | | |
| **27** | 0,0130 | -0,0784 | 0,1600 | -0,1160 | 0,3936 | 0,3924 | 0,1224 | -0,0582 | 0,1392 | 0,2589 | **0,7371** | -0,0263 | **0,8726** | 0,4214 | 0,2542 | -0,2545 | 0,1201 | 0,0319 | **0,5404** | **0,6288** | 0,3909 | -0,2824 | 0,2075 | 0,4980 | 0,3072 | 0,4412 | **1** | | | | | | | | | | | | | | | | | | | | | |
| **28** | 0,0843 | 0,2090 | -0,4090 | -0,1318 | -0,3486 | -0,0725 | 0,1547 | -0,0366 | -0,4106 | -0,2660 | -0,2324 | -0,1206 | -0,3574 | -0,2701 | 0,3670 | 0,0011 | -0,0368 | 0,3213 | 0,0316 | 0,0014 | -0,2736 | 0,2898 | -0,0426 | 0,0390 | -0,2597 | -0,1311 | -0,3290 | **1** | | | | | | | | | | | | | | | | | | | | |
| **29** | 0,1783 | 0,3789 | 0,3725 | 0,4206 | **0,6845** | 0,3439 | 0,1013 | 0,1723 | 0,0096 | **0,6542** | 0,3283 | -0,2039 | **0,6325** | **0,7814** | 0,1974 | 0,4144 | 0,5111 | 0,4056 | **0,9329** | 0,0338 | **0,6900** | -0,2680 | **0,5617** | **0,6041** | 0,4151 | **0,6101** | 0,4525 | -0,0212 | **1** | | | | | | | | | | | | | | | | | | | |
| **30** | -0,2938 | -0,3214 | 0,1186 | -0,3356 | -0,1466 | 0,4214 | 0,3151 | 0,1538 | 0,2222 | 0,2724 | **0,5432** | -0,0400 | 0,3707 | -0,0204 | 0,4979 | -0,1229 | 0,2105 | 0,0219 | 0,1325 | **0,5328** | -0,0462 | -0,0247 | -0,1411 | 0,4720 | -0,1475 | 0,2123 | **0,5480** | 0,0434 | -0,0584 | **1** | | | | | | | | | | | | | | | | | | |
| **31** | 0,2309 | 0,4402 | 0,2187 | 0,5046 | 0,1167 | 0,4527 | 0,2619 | 0,2273 | -0,0093 | **0,6238** | 0,2501 | 0,0992 | 0,1605 | **0,6863** | 0,0919 | 0,4220 | 0,3602 | 0,4945 | 0,2427 | 0,1463 | 0,4084 | -0,2539 | 0,2682 | **0,5887** | 0,1737 | 0,4843 | -0,0690 | -0,0190 | 0,3585 | 0,0748 | **1** | | | | | | | | | | | | | | | | | |
| **32** | -0,3329 | -0,1538 | -0,3613 | -0,4695 | -0,0421 | 0,2515 | 0,5172 | 0,2048 | -0,2540 | -0,1601 | -0,3107 | 0,0495 | -0,1534 | -0,1353 | 0,0360 | -0,0655 | 0,0292 | 0,0479 | -0,2001 | 0,0815 | 0,0167 | **0,9257** | 0,0049 | -0,0250 | -0,4622 | 0,1786 | -0,2172 | 0,1194 | -0,2704 | 0,0990 | -0,0821 | **1** | | | | | | | | | | | | | | | | |
| **33** | -0,0568 | 0,1015 | -0,3728 | -0,0886 | 0,3501 | 0,4397 | **0,5671** | 0,2480 | -0,3752 | 0,0785 | 0,1465 | -0,1515 | 0,2935 | 0,3504 | **0,8112** | 0,1250 | 0,2026 | **0,6476** | **0,5541** | 0,2957 | 0,3382 | 0,1349 | 0,5317 | 0,5213 | -0,0248 | 0,4834 | 0,1889 | 0,4021 | 0,3928 | 0,1216 | 0,2220 | 0,0439 | **1** | | | | | | | | | | | | | | | |
| **34** | -0,0089 | 0,0435 | 0,2065 | 0,0005 | 0,2513 | 0,2593 | 0,3691 | 0,0209 | -0,1498 | 0,4517 | 0,3446 | -0,2313 | 0,1918 | 0,3896 | 0,3034 | 0,1329 | 0,4967 | 0,1613 | 0,2323 | **0,5640** | 0,2028 | 0,3388 | 0,1598 | **0,5387** | -0,1041 | 0,3927 | 0,2028 | 0,0825 | 0,1383 | 0,4871 | 0,3787 | 0,3388 | 0,1285 | **1** | | | | | | | | | | | | | | |
| **35** | -0,2548 | -0,0125 | 0,0005 | 0,0813 | 0,2220 | 0,4503 | **0,6016** | **0,6435** | 0,1615 | 0,3992 | **0,5465** | -0,2795 | 0,4649 | 0,3299 | **0,6251** | 0,3097 | 0,4479 | 0,4515 | 0,3882 | 0,3774 | 0,3946 | 0,1402 | 0,3326 | **0,8195** | -0,2394 | 0,5235 | 0,4059 | -0,0367 | 0,2655 | **0,6152** | 0,3148 | 0,2560 | 0,4474 | 0,4916 | **1** | | | | | | | | | | | | | |
| **36** | 0,2815 | 0,2755 | -0,3815 | -0,2350 | 0,4151 | 0,3894 | **0,8608** | 0,4020 | -0,3643 | -0,1210 | -0,0458 | -0,0460 | 0,1130 | 0,1216 | 0,3757 | 0,1348 | 0,4735 | 0,4539 | 0,0795 | 0,4170 | 0,2518 | **0,5899** | 0,4479 | 0,0525 | -0,0751 | 0,3909 | 0,1126 | -0,0117 | 0,0049 | 0,0845 | -0,0695 | **0,5362** | 0,3576 | 0,2215 | 0,3271 | **1** | | | | | | | | | | | | |
| **37** | 0,2275 | 0,2031 | 0,0946 | 0,0718 | 0,3316 | **0,5454** | 0,4461 | 0,0254 | -0,2303 | 0,4777 | 0,4576 | -0,0743 | 0,3251 | **0,6877** | 0,4768 | 0,0831 | 0,4063 | 0,5123 | 0,3640 | **0,7196** | 0,3419 | -0,0787 | 0,3534 | **0,6005** | 0,1708 | 0,5057 | 0,3098 | -0,0193 | 0,2871 | 0,2954 | **0,6534** | -0,0430 | 0,4830 | **0,6777** | 0,3907 | 0,2578 | **1** | | | | | | | | | | | |
| **38** | -0,2779 | -0,1942 | -0,1034 | -0,2662 | -0,0678 | 0,2693 | 0,1256 | -0,1107 | 0,0079 | 0,0356 | 0,4517 | -0,0477 | 0,3642 | 0,2443 | -0,0239 | -0,3716 | -0,2009 | -0,0923 | 0,0164 | **0,5936** | 0,2289 | 0,3127 | -0,0097 | 0,4343 | -0,0828 | 0,2998 | 0,4561 | -0,1313 | -0,0565 | 0,4427 | 0,1161 | 0,4413 | -0,0375 | **0,5760** | 0,4165 | 0,0092 | 0,3382 | **1** | | | | | | | | | | |
| **39** | -0,3317 | -0,3706 | -0,0588 | **-0,7757** | -0,1370 | **0,7419** | 0,5151 | 0,1528 | 0,0082 | 0,1639 | 0,1316 | 0,0068 | 0,1124 | 0,0752 | 0,3698 | -0,2520 | 0,1188 | 0,1778 | 0,0760 | 0,5299 | 0,0114 | 0,3654 | -0,0519 | 0,1441 | -0,1062 | 0,2192 | 0,3101 | 0,0181 | -0,1178 | **0,5918** | 0,0209 | 0,5289 | 0,1551 | 0,3375 | 0,2897 | 0,4194 | 0,3310 | 0,4273 | **1** | | | | | | | | | |
| **40** | 0,3452 | 0,3963 | -0,3543 | 0,0226 | 0,2980 | **0,5858** | **0,9268** | **0,6271** | -0,3414 | 0,1247 | 0,3631 | -0,2291 | 0,2792 | 0,3483 | 0,3530 | 0,2117 | **0,5417** | 0,4965 | 0,2871 | 0,4541 | 0,5126 | 0,4257 | 0,5163 | 0,5074 | -0,0400 | **0,7161** | 0,2446 | 0,0727 | 0,2283 | 0,3130 | 0,2711 | 0,4549 | 0,3655 | 0,4136 | **0,6214** | **0,8028** | 0,3738 | 0,2213 | 0,3784 | **1** | | | | | | | | |
| **41** | 0,2368 | 0,4842 | -0,0289 | 0,3590 | 0,3475 | 0,4357 | 0,3902 | 0,4980 | 0,0695 | 0,2749 | 0,2414 | -0,4245 | 0,2155 | **0,6737** | 0,0360 | 0,4613 | 0,3186 | 0,4480 | 0,5087 | 0,0468 | **0,9108** | -0,0895 | **0,8378** | 0,4702 | **0,6824** | **0,6854** | 0,1619 | -0,0892 | **0,5523** | -0,0982 | 0,4865 | -0,0792 | 0,3134 | 0,0238 | 0,2458 | 0,1420 | 0,2291 | 0,0406 | -0,0749 | 0,4384 | **1** | | | | | | | |
| **42** | -0,0264 | 0,0514 | 0,0540 | -0,0434 | 0,2892 | 0,2395 | 0,2647 | -0,0900 | -0,1584 | 0,2840 | **0,5474** | -0,1861 | 0,5153 | 0,4045 | 0,3163 | -0,0942 | 0,2750 | 0,1304 | 0,3844 | **0,6972** | 0,2971 | 0,2663 | 0,1957 | **0,6507** | -0,0389 | 0,4579 | **0,5593** | 0,1184 | 0,2889 | 0,5560 | 0,1849 | 0,2527 | 0,2338 | **0,8510** | **0,5558** | 0,1580 | **0,5622** | **0,7666** | 0,3011 | 0,3997 | 0,0682 | **1** | | | | | | |
| **43** | -0,0452 | -0,0176 | 0,5007 | 0,1056 | 0,2483 | 0,3886 | 0,1420 | 0,0254 | 0,1168 | **0,7288** | 0,5141 | -0,1797 | 0,3348 | **0,5943** | 0,3136 | 0,1731 | 0,4912 | 0,2326 | 0,3653 | **0,5585** | 0,2376 | -0,1131 | 0,1056 | **0,6399** | 0,0533 | 0,3525 | 0,3506 | -0,1397 | 0,3025 | 0,4871 | **0,5650** | -0,0128 | 0,1032 | **0,8505** | 0,4833 | -0,0229 | **0,8033** | 0,4870 | 0,3301 | 0,2060 | 0,0496 | **0,7068** | **1** | | | | | |
| **44** | -0,1146 | -0,0526 | -0,0672 | -0,3126 | 0,1143 | 0,3579 | 0,3585 | -0,0328 | -0,1919 | 0,1719 | 0,4246 | -0,2933 | 0,2966 | 0,3108 | 0,3527 | -0,1717 | 0,2049 | 0,1707 | 0,2914 | **0,7340** | 0,2457 | 0,4373 | 0,1891 | 0,5322 | -0,0490 | 0,3945 | 0,4396 | 0,2340 | 0,1387 | **0,5499** | 0,1137 | 0,4212 | 0,2453 | **0,8359** | 0,4806 | 0,2397 | **0,5502** | **0,7958** | 0,5224 | 0,4252 | 0,0561 | **0,9350** | **0,6533** | **1** | | | | |
| **45** | -0,1901 | -0,0748 | 0,0340 | -0,1320 | 0,0637 | 0,3958 | 0,1282 | -0,1144 | -0,1477 | 0,3354 | 0,5033 | -0,1668 | 0,4309 | 0,4630 | 0,1388 | -0,2106 | 0,0281 | 0,0591 | 0,4052 | 0,5233 | 0,3398 | 0,2163 | 0,0948 | **0,6593** | -0,0256 | 0,4876 | 0,4490 | 0,1619 | 0,3255 | 0,4454 | 0,3153 | 0,2979 | 0,1854 | **0,7133** | 0,4239 | -0,0961 | 0,4909 | **0,8397** | 0,3825 | 0,2442 | 0,1661 | **0,8723** | **0,6550** | **0,8779** | **1** | | | |
| **46** | -0,4383 | -0,2763 | 0,0960 | -0,1752 | -0,1344 | 0,3150 | 0,0640 | 0,0266 | 0,2811 | 0,2534 | **0,5619** | 0,1070 | **0,5516** | 0,1678 | 0,2394 | -0,2280 | -0,1074 | 0,0264 | 0,1428 | 0,4740 | 0,0701 | -0,0211 | -0,1836 | **0,5985** | -0,2367 | 0,2024 | **0,5896** | -0,0831 | 0,0708 | **0,7089** | 0,1726 | 0,1926 | 0,0695 | 0,4016 | **0,6461** | -0,1507 | 0,2512 | **0,7898** | 0,4204 | 0,1350 | -0,0839 | **0,6493** | 0,4886 | **0,5924** | **0,7045** | **1** | | |
| **47** | 0,3504 | 0,4283 | -0,4394 | 0,0182 | 0,3406 | **0,5484** | **0,9681** | **0,6040** | -0,4481 | 0,0667 | 0,2628 | -0,2795 | 0,1964 | 0,3548 | 0,4779 | 0,2500 | **0,5398** | **0,6065** | 0,3283 | 0,4389 | 0,5060 | 0,4721 | **0,6072** | 0,4944 | -0,0404 | **0,6978** | 0,1437 | 0,2146 | 0,2468 | 0,2050 | 0,2732 | 0,4348 | **0,5478** | 0,4017 | **0,5823** | **0,8259** | 0,4349 | 0,1301 | 0,3150 | **0,9644** | 0,4526 | 0,3705 | 0,1708 | 0,4146 | 0,2062 | 0,0162 | **1** | |
| **48** | -0,0635 | -0,0475 | 0,0379 | -0,2524 | 0,3179 | **0,5431** | 0,5018 | 0,1415 | -0,1059 | 0,3493 | **0,6065** | -0,3018 | 0,5270 | 0,4592 | 0,4529 | -0,0757 | 0,3990 | 0,2281 | 0,4649 | **0,7860** | 0,4173 | 0,2900 | 0,3210 | **0,6615** | 0,0565 | **0,5910** | **0,6543** | 0,0000 | 0,2937 | **0,6733** | 0,1650 | 0,3189 | 0,3081 | **0,8071** | **0,6390** | 0,3824 | **0,6215** | **0,7080** | **0,5806** | **0,5866** | 0,1835 | **0,9083** | **0,7054** | **0,9196** | **0,7845** | **0,6060** | **0,5386** | **1** |

**Supplemental Table S5.** PCA score values for controls and treated samples on the PC3.

| **Samples** | **PC3 (11%)** |
|---|---|
| Control 1 (C1) | -0.415 |
| Control 2 (C2) | -1.820 |
| Control 3 (C3) | -1.175 |
| Control 4 (C4) | -2.041 |
| Control 5 (C5) | -4.537 |
| Control 6 (C6) | -2.156 |
| Control 7 (C7) | -0.643 |
| Treated 1 (R1) | 1.915 |
| Treated 2 (R2) | 2.129 |
| Treated 3 (R3) | 1.576 |
| Treated 4 (R4) | 0.803 |
| Treated 5 (R5) | 0.429 |
| Treated 6 (R6) | 5.753 |
| Treated 7 (R7) | 0.182 |
